# microRNA-132/212 deficiency enhances Aβ production and senile plaque deposition in Alzheimer’s disease triple transgenic mice

**DOI:** 10.1038/srep30953

**Published:** 2016-08-03

**Authors:** Julia Hernandez-Rapp, Sara Rainone, Claudia Goupil, Véronique Dorval, Pascal Y. Smith, Martine Saint-Pierre, Maxime Vallée, Emmanuel Planel, Arnaud Droit, Frédéric Calon, Francesca Cicchetti, Sébastien S. Hébert

**Affiliations:** 1 Axe Neurosciences, CHU de Québec-Université Laval, Québec, QC, G1V4G2, Canada; 2 Département de psychiatrie et neurosciences, Université Laval, Québec, QC, G1V 0A6, Canada; 3 Axe Endocrinologie et néphrologie, CHU de Québec-Université Laval, Québec, QC, G1V4G2, Canada; 4 Département de médecine moléculaire, Université Laval, Québec, QC, G1V 0A6, Canada; 5 Faculté de pharmacie, Université Laval, Québec, QC, G1V 0A6, Canada

## Abstract

The abnormal regulation of amyloid-β (Aβ) metabolism (e.g., production, cleavage, clearance) plays a central role in Alzheimer’s disease (AD). Among endogenous factors believed to participate in AD progression are the small regulatory non-coding microRNAs (miRs). In particular, the miR-132/212 cluster is severely reduced in the AD brain. In previous studies we have shown that miR-132/212 deficiency in mice leads to impaired memory and enhanced Tau pathology as seen in AD patients. Here we demonstrate that the genetic deletion of miR-132/212 promotes Aβ production and amyloid (senile) plaque formation in triple transgenic AD (3xTg-AD) mice. Using RNA-Seq and bioinformatics, we identified genes of the miR-132/212 network with documented roles in the regulation of Aβ metabolism, including Tau, Mapk, and Sirt1. Consistent with these findings, we show that the modulation of miR-132, or its target Sirt1, can directly regulate Aβ production in cells. Finally, both miR-132 and Sirt1 levels correlated with Aβ load in humans. Overall, our results support the hypothesis that the miR-132/212 network, including Sirt1 and likely other target genes, contributes to abnormal Aβ metabolism and senile plaque deposition in AD. This study strengthens the importance of miR-dependent networks in neurodegenerative disorders, and opens the door to multifactorial drug targets of AD by targeting Aβ and Tau.

Alzheimer’s disease (AD) is a complex neurodegenerative disorder and the most common cause of dementia. One of the main pathological hallmarks of AD is senile plaques composed of Aβ peptides. These short 40–42 amino acid peptides are generated by proteolytic cleavage of Amyloid precursor protein (APP) by BACE1/β-secretase and PSEN/γ-secretase^1^. Mutations in *APP* or *PSEN* affect Aβ metabolism (e.g., production, cleavage, clearance) and cause rare forms of autosomal dominant early onset AD^2^. However, fairly little is known about the molecular mechanisms leading to Aβ pathology in the vast majority (>99%) of sporadic AD cases^1^. The identification of endogenous factors that lead to cerebellar Aβ accumulation *in vivo* is thus of high interest.

It is now well established that changes in gene expression occur during AD progression^3^. Such changes could occur prior to or in conjunction with Aβ deposition, for instance during the so-called cellular phase of AD^1^. Accumulating evidence suggests that the small (~22 nt) non-coding microRNAs (miRs), which participate in posttranscriptional gene expression regulation^4^, can contribute to AD pathogenesis by regulating genes such as APP^5^ or BACE1/β-secretase^6^,^7^. These results add to the growing number of findings showing that miR profiles and networks are misregulated in AD^8^,^9^. In addition, recent evidence from genetic studies suggests that polymorphisms that affect miR target binding can contribute to disease risk^10^,^11^,^12^. However, while these proof-of-concept experiments are interesting, experimental support from animal studies is largely lacking. This is an important issue as endogenous miRs control biological function(s) most often *via* several target genes, particularly in mammals^13^. Thus, understanding the global impact of miRs on neuronal networks could help understand the cause and effect relationship between miR dysregulation and AD^14^.

The miR-132/212 cluster is located on chromosome 17 in humans (11 in mice) and encodes two highly related family members that are downregulated in AD^15^,^16^,^17^,^18^. Mounting evidence suggests that miR-132 and miR-212 play an important role in synaptic plasticity, neurite outgrowth, and memory formation^19^,^20^,^21^, all of which are disturbed in AD. Recently, we have shown that miR-132/212 deficiency in mice caused abnormal Tau hyperphosphorylation and aggregation, another hallmark of AD^16^. Whether the loss of miR-132/212 also participates in the molecular events leading to Aβ deposition is an interesting possibility, and would strengthen a multiple hit scenario for AD^6^,^22^.

With this in mind, we explored the impact of miR-132/212 loss on Aβ generation and senile plaque deposition in 3xTg-AD mice, a recognized AD mouse model^23^. These experiments showed that miR-132/212 deficiency caused a substantial increase in cerebral Aβ levels as well as amyloid plaque load. We identified a number of candidate miR-132/212 target genes previously implicated in the regulation of Aβ metabolism, such as Sirt1, providing a mechanism for the observed effects *in vivo*. Previous studies have shown that Sirt1 (also known as Sirtuin 1), a nicotinamide adenine dinucleotide-dependent deacetylase can, in turn, regulate Aβ production or downstream signaling events by targeting genes such as ROCK1^24^, AMPK^25^, NF-κB^26^, or ADAM10^27^. Finally, we provide correlative evidence for a physiological link between miR-132, Sirt1, and Aβ in humans. Collectively, our results add to the literature by providing clear *in vivo* evidence that the disruption of miR networks can promote Aβ accumulation and deposition, and help define how a single miR can contribute to AD neuropathogenesis and dementia.

## Results

### miR-132/212 deficiency in mice promotes Aβ pathology

**Figure 1:**
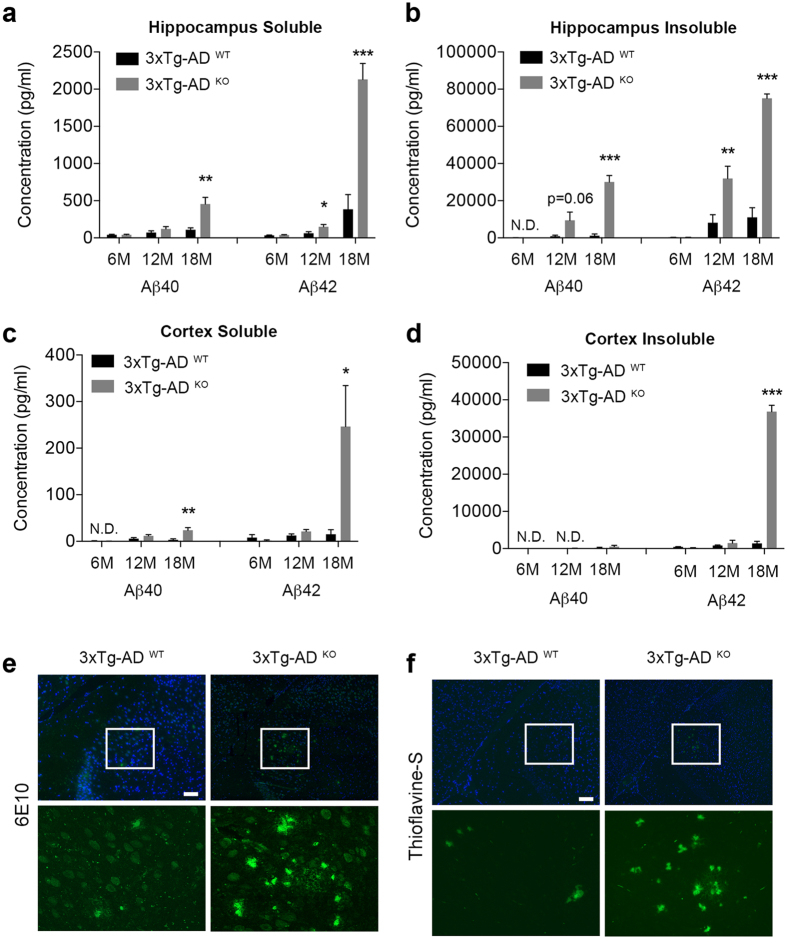
Analysis of Aβ metabolism in miR-132/212-deficient AD mice. **(a,b)** ELISA of human Aβ40 and Aβ42 in hippocampal samples of 3xTg-AD^WT^ and 3xTg-AD^KO^ mice (n = 8–10 mice for each group, mixed gender). 6M; 6 month-old, 12M; 12 month-old, 18M; 18 month-old. **(c,d)** ELISA of human Aβ40 and Aβ42 in cortical samples of 3xTg-AD^WT^ and 3xTg-AD^KO^ mice (n = 8–10 mice for each group). 6M; 6 month-old, 12M; 12 month-old, 18M; 18 month-old. **(e)** Immunohistochemical detection of Aβ plaques in the forebrains of 18 month 3xTg-AD^WT^ and 3xTg-AD^KO^ mice (n = 3 mice for each genotype). Scale bar: 100 μm. **(f)** Thioflavine-S-positive stainings of Aβ plaques in the forebrains of 18 month 3xTg-AD^WT^ and 3xTg-AD^KO^ mice (n = 3 mice for each group). Scale bar: 100 μm. Data are presented as mean ± SEM. *P ≤ 0.05, **P ≤ 0.01, ***P ≤ 0.001 (Student’s t-test).

In this study, we used miR-132/212 knockout mice^28^ that we crossed with triple transgenic AD (3xTg-AD) mice^23^. Owing to the expression of PSEN1 (PS1M146V), APP (APPSwe) and Tau (TauP301L) transgenes, these latter mice progressively develop Aβ and Tau pathologies along with cognitive deficits. We have recently shown that miR-132/212 deficiency in 3xTg-AD mice leads to enhanced Tau pathology and memory impairment, which can be rescued in part by the reintroduction of miR-132 mimics^16^. Cortical and hippocampal tissues were isolated from 3xTg-AD mice with (3xTg-AD^WT^) or without (3xTg-AD^KO^) the miR-132/212 cluster (see Methods). Starting at 12 months of age, we observed an increase of (RIPA-soluble and guanidine-insoluble) Aβ40 and Aβ42 levels in 3xTg-AD^KO^ mice when compared to aged-matched controls, as determined by sensitive ELISA (Fig. 1a–d). At 18 months of age, a drastic increase of all Aβ species studied was evident in both brain regions. The most prominent changes occurred in the insoluble tissue fractions with up to 10-fold increases in Aβ42, consistent with its aggregation-prone properties. This is in agreement with an increase in Aβ plaque load in 18-month-old mice, as determined using Aβ (6E10) antibody and thioflavin-S stainings (Fig. 1e,f). Finally, the amount of endogenous (murine) soluble Aβ42 in the hippocampus was significantly increased in 18-month-old 3xTg-AD^KO^ mice when compared to controls (see Supplementary Fig. S1). Thus, loss of miR-132/212 in mice promotes Aβ production, aggregation and deposition.

### Identification of miR-132 gene networks *in vivo*

**Figure 2:**
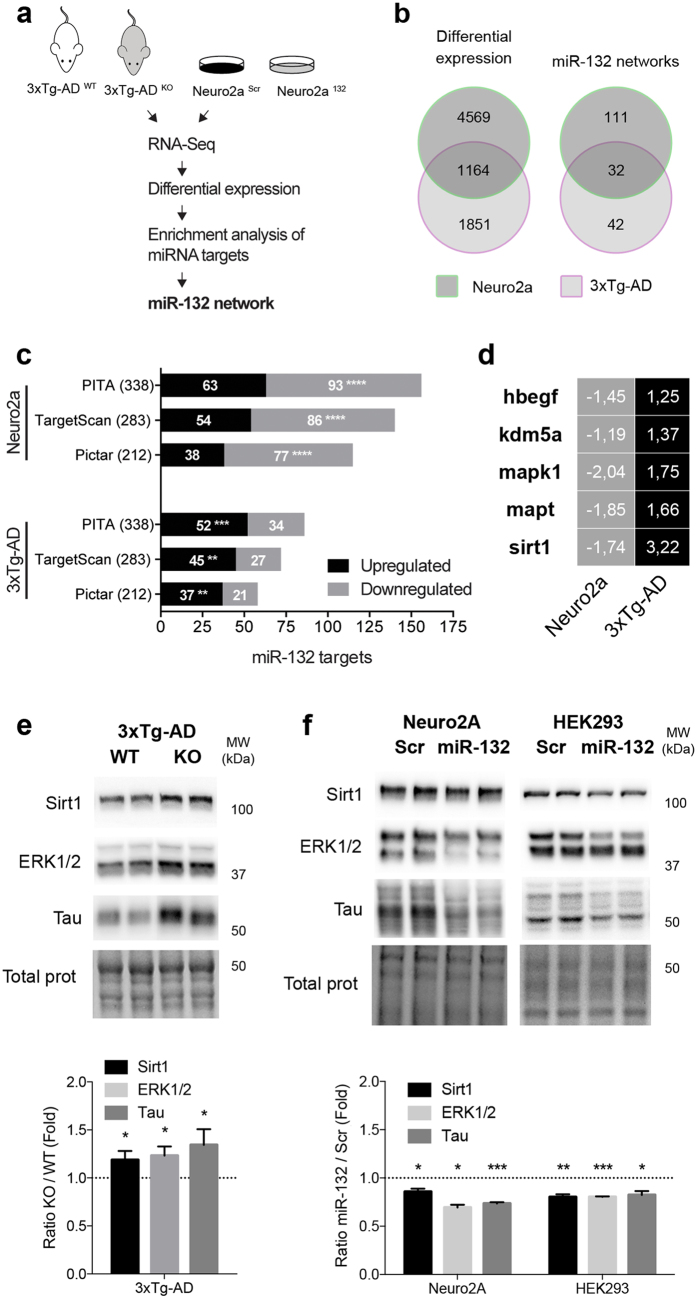
Identification of miR-132/212 targets *in vivo*. **(a)** Schematic overview of RNA-Seq experiments (n = 4/mice or cultures for each group). **(b)** Venn diagram demonstrating changes of mRNA transcripts between 3xTg-AD and Neuro2a models. Left panel: significant (ANOVA, P < 0.05) changes in expression between mice and cells. Right panel: overlap of transcripts (targets) between miR-132/212 networks in mice and cells. **(c)** Bioinformatics analysis of miR-132/212 targets in up- or down-regulated set of genes. **(d)** Heatmap analysis of validated miR-132/212 targets in 3xTg-AD and Neuro2a systems. **(e)** Western blot analysis of Sirt1, ERK1/2, and Tau in 3xTg-AD^WT^ and 3xTg-AD^KO^ mice (n = 10 mice for each group). Total proteins were used as normalization control. **(f)** Western blotting of Sirt1, ERK1/2, and Tau in native Neuro2a and HEK293 cells following miR-132 transfection (n = 3 cultures in triplicate per group). Total proteins were used as normalization control. In (**b**), data are presented as P values *P ≤ 0.05, **P ≤ 0.01, ***P ≤ 0.001, ****P ≤ 0.0001 (Fisher’s exact test). In (**d,e**), data are presented as mean ± SEM. *P ≤ 0.05, **P ≤ 0.01, ***P ≤ 0.001, ****P ≤ 0.0001 (Student’s t-test). Full-length blots/gels are presented in Supplementary Fig. S6.

Evidence from animal studies suggests that miRs coordinate the expression of related gene networks^29^,^30^,^31^. To uncover miR-132 networks, we performed genome-wide transcriptomics using RNA sequencing (RNA-Seq). Previous results have shown that miRs function mainly (initially) at the mRNA level^32^,^33^,^34^. RNA was isolated from hippocampi of 12 month-old 3xTg-AD mice (Fig. 2a). For comparative purposes, we also included RNA from Neuro2a cells treated with miR-132 mimics (Neuro2a^132^) or a scrambled control (Neuro2a^Scr^). Information about RNA-Seq reads (raw and mapped) is provided in Supplementary Table S1. Differentially expressed genes were determined by an ANOVA (Partek Genomics Suite, p < 0.05). In AD mice, we identified a total of 2847 genes (3311 transcripts) that were misregulated in the absence of miR-132/212 (Fig. 2b and Supplementary Table S2). In Neuro2a cells overexpressing miR-132, we identified 4996 genes (5772 transcripts) that were misregulated using the same criteria.

We next performed a miR target gene enrichment analysis using ToppGene Suite^35^. We found a significant enrichment of miR-132 targets in the set of upregulated (but not downregulated) genes in 3xTg-AD^KO^ mice (Fig. 2c). Note that the prediction tools (PITA, TargetScan, Pictar) made no distinction between miR-132 and miR-212 targets, as both miRs share the same seed sequence^20^. As expected, we observed a strong enrichment of miR-132 targets in the set of downregulated genes in Neuro2a^132^ cells. The miR-132 network comprised a total of 74 genes (as defined by PITA, TargetScan and Pictar) in 3xTg-AD mice and 143 genes in Neuro2a cells (Fig. 2b and Supplementary Table S3). Overall, a total of 32 targets were found in common between the 2 networks.

Using GeneMANIA^36^, we found that most of miR-132 targets were highly interconnected (see Supplementary Fig. S2). Gene ontology (GO) terms related to miR-132 networks included *neuron projection development* (GO: 0031175), *negative regulation of transcription* (GO: 0000122), and *regulation of protein phosphorylation* (GO: 0001932), all of which are important for brain function and maintenance (see Supplementary Table S4). Notably, among the 32 targets shared between 3xTg-AD and Neuro2a networks, 5 have previously been experimentally validated^37^, including *hbegf*, *kdm5a*, *mapk1*, *mapt*, and *sirt1* (Fig. 2d). These genes were inversely expressed, reflecting either loss or gain of miR function.

Strikingly, 3 out of 5 validated miR-132 targets have previously documented roles in the regulation of Aβ metabolism and/or pathology, including Sirt1, MAPK/ERK, and Tau (see Discussion). By Western blot, we confirmed the upregulation of endogenous Sirt1, Mapk1/ERK2 and Tau in 3xTg-AD^KO^ mice when compared to controls (Fig. 2e). Accordingly, all genes were downregulated upon miR-132 overexpression in Neuro2a cells (Fig. 2f). We validated these latter observations in human HEK293 cells (Fig. 2f), therefore avoiding cell-type specific effects. In these latter conditions ectopic miR-132 levels reached ~600 fold over endogenous levels (see Supplementary Fig. S3). Taken together, our studies identified a number of candidate effector genes implicated in the regulation of Aβ metabolism, providing a potential mechanism for the effects observed in mice.

### Regulation of Aβ production by miR-132

**Figure 3:**
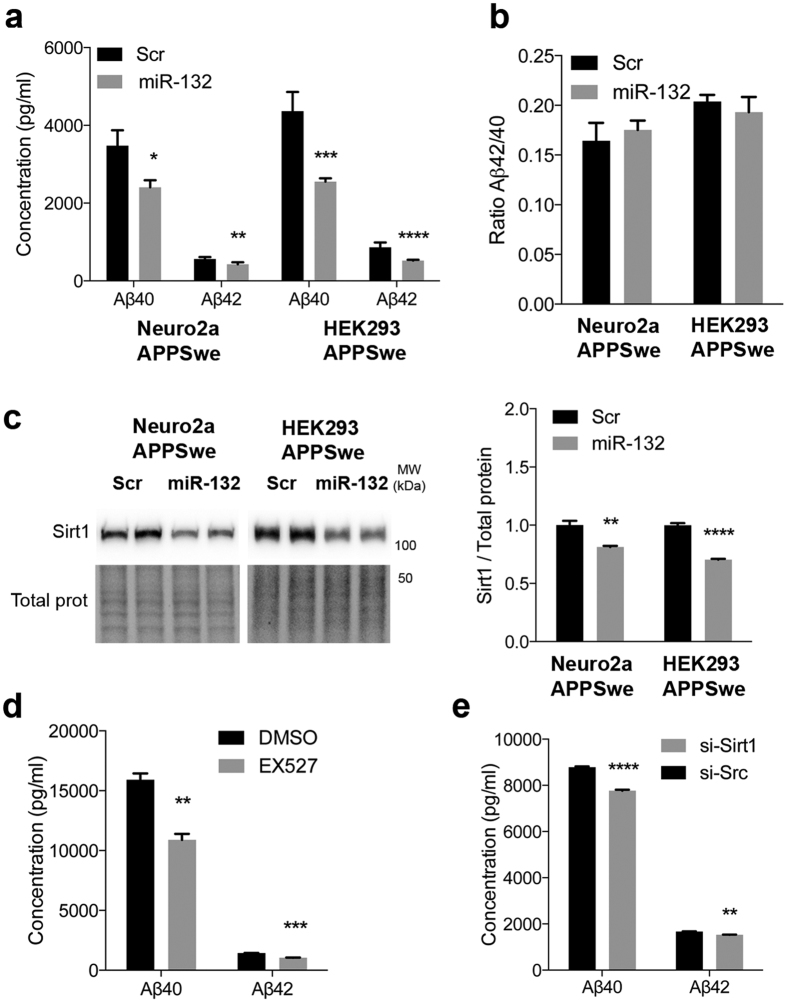
Regulation of Aβ by miR-132 and its target Sirt1. **(a,b)** ELISA of human Aβ40 and Aβ42 in Neuro2a-APPSwe and HEK293-APPSwe cells after miR-132 treatment (n = 2 cultures in triplicate per group). **(c)** Western blot analysis of Sirt1 in Neuro2a-APPSwe and HEK293-APPSwe cells following miR-132 transfection (n = 2 cultures in triplicate per group). Total proteins were used as normalization control. **(d,e)** ELISA of human Aβ40 and Aβ42 in HEK293-APPSwe cells after Sirt1 pharmacological inhibition (EX527) or genetic downregulation (si-Sirt1) (n = 2 cultures in triplicate per group). Data are presented as mean ± SEM. *P ≤ 0.05, **P ≤ 0.01, ***P ≤ 0.001, ****P ≤ 0.0001 (Student’s t-test). Full-length blots/gels are presented in Supplementary Fig. S6.

We next asked if miR-132 can directly regulate Aβ production in cells. To this end, we introduced miR-132 mimics in Neuro2a and HEK293 cells stably expressing human APPSwe (Neuro2a-APPSwe, HEK293-APPSwe)^7^. In both cell lines, miR-132 caused a significant downregulation of (soluble) human Aβ40 and Aβ42 levels as determined by ELISA (Fig. 3a). No significant changes in Aβ42/40 ratios were observed in these conditions (Fig. 3b). Considering the well characterized functional link between miR-132 and Sirt1^15^,^38^,^39^,^40^,^41^, we focused our efforts on this target gene. We first confirmed Sirt1 downregulation upon miR-132 overexpression in Neuro2a-APPSwe and HEK293-APPSwe cells (Fig. 3c). In another set of experiments, pharmacological inhibition of Sirt1 activity in HEK293-APPSwe cells caused a significant reduction of Aβ40 and Aβ42 levels (Fig. 3d). Knockdown of endogenous Sirt1 using a small interfering RNAs (siRNAs) produced similar effects (Fig. 3e). Here, we observed a 55% downregulation of Sirt1 upon siRNA treatment (see Supplementary Fig. S3). Again, no changes in Aβ42/40 ratios were observed in these conditions (see Supplemental Fig. S3). The fact that Sirt1 inhibition does not totally reproduce the effects of miR-132 on Aβ40 and Aβ42 (88.4% vs. 55.1%, and 91.8% vs. 51.9%, respectively) supports a cooperative mode of action of the miR-132 network.

### Correlation between miR-132 and Aβ in humans

**Figure 4:**
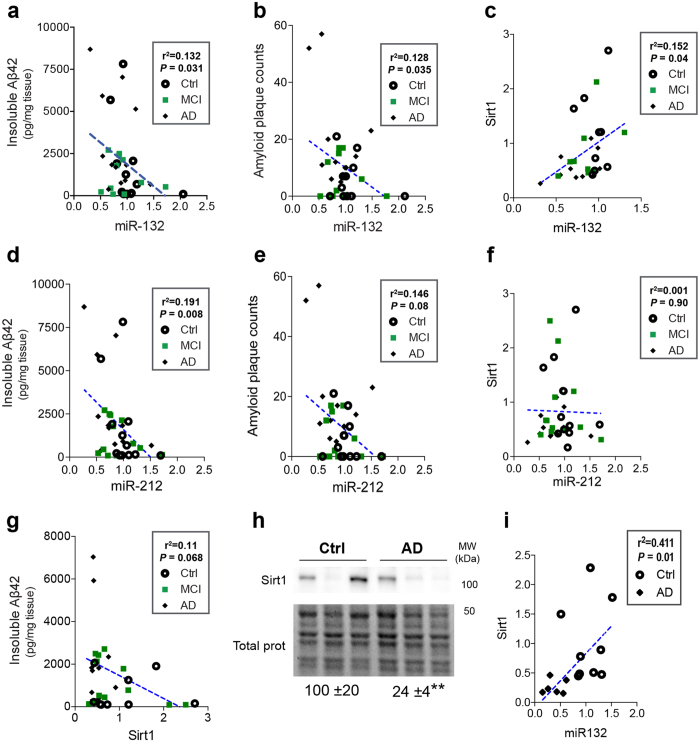
Clinical association between miR-132 and Aβ in Alzheimer’s disease. **(a–c)** Correlation between miR-132 expression levels, insoluble Aβ42, Aβ plaque load in the ROS cohort and Sirt1 protein levels (n = 10–12 cases/group). **(d–f)** Correlation between miR-212 expression levels, insoluble Aβ42, Aβ plaque load in the ROS cohort and Sirt1 protein levels (n = 10–12 cases/group). **(g)** Correlation between Sirt1 protein levels and insoluble Aβ42 levels in the ROS cohort (n = 9–10 cases/group). **(h)** Western blot analysis of Sirt1 in non-demented controls and AD patients in the Douglas Bell Canada brain bank cohort (n = 8–11/group). Total proteins were used as normalization control. **(i)** Correlation between Sirt1 protein levels and miR-132 in the Douglas Bell Canada brain bank cohort (n = 6–9 cases/group). In (**a–g,i**) data are presented as exact P values (linear regression model). In (**h**) data are presented as P values, **P ≤ 0.01 (Mann-Whitney U test). Full-length blots/gels are presented in Supplementary Fig. S6.

We finally asked if miR-132 could be clinically related to Aβ. For this, we used our published data from the Religious Orders Study (ROS)^16^. In this cohort, miR-132 (and miR-212) levels are lower in mild cognitive impairment (MCI) and AD cases compared to non-demented controls, and correlate with memory deficits. We found a significant correlation between miR-132, insoluble Aβ42 levels (Fig. 4a), and amyloid plaque count in all cases (Fig. 4b). Similar results were obtained with miR-212 (Fig. 2d,e). Previously we have shown that Sirt1 levels are lower in AD cases from the same cohort^42^. Sirt1 correlated with miR-132 (Fig. 4c) as well as insoluble Aβ42 (Fig. 4g). On the other hand, miR-212 did not correlate with Sirt1 (Fig. 4f). In an independent cohort (Douglas Bell Canada brain bank)^16^, we confirmed a decrease of Sirt1 in AD brains (Fig. 4h), and also correlated with miR-132 (Fig. 4i). Notably, no correlation was found between miR-132 (or miR-212), Sirt1 and Aβ in individual groups (controls, MCI, AD) (see Supplementary Table S5). Lastly, neither miR-132/212 levels nor the other settings (insoluble Aβ42, amyloid plaques, Sirt1) correlated with age of death of patients (see Supplementary Fig. S5).

## Discussion

The goal of this study was to determine the impact of miR-132/212 loss on Aβ metabolism. This is a direct follow-up of our previous work linking miR-132 function to Tau pathology in AD. Here we provide clear *in vivo* evidence that noncoding RNAs such as miRs are implicated in Aβ production and deposition in mice. Importantly, we identified a number of miR-132 targets with documented roles in the regulation of Aβ metabolism, providing a potential mechanism for the effects observed in cells, mice and humans. Combined with its established role in memory^16^,^19^,^21^,^43^, these results highlight the importance and multifaceted nature of miR-132 (and possibly miR-212) in AD, and set the stage for detailed functional studies.

We have previously shown that miR-132/212 deficiency in mice promoted Tau hyperphosphorylation and aggregation^16^. These effects were associated, in part, to a direct regulation of Tau (*mapt*) at the mRNA level. The RNA-Seq experiments presented herein confirm and extend these observations. Interestingly, it was shown that Tau deletion can attenuate both Aβ pathology and toxicity in mice^44^,^45^,^46^. Whether an increase of Tau alone is sufficient to promote Aβ pathology in our mouse model is certainly a possibility, but will require further investigation as it may involve various intracellular pathways^45^,^46^,^47^.

Sirt1 can deacetylate histone and non-histone proteins and other transcription factors, and is involved in the regulation of many physiological functions, including cell senescence, gene transcription, energy balance, and oxidative stress. It is perhaps not surprising that Sirt1 dysfunction is associated with neurodegenerative disorders, and in particular AD^48^. The mechanisms underlying the protective effect of Sirt1 in AD models are complex and multifaceted^48^,^49^. The effects of Sirt1 might act upstream and downstream of Aβ^50^,^51^. While an increase of Sirt1 is typically related to neuroprotection, we cannot exclude compensatory mechanisms in the 3xTg-AD mouse model. Consistent with this hypothesis, Sirt1 is downregulated in human AD samples, alongside of miR-132. Another interesting observation is a physiological “switch” between miR-132 and Sirt1 expression levels during aging^40^, which could be affected is disease conditions. Sirt1 activity was recently implicated in Tau pathology as well^52^.

Another interesting miR-132 target is Mapk1/ERK2. Usually, abnormal regulation of ERK/MAPK is associated with Aβ-induced downstream signaling events^53^. However, ERK/MAPK may also function upstream of Aβ production by regulating BACE1^54^. A role for ERK in regulating Tau phosphorylation is also plausible. The fact that Tau is hyperphosphorylated in miR-132/212 knockout mice^16^ is consistent with this hypothesis. It is likely that several genes or pathways, in addition to Tau, Sirt1 and ERK, also participate in the regulation of Aβ metabolism in our mouse model, such as autophagy impairment^16^,^55^.

It is notable that most (~97%) genes affected in our mouse or cell systems do not harbor miR-132/212 binding sites. These data are consistent with previous miR gain- or loss-of-function systems^13^,^56^, and likely result from various compensatory or regulatory mechanisms^57^. One example is a feedback loop between miR-132, BDNF and CREB^20^,^57^, a pleiotropic transcription factor involved in cell proliferation and survival. We have shown that CREB (and BDNF) is indeed affected in miR-132/212 knockout mice^19^, consistent with our RNA-Seq results. As hinted above, other examples include Sirt1 and Tau that could also serve as “master effectors” of biological processes, which seems a common feature of miR function^13^,^58^.

Nearly 25% (32/144) of genes in the miR-132 networks were found in common between 3xTg-AD^KO^ mice and Neuro2a^132^ cells. Thus, while offering a good tool to find *bona fide* miR targets, overexpression studies (mimics or inhibitors) present some limitations^13^ and should be considered with caution. It is clear that a combination of approaches [e.g., HITS-CLIP^59^ and RISC-trap^60^] will help determine the precise number of miR-132 targets *in vivo*. This task is even more challenging as miR-132 targets likely change according to age, gender, cell type, species, and/or disease state. Interestingly, close to 40% (1057/2847) of affected genes in the 3xTg-AD^KO^ mice were also changed in forebrain-specific miR-132/212 adult knockout mice^43^.

In attempt to provide clinical support for our observations, we first observed a good correlation between miR-132 and Sirt1 (protein) in humans. Positive miR:target correlations *in vivo* have been documented before^61^, including between miR-132 and Sirt1 in adults^40^. At first glance, this may seem contradictory to our results in mice and cells; however, we anticipate many compensatory mechanisms in the human AD brain that would compromise the delicate balance between miR-132 and Sirt1 regulation. Interestingly, Sirt1 mRNA levels (negatively) correlate with miR-212 in AD^62^ (in contrast to our western blot data) and corroborates a multi-layered regulation of Sirt1 expression. We also found a significant correlation between miR-132 and Aβ suggesting a clinical relationship between miR-132 levels and AD progression. It should be stressed, however, that MCI does not necessarily reflect prodromal AD, although Aβ accumulation does occur in a subset of MCI patients^63^. At this moment, the physiological meaning of this correlation is uncertain, as miR-132 could be implicated in various steps of Aβ deposition (including propagation) by targeting various genes during disease progression. It is interesting to note that miR-132/212 loss has a particularly strong impact on (mouse and human) Aβ42 production and aggregation, an effect related to yet unknown mechanisms. One caveat of this study is the rather small sample size of each patient group (non-demented controls, MCI, AD) in our cohorts (see Supplemental Table S5). Indeed, power analysis explains in part the loss of significance of these correlations when the groups are analyzed separately (Hébert S.S., unpublished observations). Thus, additional studies with a higher number of patients are necessary to draw definitive conclusions with regard to the clinical link between miR-132/212 and Aβ.

In conclusion, we provide strong evidence that the miR-132/212 network controls various aspects of AD pathologies in mice, including Aβ pathology (herein), Tau pathology, and memory impairments. Next steps include to identify key miR-132/212 target genes and to evaluate the precise role of miR-132/212 networks in various physiological and pathological contexts. The diagnostic^64^ and therapeutic applicability of miR-132 replacement therapy in AD is also an interesting possibility and warrants future investigation.

## Methods

### Study approval

All human studies were performed in accordance with the CHU de Québec – Bureau de l′éthique de la recherche guidelines and regulations, and approved by the same committee. Informed consent was obtained from all human subjects prior to donation and experimentation. All mouse studies were performed in accordance with the Université Laval ethics guidelines and regulations, and approved by the VRRC Comité de protection des animaux committee.

### Post-mortem tissues

Brain tissue from the first cohort of patients came from the Religious Orders Study, Chicago, USA, which consisted of non-demented controls, mild cognitive impairment and AD cases, based on detailed clinical diagnosis. Patient-related data and detailed clinical diagnosis criteria can be found elsewhere^16^,^42^. Brain tissue from the second, independent cohort of patients came from the Douglas Bell Canada Brain Bank, Montreal, Canada, and included non-dementia controls and AD cases, based on neuropathological diagnosis. Patient information is available elsewhere^16^,^65^,^66^. Blocks of tissue from temporal cortex were dissected and snap frozen in liquid nitrogen until use.

### Mice

The generation of 3xTg-AD mice lacking the miR-132/212 cluster has been described previously^16^. Knockout mice (mixed gender) were compared with age-matched controls. Mice were sacrificed by decapitation and the brains were removed, dissected on ice, and frozen on dry ice. All tissues were stored at −80 °C until use. For immunohistochemistry, after decapitation, brains were fixed with 4% paraformaldehyde and embedded in paraffin until use.

### Cell culture and transfection

Mouse Neuro2a cells (#CCL-131, ATCC, USA), mouse Neuro2a cells expressing the Swedish mutant of APP and Δ9 mutant of PSEN1 (Neuro2a APPSwe/Δ9) (kind gift from Dr. Gopal Thinakaran, U. Chicago, USA), human HEK293T cells (#LV900A, System Biosciences, CA, USA), and human HEK293 cells expressing the Swedish mutant of APP (HEK293-APPSwe) (kind gift from Dr. Bart De Strooper, KUL, Belgium) were cultured in Dulbecco’s modified Eagle medium supplemented with 10% fetal bovine serum. 200 000 cells (Neuro2a and N2A APPSw/Δ9) or 180 000 cells (HEK293 and HEK293-APPSwe) were seeded into 6-well plates. The next day, cells were transfected with 50 nM of miRNA mimics (Pre-miR miRNA precursor molecules (#AM17100, Life Technologies, Carlsbad, CA, USA) or 100 nM of siRNA against human Sirt1 (#23411, Dharmacon ON-TARGET*plus* SMART pool, Lafayette, CO, USA) using Lipofectamine^®^ 2000 (Life Technologies). A scrambled miRNA mimic (#AM17110, Life technologies) and scrambled siRNA oligonucleotide (#D-001810-10-05, Dharmacon ON-TARGET*plus*) were used as negative controls. Forty-eight hours post-transfection, cells were processed for RNA Sequencing, ELISA or Western Blotting. Sirt1 inhibition was carried out using EX-527 (#E7034, Sigma, St Louis, MO, USA) at 80 μM during six hours.

### Western Blotting

Protein extraction from cells, mice and humans was performed as before^16^. Ten micrograms of protein from each sample were separated by SDS-PAGE with 10% Tris-Glycine eXtended (TGX) Stain-Free™ polyacrylamide gels (Bio-Rad, Hercules, CA, USA). Stain-Free™ gels were activated by UV transillumination for 5 min using the Fusion FX5 imaging system (Vilbert Lourmat, France). Proteins were transferred to nitrocellulose membranes (Bio-Rad) and total proteins were visualized under UV using the Fusion FX5 imaging system. Bound antibody was revealed by enhanced chemiluminescence detection using a secondary antibody coupled to ImmobilonTM Western Chemiluminescent HRP Substrate (EMD Millipore, Billerica, MA, USA). The Fusion FX5 imaging system was used for immunoblot visualization. Band intensities were quantified using ImageJ 1.6 (http://imagej.nih.gov/ij/) software and normalized to the total amount of protein per lane.

### ELISA

RIPA extraction (defined as soluble) and guanidine extraction (defined as insoluble) proteins were obtained from mice hippocampus and cortex. Cells supernatants were collected 48 hours post-transfection or 6 hours post-drug-treatment. Human and mouse Aβ1–40 and Aβ1–42 levels were measured by ELISA following the manufacture’s protocol (#KHB3481 and #KHB3441 for the human, #KMB3481 and #KMB3441 for the mouse, Invitrogen, Waltham, MA, USA).

### Immunohistochemistry

Five-micrometer serial sections from paraffin blocks of mouse brain samples were collected with a microtome. To visualize the Aβ plaques in mice an immunostaining with Aβ-6E10 and with the Thioflavine-S was performed. After rehydration, slices were incubated in the antigen retrieval solution (DAKO, Glostrup, Denmark) at 95 °C for 25 minutes followed by the blocking solution (7.5% NGS; 0.4% Triton; 1% BSA; PBS) for 2 h and in Aβ-6E10 antibody solution (5% NGS; 0.4% Triton; PBS) overnight at 4 °C. Slices were incubated 2 h 30 minutes at RT in the secondary antibody solution and 5 minutes in 30 nM DAPI. For the Thioflavine-S, slices were rehydrated, incubated in Thioflavine-S 0,1% for 3 minutes, rinsed with EtOH 70% and cover-slipped with Fluoromount mounting media. As a control for specificity of antibodies, some sections were treated as described except that the primary antibody was omitted from the incubation medium. Slices were observed using a Zeiss AxioImager M2 microscope and images were processed with a computerized image analysis system (ZEN 2012 SP2 Software, Zeiss).

### miRNA quantification

Total RNA from human, mouse and cell samples was extracted using Trizol^®^ Reagent (Life technologies) according to the manufacturer’s instructions. TaqMan^®^ miRNA assay (Life Technologies) was used for miR-132 (#000457) quantifications following manufacturer’s protocol. RNU48 (#001006) and RNU19 (#000391) were used as normalization control. Relative expression was calculated by the 2^−ΔΔCT^ methods as before^65^.

### RNA sequencing

Total RNA was extracted from Neuro2a cells (mimics or control) and dissected hippocampi of mice (7 females and 1 male) using Trizol^®^ (Life technologies). Illumina TruSeq RNA Sample Preparation kit version 2 (Low Sample Protocol) was used on 1ug of total RNA. PolyA^+^ containing RNA molecules were purified using oligo-dT attached magnetic beads. Chemical fragmentation followed after two rounds of enrichment for PolyA^+^ mRNA. cDNA was synthesized using reverse transcriptase (Superscript II) and random primers. This was followed by second strand cDNA synthesis, end repair process, adenylation of 3′ ends and ligation of the adapters (single index). The products were then purified with Agencourt Ampure XP SPRI beads (Beckman Courter) and enriched with 15 cycles of PCR to create the cDNA library. Library PCR product was analyzed for appropriate size distribution with the Agilent Tapestation 2200, and quantitated using Qubit and qPCR before equimolar pooling of 12 samples. Libraries were subjected to an indexed SR sequencing run of 1 × 50 cycles on an Illumina HiSeq 2500. Raw sequencing data was demultiplexed using bcl2fastq 1.8.4. Quality trimming and size check of the raw reads has been performed using Trimmomatic 0.33^67^. Trimmed reads have then been mapped to mouse genome (mm10) with TopHat 2.0.14^68^. Mapped reads (10 reads cut-off) were analyzed for differential expression of transcripts (based on Ensembl genome browser 83) using Partek Genomics Suite (v6.6). The RNA-Seq data from this publication have been submitted to the GEO database (http://www.ncbi.nlm.nih.gov/geo/) and assigned the identifier [GSE84481].

### Antibodies

ERK1/2 (#4696) was purchased from Cell Signaling. Total tau (#A0024) was purchased from DAKO. SIRT-1 (#A21993) was purchased from Life Technology. SIRT-1 (#15404) was purchased from Santa Cruz (Dallas, TX, USA). Aβ-6E10 (#SIG-39320) was purchased from Covance (Princeton, NJ, USA). As secondary antibodies, goat anti-Mouse Alexa 488 (#A110290) was purchased from Life Technology, horseradish peroxidase (HRP) conjugated goat anti-Rabbit IgG (111-035-003) and goat anti-Mouse (115-035-003) IgG were purchased from Jackson ImmunoResearch (West Grove, PA, USA).

## Additional Information

**How to cite this article**: Hernandez-Rapp, J. *et al*. microRNA-132/212 deficiency enhances Aβ production and senile plaque deposition in Alzheimer’s disease triple transgenic mice. *Sci. Rep*. **6**, 30953; doi: 10.1038/srep30953 (2016).

## Supplementary Material

Supplementary Information
