# Supplementary material for: microRNA-132/212 deficiency enhances Aβ production and senile plaque deposition in Alzheimer’s disease triple transgenic mice
Source: Sci Rep. 2016 Aug 3;6:30953. doi: 10.1038/srep30953 (PMC4971468; doi:10.1038/srep30953)
Supplement: Supplementary Information [file srep30953-s1.pdf]

SUPPLEMENTARY INFORMATION

microRNA-132/212 deficiency enhances Aβ production and senile plaque deposition in Alzheimer's disease triple transgenic mice

Julia Hernandez-Rapp, Sara Rainone, Claudia Goupil, Véronique Dorval, Pascal Y. Smith, Martine Saint-Pierre, Maxime Vallée, Emmanuel Planel, Arnaud Droit, Frédéric Calon, Francesca Cicchetti, Sébastien S. Hébert

FIGURE LEGENDS

Supplementary Figure S1. ELISA of endogenous (murine) Aβ40 and Aβ42 in the **(a)** cortex and **(b)** hippocampus of 18 month-old $3xTgAD^{WT}$ and $3xTgAD^{KO}$ mice (n= 12 per group). Data are presented as mean ± SEM. *P≤0.05 (Student's t-test).

Supplementary Figure S2. GeneMANIA analysis of miR-132 target gene networks.

Supplementary Figure S3. miR-132 qRT-PCR in **(a)** Neuro2a and **(b)** HEK293 cells treated with a scrambled control or miR-132 mimics (n= 2 cultures in triplicate per group). RNU19 was used as normalization control. ELISA of human Aβ, presented as Aβ42/Aβ40 ratio, in HEK293-APPSwe cells after **(c)** siRNA-Sirt1 and **(d)** EX527 treatment (n=2 cultures in triplicate per group). Data are presented as mean ± SEM (Student's t-test). *P≤0.05 (Student's t-test).

Supplementary Figure S4. Western blot analysis of Sirt1 in HEK293-APPSwe cells after siRNA downregulation (n=2 cultures in triplicate per group). Total proteins were used as normalization control. Data are presented as mean ± SEM. ****P≤0.0001 (Student's t-test).

Supplementary Figure S5. Correlations of miR-132/212, insoluble Aβ42, amyloid plaque counts and Sirt1 with the age of death of patients in the ROS cohort (n=10-12 cases/group).

Supplementary Figure S6. Whole blot/gels used in the current study. Empty lanes are irrelevant samples.

TABLE LEGENDS

Supplementary Table S1. Overview of raw and mapped RNA-Seq reads**.**

Supplementary Table S2. Misregulated transcripts in 3xTg-AD^{KO} mice and Neuro2a^{132} cells**.**

Supplementary Table S3. miR-132/212 targets in 3xTg-AD^{KO} mice and Neuro2a^{132} cells**.**

Supplementary Table S4. GO terms related to miR-132 networks in 3xTg-AD^{KO} mice and Neuro2a^{132} cells.

Supplementary Table S5. Correlations between miR-132 or miR-212 expression levels, insoluble Aβ42, Aβ plaque load, and Sirt1 protein levels for each group of patients in the ROS cohort (n=10-12 cases/group).

Supplementary Figure S1

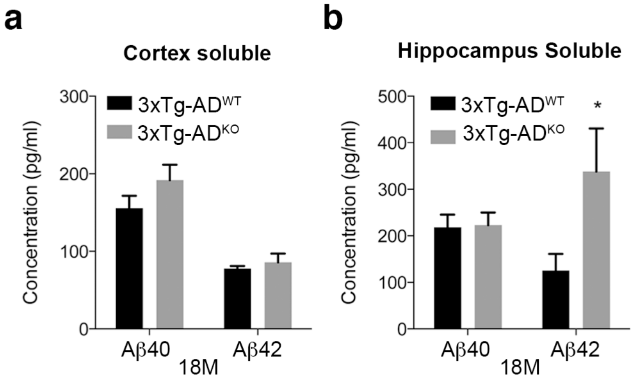

Supplementary Figure S2

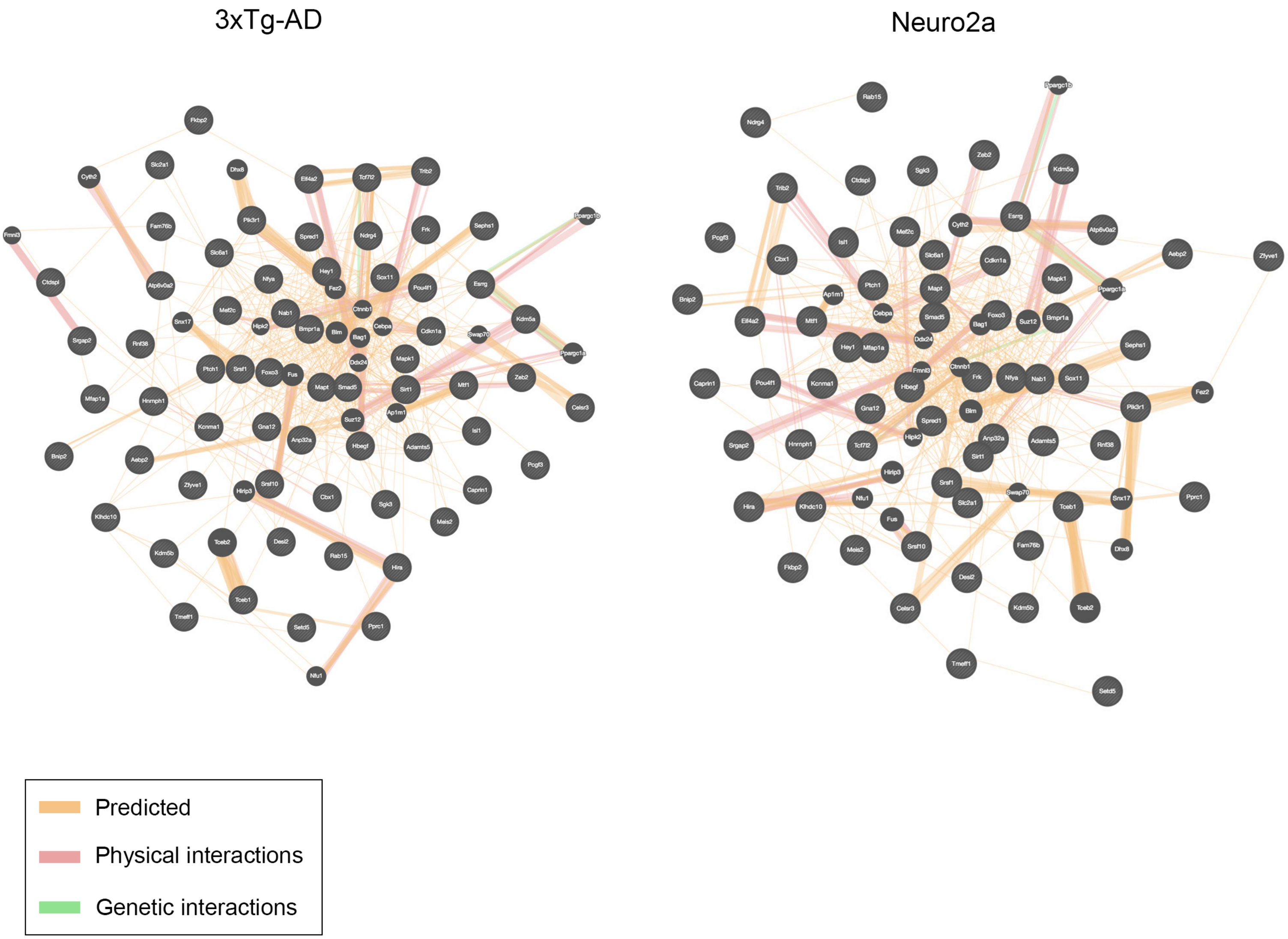

Supplementary Figure S3

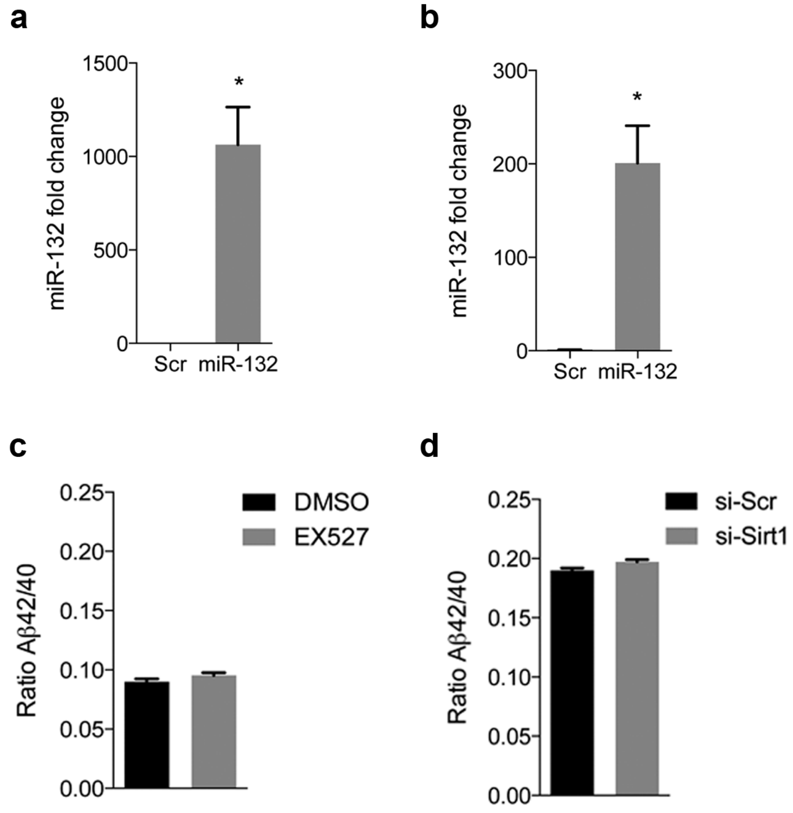

Supplementary Figure S4

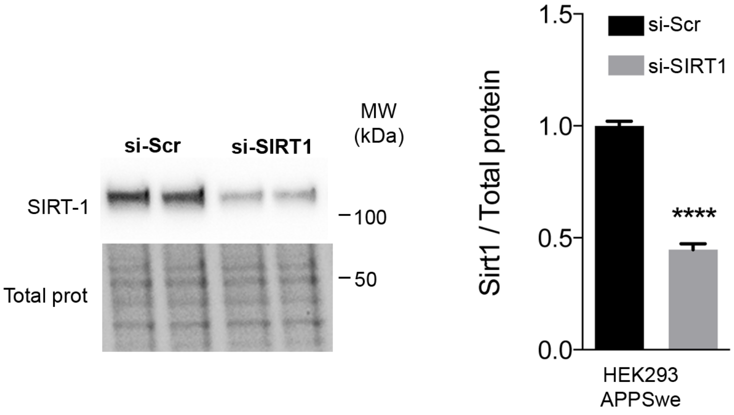

Supplementary Figure S5

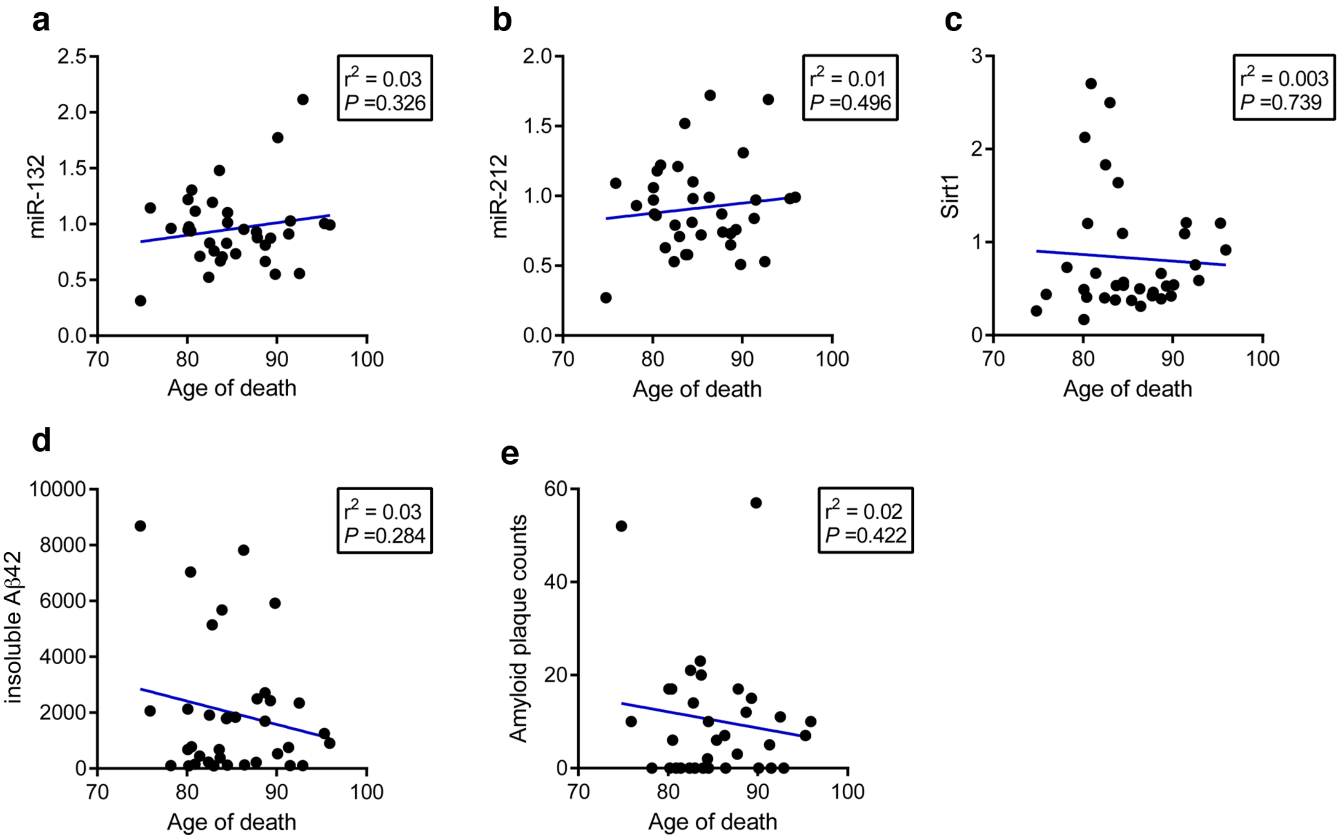

Supplementary Figure S6

Figure 2d

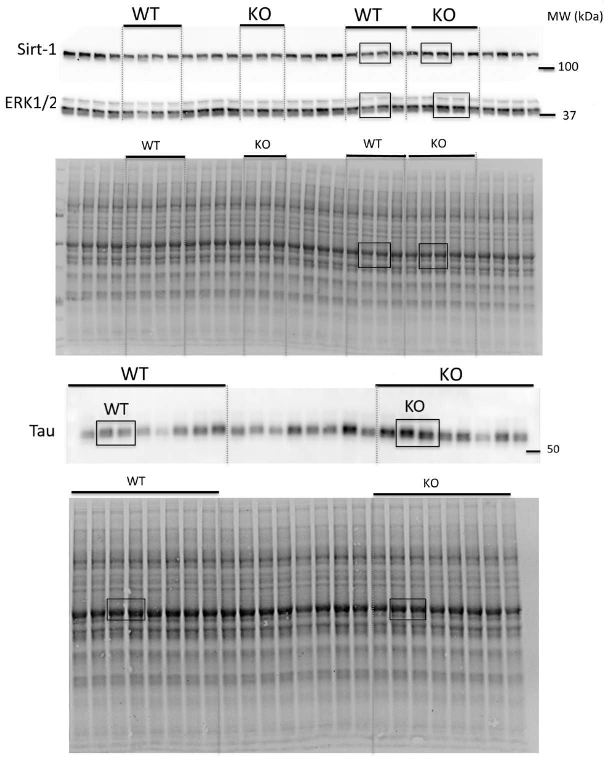

Figure 2e

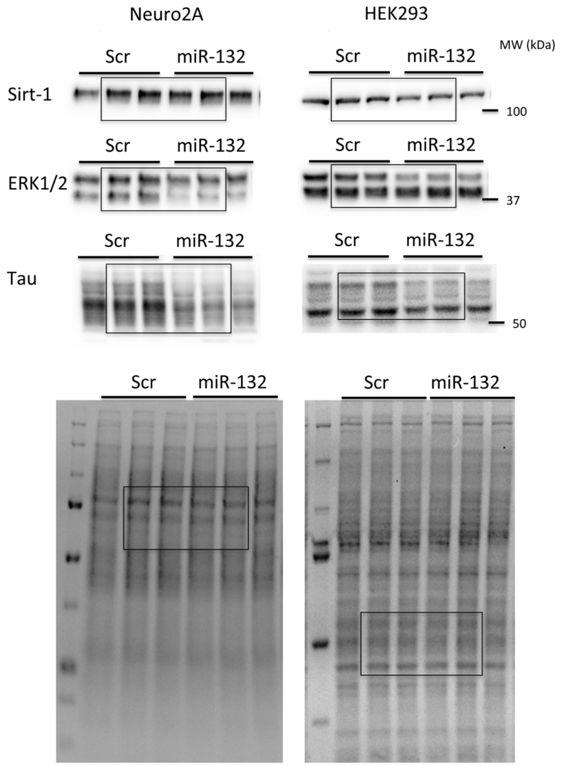

Figure 3c

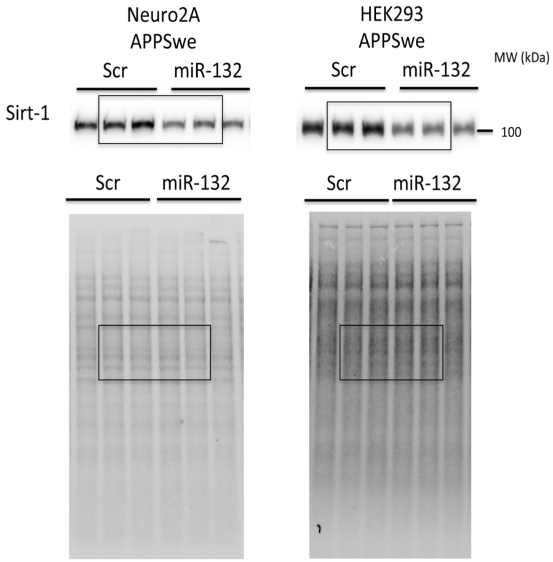

Figure 4f

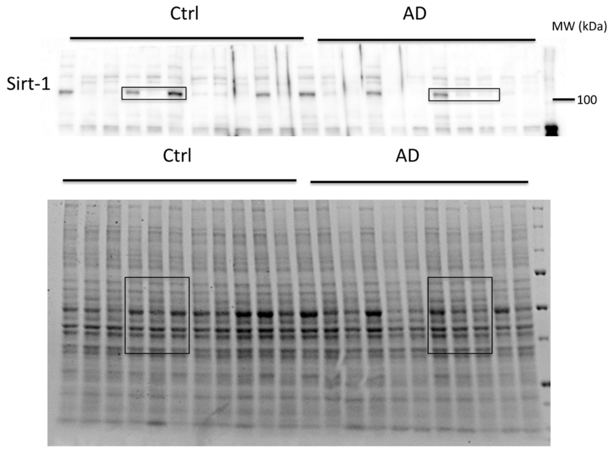

Figure S2

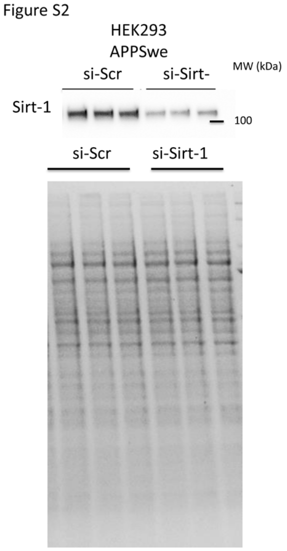

Supplementary Table S1

Sample ID	Raw reads	After trimming	Mapped	Multiple hits reads
12Mo-3xTgKO-1	14,658,099	14,475,868	14,176,291	685,492
12Mo-3xTgKO-2	13,522,495	13,355,267	13,105,801	713,386
12Mo-3xTgKO-3	20,095,936	19,931,929	19,541,036	991,957
12Mo-3xTgKO-4	10,008,104	9,926,244	9,714,450	503,373
12Mo-3XTgWT-1	8,112,412	8,007,292	7,844,073	411,849
12Mo-3XTgWT-2	17,824,275	17,597,261	17,224,382	893,425
12Mo-3XTgWT-3	18,468,579	18,124,799	17,760,844	930,596
12Mo-3XTgWT-4	22,401,758	21,991,588	21,530,896	1,246,739
Neuro2a-miRNA132-1	15,506,750	15,317,490	15,029,443	2,123,251
Neuro2a-miRNA132-2	16,846,358	16,638,101	16,316,687	1,730,920
Neuro2a-miRNA132-3	14,009,580	13,899,520	13,640,336	1,509,564
Neuro2a-miRNA132-4	14,116,956	14,001,776	13,721,093	1,463,188
Neuro2a-SCR-1	16,570,614	16,362,134	16,053,090	2,137,581
neuro2a-SCR-2	10,806,408	10,669,735	10,471,362	1,201,608
Neuro2a-SCR-3	17,261,089	16,948,711	16,525,803	1,916,186
Neuro2a-SCR-4	30,899,105	30,334,254	29,785,095	3,315,080

Supplementary Table S2

RNA-Seq expression analysis of 3xTg-AD mice

Gene Symbol	Transcript	p-value	Ratio (KO vs. WT)	Fold Change
0610005C13Rik	0610005C13Rik-009	0.0341598	5.51546e-06	-181309
0610005C13Rik	0610005C13Rik-005	0.0335434	3.20175e+08	3.20175e+08
0610038B21Rik	0610038B21Rik-001	0.0393228	2.71101	2.71101
1110002E22Rik	1110002E22Rik-001	0.0103241	0.677821	-1.47532
1110004E09Rik	1110004E09Rik-003	0.0161545	0.00639356	-156.407
1110004F10Rik	1110004F10Rik-006	0.0212205	0.46833	-2.13525
1110006O24Rik	1110006O24Rik-001	0.0461868	0.515567	-1.93961
1110059E24Rik	1110059E24Rik-007	0.0404261	2.06527e+08	2.06527e+08
1300017J02Rik	1300017J02Rik-001	0.0336934	22445.1	22445.1
1500012F01Rik	1500012F01Rik-004	0.0190517	0.671701	-1.48876
1500015O10Rik	1500015O10Rik-001	0.0453942	12.4266	12.4266
1500026H17Rik	1500026H17Rik-201	0.0128515	0.621867	-1.60806
1600029O15Rik	1600029O15Rik-001	0.00759147	5.38875	5.38875
1700001L05Rik	1700001L05Rik-004	0.0110301	180.945	180.945
1700001O22Rik	1700001O22Rik-006	0.0486048	0.0697255	-14.342
1700001O22Rik	1700001O22Rik-011	0.0280517	1984.58	1984.58
1700007F19Rik	1700007F19Rik-001	0.0105239	3.12346	3.12346
1700012B09Rik	1700012B09Rik-004	0.0376975	6157.05	6157.05
1700019D03Rik	1700019D03Rik-003	0.012562	0.287834	-3.47422
1700019D03Rik	1700019D03Rik-001	0.0101993	0.241878	-4.13431
1700019D03Rik	1700019D03Rik-002	0.0278685	1.46714	1.46714
1700019N19Rik	1700019N19Rik-001	0.0399664	3.47762	3.47762
1700021L23Rik	1700021L23Rik-001	0.0103687	0.134787	-7.41912
1700021N21Rik	1700021N21Rik-001	0.0257154	0.34938	-2.86221
1700023F06Rik	1700023F06Rik-005	0.0345539	2.18809e+07	2.18809e+07
1700028K03Rik	1700028K03Rik-003	0.0174366	0.0299594	-33.3785
1700028P14Rik	1700028P14Rik-201	0.0447788	3.03534	3.03534
1700030J22Rik	1700030J22Rik-201	0.0473772	1.19801	1.19801
1700030L20Rik	1700030L20Rik-001	0.00511771	0.0585682	-17.0741
1700037C18Rik	1700037C18Rik-005	0.0123617	1.64591	1.64591
1700037H04Rik	1700037H04Rik-003	0.037755	6.69801	6.69801
1700049G17Rik	1700049G17Rik-004	0.0418774	0.193183	-5.17645
1700101E01Rik	1700101E01Rik-002	0.0307251	0.000127876	-7820.07
1700104B16Rik	1700104B16Rik-002	0.0365932	1.64064e-05	-60951.7
1700109K24Rik	1700109K24Rik-201	0.00244522	0.603026	-1.6583
1700120B22Rik	1700120B22Rik-001	0.0366881	0.276952	-3.61073
1810041L15Rik	1810041L15Rik-002	0.00745192	0.190051	-5.26175
1810062O18Rik	1810062O18Rik-005	0.0219612	11.8233	11.8233

2010013B24Rik	2010013B24Rik-001	0.0182019	0.10034	-9.96608
2010204K13Rik	2010204K13Rik-002	0.0288904	0.726506	-1.37645
2210013O21Rik	2210013O21Rik-004	0.021503	0.503432	-1.98636
2210018M11Rik	2210018M11Rik-011	0.0402965	1.75588	1.75588
2210018M11Rik	2210018M11Rik-002	0.0386256	1.32625	1.32625
2210408F21Rik	2210408F21Rik-015	0.0340316	0.317405	-3.15055
2310001H17Rik	2310001H17Rik-006	0.000276608	4.06577e+06	4.06577e+06
2310002F09Rik	2310002F09Rik-001	0.0273644	0.564257	-1.77224
2310067B10Rik	2310067B10Rik-002	0.0167337	1.39372	1.39372
2310075C17Rik	2310075C17Rik-001	0.0482878	5.48954	5.48954
2410022M11Rik	2410022M11Rik-001	0.0327027	0.718414	-1.39195
2410131K14Rik	2410131K14Rik-001	0.0181157	1.2221	1.2221
2610001J05Rik	2610001J05Rik-002	0.0479107	0.785482	-1.2731
2610001J05Rik	2610001J05Rik-001	0.0303785	1.32206	1.32206
2610020H08Rik	2610020H08Rik-002	0.0494125	4.55139	4.55139
2610020H08Rik	2610020H08Rik-201	0.0494125	4.55139	4.55139
2610203C20Rik	2610203C20Rik-013	0.0197014	0.0119376	-83.7692
2610507I01Rik	2610507I01Rik-001	0.0330261	0.771579	-1.29604
2610524H06Rik	2610524H06Rik-001	0.0365532	0.787519	-1.26981
2610528A11Rik	2610528A11Rik-002	0.0371238	2.4517	2.4517
2810001G20Rik	2810001G20Rik-002	0.0168932	0.126085	-7.93118
2810025M15Rik	2810025M15Rik-001	0.0117598	2.47868	2.47868
2810408A11Rik	2810408A11Rik-006	0.0321868	3.68734e+07	3.68734e+07
2810428I15Rik	2810428I15Rik-006	0.0454807	5.28716	5.28716
2900041M22Rik	2900041M22Rik-001	0.00333705	0.383413	-2.60815
3110052M02Rik	3110052M02Rik-201	0.0256309	0.738145	-1.35475
3632451O06Rik	3632451O06Rik-001	0.039289	0.821624	-1.2171
4632428C04Rik	4632428C04Rik-201	0.0221921	1.81567	1.81567
4632428N05Rik	4632428N05Rik-003	0.0446542	1.77538	1.77538
4833423E24Rik	4833423E24Rik-001	0.016013	0.39026	-2.5624
4921514A10Rik	4921514A10Rik-001	0.00104517	2.3211	2.3211
4930402H24Rik	4930402H24Rik-002	0.0275765	8.67921	8.67921
4930427A07Rik	4930427A07Rik-001	0.0373109	6.96624	6.96624
4930448E22Rik	4930448E22Rik-201	0.0280782	13.7815	13.7815
4930467D21Rik	4930467D21Rik-002	0.0339172	0.288953	-3.46077
4930477O15Rik	4930477O15Rik-001	0.0142573	3.04015	3.04015
4930480K23Rik	4930480K23Rik-201	0.0413887	0.709016	-1.41041
4930502E18Rik	4930502E18Rik-001	0.00707674	0.116213	-8.60488
4930523C07Rik	4930523C07Rik-003	0.0484115	0.000104661	-9554.65
4930544I03Rik	4930544I03Rik-201	0.0494802	8.36046	8.36046
4931406P16Rik	4931406P16Rik-008	0.0439143	41.6223	41.6223
4932418E24Rik	4932418E24Rik-002	0.0355704	5.46742	5.46742
4933413J09Rik	4933413J09Rik-004	0.0321115	2.22826	2.22826

4933415A04Rik	4933415A04Rik-201	0.0304863	2.02975	2.02975
4933431E20Rik	4933431E20Rik-013	0.0483268	0.0727086	-13.7535
4933439C10Rik	4933439C10Rik-003	0.0390863	2.76807	2.76807
5031434O11Rik	5031434O11Rik-001	0.00793112	0.869125	-1.15058
5330434G04Rik	5330434G04Rik-002	0.0309033	0.782137	-1.27855
5730409E04Rik	5730409E04Rik-001	0.00830099	0.692243	-1.44458
5830415F09Rik	5830415F09Rik-002	0.0362168	0.000116327	-8596.44
6330418K02Rik	6330418K02Rik-004	0.0415893	227.208	227.208
6330549D23Rik	6330549D23Rik-002	0.0371848	1.49846e-05	-66735.4
6430511E19Rik	6430511E19Rik-001	0.0247296	0.786297	-1.27178
8030423F21Rik	8030423F21Rik-001	0.00633818	0.375599	-2.66241
9030624J02Rik	9030624J02Rik-005	0.0204737	0.137454	-7.27515
9130019P16Rik	9130019P16Rik-001	0.0294761	1.7961	1.7961
9330182L06Rik	9330182L06Rik-003	0.0384434	2.60262	2.60262
9430016H08Rik	9430016H08Rik-001	0.0347718	0.905761	-1.10404
9430091E24Rik	9430091E24Rik-001	0.0348101	1.78241	1.78241
9530026P05Rik	9530026P05Rik-004	0.0229603	0.000480771	-2079.99
9530068E07Rik	9530068E07Rik-003	0.0260034	2.42878e-09	-4.11729e+08
9530068E07Rik	9530068E07Rik-001	0.0487867	1.10002	1.10002
9530068E07Rik	9530068E07Rik-201	0.0491204	1.0999	1.0999
9630041A04Rik	9630041A04Rik-001	0.00513035	3.08559	3.08559
9930014A18Rik	9930014A18Rik-004	0.0379277	35178.9	35178.9
9930111J21Rik1	9930111J21Rik1-002	0.047877	5.90711	5.90711
A130006I12Rik	A130006I12Rik-201	0.0302163	1.83498	1.83498
A230065N10Rik	A230065N10Rik-201	0.0282423	1.87737	1.87737
A330094K24Rik	A330094K24Rik-201	0.0182556	3.33133	3.33133
A430005L14Rik	A430005L14Rik-003	0.00189089	0.112617	-8.87963
A530016L24Rik	A530016L24Rik-201	0.0253442	0.514062	-1.94529
A530058N18Rik	A530058N18Rik-007	0.0270457	5.96244e+09	5.96244e+09
A730015C16Rik	A730015C16Rik-201	0.0373852	4.87038	4.87038
A730017C20Rik	A730017C20Rik-004	0.0186016	6.87473	6.87473
A730020E08Rik	A730020E08Rik-002	0.0302653	0.759069	-1.3174
A730061H03Rik	A730061H03Rik-201	0.0345336	0.307483	-3.25222
A730089K16Rik	A730089K16Rik-001	0.0208586	0.390473	-2.56099
A830008E24Rik	A830008E24Rik-002	0.0493743	11.0407	11.0407
A830010M20Rik	A830010M20Rik-003	0.0350874	0.0385225	-25.9589
A830018L16Rik	A830018L16Rik-003	0.0498595	0.904561	-1.10551
A830018L16Rik	A830018L16Rik-007	0.0140302	0.491722	-2.03367
A830018L16Rik	A830018L16Rik-004	0.0480506	7.37221e-11	-1.35644e+10
A830019P07Rik	A830019P07Rik-001	0.041588	0.668708	-1.49542
A830036E02Rik	A830036E02Rik-001	0.00176556	2.075	2.075
A830036E02Rik	A830036E02Rik-002	0.0101659	1.97506	1.97506
A830082K12Rik	A830082K12Rik-007	0.0495417	0.7589	-1.3177

A930004D18Rik	A930004D18Rik-002	0.000828422	0.13324	-7.50527
A930033H14Rik	A930033H14Rik-001	0.0438727	0.82642	-1.21004
AA465934	AA465934-002	7.97177e-11	0.0152526	-65.5627
AA465934	AA465934-005	4.16596e-05	0.389118	-2.56991
AA465934	AA465934-003	0.00247462	12.4649	12.4649
AA467197	AA467197-001	0.0341402	6.27326e-07	-1.59407e+06
Aamdc	Aamdc-004	0.0481876	0.129013	-7.75117
Aamdc	Aamdc-013	0.0413897	0.612629	-1.63231
Aars	Aars-003	0.024039	0.26791	-3.7326
Aarsd1	Aarsd1-009	0.00548557	0.241682	-4.13767
Aass	Aass-001	0.0469601	1.31026	1.31026
Aatk	Aatk-009	0.00195042	1.46064e-07	-6.84633e+06
Abca3	Abca3-201	0.0167236	0.821812	-1.21682
Abca5	Abca5-004	0.0381058	1.00529e+09	1.00529e+09
Abcb4	Abcb4-002	0.0309406	0.198209	-5.04519
Abcb9	Abcb9-001	0.0220301	1.21652	1.21652
Abcc3	Abcc3-001	0.0316451	0.593263	-1.68559
Abcc5	Abcc5-002	0.0226062	0.34595	-2.89059
Abcf1	Abcf1-001	0.0369512	1.0906	1.0906
Abcf3	Abcf3-001	0.0302241	1.09208	1.09208
Abhd11os	Abhd11os-001	0.0498734	5.45486	5.45486
Abhd12	Abhd12-002	0.00833497	0.752956	-1.3281
Abhd14a	Abhd14a-006	0.0386227	0.293407	-3.40824
Abhd14b	Abhd14b-006	0.0220642	7.30832e-06	-136830
Abhd16a	Abhd16a-006	0.00350139	2.20636	2.20636
Abi1	Abi1-007	0.00557798	0.629019	-1.58978
Abi3bp	Abi3bp-201	0.0341766	6.32526	6.32526
Ablim1	Ablim1-006	0.0228343	0.239166	-4.1812
Abr	Abr-010	0.000557524	0.611484	-1.63537
Abr	Abr-201	0.00212027	0.8773	-1.13986
Abr	Abr-001	0.00339719	0.758038	-1.3192
AC154247.1	AC154247.1-201	0.027146	481.986	481.986
AC156028.1	AC156028.1-201	0.011353	3.48529	3.48529
Acaa1b	Acaa1b-001	0.0398333	0.286618	-3.48896
Acaa2	Acaa2-201	0.0296863	1.52209	1.52209
Acacb	Acacb-201	0.0219058	1.78553	1.78553
Acad11	Acad11-004	0.0355756	12.3366	12.3366
Acadsb	Acadsb-001	0.0378781	1.11834	1.11834
Acat3	Acat3-001	0.0157875	0.693006	-1.44299
Acbd4	Acbd4-201	0.0234978	0.435674	-2.29529
Accs	Accs-003	0.0358354	0.109838	-9.10435
Acer3	Acer3-001	0.0403951	0.87351	-1.14481
Acer3	Acer3-002	0.0388639	0.538272	-1.8578

Acin1	Acin1-008	0.0472637	1.78219	1.78219
Acly	Acly-001	0.0173986	1.25744	1.25744
Acot10	Acot10-201	0.0192098	9.70644	9.70644
Acot6	Acot6-001	0.0036528	0.814773	-1.22734
Acot8	Acot8-003	0.00110093	1.79869	1.79869
Acot9	Acot9-004	0.0254925	9.08173	9.08173
Acox1	Acox1-006	0.0238178	0.111012	-9.00804
Acp6	Acp6-004	0.0246501	128.9	128.9
Acrbp	Acrbp-005	0.0425719	3.30846	3.30846
Acsl6	Acsl6-011	0.041014	2.48554	2.48554
Acss3	Acss3-201	0.0208711	2.26059	2.26059
Acvr1	Acvr1-201	0.00823161	0.63281	-1.58025
Acvr1b	Acvr1b-001	0.00579639	1.06595	1.06595
Adam11	Adam11-002	0.00702503	0.622109	-1.60744
Adam11	Adam11-001	0.0325438	0.88068	-1.13549
Adam11	Adam11-003	0.0255085	3.92398	3.92398
Adam15	Adam15-002	0.0448074	0.641418	-1.55905
Adam1a	Adam1a-001	0.0136425	1.29099	1.29099
Adam22	Adam22-001	0.0371486	0.088209	-11.3367
Adam22	Adam22-003	0.042545	0.787815	-1.26933
Adam32	Adam32-006	0.00993811	0.139239	-7.18188
Adamts10	Adamts10-002	0.00443992	0.351004	-2.84897
Adamts5	Adamts5-201	0.0058868	1.8308	1.8308
Adamts7	Adamts7-005	0.0177226	9.99489	9.99489
Adamtsl5	Adamtsl5-007	0.0318655	8.10437	8.10437
Adamtsl5	Adamtsl5-005	0.0170894	9.21674	9.21674
Adarb1	Adarb1-011	0.0297224	2.63215e-05	-37991.8
Adarb2	Adarb2-001	0.0217472	0.835236	-1.19727
Adcy10	Adcy10-004	0.0111635	9.60713	9.60713
Adcy9	Adcy9-001	0.0340056	0.863113	-1.1586
Add2	Add2-004	0.00937376	1.32093	1.32093
Adgra3	Adgra3-003	0.0264755	1095.34	1095.34
Adgrb1	Adgrb1-001	0.0301387	1.38529	1.38529
Adgrg5	Adgrg5-001	0.0321465	7.90575	7.90575
Adgrg5	Adgrg5-201	0.0321465	7.90575	7.90575
Adgrl1	Adgrl1-009	0.0152024	0.436019	-2.29348
Adgrl2	Adgrl2-017	0.0309662	0.0030895	-323.677
Adgrl3	Adgrl3-014	0.033775	2.09023	2.09023
Adi1	Adi1-002	0.0120029	0.515517	-1.9398
Adi1	Adi1-001	0.00275524	1.1838	1.1838
Adipor1	Adipor1-002	0.00590016	1.52928	1.52928
Adipor2	Adipor2-001	0.00193825	1.37325	1.37325
Adrb3	Adrb3-001	0.0115791	0.19408	-5.1525

Aebp2	Aebp2-002	0.0492824	1.13445	1.13445
Aff3	Aff3-002	0.0111661	0.845905	-1.18217
Aff4	Aff4-003	0.0235733	0.200893	-4.97776
Aff4	Aff4-006	0.0436744	0.0459494	-21.7631
Afg3l1	Afg3l1-005	0.0397667	420.224	420.224
Agbl2	Agbl2-004	0.0314201	0.000622212	-1607.17
Agbl2	Agbl2-201	0.0484116	3.25968	3.25968
Agbl2	Agbl2-202	0.0368045	6.22792	6.22792
Agbl3	Agbl3-006	0.0023497	0.330816	-3.02283
Agbl3	Agbl3-002	0.0190911	0.659227	-1.51693
Agbl3	Agbl3-009	0.0025208	4.23824	4.23824
Aggf1	Aggf1-002	0.0100779	0.197255	-5.06957
Ago4	Ago4-001	0.0371251	1.17344	1.17344
Agpat1	Agpat1-006	0.00291535	11.0345	11.0345
Agpat1	Agpat1-008	0.0204788	1.52265	1.52265
Agpat4	Agpat4-006	0.000689748	0.188487	-5.3054
Agpat9	Agpat9-002	0.0212969	1.55732	1.55732
Agtrap	Agtrap-001	0.0106338	1.2461	1.2461
Ahctf1	Ahctf1-007	0.0254079	1.98167	1.98167
Ahcyl2	Ahcyl2-018	0.0154198	0.455143	-2.19711
Ahrr	Ahrr-002	0.0294306	19712.7	19712.7
AI118078	AI118078-002	0.0435975	2.42332	2.42332
AI413582	AI413582-001	0.00834294	1.40075	1.40075
Aif1	Aif1-004	0.00763838	184.513	184.513
Aif1l	Aif1l-007	0.0345697	1.90293e-11	-5.25505e+10
Aifm1	Aifm1-004	0.00195603	4.63713	4.63713
Aig1	Aig1-003	0.0127532	8.22011	8.22011
Aimp2	Aimp2-001	0.0226873	0.713252	-1.40203
Aip	Aip-001	0.00944953	0.607808	-1.64526
Aip	Aip-003	0.0285242	0.465476	-2.14834
Aip	Aip-002	6.6708e-05	1.19179	1.19179
Ak2	Ak2-003	0.0312899	469528	469528
Akap1	Akap1-002	0.029004	1.25634	1.25634
Akap11	Akap11-201	0.0260517	0.904583	-1.10548
Akap6	Akap6-201	0.0259984	0.917934	-1.0894
Akr1b8	Akr1b8-002	0.00985598	171579	171579
Akt1s1	Akt1s1-003	0.0247747	0.487798	-2.05003
Akt2	Akt2-002	0.0493463	0.571666	-1.74927
Akt2	Akt2-001	0.0350608	1.29071	1.29071
Akt3	Akt3-201	0.0156223	0.793015	-1.26101
Alad	Alad-002	0.00419574	0.344203	-2.90527
Alad	Alad-001	0.00608514	1.13128	1.13128
Aldh1l1	Aldh1l1-004	0.0124605	0.127388	-7.85005

Aldh1l1	Aldh1l1-001	0.0320766	1.82977	1.82977
Aldh1l2	Aldh1l2-004	0.0257467	4.19389	4.19389
Aldh4a1	Aldh4a1-001	0.038498	1.17755	1.17755
Aldh7a1	Aldh7a1-008	0.0220226	0.657399	-1.52115
Aldh7a1	Aldh7a1-002	0.0212849	1.23509	1.23509
Alg1	Alg1-001	0.00124685	3.8239	3.8239
Alg12	Alg12-001	0.0435341	0.645884	-1.54827
Alg2	Alg2-001	0.0131887	0.884476	-1.13061
Alg5	Alg5-001	0.0234072	0.925205	-1.08084
Alms1	Alms1-001	0.0159771	1.20352	1.20352
Aloxe3	Aloxe3-002	0.0192306	4.79397	4.79397
Alpk1	Alpk1-001	0.00225817	2.73691	2.73691
Alyref	Alyref-003	0.0052404	1.8124	1.8124
Ambra1	Ambra1-006	0.0467409	0.0133386	-74.9704
Amdhd2	Amdhd2-003	0.0299236	0.708938	-1.41056
Ammecr1	Ammecr1-001	0.00936088	1.29664	1.29664
Amn1	Amn1-002	0.000606554	1.81859	1.81859
Amotl1	Amotl1-001	0.00778478	1.30988	1.30988
Amotl1	Amotl1-201	0.00778535	1.30987	1.30987
Amotl1	Amotl1-004	0.0346301	6.55598	6.55598
Amph	Amph-002	0.0147254	0.852609	-1.17287
Anapc1	Anapc1-008	0.049015	6.42683e-05	-15559.8
Anapc10	Anapc10-201	0.0292787	0.846887	-1.1808
Anapc13	Anapc13-005	0.0486909	1.78029	1.78029
Anapc15	Anapc15-011	0.0139876	0.46191	-2.16492
Anapc2	Anapc2-001	0.040123	1.06524	1.06524
Angel1	Angel1-005	0.0341145	1.28667e+07	1.28667e+07
Angptl2	Angptl2-002	0.0133912	7.96503	7.96503
Ank	Ank-001	0.027021	0.931816	-1.07317
Ank2	Ank2-010	0.00473742	6.19571e+08	6.19571e+08
Ank3	Ank3-030	0.00121121	5.25334e-05	-19035.5
Ank3	Ank3-018	0.0017414	0.00135739	-736.71
Ank3	Ank3-024	0.047372	11.7946	11.7946
Ankef1	Ankef1-201	0.0150841	5.6729	5.6729
Ankhd1	Ankhd1-001	0.0131419	1.38639	1.38639
Ankle2	Ankle2-004	0.0407439	3843.63	3843.63
Ankrd13c	Ankrd13c-008	0.00040767	8.62463	8.62463
Ankrd29	Ankrd29-001	0.0350632	0.787109	-1.27047
Ankrd50	Ankrd50-201	0.0439326	0.602605	-1.65946
Ankrd9	Ankrd9-003	0.0338412	1.66686	1.66686
Anks1b	Anks1b-012	0.0225763	0.39268	-2.5466
Anks1b	Anks1b-026	0.0234546	0.144655	-6.91298
Ano1	Ano1-007	0.0184628	0.0458311	-21.8192

Ano4	Ano4-003	0.035987	0.141603	-7.06202
Anp32a	Anp32a-004	0.036369	1.75217	1.75217
Anpep	Anpep-005	0.021163	3.03204e-10	-3.29811e+09
Antxr1	Antxr1-001	0.00163097	2.33076	2.33076
Anxa3	Anxa3-009	0.0196675	32.9593	32.9593
Anxa5	Anxa5-001	0.00252557	1.13313	1.13313
Anxa9	Anxa9-201	0.00462856	23.4817	23.4817
Ap1s2	Ap1s2-003	0.0282345	2.33173	2.33173
Ap2b1	Ap2b1-002	0.00897947	0.162054	-6.17077
Ap3b2	Ap3b2-001	0.0109508	0.884208	-1.13096
Ap5z1	Ap5z1-007	0.0395204	1.5098	1.5098
Apbb1	Apbb1-003	0.00853227	0.828177	-1.20747
Apbb1	Apbb1-201	0.024579	1.17162	1.17162
Apc	Apc-201	0.0171358	0.916256	-1.0914
Apc	Apc-007	0.0144655	0.104463	-9.57278
Apeh	Apeh-005	0.0441034	6.11113	6.11113
Apobec3	Apobec3-202	0.00977299	35.8578	35.8578
Apoe	Apoe-006	0.0175333	1.40004	1.40004
Ar	Ar-001	0.00944434	0.860942	-1.16152
Araf	Araf-006	0.0219353	0.531652	-1.88093
Araf	Araf-001	0.00136741	2.56368	2.56368
Arap1	Arap1-017	0.00568345	21.6009	21.6009
Areg	Areg-001	0.0154633	10.4633	10.4633
Arfgap1	Arfgap1-010	0.0399152	0.765525	-1.30629
Arfgap1	Arfgap1-012	0.0348895	0.0136841	-73.0773
Arfgap1	Arfgap1-004	0.0480991	1.97265	1.97265
Arfgap3	Arfgap3-201	0.0229759	1.11901	1.11901
Arfrp1	Arfrp1-008	0.0309704	0.25351	-3.94461
Arfrp1	Arfrp1-001	0.00696063	1.93405	1.93405
Arhgap1	Arhgap1-003	0.0405171	0.746935	-1.3388
Arhgap1	Arhgap1-201	0.00333914	2.91592	2.91592
Arhgap12	Arhgap12-006	0.0167956	0.760551	-1.31484
Arhgap12	Arhgap12-002	0.0240124	1.34822	1.34822
Arhgap25	Arhgap25-002	0.0311646	0.2991	-3.34336
Arhgap27os3	Arhgap27os3-001	0.003864	0.277917	-3.5982
Arhgef16	Arhgef16-005	0.0333069	6.13722	6.13722
Arhgef2	Arhgef2-028	0.0141913	2.00178	2.00178
Arid1b	Arid1b-001	0.0138756	0.869387	-1.15024
Arid3b	Arid3b-201	0.0269778	1.30437	1.30437
Arl4d	Arl4d-001	0.00386582	1.38709	1.38709
Arl5a	Arl5a-001	0.0430249	0.878993	-1.13767
Arl6ip1	Arl6ip1-005	0.0350491	0.0323913	-30.8725
Arl6ip6	Arl6ip6-002	0.00522641	0.612563	-1.63248

Arl8a	Arl8a-003	0.0332162	1.49672	1.49672
Arl8b	Arl8b-001	0.0278072	0.932889	-1.07194
Arl9	Arl9-002	0.00557145	7.97867e-07	-1.25334e+06
Armcx1	Armcx1-002	0.0307721	0.52673	-1.89851
Arntl2	Arntl2-001	0.0141632	0.669028	-1.49471
Arpp19	Arpp19-004	0.0161944	1.58516	1.58516
Arpp21	Arpp21-006	0.0272013	0.425561	-2.34984
Arpp21	Arpp21-005	0.0270268	1.42882	1.42882
Arrb2	Arrb2-003	0.0215229	2.49876	2.49876
Arrb2	Arrb2-006	0.0158903	1.92298	1.92298
Arsa	Arsa-001	0.0419901	1.21248	1.21248
Arsg	Arsg-001	0.0347706	1.48793	1.48793
Arsg	Arsg-002	0.0104945	10.0138	10.0138
Art3	Art3-006	0.0267245	4.03335e-07	-2.47933e+06
Arvcf	Arvcf-009	0.0178284	2.32509	2.32509
Asb1	Asb1-002	0.0446298	0.37013	-2.70176
Asb1	Asb1-004	0.0298361	6.5275	6.5275
Asb3	Asb3-003	0.0271281	2.62189	2.62189
Ash2l	Ash2l-009	0.0408966	0.22268	-4.49076
Asic1	Asic1-201	0.0417275	1.27086	1.27086
Asl	Asl-005	0.00154172	8.38395	8.38395
Asl	Asl-009	0.00837177	1.38317	1.38317
Asphd2	Asphd2-002	0.00186718	0.507668	-1.96979
Ass1	Ass1-004	0.0192308	1598.88	1598.88
Aste1	Aste1-009	0.026037	0.2552	-3.9185
Atad3a	Atad3a-004	0.010815	1.81152	1.81152
Atcayos	Atcayos-002	0.005154	0.561041	-1.7824
Ate1	Ate1-202	0.0168816	0.704531	-1.41938
Atf1	Atf1-002	0.0287362	0.00769692	-129.922
Atg16l1	Atg16l1-011	0.00946414	1.46798	1.46798
Atg4b	Atg4b-003	0.0175557	86.6325	86.6325
Atp10d	Atp10d-001	0.00507833	2.40645	2.40645
Atp13a2	Atp13a2-006	0.00904076	0.0133732	-74.7765
Atp13a4	Atp13a4-002	0.00519855	0.191974	-5.20905
Atp1b1	Atp1b1-001	0.0302509	0.927266	-1.07844
Atp1b3	Atp1b3-001	0.0445896	1.10894	1.10894
Atp2b3	Atp2b3-003	0.0251561	1.63199	1.63199
Atp5j	Atp5j-011	0.0295447	0.310867	-3.21681
Atp5sl	Atp5sl-001	0.0345256	1.16036	1.16036
Atp6ap1l	Atp6ap1l-001	0.0186989	16.819	16.819
Atp6v0a1	Atp6v0a1-001	0.0364628	0.899149	-1.11216
Atp6v0a2	Atp6v0a2-001	0.027553	1.04955	1.04955
Atp6v0a4	Atp6v0a4-007	0.0335534	0.0102049	-97.9917

Atp6v0e	Atp6v0e-201	0.0414735	1.27146	1.27146
Atp6v1a	Atp6v1a-001	0.0421562	0.872967	-1.14552
Atp6v1c2	Atp6v1c2-006	9.9614e-07	32037.8	32037.8
Atp6v1g2	Atp6v1g2-004	0.0115584	5.88695	5.88695
Atp6v1h	Atp6v1h-006	0.0241225	0.892265	-1.12074
Atp7a	Atp7a-002	0.0477381	2.37379	2.37379
Atp8a1	Atp8a1-004	0.0199078	0.13527	-7.39264
Atpaf1	Atpaf1-005	0.0123243	4.26771	4.26771
Atpaf2	Atpaf2-006	0.00952668	6.1188	6.1188
Atraid	Atraid-002	0.0325326	0.323279	-3.09331
Atxn3	Atxn3-005	0.0318039	2490.3	2490.3
AU021092	AU021092-001	0.0139642	1.43217	1.43217
AU022252	AU022252-001	0.0430565	0.704143	-1.42017
Auts2	Auts2-003	0.0199646	0.223106	-4.48217
Awat2	Awat2-002	0.0335219	9962.11	9962.11
Axin1	Axin1-201	0.00171809	1.76626	1.76626
Azi2	Azi2-005	0.0439423	4.33636	4.33636
B020004C17Rik	B020004C17Rik-201	0.0440607	0.448536	-2.22948
B230118H07Rik	B230118H07Rik-009	0.0469179	0.150776	-6.63234
B3galt1	B3galt1-002	0.0369165	0.768195	-1.30175
B3galt2	B3galt2-001	0.0082246	0.837364	-1.19422
B3gat1	B3gat1-003	0.0275893	0.27576	-3.62635
B3gat2	B3gat2-003	0.0373991	0.000830983	-1203.39
B3gnt5	B3gnt5-001	0.0427959	0.171826	-5.81985
B3gnt8	B3gnt8-004	0.0233941	0.323817	-3.08817
Bag2	Bag2-004	0.0152441	0.000362121	-2761.51
Bag6	Bag6-004	0.0147244	1.52695	1.52695
Baiap2	Baiap2-001	0.0402378	1.32246	1.32246
Baiap3	Baiap3-007	0.00129155	7.66448	7.66448
Banp	Banp-002	0.0203402	1.62744	1.62744
Batf3	Batf3-001	0.0114313	2.31149	2.31149
BB218582	BB218582-002	0.0264687	0.230731	-4.33405
BB218582	BB218582-003	0.00976504	0.145484	-6.8736
Bbs9	Bbs9-006	0.0188851	7.02957	7.02957
Bbx	Bbx-003	0.0249326	0.147125	-6.79692
BC017158	BC017158-003	0.0498526	6.67197	6.67197
BC026585	BC026585-001	0.031127	1.26788	1.26788
BC029214	BC029214-002	0.0278247	1.65859	1.65859
BC029214	BC029214-012	0.0132889	5.87715	5.87715
BC035044	BC035044-001	0.000972094	3.14682	3.14682
BC037032	BC037032-002	0.0451274	151577	151577
BC046251	BC046251-001	0.0236543	2.59537	2.59537
BC055308	BC055308-001	0.0256288	0.517935	-1.93075

BC065403	BC065403-002	0.0478501	64.0095	64.0095
Bcam	Bcam-001	0.0417478	1.55806	1.55806
Bcar1	Bcar1-201	0.022864	1.12132	1.12132
Bcar3	Bcar3-001	0.0211031	1.39923	1.39923
Bccip	Bccip-001	0.0197298	0.898271	-1.11325
Bccip	Bccip-005	0.0149632	2.15993	2.15993
Bcl11a	Bcl11a-006	0.0151386	0.875651	-1.14201
Bcl2	Bcl2-001	0.00317438	1.31699	1.31699
Bcl2l11	Bcl2l11-005	0.0290861	0.11405	-8.76806
Bcl9l	Bcl9l-201	0.0480612	1.12181	1.12181
Bdnf	Bdnf-007	0.000795886	0.588271	-1.6999
Bdnf	Bdnf-006	0.0451855	1.31801	1.31801
Bet1	Bet1-004	0.0343519	0.0169728	-58.9178
Bhlhe40	Bhlhe40-004	0.027337	0.713291	-1.40195
Bhmt	Bhmt-001	0.0353233	3.87299	3.87299
Blvrb	Blvrb-002	0.0248401	3.70838	3.70838
Blzf1	Blzf1-002	0.0392335	0.678318	-1.47423
Bmp4	Bmp4-001	0.0143153	4.68443	4.68443
Bmp8b	Bmp8b-001	0.0243285	0.258535	-3.86794
Bmpr1a	Bmpr1a-001	0.0442701	1.0726	1.0726
Bms1	Bms1-002	0.0446345	0.540274	-1.85091
Bnc2	Bnc2-012	0.00983234	0.0729942	-13.6997
Bnip1	Bnip1-002	0.0382425	0.407341	-2.45495
Bnip2	Bnip2-201	0.0356005	4.20124	4.20124
Bok	Bok-005	0.0252033	2.31992	2.31992
Borcs8	Borcs8-003	0.0319373	0.355302	-2.8145
Bpnt1	Bpnt1-001	0.0481428	1.06657	1.06657
Brap	Brap-003	0.00191227	2.05586	2.05586
Brcc3	Brcc3-006	0.0325703	5.17447	5.17447
Brd3	Brd3-001	0.0376103	0.592016	-1.68914
Brd8	Brd8-007	0.000448145	0.12229	-8.17726
Brd8	Brd8-002	0.0444486	1.24358	1.24358
Brinp1	Brinp1-001	0.0448537	0.904096	-1.10608
Brinp2	Brinp2-002	0.0281798	2.35234	2.35234
Brox	Brox-005	0.000177068	0.10112	-9.88926
Brox	Brox-001	0.00218093	0.613525	-1.62992
Brox	Brox-002	0.0277557	1.15803	1.15803
Brpf3	Brpf3-005	0.0426692	0.0660672	-15.1361
Brpf3	Brpf3-002	0.0183653	1.97994	1.97994
Brwd1	Brwd1-005	0.0482622	3.10704	3.10704
Btbd10	Btbd10-001	0.00835708	1.29565	1.29565
Btbd6	Btbd6-202	9.65798e-05	0.761145	-1.31381
Btbd6	Btbd6-201	0.0209551	1.58264	1.58264

Btf3l4	Btf3l4-003	0.0408991	0.541999	-1.84502
Btnl9	Btnl9-001	0.0416848	12.4601	12.4601
Bud13	Bud13-002	0.0362608	0.118378	-8.44751
Bzrap1	Bzrap1-001	0.00315183	1.2728	1.2728
Bzrap1	Bzrap1-004	0.020373	1.24217	1.24217
C030005K06Rik	C030005K06Rik-003	0.00997306	0.149185	-6.70307
C030014I23Rik	C030014I23Rik-201	0.0204717	0.84928	-1.17747
C030034I22Rik	C030034I22Rik-001	0.0325	0.65144	-1.53506
C030037D09Rik	C030037D09Rik-001	1.42509e-09	0.238256	-4.19716
C030037D09Rik	C030037D09Rik-002	5.17248e-07	6.25682	6.25682
C130071C03Rik	C130071C03Rik-003	0.0325999	0.386078	-2.59015
C1d	C1d-003	0.0487736	1.31695	1.31695
C1qtnf5	C1qtnf5-002	0.0450701	0.114427	-8.73921
C1ra	C1ra-001	0.0474576	1.63581	1.63581
C230004F18Rik	C230004F18Rik-002	0.0456409	1.48121	1.48121
C230024C17Rik	C230024C17Rik-001	0.0155578	0.35648	-2.80521
C230031I18Rik	C230031I18Rik-001	0.0463056	2.28735	2.28735
C2cd5	C2cd5-008	0.0326395	0.25592	-3.90747
C2cd5	C2cd5-012	0.00749521	4.90363	4.90363
C330007P06Rik	C330007P06Rik-001	0.0209041	0.906545	-1.10309
C330007P06Rik	C330007P06Rik-201	0.0210009	0.906606	-1.10302
C4a	C4a-010	0.0369766	5.84867	5.84867
C4a	C4a-008	0.0400947	4255.12	4255.12
C6	C6-001	0.0139134	7.4128	7.4128
C630004L07Rik	C630004L07Rik-201	0.0264872	5.55441	5.55441
C630004L07Rik	C630004L07Rik-001	0.0264872	5.55441	5.55441
Cables1	Cables1-202	0.00776345	2.3613	2.3613
Cables2	Cables2-001	0.0476424	0.844683	-1.18388
Cacfd1	Cacfd1-002	0.00317988	1.17287	1.17287
Cacna1b	Cacna1b-003	0.0238444	0.640015	-1.56246
Cacna1b	Cacna1b-005	0.0299767	0.244491	-4.09013
Cacna1c	Cacna1c-015	0.0448464	0.134137	-7.45508
Cacna1e	Cacna1e-002	0.0295403	0.686691	-1.45626
Cacna1f	Cacna1f-004	0.00241977	6.67488	6.67488
Cacna1g	Cacna1g-004	0.0490033	1.39749	1.39749
Cacna1g	Cacna1g-010	0.00983254	1.49036	1.49036
Cacna1h	Cacna1h-003	0.0253349	1.72554	1.72554
Cacna2d1	Cacna2d1-006	0.0123445	0.925676	-1.08029
Cacna2d2	Cacna2d2-003	0.0412217	1.51903	1.51903
Cacna2d4	Cacna2d4-001	0.0382586	1.4952	1.4952
Cacnb2	Cacnb2-010	0.00506164	0.656803	-1.52253
Calca	Calca-003	0.00984005	0.134646	-7.42685
Calcoco1	Calcoco1-001	0.0176374	1.09438	1.09438

Caln1	Caln1-005	0.0241711	0.628222	-1.59179
Calu	Calu-001	0.022926	1.13051	1.13051
Camk2d	Camk2d-006	0.00827557	6.22076	6.22076
Camkk2	Camkk2-005	0.0184533	0.620928	-1.61049
Camta1	Camta1-012	0.0315647	0.866661	-1.15385
Camta1	Camta1-003	0.0118298	0.523319	-1.91088
Camta2	Camta2-008	0.0173864	0.811152	-1.23281
Cant1	Cant1-001	0.0438778	0.237886	-4.2037
Capn1	Capn1-202	0.0235406	0.831498	-1.20265
Capn2	Capn2-004	0.0100529	3.84361	3.84361
Capn3	Capn3-005	0.0314184	0.373324	-2.67863
Capn3	Capn3-002	0.0204427	3.2568	3.2568
Capn8	Capn8-003	0.0381844	35.2878	35.2878
Capn8	Capn8-201	0.0381844	35.2878	35.2878
Capns1	Capns1-006	0.0476509	0.260587	-3.83749
Caprin1	Caprin1-004	0.0407673	8.68101	8.68101
Car15	Car15-001	0.0284286	1.21211	1.21211
Car9	Car9-007	0.0405983	0.000663086	-1508.1
Card10	Card10-001	0.00604003	0.870322	-1.149
Card9	Card9-002	0.00999542	0.33354	-2.99814
Carhsp1	Carhsp1-201	0.0305276	1.20902	1.20902
Carm1	Carm1-001	0.0163069	1.19359	1.19359
Caskin2	Caskin2-001	0.0320561	1.09178	1.09178
Casp6	Casp6-004	0.0226712	1.77951	1.77951
Catsperg1	Catsperg1-005	0.0460555	0.350917	-2.84967
Catsperg1	Catsperg1-006	0.0187513	5.42515	5.42515
Cbfb	Cbfb-002	0.0106019	5.75911	5.75911
Cbll1	Cbll1-202	0.0350436	0.0826851	-12.0941
Cbx1	Cbx1-002	0.0326135	548.27	548.27
Cbx3	Cbx3-003	0.0154721	0.69274	-1.44354
Cbx7	Cbx7-008	0.0346102	26209.3	26209.3
Ccdc109b	Ccdc109b-001	0.0266037	0.473995	-2.10973
Ccdc109b	Ccdc109b-004	0.0460418	6.68324	6.68324
Ccdc125	Ccdc125-201	0.0165863	0.230467	-4.33902
Ccdc125	Ccdc125-202	0.00624256	8.4137	8.4137
Ccdc130	Ccdc130-006	0.0184175	2.50905	2.50905
Ccdc137	Ccdc137-005	0.0326756	2.91206e+08	2.91206e+08
Ccdc146	Ccdc146-002	0.0443471	4.47345	4.47345
Ccdc151	Ccdc151-202	0.0223817	1.97954	1.97954
Ccdc157	Ccdc157-001	0.0393506	1.50188	1.50188
Ccdc171	Ccdc171-001	0.0182	0.317028	-3.1543
Ccdc173	Ccdc173-001	0.0388838	1.49847	1.49847
Ccdc28b	Ccdc28b-009	0.0203246	1.82859	1.82859

Ccdc37	Ccdc37-005	0.021579	0.651957	-1.53384
Ccdc47	Ccdc47-004	0.037079	3.20905	3.20905
Ccdc6	Ccdc6-001	0.0456517	1.14503	1.14503
Ccdc61	Ccdc61-004	0.0260923	21.1474	21.1474
Ccdc64	Ccdc64-007	0.0332713	1.26849	1.26849
Ccdc8	Ccdc8-001	0.0432581	1.3113	1.3113
Ccdc82	Ccdc82-202	0.0283853	0.70316	-1.42215
Ccdc82	Ccdc82-201	0.00590146	1.48973	1.48973
Ccdc85a	Ccdc85a-001	0.0069989	0.722996	-1.38313
Cchcr1	Cchcr1-007	0.0422463	3.51957	3.51957
Ccl12	Ccl12-001	0.0211942	0.00152186	-657.093
Ccl27a	Ccl27a-201	0.0114201	163.58	163.58
Ccl27a	Ccl27a-001	0.0214294	256.924	256.924
Ccl6	Ccl6-002	0.00806116	2.98399	2.98399
Ccm2	Ccm2-003	0.0104385	2.8819	2.8819
Ccm2l	Ccm2l-001	0.00627773	5.3248	5.3248
Ccnd2	Ccnd2-004	0.0175273	0.502388	-1.99049
Ccnd3	Ccnd3-006	0.0282676	1.38873	1.38873
Ccndbp1	Ccndbp1-007	0.00571542	0.000757136	-1320.77
Ccnl1	Ccnl1-009	0.0073042	2.75843	2.75843
Ccr5	Ccr5-004	0.0463457	0.00078038	-1281.43
Ccr6	Ccr6-205	0.00680516	0.00245108	-407.984
Ccser1	Ccser1-002	0.0335904	0.400869	-2.49458
Ccser2	Ccser2-010	0.0359174	0.645112	-1.55012
Ccser2	Ccser2-005	0.0136543	4.35109	4.35109
Cct3	Cct3-007	0.0437777	6.70204	6.70204
Ccz1	Ccz1-003	0.0241996	2.51859	2.51859
Cd151	Cd151-203	0.0135872	1.69479	1.69479
Cd36	Cd36-002	0.0295469	0.209227	-4.77949
Cd5	Cd5-201	0.0328791	8.18476	8.18476
Cd7	Cd7-001	0.0146597	5.10934	5.10934
Cd82	Cd82-003	0.00898606	0.048388	-20.6663
Cd82	Cd82-006	0.0467593	0.0114894	-87.0368
Cd82	Cd82-004	0.0346035	2.73809e+10	2.73809e+10
Cd82	Cd82-009	0.0445678	3.24308e+10	3.24308e+10
Cd84	Cd84-001	0.0338194	0.854259	-1.1706
Cd8a	Cd8a-003	0.019846	3.08502	3.08502
Cdc23	Cdc23-006	0.0454747	2.51138	2.51138
Cdc25a	Cdc25a-002	0.0346194	2.32276e-08	-4.30522e+07
Cdc40	Cdc40-201	0.0106702	0.831956	-1.20199
Cdc42bpa	Cdc42bpa-014	0.0422371	0.684054	-1.46187
Cdc42bpa	Cdc42bpa-008	0.0233315	2.36372	2.36372
Cdc42se2	Cdc42se2-002	0.0431387	1.55e+10	1.55e+10

Cdc7	Cdc7-005	0.00165231	0.123796	-8.07779
Cdca3	Cdca3-003	0.0451626	3.41745e-05	-29261.6
Cdh10	Cdh10-001	0.0367301	0.828567	-1.2069
Cdh11	Cdh11-201	0.00548658	0.890804	-1.12258
Cdh7	Cdh7-201	0.0343765	12.2791	12.2791
Cdh9	Cdh9-001	0.0364683	0.828526	-1.20696
Cdhr4	Cdhr4-006	0.0374062	3.29872e-09	-3.03148e+08
Cdk12	Cdk12-007	0.0297013	7.90252	7.90252
Cdk2	Cdk2-201	0.0499883	0.284941	-3.5095
Cdk2	Cdk2-202	0.0463784	21.3147	21.3147
Cdk4	Cdk4-009	0.0060361	0.492866	-2.02895
Cdk5rap2	Cdk5rap2-006	0.0480953	2.32244	2.32244
Cdk7	Cdk7-201	0.0176604	0.903625	-1.10665
Cdk8	Cdk8-004	0.0245891	2.45521	2.45521
Cdkl3	Cdkl3-204	0.0127635	0.313625	-3.18853
Cdkn1a	Cdkn1a-001	0.022675	1.71382	1.71382
Cdon	Cdon-201	0.0156747	0.63231	-1.5815
Cdon	Cdon-001	0.0299162	1.61618	1.61618
Cdr2	Cdr2-001	0.0315925	2.39405	2.39405
Cdv3	Cdv3-007	0.0484159	0.0158251	-63.1907
Ceacam1	Ceacam1-004	0.0472412	0.230061	-4.34667
Celsr3	Celsr3-004	0.0210617	2.72042	2.72042
Cenpc1	Cenpc1-001	0.0352743	0.899788	-1.11137
Cenpi	Cenpi-003	0.014442	17.6449	17.6449
Cenpj	Cenpj-001	0.00291129	1.48217	1.48217
Cenpv	Cenpv-002	0.0384634	0.275453	-3.63038
Cep104	Cep104-001	0.0069911	1.18592	1.18592
Cep112	Cep112-007	0.00399912	7.69005e-05	-13003.8
Cep112	Cep112-201	0.0363419	4.33784	4.33784
Cep126	Cep126-201	0.0433391	1.19565	1.19565
Cep131	Cep131-006	0.0224493	1.42536	1.42536
Cep131	Cep131-201	0.0292804	1.37674	1.37674
Cep290	Cep290-201	0.0318487	1.18555	1.18555
Cep41	Cep41-010	0.0310366	6640.9	6640.9
Cep70	Cep70-001	0.0149157	0.811156	-1.23281
Cep83os	Cep83os-201	0.00960064	0.834323	-1.19858
Cep89	Cep89-009	0.0249805	0.157773	-6.33822
Cers3	Cers3-001	0.0243859	1.85992	1.85992
Cers4	Cers4-009	0.0316354	86.36	86.36
Cers4	Cers4-002	0.00889707	1.3107	1.3107
Cetn4	Cetn4-004	0.0074835	0.520864	-1.91989
Cfap126	Cfap126-002	0.0422563	3.58301	3.58301
Cfap46	Cfap46-007	0.00710088	7.85505	7.85505

Cfap46	Cfap46-005	0.0233528	9.3058	9.3058
Cfap69	Cfap69-002	0.0389025	291.04	291.04
Cfap70	Cfap70-001	0.0282429	16.0738	16.0738
Cflar	Cflar-007	0.0261489	0.512124	-1.95265
Cgn	Cgn-001	0.0303278	2.42938	2.42938
Cgnl1	Cgnl1-003	0.000639395	2.8811	2.8811
Chd2	Chd2-005	0.000557871	0.00122887	-813.754
Chd2	Chd2-011	0.00301621	0.0973671	-10.2704
Chd2	Chd2-013	0.031892	0.00229426	-435.87
Chdh	Chdh-001	0.0456269	1.4644	1.4644
Chfr	Chfr-005	0.0400158	2.54565	2.54565
Chid1	Chid1-004	0.0141592	1.31306	1.31306
Chil1	Chil1-002	0.0479877	1.27842	1.27842
Chka	Chka-003	0.00860197	5.03396	5.03396
Chl1	Chl1-001	0.0350594	0.891653	-1.12151
Chmp2a	Chmp2a-201	0.0217462	1.12652	1.12652
Chn1	Chn1-007	0.0161259	0.834466	-1.19837
Chn1os1	Chn1os1-001	0.0392673	0.614968	-1.6261
Chn1os3	Chn1os3-001	0.0239777	0.772096	-1.29518
Chn2	Chn2-001	0.0307711	1.70334	1.70334
Chrdl1	Chrdl1-002	0.026529	1.41402	1.41402
Chrm1	Chrm1-201	0.0156238	0.854865	-1.16978
Chrm4	Chrm4-001	0.0175943	1.5026	1.5026
Chrna2	Chrna2-001	0.0428928	0.669257	-1.49419
Chrna7	Chrna7-201	0.0242936	0.810186	-1.23428
Chst8	Chst8-004	0.00790909	0.519051	-1.92659
Chtop	Chtop-007	0.0413137	1.25748	1.25748
Ciao1	Ciao1-003	0.0122901	16.6419	16.6419
Ciao1	Ciao1-002	0.0148547	1.55955	1.55955
Cib1	Cib1-004	0.0221115	2.87228	2.87228
Cipc	Cipc-001	0.0346338	1.07892	1.07892
Cipc	Cipc-201	0.0346338	1.07892	1.07892
Cisd2	Cisd2-004	0.0298899	0.0317611	-31.4851
Cish	Cish-003	0.0280618	2.65366	2.65366
Ciz1	Ciz1-015	0.0109759	0.408451	-2.44827
Ckmt1	Ckmt1-008	0.0256958	0.0728823	-13.7208
Cks1brt	Cks1brt-201	0.0497698	0.496671	-2.0134
Clasp1	Clasp1-201	0.00196033	0.455371	-2.19601
Clasp1	Clasp1-202	0.0342074	0.277938	-3.59793
Clasp1	Clasp1-020	0.0137159	0.193124	-5.17803
Clasp1	Clasp1-003	0.0374056	0.622848	-1.60553
Clasp1	Clasp1-022	0.00988962	1.23199	1.23199
Clasp1	Clasp1-013	0.0267903	1.205	1.205

Clasp1	Clasp1-006	0.0111777	3.55041	3.55041
Clcc1	Clcc1-001	0.0476744	0.743753	-1.34453
Clcf1	Clcf1-003	0.00571561	2.17766	2.17766
Clcn1	Clcn1-007	0.0315708	9.00501	9.00501
Clcn3	Clcn3-004	0.0322636	0.702614	-1.42326
Clcn3	Clcn3-010	0.0269445	0.742465	-1.34687
Cldn1	Cldn1-001	0.0388446	3.30117	3.30117
Cldn12	Cldn12-003	0.0481508	1.72776	1.72776
Cldn3	Cldn3-001	0.0339231	4.14916	4.14916
Clec16a	Clec16a-001	0.0153461	0.902583	-1.10793
Clec4a2	Clec4a2-001	0.0016364	2.8271	2.8271
Clec5a	Clec5a-002	0.0427316	0.401924	-2.48803
Clhc1	Clhc1-002	0.00360925	0.183207	-5.45832
Clic3	Clic3-001	0.0136308	37.0522	37.0522
Clic5	Clic5-201	0.0264402	1.20187	1.20187
Clip1	Clip1-008	0.0314354	1.94066	1.94066
Clip2	Clip2-001	0.0461848	0.784578	-1.27457
Clip2	Clip2-002	0.0151317	1.11767	1.11767
Clip3	Clip3-006	0.0252513	1.06969e+09	1.06969e+09
Cln3	Cln3-017	0.0174435	0.297959	-3.35617
Cln5	Cln5-201	0.0111392	1.18092	1.18092
Cln6	Cln6-001	0.0155882	0.780604	-1.28106
Clns1a	Clns1a-001	0.019076	0.88587	-1.12883
Clstn2	Clstn2-001	0.0262684	0.873749	-1.14449
Clstn3	Clstn3-004	0.0427644	0.314634	-3.17829
Clstn3	Clstn3-002	0.0079748	1.30103	1.30103
Cltc	Cltc-005	0.0149994	0.298078	-3.35482
Cmas	Cmas-003	0.0443124	0.690963	-1.44725
Cml3	Cml3-001	0.0459929	1.35799	1.35799
Cmpk1	Cmpk1-004	0.00178633	1.27561	1.27561
Cnksr2	Cnksr2-002	0.0210159	0.849268	-1.17748
Cnksr3	Cnksr3-001	0.0074134	1.66223	1.66223
Cnnm1	Cnnm1-201	0.0285883	0.953021	-1.04929
Cnnm2	Cnnm2-201	0.0288134	1.63845	1.63845
Cnot11	Cnot11-201	0.0349048	1.1528	1.1528
Cnot2	Cnot2-009	0.0218116	0.391273	-2.55576
Cnot4	Cnot4-002	0.0434615	0.581203	-1.72057
Cnot4	Cnot4-004	0.0194412	0.366024	-2.73206
Cnot4	Cnot4-005	0.00101074	1.46777	1.46777
Cnot6	Cnot6-001	0.0114967	1.13472	1.13472
Cnot6	Cnot6-201	0.0423425	1.75815	1.75815
Cnot7	Cnot7-003	0.0296238	0.547296	-1.82717
Cnpy3	Cnpy3-002	0.0463781	2.27418	2.27418

Cnr1	Cnr1-001	0.000414998	0.829399	-1.20569
Cnst	Cnst-001	0.00054801	1.23702	1.23702
Cntln	Cntln-002	0.0330533	2.58403	2.58403
Cntn2	Cntn2-003	0.0430998	1.29022	1.29022
Cntn6	Cntn6-001	0.0130008	2.70772	2.70772
Cntrl	Cntrl-012	0.0461858	1.25179e-09	-7.98853e+08
Coa4	Coa4-003	0.0274823	2.31649	2.31649
Coa7	Coa7-004	0.0187564	1.64862	1.64862
Cog1	Cog1-001	0.00192859	0.89627	-1.11574
Cog1	Cog1-006	0.0289388	1.58176	1.58176
Cog1	Cog1-008	0.0159892	2.24093	2.24093
Cog6	Cog6-002	0.0466129	2.92595	2.92595
Coil	Coil-001	0.0310279	37.9789	37.9789
Col16a1	Col16a1-004	0.0471674	1.30213	1.30213
Col18a1	Col18a1-003	0.012069	207.261	207.261
Col19a1	Col19a1-001	0.0252518	0.526103	-1.90077
Col1a2	Col1a2-010	0.0326698	4.00819e-11	-2.49489e+10
Col26a1	Col26a1-003	0.0485084	5.36089e+06	5.36089e+06
Col2a1	Col2a1-005	0.0379526	608559	608559
Col4a3bp	Col4a3bp-202	0.0342384	0.661388	-1.51197
Col4a3bp	Col4a3bp-201	0.00515542	1.15901	1.15901
Col8a2	Col8a2-001	0.048425	6.54915	6.54915
Col9a3	Col9a3-003	0.015973	185768	185768
Col9a3	Col9a3-001	0.0484315	5.00158	5.00158
Copg2	Copg2-001	0.036709	0.877314	-1.13984
Cops5	Cops5-007	0.0301045	1.2662	1.2662
Cops7b	Cops7b-001	0.0130872	1.87923	1.87923
Coq10b	Coq10b-001	0.0104049	1.25696	1.25696
Coq5	Coq5-003	0.0116562	7.6476	7.6476
Coq6	Coq6-008	0.0359079	0.00288174	-347.012
Coq9	Coq9-006	0.0317859	1.52041	1.52041
Coro1c	Coro1c-008	0.0471493	2.07591	2.07591
Coro7	Coro7-008	0.0109629	2.37932	2.37932
Cox6a2	Cox6a2-001	0.0191001	1.61964	1.61964
Cox6b2	Cox6b2-007	0.0209198	2.68816	2.68816
Cp	Cp-011	0.0385566	6.91683	6.91683
Cpd	Cpd-001	0.0379876	0.907117	-1.10239
Cpeb4	Cpeb4-002	0.0448058	0.734524	-1.36143
Cpq	Cpq-201	0.0462752	1.69626	1.69626
Cpsf4	Cpsf4-005	0.0254087	2.54221	2.54221
Cpt1a	Cpt1a-201	0.00728221	1.44096	1.44096
Cpt2	Cpt2-006	0.0265487	4.26657	4.26657
Cramp1l	Cramp1l-201	0.0141236	1.17543	1.17543

Creb1	Creb1-010	0.03405	2.55332	2.55332
Creb3	Creb3-006	0.0488228	0.378103	-2.64478
Creb5	Creb5-010	0.00163006	0.611457	-1.63544
Crebzf	Crebzf-004	0.0196505	0.764517	-1.30801
Crem	Crem-029	0.0141574	0.0162237	-61.6382
Crem	Crem-201	0.0497828	0.0551316	-18.1384
Crip2	Crip2-001	0.0340182	1.22825	1.22825
Crlf3	Crlf3-003	0.041083	2.7887	2.7887
Crls1	Crls1-003	0.0328871	0.577554	-1.73144
Crmp1	Crmp1-001	0.0421864	0.586987	-1.70362
Cry1	Cry1-201	0.0099329	1.23599	1.23599
Cryab	Cryab-201	0.0270722	1.19347	1.19347
Cryba1	Cryba1-001	0.0421076	8.97312e-05	-11144.4
Crybg3	Crybg3-001	0.0257388	1.60163	1.60163
Cse1l	Cse1l-004	0.00383099	1.73498e-06	-576377
Csf1	Csf1-002	0.00369128	3.97907	3.97907
Csgalnact2	Csgalnact2-001	0.0246508	0.82995	-1.20489
Csnk1a1	Csnk1a1-002	0.0136582	0.707243	-1.41394
Csnk1a1	Csnk1a1-005	0.00767617	1.29426	1.29426
Csnk1d	Csnk1d-002	0.00959131	0.892837	-1.12003
Csnk1d	Csnk1d-001	0.00256366	1.38768	1.38768
Csnk1g1	Csnk1g1-014	0.00347523	0.199089	-5.02288
Csnk2b	Csnk2b-007	0.0353588	3.1834e-07	-3.14129e+06
Csnk2b	Csnk2b-006	0.00732963	3.35812	3.35812
Cspp1	Cspp1-003	0.0490073	0.157983	-6.32979
Cspp1	Cspp1-006	0.0400211	1.8986	1.8986
Csrnp3	Csrnp3-005	0.00943947	0.310176	-3.22397
Ctage5	Ctage5-006	0.0335523	0.00657802	-152.021
Ctage5	Ctage5-002	0.00244002	0.53778	-1.8595
Ctbp1	Ctbp1-009	0.0254316	9.01943	9.01943
Ctbp2	Ctbp2-003	0.0463065	6.17943	6.17943
Ctc1	Ctc1-004	0.0149538	3.70611	3.70611
Ctdsp1	Ctdsp1-002	0.031711	0.263527	-3.79468
Ctdspl	Ctdspl-001	0.00847504	1.17605	1.17605
Ctnnal1	Ctnnal1-001	0.0110427	1.60549	1.60549
Ctnnbip1	Ctnnbip1-002	0.014312	0.132694	-7.53614
Cts8	Cts8-201	0.0217232	0.448203	-2.23113
Ctsc	Ctsc-002	0.017125	0.865405	-1.15553
Ctsk	Ctsk-001	0.0394554	1.35159	1.35159
Ctso	Ctso-002	0.031069	0.572189	-1.74767
Ctu2	Ctu2-002	0.0234362	10.3934	10.3934
Cul3	Cul3-002	0.00617805	0.8929	-1.11995
Cul9	Cul9-008	0.0351051	0.546594	-1.82951

Cutal	Cutal-002	0.00912947	31.003	31.003
Cutal	Cutal-201	0.0241514	31.003	31.003
Cux1	Cux1-003	0.00126746	0.00385152	-259.637
Cux1	Cux1-004	0.0318584	1.58926	1.58926
Cwc22	Cwc22-001	0.0148369	0.226846	-4.40827
Cwh43	Cwh43-002	0.0390555	0.24272	-4.11997
Cxcl11	Cxcl11-201	0.0373611	2.7189	2.7189
Cxcr4	Cxcr4-002	0.024768	1.96719	1.96719
Cxx1a	Cxx1a-001	0.0395611	1.1258	1.1258
Cyb561	Cyb561-002	0.00968351	2.05991	2.05991
Cyb561	Cyb561-001	0.00381136	1.55926	1.55926
Cyb5d2	Cyb5d2-001	0.00250488	0.733297	-1.3637
Cyfip1	Cyfip1-011	0.0393028	0.351208	-2.84731
Cyfip1	Cyfip1-004	0.0167181	0.757172	-1.3207
Cyfip2	Cyfip2-201	0.0133293	0.891951	-1.12114
Cyp27a1	Cyp27a1-001	0.0398253	1.4815	1.4815
Cyp2j13	Cyp2j13-201	0.013769	1.77909	1.77909
Cyp4b1	Cyp4b1-003	0.0316367	0.112099	-8.92066
Cyp4v3	Cyp4v3-001	0.0446426	1.1862	1.1862
Cyp4x1os	Cyp4x1os-001	0.023916	0.472903	-2.1146
Cyp7b1	Cyp7b1-001	0.0261627	0.797631	-1.25371
Cysltr1	Cysltr1-201	0.0165385	35.0438	35.0438
Cyth2	Cyth2-004	0.0485751	4.53972	4.53972
D030055H07Rik	D030055H07Rik-003	0.00417256	8.02594	8.02594
D030055H07Rik	D030055H07Rik-001	0.0321094	2648.14	2648.14
D130040H23Rik	D130040H23Rik-201	0.0370154	0.708855	-1.41073
D17H6S53E	D17H6S53E-001	0.0143105	1.24539	1.24539
D1Ertd622e	D1Ertd622e-001	0.0491008	1.41174	1.41174
D230025D16Rik	D230025D16Rik-005	0.021624	6.53831	6.53831
D630024D03Rik	D630024D03Rik-002	0.0318987	15.8462	15.8462
D630030B08Rik	D630030B08Rik-001	0.0179172	0.255248	-3.91776
D6Wsu163e	D6Wsu163e-001	0.0138587	0.880544	-1.13566
D8Ertd738e	D8Ertd738e-201	0.0378484	1.09314	1.09314
D930015E06Rik	D930015E06Rik-001	0.0465603	0.757651	-1.31987
Daam2	Daam2-201	0.0226368	1.09535	1.09535
Dab2ip	Dab2ip-003	0.0448033	0.726932	-1.37565
Dab2ip	Dab2ip-010	0.0202086	0.472816	-2.11499
Dab2ip	Dab2ip-001	0.00080954	1.51443	1.51443
Dab2ip	Dab2ip-009	0.0158165	3.97893	3.97893
Dalrd3	Dalrd3-006	0.0159211	0.000127845	-7821.95
Dars2	Dars2-004	0.0410229	0.649369	-1.53996
Dazap1	Dazap1-001	0.0279719	0.671152	-1.48998
Dbndd2	Dbndd2-006	0.0161199	1.63125	1.63125

Dbr1	Dbr1-005	0.0326534	0.594844	-1.68111
Dcaf12	Dcaf12-001	0.0424434	1.0968	1.0968
Dcaf12l2	Dcaf12l2-001	0.00991465	0.612711	-1.63209
Dcbld1	Dcbld1-003	0.0230124	5.45169	5.45169
Dcbld2	Dcbld2-007	0.0101871	2.40367	2.40367
Dcdc2b	Dcdc2b-201	0.039243	2.59998	2.59998
Dclre1a	Dclre1a-001	0.0194299	0.719341	-1.39016
Dclre1b	Dclre1b-007	0.0489337	1.90393	1.90393
Dclre1c	Dclre1c-003	0.0151323	0.793093	-1.26089
Dctd	Dctd-004	0.042777	0.248226	-4.02859
Dctn6	Dctn6-004	0.00752188	0.718358	-1.39206
Dctn6	Dctn6-003	0.00115286	3.40686	3.40686
Dctn6	Dctn6-008	0.000145687	2.33701	2.33701
Dcun1d4	Dcun1d4-010	0.0337894	2.18855e-09	-4.56924e+08
Dda1	Dda1-001	0.0271458	0.715107	-1.39839
Dda1	Dda1-003	0.0400152	2.67748	2.67748
Dda1	Dda1-002	0.0268076	1.80416	1.80416
Ddah1	Ddah1-001	0.0461995	1.14836	1.14836
Ddhd1	Ddhd1-001	0.0469764	0.206673	-4.83855
Ddhd2	Ddhd2-201	0.0111878	0.909787	-1.09916
Ddx39b	Ddx39b-002	0.0467767	0.202164	-4.94648
Ddx47	Ddx47-001	0.0492758	1.08849	1.08849
Dedd	Dedd-004	0.0097413	0.308307	-3.24352
Dek	Dek-003	0.014227	2.32046	2.32046
Dennd1b	Dennd1b-007	0.00600362	0.297552	-3.36076
Dennd1b	Dennd1b-001	0.036182	0.698925	-1.43077
Dennd4c	Dennd4c-001	0.0183207	1.12906	1.12906
Desi1	Desi1-002	0.0197954	2.15568	2.15568
Desi2	Desi2-001	0.00922246	1.25283	1.25283
Det1	Det1-001	0.047223	1.29433	1.29433
Dffb	Dffb-001	0.0288139	0.802211	-1.24655
Dgcr8	Dgcr8-002	0.0401742	0.400613	-2.49617
Dgcr8	Dgcr8-001	0.0345003	1.21803	1.21803
Dgka	Dgka-201	0.0434242	0.885429	-1.1294
Dgke	Dgke-001	9.74064e-05	0.68501	-1.45983
Dgke	Dgke-201	0.000796268	1.47055	1.47055
Dgkg	Dgkg-004	0.0175728	0.478391	-2.09034
Dhodh	Dhodh-009	0.0156933	0.153614	-6.50984
Dhodh	Dhodh-008	0.000195041	14.9927	14.9927
Dhps	Dhps-007	0.0418752	0.149114	-6.70627
Dhrs3	Dhrs3-003	0.0221243	2.13125	2.13125
Dhrs7b	Dhrs7b-201	0.048448	1.15817	1.15817
Dhx16	Dhx16-008	0.0325198	0.00417453	-239.548

Dhx34	Dhx34-005	0.0124521	5597.2	5597.2
Dhx35	Dhx35-009	0.0498025	0.244556	-4.08905
Dhx35	Dhx35-004	0.0423046	0.630467	-1.58613
Diexf	Diexf-002	0.0378631	1.49232	1.49232
Diras2	Diras2-201	0.0372116	0.900618	-1.11035
Dkc1	Dkc1-001	0.0425103	1.0985	1.0985
Dlat	Dlat-001	0.0126793	0.885927	-1.12876
Dleu2	Dleu2-015	0.0450802	2.31385	2.31385
Dlg2	Dlg2-002	0.0230155	0.794972	-1.25791
Dlgap1	Dlgap1-003	0.0165585	0.594097	-1.68323
Dlgap1	Dlgap1-006	0.0137489	0.86545	-1.15547
Dlgap2	Dlgap2-008	0.0190268	2.05031	2.05031
Dlgap4	Dlgap4-006	0.00484641	66.8002	66.8002
Dlx1as	Dlx1as-004	0.0362577	0.814894	-1.22715
Dmap1	Dmap1-006	0.0140587	5.57991	5.57991
Dmd	Dmd-003	0.0299936	0.84191	-1.18778
Dmd	Dmd-007	0.0331106	0.419287	-2.385
Dmpk	Dmpk-011	0.035694	8.80332e-05	-11359.3
Dmtf1	Dmtf1-001	0.0019766	1.52408	1.52408
Dmxl2	Dmxl2-004	0.00097312	0.854193	-1.1707
Dmxl2	Dmxl2-008	0.00528506	2.27406	2.27406
Dnah8	Dnah8-201	0.00180231	0.617894	-1.6184
Dnaja4	Dnaja4-003	0.038219	0.154954	-6.45353
Dnajb1	Dnajb1-201	0.0398075	1.13947	1.13947
Dnajb11	Dnajb11-001	0.0395599	1.13794	1.13794
Dnajb5	Dnajb5-001	0.0182885	0.456328	-2.19141
Dnajb5	Dnajb5-201	0.0273362	1.23051	1.23051
Dnajb5	Dnajb5-006	0.0299375	1.47466	1.47466
Dnajc1	Dnajc1-005	0.0255863	0.236707	-4.22463
Dnajc10	Dnajc10-004	0.034662	0.670251	-1.49198
Dnajc13	Dnajc13-010	0.00935994	3.13686	3.13686
Dnajc17	Dnajc17-002	0.0319501	0.00710147	-140.816
Dnajc2	Dnajc2-005	0.00230427	2.17149e-07	-4.60513e+06
Dnajc25	Dnajc25-002	0.0264313	1.3296	1.3296
Dnajc28	Dnajc28-003	0.0411616	151.599	151.599
Dnajc5	Dnajc5-004	0.011093	0.0878971	-11.3769
Dnajc7	Dnajc7-007	0.0149944	1.42551	1.42551
Dnajc7	Dnajc7-004	0.0365334	1.42898	1.42898
Dnajc7	Dnajc7-005	0.0328702	25.614	25.614
Dnajc8	Dnajc8-009	0.0131291	0.126448	-7.9084
Dnajc8	Dnajc8-003	0.0286573	0.337473	-2.9632
Dnajc8	Dnajc8-002	0.0444214	1.45236	1.45236
Dnase1l2	Dnase1l2-002	0.0337507	1.64021	1.64021

Dnase1l3	Dnase1l3-001	0.0218576	0.532573	-1.87768
Dnm1	Dnm1-005	0.00338066	0.865329	-1.15563
Dnm1	Dnm1-004	0.0319729	0.755888	-1.32295
Dnm1	Dnm1-003	0.0190944	0.803327	-1.24482
Dnm1	Dnm1-008	0.0371629	2.66794	2.66794
Dnm2	Dnm2-009	0.0347913	5.89318	5.89318
Dnm2	Dnm2-005	0.0405331	1.62424	1.62424
Dnm3	Dnm3-004	0.00105503	1.52384	1.52384
Dnpep	Dnpep-008	0.0110679	0.00628997	-158.983
Dnpep	Dnpep-003	0.00219478	2.13095	2.13095
Doc2g	Doc2g-001	0.0227121	1.57619	1.57619
Dock4	Dock4-201	0.0157992	0.867837	-1.15229
Dock5	Dock5-201	0.0262194	1.26093	1.26093
Dopey1	Dopey1-011	0.0343927	0.0458539	-21.8084
Dopey1	Dopey1-016	0.0168138	1.73388	1.73388
Dpf2	Dpf2-003	0.00873073	10.1676	10.1676
Dpf2	Dpf2-005	0.0430667	1.58414	1.58414
Dph7	Dph7-003	0.036458	0.632887	-1.58006
Dpm1	Dpm1-005	0.00781632	7.51851e-06	-133005
Dpp9	Dpp9-201	0.0330445	0.913615	-1.09455
Dr1	Dr1-002	0.0217058	0.178424	-5.60462
Drc1	Drc1-003	0.0393157	16.2404	16.2404
Drg2	Drg2-005	0.0496813	1.36204	1.36204
Dscaml1	Dscaml1-201	0.0462606	0.867888	-1.15222
Dscr3	Dscr3-004	0.0208249	0.17043	-5.8675
Dsel	Dsel-004	0.0348504	1915.29	1915.29
Dst	Dst-017	0.0393936	0.180921	-5.52729
Dtl	Dtl-001	0.0297775	1.68941	1.68941
Dtnb	Dtnb-025	0.0221746	0.66517	-1.50337
Dusp15	Dusp15-001	0.0313897	1.38784	1.38784
Dusp16	Dusp16-002	0.0275528	0.0802755	-12.4571
Dusp16	Dusp16-005	0.0407153	1.97011	1.97011
Dusp23	Dusp23-001	0.029358	0.769687	-1.29923
Dvl2	Dvl2-004	0.0001217	1.85256	1.85256
Dvl2	Dvl2-003	0.0417704	4.59659	4.59659
Dync1h1	Dync1h1-008	0.0193585	0.384388	-2.60154
Dyrk1b	Dyrk1b-001	0.0148843	1.3647	1.3647
Dysf	Dysf-016	0.0363276	3.68412	3.68412
E030013I19Rik	E030013I19Rik-002	0.0237103	0.701919	-1.42466
E130111B04Rik	E130111B04Rik-001	0.00172892	0.0752784	-13.284
E130114P18Rik	E130114P18Rik-006	0.0177183	6.99621	6.99621
E130311K13Rik	E130311K13Rik-003	0.00509437	5.47138	5.47138
E230001N04Rik	E230001N04Rik-001	0.021678	0.554934	-1.80202

E2f6	E2f6-201	0.0484135	1.11521	1.11521
E330009J07Rik	E330009J07Rik-001	0.0290313	1.11116	1.11116
E4f1	E4f1-201	0.0148203	1.15092	1.15092
Eaf1	Eaf1-201	0.00223572	1.18448	1.18448
Eapp	Eapp-003	0.0136738	6332.65	6332.65
Ebag9	Ebag9-201	0.00506827	0.820598	-1.21862
Echdc1	Echdc1-002	0.0248805	1.3062	1.3062
Echdc2	Echdc2-002	0.00839535	0.291751	-3.42758
Eci1	Eci1-201	0.00741413	1.35054	1.35054
Ecscr	Ecscr-005	0.0385416	82.713	82.713
Ect2	Ect2-012	0.0350797	1.75799e+10	1.75799e+10
Eef1d	Eef1d-015	0.032391	1.77537	1.77537
Eepd1	Eepd1-001	0.0192053	1.14238	1.14238
Efcab3	Efcab3-003	0.0454163	0.235788	-4.2411
Efhc2	Efhc2-001	0.0468415	1.48428	1.48428
Efna1	Efna1-001	0.0191108	0.422128	-2.36895
Efna1	Efna1-003	0.0247566	8.39031	8.39031
Efr3a	Efr3a-011	0.0122167	0.0852054	-11.7364
Egfl7	Egfl7-019	0.0296706	0.51219	-1.9524
Egfr	Egfr-002	0.0401771	0.198071	-5.04869
Egr2	Egr2-001	0.0202495	2.89398	2.89398
Ehd1	Ehd1-004	0.0269062	0.122064	-8.19243
Ehd2	Ehd2-002	0.0191139	8.30697	8.30697
Ehd4	Ehd4-004	0.0307787	6.68831	6.68831
Ehmt1	Ehmt1-003	0.0444853	2.73788	2.73788
Ehmt2	Ehmt2-005	0.0152183	0.177338	-5.63895
Ei24	Ei24-007	0.0201559	2.73657	2.73657
Eif1ax	Eif1ax-001	0.0491777	0.91883	-1.08834
Eif2ak3	Eif2ak3-004	0.0475185	8.27203e-05	-12088.9
Eif2b5	Eif2b5-001	0.0380772	1.07146	1.07146
Eif2d	Eif2d-005	0.0107038	2.06519	2.06519
Eif2s2	Eif2s2-001	0.0478848	1.30194	1.30194
Eif3f	Eif3f-003	0.0405265	2.39693	2.39693
Eif4a2	Eif4a2-005	0.020918	1.63877	1.63877
Eif4e	Eif4e-006	0.00708464	3.20808	3.20808
Eif4enif1	Eif4enif1-010	0.0197709	18.8116	18.8116
Eif4g1	Eif4g1-014	0.0475669	5.16105	5.16105
Eif4g1	Eif4g1-004	0.0391857	1.26697	1.26697
Eif4g1	Eif4g1-016	0.0444842	6.42911	6.42911
Eif4g3	Eif4g3-010	0.0106495	0.000201003	-4975.06
Eif5a	Eif5a-004	0.0166627	0.659117	-1.51718
Eif5a	Eif5a-007	0.0436016	0.489317	-2.04367
Elac2	Elac2-004	0.0249057	0.000166737	-5997.46

Elf2	Elf2-001	0.0327232	0.582542	-1.71662
Elk4	Elk4-002	0.0232902	1.17711	1.17711
Ell	Ell-201	0.0345165	1.13094	1.13094
Elmo2	Elmo2-002	0.0115947	0.632262	-1.58162
Elmo3	Elmo3-201	0.0265661	1.56292	1.56292
Elmod3	Elmod3-004	0.0140914	0.0309465	-32.3139
Elovl2	Elovl2-001	0.0440333	0.870362	-1.14895
Elp3	Elp3-201	0.00152493	0.910027	-1.09887
Emc10	Emc10-004	0.00153042	0.285912	-3.49758
Eml6	Eml6-002	0.0316406	0.212941	-4.69614
Emp2	Emp2-001	0.0155695	1.29937	1.29937
Enah	Enah-003	0.0156543	0.92702	-1.07873
Enah	Enah-009	0.0325546	1.50952	1.50952
Enah	Enah-011	0.000715959	4.24163	4.24163
Eng	Eng-006	0.0224886	7.02276	7.02276
Eng	Eng-004	0.0468351	6.39902	6.39902
Eno4	Eno4-201	0.00446153	5.65359	5.65359
Enox1	Enox1-201	0.0234597	0.86451	-1.15672
Enox2	Enox2-002	0.000193195	0.452426	-2.2103
Enthd2	Enthd2-002	0.0233802	1.65105	1.65105
Entpd3	Entpd3-002	0.00110061	3.36959e-08	-2.96772e+07
Entpd5	Entpd5-005	0.0165959	2.73586	2.73586
Eogt	Eogt-001	0.0309555	1.17119	1.17119
Epb41	Epb41-202	0.0137857	0.345737	-2.89237
Epb41	Epb41-001	0.00935649	2.50077	2.50077
Epb41l1	Epb41l1-012	0.042476	0.383793	-2.60557
Epb41l1	Epb41l1-010	0.0234866	0.472708	-2.11547
Epb41l1	Epb41l1-005	0.0237623	1.0961	1.0961
Epb41l1	Epb41l1-007	0.0244739	1.07114	1.07114
Epb41l1	Epb41l1-002	0.0444788	1.81775	1.81775
Epc2	Epc2-001	0.0129463	0.901315	-1.10949
Epha4	Epha4-201	0.0405863	0.874181	-1.14393
Epha5	Epha5-003	0.0359117	0.740909	-1.34969
Ephb1	Ephb1-001	0.0215724	1.22202	1.22202
Ephx1	Ephx1-001	0.043191	1.40187	1.40187
Epn1	Epn1-006	0.00481224	0.169822	-5.88851
Epn3	Epn3-001	0.0277191	4.98976	4.98976
Eps8l2	Eps8l2-007	0.0080369	0.236756	-4.22375
Eps8l3	Eps8l3-001	0.00727662	0.1967	-5.08388
Eral1	Eral1-001	0.0142954	0.698786	-1.43105
Eral1	Eral1-002	0.000304643	2.02983	2.02983
Erbb2	Erbb2-003	0.0247864	0.00113587	-880.382
Erbb2ip	Erbb2ip-201	0.0387419	1.22158	1.22158

Ercc4	Ercc4-003	0.0485119	0.136636	-7.31872
Ercc4	Ercc4-005	0.0405428	2.24328	2.24328
Ercc6l	Ercc6l-001	0.0414393	3.48609	3.48609
Ergic2	Ergic2-008	0.0221572	0.403322	-2.47941
Erich4	Erich4-001	0.0273653	0.127021	-7.87272
Ermard	Ermard-201	0.000198515	0.740349	-1.35072
Esd	Esd-007	0.00450774	0.158777	-6.29813
Esm1	Esm1-001	0.00386728	9.82248	9.82248
Espn	Espn-001	0.044088	3.35701	3.35701
Esrrg	Esrrg-004	0.0307995	8.15622e+08	8.15622e+08
Etl4	Etl4-007	0.0117612	1.39748	1.39748
Etl4	Etl4-006	0.0229476	1.53306	1.53306
Etnppl	Etnppl-001	0.0478342	1.46881	1.46881
Ets1	Ets1-007	0.0342168	0.392637	-2.54688
Ets2	Ets2-004	0.00717061	0.109265	-9.15206
Etv1	Etv1-002	0.0481266	0.621046	-1.61019
Etv3	Etv3-004	0.0285244	0.302533	-3.30542
Evc2	Evc2-001	0.0430867	0.835107	-1.19745
Exoc5	Exoc5-002	0.0310539	1.80198	1.80198
Exoc6	Exoc6-201	0.0447028	0.883476	-1.13189
Exog	Exog-202	0.022424	1.27709	1.27709
Exosc10	Exosc10-001	0.0335335	0.776172	-1.28837
Exosc2	Exosc2-001	0.005823	1.11651	1.11651
Exosc4	Exosc4-002	0.0477851	0.00697344	-143.401
Eya2	Eya2-003	0.0462916	79677.8	79677.8
Ezr	Ezr-001	0.0430672	1.26815	1.26815
F730043M19Rik	F730043M19Rik-003	0.00349621	0.656946	-1.52219
F8a	F8a-001	0.0417321	0.840467	-1.18981
F9	F9-001	0.0347664	0.483603	-2.06781
Faah	Faah-001	0.0428784	0.826002	-1.21065
Fabp5	Fabp5-002	0.00646055	0.668493	-1.4959
Fabp7	Fabp7-002	0.00633823	0.248943	-4.01698
Fah	Fah-002	0.00721522	348596	348596
Fam105a	Fam105a-201	0.0349282	1.18175	1.18175
Fam107b	Fam107b-003	0.017129	0.0401192	-24.9257
Fam107b	Fam107b-002	0.0171292	0.040119	-24.9258
Fam107b	Fam107b-005	0.00172699	115.951	115.951
Fam110b	Fam110b-002	0.0315255	0.660554	-1.51388
Fam110b	Fam110b-005	0.0460185	3.82885	3.82885
Fam117a	Fam117a-001	0.0104076	1.26357	1.26357
Fam118b	Fam118b-006	0.010617	0.0119542	-83.6526
Fam118b	Fam118b-004	0.0490982	1.67549	1.67549
Fam129a	Fam129a-002	0.0484205	0.0585041	-17.0928

Fam129b	Fam129b-001	0.00547349	1.38458	1.38458
Fam132a	Fam132a-003	0.00975045	4.41088	4.41088
Fam132b	Fam132b-001	0.00999656	4.32273	4.32273
Fam134a	Fam134a-004	0.000990919	1.65504	1.65504
Fam160a1	Fam160a1-201	0.0126071	3.56732	3.56732
Fam160a1	Fam160a1-001	0.0126065	3.56733	3.56733
Fam161a	Fam161a-002	0.00970625	2.03756	2.03756
Fam171a1	Fam171a1-003	0.0261954	1.54106	1.54106
Fam171a2	Fam171a2-002	0.01321	0.538314	-1.85765
Fam171b	Fam171b-001	0.00255785	0.919261	-1.08783
Fam175a	Fam175a-007	0.00469487	0.339842	-2.94255
Fam179a	Fam179a-001	0.0400232	2.26766	2.26766
Fam185a	Fam185a-001	0.0231067	0.859972	-1.16283
Fam188a	Fam188a-019	0.00363467	0.588552	-1.69908
Fam189a2	Fam189a2-201	0.00495785	1.46328	1.46328
Fam19a4	Fam19a4-001	0.00454508	2.95617	2.95617
Fam206a	Fam206a-004	0.0454839	3.59011e+06	3.59011e+06
Fam20b	Fam20b-002	0.0186678	0.388498	-2.57402
Fam234b	Fam234b-002	0.0108992	0.359983	-2.77791
Fam3a	Fam3a-001	0.0308947	1.16093	1.16093
Fam43b	Fam43b-001	0.0351578	1.16785	1.16785
Fam46c	Fam46c-001	0.021225	1.6547	1.6547
Fam47e	Fam47e-003	0.0190737	7.43633	7.43633
Fam53b	Fam53b-004	0.00788306	0.437049	-2.28807
Fam69a	Fam69a-003	0.0260822	3.95804	3.95804
Fam69c	Fam69c-001	0.0328676	1.32946	1.32946
Fam73a	Fam73a-001	0.00653761	1.15194	1.15194
Fam76b	Fam76b-001	0.0493409	1.63108	1.63108
Fam83d	Fam83d-001	0.0317469	1.74948	1.74948
Fam92a	Fam92a-004	0.0459432	0.658854	-1.51779
Fam98c	Fam98c-006	0.0468189	5.65298	5.65298
Fanca	Fanca-011	0.00645066	3.10244	3.10244
Fancl	Fancl-003	0.0377642	0.672897	-1.48611
Fap	Fap-004	0.028384	3.83585	3.83585
Fap	Fap-008	0.0360274	11.4891	11.4891
Fap	Fap-002	0.014038	6.90773	6.90773
Fap	Fap-003	0.00904178	9.17249	9.17249
Far1	Far1-009	0.0182434	1.26748	1.26748
Far1	Far1-003	0.0296179	1.30859	1.30859
Far2os2	Far2os2-001	0.00291853	0.384814	-2.59866
Farp2	Farp2-001	0.0369449	1.33247	1.33247
Fastk	Fastk-015	0.0495841	0.286557	-3.4897
Fastk	Fastk-004	0.04422	1.32856	1.32856

Fat4	Fat4-001	0.0130692	0.80624	-1.24033
Fbln5	Fbln5-201	0.0161617	2.20259	2.20259
Fbxl2	Fbxl2-003	0.01921	2.20036	2.20036
Fbxl5	Fbxl5-002	0.0456046	0.641963	-1.55772
Fbxo22	Fbxo22-005	0.0282413	5.04787	5.04787
Fbxo30	Fbxo30-001	0.00716872	1.51079	1.51079
Fbxo34	Fbxo34-201	0.039316	0.622898	-1.6054
Fbxw15	Fbxw15-001	0.042011	1.93559	1.93559
Fbxw4	Fbxw4-009	0.0407223	17.9976	17.9976
Fbxw7	Fbxw7-003	0.0179021	0.655316	-1.52598
Fbxw9	Fbxw9-009	0.00570217	147.394	147.394
Fcgbp	Fcgbp-002	0.0407547	0.325662	-3.07067
Fcho1	Fcho1-009	0.000312657	1.9724	1.9724
Fcho2	Fcho2-202	0.00620989	0.561428	-1.78117
Fcho2	Fcho2-201	0.0464685	1.1558	1.1558
Fchsd1	Fchsd1-006	0.0480816	7.15275e+06	7.15275e+06
Fcrls	Fcrls-001	0.0233647	0.646357	-1.54713
Fdps	Fdps-007	0.0171341	0.399328	-2.50421
Fer	Fer-202	0.00910055	0.166785	-5.99574
Fgd1	Fgd1-003	0.0468739	0.147784	-6.76662
Fgd2	Fgd2-004	0.0317623	0.604522	-1.6542
Fgd4	Fgd4-003	4.42213e-05	3.27036e-05	-30577.6
Fgd4	Fgd4-009	0.0297669	0.291905	-3.42578
Fgf23	Fgf23-001	0.0369284	0.544917	-1.83514
Fgfr1op2	Fgfr1op2-006	0.00605343	4.69322	4.69322
Fgfr2	Fgfr2-017	0.0403845	5.72775	5.72775
Fgfr2	Fgfr2-012	0.0460892	7.30549	7.30549
Fgfr3	Fgfr3-006	0.0376248	0.417593	-2.39468
Fhad1	Fhad1-006	0.00476687	0.109516	-9.13105
Fhad1	Fhad1-002	0.0162096	2.64993	2.64993
Fhad1os2	Fhad1os2-001	0.0498874	2.43443	2.43443
Fhit	Fhit-001	0.00980592	18.2854	18.2854
Fis1	Fis1-005	0.00891213	2.77544	2.77544
Fkbp1a	Fkbp1a-002	0.00619739	0.7068	-1.41483
Fkbp2	Fkbp2-202	0.0101999	1.40097	1.40097
Fkbp8	Fkbp8-007	0.0195861	0.51085	-1.95752
Fkrp	Fkrp-001	0.0385958	0.908503	-1.10071
Flna	Flna-006	0.0440524	1.562	1.562
Flnc	Flnc-001	0.0311007	1.88907	1.88907
Flot1	Flot1-007	0.0323586	0.12487	-8.0083
Flywch2	Flywch2-002	0.0238342	0.0115397	-86.6576
Fmn2	Fmn2-001	0.02973	0.908026	-1.10129
Fmnl3	Fmnl3-002	0.0400937	0.263136	-3.80032

Fnbp1l	Fnbp1l-001	0.0151879	1.76076	1.76076
Fndc8	Fndc8-001	0.0441634	1.6152	1.6152
Folr1	Folr1-010	0.0250065	1.51919e+06	1.51919e+06
Folr1	Folr1-009	0.0408829	48.2525	48.2525
Fopnl	Fopnl-002	0.0242467	0.55816	-1.7916
Foxm1	Foxm1-003	0.0480594	0.000221404	-4516.64
Foxn2	Foxn2-003	0.00248479	6.85215	6.85215
Foxn3	Foxn3-008	0.00358824	4.9982	4.9982
Foxo3	Foxo3-001	0.0498241	1.14929	1.14929
Foxo4	Foxo4-001	0.00556859	1.16553	1.16553
Foxp1	Foxp1-024	6.87427e-05	0.00182291	-548.572
Foxred1	Foxred1-002	0.0384014	1.76771	1.76771
Fpgt	Fpgt-004	0.0211283	0.411764	-2.42857
Fpgt	Fpgt-001	0.00772851	0.852542	-1.17296
Frat1	Frat1-201	0.0497739	0.707239	-1.41395
Frem1	Frem1-201	0.00791928	4.76253	4.76253
Frk	Frk-201	0.0445553	2.6283	2.6283
Frmd3	Frmd3-003	0.0105663	0.574192	-1.74158
Frmd4a	Frmd4a-003	0.0233961	0.271775	-3.67951
Frmd4a	Frmd4a-006	0.0466618	0.236219	-4.23336
Frmd4b	Frmd4b-008	0.00965974	2.49676	2.49676
Frmd5	Frmd5-006	0.0183703	1.77966	1.77966
Frmd8	Frmd8-004	0.0440439	72.2975	72.2975
Frmpd1	Frmpd1-001	0.040472	0.261227	-3.82809
Frs3	Frs3-003	0.0456612	3.0698	3.0698
Fryl	Fryl-014	0.0336405	0.00505043	-198.003
Fscn1	Fscn1-001	0.0464616	1.06507	1.06507
Fscn2	Fscn2-001	0.00078317	9.50954	9.50954
Ftl1	Ftl1-001	0.0407645	1.10097	1.10097
Fuca2	Fuca2-002	0.014975	1.12715	1.12715
Fundc2	Fundc2-002	0.000394598	1.20172e-10	-8.32138e+09
Fut10	Fut10-002	0.0113569	0.540321	-1.85075
Fxn	Fxn-001	0.0293669	0.544319	-1.83716
Fxn	Fxn-002	0.0116998	3.94681	3.94681
Fxr2	Fxr2-002	0.0267603	1.83462	1.83462
Fxr2	Fxr2-003	0.0480032	2.36803	2.36803
Fyb	Fyb-006	0.0314413	3.41599e-06	-292741
Fzd10	Fzd10-001	0.0270183	0.504005	-1.98411
Fzd9	Fzd9-001	0.0339939	0.831651	-1.20243
Fzr1	Fzr1-004	0.00693829	0.669201	-1.49432
G2e3	G2e3-004	0.0310923	2.64968e-09	-3.77404e+08
G6pc3	G6pc3-002	0.0159917	1.38766	1.38766
Gaa	Gaa-001	0.0386195	0.847203	-1.18035

Gabarap	Gabarap-002	0.024509	1.24476	1.24476
Gabra4	Gabra4-002	0.00844335	0.117461	-8.51344
Gabra5	Gabra5-002	0.00227153	0.741559	-1.34851
Gabrb1	Gabrb1-001	0.0295599	0.897456	-1.11426
Gabrb2	Gabrb2-002	0.0335989	0.541775	-1.84578
Gabrb3	Gabrb3-006	0.0261211	0.840029	-1.19044
Gabrg2	Gabrg2-001	0.00375539	0.799431	-1.25089
Gabrr2	Gabrr2-003	0.0149542	46661.5	46661.5
Gale	Gale-002	0.0473808	0.281843	-3.54808
Galnt1	Galnt1-007	0.0151007	2.45091	2.45091
Galnt10	Galnt10-001	0.0415756	1.21474	1.21474
Galntl6	Galntl6-005	0.0214304	0.00156284	-639.861
Galt	Galt-005	0.0493888	0.193132	-5.1778
Galt	Galt-002	0.00763028	1.71708	1.71708
Gapdh	Gapdh-006	0.0401466	1.94173	1.94173
Gapvd1	Gapvd1-005	0.00335126	3.96113	3.96113
Garnl3	Garnl3-006	0.0447688	294924	294924
Gas2	Gas2-003	0.0405224	0.635776	-1.57288
Gas2	Gas2-002	0.0303725	2.02253	2.02253
Gas5	Gas5-012	0.049472	0.658164	-1.51938
Gas5	Gas5-011	0.0383922	0.497801	-2.00883
Gclm	Gclm-201	0.0165597	1.23544	1.23544
Gcn1l1	Gcn1l1-001	0.0152387	0.852362	-1.17321
Gdap1	Gdap1-001	0.0103707	0.910929	-1.09778
Gdf6	Gdf6-001	0.0492887	0.298654	-3.34836
Gdi1	Gdi1-003	0.0224725	1.20944	1.20944
Gemin4	Gemin4-002	0.0469484	0.120345	-8.30945
Gga2	Gga2-004	0.0274798	1.21154	1.21154
Gga2	Gga2-005	0.0419247	1.62931	1.62931
Ggnbp1	Ggnbp1-001	0.0269807	43.9938	43.9938
Ggnbp2	Ggnbp2-007	0.0253805	0.723566	-1.38204
Ghrl	Ghrl-006	0.0382481	4063.92	4063.92
Gigyf2	Gigyf2-012	0.00931943	0.747266	-1.33821
Gin1	Gin1-002	0.0414918	0.294445	-3.39622
Git2	Git2-005	0.0258915	0.642009	-1.55761
Git2	Git2-013	0.0119569	1.79709	1.79709
Gjc1	Gjc1-002	0.0407961	0.188411	-5.30756
Gjd3	Gjd3-001	0.0389197	0.553846	-1.80556
Gk5	Gk5-002	0.0407362	0.651256	-1.53549
Glb1l	Glb1l-011	0.033923	77.5215	77.5215
Glce	Glce-201	0.00704115	1.41639	1.41639
Glipr1	Glipr1-001	0.00619287	0.230479	-4.33879
Glipr1	Glipr1-201	0.0134679	0.422689	-2.3658

Glis2	Glis2-001	0.0155987	1.68829	1.68829
Glmp	Glmp-007	0.0306285	2.02864	2.02864
Glo1	Glo1-201	0.000287692	0.722923	-1.38327
Glod4	Glod4-001	0.041045	0.836213	-1.19587
Glod4	Glod4-004	2.51452e-06	32.31	32.31
Glod4	Glod4-009	0.0399437	195351	195351
Glod4	Glod4-002	0.0208608	1.49036	1.49036
Glrx	Glrx-001	0.00883302	0.84059	-1.18964
Gls	Gls-002	0.00439078	0.906634	-1.10298
Gls	Gls-014	0.0229451	0.620777	-1.61088
Glt8d2	Glt8d2-005	0.047259	3.56191e-10	-2.80748e+09
Gm10138	Gm10138-001	0.00960584	1.90915	1.90915
Gm10190	Gm10190-201	0.00514872	3.9479	3.9479
Gm10390	Gm10390-002	0.00924322	0.120188	-8.32029
Gm10390	Gm10390-001	0.0363539	0.383933	-2.60462
Gm10419	Gm10419-005	0.0111438	0.745052	-1.34219
Gm10463	Gm10463-001	0.00894084	5.8065	5.8065
Gm10463	Gm10463-201	0.00894084	5.8065	5.8065
Gm10509	Gm10509-201	0.0165948	2.494	2.494
Gm10575	Gm10575-003	0.014081	4470.37	4470.37
Gm10636	Gm10636-001	0.0394563	0.271616	-3.68166
Gm10649	Gm10649-002	0.0445458	0.69262	-1.44379
Gm10653	Gm10653-001	0.00319812	0.0583325	-17.1431
Gm10653	Gm10653-003	0.0125988	0.101279	-9.87368
Gm10710	Gm10710-001	0.0445736	0.615959	-1.62348
Gm11128	Gm11128-002	0.0496191	1.98035	1.98035
Gm11149	Gm11149-201	0.00600123	0.286698	-3.48799
Gm11346	Gm11346-004	0.0358656	2.73484e+08	2.73484e+08
Gm11410	Gm11410-001	0.00193825	0.526011	-1.9011
Gm11423	Gm11423-002	0.0460937	2.74696	2.74696
Gm11469	Gm11469-001	0.0156315	3.06675	3.06675
Gm11517	Gm11517-001	0.0456562	0.580156	-1.72367
Gm11626	Gm11626-001	0.0263967	6.90466	6.90466
Gm11855	Gm11855-001	0.0433392	1.96913	1.96913
Gm11948	Gm11948-001	0.00781229	0.48063	-2.0806
Gm11974	Gm11974-002	0.0196696	0.23717	-4.21638
Gm12103	Gm12103-001	0.045053	5.37088	5.37088
Gm12185	Gm12185-201	0.0053548	1.94367	1.94367
Gm12523	Gm12523-001	0.0393945	0.244007	-4.09825
Gm12703	Gm12703-001	0.0451938	3.99202	3.99202
Gm12749	Gm12749-001	0.020774	2.28715	2.28715
Gm12784	Gm12784-002	0.0352368	6.10526	6.10526
Gm13033	Gm13033-001	0.0117999	0.493904	-2.02469

Gm1305	Gm1305-001	0.0209508	0.371709	-2.69028
Gm13275	Gm13275-001	0.048479	4.17871e+08	4.17871e+08
Gm13276	Gm13276-001	0.048479	4.0976e+08	4.0976e+08
Gm13277	Gm13277-001	0.048479	4.0976e+08	4.0976e+08
Gm13278	Gm13278-001	0.048479	4.0976e+08	4.0976e+08
Gm13279	Gm13279-001	0.048479	4.0976e+08	4.0976e+08
Gm13289	Gm13289-001	0.048479	4.0976e+08	4.0976e+08
Gm13373	Gm13373-001	0.000523716	3.20157	3.20157
Gm13582	Gm13582-001	0.0482878	5.48954	5.48954
Gm13594	Gm13594-001	0.0334781	0.225446	-4.43565
Gm13833	Gm13833-001	0.0286572	0.149097	-6.70704
Gm13841	Gm13841-001	0.0459539	14.8878	14.8878
Gm14120	Gm14120-001	0.00410977	0.125728	-7.95366
Gm14239	Gm14239-001	0.0220256	0.253854	-3.93927
Gm14246	Gm14246-001	0.00202668	0.392341	-2.5488
Gm14257	Gm14257-001	0.0342786	0.279736	-3.5748
Gm14275	Gm14275-001	0.0457966	4.73854	4.73854
Gm14321	Gm14321-002	0.0308859	4.27638	4.27638
Gm14410	Gm14410-001	0.0341547	4.10338	4.10338
Gm14413	Gm14413-001	0.0310437	8005.17	8005.17
Gm14703	Gm14703-001	0.0497332	0.646676	-1.54637
Gm14817	Gm14817-001	0.0365205	0.524051	-1.90821
Gm15246	Gm15246-002	0.00219919	4.79864	4.79864
Gm15392	Gm15392-001	0.0491918	0.292354	-3.42051
Gm15413	Gm15413-001	0.010504	4.16589	4.16589
Gm15421	Gm15421-001	0.0069452	15.3627	15.3627
Gm15422	Gm15422-001	0.0374259	0.397109	-2.5182
Gm15487	Gm15487-001	0.0130646	0.664731	-1.50437
Gm15503	Gm15503-001	0.0403941	0.558542	-1.79038
Gm15513	Gm15513-001	0.0338538	1.42041	1.42041
Gm15535	Gm15535-002	0.044057	0.482995	-2.07042
Gm15587	Gm15587-001	0.0499368	1.85738	1.85738
Gm15718	Gm15718-001	0.0426291	6.35647	6.35647
Gm15753	Gm15753-001	0.0475569	7.6932	7.6932
Gm15758	Gm15758-001	0.00854135	0.00042499	-2353
Gm15813	Gm15813-001	0.00869758	2.2433	2.2433
Gm15842	Gm15842-001	0.0437545	0.234063	-4.27235
Gm15952	Gm15952-001	0.0265747	0.385507	-2.59399
Gm1604a	Gm1604a-201	0.0367112	1.71552	1.71552
Gm16062	Gm16062-001	0.0214811	0.382201	-2.61642
Gm16133	Gm16133-001	0.0344329	0.198679	-5.03323
Gm16159	Gm16159-001	0.0379569	8.47585	8.47585
Gm16268	Gm16268-001	0.0291531	0.531186	-1.88258

Gm16286	Gm16286-004	0.0297592	0.563855	-1.77351
Gm16294	Gm16294-001	0.00641622	0.109662	-9.11891
Gm16308	Gm16308-001	0.0153853	0.140951	-7.09466
Gm16323	Gm16323-001	0.049679	1.51466	1.51466
Gm16567	Gm16567-001	0.0304092	8.46935	8.46935
Gm16845	Gm16845-201	0.0337422	1.62131	1.62131
Gm17039	Gm17039-001	0.0214073	10.5112	10.5112
Gm17098	Gm17098-001	0.00520021	7.70627	7.70627
Gm17203	Gm17203-001	0.0379858	0.318442	-3.14029
Gm17529	Gm17529-201	0.0311749	0.598483	-1.67089
Gm17546	Gm17546-201	0.00240833	8.68799	8.68799
Gm1947	Gm1947-001	0.0125759	1.96433	1.96433
Gm19721	Gm19721-001	0.0137215	0.470233	-2.12661
Gm19744	Gm19744-002	0.0428231	2.01862	2.01862
Gm20162	Gm20162-001	0.0386022	4.8267e+08	4.8267e+08
Gm20302	Gm20302-001	0.0400161	4.39155	4.39155
Gm20319	Gm20319-003	0.0240109	58.6649	58.6649
Gm20511	Gm20511-001	0.0422678	0.402744	-2.48296
Gm20554	Gm20554-001	0.013959	9.37388e-07	-1.06679e+06
Gm20627	Gm20627-001	0.0469064	5.13178	5.13178
Gm20655	Gm20655-002	0.0392248	2.60741	2.60741
Gm20661	Gm20661-002	0.0452343	347.627	347.627
Gm20663	Gm20663-001	0.0365141	0.248117	-4.03035
Gm20683	Gm20683-001	0.0270489	1.16952	1.16952
Gm20696	Gm20696-003	0.0432214	0.0211833	-47.207
Gm20726	Gm20726-201	0.0321335	9.87115	9.87115
Gm20878	Gm20878-005	0.0483091	0.214388	-4.66445
Gm21092	Gm21092-202	0.0298418	1.52508	1.52508
Gm21816	Gm21816-001	0.00154696	0.23103	-4.32843
Gm21958	Gm21958-201	0.0478826	0.376436	-2.65649
Gm21988	Gm21988-002	0.0446614	0.486768	-2.05437
Gm21992	Gm21992-006	0.0343099	0.0289254	-34.5716
Gm22314	Gm22314-201	0.0263503	3.72782	3.72782
Gm2238	Gm2238-001	0.039139	1.51371	1.51371
Gm22820	Gm22820-201	0.00743317	0.0776964	-12.8706
Gm22952	Gm22952-201	0.0335515	6.81923	6.81923
Gm23257	Gm23257-201	0.00939616	2.10876	2.10876
Gm23356	Gm23356-201	0.0378487	8.39459e+09	8.39459e+09
Gm24119	Gm24119-201	0.0272234	0.206016	-4.85399
Gm24368	Gm24368-201	0.0436962	2.16288e+09	2.16288e+09
Gm24451	Gm24451-201	0.0457631	0.142175	-7.03358
Gm24564	Gm24564-201	0.00360477	5.96468	5.96468
Gm25212	Gm25212-201	0.0322443	3.88225e-06	-257582

Gm25266	Gm25266-201	0.047629	0.329818	-3.03198
Gm25742	Gm25742-201	0.0360824	3.17709e+08	3.17709e+08
Gm25791	Gm25791-201	0.0354658	0.318899	-3.13579
Gm25855	Gm25855-201	0.0470683	1.09617e+09	1.09617e+09
Gm2623	Gm2623-001	0.0411851	0.123722	-8.08262
Gm2639	Gm2639-001	0.00280027	4.02061	4.02061
Gm26397	Gm26397-201	0.0311858	0.197381	-5.06635
Gm26551	Gm26551-201	0.0093869	2.83901	2.83901
Gm26555	Gm26555-002	0.0499881	5.94076e-05	-16832.8
Gm26603	Gm26603-201	0.0406555	2.27385	2.27385
Gm26636	Gm26636-001	0.0158349	1.38196e+08	1.38196e+08
Gm26658	Gm26658-201	0.0318504	0.539714	-1.85283
Gm26668	Gm26668-201	0.0279353	0.523686	-1.90954
Gm26679	Gm26679-201	0.0260207	0.532322	-1.87856
Gm26711	Gm26711-201	0.0228493	1.58822	1.58822
Gm26747	Gm26747-001	0.00300571	2.39722	2.39722
Gm26773	Gm26773-201	0.0180487	1.42976	1.42976
Gm26776	Gm26776-201	0.0131424	7.04978	7.04978
Gm26793	Gm26793-201	0.000263039	0.113449	-8.81457
Gm26798	Gm26798-201	0.0347087	0.806774	-1.2395
Gm26824	Gm26824-201	0.00435391	0.471121	-2.1226
Gm26853	Gm26853-201	0.0257834	1.27086	1.27086
Gm26858	Gm26858-201	0.0103995	0.251515	-3.97591
Gm26905	Gm26905-201	0.027172	0.538612	-1.85663
Gm26945	Gm26945-001	0.0227916	1.84218	1.84218
Gm27026	Gm27026-001	0.0425167	5.80742	5.80742
Gm27608	Gm27608-201	0.0311255	14.361	14.361
Gm2762	Gm2762-008	0.00901651	0.739733	-1.35184
Gm28040	Gm28040-002	0.00918595	0.133245	-7.50494
Gm28187	Gm28187-001	0.0207586	0.501436	-1.99427
Gm28268	Gm28268-001	0.0435798	4.27559	4.27559
Gm28271	Gm28271-001	0.0393328	0.408995	-2.44502
Gm28528	Gm28528-001	0.0219943	5.98788	5.98788
Gm29427	Gm29427-001	0.0305837	0.055336	-18.0714
Gm29491	Gm29491-001	0.00408114	2.65893	2.65893
Gm29508	Gm29508-001	0.0489756	0.326883	-3.0592
Gm29508	Gm29508-002	0.00872803	13.9836	13.9836
Gm29856	Gm29856-002	0.0227157	7.76142e-07	-1.28842e+06
Gm36934	Gm36934-001	0.0400161	4.39155	4.39155
Gm36944	Gm36944-001	0.0286855	2.77568	2.77568
Gm36955	Gm36955-001	0.000477365	11.9546	11.9546
Gm37020	Gm37020-001	0.0332727	3.2342	3.2342
Gm37048	Gm37048-001	0.0381177	3.51317	3.51317

Gm37056	Gm37056-001	0.0153595	0.65265	-1.53221
Gm37158	Gm37158-001	0.0306293	0.489749	-2.04186
Gm37174	Gm37174-001	0.0252926	0.696385	-1.43599
Gm37276	Gm37276-001	0.010059	0.67574	-1.47986
Gm37373	Gm37373-001	0.0154924	0.193985	-5.15504
Gm37422	Gm37422-001	0.0356601	0.801724	-1.24731
Gm37437	Gm37437-001	0.00985424	0.129915	-7.69732
Gm37459	Gm37459-002	0.00197266	0.113668	-8.79755
Gm37488	Gm37488-001	0.0463284	0.192256	-5.20141
Gm37522	Gm37522-001	0.0355225	0.381693	-2.61991
Gm37524	Gm37524-001	0.0201619	0.129235	-7.73785
Gm37534	Gm37534-001	0.00960348	0.270453	-3.69749
Gm37569	Gm37569-001	0.0235942	0.142866	-6.99955
Gm37592	Gm37592-001	0.0288567	0.499515	-2.00194
Gm37629	Gm37629-001	0.0408936	3.45099	3.45099
Gm3764	Gm3764-008	0.0302835	0.785962	-1.27233
Gm37699	Gm37699-001	0.034227	2.09826	2.09826
Gm37755	Gm37755-001	0.0397295	0.721148	-1.38668
Gm37934	Gm37934-001	0.0097908	0.269268	-3.71377
Gm37983	Gm37983-001	0.0366081	2.83358	2.83358
Gm38071	Gm38071-001	0.00299724	0.63721	-1.56934
Gm38080	Gm38080-001	0.000189509	3.24305	3.24305
Gm38344	Gm38344-001	0.0126885	0.391692	-2.55303
Gm38357	Gm38357-001	0.0470156	1.4864	1.4864
Gm38364	Gm38364-001	0.0265093	0.232893	-4.29382
Gm4117	Gm4117-001	0.023843	0.683036	-1.46405
Gm42429	Gm42429-001	0.0392897	0.702408	-1.42367
Gm42446	Gm42446-001	0.0167319	0.279079	-3.58322
Gm42463	Gm42463-001	0.00212034	0.547785	-1.82553
Gm42480	Gm42480-001	0.0414711	4.17699	4.17699
Gm42576	Gm42576-001	0.0389708	2.807	2.807
Gm42585	Gm42585-001	0.00462319	0.450469	-2.21991
Gm42600	Gm42600-001	0.000488188	2.24892	2.24892
Gm42634	Gm42634-001	0.0331807	0.224572	-4.45291
Gm42693	Gm42693-001	0.011976	4.68336	4.68336
Gm42735	Gm42735-001	0.00769557	1.60207	1.60207
Gm42744	Gm42744-001	0.0209709	0.366142	-2.73118
Gm42802	Gm42802-001	2.72271e-05	1.46321e-10	-6.83427e+09
Gm42819	Gm42819-001	0.0276	4.21159	4.21159
Gm42820	Gm42820-001	0.0455321	0.323617	-3.09007
Gm42866	Gm42866-001	0.0489657	2.87287	2.87287
Gm42868	Gm42868-001	0.0498303	3.37179	3.37179
Gm42894	Gm42894-001	0.00602544	0.579605	-1.72531

Gm42913	Gm42913-001	0.0112898	0.56831	-1.7596
Gm42936	Gm42936-001	0.0153467	0.0539434	-18.538
Gm42967	Gm42967-001	0.0263536	1.76907	1.76907
Gm42972	Gm42972-001	0.0371751	3.33234	3.33234
Gm42993	Gm42993-001	0.021933	0.363605	-2.75024
Gm42995	Gm42995-001	0.0491918	0.292354	-3.42051
Gm42997	Gm42997-002	0.0480324	122.087	122.087
Gm43011	Gm43011-001	0.0483837	2.4056	2.4056
Gm43076	Gm43076-001	0.00933225	0.756127	-1.32253
Gm43140	Gm43140-001	0.039175	0.704032	-1.42039
Gm43162	Gm43162-001	0.0419023	2.6853	2.6853
Gm43175	Gm43175-001	0.045974	2.62661	2.62661
Gm43335	Gm43335-001	0.0356648	6.59137	6.59137
Gm43455	Gm43455-001	0.025377	1.308e-06	-764523
Gm43536	Gm43536-001	0.0434839	0.41348	-2.4185
Gm43537	Gm43537-001	0.0325314	3.20633	3.20633
Gm43573	Gm43573-001	0.0339696	4.84905e-08	-2.06226e+07
Gm43765	Gm43765-001	0.0486044	5.06306	5.06306
Gm43794	Gm43794-001	0.0473633	0.231049	-4.32809
Gm43795	Gm43795-001	0.0430684	2.65681	2.65681
Gm43860	Gm43860-001	0.0488377	1.33801	1.33801
Gm4787	Gm4787-001	0.0369453	1.7495	1.7495
Gm5141	Gm5141-201	0.0262514	0.22933	-4.36053
Gm5403	Gm5403-001	0.0436816	0.714521	-1.39954
Gm5431	Gm5431-002	0.0110444	2.46728	2.46728
Gm5535	Gm5535-201	0.0243501	0.235576	-4.24492
Gm5989	Gm5989-001	0.0491744	6.87457e-07	-1.45464e+06
Gm6123	Gm6123-001	0.04319	0.374607	-2.66946
Gm6293	Gm6293-001	0.0480205	5863.82	5863.82
Gm6483	Gm6483-003	0.0122463	1.26306	1.26306
Gm6768	Gm6768-201	0.0491918	0.292354	-3.42051
Gm7173	Gm7173-202	0.0251357	1.32365e+10	1.32365e+10
Gm7765	Gm7765-001	0.037177	5.73181	5.73181
Gm7893	Gm7893-001	0.00391034	9.33606	9.33606
Gm8091	Gm8091-001	0.0173445	4.77026	4.77026
Gm8152	Gm8152-001	0.017025	2.16745	2.16745
Gm8210	Gm8210-001	0.0459539	14.8878	14.8878
Gm8494	Gm8494-001	0.00125594	0.138336	-7.22875
Gm8668	Gm8668-001	0.0267753	8.27263	8.27263
Gm8850	Gm8850-001	0.00213768	0.185713	-5.38464
Gm8898	Gm8898-003	0.0386378	1.21284	1.21284
Gm973	Gm973-006	0.0466166	2.18505	2.18505
Gm9826	Gm9826-001	0.0320607	9.29736	9.29736

Gm9828	Gm9828-201	0.00317438	9.49843e-07	-1.05281e+06
Gm9864	Gm9864-201	0.0129985	1.21877	1.21877
Gm9910	Gm9910-001	0.0462148	2.51225	2.51225
Gm9922	Gm9922-201	0.0313486	11.0116	11.0116
Gm9947	Gm9947-001	0.0426299	0.589951	-1.69506
Gm9954	Gm9954-001	0.0147034	0.816267	-1.22509
Gmfb	Gmfb-201	0.038819	0.865002	-1.15607
Gmip	Gmip-004	0.0234866	17.777	17.777
Gna12	Gna12-001	0.0498217	1.07661	1.07661
Gnaq	Gnaq-001	0.0255484	0.907615	-1.10179
Gnas	Gnas-013	0.0447518	0.0948476	-10.5432
Gnas	Gnas-010	0.00951293	3.46623	3.46623
Gnb1l	Gnb1l-006	0.0434798	46.7613	46.7613
Gnb2	Gnb2-003	0.0150239	1.35596	1.35596
Gnb2	Gnb2-006	0.0251869	1.25789	1.25789
Gnb2	Gnb2-002	0.0163609	1.13618	1.13618
Gnb4	Gnb4-001	0.010762	1.2932	1.2932
Gnb4	Gnb4-006	0.0462985	1.92461	1.92461
Gnb4	Gnb4-004	0.0397889	1.90943	1.90943
Gng13	Gng13-001	0.0103472	3.59711	3.59711
Gng2	Gng2-001	0.0464023	0.895612	-1.11656
Gng8	Gng8-006	0.038755	5.863	5.863
Gngt2	Gngt2-007	0.0446958	1.71448e-08	-5.83266e+07
Golga1	Golga1-005	0.00326688	0.0276405	-36.1787
Golga1	Golga1-004	0.0270659	1.49159	1.49159
Golga2	Golga2-008	0.0292409	0.204907	-4.88026
Golga4	Golga4-201	0.0107089	1.1102	1.1102
Golph3l	Golph3l-003	0.029438	0.275785	-3.62601
Gorasp1	Gorasp1-201	0.0230054	1.15183	1.15183
Gpalpp1	Gpalpp1-201	0.00803646	0.945334	-1.05783
Gpank1	Gpank1-004	0.0239773	487.135	487.135
Gphn	Gphn-201	0.0323716	0.864884	-1.15622
Gpr108	Gpr108-201	0.0034836	1.21625	1.21625
Gpr141	Gpr141-201	0.0151337	1.95236	1.95236
Gpr19	Gpr19-010	0.00111024	0.2791	-3.58295
Gpr22	Gpr22-001	0.0016985	0.746057	-1.34038
Gpr22	Gpr22-002	0.0319159	0.763641	-1.30952
Gpr61	Gpr61-002	0.00628514	0.751655	-1.3304
Gpr61	Gpr61-001	0.037927	1.23217	1.23217
Gpr63	Gpr63-001	0.0306059	0.820111	-1.21935
Gpr68	Gpr68-201	0.033802	1.62149	1.62149
Gps1	Gps1-004	0.0346198	0.534607	-1.87053
Gpx1	Gpx1-003	0.0465101	1.39516	1.39516

Gpx3	Gpx3-001	0.0137174	1.48476	1.48476
Gpx8	Gpx8-201	0.0398083	3.47638	3.47638
Gramd1b	Gramd1b-007	0.0364448	0.735635	-1.35937
Gramd3	Gramd3-201	0.0307539	1.15388	1.15388
Grb10	Grb10-009	0.0197371	1.54388	1.54388
Grb14	Grb14-007	0.0425423	0.262061	-3.81591
Grb7	Grb7-003	0.00158938	53.8857	53.8857
Greb1l	Greb1l-001	0.0449042	0.838162	-1.19309
Grhpr	Grhpr-005	0.0291187	2.67712	2.67712
Gria2	Gria2-001	0.00512309	0.892931	-1.11991
Grik1	Grik1-203	0.0328887	3.44165	3.44165
Grik4	Grik4-001	0.0240079	0.788556	-1.26814
Grin1	Grin1-005	0.0288869	0.855283	-1.1692
Grin2c	Grin2c-001	0.00798971	0.84461	-1.18398
Gripap1	Gripap1-001	0.00575501	0.326515	-3.06265
Grm4	Grm4-001	0.0198448	8.34595	8.34595
Grm4	Grm4-002	0.0479966	1.84674	1.84674
Gsc	Gsc-001	0.0401521	9.34859	9.34859
Gsg1	Gsg1-003	0.0395623	5.94473	5.94473
Gspt1	Gspt1-001	0.0270438	0.877472	-1.13964
Gsta3	Gsta3-004	0.035719	0.303985	-3.28963
Gsta3	Gsta3-002	0.0138905	0.123495	-8.09748
Gsta3	Gsta3-003	0.000385767	7.27652	7.27652
Gtf2a2	Gtf2a2-001	0.0428865	2.22883	2.22883
Gtf2a2	Gtf2a2-010	0.0143811	1.43733	1.43733
Gtf2e2	Gtf2e2-201	0.010566	0.134586	-7.43017
Gtf2i	Gtf2i-004	0.0281602	0.822203	-1.21625
Gtf2i	Gtf2i-025	0.0416296	0.122695	-8.15032
Gtf2i	Gtf2i-201	0.0198079	1.41923	1.41923
Gtpbp1	Gtpbp1-201	0.0265051	1.06277	1.06277
Gucd1	Gucd1-002	0.0358908	0.323316	-3.09295
Gucy2g	Gucy2g-001	0.04419	0.65931	-1.51674
Guf1	Guf1-009	0.0464033	0.5274	-1.89609
Guf1	Guf1-001	0.019935	1.16225	1.16225
Gulp1	Gulp1-002	0.0141324	4742.21	4742.21
Gusb	Gusb-001	0.0449848	0.746208	-1.34011
Gusb	Gusb-006	0.0176296	2.61266	2.61266
Gys1	Gys1-001	0.00278475	1.24666	1.24666
H19	H19-001	0.033138	58307.5	58307.5
H2-DMb1	H2-DMb1-001	0.0378754	2.57334	2.57334
H2-K1	H2-K1-004	0.0291376	0.309513	-3.23089
H2-Q7	H2-Q7-003	0.00563791	0.557356	-1.79418
H2afy	H2afy-006	0.0399816	5.06238e-10	-1.97536e+09

H60b	H60b-201	0.0473159	7.05623	7.05623
Haao	Haao-201	0.0112885	0.477157	-2.09575
Hacd4	Hacd4-001	0.033418	0.769698	-1.29921
Harbi1	Harbi1-001	0.00228085	1.33354	1.33354
Hars2	Hars2-003	0.0380923	0.00628997	-158.983
Hars2	Hars2-002	0.00790384	1.4911	1.4911
Hba-a1	Hba-a1-001	0.0485378	2.92222	2.92222
Hbegf	Hbegf-201	0.0360395	1.2553	1.2553
Hcar1	Hcar1-001	0.0355141	0.581364	-1.72009
Hcfc1r1	Hcfc1r1-003	0.00661758	0.716567	-1.39554
Hcfc1r1	Hcfc1r1-002	0.00168018	2.29915	2.29915
Hcls1	Hcls1-001	0.0140255	1.38855	1.38855
Hdac11	Hdac11-002	0.0308911	0.26259	-3.80822
Hdac6	Hdac6-005	0.0185336	6.91241	6.91241
Hdgfrp3	Hdgfrp3-003	0.011862	1.57102	1.57102
Hectd1	Hectd1-202	0.0394164	0.766602	-1.30446
Heg1	Heg1-001	0.0308226	1.37045	1.37045
Helz2	Helz2-004	0.0201716	1.7579	1.7579
Hemk1	Hemk1-002	0.0166835	0.63106	-1.58464
Hemk1	Hemk1-008	0.0288446	2.24048	2.24048
Henmt1	Henmt1-006	0.0306546	2.07148e-06	-482748
Herc1	Herc1-003	0.0491028	1.59952	1.59952
Herc6	Herc6-004	0.0221482	114.687	114.687
Herpud1	Herpud1-001	0.0261399	1.33342	1.33342
Hexim2	Hexim2-001	0.0131904	0.673381	-1.48504
Hexim2	Hexim2-002	0.00781843	2.25525	2.25525
Hey1	Hey1-001	0.0146826	1.09891	1.09891
Hey2	Hey2-001	0.0167784	1.38053	1.38053
Hfm1	Hfm1-002	0.0332796	2.4121	2.4121
Hgf	Hgf-004	0.0201441	6.47012	6.47012
Hgsnat	Hgsnat-201	0.0445595	0.933855	-1.07083
Hhat	Hhat-006	0.0397045	17.4264	17.4264
Hiatl1	Hiatl1-001	0.042633	1.39968	1.39968
Hibadh	Hibadh-002	0.0121367	0.181285	-5.51618
Hip1	Hip1-008	0.0197829	411724	411724
Hira	Hira-004	0.012748	1.85735	1.85735
Hist1h3h	Hist1h3h-001	0.0430767	23.6035	23.6035
Hist2h3c2	Hist2h3c2-001	0.0178856	1.70236	1.70236
Hk3	Hk3-003	0.0111907	4.56307	4.56307
Hmgcr	Hmgcr-005	0.0490011	2.91814	2.91814
Hmgcs2	Hmgcs2-001	0.0266775	1.52452	1.52452
Hmgn2	Hmgn2-013	0.0141231	4.20703	4.20703
Hn1	Hn1-002	0.00578076	0.277775	-3.60004

Hnf1b	Hnf1b-003	0.000127228	0.110068	-9.08528
Hnf1b	Hnf1b-002	0.0167253	4.10685	4.10685
Hnmt	Hnmt-001	0.0357965	1.43955	1.43955
Hnrnph1	Hnrnph1-012	0.038711	0.000716552	-1395.57
Hnrnph1	Hnrnph1-017	0.0413044	1.49344	1.49344
Hnrnpk	Hnrnpk-013	0.00744143	2.62793e-10	-3.80527e+09
Hnrnpk	Hnrnpk-019	0.0116838	1.44936	1.44936
Hnrnpll	Hnrnpll-002	0.0180661	0.34931	-2.86279
Hnrnpr	Hnrnpr-002	0.0230682	0.864809	-1.15633
Hoga1	Hoga1-001	0.0191359	0.661683	-1.5113
Hook1	Hook1-005	0.0422202	0.599963	-1.66677
Hook1	Hook1-001	0.0303094	1.24892	1.24892
Hook3	Hook3-003	0.0293888	0.198504	-5.03768
Hoxc8	Hoxc8-001	0.0394886	4.94122	4.94122
Hp1bp3	Hp1bp3-010	0.020686	1.85259	1.85259
Hpca	Hpca-007	0.0424524	0.571187	-1.75074
Hpse	Hpse-002	0.0409605	525560	525560
Hrk	Hrk-001	0.0295117	1.1454	1.1454
Hsd11b1	Hsd11b1-001	0.0139717	0.57586	-1.73653
Hsd17b10	Hsd17b10-001	0.0376855	0.894083	-1.11847
Hsd3b6	Hsd3b6-201	0.0234366	1.07162e-07	-9.3317e+06
Hsdl1	Hsdl1-201	0.0398215	1.10937	1.10937
Hsp90b1	Hsp90b1-005	0.0468291	4.40909	4.40909
Hspa12a	Hspa12a-201	0.00494004	0.867106	-1.15326
Hspa12b	Hspa12b-001	0.0447029	1.38574	1.38574
Hsph1	Hsph1-006	0.00525097	2.84466	2.84466
Htr3a	Htr3a-201	0.0207258	0.758706	-1.31803
Htt	Htt-002	0.0442901	3.05973	3.05973
Hus1	Hus1-001	0.0177351	1.51352	1.51352
Hvcn1	Hvcn1-004	0.0308305	1.69709e+08	1.69709e+08
Hyi	Hyi-201	0.0147872	0.68129	-1.4678
Hyi	Hyi-001	0.0261443	2.29154	2.29154
Hyls1	Hyls1-001	0.0208155	0.792128	-1.26242
Hyls1	Hyls1-002	0.0112218	0.56968	-1.75537
Hyou1	Hyou1-005	0.0452161	4.36726e-07	-2.28977e+06
Hyou1	Hyou1-008	0.0167652	9.91108	9.91108
Ica1	Ica1-005	0.0284877	0.193924	-5.15667
Ica1	Ica1-006	0.0444849	1.46778	1.46778
Ict1	Ict1-002	0.0449114	0.797876	-1.25333
Id1	Id1-002	0.00663812	5.1455	5.1455
Id3	Id3-002	0.0474697	2.02681	2.02681
Id4	Id4-201	0.0490596	1.50068	1.50068
Idh3b	Idh3b-001	0.0301033	0.944493	-1.05877

Ido1	Ido1-001	0.039957	9.22321	9.22321
Ido2	Ido2-001	0.0338656	13.4218	13.4218
Ier3	Ier3-001	0.0319466	1.29848	1.29848
Iffo2	Iffo2-201	0.0310007	2.50376	2.50376
Ifi203	Ifi203-002	0.0338643	0.708783	-1.41087
Ifi27l2b	Ifi27l2b-001	0.02026	9.97575	9.97575
Ifitm2	Ifitm2-201	0.0251628	1.42816	1.42816
Ifnar2	Ifnar2-002	0.0298133	1.32661	1.32661
Ifngr2	Ifngr2-003	0.0221166	0.495511	-2.01812
Ifrd2	Ifrd2-005	0.0441248	2.64036	2.64036
Ift22	Ift22-002	0.0289861	1.49147	1.49147
Ift46	Ift46-002	0.0295669	1.1313	1.1313
Ift81	Ift81-007	0.049109	0.0213562	-46.8248
Igf2	Igf2-008	0.0446012	3.28384	3.28384
Igf2	Igf2-001	0.0475682	3.66641	3.66641
Igfbp2	Igfbp2-002	0.0196891	9.03922	9.03922
Igfbp4	Igfbp4-001	0.0497449	0.874733	-1.14321
Igfbp7	Igfbp7-001	0.0351579	1.60632	1.60632
Igfn1	Igfn1-002	0.0264121	3.83924	3.83924
Ighm	Ighm-002	0.0470012	0.7474	-1.33797
Igsf3	Igsf3-002	0.0101166	11.186	11.186
Igsf5	Igsf5-002	0.0312229	7.01511	7.01511
Igsf9	Igsf9-012	0.014563	0.245529	-4.07283
Il17rc	Il17rc-002	0.0107123	2.70153	2.70153
Il1r2	Il1r2-004	0.049549	0.209669	-4.76942
Il1rapl1	Il1rapl1-201	0.00390709	19.251	19.251
Il2ra	Il2ra-001	0.0111701	1.34606	1.34606
Il6st	Il6st-001	0.00959562	1.20762	1.20762
Ilkap	Ilkap-012	0.0120414	2.97524	2.97524
Ilkap	Ilkap-002	0.0217325	2.29356	2.29356
Ilvbl	Ilvbl-201	0.0135767	1.15057	1.15057
Imp4	Imp4-001	0.042204	1.14382	1.14382
Inadl	Inadl-010	0.0126902	9.11799	9.11799
Inadl	Inadl-006	0.0178602	9.00347	9.00347
Ing3	Ing3-002	0.0173859	0.240339	-4.1608
Ing3	Ing3-003	0.0339925	1.55138	1.55138
Inpp4b	Inpp4b-005	2.36232e-05	0.0309486	-32.3117
Inpp5a	Inpp5a-003	0.0471223	0.437213	-2.28722
Inpp5e	Inpp5e-005	0.0444969	1.77793	1.77793
Inpp5k	Inpp5k-001	0.011405	0.666291	-1.50084
Inpp5k	Inpp5k-003	0.0417085	1.66453	1.66453
Inpp5k	Inpp5k-006	0.0297423	1.7639	1.7639
Insig2	Insig2-014	0.0344257	36.5613	36.5613

Ints12	Ints12-002	0.0417714	0.41336	-2.4192
Ints12	Ints12-003	0.0204694	20.0406	20.0406
Ints3	Ints3-004	0.0107801	0.308868	-3.23763
Ints3	Ints3-006	0.0156409	1.15942e-06	-862501
Ints8	Ints8-006	0.0431494	0.490536	-2.03859
Intu	Intu-001	0.0420728	1.17715	1.17715
Ip6k2	Ip6k2-001	0.0266771	0.652897	-1.53164
Ipo13	Ipo13-002	0.0221008	0.0260055	-38.4535
Ipo5	Ipo5-201	0.0394344	0.9126	-1.09577
Ipo9	Ipo9-007	0.0150757	0.712118	-1.40426
Iqcf3	Iqcf3-003	0.030741	0.000632145	-1581.92
Iqgap1	Iqgap1-005	0.0158869	2.15126	2.15126
Iqgap1	Iqgap1-002	0.0409338	2.39523	2.39523
Iqsec1	Iqsec1-001	0.0187373	0.664664	-1.50452
Iqsec1	Iqsec1-003	0.0318563	6.5204e+08	6.5204e+08
Iqub	Iqub-001	0.0305758	2.17018	2.17018
Irf7	Irf7-012	0.036339	0.025166	-39.7362
Irx5	Irx5-001	0.00146219	3.15717	3.15717
Isca1	Isca1-002	0.0362978	0.473384	-2.11245
Isl1	Isl1-002	0.0234574	1.45796e+09	1.45796e+09
Itfg2	Itfg2-004	0.000773854	7.71488	7.71488
Itga2b	Itga2b-007	0.00687138	0.000128217	-7799.3
Itga4	Itga4-001	0.0107843	0.801127	-1.24824
Itga5	Itga5-201	0.0121258	3.29011	3.29011
Itga6	Itga6-002	0.00481359	2.1306	2.1306
Itga8	Itga8-001	0.0159871	0.773182	-1.29336
Itgax	Itgax-004	0.0381588	4.33928e-08	-2.30453e+07
Itgb1bp1	Itgb1bp1-007	0.0131344	2.57272	2.57272
Itgb4	Itgb4-003	0.0192851	0.394236	-2.53655
Itgb4	Itgb4-002	0.0389595	1.54578	1.54578
Itpr2	Itpr2-001	0.0499529	0.925359	-1.08066
Itpr2	Itpr2-008	0.0272235	24.6236	24.6236
Itpripl1	Itpripl1-001	0.0410832	2.18266	2.18266
Ivns1abp	Ivns1abp-002	0.0135321	1.73906	1.73906
Ivns1abp	Ivns1abp-003	0.0452941	1.67712	1.67712
Jak2	Jak2-202	0.00931582	1.22852	1.22852
Jakmip1	Jakmip1-001	0.0186425	1.18508	1.18508
Jazf1	Jazf1-002	0.010647	0.315139	-3.17321
Jazf1	Jazf1-003	0.0210114	0.291666	-3.42858
Jazf1	Jazf1-001	0.0179556	0.874524	-1.14348
Jmjd7	Jmjd7-004	0.00142417	0.0592142	-16.8878
Josd1	Josd1-201	0.00464289	1.12263	1.12263
Josd2	Josd2-021	0.0467716	0.00130697	-765.131

Jpx	Jpx-001	0.0370321	0.828615	-1.20683
Jrk	Jrk-001	0.0389341	0.821893	-1.2167
Kalrn	Kalrn-013	0.0227277	0.225718	-4.4303
Kalrn	Kalrn-004	0.0230076	0.768814	-1.30071
Kank1	Kank1-004	0.0319398	6.10707	6.10707
Kansl1l	Kansl1l-005	0.0445918	5.19526	5.19526
Kansl3	Kansl3-004	0.00704345	0.394956	-2.53193
Kansl3	Kansl3-003	0.0153924	1.93815	1.93815
Kars	Kars-201	0.0305193	0.875285	-1.14248
Kars	Kars-203	0.0183968	2.30255	2.30255
Katnal2	Katnal2-002	0.0360953	0.229273	-4.36161
Katnal2	Katnal2-007	0.0208264	15.5551	15.5551
Kazn	Kazn-002	0.0206846	1.38704	1.38704
Kbtbd2	Kbtbd2-002	0.0422524	0.574491	-1.74067
Kcnd3	Kcnd3-002	0.0198511	0.890091	-1.12348
Kcne2	Kcne2-001	0.0384792	38.1084	38.1084
Kcne3	Kcne3-204	0.0298969	0.1324	-7.55289
Kcng2	Kcng2-001	0.0399275	0.755954	-1.32283
Kcnh2	Kcnh2-002	0.0434253	3.27457	3.27457
Kcnip4	Kcnip4-002	0.00683334	0.633728	-1.57796
Kcnip4	Kcnip4-003	0.0316354	3.27207	3.27207
Kcnj3	Kcnj3-002	0.0426681	0.796756	-1.25509
Kcnk10	Kcnk10-201	0.000601566	0.679206	-1.47231
Kcnk7	Kcnk7-001	0.00923966	7.14098	7.14098
Kcnma1	Kcnma1-003	0.0210533	0.576526	-1.73453
Kcnma1	Kcnma1-006	0.00141945	2.29559	2.29559
Kcnma1	Kcnma1-209	0.0472077	2.52247	2.52247
Kcnmb2	Kcnmb2-002	0.0319275	0.431771	-2.31604
Kcnmb4	Kcnmb4-004	0.0313153	11.1661	11.1661
Kcnt1	Kcnt1-002	0.0334728	9.9038	9.9038
Kcnt2	Kcnt2-002	0.00093777	0.775726	-1.28912
Kcnv1	Kcnv1-201	0.0422372	0.849229	-1.17754
Kctd14	Kctd14-202	0.0194799	0.125262	-7.98325
Kctd14	Kctd14-004	0.0116881	2.3356	2.3356
Kctd18	Kctd18-006	0.0414254	122.811	122.811
Kctd7	Kctd7-001	0.0357015	1.22218	1.22218
Kctd9	Kctd9-003	0.0322178	12.5023	12.5023
Kdm2a	Kdm2a-201	0.0132943	1.11914	1.11914
Kdm2b	Kdm2b-007	0.0410902	2.57159	2.57159
Kdm5a	Kdm5a-007	0.0138478	0.15382	-6.5011
Kdm5a	Kdm5a-008	0.00302353	1.37658	1.37658
Kdm5a	Kdm5a-001	0.0346348	1.08542	1.08542
Kdm5b	Kdm5b-001	0.0147304	1.13056	1.13056

Kdm7a	Kdm7a-002	0.0371376	1.90145	1.90145
Khk	Khk-004	0.0195196	0.44236	-2.2606
Khk	Khk-003	0.0140196	0.32842	-3.04488
Khk	Khk-002	0.0132863	5.55123	5.55123
Kif24	Kif24-001	0.0148595	1.33798	1.33798
Kif27	Kif27-201	0.00525185	1.63553	1.63553
Kif2a	Kif2a-007	0.0210185	0.492235	-2.03155
Kif3a	Kif3a-006	0.00302898	0.692605	-1.44382
Kif4	Kif4-001	0.0204487	1.51333	1.51333
Kifap3	Kifap3-001	0.0423314	0.902769	-1.1077
Kirrel	Kirrel-001	0.0476385	1.58453	1.58453
Kit	Kit-008	0.0360327	0.459884	-2.17446
Kl	Kl-002	0.032381	7.21861	7.21861
Klc1	Klc1-009	0.0201247	1.48167	1.48167
Klf3	Klf3-001	0.0281796	0.913066	-1.09521
Klf8	Klf8-002	0.0206988	0.616345	-1.62247
Klhdc1	Klhdc1-001	0.0484038	0.879344	-1.13721
Klhdc10	Klhdc10-003	0.0446241	0.00309243	-323.371
Klhdc10	Klhdc10-007	0.0415849	3.78088	3.78088
Klhdc4	Klhdc4-006	0.030932	1.00042e-10	-9.99577e+09
Klhl12	Klhl12-001	0.0181187	0.385062	-2.59698
Klhl14	Klhl14-001	0.0334668	70.7621	70.7621
Klhl15	Klhl15-002	0.0424498	23.7411	23.7411
Klhl40	Klhl40-201	0.0219319	0.733791	-1.36279
Klhl42	Klhl42-001	0.0308639	1.09364	1.09364
Klhl7	Klhl7-002	0.0192054	1.09862	1.09862
Klri2	Klri2-001	0.0219968	2.47439	2.47439
Kmt2c	Kmt2c-003	0.0495722	0.254509	-3.92913
Kmt2c	Kmt2c-007	0.0299099	15.9817	15.9817
Kntc1	Kntc1-003	0.0301912	3.84057e-05	-26037.8
Kptn	Kptn-006	0.0465761	0.365436	-2.73646
Kras	Kras-004	0.00367964	0.173463	-5.76492
Krt12	Krt12-001	0.00235725	2.69247	2.69247
Krt20	Krt20-001	0.030831	1.82893	1.82893
Krtcap3	Krtcap3-009	4.52009e-05	12.6397	12.6397
Lactb	Lactb-201	0.0038259	1.19827	1.19827
Lair1	Lair1-002	0.0407528	8.00247	8.00247
Lama5	Lama5-001	0.0475634	1.56886	1.56886
Lamb1	Lamb1-007	0.00834165	3.91463	3.91463
Lamp2	Lamp2-005	0.00131656	2.96537	2.96537
Lamtor3	Lamtor3-008	0.0373389	1.61073	1.61073
Las1l	Las1l-007	0.0392262	1.71144	1.71144
Lbp	Lbp-001	0.0412819	5.79712	5.79712

Lca5	Lca5-006	0.0328347	6.30814	6.30814
Lck	Lck-004	0.0380249	0.149345	-6.69592
Lcn2	Lcn2-005	0.0449897	946.797	946.797
Lcp1	Lcp1-008	0.0378433	0.265854	-3.76147
Lcp2	Lcp2-002	0.0200151	2.39784	2.39784
Ldb2	Ldb2-006	0.025766	0.348697	-2.86782
Ldhb	Ldhb-001	0.0130118	1.10368	1.10368
Ldhd	Ldhd-001	0.0139327	0.695585	-1.43764
Ldhd	Ldhd-003	0.0305787	1.54836	1.54836
Ldlrad3	Ldlrad3-003	0.0350515	5.23693	5.23693
Ldlrap1	Ldlrap1-001	0.0338546	1.40751	1.40751
Ldoc1l	Ldoc1l-201	0.00390888	0.920246	-1.08667
Lect1	Lect1-005	0.0347214	0.232248	-4.30575
Leo1	Leo1-201	0.0409809	1.07608	1.07608
Lgals2	Lgals2-201	0.0140704	2.24248	2.24248
Lgals3	Lgals3-001	0.0307053	1.75429e-07	-5.7003e+06
Lgi1	Lgi1-009	0.0243564	0.914543	-1.09344
Lhb	Lhb-001	0.0352001	3.36006	3.36006
Lhfpl3	Lhfpl3-002	0.00441604	1.3968	1.3968
Lhx9	Lhx9-005	0.0295642	0.430118	-2.32494
Limch1	Limch1-016	0.0357179	8.75861	8.75861
Lime1	Lime1-004	0.0209765	0.544178	-1.83764
Lin7b	Lin7b-005	0.0318646	1.55296	1.55296
Lincpint	Lincpint-004	0.00341018	1.09704e-07	-9.1154e+06
Lingo2	Lingo2-004	0.0421955	0.0014701	-680.225
Lingo3	Lingo3-201	0.0278047	1.31344	1.31344
Lipe	Lipe-007	0.019723	0.401915	-2.48809
Lipe	Lipe-001	0.00834925	2.06668	2.06668
Lipe	Lipe-005	0.00310782	16.6279	16.6279
Lmln	Lmln-004	0.04224	64.1536	64.1536
Lmntd2	Lmntd2-201	0.0403296	2.8723	2.8723
Lmo2	Lmo2-004	0.0298607	0.211418	-4.72996
Lnp	Lnp-004	0.0217362	0.282804	-3.53602
Lnp	Lnp-012	0.0441309	0.0116334	-85.959
Lonp2	Lonp2-001	0.0144622	0.880439	-1.1358
Lonrf2	Lonrf2-201	0.0397157	0.801882	-1.24707
Lpar1	Lpar1-005	0.0162098	1.31322	1.31322
Lpar2	Lpar2-201	0.0373672	1.92203	1.92203
Lpl	Lpl-002	0.0451175	4.11947	4.11947
Lpo	Lpo-001	0.041884	3.02004	3.02004
Lppr3	Lppr3-004	0.0490466	2.08242	2.08242
Lrch4	Lrch4-007	0.0330565	0.242823	-4.11823
Lrfn5	Lrfn5-001	0.0474186	0.889487	-1.12424

Lrif1	Lrif1-002	0.0329663	0.599113	-1.66913
Lrp11	Lrp11-001	0.00835457	0.837001	-1.19474
Lrp1b	Lrp1b-005	0.00401095	0.324289	-3.08367
Lrp1b	Lrp1b-009	0.0414531	27.934	27.934
Lrp2bp	Lrp2bp-005	0.00667751	0.0593568	-16.8473
Lrp4	Lrp4-003	0.0187017	0.362973	-2.75502
Lrp6	Lrp6-001	0.0389196	1.16832	1.16832
Lrpap1	Lrpap1-004	0.0445295	1.25548	1.25548
Lrpprc	Lrpprc-005	0.000552665	0.495338	-2.01882
Lrrc10	Lrrc10-201	0.0236859	0.272267	-3.67287
Lrrc14	Lrrc14-001	0.0313456	0.662368	-1.50974
Lrrc14	Lrrc14-003	0.0452145	0.489221	-2.04407
Lrrc18	Lrrc18-001	0.0195041	2.08825	2.08825
Lrrc26	Lrrc26-001	0.0208881	0.718556	-1.39168
Lrrc38	Lrrc38-001	0.0384092	1.57655	1.57655
Lrrc40	Lrrc40-005	0.00777409	0.138434	-7.22363
Lrrc51	Lrrc51-007	0.026227	0.0515003	-19.4174
Lrrc51	Lrrc51-005	0.0310707	0.0271798	-36.792
Lrrcc1	Lrrcc1-001	0.0304665	0.839581	-1.19107
Lrriq1	Lrriq1-001	0.0483249	26.7022	26.7022
Lrrn3	Lrrn3-201	0.0103012	0.904644	-1.10541
Lrtm2	Lrtm2-201	0.0160601	2.09071	2.09071
Lsamp	Lsamp-003	0.00265589	0.655248	-1.52614
Lsamp	Lsamp-002	0.00310293	0.894021	-1.11854
Lsg1	Lsg1-004	0.0192856	1.26394	1.26394
Lsm6	Lsm6-002	0.0116291	1.26081	1.26081
Lsp1	Lsp1-008	0.0306749	6.96697	6.96697
Ltbp1	Ltbp1-007	0.0118613	0.450602	-2.21925
Ltbp1	Ltbp1-002	0.0397729	2.38128	2.38128
Ltbp3	Ltbp3-201	0.0192751	1.41918	1.41918
Ltbr	Ltbr-001	0.00823127	1.36893	1.36893
Luc7l	Luc7l-007	0.0468825	10.9589	10.9589
Luc7l2	Luc7l2-004	0.0342573	0.00251657	-397.367
Luc7l3	Luc7l3-002	0.00359094	2.34031	2.34031
Ly6e	Ly6e-013	0.0152874	2.65883	2.65883
Lypd1	Lypd1-002	0.0232734	0.832945	-1.20056
Lypd8	Lypd8-001	0.0497005	0.192256	-5.20141
Lypla1	Lypla1-001	0.0427415	1.14065	1.14065
Lysmd4	Lysmd4-202	0.0250502	0.727066	-1.37539
Lztr1	Lztr1-001	0.04873	0.539024	-1.8552
Lzts1	Lzts1-001	0.0128409	1.21699	1.21699
Lzts1	Lzts1-003	0.0441549	2.20044	2.20044
Maff	Maff-202	0.0167243	2.99168	2.99168

Maged2	Maged2-201	0.049693	0.745343	-1.34166
Magee2	Magee2-001	0.0428328	0.866155	-1.15453
Magi2	Magi2-010	0.0394792	7.11163	7.11163
Magt1	Magt1-201	0.00354249	0.580564	-1.72246
Magt1	Magt1-001	0.00570555	1.4189	1.4189
Mal	Mal-001	0.0202699	1.15395	1.15395
Mal2	Mal2-201	0.0460253	0.863658	-1.15787
Malsu1	Malsu1-001	0.00991636	0.58737	-1.7025
Mamstr	Mamstr-003	0.0305157	0.524139	-1.90789
Man2c1	Man2c1-005	0.0344097	0.573505	-1.74366
Man2c1	Man2c1-002	0.0175398	2.4229	2.4229
Man2c1os	Man2c1os-001	0.00405721	0.547324	-1.82707
Manf	Manf-002	0.00484148	1.44522	1.44522
Manf	Manf-004	0.0424642	1.78427	1.78427
Manf	Manf-007	0.0283643	4.37321	4.37321
Map2	Map2-201	0.00101459	0.894769	-1.11761
Map2	Map2-018	0.0436094	1.82201	1.82201
Map2k4	Map2k4-005	0.0473018	0.921038	-1.08573
Map2k6	Map2k6-003	0.026648	3.48542	3.48542
Map2k7	Map2k7-002	0.0284346	0.751889	-1.32998
Map2k7	Map2k7-004	0.0323466	4.34042	4.34042
Map3k11	Map3k11-201	0.00474989	1.16894	1.16894
Map3k5	Map3k5-006	0.0485073	1.78699e+06	1.78699e+06
Map4k2	Map4k2-017	0.0461158	363.801	363.801
Mapk1	Mapk1-004	0.0447469	1.75206	1.75206
Mapk10	Mapk10-004	0.00253488	0.481116	-2.0785
Mapk10	Mapk10-006	0.0371496	0.554839	-1.80232
Mapk4	Mapk4-001	0.0180978	1.28182	1.28182
Mapk8ip2	Mapk8ip2-001	0.0264326	0.939061	-1.06489
Mapk8ip3	Mapk8ip3-007	0.0195804	0.533551	-1.87424
Mapk8ip3	Mapk8ip3-008	0.00133307	0.38164	-2.62027
Mapk8ip3	Mapk8ip3-003	0.0242753	0.635199	-1.57431
Mapk9	Mapk9-202	0.0479435	0.774511	-1.29114
Mapkapk5	Mapkapk5-013	0.0145984	0.135543	-7.37773
Mapkapk5	Mapkapk5-006	0.0116426	2.06372	2.06372
Mapt	Mapt-001	0.0333272	1.07207	1.07207
Mapt	Mapt-003	0.0298696	1.66551	1.66551
Mapt	Mapt-012	0.0447753	11.3469	11.3469
01-Mar	March1-001	0.0202535	0.42704	-2.3417
March1	March1-202	0.0380002	0.551609	-1.81288
Marc1	Marc1-201	0.027415	4.82154	4.82154
Marc1	Marc1-001	0.0274151	4.82153	4.82153
Marc2	Marc2-009	0.0424204	2.35201	2.35201

March2	March2-001	0.0285428	1.31312	1.31312
March3	March3-002	0.0413584	2.64598	2.64598
March6	March6-201	0.00151961	0.923702	-1.0826
March10	March10-201	0.0421756	0.422782	-2.36529
Mark2	Mark2-011	0.00140575	4.01808e-07	-2.48875e+06
Marveld1	Marveld1-001	0.0487161	1.56238	1.56238
Marveld3	Marveld3-001	0.0175077	18.774	18.774
Mas1	Mas1-006	0.0200259	0.497231	-2.01114
Mast1	Mast1-003	0.036648	0.592184	-1.68866
Mast4	Mast4-018	0.0214357	0.461444	-2.16711
Matk	Matk-011	0.0461878	0.888758	-1.12517
Matk	Matk-010	0.0037599	11.1146	11.1146
Matr3	Matr3-011	0.00766639	0.000184775	-5411.99
Matr3	Matr3-005	0.0139607	0.172799	-5.78707
Matr3	Matr3-006	0.00447453	5.52636	5.52636
Mbd4	Mbd4-003	0.0265113	1.7384	1.7384
Mblac2	Mblac2-201	0.00190755	0.891967	-1.12112
Mbp	Mbp-202	0.0445211	1.14585	1.14585
Mbtps2	Mbtps2-006	0.0254106	0.137917	-7.25075
Mcf2l	Mcf2l-015	0.0371896	2.33817	2.33817
Mcfd2	Mcfd2-001	0.0230226	1.14415	1.14415
Mchr1	Mchr1-201	0.0201057	1.34597	1.34597
Mcm10	Mcm10-006	0.030542	0.000414889	-2410.28
Mcm3	Mcm3-005	0.0493308	3.92669e-07	-2.54667e+06
Mcm8	Mcm8-003	0.00718693	4.60097e-07	-2.17345e+06
Mcmbp	Mcmbp-002	0.0422421	1.48679	1.48679
Mctp1	Mctp1-201	0.0387454	2.06801	2.06801
Mcts1	Mcts1-005	0.0371723	1.71754	1.71754
Mdc1	Mdc1-001	0.0205897	1.15525	1.15525
Mdfic	Mdfic-001	0.0214208	2.35785	2.35785
Mdfic	Mdfic-006	0.0214208	2.35785	2.35785
Mdn1	Mdn1-007	0.0358846	4.55108e-11	-2.19728e+10
Mdp1	Mdp1-201	0.0391548	0.896484	-1.11547
Meaf6	Meaf6-004	0.0441508	0.477421	-2.09459
Meaf6	Meaf6-002	0.0344036	3.22427	3.22427
Mecr	Mecr-001	0.0144278	1.41497	1.41497
Mecr	Mecr-010	0.0157882	11.6969	11.6969
Med1	Med1-001	0.0133024	0.885087	-1.12983
Med14	Med14-001	0.0391969	0.848193	-1.17898
Med20	Med20-001	0.0313532	1.18115	1.18115
Med28	Med28-005	0.0249434	0.524152	-1.90785
Med8	Med8-007	0.0451759	2.69042	2.69042
Medag	Medag-003	0.00708916	0.236868	-4.22175

Medag	Medag-001	0.0103822	1.66556	1.66556
Mef2c	Mef2c-008	0.0448797	2.82692	2.82692
Megf9	Megf9-001	0.0299959	0.906939	-1.10261
Meis2	Meis2-006	0.0383967	1.80836	1.80836
Men1	Men1-006	0.0026465	0.432439	-2.31246
Men1	Men1-011	0.0419338	0.00224656	-445.125
Mesdc1	Mesdc1-001	0.00325785	1.25064	1.25064
Metap1	Metap1-006	0.00358756	9.58095	9.58095
Mettl13	Mettl13-006	0.041909	12.2836	12.2836
Mettl18	Mettl18-002	0.0390265	2.20834	2.20834
Mettl23	Mettl23-004	0.0066768	0.299538	-3.33847
Mettl23	Mettl23-001	0.0468653	1.41787	1.41787
Mettl25	Mettl25-002	0.000370194	0.110017	-9.0895
Mettl8	Mettl8-010	0.0430984	0.126503	-7.90494
Mfap1a	Mfap1a-001	0.037359	1.06657	1.06657
Mfsd4	Mfsd4-003	0.0037789	0.785179	-1.27359
Mfsd4	Mfsd4-011	0.0419742	247.139	247.139
Mfsd6	Mfsd6-002	0.0263319	0.668348	-1.49623
Mgat1	Mgat1-012	0.0431025	1.5089	1.5089
Mgat1	Mgat1-004	0.0144365	3.26201	3.26201
Mgat4b	Mgat4b-005	0.000908499	2.50178	2.50178
Mgat4b	Mgat4b-003	0.0258961	2.29114	2.29114
Mgat4c	Mgat4c-001	0.0291806	0.43511	-2.29827
Mgll	Mgll-201	0.0184253	0.657374	-1.5212
Mical3	Mical3-015	0.0194413	2.35705	2.35705
Mical3	Mical3-009	0.0423296	1.14149	1.14149
Mical3	Mical3-001	0.0412891	1.28767	1.28767
Micall1	Micall1-201	0.0369525	1.13687	1.13687
Mief1	Mief1-201	0.0314321	0.9109	-1.09782
Mina	Mina-003	0.0465975	46.5418	46.5418
Mink1	Mink1-013	0.0238435	0.785714	-1.27273
Mir421	Mir421-201	0.0369308	0.136554	-7.32311
Mir8114	Mir8114-201	0.0492157	0.221911	-4.50632
Misp	Misp-202	0.0486128	2.01957	2.01957
Misp	Misp-201	0.048613	2.01957	2.01957
Mitf	Mitf-002	0.00917556	5.54971	5.54971
Mitf	Mitf-001	0.0165585	1.33286	1.33286
Mknk1	Mknk1-009	0.0405321	2.07554	2.07554
Mllt10	Mllt10-001	0.00194209	0.125356	-7.97729
Mllt11	Mllt11-001	0.014888	0.925579	-1.0804
Mllt3	Mllt3-001	0.0166647	0.818378	-1.22193
Mlxipl	Mlxipl-005	0.02435	11.0236	11.0236
Mmd	Mmd-001	0.00735873	0.781437	-1.27969

Mmd2	Mmd2-002	0.0247126	0.171659	-5.82549
Mmp11	Mmp11-003	0.0417422	5.11518e-06	-195497
Mmp15	Mmp15-001	0.00337057	1.37659	1.37659
Mmp2	Mmp2-201	0.0124469	1.89141	1.89141
Mmp21	Mmp21-003	0.0232184	1.37094e-08	-7.29427e+07
Mmp23	Mmp23-004	0.0279938	4.95973	4.95973
Mms19	Mms19-004	0.00629291	1.73805	1.73805
Mn1	Mn1-001	0.0210846	1.34908	1.34908
Mobp	Mobp-001	0.0084739	1.23127	1.23127
Mobp	Mobp-201	0.00557624	1.85983	1.85983
Mocs1	Mocs1-003	0.0305859	0.627911	-1.59258
Mocs1	Mocs1-007	0.00180357	3.01415	3.01415
Mocs1	Mocs1-008	0.00753027	539.527	539.527
Morc2a	Morc2a-005	0.00991689	0.489216	-2.04409
Morn1	Morn1-003	0.0395339	1.5868	1.5868
Morn5	Morn5-001	0.0367168	1.85584	1.85584
Mospd2	Mospd2-007	0.0434085	1.09046e-10	-9.17044e+09
Mospd3	Mospd3-006	0.00881561	0.000885067	-1129.86
Mpc2	Mpc2-003	0.0294684	0.617388	-1.61973
Mpdz	Mpdz-014	0.0415264	0.034695	-28.8226
Mpp3	Mpp3-001	0.0211341	0.88904	-1.12481
Mpp4	Mpp4-007	0.0413658	5.35281	5.35281
Mpp6	Mpp6-002	0.0139309	1.58983	1.58983
Mpp7	Mpp7-201	0.00534615	1.92627	1.92627
Mpv17	Mpv17-006	0.0319242	37.6839	37.6839
Mpv17l	Mpv17l-002	0.0211264	0.751537	-1.33061
Mr1	Mr1-003	0.000628628	5.09943	5.09943
Mras	Mras-004	0.00939044	0.00256034	-390.573
Mre11a	Mre11a-001	0.031149	1.84374	1.84374
Mrgbp	Mrgbp-005	0.0363749	1.42441	1.42441
Mroh7	Mroh7-003	0.0311963	1.59841	1.59841
Mrpl12	Mrpl12-002	0.0381054	1.44698	1.44698
Mrpl15	Mrpl15-007	0.0110789	0.518803	-1.92751
Mrpl15	Mrpl15-001	0.0273088	1.36506	1.36506
Mrpl27	Mrpl27-004	0.0108955	0.0103336	-96.772
Mrpl27	Mrpl27-003	0.00483731	40.2073	40.2073
Mrpl3	Mrpl3-001	0.0210356	0.846932	-1.18073
Mrpl30	Mrpl30-007	0.0108685	0.633222	-1.57923
Mrpl33	Mrpl33-003	0.0455613	0.0428761	-23.323
Mrpl48	Mrpl48-005	0.0298914	2.9388	2.9388
Mrpl55	Mrpl55-004	0.0139406	0.338717	-2.95231
Mrpl55	Mrpl55-001	0.0355131	2.66185	2.66185
Mrps14	Mrps14-001	0.028082	0.821164	-1.21778

Mrps17	Mrps17-003	0.0435529	0.356129	-2.80797
Mrps18c	Mrps18c-005	0.00380196	0.138647	-7.21256
Mrps18c	Mrps18c-002	0.00450669	0.235706	-4.24257
Mrps21	Mrps21-004	0.0288378	0.32	-3.125
Mrps23	Mrps23-002	0.0112503	0.305079	-3.27784
Mrps23	Mrps23-001	0.0111455	1.39085	1.39085
Mrps23	Mrps23-003	0.0345933	807959	807959
Mrps24	Mrps24-004	0.0350395	0.00194018	-515.415
Mrps35	Mrps35-003	0.0295624	0.482152	-2.07404
Msc	Msc-001	0.0396874	0.397422	-2.51622
Msh2	Msh2-001	0.0357597	0.870533	-1.14872
Msl1	Msl1-006	0.0287214	9.49359	9.49359
Msl2	Msl2-002	0.0393849	0.806486	-1.23995
Msrb2	Msrb2-002	0.0318939	1.03336e-07	-9.67715e+06
Mst1r	Mst1r-001	0.0498152	0.340573	-2.93623
Mta1	Mta1-001	0.0434715	0.579042	-1.72699
Mta2	Mta2-001	0.0240788	1.10783	1.10783
Mtch2	Mtch2-002	0.00737103	0.755804	-1.32309
Mtcl1	Mtcl1-011	0.0209052	1.66631	1.66631
Mtf1	Mtf1-201	0.017072	1.11136	1.11136
Mthfr	Mthfr-001	0.014422	1.23355	1.23355
Mthfr	Mthfr-006	0.00901952	7.20014	7.20014
Mthfsd	Mthfsd-005	0.028242	0.225022	-4.44401
Mthfsd	Mthfsd-011	0.0446285	11.8327	11.8327
Mthfsd	Mthfsd-010	0.0122879	1.71979	1.71979
Mthfsd	Mthfsd-003	0.039083	2.20505	2.20505
Mtm1	Mtm1-007	0.0405107	1.72312	1.72312
Mtmr11	Mtmr11-005	0.0181678	0.137711	-7.26159
Mtmr14	Mtmr14-002	0.00746228	11.8567	11.8567
Mtrf1l	Mtrf1l-001	0.00847816	1.25371	1.25371
Mturn	Mturn-001	0.0392181	0.893492	-1.1192
Muc19	Muc19-201	0.0168556	8.6096	8.6096
Mum1	Mum1-003	0.0143256	1.36416	1.36416
Mum1l1	Mum1l1-202	0.0117975	2.45588	2.45588
Mus81	Mus81-011	0.0111361	3716.47	3716.47
Mxd4	Mxd4-003	0.00729145	1.28072	1.28072
Mxd4	Mxd4-001	0.0260845	1.16963	1.16963
Mxra7	Mxra7-001	0.0290628	1.25292	1.25292
Mxra8	Mxra8-004	0.0472118	5.54135e-08	-1.80462e+07
Mxra8	Mxra8-003	0.00111075	2.78975e+09	2.78975e+09
Mxra8	Mxra8-005	0.010749	4.0952	4.0952
Myc	Myc-001	0.0225587	0.408375	-2.44873
Myc	Myc-005	0.0225587	0.408375	-2.44873

Myc	Myc-202	0.0404886	18.1017	18.1017
Mycbp	Mycbp-001	0.0390515	1.4149	1.4149
Mycbp2	Mycbp2-002	0.041147	0.837062	-1.19466
Mycn	Mycn-001	0.0219072	0.544295	-1.83724
Myh10	Myh10-001	0.0216797	1.12301	1.12301
Myh7b	Myh7b-002	0.0491984	0.609376	-1.64102
Myl3	Myl3-005	0.0321906	356918	356918
Myl6	Myl6-201	0.0241273	1.11918	1.11918
Mylip	Mylip-201	0.0457412	0.872501	-1.14613
Mynn	Mynn-005	0.0466375	3.36205	3.36205
Myo1c	Myo1c-001	0.0386947	1.38742	1.38742
Myo1e	Myo1e-201	0.0423752	1.22156	1.22156
Myo5b	Myo5b-009	0.00406141	0.711347	-1.40578
Myo9a	Myo9a-001	0.00364435	0.736125	-1.35847
Myo9a	Myo9a-003	0.0386813	1.2161	1.2161
Myrf	Myrf-004	0.00627093	4.1321	4.1321
Myrip	Myrip-001	0.039769	1.14322	1.14322
Mzf1	Mzf1-006	0.0192473	0.804164	-1.24353
Mzf1	Mzf1-003	0.0480188	113.534	113.534
Mzt2	Mzt2-003	0.0277606	0.159412	-6.27307
n-R5s8	n-R5s8-201	0.0492586	0.232893	-4.29382
N4bp1	N4bp1-201	0.0182919	1.10937	1.10937
N4bp2	N4bp2-007	0.02345	6.97177	6.97177
N4bp3	N4bp3-001	0.00874805	1.38057	1.38057
Naa15	Naa15-002	0.0481598	0.630735	-1.58545
Naa15	Naa15-001	0.038497	0.654485	-1.52792
Naa35	Naa35-014	0.0486096	37.0365	37.0365
Naaladl2	Naaladl2-002	0.0136675	1.63264	1.63264
Nab1	Nab1-002	0.0414409	220.469	220.469
Nabp1	Nabp1-006	0.00964022	0.579192	-1.72654
Nadk	Nadk-001	0.0200711	0.676615	-1.47794
Nadk	Nadk-002	0.0483513	1.40691	1.40691
Nadk2	Nadk2-002	0.00272417	0.770504	-1.29785
Nadk2	Nadk2-201	0.00588576	1.27825	1.27825
Nadk2	Nadk2-001	0.0047872	1.28748	1.28748
Nagk	Nagk-008	0.0294504	0.21696	-4.60915
Naip6	Naip6-201	0.0118079	2.14657	2.14657
Nanos1	Nanos1-201	0.0348934	1.12186	1.12186
Napb	Napb-001	0.0121067	0.894351	-1.11813
Napg	Napg-001	0.048058	0.923429	-1.08292
Narfl	Narfl-002	0.0383465	1.52993	1.52993
Nav3	Nav3-001	0.0432317	0.778884	-1.28389
Nbea	Nbea-008	0.0445036	0.509403	-1.96308

Nbeal1	Nbeal1-006	0.00881933	0.117975	-8.47636
Nbr1	Nbr1-012	0.0492178	1.80685	1.80685
Ncam1	Ncam1-001	0.0377618	0.910544	-1.09825
Ncapd2	Ncapd2-006	0.0180957	0.318913	-3.13565
Nckap1	Nckap1-003	0.024713	0.641999	-1.55764
Ncmap	Ncmap-201	0.0103956	3.55563	3.55563
Ncoa1	Ncoa1-201	0.0234468	0.916165	-1.09151
Ncoa3	Ncoa3-201	0.045968	1.23413	1.23413
Ncoa4	Ncoa4-004	0.0369858	0.662443	-1.50956
Ncoa7	Ncoa7-201	0.014441	0.940052	-1.06377
Ncor1	Ncor1-006	0.0009529	0.594374	-1.68244
Ncor1	Ncor1-015	0.0465846	0.241565	-4.13968
Ncor2	Ncor2-001	0.029421	1.25918	1.25918
Ndc1	Ndc1-002	0.0468465	0.625476	-1.59878
Ndfip2	Ndfip2-003	0.0391776	0.835826	-1.19642
Ndnl2	Ndnl2-001	0.00980997	0.817387	-1.22341
Ndrg3	Ndrg3-006	0.00703182	2.24314e-10	-4.45804e+09
Ndrg4	Ndrg4-007	0.0269663	1.60984	1.60984
Ndrg4	Ndrg4-010	0.0276112	1.19073	1.19073
Ndufaf4	Ndufaf4-002	0.0093097	0.848027	-1.17921
Ndufaf4	Ndufaf4-003	0.0242284	1.5358	1.5358
Ndufaf7	Ndufaf7-201	0.0436983	1.12012	1.12012
Ndufb10	Ndufb10-005	0.0152728	2.69328	2.69328
Ndufb2	Ndufb2-001	0.0203835	1.20607	1.20607
Ndufs7	Ndufs7-004	0.0147344	2.52478	2.52478
Ndufv2	Ndufv2-004	0.0112666	0.205957	-4.85539
Necab3	Necab3-001	0.0399827	1.26707	1.26707
Necap2	Necap2-005	0.00480079	4.62627	4.62627
Nedd4	Nedd4-003	0.0195446	0.16635	-6.01144
Nedd4	Nedd4-008	0.0449793	3.08806	3.08806
Nedd4l	Nedd4l-201	0.0378773	1.12747	1.12747
Nek1	Nek1-002	0.027073	0.690482	-1.44826
Nek1	Nek1-001	0.0459058	1.10907	1.10907
Nek10	Nek10-001	0.0308182	2.05596	2.05596
Nek5	Nek5-201	0.0416823	3.97033	3.97033
Nelfe	Nelfe-005	0.0469516	2.8063	2.8063
Neto1	Neto1-201	0.0408382	0.898104	-1.11346
Neurl3	Neurl3-001	0.0172812	1.57393	1.57393
Neurl4	Neurl4-008	0.0382443	0.666914	-1.49944
Neurl4	Neurl4-007	0.0359943	0.67731	-1.47643
Neurod6	Neurod6-001	0.0452685	0.834687	-1.19805
Nexn	Nexn-004	0.00563592	9.84617	9.84617
Nf1	Nf1-007	0.00672056	2.06816	2.06816

Nfat5	Nfat5-009	0.0417199	0.414554	-2.41223
Nfatc2ip	Nfatc2ip-001	0.019978	0.785711	-1.27273
Nfe2l1	Nfe2l1-003	0.0218237	11.3555	11.3555
Nfia	Nfia-002	0.0201672	0.66827	-1.4964
Nfkb1	Nfkb1-005	0.03703	0.242938	-4.11628
Nfkb1	Nfkb1-007	0.047384	0.426316	-2.34568
Nfkbid	Nfkbid-001	0.0245452	24.7833	24.7833
Nfkbiz	Nfkbiz-003	0.017083	0.320689	-3.11829
Nfxl1	Nfxl1-005	0.0306627	0.000110341	-9062.84
Nfya	Nfya-010	0.0372081	1.61361	1.61361
Nfya	Nfya-005	0.00586136	5.39426	5.39426
Ngef	Ngef-002	0.0481967	1.40237	1.40237
Nhlh1	Nhlh1-001	0.029395	0.691287	-1.44658
Ninj1	Ninj1-001	0.0382237	1.3921	1.3921
Ninj2	Ninj2-002	0.0457922	542.917	542.917
Nipal2	Nipal2-201	0.0179714	1.41362	1.41362
Nipsnap1	Nipsnap1-002	0.0219	0.621963	-1.60781
Nisch	Nisch-001	0.0044381	0.882306	-1.13339
Nkiras2	Nkiras2-004	0.00312912	0.256817	-3.89382
Nle1	Nle1-001	0.00392869	0.791452	-1.2635
Nle1	Nle1-006	0.0297264	5.05761	5.05761
Nle1	Nle1-002	0.00563461	5.54132	5.54132
Nlgn1	Nlgn1-201	0.014001	0.649569	-1.53948
Nlgn3	Nlgn3-002	0.0408776	0.780603	-1.28106
Nmbr	Nmbr-001	0.00474351	1.56613	1.56613
Nme3	Nme3-003	0.0239453	26.13	26.13
Nmnat1	Nmnat1-001	0.016354	1.99141	1.99141
Nnt	Nnt-001	0.00312272	2.25105	2.25105
Nog	Nog-001	0.00447788	0.683591	-1.46286
Nol4	Nol4-001	0.0391884	0.829033	-1.20622
Nop58	Nop58-001	0.0133453	0.874497	-1.14351
Nop58	Nop58-005	0.0321629	0.273771	-3.65269
Nop58	Nop58-002	0.0031576	1.15648	1.15648
Nos1	Nos1-001	0.0044678	0.805114	-1.24206
Nos1ap	Nos1ap-005	0.0135229	0.750028	-1.33328
Nostrin	Nostrin-001	0.0349693	1.64354	1.64354
Notch1	Notch1-011	0.0267147	0.169989	-5.88274
Notch4	Notch4-001	0.0390825	1.42591	1.42591
Noxo1	Noxo1-003	0.0381818	53.7172	53.7172
Npas1	Npas1-201	0.0252314	0.801475	-1.2477
Npepps	Npepps-009	0.0424867	5.91913	5.91913
Nploc4	Nploc4-004	0.0484279	2.32138	2.32138
Nprl2	Nprl2-004	0.0453709	0.248682	-4.0212

Nprl3	Nprl3-015	0.0483053	0.666122	-1.50123
Nr1h2	Nr1h2-011	0.0476184	0.12587	-7.94473
Nr1h2	Nr1h2-006	0.0366899	1.44147	1.44147
Nr1h3	Nr1h3-008	0.0416033	0.000451993	-2212.42
Nr1i2	Nr1i2-001	0.000120962	7.26552	7.26552
Nr2f2	Nr2f2-001	0.0496995	0.638883	-1.56523
Nr2f6	Nr2f6-007	0.0486185	157.491	157.491
Nrf1	Nrf1-204	0.0248207	0.0259592	-38.522
Nrg4	Nrg4-006	0.04887	0.357273	-2.79898
Nrip3	Nrip3-001	0.0302695	0.777209	-1.28666
Nrip3	Nrip3-002	0.010247	0.821288	-1.2176
Nrxn1	Nrxn1-021	0.02263	3.36216	3.36216
Nrxn2	Nrxn2-006	0.0106304	1.92154	1.92154
Nrxn3	Nrxn3-002	0.0177295	0.399294	-2.50442
Nsf	Nsf-001	0.00241369	0.8864	-1.12816
Nsf	Nsf-005	0.00885064	3.38842	3.38842
Nsun5	Nsun5-008	0.00501327	0.621241	-1.60968
Nsun6	Nsun6-001	0.0169909	0.677156	-1.47677
Nsun6	Nsun6-003	0.000838753	16.1528	16.1528
Nt5c3	Nt5c3-006	0.0477578	0.0977922	-10.2258
Nt5e	Nt5e-002	0.0463166	1.63436	1.63436
Ntpcr	Ntpcr-002	0.0292215	1.46044	1.46044
Ntrk2	Ntrk2-202	0.00583065	1.11359	1.11359
Ntrk3	Ntrk3-001	0.0128734	0.877128	-1.14009
Nubp2	Nubp2-201	0.0347721	0.893121	-1.11967
Nucb1	Nucb1-006	0.0334419	8.1757e-05	-12231.4
Nudc	Nudc-002	0.0320194	9.39409e-06	-106450
Nudc	Nudc-001	0.0270629	1.07341	1.07341
Nudt17	Nudt17-003	0.0398773	0.489273	-2.04385
Nufip1	Nufip1-201	0.0228371	0.894816	-1.11755
Numb	Numb-004	0.026492	2.46636	2.46636
Nup160	Nup160-006	0.0272894	16.2935	16.2935
Nup85	Nup85-007	0.0256276	0.366181	-2.73089
Nup88	Nup88-004	0.0334411	0.474869	-2.10585
Nup88	Nup88-002	0.0431308	1.24413	1.24413
Nupl2	Nupl2-004	0.00923585	0.184806	-5.41108
Nupr1l	Nupr1l-004	0.00137431	7.04497e-08	-1.41945e+07
Nxpe3	Nxpe3-201	0.00854286	0.868686	-1.15116
Nxt2	Nxt2-001	0.0263222	0.813853	-1.22872
Oaz2	Oaz2-001	0.0226272	1.13538	1.13538
Obscn	Obscn-202	0.0246299	2.91559	2.91559
Obsl1	Obsl1-014	0.032915	0.000751414	-1330.82
Odf2	Odf2-017	0.014178	1.43192e-10	-6.98364e+09

Odf2	Odf2-019	0.041281	0.0393605	-25.4062
Odf2l	Odf2l-013	0.00470602	53.6778	53.6778
Ogdh	Ogdh-006	0.00734388	6.29137	6.29137
Ogfrl1	Ogfrl1-001	0.0443777	0.892469	-1.12049
Ogfrl1	Ogfrl1-003	0.0206073	0.306262	-3.26517
Olfr287	Olfr287-004	0.00496573	2.41372e-05	-41429.9
Olfr61	Olfr61-201	0.0400241	0.368438	-2.71416
Olfr658	Olfr658-001	0.0327686	7.13524	7.13524
Olig2	Olig2-001	0.0335653	0.8617	-1.1605
Opcml	Opcml-001	0.0375161	0.888717	-1.12522
Oplah	Oplah-001	0.00393299	1.71234	1.71234
Opn3	Opn3-001	0.0182127	1.86993	1.86993
Oprk1	Oprk1-002	0.0379522	42754.8	42754.8
Orc2	Orc2-004	0.0356702	0.271057	-3.68926
Orc3	Orc3-011	0.0278983	0.777134	-1.28678
Osbpl3	Osbpl3-011	0.0372731	0.00933904	-107.077
Osbpl9	Osbpl9-006	0.0346281	7.30075e+10	7.30075e+10
Osgep	Osgep-001	0.0440269	0.747679	-1.33747
Osgepl1	Osgepl1-001	0.0181044	0.489177	-2.04425
Otop1	Otop1-201	0.0114392	2.36017e-09	-4.23698e+08
Otop1	Otop1-202	0.0114392	2.36017e-09	-4.23698e+08
Otop1	Otop1-001	0.0114392	2.36017e-09	-4.23698e+08
Otud4	Otud4-001	0.0434553	1.34349	1.34349
Otud5	Otud5-004	0.0356893	1.3107	1.3107
Otud7b	Otud7b-001	0.00471122	1.28059	1.28059
Otx2	Otx2-001	0.0450489	8.27525	8.27525
Oxsm	Oxsm-001	0.0113002	0.733979	-1.36244
P2rx7	P2rx7-008	0.0064713	0.00510562	-195.862
P2ry1	P2ry1-003	0.0272818	5.67094	5.67094
P3h1	P3h1-007	0.0136576	2.32954	2.32954
P4ha1	P4ha1-003	0.0217992	1.35972	1.35972
P4htm	P4htm-003	0.0292147	0.723145	-1.38285
Pabpc4l	Pabpc4l-002	0.0448953	132.143	132.143
Pabpc5	Pabpc5-001	0.0104597	0.695866	-1.43706
Pacsin1	Pacsin1-002	0.0236048	0.718087	-1.39259
Pafah1b1	Pafah1b1-002	0.0071485	0.89827	-1.11325
Pafah1b1	Pafah1b1-005	0.0033208	2.57263	2.57263
Pafah1b3	Pafah1b3-007	0.00880915	2.16303	2.16303
Pafah2	Pafah2-002	0.000714951	16.2015	16.2015
Pak3	Pak3-201	0.000672292	0.730399	-1.36911
Pak3	Pak3-005	0.000672196	0.730409	-1.3691
Pak3	Pak3-002	0.0148329	0.639157	-1.56456
Pak3	Pak3-008	0.022263	1.31527	1.31527

Pald1	Pald1-201	0.00690152	0.846823	-1.18088
Palld	Palld-004	0.00102822	0.277752	-3.60033
Palm	Palm-201	0.0203852	0.742507	-1.34679
Palm	Palm-202	0.0340718	1.09965	1.09965
Palm2	Palm2-004	0.00076058	0.557012	-1.79529
Palmd	Palmd-003	0.0336107	5.63037	5.63037
Panct2	Panct2-001	0.0313947	0.791839	-1.26288
Pank2	Pank2-007	0.0202008	3.6034	3.6034
Papd5	Papd5-201	0.0122395	1.44679	1.44679
Papd5	Papd5-003	0.0377196	2.49962	2.49962
Pard3	Pard3-014	0.000205247	3.08881	3.08881
Pard3b	Pard3b-002	0.0416161	16.1599	16.1599
Pard6b	Pard6b-001	0.00202545	1.70775	1.70775
Park2	Park2-201	0.0412851	6.23776e-09	-1.60314e+08
Parl	Parl-008	0.00429928	1.72003	1.72003
Parp11	Parp11-004	0.0384914	122.296	122.296
Parp4	Parp4-001	0.0349961	1.3528	1.3528
Parvg	Parvg-005	0.0306248	5.31996e-08	-1.87971e+07
Pax6	Pax6-011	0.0351103	0.270968	-3.69048
Pax6	Pax6-026	0.0338408	12.608	12.608
Pbrm1	Pbrm1-015	0.033009	0.347068	-2.88128
Pbrm1	Pbrm1-011	0.0283885	1.88695	1.88695
Pcbp3	Pcbp3-201	0.047688	1.61527	1.61527
Pcbp4	Pcbp4-005	0.00326006	0.18297	-5.46538
Pcbp4	Pcbp4-012	0.000471347	2.41154	2.41154
Pcbp4	Pcbp4-008	0.0405964	2.1538	2.1538
Pcdh15	Pcdh15-033	0.0461128	2.97094e-07	-3.36594e+06
Pcdh15	Pcdh15-037	0.0211486	0.0708115	-14.122
Pcdh15	Pcdh15-039	0.0456425	0.00132224	-756.294
Pcdha11	Pcdha11-005	0.0138928	42.7098	42.7098
Pcdhb17	Pcdhb17-001	0.0395131	0.910012	-1.09889
Pced1a	Pced1a-006	0.0168351	0.648647	-1.54167
Pced1a	Pced1a-005	0.0134187	1.40511	1.40511
Pcgf1	Pcgf1-201	0.00847006	0.214882	-4.65372
Pcgf2	Pcgf2-003	0.0155434	3.07648	3.07648
Pcgf3	Pcgf3-001	0.0065673	1.14056	1.14056
Pcnp	Pcnp-006	0.0440091	3.29841e-05	-30317.6
Pcnp	Pcnp-003	0.0214085	2.81966	2.81966
Pcp2	Pcp2-005	0.0152872	17.0023	17.0023
Pcp4	Pcp4-001	0.0399686	1.56691	1.56691
Pcp4l1	Pcp4l1-001	0.0336301	2.0821	2.0821
Pcsk7	Pcsk7-201	0.00904295	1.13889	1.13889
Pcyox1	Pcyox1-002	0.0086957	0.0402334	-24.855

Pcyt1a	Pcyt1a-002	0.0428057	1.89818	1.89818
Pdcl3	Pdcl3-001	0.0488016	1.36206	1.36206
Pdcl3	Pdcl3-004	0.00111878	1.29445	1.29445
Pde10a	Pde10a-005	0.0320112	17.4851	17.4851
Pde11a	Pde11a-002	0.0396545	0.199066	-5.02345
Pde1a	Pde1a-009	0.00318627	0.634478	-1.5761
Pde1a	Pde1a-002	0.0168338	0.645083	-1.55019
Pde3b	Pde3b-002	0.0166778	183129	183129
Pde4b	Pde4b-012	0.0180944	2.09436	2.09436
Pde8b	Pde8b-001	0.0372056	0.721443	-1.38611
Pdk4	Pdk4-001	0.00503333	1.22889	1.22889
Pdlim4	Pdlim4-001	0.0112684	1.55455	1.55455
Pdlim4	Pdlim4-002	0.00129037	2.94672	2.94672
Pdp1	Pdp1-001	0.0239185	1.31404	1.31404
Pdp1	Pdp1-002	0.0411943	1.48999	1.48999
Pdp2	Pdp2-201	0.0148672	1.18284	1.18284
Pdrg1	Pdrg1-005	0.0419452	34.7929	34.7929
Pds5a	Pds5a-006	0.0487927	1.45559	1.45559
Pds5b	Pds5b-006	0.0264543	1.58917	1.58917
Pdss2	Pdss2-001	0.0151748	0.859008	-1.16413
Pdss2	Pdss2-005	0.0378193	6.51752	6.51752
Pdxk	Pdxk-001	0.0375348	1.14246	1.14246
Pdzd11	Pdzd11-003	0.0162467	1.72864	1.72864
Pdzd2	Pdzd2-004	0.0178806	1.32242	1.32242
Pdzd2	Pdzd2-011	0.0345263	6.06307	6.06307
Peak1os	Peak1os-001	0.0142957	0.456133	-2.19234
Peli1	Peli1-001	0.0300292	0.926559	-1.07926
Pelp1	Pelp1-002	0.0241174	1.83449e-08	-5.45109e+07
Pelp1	Pelp1-001	0.0245531	1.06912	1.06912
Per2	Per2-001	0.0124539	1.34389	1.34389
Pet117	Pet117-001	3.90527e-05	59.5237	59.5237
Pex1	Pex1-003	0.00849746	2.26337	2.26337
Pex1	Pex1-009	0.0212184	1.49537	1.49537
Pex11b	Pex11b-006	0.0351663	0.486318	-2.05627
Pex11b	Pex11b-005	0.0342858	3.57117	3.57117
Pex12	Pex12-002	0.0240956	1.73929	1.73929
Pex5	Pex5-002	0.0465793	1.59818	1.59818
Pex5l	Pex5l-005	0.0141152	1.71195	1.71195
Pex7	Pex7-201	0.0250429	0.327653	-3.05201
Pfas	Pfas-003	0.0285332	912.38	912.38
Pfkfb2	Pfkfb2-005	0.00832539	0.702565	-1.42336
Pfkfb3	Pfkfb3-003	0.0387001	0.387443	-2.58102
Pfkfb3	Pfkfb3-002	0.0391185	1.65164	1.65164

Pfkp	Pfkp-013	0.0494996	16.5914	16.5914
Pgap2	Pgap2-024	0.00728267	12.8728	12.8728
Pgap3	Pgap3-003	0.00183894	0.25256	-3.95946
Pgbd1	Pgbd1-003	0.0416508	8825.05	8825.05
Pgbd5	Pgbd5-004	0.0158469	0.148671	-6.72625
Phactr1	Phactr1-010	0.0225659	6.62923e-10	-1.50847e+09
Phactr1	Phactr1-005	0.0221786	0.507822	-1.96919
Phactr1	Phactr1-004	0.0432608	1.7087	1.7087
Phc1	Phc1-013	0.00979896	1.4684e-11	-6.81014e+10
Phc2	Phc2-003	0.000106486	7.46128	7.46128
Phf10	Phf10-005	0.035864	2.99982	2.99982
Phf14	Phf14-005	0.017648	0.0098524	-101.498
Phf20	Phf20-007	0.0309254	4.79454	4.79454
Phf21a	Phf21a-001	0.00894344	2.34561	2.34561
Phf21b	Phf21b-003	0.0333714	3.92988	3.92988
Phf24	Phf24-007	0.0439279	1.53627	1.53627
Phip	Phip-011	0.00363305	3.67224	3.67224
Phka1	Phka1-006	0.0259058	0.128254	-7.79702
Phka1	Phka1-001	0.0239592	0.746064	-1.34037
Phkg1	Phkg1-005	0.0491903	0.610614	-1.6377
Phlda1	Phlda1-002	0.026519	2.47599	2.47599
Phospho1	Phospho1-003	0.0387516	0.0745546	-13.413
Phrf1	Phrf1-008	0.0483234	0.0171675	-58.2497
Phtf1	Phtf1-001	0.0448569	0.350161	-2.85583
Phtf1	Phtf1-005	0.0469456	1.29833	1.29833
Phtf1	Phtf1-004	0.0404187	1.38932	1.38932
Phyhd1	Phyhd1-008	0.0128471	2.49557	2.49557
Pi16	Pi16-001	0.0319939	0.216766	-4.61326
Pi4ka	Pi4ka-004	0.013048	0.00287079	-348.336
Pi4ka	Pi4ka-010	0.0209063	1.49103	1.49103
Pid1	Pid1-001	0.0215922	1.19461	1.19461
Piezo1	Piezo1-007	0.00546597	10.9694	10.9694
Pigf	Pigf-001	0.00386722	0.795799	-1.2566
Pigk	Pigk-006	0.0250802	0.744839	-1.34257
Pigq	Pigq-013	0.00144717	0.601549	-1.66237
Pigr	Pigr-001	0.0406567	0.231062	-4.32784
Pigt	Pigt-002	0.00635728	1.94313	1.94313
Pigu	Pigu-002	0.00214218	0.290161	-3.44636
Pigu	Pigu-201	0.009797	2.23486	2.23486
Pigu	Pigu-003	0.0375688	1.46001	1.46001
Pigx	Pigx-004	0.0146561	0.0263647	-37.9296
Pik3c2b	Pik3c2b-001	0.0476559	1.22915	1.22915
Pik3cb	Pik3cb-001	0.0130385	0.714857	-1.39888

Pik3cd	Pik3cd-001	0.0347135	0.46459	-2.15244
Pik3ip1	Pik3ip1-004	0.0428922	0.188569	-5.30311
Pik3r1	Pik3r1-001	0.0134053	1.10316	1.10316
Pik3r4	Pik3r4-011	0.0147382	854.395	854.395
Pikfyve	Pikfyve-007	0.0398978	831.588	831.588
Pilra	Pilra-001	0.00634038	0.231902	-4.31216
Pim1	Pim1-001	0.01174	1.47388	1.47388
Pip4k2b	Pip4k2b-002	0.0420929	1.97924	1.97924
Pisd	Pisd-006	0.00983966	0.0656575	-15.2306
Pja1	Pja1-201	0.0194904	0.877602	-1.13947
Pja1	Pja1-002	0.00674934	1.73784	1.73784
Pja2	Pja2-002	0.0126683	0.884714	-1.13031
Pkdrej	Pkdrej-201	0.000413365	0.494702	-2.02142
Pkib	Pkib-003	0.0128101	2.6118e-07	-3.82878e+06
Pkib	Pkib-011	0.0327921	81.7573	81.7573
Pkmyt1	Pkmyt1-201	0.0492842	1.52847	1.52847
Pkn1	Pkn1-009	0.00876591	0.268036	-3.73085
Pkn2	Pkn2-008	0.0382152	0.00941907	-106.168
Pkn3	Pkn3-004	0.0455606	4.59111	4.59111
Pkn3	Pkn3-003	0.0469889	2766.03	2766.03
Pknox1	Pknox1-001	0.0363344	1.24774	1.24774
Pknox2	Pknox2-201	0.0124662	1.59056	1.59056
Pkp3	Pkp3-001	0.0335441	10.0656	10.0656
Pkp3	Pkp3-002	0.0335441	10.0656	10.0656
Pla2g16	Pla2g16-002	0.0366297	1.32757	1.32757
Pla2g16	Pla2g16-001	0.0339376	1.14739	1.14739
Pla2g4e	Pla2g4e-002	0.0293096	0.324329	-3.08329
Pla2g5	Pla2g5-004	0.032332	6.36513e-05	-15710.6
Pla2g6	Pla2g6-004	0.0269036	0.38717	-2.58284
Pla2g7	Pla2g7-001	0.0374619	0.904426	-1.10567
Platr14	Platr14-004	0.00652207	5.03749e-09	-1.98511e+08
Plcb4	Plcb4-003	0.0281029	1.41157	1.41157
Plch1	Plch1-005	0.0454726	0.275252	-3.63303
Plch1	Plch1-002	0.0139598	2.0969	2.0969
Plch2	Plch2-002	0.0431877	0.686042	-1.45764
Plcxd1	Plcxd1-003	0.0188945	2.24114	2.24114
Plek2	Plek2-001	0.0114129	3.78882	3.78882
Plekha1	Plekha1-007	0.00143434	3.30604e-12	-3.02476e+11
Plekha4	Plekha4-005	0.0377693	0.0770911	-12.9717
Plekha5	Plekha5-002	0.0219128	68.8502	68.8502
Plekhb1	Plekhb1-009	0.0422875	2.97444	2.97444
Plekhb2	Plekhb2-005	0.0299117	222.828	222.828
Plekhg3	Plekhg3-201	0.0264568	1.32679	1.32679

Plekhg4	Plekhg4-002	0.0316145	0.000138006	-7246.05
Plekhg5	Plekhg5-006	0.0425634	0.31989	-3.12608
Plekhs1	Plekhs1-201	0.0338581	2.21361	2.21361
Plin3	Plin3-201	0.00230479	1.23258	1.23258
Plod3	Plod3-001	0.0367851	1.082	1.082
Plod3	Plod3-003	0.0259858	2.12715	2.12715
Plp1	Plp1-003	0.0276845	1.25113	1.25113
Pls3	Pls3-005	0.0301322	0.8347	-1.19804
Plxna4	Plxna4-001	0.0354103	0.882056	-1.13371
Plxna4os1	Plxna4os1-001	0.000495854	0.0702944	-14.2259
Pmepa1	Pmepa1-001	0.0438158	1.27001	1.27001
Pmf1	Pmf1-004	0.00464277	0.000324998	-3076.94
Pmpca	Pmpca-004	0.0444146	0.417927	-2.39276
Pms2	Pms2-001	0.0462312	0.659507	-1.51628
Pnck	Pnck-004	0.0462202	0.705772	-1.41689
Pnisr	Pnisr-008	0.0335762	0.088146	-11.3448
Pnkp	Pnkp-003	0.0479179	0.63028	-1.5866
Pnkp	Pnkp-004	0.0333338	6.52775	6.52775
Poc1a	Poc1a-001	0.0396905	0.688576	-1.45227
Poglut1	Poglut1-003	0.0440797	0.110324	-9.0642
Pogz	Pogz-007	0.0110413	2.40883	2.40883
Pola1	Pola1-001	0.0202552	1.21093	1.21093
Pold1	Pold1-001	0.0137984	0.361605	-2.76545
Pold3	Pold3-008	0.0308386	1.54683e-06	-646485
Pold3	Pold3-001	0.0311578	1.14193	1.14193
Polg	Polg-016	0.0235665	12.6229	12.6229
Polq	Polq-004	0.0254839	0.19335	-5.17197
Polr1a	Polr1a-006	0.0046839	7.7871	7.7871
Polr1d	Polr1d-006	0.0282567	0.249288	-4.01142
Polr1e	Polr1e-002	0.0459611	0.470151	-2.12698
Polr2e	Polr2e-004	0.0443073	4.34996	4.34996
Polr3d	Polr3d-202	0.0402922	2.5732	2.5732
Pomgnt1	Pomgnt1-005	0.0176765	3.46874	3.46874
Pomgnt1	Pomgnt1-001	0.0351102	1.30123	1.30123
Pomk	Pomk-201	0.0390143	0.908494	-1.10072
Pomt1	Pomt1-009	0.00707776	2.96693	2.96693
Pon2	Pon2-001	0.00658315	1.2831	1.2831
Popdc2	Popdc2-003	0.010385	0.00636625	-157.078
Porcn	Porcn-003	0.0373585	0.876643	-1.14072
Postn	Postn-004	0.0272209	13.63	13.63
Pou2f1	Pou2f1-006	0.0277231	485.828	485.828
Pou3f2	Pou3f2-001	0.0264526	1.27396	1.27396
Pou4f1	Pou4f1-001	0.0229789	5.44446	5.44446

Ppap2a	Ppap2a-202	0.000127615	1.60413	1.60413
Ppara	Ppara-003	0.0181358	17.3392	17.3392
Ppard	Ppard-002	0.0324647	2.12064	2.12064
Ppdpf	Ppdpf-004	0.0284641	0.724538	-1.38019
Ppfia2	Ppfia2-201	0.0280161	0.848353	-1.17875
Ppie	Ppie-003	0.0321604	0.390297	-2.56215
Ppm1e	Ppm1e-001	0.0381578	0.878953	-1.13772
Ppp1r12c	Ppp1r12c-007	0.0362586	1.808	1.808
Ppp1r13l	Ppp1r13l-001	0.0459924	1.37946	1.37946
Ppp1r15b	Ppp1r15b-001	0.0397377	1.14363	1.14363
Ppp1r17	Ppp1r17-001	0.0107689	2.72734	2.72734
Ppp1r18	Ppp1r18-004	0.0374546	1.80192	1.80192
Ppp1r1b	Ppp1r1b-007	0.0117583	101.239	101.239
Ppp1r1b	Ppp1r1b-002	0.0238482	18.962	18.962
Ppp1r21	Ppp1r21-005	0.0433398	8.45562e-05	-11826.5
Ppp1r35	Ppp1r35-003	0.0286416	1.92954	1.92954
Ppp1r37	Ppp1r37-201	0.015832	1.09898	1.09898
Ppp1r3f	Ppp1r3f-004	0.0469537	2.40789	2.40789
Ppp1r9a	Ppp1r9a-003	0.0360446	0.266651	-3.75022
Ppp2r1b	Ppp2r1b-001	0.0192336	1.53183	1.53183
Ppp2r1b	Ppp2r1b-002	0.022868	1.16277	1.16277
Ppp2r3a	Ppp2r3a-001	0.0327719	0.884762	-1.13025
Ppp2r5e	Ppp2r5e-201	0.0498	0.940957	-1.06275
Ppp4r1	Ppp4r1-010	0.0128178	0.176881	-5.65352
Ppp4r1l-ps	Ppp4r1l-ps-004	0.00880422	720.016	720.016
Ppp4r2	Ppp4r2-003	0.00290032	1.67911	1.67911
Pprc1	Pprc1-004	0.0196111	1.49121	1.49121
Pqlc1	Pqlc1-008	0.0260917	0.430134	-2.32486
Pqlc1	Pqlc1-004	0.0218271	3.1255	3.1255
Pqlc3	Pqlc3-001	0.00123345	9.09	9.09
Prc1	Prc1-012	0.0246853	0.472472	-2.11653
Prc1	Prc1-005	0.0348504	0.139268	-7.18041
Prdx1	Prdx1-003	0.0288075	1.48379	1.48379
Prdx2	Prdx2-007	0.00201898	0.625711	-1.59818
Prdx2	Prdx2-201	0.0117683	1.23854	1.23854
Prdx4	Prdx4-001	0.0153786	1.28188	1.28188
Preb	Preb-007	0.0395212	1.96348	1.96348
Prex1	Prex1-008	0.00612443	4.47874	4.47874
Prg4	Prg4-201	0.00337855	0.0308713	-32.3926
Prickle3	Prickle3-002	0.00815503	2.9218	2.9218
Prim2	Prim2-002	0.0323363	7.79046	7.79046
Prkar1a	Prkar1a-001	0.0205224	0.922246	-1.08431
Prkar2b	Prkar2b-003	0.0262857	2.00902	2.00902

Prkcd	Prkcd-202	0.0464102	1.96776	1.96776
Prkd1	Prkd1-201	0.0437921	0.869117	-1.15059
Prkx	Prkx-001	0.000170808	1.31863	1.31863
Prlr	Prlr-009	0.0297146	9339.31	9339.31
Prlr	Prlr-008	0.0276969	4.74562	4.74562
Prmt2	Prmt2-003	0.0248323	1.72564	1.72564
Prmt5	Prmt5-003	0.0453797	0.128239	-7.79797
Prmt6	Prmt6-001	0.027584	0.692605	-1.44383
Prmt7	Prmt7-001	0.0384725	0.90578	-1.10402
Prnp	Prnp-001	0.0325203	0.918458	-1.08878
Proser3	Proser3-002	0.00973812	2.56987	2.56987
Prpf38b	Prpf38b-006	0.00311271	1.46228	1.46228
Prpf39	Prpf39-001	0.0144418	0.863362	-1.15826
Prpf40b	Prpf40b-015	0.0199412	0.6624	-1.50966
Prpf40b	Prpf40b-003	0.0441753	1.45666	1.45666
Prpsap2	Prpsap2-002	0.0359244	0.52456	-1.90636
Prr22	Prr22-002	0.0355559	1.22491e-08	-8.1639e+07
Prr3	Prr3-001	0.0172925	1.93483	1.93483
Prr36	Prr36-002	0.035711	0.298493	-3.35017
Prrc2c	Prrc2c-009	0.0365008	1.50387	1.50387
Prrg4	Prrg4-001	0.0352298	2.43907	2.43907
Prrx2	Prrx2-003	0.0394774	5.91433e-08	-1.69081e+07
Prss41	Prss41-002	0.0391986	0.411795	-2.42839
Prtg	Prtg-001	0.0488845	0.777113	-1.28681
Psd2	Psd2-005	0.0168367	1.44546	1.44546
Psd4	Psd4-009	0.000295378	8.94751	8.94751
Psen2	Psen2-001	0.0241032	1.52083	1.52083
Psma7	Psma7-005	0.0265311	0.692611	-1.44381
Psmb9	Psmb9-004	0.0149453	0.039709	-25.1832
Psmc3ip	Psmc3ip-004	0.0269567	0.355579	-2.81232
Psmd11	Psmd11-013	0.0488809	5.42983	5.42983
Psme1	Psme1-012	0.00605413	1.83971	1.83971
Psmf1	Psmf1-003	0.023126	1.42354	1.42354
Psmg4	Psmg4-002	0.0102675	2.97734	2.97734
Psors1c2	Psors1c2-001	0.0197288	0.157616	-6.34453
Pstpip2	Pstpip2-201	0.000700447	0.770363	-1.29809
Ptcd3	Ptcd3-001	0.00031314	0.854352	-1.17048
Ptch1	Ptch1-004	0.0326159	0.000123784	-8078.57
Ptch1	Ptch1-005	0.0068255	1.21934	1.21934
Pter	Pter-001	0.00954165	0.834524	-1.19829
Ptges3	Ptges3-002	0.0472776	0.704512	-1.41942
Ptgfrn	Ptgfrn-001	0.00499435	1.47969	1.47969
Ptgs2	Ptgs2-001	0.025837	0.719628	-1.38961

Pth1r	Pth1r-201	0.0403898	0.356135	-2.80792
Ptk2	Ptk2-201	0.0217065	0.911522	-1.09707
Ptma	Ptma-003	0.0482575	0.375637	-2.66215
Ptn	Ptn-001	0.00167879	1.12252	1.12252
Ptpn12	Ptpn12-007	0.0373819	5.34745e-05	-18700.5
Ptpn4	Ptpn4-011	0.0494644	0.0448951	-22.2742
Ptprf	Ptprf-006	0.000781374	9.80768	9.80768
Ptprg	Ptprg-001	0.0316983	0.884199	-1.13097
Ptprh	Ptprh-003	0.0416385	1.89161e+08	1.89161e+08
Ptprr	Ptprr-001	0.0483746	0.792291	-1.26216
Ptprr	Ptprr-007	0.0066189	2.53667	2.53667
Ptprs	Ptprs-002	0.0406776	0.935332	-1.06914
Ptrh1	Ptrh1-003	0.00512086	0.0982989	-10.1731
Ptrh1	Ptrh1-001	0.0136353	2.12591	2.12591
Pttg1	Pttg1-003	0.0118219	1.93928	1.93928
Pttg1ip	Pttg1ip-001	0.00155813	1.18174	1.18174
Puf60	Puf60-201	0.0408279	1.23281	1.23281
Pum1	Pum1-001	0.0108925	0.849856	-1.17667
Pum2	Pum2-007	0.0306447	7.56605e-05	-13216.9
Pus1	Pus1-006	0.00199152	0.0116077	-86.1494
Pus10	Pus10-002	0.0131371	0.363561	-2.75057
Pus3	Pus3-003	0.033454	1.4761	1.4761
Pvrl3	Pvrl3-002	0.0211307	0.802398	-1.24627
Pvrl4	Pvrl4-002	0.0356532	0.82974	-1.2052
Pvt1	Pvt1-001	0.0234716	0.454759	-2.19897
Pwwp2a	Pwwp2a-004	0.0455769	0.730419	-1.36908
Pwwp2a	Pwwp2a-003	0.00488192	2.72617	2.72617
Pycard	Pycard-002	0.0440262	1.69931	1.69931
Pyroxd1	Pyroxd1-004	0.0307692	0.714869	-1.39886
Pyroxd1	Pyroxd1-002	0.0420377	1.74924	1.74924
Pzp	Pzp-003	0.00569749	0.101205	-9.88089
Qars	Qars-006	0.0302592	0.00898928	-111.244
Qdpr	Qdpr-006	0.0473754	1.31911	1.31911
Qrich1	Qrich1-007	0.0453281	0.545652	-1.83267
Qrich1	Qrich1-008	0.0356038	0.205258	-4.87192
Qrsl1	Qrsl1-003	0.0141001	0.399992	-2.50005
R3hcc1	R3hcc1-002	0.0250574	0.671102	-1.49009
R3hcc1	R3hcc1-001	0.0155334	1.38872	1.38872
R3hdm1	R3hdm1-009	0.00252059	1.32781	1.32781
Rab10os	Rab10os-010	0.0167098	0.27283	-3.66529
Rab13	Rab13-001	0.0381586	1.30221	1.30221
Rab14	Rab14-003	0.0355346	6.56583e-08	-1.52304e+07
Rab15	Rab15-001	0.0308291	1.107	1.107

Rab22a	Rab22a-002	0.0233748	1.53128	1.53128
Rab23	Rab23-007	0.00347474	1.84227	1.84227
Rab30	Rab30-003	0.0236359	6.4693	6.4693
Rab39b	Rab39b-001	0.0116291	0.881897	-1.13392
Rab3d	Rab3d-005	0.0282604	17.6195	17.6195
Rab40c	Rab40c-201	0.00839125	1.95837	1.95837
Rab43	Rab43-201	0.0321941	2.33802	2.33802
Rab8a	Rab8a-001	0.0446424	1.06164	1.06164
Rabepk	Rabepk-006	0.0342419	0.627325	-1.59407
Rabgap1l	Rabgap1l-003	0.0310704	0.924197	-1.08202
Rabgef1	Rabgef1-006	0.0315282	1.89198	1.89198
Racgap1	Racgap1-005	0.000551064	8.56289	8.56289
Rad51ap1	Rad51ap1-001	0.00877026	0.545506	-1.83316
Radil	Radil-010	0.0292661	0.606493	-1.64882
Raf1	Raf1-004	0.0114678	0.00249957	-400.068
Ralgds	Ralgds-007	0.00622226	2.68535	2.68535
Ralgps2	Ralgps2-013	0.0446086	0.00737332	-135.624
Raly	Raly-004	0.0201336	2.13379	2.13379
Ran	Ran-002	0.0456376	0.668556	-1.49576
Rap1a	Rap1a-003	0.0453675	2.36165	2.36165
Rap1gds1	Rap1gds1-009	0.0487415	0.130667	-7.65303
Rap2b	Rap2b-001	0.0031035	0.847729	-1.17962
Rap2c	Rap2c-001	0.0225373	0.889008	-1.12485
Rapgef4	Rapgef4-010	0.00388702	0.154718	-6.46338
Rapgef4os2	Rapgef4os2-001	0.0120643	1.99627	1.99627
Rars	Rars-003	0.0118806	0.331561	-3.01604
Rars	Rars-004	0.0258422	3.30948	3.30948
Rasa1	Rasa1-003	0.0331157	0.351592	-2.84421
Rasal3	Rasal3-007	0.0269086	8.54836	8.54836
Rasgef1b	Rasgef1b-001	0.0401472	2.75519	2.75519
Rasgef1c	Rasgef1c-003	0.00149058	8.17889	8.17889
Rasgrf1	Rasgrf1-001	0.000115028	0.861581	-1.16066
Rasgrp1	Rasgrp1-001	0.0367385	0.856768	-1.16718
Rasgrp2	Rasgrp2-001	0.0421784	5.53752	5.53752
Rasgrp2	Rasgrp2-015	0.0408886	2.97529	2.97529
Rasgrp2	Rasgrp2-201	0.0432929	5.77707	5.77707
Rasl10b	Rasl10b-002	0.0379361	2.04081	2.04081
Rassf7	Rassf7-001	0.0107402	2.93532	2.93532
Rassf8	Rassf8-201	0.0440093	1.47562	1.47562
Rassf9	Rassf9-001	0.02799	1.861	1.861
Rb1cc1	Rb1cc1-014	0.0291785	10.8927	10.8927
Rbbp6	Rbbp6-006	0.0370889	0.00109595	-912.447
Rbm15b	Rbm15b-001	0.00351154	1.08202	1.08202

Rbm18	Rbm18-003	0.0255238	4.0907	4.0907
Rbm19	Rbm19-003	0.0308986	3.43871	3.43871
Rbm20	Rbm20-003	0.0439659	6.93098e-06	-144280
Rbm3	Rbm3-010	0.0470927	3.05957	3.05957
Rbm39	Rbm39-013	0.0471471	0.140113	-7.13712
Rbm39	Rbm39-010	0.005367	3.18861	3.18861
Rbm6	Rbm6-018	0.0315597	0.0142138	-70.3542
Rbms1	Rbms1-001	0.0279178	1.38944	1.38944
Rbms3	Rbms3-011	0.0378726	1.03863e-06	-962802
Rbp7	Rbp7-001	0.0353031	6.90958e+08	6.90958e+08
Rc3h2	Rc3h2-004	0.0235892	1.41629	1.41629
Rc3h2	Rc3h2-008	0.0305706	2.18937	2.18937
Rcan2	Rcan2-203	0.0439826	3.20418	3.20418
Rcbtb1	Rcbtb1-008	0.00219248	0.457734	-2.18467
Rccd1	Rccd1-009	0.0241691	2.0378	2.0378
Rchy1	Rchy1-002	0.000730824	0.548712	-1.82245
Rcor2	Rcor2-003	0.011953	0.253644	-3.94253
Rd3	Rd3-004	0.0329597	4.27287e+07	4.27287e+07
Rdh12	Rdh12-002	0.0204617	0.445436	-2.24499
Rdh13	Rdh13-006	0.0426154	2.34578	2.34578
Rdm1	Rdm1-004	0.0149638	5.78953e-06	-172726
Recql	Recql-003	0.005752	0.54394	-1.83844
Reep3	Reep3-201	0.0100647	1.08458	1.08458
Reep6	Reep6-003	0.00743966	0.0250784	-39.875
Relb	Relb-002	0.0362449	1.57995	1.57995
Ren1	Ren1-001	0.0331905	0.382268	-2.61596
Rep15	Rep15-001	0.0216378	1.60455	1.60455
Rev1	Rev1-001	0.0134312	0.893346	-1.11939
Rev1	Rev1-002	0.0401665	1.61269	1.61269
Rexo1	Rexo1-003	0.00278456	0.32956	-3.03435
Rexo1	Rexo1-008	0.00788667	3.55808	3.55808
Rexo1	Rexo1-007	0.0208188	1.85846	1.85846
Rfc4	Rfc4-005	0.034652	273.51	273.51
Rfng	Rfng-001	0.0125488	0.843259	-1.18588
Rfwd2	Rfwd2-002	0.0315493	2.47086	2.47086
Rfx4	Rfx4-001	0.0486189	5.32261e-10	-1.87878e+09
Rfx7	Rfx7-003	0.00482295	234.434	234.434
Rfxank	Rfxank-201	0.042892	0.827848	-1.20795
Rgmb	Rgmb-201	0.00296317	0.888829	-1.12508
Rgs12	Rgs12-009	0.0366137	150689	150689
Rgs19	Rgs19-008	0.0395992	0.139776	-7.15429
Rgs2	Rgs2-001	0.0157961	1.23636	1.23636
Rgs3	Rgs3-012	0.0306728	1.57021e-10	-6.36858e+09

Rhbdd3	Rhbdd3-201	0.0324244	2.18136	2.18136
Rhbdf1	Rhbdf1-006	0.0128757	0.391532	-2.55407
Rhbdf1	Rhbdf1-008	0.0206881	2.45013	2.45013
Rhbdl2	Rhbdl2-201	0.0446676	0.0315937	-31.6519
Rhbdl3	Rhbdl3-001	0.0174729	1.20902	1.20902
Rhno1	Rhno1-004	0.0370064	0.00499934	-200.026
Rhoa	Rhoa-004	0.0451361	15.827	15.827
Rhobtb1	Rhobtb1-007	0.00440258	0.186672	-5.35699
Rhpn1	Rhpn1-001	0.0384434	0.745029	-1.34223
Rhpn2	Rhpn2-002	0.0101942	2.49365e+08	2.49365e+08
Riiad1	Riiad1-003	0.0313513	0.00970337	-103.057
Rilp	Rilp-002	0.0122619	6.53044	6.53044
Rimklb	Rimklb-007	0.012076	0.559137	-1.78847
Rimklb	Rimklb-001	0.0413686	1.23669	1.23669
Rims2	Rims2-201	0.0305065	1.36416	1.36416
Rin2	Rin2-001	0.00571881	1.24159	1.24159
Rin2	Rin2-201	0.00571881	1.24159	1.24159
Ripk1	Ripk1-003	0.0180055	10.5134	10.5134
Rlbp1	Rlbp1-008	0.00975794	3.96733	3.96733
Rlf	Rlf-201	0.0150717	0.248929	-4.01721
Rln1	Rln1-001	0.0210694	0.481842	-2.07537
Rmdn2	Rmdn2-201	0.0429303	1.285	1.285
Rnf112	Rnf112-003	0.044226	0.817042	-1.22393
Rnf121	Rnf121-003	0.0395414	7.13902	7.13902
Rnf122	Rnf122-002	0.0304515	2.04772	2.04772
Rnf14	Rnf14-202	0.0334652	1.43013	1.43013
Rnf146	Rnf146-002	0.00450396	1.42143	1.42143
Rnf150	Rnf150-201	0.0293297	1.10309	1.10309
Rnf166	Rnf166-001	0.0111297	1.21598	1.21598
Rnf167	Rnf167-005	0.0371988	0.58676	-1.70427
Rnf169	Rnf169-001	0.0180007	1.12459	1.12459
Rnf207	Rnf207-012	0.00613187	3.09405	3.09405
Rnf207	Rnf207-006	0.0224255	2.84372	2.84372
Rnf208	Rnf208-001	0.049875	1.36547	1.36547
Rnf24	Rnf24-004	0.00194607	0.0664308	-15.0532
Rnf25	Rnf25-010	0.0364014	0.206051	-4.85317
Rnf26	Rnf26-005	0.0310695	6.00834e+10	6.00834e+10
Rnf38	Rnf38-017	0.0153659	1.19771	1.19771
Rnf39	Rnf39-002	0.031452	0.125021	-7.99865
Rnf4	Rnf4-011	0.000404668	11.4464	11.4464
Rnf43	Rnf43-001	0.00720042	4.48404	4.48404
Rnf44	Rnf44-014	0.0333765	67.6847	67.6847
Rnf44	Rnf44-011	0.0389033	2.8641	2.8641

Rnf6	Rnf6-201	0.0430729	0.691188	-1.44678
Rnf6	Rnf6-001	0.0327821	1.22035	1.22035
Rnft1	Rnft1-003	0.00205793	1.73953	1.73953
Rnmt	Rnmt-001	0.0096648	0.861515	-1.16075
Robo2	Robo2-002	0.0411277	1.36716	1.36716
Robo3	Robo3-002	0.034805	0.227095	-4.40345
Robo3	Robo3-005	0.0307994	34.6409	34.6409
Rock1	Rock1-201	0.0310507	1.12551	1.12551
Rom1	Rom1-201	0.0379683	1.65851	1.65851
Rorb	Rorb-001	0.0415498	0.557659	-1.79321
Rorb	Rorb-005	0.0393094	1.59203	1.59203
Rp2	Rp2-001	0.0136373	1.35897	1.35897
RP23-142A14.9	RP23-142A14.9-001	0.0298149	0.540861	-1.8489
RP23-144N15.8	RP23-144N15.8-002	0.0426348	5.44772	5.44772
RP23-179K11.4	RP23-179K11.4-001	0.0260909	3.11196	3.11196
RP23-26J4.3	RP23-26J4.3-001	0.00281807	0.665756	-1.50205
RP23-312I2.6	RP23-312I2.6-001	0.049387	0.842999	-1.18624
RP23-330J17.3	RP23-330J17.3-001	0.0256443	5.48954	5.48954
RP23-355N5.6	RP23-355N5.6-001	0.0341341	5.88352	5.88352
RP23-382G13.1	RP23-382G13.1-003	0.0471214	580112	580112
RP23-392K24.6	RP23-392K24.6-001	0.0257806	1.64334	1.64334
RP23-441J16.4	RP23-441J16.4-002	0.0353929	4.07233	4.07233
RP23-441J16.5	RP23-441J16.5-001	0.0201258	4.67439	4.67439
RP24-201D17.2	RP24-201D17.2-001	0.028422	0.135412	-7.38485
RP24-238C11.4	RP24-238C11.4-001	0.00424204	1.38794	1.38794
RP24-238J9.2	RP24-238J9.2-001	0.0381027	3.13613	3.13613
RP24-315D19.3	RP24-315D19.3-001	0.0304735	0.000602284	-1660.35
RP24-327I4.8	RP24-327I4.8-001	0.0298213	0.211395	-4.73049
RP24-576D1.5	RP24-576D1.5-001	0.0215611	0.133257	-7.50432
Rpa1	Rpa1-001	0.000142247	0.517944	-1.93071
Rpa2	Rpa2-002	0.030255	10.1757	10.1757
Rpap2	Rpap2-006	0.0118063	4.25863	4.25863
Rph3a	Rph3a-003	0.0435084	0.678476	-1.47389
Rpl10	Rpl10-009	0.00792944	0.274438	-3.64381
Rpl14-ps1	Rpl14-ps1-201	0.00181515	2.90697	2.90697
Rpl26	Rpl26-003	0.0167891	0.123546	-8.09416
Rpl26	Rpl26-201	0.0147179	3.01641	3.01641
Rpl3-ps1	Rpl3-ps1-001	0.0110231	0.699467	-1.42966
Rpl30	Rpl30-006	0.00108985	0.410705	-2.43484
Rpl35	Rpl35-002	0.0060488	1.39587	1.39587
Rpl35a	Rpl35a-006	0.0243648	0.0272329	-36.7204
Rpl35a	Rpl35a-004	0.0361211	1.2663	1.2663
Rpl36a	Rpl36a-002	0.0241451	3.53474	3.53474

Rpl37rt	Rpl37rt-003	0.0192594	9.0002	9.0002
Rpl38	Rpl38-004	0.0416517	0.28855	-3.4656
Rpl41	Rpl41-007	0.0499527	0.485447	-2.05996
Rpl5	Rpl5-006	0.0333877	0.67612	-1.47903
Rpl6	Rpl6-006	0.0100447	0.396395	-2.52274
Rpl6	Rpl6-003	0.0104496	7.53569	7.53569
Rpl7a	Rpl7a-006	0.0078856	1.84407e+11	1.84407e+11
Rpl7a	Rpl7a-004	0.0126615	1.80421	1.80421
Rpl9	Rpl9-009	0.00867256	5.96164	5.96164
Rpn2	Rpn2-002	0.00803156	0.492334	-2.03114
Rpn2	Rpn2-001	0.0203391	1.79065	1.79065
Rps19	Rps19-001	0.0128806	0.406947	-2.45732
Rps19	Rps19-006	0.0390657	1.33505	1.33505
Rps27	Rps27-008	0.0479256	0.436985	-2.28841
Rps3	Rps3-002	0.0155625	2.28929	2.28929
Rps3a3	Rps3a3-001	0.0314759	2.75988	2.75988
Rps5	Rps5-006	0.0364579	0.368197	-2.71593
Rps6ka1	Rps6ka1-004	0.0446608	0.0603897	-16.5591
Rps6ka2	Rps6ka2-201	0.00506209	1.16586	1.16586
Rps6ka6	Rps6ka6-004	0.00257758	4.90338e-11	-2.03941e+10
Rps6ka6	Rps6ka6-001	0.0207152	1.91013	1.91013
Rps6ka6	Rps6ka6-201	0.0176728	1.91013	1.91013
Rps9	Rps9-005	0.0306544	0.304752	-3.28135
Rptor	Rptor-002	0.0203952	1.11077e-09	-9.00275e+08
Rptor	Rptor-003	0.039437	0.833921	-1.19915
Rrh	Rrh-004	0.013461	3.67078e+07	3.67078e+07
Rrm2b	Rrm2b-001	0.0482878	0.88601	-1.12865
Rrm2b	Rrm2b-007	0.016448	1.7464	1.7464
Rrp1b	Rrp1b-005	0.0174167	5.21316	5.21316
Rrp7a	Rrp7a-001	0.00696839	1.10834	1.10834
Rrp9	Rrp9-005	0.0432301	0.168041	-5.95092
Rsf1	Rsf1-003	0.0430647	1.23329	1.23329
Rsl1d1	Rsl1d1-002	0.0228245	2.34671	2.34671
Rsph14	Rsph14-004	0.0190124	3.42549	3.42549
Rsrc2	Rsrc2-008	0.0216345	0.138856	-7.20173
Rtn3	Rtn3-202	0.0092546	0.864937	-1.15615
Rundc1	Rundc1-001	0.000746281	1.20359	1.20359
Rundc3a	Rundc3a-009	0.00761754	10.3548	10.3548
Rwdd3	Rwdd3-001	0.000611236	0.436724	-2.28978
Rwdd4a	Rwdd4a-005	0.0389444	0.36943	-2.70687
Rxfp2	Rxfp2-004	0.0103259	5.52801e-08	-1.80897e+07
Rxfp2	Rxfp2-003	0.0170195	6.61566	6.61566
Rxrg	Rxrg-002	0.0150784	2.90587	2.90587

S100a1	S100a1-001	0.0172925	1.16631	1.16631
S100a16	S100a16-007	0.0368387	2.14286	2.14286
S100pbp	S100pbp-002	0.0233886	1.55322	1.55322
Sacs	Sacs-004	0.0241801	7.52985	7.52985
Safb	Safb-008	0.0396926	966.076	966.076
Sall4	Sall4-005	0.000635833	0.137097	-7.29412
Samd15	Samd15-201	0.00238561	0.00502827	-198.875
Sap18	Sap18-004	0.0132998	0.137241	-7.28647
Sarm1	Sarm1-001	0.00175202	0.656929	-1.52224
Sart3	Sart3-003	0.0429126	5.8167	5.8167
Sat2	Sat2-001	0.00590873	0.579131	-1.72672
Sat2	Sat2-003	0.038762	0.765161	-1.30691
Sat2	Sat2-002	0.0480121	5.79157	5.79157
Sbk1	Sbk1-001	0.0130613	1.1703	1.1703
Sbno1	Sbno1-005	0.0489304	0.0193175	-51.7666
Sbno1	Sbno1-002	0.036298	3.18161	3.18161
Sbno1	Sbno1-012	0.038284	2.33146	2.33146
Sbno2	Sbno2-201	0.0128703	1.15231	1.15231
Sbsn	Sbsn-003	0.0342765	6523.68	6523.68
Sbspon	Sbspon-001	0.0446996	2.26756	2.26756
Scd1	Scd1-201	0.02427	1.15693	1.15693
Scg5	Scg5-002	0.0339439	0.726126	-1.37717
Schip1	Schip1-009	0.0209179	1.40132	1.40132
Sclt1	Sclt1-001	0.0308063	1.8322	1.8322
Scmh1	Scmh1-202	0.0118428	1.84971	1.84971
Scmh1	Scmh1-003	0.0224149	1.57392	1.57392
Scmh1	Scmh1-006	0.0469817	7.04362e+06	7.04362e+06
Scn8a	Scn8a-201	0.0128738	0.847858	-1.17944
Scnm1	Scnm1-007	0.0375196	0.452326	-2.21079
Scnm1	Scnm1-008	0.0404828	0.583315	-1.71434
Scnn1a	Scnn1a-004	0.0322496	0.384266	-2.60236
Sco2	Sco2-201	0.00574994	0.744864	-1.34253
Scp2	Scp2-003	0.0398992	0.20472	-4.88472
Scpep1	Scpep1-001	0.024679	1.22233	1.22233
Scrn3	Scrn3-001	0.0147969	0.790068	-1.26571
Sdcbp	Sdcbp-001	0.0444334	0.827987	-1.20775
Sdccag3	Sdccag3-011	0.0428976	0.239282	-4.17917
Sec11a	Sec11a-006	0.0419476	0.0253612	-39.4303
Sec11a	Sec11a-001	0.00363078	1.66535	1.66535
Sec14l2	Sec14l2-001	0.0280171	0.406137	-2.46222
Sec31a	Sec31a-001	0.0301697	0.894825	-1.11754
Sec61a1	Sec61a1-010	0.0124414	0.164867	-6.0655
Secisbp2	Secisbp2-001	0.0361315	1.10944	1.10944

Selenbp1	Selenbp1-007	0.0388947	127.884	127.884
Sema3a	Sema3a-001	0.0235486	1.9836	1.9836
Sema3b	Sema3b-007	0.0347604	0.000699277	-1430.05
Sema3b	Sema3b-003	0.0155532	1788.68	1788.68
Sema3f	Sema3f-001	0.0207795	2.5286	2.5286
Sema4a	Sema4a-202	0.0278991	0.528573	-1.89189
Sema4d	Sema4d-001	0.026031	0.722181	-1.38469
Sema6d	Sema6d-008	0.0398198	0.804872	-1.24243
Senp1	Senp1-005	0.0283459	2.53489	2.53489
Senp5	Senp5-003	0.0304907	0.259725	-3.85023
Senp6	Senp6-004	0.00799031	2.85455	2.85455
Senp7	Senp7-001	0.00281685	0.666401	-1.5006
Senp7	Senp7-011	0.020587	13.6761	13.6761
Senp7	Senp7-202	0.0197214	1.49036	1.49036
Sephs1	Sephs1-003	0.0257453	2.74997	2.74997
Sephs1	Sephs1-004	0.0212261	5.49992	5.49992
02-Sep	Sept2-203	0.0117131	1.26899	1.26899
06-Sep	Sept6-002	0.0102721	0.817452	-1.22331
08-Sep	Sept8-002	0.0484632	1.29367	1.29367
Serac1	Serac1-006	0.00371623	8.78096	8.78096
Serac1	Serac1-004	0.0394885	16784.2	16784.2
Serhl	Serhl-011	0.0351701	0.275199	-3.63374
Serhl	Serhl-015	0.0328682	7167.47	7167.47
Serpinb6a	Serpinb6a-010	0.000764751	192.537	192.537
Sertad2	Sertad2-001	0.0305244	0.527849	-1.89448
Sertm1	Sertm1-001	0.0345367	0.814523	-1.22771
Sesn3	Sesn3-001	0.0253987	1.27022	1.27022
Setd5	Setd5-004	0.0477329	2.24235	2.24235
Setd8	Setd8-004	0.0357553	11.5059	11.5059
Setmar	Setmar-002	0.0341257	0.0589134	-16.9741
Sez6	Sez6-007	0.0484495	1.27872	1.27872
Sez6l2	Sez6l2-007	0.0449368	1.51719	1.51719
Sf1	Sf1-003	0.0165309	2.26401	2.26401
Sf3a1	Sf3a1-001	0.0306033	1.84614	1.84614
Sfrp1	Sfrp1-001	0.000898562	2.61455	2.61455
Sft2d2	Sft2d2-001	0.00565667	1.24352	1.24352
Sfxn5	Sfxn5-009	0.0343711	0.65249	-1.53259
Sgca	Sgca-202	0.0102542	0.00136461	-732.812
Sgce	Sgce-010	0.0264342	2.13399	2.13399
Sgcg	Sgcg-001	0.023454	4.86356	4.86356
Sgk3	Sgk3-010	0.000730703	2.53888	2.53888
Sgpl1	Sgpl1-003	0.0369404	4.54626e-06	-219961
Sgsm2	Sgsm2-002	0.0195373	0.839256	-1.19153

Sh2b1	Sh2b1-010	0.027838	3571.46	3571.46
Sh2b1	Sh2b1-002	0.0485485	1.72312	1.72312
Sh2d4b	Sh2d4b-002	0.0379216	2.48576	2.48576
Sh3bgr	Sh3bgr-004	0.0452219	0.0041943	-238.419
Sh3bgr	Sh3bgr-005	0.0111215	6.67223	6.67223
Sh3bp1	Sh3bp1-002	0.0114715	9.32405	9.32405
Sh3bp5l	Sh3bp5l-001	0.0432541	1.25826	1.25826
Sh3d19	Sh3d19-001	0.0128332	1.44063	1.44063
Sh3gl1	Sh3gl1-201	0.0218665	1.08496	1.08496
Sh3glb1	Sh3glb1-003	0.0184112	1.10942	1.10942
Sh3pxd2b	Sh3pxd2b-001	0.0149051	1.19277	1.19277
Sh3rf2	Sh3rf2-002	0.045372	8.33863	8.33863
Shb	Shb-001	0.0365522	1.37407	1.37407
Shisa6	Shisa6-002	0.0317294	0.794255	-1.25904
Shisa7	Shisa7-003	0.00237757	1.45433	1.45433
Shkbp1	Shkbp1-004	0.00119535	0.0953921	-10.483
Shkbp1	Shkbp1-011	0.00916423	2.10689	2.10689
Shpk	Shpk-002	5.27982e-05	0.617403	-1.61969
Shtn1	Shtn1-201	0.00010121	1.7391	1.7391
Sik1	Sik1-201	0.0400648	1.33688	1.33688
Sipa1l1	Sipa1l1-202	0.0258559	0.863031	-1.15871
Sipa1l3	Sipa1l3-013	0.024316	3.47269	3.47269
Sirpa	Sirpa-001	0.0102	0.897299	-1.11446
Sirpa	Sirpa-201	0.0198956	2.07666	2.07666
Sirt1	Sirt1-004	0.00723894	3.22747	3.22747
Sirt7	Sirt7-006	0.0150676	1.55089	1.55089
Sirt7	Sirt7-009	0.0406446	2.01789	2.01789
Six3os1	Six3os1-013	0.0148559	35038.3	35038.3
Six3os1	Six3os1-002	0.0234724	6.25249	6.25249
Six3os1	Six3os1-001	0.0358688	20.416	20.416
Skida1	Skida1-001	0.0199467	0.754322	-1.32569
Skida1	Skida1-201	0.0165849	2.21646	2.21646
Skil	Skil-003	0.0141817	0.316586	-3.1587
Skp2	Skp2-001	0.0248415	1.75735	1.75735
Slamf6	Slamf6-003	0.0345386	5.14304	5.14304
Slbp	Slbp-007	0.0360405	0.0890201	-11.2334
Slc12a3	Slc12a3-201	0.0305343	6.88085	6.88085
Slc12a4	Slc12a4-003	0.0215826	13.3038	13.3038
Slc16a8	Slc16a8-001	0.020406	10.6796	10.6796
Slc17a7	Slc17a7-201	0.0445068	0.907829	-1.10153
Slc1a1	Slc1a1-001	0.0190925	0.909019	-1.10009
Slc1a4	Slc1a4-201	0.0200821	0.815563	-1.22615
Slc22a3	Slc22a3-201	0.0134621	2.44568	2.44568

Slc24a2	Slc24a2-201	0.0101614	0.81879	-1.22131
Slc25a19	Slc25a19-001	0.0071246	1.34322	1.34322
Slc25a22	Slc25a22-002	0.0417915	0.87109	-1.14799
Slc25a25	Slc25a25-001	0.0205397	1.47223	1.47223
Slc25a33	Slc25a33-001	0.000417148	1.18418	1.18418
Slc25a38	Slc25a38-001	0.0141179	1.20411	1.20411
Slc25a42	Slc25a42-004	0.00334257	1.51078	1.51078
Slc25a48	Slc25a48-001	0.0166406	0.392592	-2.54718
Slc26a2	Slc26a2-002	0.0395552	1.22671e-05	-81519
Slc27a3	Slc27a3-001	0.0201463	2.29097	2.29097
Slc27a4	Slc27a4-002	0.0231177	2.75788	2.75788
Slc28a3	Slc28a3-001	0.0449579	1.52297	1.52297
Slc29a1	Slc29a1-005	0.0488664	0.175208	-5.7075
Slc2a1	Slc2a1-001	0.0405955	1.21587	1.21587
Slc2a6	Slc2a6-003	0.0141016	0.690989	-1.4472
Slc30a9	Slc30a9-004	0.00332634	0.0475535	-21.029
Slc35a1	Slc35a1-005	0.0243449	0.246589	-4.05533
Slc35a1	Slc35a1-001	0.0476048	1.17233	1.17233
Slc35b1	Slc35b1-002	0.0296468	0.850004	-1.17646
Slc35b1	Slc35b1-005	0.0198345	0.493276	-2.02726
Slc35b1	Slc35b1-001	0.0280968	1.34269	1.34269
Slc35b2	Slc35b2-201	0.0482939	1.1313	1.1313
Slc35c1	Slc35c1-001	0.0244334	0.801523	-1.24763
Slc35c2	Slc35c2-002	0.0384844	2.78375	2.78375
Slc37a3	Slc37a3-010	0.0414383	0.00878188	-113.871
Slc37a3	Slc37a3-013	6.9588e-05	15.3379	15.3379
Slc37a3	Slc37a3-004	0.0039323	3.09295	3.09295
Slc37a4	Slc37a4-201	0.0270179	0.904528	-1.10555
Slc38a10	Slc38a10-007	0.0266198	4.24036e-07	-2.35829e+06
Slc38a3	Slc38a3-005	0.0222475	1.59307	1.59307
Slc38a3	Slc38a3-003	0.0221294	1.72549	1.72549
Slc38a3	Slc38a3-002	0.0367558	1.34507	1.34507
Slc38a4	Slc38a4-202	0.00775274	1074.94	1074.94
Slc39a13	Slc39a13-008	0.0445161	0.58087	-1.72155
Slc39a3	Slc39a3-003	0.0148587	0.117231	-8.53019
Slc39a3	Slc39a3-004	0.00299424	3.29589	3.29589
Slc39a7	Slc39a7-007	0.0253877	0.0984384	-10.1586
Slc39a7	Slc39a7-005	0.00410476	2.64261	2.64261
Slc39a8	Slc39a8-003	0.0177956	0.234314	-4.26779
Slc44a5	Slc44a5-002	0.0326031	0.881265	-1.13473
Slc44a5	Slc44a5-001	0.0474111	0.757443	-1.32023
Slc45a3	Slc45a3-002	0.0362139	3.61471	3.61471
Slc4a10	Slc4a10-003	0.0127668	1.3819	1.3819

Slc4a1ap	Slc4a1ap-005	0.0296423	6.17525	6.17525
Slc4a1ap	Slc4a1ap-006	0.0228153	5.87042	5.87042
Slc4a1ap	Slc4a1ap-013	0.0373617	64305.8	64305.8
Slc4a1ap	Slc4a1ap-012	0.0463153	25607.2	25607.2
Slc4a2	Slc4a2-001	0.0300507	3.88149	3.88149
Slc4a7	Slc4a7-201	0.00865003	0.830959	-1.20343
Slc50a1	Slc50a1-008	0.0462971	0.00965598	-103.563
Slc5a11	Slc5a11-201	0.0229081	0.221461	-4.51547
Slc6a1	Slc6a1-005	0.0240678	4.88168	4.88168
Slc6a15	Slc6a15-202	0.0324301	0.884363	-1.13076
Slc6a20a	Slc6a20a-003	0.0360231	207.022	207.022
Slc6a9	Slc6a9-012	0.0396137	0.687753	-1.45401
Slc7a14	Slc7a14-002	0.0352428	2.76517	2.76517
Slc7a15	Slc7a15-001	0.0365965	2.85245e-06	-350575
Slc7a7	Slc7a7-003	0.0431791	2.54344e-07	-3.93169e+06
Slc9b2	Slc9b2-003	0.0291363	0.278609	-3.58926
Slco1c1	Slco1c1-002	0.00150722	1.67323	1.67323
Slco2a1	Slco2a1-002	0.0263241	0.334225	-2.99199
Slf1	Slf1-002	0.0293535	1.37498	1.37498
Slitrk1	Slitrk1-201	0.00582221	0.848929	-1.17795
Slitrk2	Slitrk2-001	0.0470245	0.822371	-1.216
Slitrk3	Slitrk3-001	0.00747286	0.843191	-1.18597
Slitrk4	Slitrk4-002	0.0298529	0.636922	-1.57005
Slitrk5	Slitrk5-201	0.0115543	0.836453	-1.19552
Slmap	Slmap-009	0.0138953	0.000124076	-8059.57
Slmap	Slmap-001	0.0453142	0.874281	-1.1438
Slmo2	Slmo2-002	0.00289472	0.131035	-7.63156
Slpi	Slpi-001	0.0331617	3.5334e-06	-283014
Slx1b	Slx1b-004	0.0440074	0.500632	-1.99747
Slx4	Slx4-008	0.0453652	2.79229	2.79229
Smad4	Smad4-001	0.0445379	1.11512	1.11512
Smad5	Smad5-001	0.00127087	1.14445	1.14445
Smarca5	Smarca5-005	0.0242129	0.239494	-4.17548
Smarcad1	Smarcad1-012	0.00149186	1.44843e-10	-6.90403e+09
Smarcd2	Smarcd2-003	0.0308515	0.0698175	-14.3231
Smc2	Smc2-002	0.0388067	0.605957	-1.65028
Smc2os	Smc2os-001	0.00821473	0.302248	-3.30854
Smc3	Smc3-001	0.0158479	1.08704	1.08704
Smg7	Smg7-004	0.0260868	0.0283711	-35.2472
Smim5	Smim5-001	0.0177199	3.3174	3.3174
Smn1	Smn1-008	0.0440115	44.2859	44.2859
Smox	Smox-009	0.00875165	2.45023	2.45023
Smpd4	Smpd4-012	0.0393737	0.533728	-1.87362

Smpd4	Smpd4-007	0.0246638	7.34111	7.34111
Smug1	Smug1-201	0.0448207	1.16161	1.16161
Smurf2	Smurf2-001	0.0212928	1.12029	1.12029
Smyd4	Smyd4-002	0.0467806	0.419403	-2.38434
Snapc4	Snapc4-201	0.0334586	1.41106	1.41106
Snapin	Snapin-003	0.00311592	1.88102	1.88102
Sncaip	Sncaip-003	0.0433671	0.348262	-2.8714
Sncaip	Sncaip-005	0.00660771	12.1548	12.1548
Snd1	Snd1-002	0.034024	0.589801	-1.69549
Sned1	Sned1-006	0.0172658	9.16938	9.16938
Sned1	Sned1-005	0.0449925	8.33794	8.33794
Snhg17	Snhg17-004	0.0336124	0.321257	-3.11278
Snhg17	Snhg17-001	0.0147875	11.7457	11.7457
Snhg5	Snhg5-008	0.042867	1.49035	1.49035
Snhg6	Snhg6-005	0.0478299	0.302342	-3.30752
Snord104	Snord104-201	0.0174453	0.338213	-2.95672
Snord14e	Snord14e-201	0.0452348	1.46691e+06	1.46691e+06
Snord7	Snord7-201	0.019853	0.262463	-3.81006
Snrnp27	Snrnp27-001	0.0295561	0.920431	-1.08645
Snrpg	Snrpg-001	0.0191189	0.640283	-1.56181
Sntg1	Sntg1-005	0.0236565	0.716242	-1.39618
Sntg1	Sntg1-004	0.0194384	0.310657	-3.21898
Snurf	Snurf-001	0.0140819	0.820065	-1.21942
Snx13	Snx13-201	0.0399078	0.916419	-1.0912
Snx15	Snx15-002	0.00599619	0.124257	-8.04787
Snx27	Snx27-009	0.0434692	0.00339764	-294.322
Snx33	Snx33-201	0.0183256	1.25804	1.25804
Snx5	Snx5-003	0.0156225	0.902237	-1.10836
Snx7	Snx7-004	0.0125631	0.357726	-2.79544
Snx8	Snx8-005	0.0314605	4.3034	4.3034
Soat1	Soat1-007	0.0267168	3.73148	3.73148
Sorbs2	Sorbs2-024	0.0113564	5.03129	5.03129
Sorbs2	Sorbs2-021	0.0189102	51561.3	51561.3
Sorbs3	Sorbs3-201	0.0277617	1.22191	1.22191
Sort1	Sort1-001	0.0244107	0.89746	-1.11426
Sox11	Sox11-001	9.04049e-05	1.34153	1.34153
Sox2ot	Sox2ot-014	0.00390617	0.291394	-3.43179
Sox8	Sox8-002	0.0154802	5.75416	5.75416
Sp1	Sp1-005	0.00966952	34.2984	34.2984
Sp100	Sp100-012	0.0303374	0.217391	-4.6
Sp3	Sp3-001	0.0362054	1.32839	1.32839
Spag6	Spag6-001	0.0368709	1.34778	1.34778
Sparc	Sparc-005	0.0351404	0.00346521	-288.583

Sparc	Sparc-006	0.0194444	2.29626e+07	2.29626e+07
Spata1	Spata1-004	0.0177573	2.45294	2.45294
Spata18	Spata18-003	0.0348299	0.0119745	-83.5106
Spata2l	Spata2l-202	0.0228906	1.25475	1.25475
Spata31d1d	Spata31d1d-001	0.0466811	0.189678	-5.27209
Spata32	Spata32-001	0.0399474	0.525524	-1.90286
Spdya	Spdya-003	0.0417179	3.6738e+06	3.6738e+06
Specc1	Specc1-013	0.0299806	0.873847	-1.14437
Specc1l	Specc1l-201	0.0298027	1.31267	1.31267
Speer5-ps1	Speer5-ps1-001	0.0168954	6.58725	6.58725
Speg	Speg-011	0.0417988	2.4112	2.4112
Spink10	Spink10-006	0.0311913	3.7167e+06	3.7167e+06
Spink8	Spink8-001	0.0219414	0.763383	-1.30996
Spint2	Spint2-201	0.0152526	4.21323	4.21323
Spock3	Spock3-003	0.0484426	1.35075	1.35075
Spopl	Spopl-002	0.0223273	3.55292	3.55292
Spp1	Spp1-003	0.0261254	23.4985	23.4985
Spred1	Spred1-001	0.0158028	1.13326	1.13326
Sprn	Sprn-001	0.00554654	0.851986	-1.17373
Spsb1	Spsb1-009	0.0489926	19.6058	19.6058
Sptan1	Sptan1-004	0.00121129	0.907147	-1.10236
Sptan1	Sptan1-008	0.00631298	0.654582	-1.52769
Sptb	Sptb-001	0.0331714	0.92719	-1.07853
Sptb	Sptb-002	0.000325026	2.47037	2.47037
Src	Src-001	0.03087	0.831485	-1.20267
Srfbp1	Srfbp1-201	0.019563	0.913819	-1.09431
Srgap2	Srgap2-005	0.0113431	1.42339	1.42339
Srgap3	Srgap3-006	0.0491571	0.613778	-1.62925
Sri	Sri-001	0.0330318	0.612016	-1.63394
Srl	Srl-003	0.033915	4.71758	4.71758
Srm	Srm-001	0.0338187	1.12339	1.12339
Srms	Srms-001	0.0205122	0.517787	-1.93129
Srpk1	Srpk1-003	0.00971144	0.21846	-4.5775
Srrt	Srrt-009	0.041518	1.34462	1.34462
Srsf1	Srsf1-001	0.00556959	1.17251	1.17251
Srsf10	Srsf10-006	0.0099341	2.80052	2.80052
Srsf11	Srsf11-016	0.0475173	0.604029	-1.65555
Srsf6	Srsf6-004	0.0475437	1.24518	1.24518
Ssbp2	Ssbp2-009	0.00997382	0.180133	-5.55146
Ssbp3	Ssbp3-014	0.0335101	0.585637	-1.70754
Ssfa2	Ssfa2-005	0.00585957	2.53082	2.53082
Ssr2	Ssr2-003	0.0478541	4.06311	4.06311
Sssca1	Sssca1-004	0.0187868	0.350351	-2.85428

St14	St14-004	0.0437579	3.95504	3.95504
St5	St5-203	0.016937	0.24186	-4.13463
St6gal1	St6gal1-002	0.0186063	137.556	137.556
St6galnac2	St6galnac2-002	0.0334575	2.07444	2.07444
St6galnac2	St6galnac2-001	0.0205824	2.61089	2.61089
St6galnac5	St6galnac5-001	0.0076705	0.837475	-1.19407
St7	St7-007	0.0318691	3.58484	3.58484
St7l	St7l-002	0.00588245	0.662624	-1.50915
St7l	St7l-010	0.000605665	2.3269	2.3269
Stab1	Stab1-006	0.0376743	0.0233228	-42.8766
Stam	Stam-009	0.0252933	0.91681	-1.09074
Stambp	Stambp-001	0.00130226	1.5942	1.5942
Stard10	Stard10-006	0.00180153	6.87536	6.87536
Stard3	Stard3-001	0.029543	1.15139	1.15139
Stat5b	Stat5b-002	0.0488311	2.48244	2.48244
Steap2	Steap2-004	0.039494	3.40071	3.40071
Steap2	Steap2-002	0.00457019	8.8076	8.8076
Steap3	Steap3-005	0.0013813	14.9308	14.9308
Steap3	Steap3-002	0.00331778	6.96077	6.96077
Stk17b	Stk17b-001	0.00710795	1.28985	1.28985
Stk25	Stk25-003	0.00713628	0.815354	-1.22646
Stk32c	Stk32c-002	0.0426357	1.39754	1.39754
Stk35	Stk35-002	0.0161067	1.1311	1.1311
Stk38	Stk38-015	0.00338102	0.417482	-2.39531
Stpg1	Stpg1-002	0.0148396	7.56512	7.56512
Stra6	Stra6-012	0.0169558	0.00339333	-294.696
Stra6	Stra6-002	0.0259194	4.8294	4.8294
Strap	Strap-002	0.0374645	4.91632	4.91632
Strip2	Strip2-002	0.0391137	2.73946	2.73946
Strn	Strn-003	0.00143991	5018.26	5018.26
Stx18	Stx18-009	0.0301007	3.31768	3.31768
Stx1a	Stx1a-003	0.0186456	1.79027	1.79027
Stx8	Stx8-004	0.00114329	0.00654886	-152.698
Stxbp2	Stxbp2-010	0.0451739	0.14101	-7.09169
Styk1	Styk1-003	0.0062802	3.3398	3.3398
Sucla2	Sucla2-002	0.0431017	0.181248	-5.51731
Suco	Suco-001	0.0298845	1.07987	1.07987
Sulf1	Sulf1-002	0.0225224	9.77199	9.77199
Sumf1	Sumf1-006	0.0079671	0.244841	-4.08428
Sumf1	Sumf1-001	0.018394	1.16942	1.16942
Sumo1	Sumo1-004	0.0211995	0.019904	-50.2411
Sun1	Sun1-005	0.0233208	17.1437	17.1437
Sun2	Sun2-002	0.0287362	1.54282	1.54282

Supt20	Supt20-001	0.0134426	0.382235	-2.61619
Supt20	Supt20-201	0.0365709	0.367682	-2.71974
Supt20	Supt20-009	0.00713357	4.33474	4.33474
Supt3	Supt3-001	0.0234776	0.449714	-2.22363
Supt7l	Supt7l-003	0.0227669	1.50867	1.50867
Supv3l1	Supv3l1-002	0.026777	1.97332	1.97332
Surf2	Surf2-001	0.0169771	0.772803	-1.29399
Surf6	Surf6-005	0.02915	118.002	118.002
Surf6	Surf6-001	0.0278479	1.15096	1.15096
Suv420h1	Suv420h1-001	0.00534423	0.766697	-1.3043
Svil	Svil-008	0.041617	32.3837	32.3837
Swi5	Swi5-003	0.00383659	0.285315	-3.5049
Swt1	Swt1-009	0.0419748	0.234675	-4.26121
Swt1	Swt1-003	0.0343171	17.4418	17.4418
Syap1	Syap1-002	0.0285839	0.501216	-1.99515
Sybu	Sybu-201	0.0305308	0.671169	-1.48994
Sycp1	Sycp1-003	9.74892e-05	0.282182	-3.54381
Sycp1	Sycp1-001	0.0266672	1.63793	1.63793
Syn2	Syn2-001	0.00703738	0.879496	-1.13701
Syn2	Syn2-003	0.00460154	0.849474	-1.1772
Syn2	Syn2-005	0.0213399	0.682878	-1.46439
Syncrip	Syncrip-007	0.0199091	3.714	3.714
Syncrip	Syncrip-006	0.00839625	2.62509	2.62509
Syne1	Syne1-201	0.0265898	0.867357	-1.15293
Syne2	Syne2-001	0.0499261	1.77306	1.77306
Syne2	Syne2-201	0.0490965	1.8577	1.8577
Syngr2	Syngr2-007	0.0267339	0.111571	-8.96287
Synj1	Synj1-002	0.00426762	0.822557	-1.21572
Synj2	Synj2-011	0.0159129	5.09799	5.09799
Sypl	Sypl-006	0.00308091	14.3568	14.3568
Syt14	Syt14-004	0.0477377	0.0600199	-16.6611
Syt2	Syt2-003	0.00113966	0.14289	-6.9984
Sytl2	Sytl2-001	0.0271942	0.446871	-2.23778
Sytl2	Sytl2-005	0.00714696	4.06069	4.06069
Szt2	Szt2-003	0.0232287	5.02296	5.02296
Tab2	Tab2-004	0.0413037	2.02434	2.02434
Tacc2	Tacc2-201	0.0026591	0.840924	-1.18917
Tacc2	Tacc2-202	0.0016057	2.09571	2.09571
Tada1	Tada1-002	0.0179736	1.29527	1.29527
Tada3	Tada3-004	0.0402065	0.35724	-2.79924
Taf1	Taf1-201	0.0260762	1.99772	1.99772
Taf10	Taf10-001	0.0494596	1.16221	1.16221
Taf13	Taf13-003	0.0473561	1.25588	1.25588

Taf15	Taf15-004	0.0325348	3.05613e-05	-32721.1
Taf1b	Taf1b-201	0.038226	0.926618	-1.07919
Taf1c	Taf1c-005	0.0199882	152.927	152.927
Taf4a	Taf4a-001	0.024288	1.13595	1.13595
Taf6	Taf6-001	0.00220319	1.56755	1.56755
Taf6l	Taf6l-001	0.0203883	0.484281	-2.06492
Taf6l	Taf6l-203	0.0221488	4.94292	4.94292
Tagap	Tagap-001	0.0303116	0.705171	-1.4181
Tagap1	Tagap1-201	0.0160523	1.37149	1.37149
Tal1	Tal1-003	0.0219817	0.145689	-6.86393
Tamm41	Tamm41-005	0.024634	0.507995	-1.96852
Tamm41	Tamm41-003	0.0229296	1.59703	1.59703
Tanc1	Tanc1-012	0.0103665	3.46598	3.46598
Taok3	Taok3-001	0.00769877	0.728316	-1.37303
Tardbp	Tardbp-019	0.00746993	24.9092	24.9092
Tars2	Tars2-017	0.0449764	0.119147	-8.39301
Tars2	Tars2-016	0.0490217	0.21956	-4.55457
Tatdn1	Tatdn1-201	0.000611373	0.858986	-1.16416
Tatdn3	Tatdn3-001	0.0322234	0.403885	-2.47595
Taz	Taz-019	0.0150417	0.546368	-1.83027
Taz	Taz-007	0.020123	2.96355	2.96355
Tbc1d16	Tbc1d16-001	0.036617	1.17212	1.17212
Tbc1d17	Tbc1d17-005	0.0262478	0.337934	-2.95916
Tbc1d24	Tbc1d24-201	0.00251909	0.77658	-1.2877
Tbc1d24	Tbc1d24-011	0.04423	0.164068	-6.09503
Tbc1d2b	Tbc1d2b-002	0.0156486	0.255969	-3.90672
Tbc1d5	Tbc1d5-201	0.0275093	0.932453	-1.07244
Tbc1d8b	Tbc1d8b-001	0.0287765	0.851202	-1.17481
Tbl3	Tbl3-002	0.00638432	1.26453	1.26453
Tbp	Tbp-009	0.0493443	1.71271	1.71271
Tbxas1	Tbxas1-002	0.0310359	0.504881	-1.98067
Tcam1	Tcam1-002	0.049677	0.514772	-1.94261
Tcea2	Tcea2-001	0.0455419	0.710168	-1.40812
Tceanc2	Tceanc2-010	0.0425774	0.386052	-2.59032
Tceb1	Tceb1-009	0.0477152	0.733589	-1.36316
Tceb1	Tceb1-004	0.011497	15.611	15.611
Tcf4	Tcf4-201	0.0101901	0.853107	-1.17219
Tcf7l2	Tcf7l2-007	0.0421369	32.8566	32.8566
Tcfl5	Tcfl5-001	0.0255683	5.99127	5.99127
Tchp	Tchp-005	0.00389055	0.00536988	-186.224
Tcp11	Tcp11-005	0.0378746	6.21693	6.21693
Tdp1	Tdp1-201	0.0197406	1.83456	1.83456
Tdp2	Tdp2-002	0.0345917	9.81773	9.81773

Tead1	Tead1-003	0.0159139	0.0705292	-14.1785
Tead2	Tead2-004	0.0462149	4.45352e-06	-224541
Tead2	Tead2-007	0.0469221	5.34089e+08	5.34089e+08
Tead2	Tead2-012	0.0492998	61.7467	61.7467
Tead4	Tead4-003	0.0314153	6.53884e+07	6.53884e+07
Tecpr1	Tecpr1-008	0.0290183	0.488135	-2.04861
Telo2	Telo2-005	0.0317883	0.616421	-1.62227
Tenm4	Tenm4-003	0.0487665	0.108012	-9.25823
Terf1	Terf1-004	0.0171541	139.005	139.005
Tesc	Tesc-003	0.00990112	5.37647	5.37647
Tex30	Tex30-001	0.00370679	31.129	31.129
Tex9	Tex9-002	0.00262583	5.67702	5.67702
Tex9	Tex9-005	0.00414203	2.26189	2.26189
Tex9	Tex9-009	0.0123629	91.5765	91.5765
Tfdp2	Tfdp2-012	0.00575853	0.0305429	-32.7408
Tfg	Tfg-004	0.0367541	9.669e-06	-103423
Tfip11	Tfip11-004	0.0287832	0.104263	-9.5911
Tfpi	Tfpi-002	0.0252131	5.96727	5.96727
Tfr2	Tfr2-004	0.0413595	0.410233	-2.43764
Tfr2	Tfr2-005	0.0402041	978.36	978.36
Tfrc	Tfrc-001	0.0141754	0.85492	-1.1697
Tgfa	Tgfa-001	0.0325934	1.3953	1.3953
Tgfb1i1	Tgfb1i1-006	0.0463889	0.0165464	-60.436
Tgif1	Tgif1-003	0.0275146	0.0252255	-39.6424
Tgif1	Tgif1-006	0.0284583	7.87104	7.87104
Tgoln1	Tgoln1-001	0.0336773	1.10061	1.10061
Thap2	Thap2-201	0.0208247	1.17081	1.17081
Thap4	Thap4-002	0.0156816	1.30836	1.30836
Thbs3	Thbs3-004	0.02875	3.54517	3.54517
Thnsl2	Thnsl2-002	0.0493271	0.528193	-1.89325
Thoc6	Thoc6-007	0.0405624	8.6293e-07	-1.15884e+06
Thrap3	Thrap3-005	0.0141187	0.174504	-5.73053
Thsd1	Thsd1-002	0.0167102	0.506573	-1.97405
Thsd1	Thsd1-003	0.0405874	2.36723	2.36723
Tia1	Tia1-018	0.000119217	0.0524164	-19.078
Tia1	Tia1-016	0.0132225	0.541629	-1.84628
Tia1	Tia1-005	0.0137218	0.753082	-1.32788
Tia1	Tia1-022	0.0360966	4.28526	4.28526
Tial1	Tial1-012	0.0072982	0.131341	-7.61374
Tiam1	Tiam1-003	0.0153713	2.13387	2.13387
Tifab	Tifab-201	0.02228	0.775252	-1.2899
Timm13	Timm13-201	0.0362162	1.16695	1.16695
Timm17a	Timm17a-001	0.0461199	0.94326	-1.06015

Timm17b	Timm17b-003	0.0432139	2.03656	2.03656
Timm22	Timm22-002	0.0414168	1.63978	1.63978
Timm23	Timm23-006	0.010676	3.43474	3.43474
Timm44	Timm44-001	0.0232277	1.26462	1.26462
Timm8a1	Timm8a1-001	0.0156835	1.11587	1.11587
Tinagl1	Tinagl1-002	0.032011	5432.11	5432.11
Tktl1	Tktl1-001	0.03223	0.157831	-6.3359
Tldc1	Tldc1-201	0.0431117	1.24499	1.24499
Tle3	Tle3-018	0.0372274	0.00293292	-340.957
Tle3	Tle3-010	0.0170826	7.62543	7.62543
Tlr6	Tlr6-001	0.00993218	0.524055	-1.9082
Tm7sf3	Tm7sf3-002	0.0394002	7738	7738
Tm9sf1	Tm9sf1-007	0.0116329	3.3669	3.3669
Tmbim6	Tmbim6-001	0.0389711	1.08235	1.08235
Tmbim6	Tmbim6-009	0.0495955	4.46295	4.46295
Tmcc2	Tmcc2-004	0.0462266	0.665714	-1.50215
Tmco5	Tmco5-003	0.0131175	31.4529	31.4529
Tmed3	Tmed3-001	0.0485586	1.16637	1.16637
Tmed4	Tmed4-003	0.00683949	0.602826	-1.65885
Tmeff1	Tmeff1-002	0.00415222	2.86478	2.86478
Tmem100	Tmem100-001	0.000287225	1.44762	1.44762
Tmem116	Tmem116-005	0.022205	0.15386	-6.49942
Tmem125	Tmem125-005	0.0475352	4.31507e-06	-231746
Tmem132b	Tmem132b-001	0.000463175	0.892504	-1.12044
Tmem144	Tmem144-001	0.0492446	0.766929	-1.3039
Tmem161a	Tmem161a-002	0.0482995	0.606755	-1.64811
Tmem161a	Tmem161a-012	0.0151302	5.04503	5.04503
Tmem180	Tmem180-004	0.0310364	2.53421	2.53421
Tmem181a	Tmem181a-001	0.0329922	0.799818	-1.25028
Tmem196	Tmem196-005	0.0481601	1.30805	1.30805
Tmem202	Tmem202-201	0.0165908	1.81436	1.81436
Tmem210	Tmem210-002	0.0304332	186.95	186.95
Tmem229b	Tmem229b-003	0.0325771	1.64822	1.64822
Tmem232	Tmem232-201	0.0295934	3.6334	3.6334
Tmem234	Tmem234-010	0.0242059	0.713789	-1.40097
Tmem234	Tmem234-009	0.0407076	1.23054	1.23054
Tmem237	Tmem237-002	0.0382427	0.360698	-2.77241
Tmem237	Tmem237-004	0.0152917	3.47813	3.47813
Tmem255a	Tmem255a-002	0.00555879	0.540869	-1.84888
Tmem29	Tmem29-002	0.0123958	0.624295	-1.60181
Tmem37	Tmem37-001	0.0295352	1.75043	1.75043
Tmem40	Tmem40-004	0.0347415	2.63215	2.63215
Tmem41b	Tmem41b-001	0.0059159	0.777789	-1.2857

Tmem59l	Tmem59l-201	0.00417044	0.822997	-1.21507
Tmem64	Tmem64-001	0.0370879	1.13305	1.13305
Tmem72	Tmem72-001	0.0112327	11.139	11.139
Tmem8	Tmem8-001	0.00504287	1.22388	1.22388
Tmem82	Tmem82-002	0.0473165	1.92816	1.92816
Tmem8b	Tmem8b-002	0.00429448	1.43931	1.43931
Tmem91	Tmem91-001	0.0192987	2.15638	2.15638
Tmem98	Tmem98-001	0.0432587	1.5655	1.5655
Tmie	Tmie-001	0.04588	1.44841	1.44841
Tmod3	Tmod3-201	0.0273115	1.14296	1.14296
Tmprss11a	Tmprss11a-002	0.00217692	9.79107	9.79107
Tmprss11a	Tmprss11a-001	0.0279482	30.4489	30.4489
Tmsb15l	Tmsb15l-201	0.00114941	0.249427	-4.00918
Tmtc1	Tmtc1-003	0.0330536	1.44895	1.44895
Tmtc3	Tmtc3-001	0.0172155	1.17598	1.17598
Tmub2	Tmub2-003	0.0300878	0.812039	-1.23147
Tnfaip2	Tnfaip2-008	0.0354855	3.8873e-05	-25724.8
Tnfaip8	Tnfaip8-001	0.0199154	1.97407	1.97407
Tnfaip8l3	Tnfaip8l3-201	0.0151961	0.801439	-1.24776
Tnfrsf11b	Tnfrsf11b-201	0.0210704	1.36639	1.36639
Tnfsf13os	Tnfsf13os-001	0.00279726	7.05189	7.05189
Tnip1	Tnip1-011	0.0382862	7.32345	7.32345
Tnip2	Tnip2-007	0.0200904	0.61013	-1.63899
Tnk1	Tnk1-002	0.0473604	24.9798	24.9798
Tnks1bp1	Tnks1bp1-003	0.0371456	0.823228	-1.21473
Tnmd	Tnmd-001	0.0277309	5.83661	5.83661
Tnni3	Tnni3-007	0.00469156	7.35427	7.35427
Tnr	Tnr-001	0.0206568	0.883005	-1.1325
Tnrc6a	Tnrc6a-005	0.00133472	1.84189	1.84189
Tnrc6c	Tnrc6c-001	0.0204169	0.749684	-1.33389
Tns1	Tns1-201	0.0143584	1.47763	1.47763
Tns1	Tns1-008	0.0439195	1.55652	1.55652
Top3b	Top3b-005	0.00509584	0.091822	-10.8906
Topbp1	Topbp1-003	8.52017e-05	0.00752711	-132.853
Topbp1	Topbp1-001	0.0205209	0.911584	-1.09699
Tor2a	Tor2a-001	0.046839	0.845333	-1.18297
Tpd52	Tpd52-011	0.0312419	1.71366	1.71366
Tpd52l2	Tpd52l2-011	0.00746799	7.43699e-10	-1.34463e+09
Tpd52l2	Tpd52l2-001	0.0438655	2.00992	2.00992
Tpra1	Tpra1-013	0.0481366	2.33304	2.33304
Tprkb	Tprkb-002	0.0382788	0.564921	-1.77016
Tpst1	Tpst1-002	0.0371428	0.75297	-1.32807
Tpst1	Tpst1-001	0.00249302	1.40903	1.40903

Tpt1	Tpt1-003	0.0326427	0.37252	-2.68442
Tra2b	Tra2b-002	0.0207813	1.37351	1.37351
Traf1	Traf1-006	0.0482096	5.96023	5.96023
Traf2	Traf2-003	0.0491588	1.41773	1.41773
Traf3ip1	Traf3ip1-001	0.0140152	1.30606	1.30606
Traf3ip3	Traf3ip3-014	0.0463413	9.80917	9.80917
Traf7	Traf7-011	0.0343524	0.101518	-9.85048
Traf7	Traf7-019	0.0406263	3.67284	3.67284
Traip	Traip-003	0.0466618	1.87671	1.87671
Trak2	Trak2-001	0.0383219	1.08324	1.08324
Tram2	Tram2-001	0.0302254	1.48983	1.48983
Trappc1	Trappc1-002	0.0432053	0.825639	-1.21118
Trappc6a	Trappc6a-005	0.00482493	0.542652	-1.8428
Trhr	Trhr-201	0.028033	0.709925	-1.4086
Trhr2	Trhr2-001	0.0282193	5.66293	5.66293
Trib2	Trib2-001	0.0269922	1.25432	1.25432
Trib3	Trib3-001	0.0309891	1.75952	1.75952
Trim33	Trim33-007	0.0293669	0.346954	-2.88223
Trim43b	Trim43b-201	0.0244896	0.653719	-1.52971
Trim44	Trim44-001	0.0216732	0.967044	-1.03408
Trim46	Trim46-006	0.0329402	298.235	298.235
Trim56	Trim56-001	0.0276065	1.26779	1.26779
Triobp	Triobp-008	0.000601918	360.541	360.541
Trip12	Trip12-011	0.00350876	5.558	5.558
Trmt1	Trmt1-030	0.00724751	0.0560317	-17.847
Trmt10a	Trmt10a-005	0.0144414	0.29088	-3.43784
Trmt2a	Trmt2a-010	0.00104797	0.354227	-2.82305
Trmt6	Trmt6-003	0.0152891	0.440662	-2.26931
Trmt61a	Trmt61a-202	0.0409808	2.83463	2.83463
Trnau1ap	Trnau1ap-002	0.0276756	0.759004	-1.31752
Tro	Tro-006	0.0329445	33.7799	33.7799
Trp53inp2	Trp53inp2-001	0.0114975	1.12384	1.12384
Trpc4	Trpc4-001	0.0245052	0.697295	-1.43411
Trpm3	Trpm3-205	0.0140713	14.6134	14.6134
Trpm6	Trpm6-201	0.0405739	1.7619	1.7619
Trpt1	Trpt1-201	0.0435524	1.23645	1.23645
Trpv4	Trpv4-005	0.00554758	40.622	40.622
Tsacc	Tsacc-001	0.0363131	0.213605	-4.68154
Tsen2	Tsen2-002	0.0278891	0.589165	-1.69732
Tsg101	Tsg101-003	0.0269748	6.6382	6.6382
Tshz1	Tshz1-001	0.0332207	1.35198	1.35198
Tspan13	Tspan13-001	0.0403097	0.872157	-1.14658
Tspan31	Tspan31-201	0.0131877	1.15308	1.15308

Tspan32	Tspan32-003	0.0316198	0.00667577	-149.795
Tstd2	Tstd2-006	0.0417213	2.65151	2.65151
Ttc13	Ttc13-001	0.000349238	0.429269	-2.32954
Ttc13	Ttc13-003	0.0181257	1.39985	1.39985
Ttc14	Ttc14-003	0.0101357	8.8699e-10	-1.12741e+09
Ttc14	Ttc14-011	0.0405252	4.27797	4.27797
Ttc16	Ttc16-001	0.0413414	0.0111997	-89.2878
Ttc3	Ttc3-001	0.0352614	0.945531	-1.05761
Ttc39a	Ttc39a-001	0.0350597	1.93342	1.93342
Ttc4	Ttc4-002	0.00360417	2.51141	2.51141
Ttc4	Ttc4-008	0.0496357	85.5861	85.5861
Ttc9c	Ttc9c-003	0.0379987	0.592362	-1.68816
Ttll11	Ttll11-201	0.0349574	0.751088	-1.3314
Ttll12	Ttll12-002	0.00222377	2.12167	2.12167
Ttll2	Ttll2-201	0.000342157	0.260369	-3.84071
Ttll5	Ttll5-012	0.0312068	0.354241	-2.82294
Ttll5	Ttll5-013	0.0370457	2.19385	2.19385
Ttr	Ttr-201	0.0364316	30.5773	30.5773
Ttyh3	Ttyh3-002	0.0190285	1.27021	1.27021
Tuba4a	Tuba4a-003	0.0141263	1.16686	1.16686
Tubgcp2	Tubgcp2-201	0.0318515	0.909411	-1.09961
Tug1	Tug1-003	0.0464925	0.763719	-1.30938
Tug1	Tug1-001	0.0165394	1.36111	1.36111
Tulp3	Tulp3-003	0.0228458	0.244807	-4.08485
Tulp4	Tulp4-003	0.00667848	1.88942	1.88942
Twistnb	Twistnb-201	0.0415702	0.852759	-1.17266
Txndc11	Txndc11-003	0.010181	2.08582	2.08582
Txndc5	Txndc5-002	0.000405236	2.78629	2.78629
Tyro3	Tyro3-007	0.0498691	0.18508	-5.40308
Tyro3	Tyro3-002	0.00795828	1.22106	1.22106
Tyro3	Tyro3-003	0.0189514	1.64302	1.64302
Tyrp1	Tyrp1-003	0.0428134	45307.7	45307.7
Tysnd1	Tysnd1-201	0.0367023	1.13698	1.13698
Uba2	Uba2-002	0.00571676	2.97122	2.97122
Uba3	Uba3-004	0.0308614	2.4209	2.4209
Uba52	Uba52-003	0.0265786	0.686601	-1.45645
Uba7	Uba7-004	0.0158163	0.0976865	-10.2368
Ubap2l	Ubap2l-008	0.0414888	0.365328	-2.73727
Ube2b	Ube2b-003	0.0210871	1.39877	1.39877
Ube2d3	Ube2d3-010	0.0277418	0.236856	-4.22198
Ube2d3	Ube2d3-002	0.0495925	1.16152	1.16152
Ube2e3	Ube2e3-004	0.00985555	0.0143566	-69.6544
Ube2k	Ube2k-008	0.0421007	3.46358	3.46358

Ube2l6	Ube2l6-003	0.0306336	1.30526e-08	-7.66132e+07
Ube2m	Ube2m-002	0.0270752	0.187949	-5.3206
Ube2q1	Ube2q1-002	0.036214	0.936457	-1.06785
Ube2u	Ube2u-001	0.0444804	0.0786978	-12.7068
Ube2v1	Ube2v1-007	0.0208	3.61842	3.61842
Ube3a	Ube3a-016	0.001498	4.42433	4.42433
Ube3a	Ube3a-003	0.0298129	1.50021	1.50021
Ube3b	Ube3b-003	0.0248166	3.72386	3.72386
Ube4a	Ube4a-002	0.026436	1.74675	1.74675
Ube4b	Ube4b-002	0.0388078	1.44074	1.44074
Ube4bos2	Ube4bos2-001	0.00604111	7.96253	7.96253
Ubfd1	Ubfd1-001	0.024438	1.04574	1.04574
Ubiad1	Ubiad1-001	0.0277586	1.13499	1.13499
Ubl3	Ubl3-002	0.0198868	1.68285	1.68285
Ubn2	Ubn2-008	0.0168179	0.0129788	-77.049
Ubr3	Ubr3-008	0.0319692	0.621086	-1.61008
Ubxn10	Ubxn10-002	0.0341866	2.62048	2.62048
Ubxn6	Ubxn6-004	0.0109577	1.27476	1.27476
Uchl5	Uchl5-008	0.0352475	0.448587	-2.22922
Uckl1	Uckl1-016	0.0099672	0.165895	-6.02791
Uckl1	Uckl1-015	0.0107356	5.71566	5.71566
Ufd1l	Ufd1l-201	0.0289745	0.923206	-1.08318
Ufsp2	Ufsp2-001	0.00060233	0.630951	-1.58491
Ugdh	Ugdh-003	0.0284675	5.01027	5.01027
Uggt2	Uggt2-006	0.0357678	2.88098	2.88098
Uhrf1bp1l	Uhrf1bp1l-201	0.0280478	0.927278	-1.07843
Ulk1	Ulk1-011	0.00153983	5313.2	5313.2
Umad1	Umad1-006	0.00138302	6.22956	6.22956
Umodl1	Umodl1-201	0.00821146	0.0437687	-22.8474
Unc13b	Unc13b-013	0.0207864	0.601413	-1.66275
Unc45a	Unc45a-007	0.0252414	0.550408	-1.81683
Unk	Unk-003	0.0362438	1.98209	1.98209
Upp2	Upp2-001	0.0125315	0.644923	-1.55057
Uqcc1	Uqcc1-011	0.0370811	0.164377	-6.08359
Uqcc1	Uqcc1-012	0.0267217	1.86969	1.86969
Urb1	Urb1-001	0.0485733	0.000669482	-1493.69
Uri1	Uri1-001	0.0110451	0.735027	-1.36049
Urod	Urod-005	0.0441131	8.66029	8.66029
Uros	Uros-002	0.0399861	2.42608	2.42608
Use1	Use1-010	0.026502	0.547048	-1.82799
Use1	Use1-004	0.0407888	3.94529	3.94529
Usp10	Usp10-002	0.047735	0.269767	-3.7069
Usp19	Usp19-001	0.0106268	1.55919	1.55919

Usp19	Usp19-007	0.0334086	1.864e+09	1.864e+09
Usp21	Usp21-002	0.0348783	1.96399	1.96399
Usp32	Usp32-003	0.0121664	1.31535	1.31535
Usp36	Usp36-003	0.00976049	3.9224	3.9224
Usp36	Usp36-005	0.00212509	3.74197	3.74197
Usp50	Usp50-003	0.0485194	0.512935	-1.94957
Usp53	Usp53-003	0.000717303	0.0574756	-17.3987
Usp7	Usp7-013	0.0114493	4.32741	4.32741
Uspl1	Uspl1-011	0.0263631	1.36567	1.36567
Utp14a	Utp14a-002	0.0472043	1.40081	1.40081
Utp14b	Utp14b-002	0.0265056	0.30586	-3.26947
Utp15	Utp15-201	0.01576	0.925013	-1.08107
Utp18	Utp18-001	0.000478184	0.764378	-1.30825
Utp23	Utp23-004	0.0459663	1.03368e+07	1.03368e+07
Utrn	Utrn-201	0.0331839	1.22447	1.22447
Vamp4	Vamp4-007	0.0325978	3.59157e-06	-278429
Vamp5	Vamp5-001	0.0454423	0.38051	-2.62805
Vamp5	Vamp5-003	0.0203245	2.60289	2.60289
Vangl2	Vangl2-001	0.0333678	0.851707	-1.17411
Vars	Vars-012	0.0348421	0.00230724	-433.418
Vars2	Vars2-003	0.010797	1.88973	1.88973
Vat1l	Vat1l-001	0.00911914	2.97467	2.97467
Vcan	Vcan-003	0.00255166	2.77456	2.77456
Vcpip1	Vcpip1-001	0.0176577	0.929385	-1.07598
Vegfa	Vegfa-001	0.0113534	1.29813	1.29813
Vezf1	Vezf1-001	0.0150129	1.20144	1.20144
Vezt	Vezt-011	0.0124906	0.00598247	-167.155
Vezt	Vezt-002	0.0372142	1.36366	1.36366
Vldlr	Vldlr-001	0.00728839	1.08358	1.08358
Vma21	Vma21-001	0.0361741	0.840614	-1.18961
Vma21	Vma21-002	0.00715497	2.65328	2.65328
Vmn1r-ps128	Vmn1r-ps128-001	0.0270117	1.78396	1.78396
Vpreb2	Vpreb2-201	0.018026	0.000113576	-8804.7
Vps25	Vps25-007	0.0331639	0.244437	-4.09103
Vps29	Vps29-007	0.0458233	1.71184	1.71184
Vps33a	Vps33a-002	0.0389523	37.2842	37.2842
Vps39	Vps39-009	0.0302785	0.160779	-6.21971
Vps52	Vps52-007	0.0245326	0.547668	-1.82592
Vps52	Vps52-001	0.0487243	1.10353	1.10353
Vps53	Vps53-011	0.00410004	0.10489	-9.53379
Vps53	Vps53-001	0.0233116	0.822635	-1.21561
Vps9d1	Vps9d1-004	0.0425051	1.48554	1.48554
Vps9d1	Vps9d1-008	0.0246693	1.64608	1.64608

Vsig1	Vsig1-002	0.0306227	0.0489477	-20.4299
Vstm2a	Vstm2a-002	0.0204756	0.873254	-1.14514
Vta1	Vta1-006	0.0419392	0.643979	-1.55285
Vti1b	Vti1b-004	0.0224477	1.32187	1.32187
Vwa5b1	Vwa5b1-005	0.0472489	6.27324	6.27324
Vwc2	Vwc2-201	0.047693	1.48896	1.48896
Vwf	Vwf-011	0.00742517	3.42836	3.42836
Was	Was-001	0.0239348	1.49158	1.49158
Wash1	Wash1-202	0.025358	0.763277	-1.31014
Wash1	Wash1-201	0.0284665	1.43068	1.43068
Wbscr17	Wbscr17-001	0.0313138	0.863254	-1.15841
Wbscr22	Wbscr22-009	0.0382949	2.68046	2.68046
Wdr1	Wdr1-008	0.03127	0.000102868	-9721.24
Wdr26	Wdr26-005	0.0415118	2.27605	2.27605
Wdr31	Wdr31-001	0.0487625	0.491239	-2.03567
Wdr33	Wdr33-201	0.0290128	1.08369	1.08369
Wdr37	Wdr37-201	0.0111563	0.75759	-1.31997
Wdr4	Wdr4-004	0.0129063	5.23327	5.23327
Wdr44	Wdr44-004	0.0478387	0.181178	-5.51945
Wdr45	Wdr45-007	0.0364477	8.59339	8.59339
Wdr49	Wdr49-001	0.0241326	2.68558	2.68558
Wdr5	Wdr5-002	0.000695234	8.22792e+10	8.22792e+10
Wdr7	Wdr7-201	0.000501686	0.884811	-1.13019
Wdr70	Wdr70-201	0.00230882	0.808309	-1.23715
Wdr73	Wdr73-007	0.0179384	4.52692	4.52692
Wdr78	Wdr78-005	0.0317979	1.91324	1.91324
Wdsub1	Wdsub1-009	0.0221597	2.84086	2.84086
Wfdc2	Wfdc2-004	0.00645163	15.9214	15.9214
Wfikkn1	Wfikkn1-001	0.0142083	0.226911	-4.40701
Whrn	Whrn-009	0.037695	1.87639	1.87639
Whrn	Whrn-010	0.014532	3.24985	3.24985
Whsc1l1	Whsc1l1-008	0.0204806	0.674036	-1.4836
Wiz	Wiz-009	0.00664497	2.55951	2.55951
Wls	Wls-001	0.00456157	1.52877	1.52877
Wnk1	Wnk1-201	0.00737514	0.617416	-1.61965
Wnk1	Wnk1-005	0.0227277	2.19784	2.19784
Wnk2	Wnk2-203	0.0247479	0.527476	-1.89582
Wnk2	Wnk2-204	0.0247282	0.454227	-2.20154
Wnk2	Wnk2-202	0.00801416	1.42402	1.42402
Wnk3	Wnk3-002	0.0437273	0.388576	-2.5735
Wnk4	Wnk4-005	0.0426876	16.436	16.436
Wnt10b	Wnt10b-202	0.0395989	0.0295604	-33.829
Wnt3	Wnt3-001	0.0365443	0.704533	-1.41938

Wnt9a	Wnt9a-201	0.0231664	0.591511	-1.69059
Xpnpep2	Xpnpep2-002	0.0378439	0.222407	-4.49627
Xrcc1	Xrcc1-010	0.0391135	0.0473445	-21.1218
Xrcc1	Xrcc1-001	0.0372572	0.0361124	-27.6913
Xrcc6bp1	Xrcc6bp1-202	0.0356077	1.24101	1.24101
Ybey	Ybey-201	0.0191289	0.813181	-1.22974
Ybx2	Ybx2-002	0.0242123	0.180524	-5.53942
Yipf1	Yipf1-009	0.0102483	0.00254514	-392.905
Yipf3	Yipf3-006	0.0173502	13.3797	13.3797
Yipf3	Yipf3-003	0.00760682	2.43243	2.43243
Yod1	Yod1-001	0.021366	0.884998	-1.12995
Ypel1	Ypel1-004	0.024577	0.583558	-1.71363
Ywhaq	Ywhaq-003	0.0253445	1.2885	1.2885
Zbtb11	Zbtb11-003	0.0215991	2.80317	2.80317
Zbtb17	Zbtb17-003	0.0426547	0.589282	-1.69698
Zbtb17	Zbtb17-001	0.0188665	1.21146	1.21146
Zbtb18	Zbtb18-009	0.0124755	0.803235	-1.24497
Zbtb33	Zbtb33-201	0.0449711	0.922045	-1.08455
Zbtb38	Zbtb38-005	0.0324828	1.97486	1.97486
Zbtb48	Zbtb48-001	0.033156	0.0581192	-17.206
Zbtb49	Zbtb49-010	0.0415651	0.420598	-2.37757
Zbtb5	Zbtb5-002	0.0385851	1.53812	1.53812
Zbtb7a	Zbtb7a-001	0.014671	1.13	1.13
Zc2hc1c	Zc2hc1c-001	0.0425466	1.33917	1.33917
Zc3h12b	Zc3h12b-002	0.0353568	0.899314	-1.11196
Zc3hav1l	Zc3hav1l-001	0.00146053	0.851254	-1.17474
Zc3hc1	Zc3hc1-002	0.0377655	0.664772	-1.50428
Zc3hc1	Zc3hc1-004	0.0118638	2.62957	2.62957
Zc3hc1	Zc3hc1-009	0.0463275	3.89513	3.89513
Zcchc9-ps	Zcchc9-ps-001	0.0358262	0.176297	-5.67225
Zcrb1	Zcrb1-008	0.0309079	0.632407	-1.58126
Zdhhc15	Zdhhc15-001	0.026419	0.84359	-1.18541
Zeb2	Zeb2-001	0.00989178	0.759359	-1.3169
Zeb2	Zeb2-019	0.0304011	41.1714	41.1714
Zer1	Zer1-002	0.0374583	0.81242	-1.23089
Zfand1	Zfand1-005	0.00919609	0.610489	-1.63803
Zfand5	Zfand5-001	0.0483054	0.922139	-1.08444
Zfp11	Zfp11-001	0.0107528	0.83334	-1.19999
Zfp12	Zfp12-003	0.0484344	1.4052	1.4052
Zfp133-ps	Zfp133-ps-004	0.0339565	3.616	3.616
Zfp180	Zfp180-001	0.0255207	1.51932	1.51932
Zfp191	Zfp191-001	0.0268565	1.08541	1.08541
Zfp260	Zfp260-002	0.0192843	0.856106	-1.16808

Zfp266	Zfp266-201	0.0341456	0.234677	-4.26117
Zfp276	Zfp276-006	0.0361669	0.436065	-2.29324
Zfp280c	Zfp280c-005	0.00102019	0.114514	-8.73257
Zfp281	Zfp281-002	0.00873201	0.823835	-1.21384
Zfp287	Zfp287-004	0.0378438	0.438598	-2.27999
Zfp30	Zfp30-003	0.039866	0.618621	-1.6165
Zfp317	Zfp317-201	0.0489373	0.867374	-1.1529
Zfp322a	Zfp322a-004	0.0134104	2.29478	2.29478
Zfp346	Zfp346-006	0.00320149	4.14878	4.14878
Zfp354a	Zfp354a-002	0.0213801	4.72861	4.72861
Zfp384	Zfp384-004	0.0440897	0.245407	-4.07486
Zfp384	Zfp384-014	0.0311102	228.812	228.812
Zfp407	Zfp407-005	0.00330388	0.25794	-3.87688
Zfp414	Zfp414-002	0.0385799	0.591529	-1.69053
Zfp423	Zfp423-006	0.0288604	0.133508	-7.49019
Zfp449	Zfp449-001	0.0315048	1.27229	1.27229
Zfp451	Zfp451-007	0.0261463	15.6521	15.6521
Zfp454	Zfp454-001	0.00186282	2.97503	2.97503
Zfp456	Zfp456-001	0.0102897	1.47103	1.47103
Zfp46	Zfp46-005	0.0312235	0.5716	-1.74947
Zfp46	Zfp46-002	0.0420989	3.03895e+07	3.03895e+07
Zfp467	Zfp467-005	0.0273475	0.34238	-2.92073
Zfp467	Zfp467-001	0.0281203	1.89564	1.89564
Zfp503	Zfp503-001	0.0474779	1.31373	1.31373
Zfp512	Zfp512-007	0.00878089	0.740333	-1.35074
Zfp516	Zfp516-202	0.0227203	1.34574	1.34574
Zfp518a	Zfp518a-201	0.0429977	0.818583	-1.22162
Zfp52	Zfp52-201	0.00373124	1.33866	1.33866
Zfp532	Zfp532-001	0.0497455	0.459695	-2.17536
Zfp563	Zfp563-002	0.0414508	4.41281e+07	4.41281e+07
Zfp568	Zfp568-001	0.0476051	1.30714	1.30714
Zfp574	Zfp574-002	0.045393	1.12068	1.12068
Zfp58	Zfp58-004	0.0442891	10.2074	10.2074
Zfp598	Zfp598-201	0.00273364	1.22847	1.22847
Zfp608	Zfp608-201	0.000393283	0.750779	-1.33195
Zfp638	Zfp638-017	0.0494009	5.05984	5.05984
Zfp644	Zfp644-201	0.0482815	0.822509	-1.21579
Zfp672	Zfp672-001	0.0496803	0.781865	-1.27899
Zfp688	Zfp688-002	0.00570142	0.495565	-2.0179
Zfp69	Zfp69-201	0.00781265	0.165825	-6.03046
Zfp697	Zfp697-001	0.0173651	0.741609	-1.34842
Zfp704	Zfp704-003	0.0244921	2.59973	2.59973
Zfp707	Zfp707-001	0.0286355	3.50291	3.50291

Zfp710	Zfp710-004	0.0472051	2.27263	2.27263
Zfp759	Zfp759-201	0.0151767	0.824421	-1.21297
Zfp770	Zfp770-001	0.00622012	0.892907	-1.11994
Zfp804b	Zfp804b-001	0.0210918	0.242377	-4.1258
Zfp804b	Zfp804b-201	0.000311318	6.80011	6.80011
Zfp810	Zfp810-201	0.0334272	0.848635	-1.17836
Zfp85	Zfp85-201	0.0243994	0.671042	-1.49022
Zfp868	Zfp868-001	0.00871461	1.2678	1.2678
Zfp9	Zfp9-001	0.00400173	0.910499	-1.0983
Zfp91	Zfp91-003	0.036556	15.5956	15.5956
Zfp93	Zfp93-001	0.0241081	0.605002	-1.65289
Zfp932	Zfp932-005	0.000589797	16.6381	16.6381
Zfp940	Zfp940-002	0.0182397	0.705882	-1.41667
Zfp945	Zfp945-201	0.0469187	0.834782	-1.19792
Zfp945	Zfp945-001	0.029207	0.511577	-1.95474
Zfp945	Zfp945-002	0.0314849	1482.1	1482.1
Zfp946	Zfp946-002	0.0300361	2.55707	2.55707
Zfp950	Zfp950-007	0.0127939	0.143221	-6.98224
Zfp950	Zfp950-202	0.036725	0.00653858	-152.938
Zfpm2	Zfpm2-201	0.00428239	0.784478	-1.27473
Zfr	Zfr-001	0.013451	0.921986	-1.08462
Zfr	Zfr-003	0.0433381	0.419929	-2.38135
Zfr	Zfr-002	0.046624	1.70525	1.70525
Zfyve1	Zfyve1-201	0.0411557	1.1109	1.1109
Zfyve16	Zfyve16-001	0.0114935	1.28163	1.28163
Zfyve28	Zfyve28-003	0.0360637	0.219064	-4.56487
Zfyve28	Zfyve28-002	0.0404861	3.6542	3.6542
Zfyve9	Zfyve9-003	0.0297318	1.21799	1.21799
Zgpat	Zgpat-003	0.0240406	1.36388	1.36388
Zgpat	Zgpat-005	0.0399144	210.213	210.213
Zhx3	Zhx3-005	0.0358514	133.687	133.687
Zic1	Zic1-002	0.0390546	2.57411	2.57411
Zic4	Zic4-005	0.00590281	2.91403	2.91403
Zmpste24	Zmpste24-002	0.0203359	1.69953	1.69953
Zmym1	Zmym1-201	0.00285646	2.12318	2.12318
Zmym3	Zmym3-008	0.00659965	6.97167	6.97167
Znf512b	Znf512b-005	0.0408533	0.404204	-2.474
Znhit1	Znhit1-001	0.0439176	0.446213	-2.24108
Znhit3	Znhit3-002	0.0446395	0.565257	-1.76911
Znhit3	Znhit3-005	0.00448781	2.19273	2.19273
Znhit6	Znhit6-005	0.00811622	16.8918	16.8918
Zpld1	Zpld1-201	0.0357851	0.344617	-2.90177
Zranb2	Zranb2-006	0.0242919	9.9983	9.9983

Zscan10	Zscan10-005	0.0445897	27.343	27.343
Zscan29	Zscan29-002	0.0138672	2.0852	2.0852

RNA-Seq expression analysis of Neuro2a cells

Gene Symbol	Transcript	p-value	Ratio (132 vs. SCR)	Fold Change
0610009B22Rik	0610009B22Rik-001	0.0402167	1.28349	1.28349
0610009L18Rik	0610009L18Rik-001	0.0035234	1.94342	1.94342
0610009O20Rik	0610009O20Rik-201	0.000454318	1.22975	1.22975
0610010F05Rik	0610010F05Rik-001	0.00175875	3.3169	3.3169
0610010K14Rik	0610010K14Rik-006	0.0107714	2.18738	2.18738
0610011F06Rik	0610011F06Rik-001	0.00418816	0.453423	-2.20545
0610038B21Rik	0610038B21Rik-002	0.041643	2.41719	2.41719
1110001J03Rik	1110001J03Rik-001	0.0201975	1.28706	1.28706
1110004F10Rik	1110004F10Rik-006	0.0382005	0.421668	-2.37153
1110004F10Rik	1110004F10Rik-001	0.0237741	1.36855	1.36855
1110012L19Rik	1110012L19Rik-001	1.21282e-05	1.43291	1.43291
1110020A21Rik	1110020A21Rik-201	0.0133021	2.35792	2.35792
1200014J11Rik	1200014J11Rik-001	0.000366047	1.29834	1.29834
1500009C09Rik	1500009C09Rik-001	0.00975786	2.36472	2.36472
1500009L16Rik	1500009L16Rik-201	0.0448158	0.636483	-1.57113
1500011K16Rik	1500011K16Rik-001	0.00622297	1.26065	1.26065
1500015A07Rik	1500015A07Rik-002	0.0247761	1.7912	1.7912
1500017E21Rik	1500017E21Rik-004	0.0271182	862.91	862.91
1500017E21Rik	1500017E21Rik-003	0.0245372	909.964	909.964
1500026H17Rik	1500026H17Rik-201	0.000877092	8.08585	8.08585
1600002D24Rik	1600002D24Rik-002	0.0108698	10.3323	10.3323
1600014C23Rik	1600014C23Rik-201	0.0191334	1.85951	1.85951
1600023N17Rik	1600023N17Rik-001	0.0403409	2.78159	2.78159
1600029O15Rik	1600029O15Rik-002	0.00123947	1.83662	1.83662
1700001L19Rik	1700001L19Rik-201	0.0408555	2.67483	2.67483
1700001P01Rik	1700001P01Rik-001	0.0110431	1.67774	1.67774
1700003F12Rik	1700003F12Rik-003	0.0325633	0.330731	-3.02361
1700013F07Rik	1700013F07Rik-001	0.0262438	11.2581	11.2581
1700019G17Rik	1700019G17Rik-001	0.00218278	1.98297	1.98297
1700020D05Rik	1700020D05Rik-001	0.0468749	1.5721	1.5721
1700020I14Rik	1700020I14Rik-002	0.00158063	2.25627	2.25627
1700020L24Rik	1700020L24Rik-002	0.0431092	3.97096	3.97096
1700021F05Rik	1700021F05Rik-201	8.26714e-06	1.40385	1.40385
1700025G04Rik	1700025G04Rik-004	0.0444531	0.46122	-2.16816
1700025G04Rik	1700025G04Rik-001	0.000517349	0.720067	-1.38876
1700025N23Rik	1700025N23Rik-001	0.00037333	1.39203	1.39203

1700028I16Rik	1700028I16Rik-001	0.0187012	1.63347	1.63347
1700028I16Rik	1700028I16Rik-201	0.0187013	1.63347	1.63347
1700029I15Rik	1700029I15Rik-002	0.00144678	0.432375	-2.31281
1700029J07Rik	1700029J07Rik-002	0.0050067	1.54047	1.54047
1700030J22Rik	1700030J22Rik-201	0.0222297	1.37315	1.37315
1700034P13Rik	1700034P13Rik-001	0.0200423	2.32441	2.32441
1700037C18Rik	1700037C18Rik-001	0.0327911	2.25583	2.25583
1700040D17Rik	1700040D17Rik-001	0.0187954	2.61474	2.61474
1700066M21Rik	1700066M21Rik-001	0.0390421	1.17483	1.17483
1700069B07Rik	1700069B07Rik-201	0.0441292	0.654692	-1.52744
1700086P04Rik	1700086P04Rik-001	1.47083e-06	2.26894	2.26894
1700101I11Rik	1700101I11Rik-002	0.0158652	5.80803e-09	-1.72175e+08
1700110I01Rik	1700110I01Rik-001	0.0296951	2.02744	2.02744
1700124L16Rik	1700124L16Rik-002	0.0407923	1.78463	1.78463
1810010H24Rik	1810010H24Rik-201	0.0497741	6.67113	6.67113
1810013L24Rik	1810013L24Rik-201	0.00604544	1.10594	1.10594
1810019D21Rik	1810019D21Rik-003	0.0056361	3.6956	3.6956
1810044D09Rik	1810044D09Rik-001	0.00813153	2.09134	2.09134
1810058I24Rik	1810058I24Rik-001	0.0119052	0.753848	-1.32653
1810062O18Rik	1810062O18Rik-003	0.0492808	2.3419	2.3419
2010310C07Rik	2010310C07Rik-002	0.0313251	3.08851	3.08851
2210013O21Rik	2210013O21Rik-001	0.00857364	1.34224	1.34224
2210016L21Rik	2210016L21Rik-001	0.00612788	1.28454	1.28454
2210018M11Rik	2210018M11Rik-001	0.000658659	0.565714	-1.76768
2210018M11Rik	2210018M11Rik-004	0.028261	4.71905	4.71905
2210404O09Rik	2210404O09Rik-201	0.0108191	1.60766	1.60766
2300009A05Rik	2300009A05Rik-002	0.0167909	1.50216	1.50216
2300009A05Rik	2300009A05Rik-001	0.00168791	1.56728	1.56728
2310009A05Rik	2310009A05Rik-001	0.0325473	1.53375	1.53375
2310009A05Rik	2310009A05Rik-002	0.0343251	2.4348	2.4348
2310030G06Rik	2310030G06Rik-201	0.00105338	0.584798	-1.70999
2310034O05Rik	2310034O05Rik-002	0.0385712	0.0440156	-22.7192
2310035C23Rik	2310035C23Rik-007	0.00066627	0.576123	-1.73574
2310035C23Rik	2310035C23Rik-004	0.0229373	1.37802	1.37802
2310039H08Rik	2310039H08Rik-201	0.0088791	1.95165	1.95165
2310039L15Rik	2310039L15Rik-001	0.011523	1.25698	1.25698
2310040G07Rik	2310040G07Rik-001	0.0111085	0.189078	-5.28881
2310058D17Rik	2310058D17Rik-001	0.00653976	1.73526	1.73526
2410002F23Rik	2410002F23Rik-011	0.0453983	7.80564	7.80564
2410002F23Rik	2410002F23Rik-014	0.00801486	37.284	37.284
2510002D24Rik	2510002D24Rik-004	0.0437521	2.01573	2.01573
2510046G10Rik	2510046G10Rik-001	0.0165732	2.66678	2.66678
2610001J05Rik	2610001J05Rik-003	0.0242322	0.478423	-2.0902

2610002M06Rik	2610002M06Rik-001	0.0123483	0.712684	-1.40315
2610005L07Rik	2610005L07Rik-002	0.000109565	1.49144	1.49144
2610005L07Rik	2610005L07Rik-001	2.26404e-05	1.51774	1.51774
2610008E11Rik	2610008E11Rik-001	0.00111985	1.76269	1.76269
2610015P09Rik	2610015P09Rik-201	5.77951e-05	2.01775	2.01775
2610020C07Rik	2610020C07Rik-201	0.00721867	1.43591	1.43591
2610020H08Rik	2610020H08Rik-001	0.018675	4.13479	4.13479
2610020H08Rik	2610020H08Rik-008	0.0297717	4.99388	4.99388
2610021A01Rik	2610021A01Rik-001	0.0101316	1.26199	1.26199
2610035F20Rik	2610035F20Rik-002	0.0154055	2.00303	2.00303
2610037D02Rik	2610037D02Rik-001	0.00117144	1.66421	1.66421
2610203C22Rik	2610203C22Rik-001	0.040414	0.37704	-2.65224
2700029L08Rik	2700029L08Rik-001	0.0052179	0.381598	-2.62056
2700029M09Rik	2700029M09Rik-001	0.00431223	1.2038	1.2038
2700029M09Rik	2700029M09Rik-005	0.0225468	2.59686	2.59686
2700033N17Rik	2700033N17Rik-001	0.00711297	1.62744	1.62744
2700046G09Rik	2700046G09Rik-201	0.00282544	2.56627	2.56627
2700060E02Rik	2700060E02Rik-201	0.0495092	1.14191	1.14191
2700062C07Rik	2700062C07Rik-201	0.0110346	1.4195	1.4195
2700097O09Rik	2700097O09Rik-001	0.00998168	1.54855	1.54855
2700099C18Rik	2700099C18Rik-004	0.0300959	0.52714	-1.89703
2700099C18Rik	2700099C18Rik-002	0.0281002	1.28697	1.28697
2810001G20Rik	2810001G20Rik-001	0.000386818	1.62745	1.62745
2810002D19Rik	2810002D19Rik-001	0.0498194	1.36189	1.36189
2810006K23Rik	2810006K23Rik-001	0.0131342	1.12568	1.12568
2810013P06Rik	2810013P06Rik-001	0.0299175	1.7898	1.7898
2810025M15Rik	2810025M15Rik-002	0.00636159	1.23442	1.23442
2810403D21Rik	2810403D21Rik-003	0.0142092	2.49743	2.49743
2810405F17Rik	2810405F17Rik-001	0.00772547	1.83243	1.83243
2810408I11Rik	2810408I11Rik-001	0.00406341	1.59352	1.59352
2810414N06Rik	2810414N06Rik-001	0.00253989	2.05461	2.05461
2810429I04Rik	2810429I04Rik-002	0.0343468	2.54836	2.54836
2900026A02Rik	2900026A02Rik-001	0.00781706	0.617495	-1.61945
2900052L18Rik	2900052L18Rik-001	0.0216337	1.76994	1.76994
2900076A07Rik	2900076A07Rik-007	0.0436235	1.51438	1.51438
2900076A07Rik	2900076A07Rik-003	0.0194252	2.74069	2.74069
2900076A07Rik	2900076A07Rik-005	0.0179153	5.79751	5.79751
2900093K20Rik	2900093K20Rik-001	0.0338635	1.92613	1.92613
3010026O09Rik	3010026O09Rik-001	0.00415747	1.46396	1.46396
3110002H16Rik	3110002H16Rik-001	0.0027638	0.530949	-1.88342
3110002H16Rik	3110002H16Rik-003	0.0108697	2.38119	2.38119
3110021N24Rik	3110021N24Rik-201	0.0250082	3.23486	3.23486
3110040N11Rik	3110040N11Rik-003	0.0482147	1.41313	1.41313

3110045C21Rik	3110045C21Rik-001	0.0354704	22.0067	22.0067
3110052M02Rik	3110052M02Rik-201	0.039495	0.778627	-1.28431
3110056K07Rik	3110056K07Rik-002	0.0220655	1.60404	1.60404
3110067C02Rik	3110067C02Rik-001	0.0215675	1.73714	1.73714
3110083C13Rik	3110083C13Rik-201	0.0123472	2.12244	2.12244
3300002I08Rik	3300002I08Rik-002	0.00329019	0.433308	-2.30783
3300002I08Rik	3300002I08Rik-003	0.0128083	4.37605	4.37605
3930402G23Rik	3930402G23Rik-001	0.0469503	0.564508	-1.77145
4632404H12Rik	4632404H12Rik-002	0.0246564	1.63074	1.63074
4632427E13Rik	4632427E13Rik-201	0.0233722	2.97358	2.97358
4632432E15Rik	4632432E15Rik-001	0.00882718	1.39098	1.39098
4833427F10Rik	4833427F10Rik-001	0.0451242	2.32747	2.32747
4833439L19Rik	4833439L19Rik-006	0.0218791	2.16512	2.16512
4921514A10Rik	4921514A10Rik-001	0.0150695	0.562968	-1.7763
4921531C22Rik	4921531C22Rik-002	0.0253192	4.35322	4.35322
4921536K21Rik	4921536K21Rik-001	0.0231373	0.550279	-1.81726
4930402H24Rik	4930402H24Rik-001	0.00040407	0.656596	-1.52301
4930402H24Rik	4930402H24Rik-005	0.000133542	2.22897	2.22897
4930402H24Rik	4930402H24Rik-009	0.00226493	2.53096	2.53096
4930405A21Rik	4930405A21Rik-001	0.0292142	1.96832	1.96832
4930430F08Rik	4930430F08Rik-201	0.00450592	1.2765	1.2765
4930431F12Rik	4930431F12Rik-201	0.00774633	1.56479	1.56479
4930438A08Rik	4930438A08Rik-001	0.0146837	9.34786	9.34786
4930444G20Rik	4930444G20Rik-001	0.0317988	7.16087	7.16087
4930447F24Rik	4930447F24Rik-002	0.0243637	1.90677e+08	1.90677e+08
4930452B06Rik	4930452B06Rik-001	0.0366476	1.27869	1.27869
4930486L24Rik	4930486L24Rik-201	0.001338	0.443171	-2.25647
4930512B01Rik	4930512B01Rik-201	0.0236349	6.82744	6.82744
4930516B21Rik	4930516B21Rik-201	0.00145379	0.500328	-1.99869
4930518I15Rik	4930518I15Rik-201	0.0082454	1.91497	1.91497
4930520O04Rik	4930520O04Rik-002	0.0246635	1.94844	1.94844
4930522L14Rik	4930522L14Rik-001	0.00132471	2.50659	2.50659
4930533O14Rik	4930533O14Rik-201	0.00886573	0.692379	-1.4443
4930539E08Rik	4930539E08Rik-001	0.00104045	1.63518	1.63518
4930550C14Rik	4930550C14Rik-201	0.00185662	1.72722	1.72722
4930556J24Rik	4930556J24Rik-001	0.0181615	3.55065	3.55065
4930562C15Rik	4930562C15Rik-008	0.00233888	2.91263	2.91263
4930563E22Rik	4930563E22Rik-201	0.0105741	1.90064	1.90064
4930568G15Rik	4930568G15Rik-001	0.00148561	0.226902	-4.40719
4930579G24Rik	4930579G24Rik-001	0.00300037	0.719964	-1.38896
4931403G20Rik	4931403G20Rik-201	0.037133	6.2379	6.2379
4931406C07Rik	4931406C07Rik-202	0.0117029	0.689581	-1.45016
4931414P19Rik	4931414P19Rik-201	0.0295238	0.581207	-1.72056

4931428F04Rik	4931428F04Rik-202	0.0479258	0.737869	-1.35525
4932422M17Rik	4932422M17Rik-001	0.0415374	1.33923	1.33923
4932443L11Rik	4932443L11Rik-201	0.023858	4.69191	4.69191
4933415A04Rik	4933415A04Rik-201	0.0392227	0.532416	-1.87823
4933421O10Rik	4933421O10Rik-001	0.000644468	1.32292	1.32292
4933431E20Rik	4933431E20Rik-001	2.96939e-05	1.88839	1.88839
4933431K14Rik	4933431K14Rik-001	0.0211615	1.69694	1.69694
4933434E20Rik	4933434E20Rik-006	0.0319838	2.5906	2.5906
4933434E20Rik	4933434E20Rik-002	0.0248308	2.78633	2.78633
5031425E22Rik	5031425E22Rik-004	0.0189557	1.24804	1.24804
5031439G07Rik	5031439G07Rik-001	0.0200609	0.522892	-1.91244
5033428I22Rik	5033428I22Rik-201	0.0159265	1.39537	1.39537
5330438D12Rik	5330438D12Rik-201	0.0309224	1.55577	1.55577
5430403G16Rik	5430403G16Rik-001	0.0328073	1.62432	1.62432
5530601H04Rik	5530601H04Rik-003	0.0267562	4.98515	4.98515
5530601H04Rik	5530601H04Rik-008	0.0447636	4831.86	4831.86
5730455P16Rik	5730455P16Rik-201	0.0471587	1.49623	1.49623
5730455P16Rik	5730455P16Rik-003	0.0404905	4.10431	4.10431
5730508B09Rik	5730508B09Rik-001	0.0274344	0.229847	-4.35072
5830444B04Rik	5830444B04Rik-001	0.0345863	0.518498	-1.92865
5830487J09Rik	5830487J09Rik-002	0.0166597	0.711153	-1.40617
5930430L01Rik	5930430L01Rik-001	0.0358141	1.87142	1.87142
6030419C18Rik	6030419C18Rik-201	0.037954	0.565644	-1.7679
6030443J06Rik	6030443J06Rik-004	0.0303663	1.59165	1.59165
6330403K07Rik	6330403K07Rik-001	0.0141246	0.754767	-1.32491
6820431F20Rik	6820431F20Rik-001	0.00503286	1.0972	1.0972
8030453O22Rik	8030453O22Rik-002	0.0053371	1.74878	1.74878
8030462N17Rik	8030462N17Rik-201	0.013935	1.14215	1.14215
8430429K09Rik	8430429K09Rik-004	0.000243055	2.07629	2.07629
8430429K09Rik	8430429K09Rik-003	0.049594	2.43838	2.43838
9130011E15Rik	9130011E15Rik-201	0.000248718	1.46986	1.46986
9130604C24Rik	9130604C24Rik-001	0.0319678	2.00471	2.00471
9230110C19Rik	9230110C19Rik-201	0.000503559	1.69633	1.69633
9230111E07Rik	9230111E07Rik-001	0.00385757	1.86799	1.86799
9330020H09Rik	9330020H09Rik-001	0.0346489	1.85046	1.85046
9330151L19Rik	9330151L19Rik-201	2.67268e-05	1.4589	1.4589
9330159M07Rik	9330159M07Rik-003	0.00381459	1.78775e+06	1.78775e+06
9330182L06Rik	9330182L06Rik-002	0.0306033	0.671072	-1.49015
9430037G07Rik	9430037G07Rik-001	0.00432407	5.4452	5.4452
9430037O13Rik	9430037O13Rik-001	0.0452867	0.387505	-2.58061
9430038I01Rik	9430038I01Rik-004	0.0203526	0.259851	-3.84836
9530056K15Rik	9530056K15Rik-001	0.00484524	2.27549	2.27549
9530056K15Rik	9530056K15Rik-201	0.00111346	3.33929	3.33929

9530068E07Rik	9530068E07Rik-002	0.00600406	1.54192	1.54192
9530077C05Rik	9530077C05Rik-201	0.00105374	1.43436	1.43436
9530085P06Rik	9530085P06Rik-001	0.0388881	3.44821	3.44821
9930021J03Rik	9930021J03Rik-002	0.0277663	1.43858	1.43858
9930104L06Rik	9930104L06Rik-002	0.00209258	1.71151	1.71151
9930104L06Rik	9930104L06Rik-001	0.00497264	1.93107	1.93107
A130010J15Rik	A130010J15Rik-002	0.0162956	2.71376	2.71376
A130014A01Rik	A130014A01Rik-001	1.35955e-06	1.79908	1.79908
A230006K03Rik	A230006K03Rik-001	0.0185991	3.12472	3.12472
A230072C01Rik	A230072C01Rik-002	0.00703173	6.81684	6.81684
A230103J11Rik	A230103J11Rik-002	0.0191988	0.394102	-2.53742
A330017A19Rik	A330017A19Rik-201	0.028558	1.29789	1.29789
A330032B11Rik	A330032B11Rik-201	0.0304039	1.27633	1.27633
A430005L14Rik	A430005L14Rik-001	0.00103272	1.72404	1.72404
A430033K04Rik	A430033K04Rik-002	0.0379316	1.17407	1.17407
A430035B10Rik	A430035B10Rik-001	0.00536981	1728.61	1728.61
A430046D13Rik	A430046D13Rik-201	0.0100118	1.45178	1.45178
A430105J06Rik	A430105J06Rik-001	0.00565904	1.65146	1.65146
A530017D24Rik	A530017D24Rik-001	0.0038434	0.563568	-1.77441
A530095I07Rik	A530095I07Rik-001	0.0346487	3.02646	3.02646
A730011C13Rik	A730011C13Rik-001	0.024924	5.25805	5.25805
A730018C14Rik	A730018C14Rik-201	0.0302597	1.86919	1.86919
A830010M20Rik	A830010M20Rik-001	0.00159825	1.38897	1.38897
A830039N20Rik	A830039N20Rik-001	0.0178129	0.566111	-1.76644
A830080D01Rik	A830080D01Rik-001	0.0229897	0.896837	-1.11503
A830080D01Rik	A830080D01Rik-002	0.00715499	2.18254	2.18254
A930005H10Rik	A930005H10Rik-003	0.0197562	2.07253	2.07253
A930005H10Rik	A930005H10Rik-002	0.0196861	2.53007	2.53007
A930005H10Rik	A930005H10Rik-005	2.69273e-05	4.93756	4.93756
A930007I19Rik	A930007I19Rik-201	0.00918341	4.03506	4.03506
A930018M24Rik	A930018M24Rik-201	0.00138663	5.93849	5.93849
A930019D19Rik	A930019D19Rik-001	0.000825859	2.76521	2.76521
A930032L01Rik	A930032L01Rik-001	0.0243768	7.588	7.588
A930033H14Rik	A930033H14Rik-001	0.0308162	2.12908	2.12908
AA386476	AA386476-201	0.00453055	2.32357	2.32357
AA792892	AA792892-001	0.0473019	1.71488	1.71488
AA914427	AA914427-001	0.0417381	2.65442	2.65442
Aacs	Aacs-001	0.0450171	0.743762	-1.34452
Aaed1	Aaed1-201	0.00185717	1.49916	1.49916
Aamp	Aamp-001	0.0387286	0.838965	-1.19194
Aars	Aars-001	0.0198183	1.35233	1.35233
Aars	Aars-003	0.00325246	2.08461	2.08461
Aars2	Aars2-201	0.00165271	0.765323	-1.30664

Aasdh	Aasdh-014	0.0484684	1.80435	1.80435
Aasdhppt	Aasdhppt-201	0.0261988	1.10928	1.10928
Abca1	Abca1-001	0.00011766	0.489896	-2.04125
Abca2	Abca2-001	0.0193574	0.827526	-1.20842
Abca7	Abca7-001	0.0137726	0.578269	-1.7293
Abca7	Abca7-202	0.035218	0.646266	-1.54735
Abcb1a	Abcb1a-001	0.00309968	1.78843	1.78843
Abcb8	Abcb8-201	0.0217418	0.713665	-1.40122
Abcb9	Abcb9-001	0.000116551	0.327352	-3.05482
Abcb9	Abcb9-002	0.0179533	0.429649	-2.32748
Abcc10	Abcc10-002	0.0191308	3.00901	3.00901
Abcc3	Abcc3-201	0.0027862	0.597064	-1.67486
Abcc3	Abcc3-001	0.0243189	0.618022	-1.61807
Abcc5	Abcc5-003	0.0131276	1.28645	1.28645
Abcc5	Abcc5-006	0.00962602	1.37493	1.37493
Abcc5	Abcc5-008	0.00872924	4.3569	4.3569
Abcc9	Abcc9-003	0.000259923	0.00101506	-985.163
Abcc9	Abcc9-202	0.000259927	0.00101506	-985.161
Abcc9	Abcc9-201	0.000259935	0.00101506	-985.161
Abcc9	Abcc9-001	0.0287023	2.95904	2.95904
Abcd1	Abcd1-001	0.0275881	0.776098	-1.2885
Abcf1	Abcf1-008	0.00919407	3.97618	3.97618
Abhd11	Abhd11-007	0.0105713	2.29142	2.29142
Abhd12	Abhd12-003	0.00442389	2.45818	2.45818
Abhd14b	Abhd14b-001	0.0191215	1.59068	1.59068
Abhd14b	Abhd14b-005	0.00317511	2.91262	2.91262
Abhd16a	Abhd16a-001	0.046248	0.720821	-1.38731
Abhd5	Abhd5-002	0.0269205	1.92562	1.92562
Abhd6	Abhd6-201	0.00106714	2.16443	2.16443
Abi2	Abi2-001	0.0128264	0.72324	-1.38267
Abl1	Abl1-008	0.00110934	0.513721	-1.94658
Ablim1	Ablim1-008	0.014854	4.61437	4.61437
Ablim2	Ablim2-015	0.0306673	0.0416746	-23.9954
Abr	Abr-010	0.049421	0.492321	-2.03119
Abracl	Abracl-201	0.00557073	0.757804	-1.3196
Acaca	Acaca-007	0.0219647	0.393936	-2.53848
Acad10	Acad10-001	0.0116871	3.97388	3.97388
Acad11	Acad11-008	0.0198623	1.48658	1.48658
Acad11	Acad11-001	0.0199461	2.03433	2.03433
Acad11	Acad11-002	0.0211889	2.3777	2.3777
Acad8	Acad8-201	0.00433461	1.46958	1.46958
Acads	Acads-001	0.0232053	0.750934	-1.33168
Acap3	Acap3-001	0.0178095	0.634407	-1.57628

Acat1	Acat1-001	0.0363478	1.08075	1.08075
Acat1	Acat1-003	0.00100948	1.79119	1.79119
Acbd3	Acbd3-001	0.000820465	1.16533	1.16533
Acbd5	Acbd5-202	0.024768	1.31087	1.31087
Acbd5	Acbd5-201	0.0226796	1.39034	1.39034
Acbd6	Acbd6-001	0.0134006	0.472379	-2.11694
Acbd6	Acbd6-002	0.000394423	2.52187	2.52187
Accs	Accs-009	0.0458449	2.90927e-06	-343729
Accs	Accs-001	0.0495	0.637389	-1.5689
Acd	Acd-201	0.00163682	1.36595	1.36595
Acin1	Acin1-024	0.00749277	1.31445	1.31445
Acin1	Acin1-004	0.00764171	1.83105	1.83105
Acly	Acly-005	0.0308583	0.795199	-1.25755
Acly	Acly-004	0.0497907	0.903156	-1.10723
Aco1	Aco1-001	0.0463459	1.29269	1.29269
Aco2	Aco2-006	0.0211008	0.626358	-1.59653
Acot13	Acot13-001	7.80701e-05	1.62184	1.62184
Acot2	Acot2-001	0.000202372	2.38078	2.38078
Acot7	Acot7-002	0.0271603	0.556176	-1.79799
Acot8	Acot8-003	0.0459651	1.4031	1.4031
Acox1	Acox1-003	0.0126252	0.655286	-1.52605
Acsf2	Acsf2-001	0.0279337	0.387317	-2.58186
Acsf3	Acsf3-201	0.000575082	1.40329	1.40329
Acsl4	Acsl4-004	5.1568e-05	0.357278	-2.79894
Acsl4	Acsl4-001	0.0426242	0.562911	-1.77648
Acsl6	Acsl6-008	0.0283011	0.0189864	-52.6692
Acsl6	Acsl6-015	0.0399157	51.822	51.822
Acsm3	Acsm3-002	0.0492341	3.36768	3.36768
Actb	Actb-008	0.00704631	0.632796	-1.58029
Actg1	Actg1-004	0.0198804	0.585998	-1.70649
Actg1	Actg1-006	0.0213361	0.636363	-1.57143
Actg1	Actg1-201	0.00169782	0.756015	-1.32273
Actg1	Actg1-001	0.0129412	0.767367	-1.30316
Actl10	Actl10-001	0.0175496	0.619279	-1.61478
Actl6a	Actl6a-007	0.0439888	17.2255	17.2255
Actl6b	Actl6b-007	0.0208874	0.135479	-7.38122
Actn1	Actn1-201	0.00138859	0.605545	-1.65141
Actn2	Actn2-201	0.0459845	0.237049	-4.21854
Actn4	Actn4-007	0.000852966	0.368782	-2.71163
Actr10	Actr10-201	0.0233699	1.09631	1.09631
Actr1a	Actr1a-201	0.014318	0.855383	-1.16907
Actr1b	Actr1b-003	0.0141682	0.0744364	-13.4343
Actr2	Actr2-003	2.94348e-07	0.439326	-2.27622

Actr3	Actr3-001	0.00165256	0.863756	-1.15773
Acy1	Acy1-001	0.000718867	0.664638	-1.50458
Acyp1	Acyp1-003	0.0230211	1.60486e+06	1.60486e+06
Ada	Ada-001	0.0249463	1.5666	1.5666
Adam11	Adam11-002	0.0455997	67.0982	67.0982
Adam19	Adam19-001	0.0137939	0.696877	-1.43497
Adam1b	Adam1b-001	0.0353748	4.80164	4.80164
Adam21	Adam21-001	0.00885302	5.85132	5.85132
Adam22	Adam22-003	0.0101153	0.51262	-1.95076
Adam22	Adam22-202	0.000120894	0.566211	-1.76613
Adam22	Adam22-201	0.0208014	0.578622	-1.72825
Adam22	Adam22-013	0.0025936	7.19863	7.19863
Adam23	Adam23-005	0.00128279	0.557724	-1.793
Adam23	Adam23-004	0.0178001	2.58929	2.58929
Adamts1	Adamts1-001	0.0215109	0.548786	-1.8222
Adamts10	Adamts10-001	0.0430409	0.414805	-2.41077
Adamts13	Adamts13-002	0.0311512	0.821135	-1.21783
Adamts4	Adamts4-001	4.61649e-05	0.471137	-2.12252
Adarb1	Adarb1-001	0.0340924	1.52959	1.52959
Adarb1	Adarb1-010	0.000565317	1.7888	1.7888
Adarb1	Adarb1-003	0.0185854	2.23398	2.23398
Adat2	Adat2-001	0.000943255	1.55692	1.55692
Adcy6	Adcy6-201	0.000369908	0.773671	-1.29254
Adcy7	Adcy7-201	0.0138168	0.366236	-2.73048
Adcy7	Adcy7-203	0.0272606	0.736009	-1.35868
Adcyap1r1	Adcyap1r1-007	0.0380063	9.63149e-10	-1.03826e+09
Add2	Add2-005	0.0329072	0.116103	-8.61306
Adgra2	Adgra2-001	0.0192492	0.720417	-1.38808
Adgra3	Adgra3-005	0.0404841	1.63968	1.63968
Adgrb2	Adgrb2-004	0.00112895	0.237627	-4.20827
Adgrd1	Adgrd1-001	0.015654	1.39176	1.39176
Adgrf5	Adgrf5-201	0.0318804	1.47374	1.47374
Adgrl1	Adgrl1-001	0.0116712	0.751803	-1.33014
Adgrl1	Adgrl1-005	0.0479623	1.82003	1.82003
Adgrl1	Adgrl1-004	0.0325663	2.21304	2.21304
Adgrl2	Adgrl2-201	0.010818	0.373229	-2.67932
Adgrl2	Adgrl2-019	0.0326476	1.40907	1.40907
Adh5	Adh5-001	0.0457911	1.1548	1.1548
Adi1	Adi1-001	0.00169489	1.462	1.462
Adipor2	Adipor2-001	0.000176281	0.650495	-1.53729
Adm2	Adm2-201	0.00593543	2.2415	2.2415
Adnp	Adnp-004	0.00578475	1.66504	1.66504
Adora2a	Adora2a-201	0.0285281	0.722451	-1.38418

Adprm	Adprm-004	0.0292542	1.35715	1.35715
Adrbk1	Adrbk1-008	0.000544905	3.1917	3.1917
Adrbk1	Adrbk1-010	0.00524922	4.74053	4.74053
Adrbk2	Adrbk2-004	0.0219935	1.33533	1.33533
Adrbk2	Adrbk2-001	0.00584668	4.93795	4.93795
Adrm1	Adrm1-007	0.0325494	0.0353203	-28.3124
Adsl	Adsl-009	0.0204216	0.51558	-1.93956
Aebp2	Aebp2-001	0.0022464	0.790601	-1.26486
Aebp2	Aebp2-201	0.025377	1.3686	1.3686
AF067063	AF067063-001	0.0246005	1.36385e-09	-7.33219e+08
Aff4	Aff4-001	0.040754	0.808204	-1.23731
Aff4	Aff4-006	0.00360669	170.475	170.475
Aftph	Aftph-002	0.0227433	1.28607	1.28607
Aga	Aga-201	0.000881576	1.31849	1.31849
Agap1	Agap1-003	0.000383254	0.560804	-1.78316
Agap1	Agap1-002	0.000832873	1.6434	1.6434
Agap2	Agap2-201	0.0136784	0.786333	-1.27173
Agbl3	Agbl3-006	0.0169839	2.22838	2.22838
Agbl3	Agbl3-007	0.0294455	1.1177e+10	1.1177e+10
Agbl5	Agbl5-006	0.0294884	0.352215	-2.83917
Agbl5	Agbl5-002	0.0148555	2.0389	2.0389
Agbl5	Agbl5-010	0.00193947	5.44644	5.44644
Ager	Ager-001	0.0022869	0.0185722	-53.8439
Agfg1	Agfg1-002	0.000649773	1.40534	1.40534
Agfg1	Agfg1-004	0.00864317	2.08477	2.08477
Agfg1	Agfg1-007	0.00239113	6.02352	6.02352
Agl	Agl-007	0.0330176	1.29267	1.29267
Agl	Agl-002	0.00303644	2.41208	2.41208
Agl	Agl-005	0.0144383	3.22065	3.22065
Agpat5	Agpat5-001	0.00659045	0.851357	-1.17459
Agpat5	Agpat5-002	0.0257519	1.26623	1.26623
Agpat9	Agpat9-003	0.00581407	8.12377	8.12377
Agps	Agps-001	0.0262548	1.16862	1.16862
Agps	Agps-007	0.0111743	1.98357	1.98357
Agrn	Agrn-001	0.00413395	0.377933	-2.64597
Agrn	Agrn-004	0.00234213	0.395209	-2.53031
Agrn	Agrn-002	0.00693408	0.403381	-2.47905
Agrn	Agrn-006	0.000542425	0.465562	-2.14794
Agtpbp1	Agtpbp1-018	0.0150626	4.49349	4.49349
Agtrap	Agtrap-003	0.0395001	0.0141322	-70.7603
Ahcyl2	Ahcyl2-015	0.0292781	8658.13	8658.13
Ahdc1	Ahdc1-004	0.00928333	0.731776	-1.36654
Ahi1	Ahi1-201	0.0247899	1.28308	1.28308

Ahnak	Ahnak-202	0.0170655	0.420875	-2.37601
AI118078	AI118078-001	0.00200915	2.41179	2.41179
AI314180	AI314180-006	0.0496235	1.63821	1.63821
AI314180	AI314180-003	0.0439784	4.21572	4.21572
AI314180	AI314180-007	0.00510354	8.56727	8.56727
AI429214	AI429214-201	0.0196388	0.756063	-1.32264
AI597479	AI597479-001	0.000621466	1.51497	1.51497
AI606181	AI606181-001	0.000327694	0.510904	-1.95731
AI854517	AI854517-007	0.0437782	0.348361	-2.87059
AI854517	AI854517-005	0.0342722	13.9939	13.9939
AI854703	AI854703-001	0.0383283	1.76684	1.76684
Aida	Aida-001	0.000153864	0.675598	-1.48017
Aida	Aida-004	0.0106868	0.720103	-1.38869
Aida	Aida-003	0.0182401	0.733499	-1.36333
Aif1l	Aif1l-001	0.0190209	1.84621	1.84621
Aig1	Aig1-001	0.0497941	1.8416	1.8416
Aim1	Aim1-001	0.00716604	99.9364	99.9364
Aim1l	Aim1l-009	0.0336511	2.01003	2.01003
Aire	Aire-013	0.0210282	0.667339	-1.49849
Ajuba	Ajuba-201	0.0482719	1.43067	1.43067
AK010878	AK010878-001	0.00312663	1.66851	1.66851
Ak1	Ak1-003	0.0372991	0.640849	-1.56043
Ak2	Ak2-002	0.0116206	1.49634	1.49634
Ak4	Ak4-001	0.0152246	0.691374	-1.44639
Ak4	Ak4-007	0.0425096	0.735148	-1.36027
Ak6	Ak6-201	0.00115949	1.56633	1.56633
Ak7	Ak7-201	0.0330454	0.391347	-2.55527
Akap1	Akap1-005	0.00133164	1.8791	1.8791
Akap13	Akap13-201	0.0246486	0.801875	-1.24708
Akap2	Akap2-003	0.0271586	13.904	13.904
Akap8l	Akap8l-201	0.00776076	1.47084	1.47084
Akirin1	Akirin1-003	0.0215737	2.31908	2.31908
Akirin2	Akirin2-001	0.0313454	1.13241	1.13241
Akna	Akna-001	0.0242069	1.73627	1.73627
Akr1a1	Akr1a1-002	0.0239085	0.740577	-1.3503
Akr1a1	Akr1a1-001	0.00366071	1.23694	1.23694
Akt3	Akt3-201	0.00154953	0.681898	-1.4665
Akt3	Akt3-002	0.00053874	3.47602	3.47602
Aldh16a1	Aldh16a1-010	0.00407047	0.600146	-1.66626
Aldh16a1	Aldh16a1-002	0.0463342	0.688373	-1.4527
Aldh18a1	Aldh18a1-004	0.0492321	0.636515	-1.57105
Aldh18a1	Aldh18a1-007	0.000521299	1.48615	1.48615
Aldh1l2	Aldh1l2-001	0.00101105	1.63168	1.63168

Aldh1l2	Aldh1l2-003	0.011954	3.7528	3.7528
Aldh1l2	Aldh1l2-002	9.46337e-05	5.29592	5.29592
Aldh3a1	Aldh3a1-001	0.000419496	0.126095	-7.93051
Aldh3a2	Aldh3a2-001	0.00875585	3.90694	3.90694
Aldh5a1	Aldh5a1-001	0.00170118	1.44176	1.44176
Aldh7a1	Aldh7a1-001	0.00452181	1.48164	1.48164
Aldh9a1	Aldh9a1-001	0.00967619	1.20489	1.20489
Alg11	Alg11-004	0.0167875	2.78919	2.78919
Alg12	Alg12-001	0.00365927	0.561367	-1.78137
Alg12	Alg12-002	0.0137233	2.65286	2.65286
Alg13	Alg13-008	0.00787507	2.24549	2.24549
Alg5	Alg5-002	0.0418772	0.445515	-2.24459
Alk	Alk-201	0.00258517	0.688753	-1.4519
Alkbh4	Alkbh4-004	0.0304074	3.72304	3.72304
Alkbh6	Alkbh6-002	0.00315767	1.95616	1.95616
Alms1	Alms1-002	0.0489818	0.687087	-1.45542
Alpi	Alpi-001	0.0269332	2.85828	2.85828
Als2	Als2-003	0.0267499	1.28259	1.28259
Amacr	Amacr-201	0.00751758	1.37979	1.37979
Ambra1	Ambra1-003	0.0299008	0.52936	-1.88907
Amdhd2	Amdhd2-001	0.0144299	0.39831	-2.5106
Amer1	Amer1-001	0.00150337	1.43805	1.43805
Amfr	Amfr-001	0.00927648	0.83054	-1.20404
Amh	Amh-201	0.0394339	1.78211	1.78211
Amigo2	Amigo2-201	0.0181494	0.518873	-1.92725
Amn1	Amn1-009	0.0311673	0.318777	-3.13699
Amz1	Amz1-006	0.0218497	1.6094	1.6094
Amz2	Amz2-005	0.0190757	0.570531	-1.75275
Anapc1	Anapc1-010	0.0344933	1.25183	1.25183
Anapc1	Anapc1-009	0.0145129	1.53014	1.53014
Anapc1	Anapc1-005	0.00582898	2.51966	2.51966
Anapc11	Anapc11-002	0.00410908	0.543853	-1.83873
Anapc11	Anapc11-004	0.0281037	0.688204	-1.45306
Anapc16	Anapc16-002	0.00822866	1.52744	1.52744
Anapc16	Anapc16-005	0.00552444	1.72394	1.72394
Anapc5	Anapc5-003	0.0332284	0.697897	-1.43288
Anapc5	Anapc5-005	0.00174344	0.757722	-1.31975
Anapc5	Anapc5-012	0.0340353	2.60729	2.60729
Angpt2	Angpt2-001	0.00411639	0.456054	-2.19272
Angptl1	Angptl1-002	0.0409184	0.258271	-3.8719
Angptl3	Angptl3-002	0.0199269	0.173819	-5.75311
Angptl4	Angptl4-003	0.00735263	0.0168392	-59.3853
Angptl6	Angptl6-001	0.0466998	1.98648	1.98648

Ank	Ank-001	0.0489042	0.60938	-1.64101
Ank2	Ank2-020	0.0187799	0.0942078	-10.6148
Ank2	Ank2-003	0.0370075	0.347417	-2.87839
Ank2	Ank2-015	0.0478684	1.47796	1.47796
Ank2	Ank2-022	0.00556547	1.48087	1.48087
Ank2	Ank2-024	0.00179638	1.68344	1.68344
Ank3	Ank3-001	0.0163764	0.632971	-1.57985
Ank3	Ank3-029	0.000351342	0.695487	-1.43784
Ank3	Ank3-018	0.0354368	2.85851	2.85851
Ankef1	Ankef1-001	0.0204266	2.01959	2.01959
Ankef1	Ankef1-006	0.00755493	8.21489	8.21489
Ankfy1	Ankfy1-005	0.0205618	0.779472	-1.28292
Ankhd1	Ankhd1-018	0.0270922	0.701936	-1.42463
Ankib1	Ankib1-002	0.0226988	0.245695	-4.07009
Ankle2	Ankle2-002	0.0452701	1.14459	1.14459
Ankmy2	Ankmy2-201	0.00257296	1.3326	1.3326
Ankra2	Ankra2-003	0.0363439	0.691291	-1.44657
Ankra2	Ankra2-005	0.0343868	1.45772	1.45772
Ankrd10	Ankrd10-202	0.0233002	0.88929	-1.12449
Ankrd11	Ankrd11-001	0.0101369	1.11149	1.11149
Ankrd11	Ankrd11-003	0.0357191	1.44305	1.44305
Ankrd11	Ankrd11-002	0.00997695	1.45974	1.45974
Ankrd11	Ankrd11-201	0.0059592	2.04025	2.04025
Ankrd12	Ankrd12-005	0.00854072	7.18903	7.18903
Ankrd13b	Ankrd13b-001	0.0367795	0.640445	-1.56141
Ankrd13c	Ankrd13c-009	0.044222	0.00233195	-428.826
Ankrd13c	Ankrd13c-201	0.00577084	2.08578	2.08578
Ankrd17	Ankrd17-002	0.0103702	0.715645	-1.39734
Ankrd24	Ankrd24-013	0.0378582	0.0751659	-13.3039
Ankrd27	Ankrd27-002	0.000590576	1.64278	1.64278
Ankrd27	Ankrd27-009	0.042649	3.36149	3.36149
Ankrd29	Ankrd29-001	0.0450435	3.58871	3.58871
Ankrd40	Ankrd40-005	0.0121255	0.388348	-2.57501
Ankrd42	Ankrd42-005	0.0332317	0.103396	-9.67159
Ankrd45	Ankrd45-001	0.0174099	0.715597	-1.39743
Ankrd46	Ankrd46-201	0.000737572	1.20827	1.20827
Ankrd50	Ankrd50-001	0.0110878	0.767633	-1.30271
Ankrd50	Ankrd50-003	0.0384773	1.57733	1.57733
Ankrd9	Ankrd9-005	0.0302429	91.1251	91.1251
Anks3	Anks3-201	0.0202007	1.18883	1.18883
Ano8	Ano8-201	0.000200735	0.674789	-1.48194
Anp32a	Anp32a-001	0.000152007	0.74435	-1.34345
Anp32b	Anp32b-001	0.029629	1.24189	1.24189

Anp32e	Anp32e-004	0.0113105	1.83083	1.83083
Antxr2	Antxr2-001	0.00475933	0.784598	-1.27454
Anxa2	Anxa2-005	0.0368692	0.285887	-3.49788
Anxa4	Anxa4-009	0.000261062	2.8019	2.8019
Anxa4	Anxa4-008	0.0437531	4.05882	4.05882
Anxa6	Anxa6-001	0.000195928	0.566559	-1.76504
Anxa9	Anxa9-201	0.0356686	0.0108943	-91.791
Ap1ar	Ap1ar-006	0.00272411	2.07006	2.07006
Ap1g1	Ap1g1-001	0.000411417	0.855373	-1.16908
Ap1g1	Ap1g1-002	0.0027485	1.96261	1.96261
Ap1g2	Ap1g2-001	1.38891e-05	0.367944	-2.71781
Ap1m1	Ap1m1-002	0.0472092	1.31294	1.31294
Ap1m1	Ap1m1-004	8.16904e-05	1.89228	1.89228
Ap1s2	Ap1s2-003	0.00135028	0.71044	-1.40758
Ap1s3	Ap1s3-001	0.00923592	1.10425	1.10425
Ap2a1	Ap2a1-204	0.00249727	1.50272	1.50272
Ap2a2	Ap2a2-001	0.0427462	0.953325	-1.04896
Ap2b1	Ap2b1-002	0.0495466	0.234449	-4.26532
Ap2b1	Ap2b1-001	0.000172264	0.565213	-1.76924
Ap2b1	Ap2b1-005	0.0289377	1.37524	1.37524
Ap2b1	Ap2b1-006	0.013038	3.84507	3.84507
Ap3m1	Ap3m1-003	0.00698894	2.65015	2.65015
Ap3m1	Ap3m1-006	0.0390737	8.34076e+06	8.34076e+06
Ap3m2	Ap3m2-201	0.00581953	1.16668	1.16668
Ap4e1	Ap4e1-001	0.00371977	1.24568	1.24568
Ap4e1	Ap4e1-007	0.0182688	14.0262	14.0262
Ap5s1	Ap5s1-002	0.00515192	0.123816	-8.0765
Ap5s1	Ap5s1-003	0.0378271	1.428	1.428
Apba1	Apba1-201	0.0490751	1.41232	1.41232
Apbb2	Apbb2-001	0.0112135	3.24061	3.24061
Apc	Apc-201	0.0053921	0.689101	-1.45117
Apeh	Apeh-001	0.0464405	0.521799	-1.91645
Aph1b	Aph1b-001	9.8684e-05	0.454869	-2.19844
Aph1b	Aph1b-003	0.00444406	4.02902	4.02902
Aph1c	Aph1c-001	0.017636	1.63435	1.63435
Apip	Apip-001	0.00479745	1.35249	1.35249
Apitd1	Apitd1-003	0.011877	3.80383	3.80383
Aplp1	Aplp1-201	0.0183073	0.696667	-1.43541
Aplp2	Aplp2-202	0.00147912	0.675887	-1.47954
Aplp2	Aplp2-201	0.0136455	1.12472	1.12472
Apmap	Apmap-001	0.0476986	1.18281	1.18281
Apobr	Apobr-001	0.00185578	1.43841	1.43841
Apold1	Apold1-001	0.000231411	5.82755	5.82755

Apoo	Apoo-001	0.0279133	1.49802	1.49802
Apopt1	Apopt1-001	0.00523724	1.27305	1.27305
App	App-201	0.000320891	0.83761	-1.19387
Appl1	Appl1-003	0.0350338	5.62191e-07	-1.77875e+06
Aqp10-ps	Aqp10-ps-001	0.0124064	4.20089	4.20089
Araf	Araf-001	0.0225816	0.497425	-2.01035
Araf	Araf-004	0.0393311	1.54197	1.54197
Araf	Araf-008	0.0249559	2.09136	2.09136
Araf	Araf-007	0.0170339	5.60771	5.60771
Arap3	Arap3-201	0.0136143	0.500712	-1.99715
Arcn1	Arcn1-001	0.00728133	1.25861	1.25861
Arcn1	Arcn1-003	0.0185116	2.42923	2.42923
Arf1	Arf1-001	0.0132561	1.10825	1.10825
Arfgap1	Arfgap1-013	0.00327036	0.194748	-5.13484
Arfgef2	Arfgef2-002	0.0282441	6.81582	6.81582
Arfrp1	Arfrp1-001	0.0199737	0.606842	-1.64787
Arhgap1	Arhgap1-201	0.0106951	2.89414	2.89414
Arhgap19	Arhgap19-001	0.0367939	1.28165	1.28165
Arhgap21	Arhgap21-010	0.0249616	0.615074	-1.62582
Arhgap22	Arhgap22-005	0.0374947	8.13261e-10	-1.22962e+09
Arhgap22	Arhgap22-006	0.0277159	0.190474	-5.25006
Arhgap26	Arhgap26-017	0.028063	1.61089	1.61089
Arhgap28	Arhgap28-001	0.00911792	0.565151	-1.76944
Arhgap30	Arhgap30-002	0.0387952	0.656461	-1.52332
Arhgap32	Arhgap32-002	0.00887106	0.267795	-3.7342
Arhgap33os	Arhgap33os-001	0.0437378	1.57289	1.57289
Arhgap44	Arhgap44-003	0.0293493	0.126266	-7.91979
Arhgdib	Arhgdib-003	0.0115781	0.0441723	-22.6386
Arhgdig	Arhgdig-002	0.00269293	4.85027	4.85027
Arhgef1	Arhgef1-016	0.0259418	0.535411	-1.86773
Arhgef1	Arhgef1-003	0.0419746	0.664045	-1.50592
Arhgef10	Arhgef10-001	0.0281956	2.14821	2.14821
Arhgef19	Arhgef19-003	0.0252392	320.696	320.696
Arhgef2	Arhgef2-003	0.010285	1.6153	1.6153
Arhgef2	Arhgef2-004	0.0467921	1.815	1.815
Arhgef2	Arhgef2-002	0.00100905	2.52253	2.52253
Arhgef25	Arhgef25-202	0.00232648	0.744218	-1.34369
Arhgef28	Arhgef28-201	0.00388646	1.3448	1.3448
Arhgef3	Arhgef3-201	0.0175599	1.28348	1.28348
Arhgef40	Arhgef40-004	0.0473008	0.464593	-2.15242
Arhgef40	Arhgef40-014	0.0356185	0.497198	-2.01127
Arhgef40	Arhgef40-003	0.00535614	0.578868	-1.72751
Arhgef40	Arhgef40-020	0.020278	0.661792	-1.51105

Arhgef40	Arhgef40-011	0.00655023	0.690478	-1.44827
Arhgef6	Arhgef6-003	0.0150197	0.347548	-2.8773
Arid1b	Arid1b-001	0.0337488	0.791477	-1.26346
Arid2	Arid2-001	0.0481835	0.747078	-1.33855
Arid3a	Arid3a-003	0.0277122	0.540351	-1.85065
Arid3a	Arid3a-001	0.0358165	0.62356	-1.60369
Arid3b	Arid3b-201	0.0349991	1.41706	1.41706
Arid4a	Arid4a-003	0.0338442	1.27773e+06	1.27773e+06
Arid4b	Arid4b-001	0.00352574	0.836728	-1.19513
Arih1	Arih1-001	3.6346e-05	0.632789	-1.58031
Arih1	Arih1-002	0.000739691	2.04313	2.04313
Arih2	Arih2-009	0.0242928	4.05203	4.05203
Arl1	Arl1-001	0.000134278	1.28533	1.28533
Arl10	Arl10-004	0.000234215	10.3239	10.3239
Arl11	Arl11-201	0.0426373	3.17342	3.17342
Arl14ep	Arl14ep-004	0.00179893	1.72062	1.72062
Arl14ep	Arl14ep-002	0.000715375	2.60723	2.60723
Arl15	Arl15-001	0.0124558	1.28677	1.28677
Arl16	Arl16-003	0.0257765	1.69042	1.69042
Arl2	Arl2-003	0.0191518	3.17304	3.17304
Arl4a	Arl4a-001	0.0346429	0.807924	-1.23774
Arl4a	Arl4a-003	0.00283389	2.20414	2.20414
Arl4a	Arl4a-004	0.0478996	3.28103	3.28103
Arl4c	Arl4c-001	0.0336614	0.292465	-3.41922
Arl5b	Arl5b-002	0.0237578	0.688629	-1.45216
Arl5b	Arl5b-001	0.00569006	0.775759	-1.28906
Arl6	Arl6-003	0.0301276	1.53156	1.53156
Arl6ip1	Arl6ip1-006	0.00229575	5.59089	5.59089
Arl8b	Arl8b-002	0.0147736	1.45269	1.45269
Arl8b	Arl8b-001	1.98892e-05	1.58914	1.58914
Arl9	Arl9-002	0.00481873	2.76823	2.76823
Armc2	Armc2-004	0.015239	1.92686	1.92686
Armc8	Armc8-004	0.00963411	2.82609	2.82609
Armcx2	Armcx2-003	0.0268389	3.68891	3.68891
Armt1	Armt1-005	0.000359997	0.244392	-4.09178
Armt1	Armt1-002	0.0305712	0.805663	-1.24121
Arntl	Arntl-201	0.0111246	0.734862	-1.3608
Arpc1a	Arpc1a-005	0.0283114	0.215268	-4.64537
Arpc1b	Arpc1b-010	0.0484797	0.54421	-1.83753
Arpc1b	Arpc1b-011	0.0309463	0.675854	-1.47961
Arpc2	Arpc2-003	0.0492959	0.509701	-1.96193
Arpc2	Arpc2-001	0.0205643	0.819587	-1.22013
Arpc3	Arpc3-001	0.00334802	0.77047	-1.29791

Arpc5	Arpc5-001	6.67893e-06	0.674275	-1.48308
Arpc5	Arpc5-002	5.03734e-05	2.03887	2.03887
Arpc5l	Arpc5l-001	0.00177225	0.54757	-1.82625
Arpc5l	Arpc5l-003	0.000537029	1.96502	1.96502
Arpp19	Arpp19-201	0.00118904	0.592909	-1.6866
Arpp19	Arpp19-001	0.0131794	0.61995	-1.61303
Arpp19	Arpp19-010	0.000386669	1.28312	1.28312
Arrb2	Arrb2-008	0.0118215	2.75553	2.75553
Arrb2	Arrb2-001	0.00940284	4.04802	4.04802
Arrdc3	Arrdc3-001	2.28952e-05	0.524156	-1.90783
Arsb	Arsb-201	0.0440654	0.715462	-1.3977
Arsk	Arsk-003	0.00867597	2.79786	2.79786
Arvcf	Arvcf-201	0.00519802	0.442358	-2.26061
Arvcf	Arvcf-001	0.00831833	0.465321	-2.14905
Arvcf	Arvcf-005	0.0321284	0.760594	-1.31476
Asap1	Asap1-004	0.00403468	0.648017	-1.54317
Asb1	Asb1-002	0.00996691	12.7369	12.7369
Asb13	Asb13-001	0.00426878	1.62429	1.62429
Asb13	Asb13-004	0.00669605	1.63133	1.63133
Asb16	Asb16-001	0.00114572	3.51239	3.51239
Asb2	Asb2-002	0.0489618	0.184635	-5.41608
Ascc1	Ascc1-202	0.0143718	2.04132	2.04132
Ascc2	Ascc2-002	0.0302972	0.741094	-1.34936
Ascc2	Ascc2-001	0.0113589	1.39741	1.39741
Ascc3	Ascc3-201	0.00261196	1.15013	1.15013
Ash1l	Ash1l-002	0.0329533	0.339928	-2.9418
Ash1l	Ash1l-201	0.0217413	1.31192	1.31192
Ash2l	Ash2l-007	0.0348655	6.17222e-12	-1.62016e+11
Ash2l	Ash2l-201	0.025792	1.36057	1.36057
Asic1	Asic1-201	0.004021	1.37439	1.37439
Asic3	Asic3-004	0.0484902	3.33709e-05	-29966.2
Asic5	Asic5-001	0.0119655	0.379644	-2.63405
Asl	Asl-006	0.0209481	0.654515	-1.52785
Asns	Asns-001	0.000898074	1.45236	1.45236
Asns	Asns-002	0.012505	1.50281	1.50281
Asph	Asph-002	0.0154429	1.25532	1.25532
Asph	Asph-007	0.00939982	3.09994	3.09994
Asrgl1	Asrgl1-001	0.000148479	1.42878	1.42878
Aste1	Aste1-005	0.00507408	0.275866	-3.62495
Aste1	Aste1-004	0.0136072	2.47054	2.47054
Astn1	Astn1-001	0.00015549	0.508373	-1.96706
Astn1	Astn1-005	0.00772945	0.616947	-1.62088
Asxl2	Asxl2-006	0.00293939	1.80124	1.80124

Ate1	Ate1-202	0.00575313	0.869956	-1.14948
Ate1	Ate1-203	0.00913896	2.88942	2.88942
Atf1	Atf1-005	0.0308219	0.390916	-2.5581
Atf1	Atf1-003	0.01383	1.89891	1.89891
Atf2	Atf2-201	0.00290173	0.396524	-2.52191
Atf2	Atf2-005	0.0122825	0.627208	-1.59437
Atf2	Atf2-006	0.0367875	1.61838	1.61838
Atf2	Atf2-007	0.026707	1.65873	1.65873
Atf3	Atf3-001	0.00216171	3.52233	3.52233
Atf3	Atf3-002	0.000558489	8.66888	8.66888
Atf4	Atf4-201	0.000255355	1.52739	1.52739
Atf5	Atf5-202	0.0168866	0.348289	-2.87118
Atf6	Atf6-001	0.000295613	1.82823	1.82823
Atf7	Atf7-009	0.000631269	0.0432758	-23.1076
Atf7	Atf7-006	0.0105603	14.04	14.04
Atf7ip	Atf7ip-001	0.000627188	0.666373	-1.50066
Atf7ip	Atf7ip-003	0.0497483	1.45272	1.45272
Atg12	Atg12-201	0.0353356	0.670502	-1.49142
Atg13	Atg13-002	0.0393075	1.07759	1.07759
Atg14	Atg14-201	0.00219504	1.34479	1.34479
Atg16l1	Atg16l1-010	0.0373266	0.0905676	-11.0415
Atg16l1	Atg16l1-011	0.0406434	1.46811	1.46811
Atg2b	Atg2b-201	0.0300457	1.15298	1.15298
Atg4a-ps	Atg4a-ps-001	0.00313231	1.24468	1.24468
Atg5	Atg5-201	0.00125886	1.37231	1.37231
Atg7	Atg7-001	0.0231532	0.818622	-1.22156
Atg7	Atg7-004	0.00590577	10.1094	10.1094
Atg7	Atg7-002	0.0105093	11.9262	11.9262
Atg7	Atg7-003	0.0100444	44.2134	44.2134
Atg9a	Atg9a-201	0.0105196	1.60291	1.60291
Atg9a	Atg9a-003	0.000431692	3.49384	3.49384
Atg9a	Atg9a-007	0.000372649	4.80175	4.80175
Atl2	Atl2-201	0.0314958	1.11781	1.11781
Atl3	Atl3-201	0.0100228	0.742001	-1.34771
Atp10d	Atp10d-001	0.00495973	0.631292	-1.58405
Atp1a1	Atp1a1-003	0.0199876	0.658648	-1.51826
Atp1a1	Atp1a1-001	0.000887423	0.709734	-1.40898
Atp1a3	Atp1a3-001	0.00190109	0.753723	-1.32675
Atp1a3	Atp1a3-004	0.0200964	0.830187	-1.20455
Atp2a2	Atp2a2-003	0.028368	0.559794	-1.78637
Atp2b1	Atp2b1-201	2.33786e-05	0.737908	-1.35518
Atp2b4	Atp2b4-001	0.00846755	0.286128	-3.49494
Atp2b4	Atp2b4-201	0.00740097	0.320198	-3.12307

Atp2c1	Atp2c1-007	0.0301772	0.000182634	-5475.42
Atp2c1	Atp2c1-005	0.00747816	0.771416	-1.29632
Atp5b	Atp5b-005	0.0333477	0.63961	-1.56345
Atp5c1	Atp5c1-003	0.00625069	2.14844	2.14844
Atp5e	Atp5e-002	0.0406315	0.739214	-1.35279
Atp5g3	Atp5g3-004	0.0391012	0.632914	-1.57999
Atp5j	Atp5j-002	0.0151165	0.246729	-4.05303
Atp5o	Atp5o-007	0.0485243	1.71985	1.71985
Atp5s	Atp5s-201	0.0127201	1.60973	1.60973
Atp6ap1	Atp6ap1-006	0.033785	0.477085	-2.09606
Atp6ap1l	Atp6ap1l-001	0.000274009	2.0377	2.0377
Atp6v0e2	Atp6v0e2-001	0.0166337	1.17845	1.17845
Atp6v1a	Atp6v1a-006	0.0409348	0.146459	-6.82783
Atp6v1a	Atp6v1a-002	0.0025167	0.638443	-1.56631
Atp6v1a	Atp6v1a-001	0.000658418	0.728092	-1.37345
Atp6v1b2	Atp6v1b2-004	0.00411333	0.195046	-5.12699
Atp6v1b2	Atp6v1b2-001	0.00174867	1.23047	1.23047
Atp6v1d	Atp6v1d-201	0.00353015	1.1411	1.1411
Atp6v1f	Atp6v1f-003	0.0185576	0.389336	-2.56847
Atp7b	Atp7b-001	0.00388511	1.47824	1.47824
Atp8a1	Atp8a1-003	0.0337274	1.31896	1.31896
Atp8a1	Atp8a1-005	0.0251443	2.18264	2.18264
Atp8a1	Atp8a1-014	0.00230821	5.89817	5.89817
Atp8b2	Atp8b2-003	0.0202276	0.59208	-1.68896
Atp9b	Atp9b-201	0.035938	0.72492	-1.37946
Atpaf2	Atpaf2-001	0.000233494	1.47008	1.47008
Atpif1	Atpif1-001	0.00427785	0.766535	-1.30457
Atr	Atr-001	0.00711937	0.895646	-1.11651
Atr	Atr-009	0.00338429	2.53527	2.53527
Atrnl1	Atrnl1-201	0.000912474	0.885006	-1.12994
Atrx	Atrx-001	0.0027492	0.864008	-1.1574
Atrx	Atrx-021	0.03193	1.63573	1.63573
Atrx	Atrx-006	0.00067821	2.60295	2.60295
Atrx	Atrx-011	0.0164833	3.54683	3.54683
Atxn10	Atxn10-201	0.000600279	0.791113	-1.26404
Atxn1l	Atxn1l-201	0.0468369	0.788561	-1.26813
Atxn2	Atxn2-019	0.0441865	0.802618	-1.24592
Atxn2	Atxn2-002	0.0355544	1.26813	1.26813
Atxn2	Atxn2-004	0.0253555	1.48414	1.48414
Atxn2l	Atxn2l-005	0.00120827	1.35389	1.35389
Atxn2l	Atxn2l-002	0.00758409	1.45179	1.45179
Atxn3	Atxn3-001	0.0306699	1.14893	1.14893
Atxn7l3b	Atxn7l3b-001	0.0441812	1.21595	1.21595

AU040320	AU040320-003	0.0044508	1.23919	1.23919
AU041133	AU041133-201	0.00392475	8.40526	8.40526
Auh	Auh-002	0.00341242	1.72177	1.72177
Aup1	Aup1-003	0.00435178	2.43813	2.43813
Aurka	Aurka-002	0.0316258	1.61183	1.61183
Aurka	Aurka-201	0.0316258	1.61183	1.61183
Aurkaip1	Aurkaip1-002	0.00138795	1.56992	1.56992
Auts2	Auts2-015	0.0484127	0.118753	-8.42083
Auts2	Auts2-001	0.0161261	0.67032	-1.49183
Auts2	Auts2-203	0.0377558	3.82756	3.82756
AV026068	AV026068-002	9.24919e-06	5.07314	5.07314
Avpi1	Avpi1-001	0.0213806	1.67183	1.67183
AY512931	AY512931-001	0.0378392	1.52193	1.52193
Azi2	Azi2-008	0.0238774	0.430951	-2.32045
Azi2	Azi2-001	0.041967	0.795728	-1.25671
Azin1	Azin1-003	0.0245641	0.722036	-1.38497
Azin1	Azin1-005	0.0359811	1.47355	1.47355
Azin2	Azin2-004	0.0391317	1.49058	1.49058
B130034C11Rik	B130034C11Rik-001	0.0127298	3.01171	3.01171
B130046B21Rik	B130046B21Rik-201	0.0471099	1.1985	1.1985
B230118H07Rik	B230118H07Rik-003	0.000717503	1.6806	1.6806
B230216N24Rik	B230216N24Rik-005	0.0308346	1.28275	1.28275
B230217C12Rik	B230217C12Rik-004	0.000841744	2.95281	2.95281
B230219D22Rik	B230219D22Rik-201	0.00128194	1.58337	1.58337
B230307C23Rik	B230307C23Rik-001	0.0262091	1.6516	1.6516
B230317F23Rik	B230317F23Rik-001	0.0249735	2.85054	2.85054
B2m	B2m-001	4.54769e-05	0.614419	-1.62755
B330016D10Rik	B330016D10Rik-201	0.00077401	1.93899	1.93899
B3galt1	B3galt1-002	0.00640313	0.49781	-2.0088
B3galt1	B3galt1-201	1.54727e-05	0.514832	-1.94238
B3galt1	B3galt1-003	0.0422586	0.515103	-1.94136
B3gat3	B3gat3-201	0.0177988	0.819912	-1.21964
B3gnt2	B3gnt2-002	0.00654046	0.496621	-2.01361
B430212C06Rik	B430212C06Rik-001	0.0131136	0.135819	-7.36272
B430212C06Rik	B430212C06Rik-003	0.0131136	0.13582	-7.36271
B4galt1	B4galt1-001	0.0232515	0.698218	-1.43222
B4galt1	B4galt1-002	0.0320926	5.12156e+07	5.12156e+07
B4galt3	B4galt3-012	0.0278772	9.04916	9.04916
B4galt5	B4galt5-001	0.0132143	0.710586	-1.40729
B4galt6	B4galt6-201	0.0189909	0.77909	-1.28355
B630019A10Rik	B630019A10Rik-001	0.0204917	0.303065	-3.29962
B930036N10Rik	B930036N10Rik-201	0.0323035	0.000619048	-1615.38
B930095G15Rik	B930095G15Rik-002	0.00916682	0.690403	-1.44843

B9d1	B9d1-001	0.00435697	0.709028	-1.41038
Bace2	Bace2-201	0.00911194	0.832244	-1.20157
Bag1	Bag1-002	0.0305431	1.29356	1.29356
Bag6	Bag6-001	0.0185757	1.16161	1.16161
Bahcc1	Bahcc1-003	0.000712145	1.7457	1.7457
Baiap2l2	Baiap2l2-004	0.0185054	0.153685	-6.5068
Bak1	Bak1-002	0.0332202	1.63554	1.63554
Banp	Banp-001	0.0223235	0.533631	-1.87395
Banp	Banp-013	0.0380524	422.787	422.787
Bard1	Bard1-001	0.0245721	0.780554	-1.28114
Barhl1	Barhl1-001	0.00308872	0.0807185	-12.3887
Baz2a	Baz2a-202	0.00163853	0.699215	-1.43018
Baz2a	Baz2a-201	0.00881013	0.750477	-1.33249
BB557941	BB557941-001	0.0253106	6.60222	6.60222
Bbs10	Bbs10-201	0.000175819	2.03053	2.03053
Bbs12	Bbs12-001	0.0061162	1.81012	1.81012
Bbs2	Bbs2-001	0.000866286	1.62216	1.62216
Bbs4	Bbs4-201	0.00193986	1.65236	1.65236
Bbs9	Bbs9-003	0.0479821	2.03687	2.03687
Bbx	Bbx-001	0.000498693	0.744134	-1.34384
BC001981	BC001981-001	0.00113363	8.4723	8.4723
BC003331	BC003331-003	0.0333232	1.26206	1.26206
BC003331	BC003331-006	0.0485419	2.28826	2.28826
BC003331	BC003331-005	0.0351368	3.942	3.942
BC021767	BC021767-002	0.0336384	410602	410602
BC025920	BC025920-001	0.0472588	11.3268	11.3268
BC029214	BC029214-011	0.0382866	3.35238	3.35238
BC029214	BC029214-008	0.0196768	9.10882	9.10882
BC030343	BC030343-001	0.00129692	2.31088	2.31088
BC030867	BC030867-003	0.0342741	2.29108	2.29108
BC037034	BC037034-001	0.0423994	0.631409	-1.58376
BC039771	BC039771-001	0.0258463	1.23476	1.23476
BC048644	BC048644-002	0.0310233	2.98877	2.98877
BC052040	BC052040-001	0.00573711	1.17389	1.17389
BC055324	BC055324-004	0.0013723	3.40084	3.40084
BC065397	BC065397-006	0.0479009	106.336	106.336
BC089491	BC089491-201	0.0224466	3.75408	3.75408
Bcam	Bcam-001	0.0143659	0.440805	-2.26858
Bcap29	Bcap29-202	0.0396597	1.13966	1.13966
Bcap31	Bcap31-001	0.0283324	0.783436	-1.27643
Bcar1	Bcar1-201	0.0325116	0.825204	-1.21182
Bcas3os2	Bcas3os2-001	0.0255999	4.94036	4.94036
Bcat1	Bcat1-002	0.0277636	1.39455	1.39455

Bcat2	Bcat2-005	0.00214461	1.86711	1.86711
Bccip	Bccip-001	0.0227648	1.60547	1.60547
Bcdin3d	Bcdin3d-201	0.0132344	1.45288	1.45288
Bche	Bche-001	0.0300686	13.3857	13.3857
Bcl2	Bcl2-001	0.0398819	1.28867	1.28867
Bcl2l1	Bcl2l1-002	0.0368919	0.38316	-2.60988
Bcl2l11	Bcl2l11-001	0.00758186	0.482732	-2.07154
Bcl2l13	Bcl2l13-001	0.0253809	1.12621	1.12621
Bcl2l2	Bcl2l2-001	0.0103827	1.18186	1.18186
Bcl7b	Bcl7b-003	0.0495461	1.4274	1.4274
Bcl9l	Bcl9l-201	0.00110892	0.719222	-1.39039
Bclaf1	Bclaf1-002	0.00633414	0.761092	-1.3139
Bclaf1	Bclaf1-008	0.0483574	1.4497	1.4497
Bcor	Bcor-003	0.0350087	0.727355	-1.37484
Bcr	Bcr-201	0.0466384	0.941339	-1.06232
Begain	Begain-001	0.00552847	0.697327	-1.43405
Bend4	Bend4-001	0.00305752	0.30682	-3.25924
Bend4	Bend4-002	0.00690348	0.726711	-1.37606
Bend6	Bend6-001	0.000354907	2.04156	2.04156
Bet1	Bet1-001	0.049259	1.16562	1.16562
Bex1	Bex1-001	0.0299196	1.22505	1.22505
Bgn	Bgn-001	0.00579027	0.282697	-3.53736
Bicd1	Bicd1-001	0.00848553	0.612536	-1.63256
Bid	Bid-004	0.0382656	0.343228	-2.91352
Bin3	Bin3-201	0.000615791	0.723215	-1.38271
Bivm	Bivm-003	0.0361212	1.55766	1.55766
Blcap	Blcap-001	0.0193053	1.23731	1.23731
Blcap	Blcap-006	0.0494432	14.5303	14.5303
Bloc1s1	Bloc1s1-001	0.00030732	0.744622	-1.34296
Bloc1s2	Bloc1s2-201	0.0255579	1.39879	1.39879
Bloc1s4	Bloc1s4-001	0.00426441	0.549866	-1.81862
Bloc1s5	Bloc1s5-201	0.00388004	1.37383	1.37383
Bloc1s6	Bloc1s6-002	0.000170319	1.40052	1.40052
Blvra	Blvra-001	1.98482e-06	1.54462	1.54462
Blzf1	Blzf1-003	0.00128707	1.32786	1.32786
Blzf1	Blzf1-001	0.0473484	1.92383	1.92383
Bmf	Bmf-001	0.000534113	0.444801	-2.24819
Bmp1	Bmp1-201	0.00528403	0.718743	-1.39132
Bmpr1a	Bmpr1a-001	0.015981	0.75603	-1.3227
Bmpr2	Bmpr2-001	0.0211471	0.76996	-1.29877
Bms1	Bms1-002	0.0277334	1.76634	1.76634
Bnip1	Bnip1-001	0.0306765	1.20732	1.20732
Bok	Bok-001	0.000680626	0.660397	-1.51424

Bola1	Bola1-002	0.0273724	1.51406	1.51406
Bola3	Bola3-002	0.0294848	1.59099	1.59099
Bpgm	Bpgm-001	0.0131368	1.46071	1.46071
Bpgm	Bpgm-002	0.0145344	1.51916	1.51916
Bpgm	Bpgm-005	0.0392251	2.18388	2.18388
Bphl	Bphl-201	0.00220271	1.85189	1.85189
Bpi	Bpi-001	0.00537433	4.64212	4.64212
Bpi	Bpi-002	0.00537434	4.64212	4.64212
Bpnt1	Bpnt1-001	0.0260823	1.39343	1.39343
Bpnt1	Bpnt1-002	0.0230801	2.07774	2.07774
Bptf	Bptf-005	0.0025558	1.75985	1.75985
Braf	Braf-001	0.000527374	0.849255	-1.1775
Brap	Brap-005	0.0277302	0.363799	-2.74877
Brap	Brap-002	0.0150627	1.88388	1.88388
Brcc3	Brcc3-007	0.0441437	1.33169	1.33169
Brcc3	Brcc3-003	0.0151006	1.37631	1.37631
Brcc3	Brcc3-005	1.77162e-05	4.33085	4.33085
Brd2	Brd2-005	0.000887241	1.30361	1.30361
Brd2	Brd2-001	0.015904	1.3134	1.3134
Brd3	Brd3-202	0.0455277	1.75554	1.75554
Brd4	Brd4-004	0.0406284	1.14376	1.14376
Brd4	Brd4-003	0.0342295	1.18619	1.18619
Brd8	Brd8-003	0.00910915	1.33441	1.33441
Brdt	Brdt-002	0.0208975	2.88194	2.88194
Brf1	Brf1-201	0.00156478	1.29015	1.29015
Bri3	Bri3-001	0.0107877	0.602913	-1.65861
Bri3bp	Bri3bp-001	0.0045391	0.812047	-1.23146
Bri3bp	Bri3bp-002	0.000355805	1.43903	1.43903
Brinp2	Brinp2-001	0.00220215	0.55081	-1.81551
Brk1	Brk1-001	0.00910603	1.14801	1.14801
Brms1l	Brms1l-201	0.0104922	0.805948	-1.24078
Brsk1	Brsk1-002	0.0284886	0.490754	-2.03768
Brsk2	Brsk2-002	0.0188646	2.41282	2.41282
Brsk2	Brsk2-014	0.0402976	23.9554	23.9554
Brwd1	Brwd1-006	0.0199248	0.617439	-1.61959
Brwd1	Brwd1-001	0.00359253	0.782748	-1.27755
Brwd1	Brwd1-002	0.0153719	0.789407	-1.26677
Brwd3	Brwd3-001	0.00780898	0.808099	-1.23747
Brwd3	Brwd3-002	0.0297765	1.79598	1.79598
Bscl2	Bscl2-004	0.0463066	0.748565	-1.33589
Bscl2	Bscl2-007	0.0161202	1.67527	1.67527
Btaf1	Btaf1-001	0.0283388	1.32223	1.32223
Btbd1	Btbd1-201	0.00244977	0.764552	-1.30796

Btbd10	Btbd10-001	0.0149365	1.42007	1.42007
Btbd2	Btbd2-001	0.0148963	1.21791	1.21791
Btbd7	Btbd7-201	0.0455689	0.849665	-1.17693
Btbd8	Btbd8-001	0.0032205	1.68614	1.68614
Btg1	Btg1-201	0.0316671	1.31248	1.31248
Btg2	Btg2-001	0.0302958	0.627722	-1.59306
Btg3	Btg3-001	0.0151672	1.12201	1.12201
Bud13	Bud13-001	0.021185	1.31903	1.31903
Bud31	Bud31-005	0.00729371	1.77485	1.77485
Bves	Bves-201	5.04197e-05	1.61245	1.61245
C030034I22Rik	C030034I22Rik-001	0.0300631	2.12831	2.12831
C030034I22Rik	C030034I22Rik-201	0.0111748	2.20765	2.20765
C030037D09Rik	C030037D09Rik-001	0.0152778	1.22204	1.22204
C130060C02Rik	C130060C02Rik-201	0.0109711	3.58779	3.58779
C130080G10Rik	C130080G10Rik-002	0.0198262	5.3697	5.3697
C1d	C1d-004	0.040584	2.29364	2.29364
C1qa	C1qa-001	0.0236671	0.521219	-1.91858
C1qbp	C1qbp-003	0.023985	0.510975	-1.95704
C1ql3	C1ql3-001	0.0265352	0.682606	-1.46497
C1qtnf4	C1qtnf4-001	0.0333993	0.585557	-1.70778
C1qtnf6	C1qtnf6-001	0.0276945	0.799903	-1.25015
C1ra	C1ra-001	0.0265633	1.41574	1.41574
C1s1	C1s1-002	0.0139273	0.350691	-2.85151
C230035I16Rik	C230035I16Rik-002	0.0092525	3.66703	3.66703
C2cd3	C2cd3-001	0.00190701	1.50716	1.50716
C2cd3	C2cd3-201	0.0326647	3.72232	3.72232
C4a	C4a-002	0.0283483	38.8513	38.8513
C530043A13Rik	C530043A13Rik-003	0.0278776	5.04465	5.04465
C730034F03Rik	C730034F03Rik-201	0.00921342	1.66256	1.66256
C87436	C87436-002	0.0337356	0.211417	-4.73
C87436	C87436-006	0.0426963	1.58507	1.58507
C920009B18Rik	C920009B18Rik-001	0.0343268	0.589884	-1.69525
Cab39	Cab39-002	0.000896851	3.09768	3.09768
Cab39l	Cab39l-201	0.0010629	1.30853	1.30853
Cabp1	Cabp1-004	0.000230605	0.3877	-2.57931
Cabp1	Cabp1-006	0.0135225	0.441758	-2.26368
Cabp1	Cabp1-003	0.0200371	0.601905	-1.66139
Cabp1	Cabp1-007	0.0181868	1.14878	1.14878
Cabp1	Cabp1-002	0.00936928	2.95619	2.95619
Cacna1b	Cacna1b-007	0.000791781	0.126848	-7.88344
Cacna1b	Cacna1b-004	0.00907776	0.661341	-1.51208
Cacna1c	Cacna1c-006	0.043646	0.397954	-2.51285
Cacna1c	Cacna1c-001	0.00477258	0.447236	-2.23595

Cacna1e	Cacna1e-201	0.0120407	0.0167883	-59.5652
Cacna1g	Cacna1g-001	0.00148916	0.386802	-2.5853
Cacna1g	Cacna1g-201	0.0166136	0.511468	-1.95516
Cacna1g	Cacna1g-014	0.0375402	0.723264	-1.38262
Cacna1i	Cacna1i-001	0.00376154	0.683537	-1.46298
Cacnb1	Cacnb1-001	0.0399936	0.817719	-1.22291
Cacnb2	Cacnb2-010	0.00334474	1.93894e-07	-5.15745e+06
Cacnb3	Cacnb3-202	0.0379115	0.71884	-1.39113
Cacnb4	Cacnb4-001	0.0422668	1.4779	1.4779
Cacnb4	Cacnb4-003	0.0422676	1.4779	1.4779
Cacng4	Cacng4-001	0.0399667	0.793435	-1.26034
Cacng8	Cacng8-201	0.0383617	0.0677908	-14.7513
Cadm1	Cadm1-001	0.00814094	0.423014	-2.36399
Cadm1	Cadm1-003	0.0243302	0.724067	-1.38109
Cadm1	Cadm1-004	0.00458487	2.50048	2.50048
Cage1	Cage1-003	0.000894144	2.14025	2.14025
Cald1	Cald1-006	0.0274142	0.554654	-1.80292
Cald1	Cald1-001	0.00355379	0.594282	-1.6827
Cald1	Cald1-016	0.0444661	0.602152	-1.66071
Calm2	Calm2-001	0.00207796	0.908042	-1.10127
Calm3	Calm3-001	0.0437987	0.933509	-1.07123
Calm3	Calm3-004	0.0192245	2.54015	2.54015
Calu	Calu-009	0.0149969	0.564795	-1.77055
Calu	Calu-001	2.82703e-05	0.597937	-1.67242
Calu	Calu-002	0.000649128	0.641749	-1.55824
Camk2a	Camk2a-001	0.0324141	0.776321	-1.28813
Camk2b	Camk2b-003	0.0282858	0.506983	-1.97245
Camk2d	Camk2d-001	0.00847077	0.488421	-2.04742
Camk2d	Camk2d-009	0.0258872	0.597751	-1.67294
Camk2d	Camk2d-023	0.0498327	0.704839	-1.41876
Camk2d	Camk2d-004	0.0398168	0.782236	-1.27839
Camk2d	Camk2d-016	0.0231859	2.75324	2.75324
Camk2d	Camk2d-012	0.0354995	3.1013	3.1013
Camk2d	Camk2d-007	0.0480081	4.08327	4.08327
Camk2d	Camk2d-018	0.0455921	4.87485	4.87485
Camk4	Camk4-201	0.000178605	1.18106	1.18106
Camkk2	Camkk2-006	0.00536566	0.738087	-1.35485
Camkk2	Camkk2-005	0.00213888	3.22756	3.22756
Camkv	Camkv-001	0.044455	0.441617	-2.26441
Camsap1	Camsap1-001	0.00315875	0.655468	-1.52563
Camsap1	Camsap1-201	0.0143889	0.681038	-1.46835
Camsap1	Camsap1-002	0.0011573	7.32968	7.32968
Camsap2	Camsap2-004	0.0367392	0.388607	-2.5733

Camsap3	Camsap3-202	0.0330715	2.07156	2.07156
Camta1	Camta1-013	0.0191438	1.42621	1.42621
Camta1	Camta1-001	0.0162834	2.57764	2.57764
Camta2	Camta2-001	0.0341521	1.91286	1.91286
Cap1	Cap1-001	0.0141664	0.678464	-1.47392
Capn1	Capn1-201	0.00811512	0.439163	-2.27706
Capn10	Capn10-002	0.0165825	1.7239	1.7239
Capn2	Capn2-001	0.00326939	0.765952	-1.30556
Capn5	Capn5-001	0.00445665	0.630096	-1.58706
Capn6	Capn6-001	0.0154383	1.24445	1.24445
Capn7	Capn7-001	0.00737029	1.32201	1.32201
Caprin1	Caprin1-008	0.0399439	1.59866	1.59866
Caprin2	Caprin2-007	0.0300331	101.285	101.285
Capzb	Capzb-011	0.0155644	1.55118	1.55118
Car4	Car4-001	0.0397692	0.0041421	-241.423
Car8	Car8-001	0.0376663	0.524566	-1.90634
Carkd	Carkd-002	0.0119072	0.539775	-1.85262
Carnmt1	Carnmt1-201	0.031498	1.22223	1.22223
Cars	Cars-001	0.0223748	1.43374	1.43374
Cars	Cars-002	0.000107043	1.48509	1.48509
Casc3	Casc3-002	0.039867	0.354016	-2.82473
Casc3	Casc3-201	0.0480762	1.08545	1.08545
Casc4	Casc4-001	0.00843382	1.13594	1.13594
Cask	Cask-002	0.000713044	1.337	1.337
Casp2	Casp2-001	0.0296494	1.24449	1.24449
Casp3	Casp3-201	0.00409541	0.838631	-1.19242
Cast	Cast-201	0.0259196	1.18366	1.18366
Casz1	Casz1-001	0.0270324	1.61681	1.61681
Catsperd	Catsperd-201	0.049661	1.53996	1.53996
Catsperg1	Catsperg1-008	0.0362683	0.20112	-4.97217
Catsperg1	Catsperg1-011	0.016559	5.70439	5.70439
Catsperg2	Catsperg2-201	0.00124632	0.373788	-2.67531
Cbfa2t3	Cbfa2t3-003	0.00765713	2.55062	2.55062
Cbfb	Cbfb-006	0.00318494	1.84484e-09	-5.42053e+08
Cbl	Cbl-001	0.00479407	0.674658	-1.48223
Cbr1	Cbr1-001	0.00634711	1.35248	1.35248
Cbs	Cbs-003	0.040432	0.592845	-1.68678
Cbx3	Cbx3-001	0.0134853	2.05339	2.05339
Cbx3	Cbx3-007	0.0373483	27.2549	27.2549
Cbx4	Cbx4-001	0.00221165	1.42627	1.42627
Cbx4	Cbx4-002	0.000225981	1.49514	1.49514
Cbx7	Cbx7-007	0.0188955	0.809291	-1.23565
Cbx8	Cbx8-002	0.0100268	1.62885	1.62885

Cby1	Cby1-201	0.012435	1.6983	1.6983
Cc2d1b	Cc2d1b-001	0.00401719	0.605912	-1.6504
Cc2d1b	Cc2d1b-005	0.00185767	11.1002	11.1002
Ccar2	Ccar2-201	0.00739507	0.912313	-1.09612
Ccdc107	Ccdc107-001	0.0388405	1.41238	1.41238
Ccdc107	Ccdc107-002	0.0310831	1.52499	1.52499
Ccdc108	Ccdc108-001	0.0488658	2.70121	2.70121
Ccdc114	Ccdc114-001	0.0262025	16.7051	16.7051
Ccdc115	Ccdc115-001	0.00113668	1.36987	1.36987
Ccdc115	Ccdc115-002	0.0372139	1.47115	1.47115
Ccdc127	Ccdc127-001	0.000133985	1.46468	1.46468
Ccdc127	Ccdc127-005	0.00352211	1.47432	1.47432
Ccdc127	Ccdc127-002	0.00879556	4.50289	4.50289
Ccdc132	Ccdc132-002	0.0413553	0.467752	-2.13788
Ccdc132	Ccdc132-001	0.0471382	1.54817	1.54817
Ccdc132	Ccdc132-005	0.00918019	1.63449	1.63449
Ccdc134	Ccdc134-201	0.019663	1.53124	1.53124
Ccdc138	Ccdc138-201	0.00173128	1.31756	1.31756
Ccdc142	Ccdc142-001	0.022741	0.587744	-1.70142
Ccdc148	Ccdc148-001	0.000660836	2.27933	2.27933
Ccdc15	Ccdc15-201	0.0385614	1.23639	1.23639
Ccdc157	Ccdc157-001	0.0340238	1.31276	1.31276
Ccdc163	Ccdc163-004	0.0212349	2.15174	2.15174
Ccdc173	Ccdc173-001	0.0147405	1.50712	1.50712
Ccdc174	Ccdc174-002	0.0181418	1.49877	1.49877
Ccdc181	Ccdc181-001	0.0423771	1.22943	1.22943
Ccdc186	Ccdc186-001	0.00120625	1.14432	1.14432
Ccdc22	Ccdc22-001	0.0406925	0.853404	-1.17178
Ccdc23	Ccdc23-001	0.0256121	1.38704	1.38704
Ccdc24	Ccdc24-006	0.0421208	0.523968	-1.90851
Ccdc28a	Ccdc28a-004	0.0130607	2.17186	2.17186
Ccdc30	Ccdc30-010	0.0169884	5.39047	5.39047
Ccdc33	Ccdc33-009	0.020837	2.01361e-07	-4.9662e+06
Ccdc34	Ccdc34-004	0.0290596	0.430996	-2.32021
Ccdc34	Ccdc34-001	0.0368807	1.14841	1.14841
Ccdc38	Ccdc38-003	0.0142218	1.8802	1.8802
Ccdc39	Ccdc39-001	0.0306182	1.52691	1.52691
Ccdc39	Ccdc39-002	0.0188101	13.2772	13.2772
Ccdc43	Ccdc43-003	0.0401182	0.413881	-2.41615
Ccdc43	Ccdc43-002	0.0060729	2.0981	2.0981
Ccdc43	Ccdc43-004	7.23325e-05	2.80838	2.80838
Ccdc47	Ccdc47-001	0.0377229	1.26035	1.26035
Ccdc47	Ccdc47-003	0.0322709	1.29035	1.29035

Ccdc47	Ccdc47-005	0.00383691	1.31005	1.31005
Ccdc50	Ccdc50-003	0.0135034	2.12422	2.12422
Ccdc50	Ccdc50-004	0.000228143	5.65201	5.65201
Ccdc53	Ccdc53-007	0.000554118	1.4544	1.4544
Ccdc55	Ccdc55-001	0.00923533	1.3203	1.3203
Ccdc57	Ccdc57-001	0.0011093	1.51128	1.51128
Ccdc60	Ccdc60-001	0.00477263	17.4237	17.4237
Ccdc65	Ccdc65-201	0.0228664	2.62884	2.62884
Ccdc66	Ccdc66-201	0.00606969	1.46012	1.46012
Ccdc71l	Ccdc71l-001	0.0228349	0.49736	-2.01062
Ccdc73	Ccdc73-001	0.0295123	1.306	1.306
Ccdc74a	Ccdc74a-010	0.000359378	0.000112354	-8900.45
Ccdc74a	Ccdc74a-001	0.0210203	6.30337	6.30337
Ccdc78	Ccdc78-201	0.0474781	0.667077	-1.49908
Ccdc7b	Ccdc7b-001	0.0238972	0.00775224	-128.995
Ccdc84	Ccdc84-201	0.00219324	1.3651	1.3651
Ccdc88a	Ccdc88a-003	0.0213792	1.40168	1.40168
Ccdc9	Ccdc9-011	0.0489601	0.282912	-3.53467
Ccdc90b	Ccdc90b-001	0.00134584	1.31853	1.31853
Ccdc91	Ccdc91-003	0.0425683	0.204763	-4.8837
Ccdc93	Ccdc93-002	0.000609496	3.25294	3.25294
Cchcr1	Cchcr1-004	0.0120221	0.488521	-2.047
Cchcr1	Cchcr1-001	0.0491907	0.604197	-1.65509
Ccl2	Ccl2-001	0.00761385	0.130399	-7.66877
Ccl5	Ccl5-001	0.0478444	0.42404	-2.35827
Ccnc	Ccnc-002	0.0375833	2.17307	2.17307
Ccnd1	Ccnd1-002	0.00914689	4.96174	4.96174
Ccnd3	Ccnd3-005	0.024329	0.714158	-1.40025
Ccnd3	Ccnd3-012	0.0204084	4.1485	4.1485
Ccnd3	Ccnd3-015	0.0372001	5.626	5.626
Ccndbp1	Ccndbp1-002	0.0265575	0.471852	-2.11931
Ccng1	Ccng1-001	0.0264127	0.794479	-1.25869
Ccnh	Ccnh-003	0.0102759	1.3226	1.3226
Ccnk	Ccnk-201	0.000105578	1.42539	1.42539
Ccnl1	Ccnl1-013	0.00234426	14.914	14.914
Ccnt1	Ccnt1-006	0.0419106	1463.86	1463.86
Ccnt2	Ccnt2-002	0.00369947	1.1979	1.1979
Ccny	Ccny-201	0.000646111	1.34963	1.34963
Ccpg1	Ccpg1-010	0.00755348	3.12943	3.12943
Ccr2	Ccr2-003	0.0456752	6.11249e-07	-1.63599e+06
Ccr2	Ccr2-201	0.0383504	1743.96	1743.96
Ccrl2	Ccrl2-002	0.00685782	0.183851	-5.4392
Cct4	Cct4-007	0.00828763	0.451512	-2.21478

Cct7	Cct7-001	0.0247909	1.13779	1.13779
Cct7	Cct7-006	0.00511979	1.22678	1.22678
Cd151	Cd151-203	0.00065845	0.683783	-1.46245
Cd164	Cd164-001	0.000808483	0.723372	-1.38241
Cd209c	Cd209c-001	0.018666	1.8789	1.8789
Cd22	Cd22-007	0.016758	1.51904	1.51904
Cd248	Cd248-201	0.000630628	0.448889	-2.22772
Cd24a	Cd24a-201	0.00632617	0.756985	-1.32103
Cd274	Cd274-001	0.00279914	2.4438	2.4438
Cd2ap	Cd2ap-201	0.0235845	1.14298	1.14298
Cd2bp2	Cd2bp2-004	0.0481813	1.56269	1.56269
Cd300a	Cd300a-001	0.00245114	1.51901	1.51901
Cd300a	Cd300a-002	0.00244956	1.51904	1.51904
Cd40	Cd40-201	0.039652	0.645488	-1.54922
Cd81	Cd81-001	0.0395772	0.807675	-1.23812
Cdc123	Cdc123-005	0.0359621	1.53754	1.53754
Cdc25a	Cdc25a-003	0.0105387	0.69193	-1.44523
Cdc25c	Cdc25c-201	0.0121204	1.22854	1.22854
Cdc27	Cdc27-002	0.00404303	1.7777	1.7777
Cdc37	Cdc37-201	0.0416533	1.17038	1.17038
Cdc42	Cdc42-001	0.0295188	0.967907	-1.03316
Cdc42bpa	Cdc42bpa-009	0.0178605	0.366997	-2.72482
Cdc42bpa	Cdc42bpa-001	0.0121677	0.481315	-2.07764
Cdc42bpa	Cdc42bpa-004	0.0188232	1.55911	1.55911
Cdc42bpa	Cdc42bpa-008	0.023397	1.63508	1.63508
Cdc42bpa	Cdc42bpa-005	0.0204939	2.04864	2.04864
Cdc42bpa	Cdc42bpa-017	0.0112792	3.92138	3.92138
Cdc42bpg	Cdc42bpg-001	0.00287688	0.784137	-1.27529
Cdc42ep1	Cdc42ep1-201	0.041322	0.639714	-1.5632
Cdc42ep2	Cdc42ep2-201	0.0246116	0.438361	-2.28123
Cdc42ep4	Cdc42ep4-002	0.0332435	3.03163	3.03163
Cdc7	Cdc7-001	0.00561299	1.28409	1.28409
Cdc73	Cdc73-002	0.00335093	1.39948	1.39948
Cdc73	Cdc73-003	0.0492976	1.56033	1.56033
Cdca3	Cdca3-003	0.0478552	0.77073	-1.29747
Cdca7	Cdca7-003	0.0109629	0.289467	-3.45463
Cdca7l	Cdca7l-001	0.0133165	1.36195	1.36195
Cdca8	Cdca8-004	0.0120667	2.62261	2.62261
Cdh11	Cdh11-201	0.0224312	1.2111	1.2111
Cdh17	Cdh17-001	0.0012663	0.567954	-1.76071
Cdh24	Cdh24-201	0.0131362	1.38256	1.38256
Cdhr2	Cdhr2-001	0.0429574	1.68457	1.68457
Cdip1	Cdip1-002	0.024308	2.56583	2.56583

Cdipt	Cdipt-001	0.0237926	1.50166	1.50166
Cdk10	Cdk10-201	0.00498715	0.621514	-1.60897
Cdk13	Cdk13-201	0.000143754	0.699568	-1.42945
Cdk16	Cdk16-007	0.00472463	1.88055	1.88055
Cdk19	Cdk19-202	0.0287965	0.471407	-2.12131
Cdk20	Cdk20-201	0.00230829	2.30174	2.30174
Cdk2ap1	Cdk2ap1-002	0.0427085	2.58154	2.58154
Cdk4	Cdk4-004	0.0476311	0.508094	-1.96814
Cdk5	Cdk5-003	0.000418637	0.542165	-1.84446
Cdk5	Cdk5-001	0.00372667	0.589293	-1.69695
Cdk5rap1	Cdk5rap1-201	0.0232544	1.63295	1.63295
Cdk5rap2	Cdk5rap2-003	0.0303376	1.16699	1.16699
Cdk8	Cdk8-008	0.00310943	1.49755	1.49755
Cdk9	Cdk9-003	0.0463755	0.240918	-4.15079
Cdkal1	Cdkal1-001	0.0093122	1.26587	1.26587
Cdkl3	Cdkl3-009	0.00923624	0.176372	-5.66984
Cdkl3	Cdkl3-013	0.0241256	1.32801	1.32801
Cdkl4	Cdkl4-201	0.000499014	2.29405	2.29405
Cdkl5	Cdkl5-003	0.00272982	0.773022	-1.29362
Cdnf	Cdnf-001	0.00196744	1.98858	1.98858
Cdpf1	Cdpf1-001	0.01515	1.65519	1.65519
Cdr2l	Cdr2l-001	0.00994864	0.864069	-1.15731
Cds2	Cds2-002	0.00469687	0.671072	-1.49015
Cds2	Cds2-006	0.00217483	11.8189	11.8189
Cdsn	Cdsn-001	0.0388846	2.00145	2.00145
Cdyl	Cdyl-202	0.0155516	1.24628	1.24628
Cebpb	Cebpb-001	0.0176838	1.6485	1.6485
Cebpg	Cebpg-003	0.00757139	2.13489	2.13489
Cebpg	Cebpg-002	0.0053947	2.301	2.301
Cecr5	Cecr5-001	0.0138274	1.25337	1.25337
Cecr5	Cecr5-002	1.44221e-05	2.89646	2.89646
Cela1	Cela1-201	0.0005736	0.710606	-1.40725
Celf1	Celf1-010	0.0256426	0.459287	-2.17729
Celf3	Celf3-006	0.0402733	0.585801	-1.70706
Celf3	Celf3-009	0.01484	0.639124	-1.56464
Celf3	Celf3-007	0.0305276	1.29313	1.29313
Celf3	Celf3-013	0.00705584	4.65659	4.65659
Celsr3	Celsr3-005	0.000145425	0.734735	-1.36103
Cenpc1	Cenpc1-003	0.0348915	5.32003e-11	-1.87969e+10
Cenpe	Cenpe-001	0.0346486	1.17779	1.17779
Cenpk	Cenpk-201	0.0363461	1.43981	1.43981
Cenpl	Cenpl-005	0.0203414	0.21276	-4.70013
Cenpo	Cenpo-004	0.0329946	1.62481	1.62481

Cenpq	Cenpq-001	0.0392974	1.33266	1.33266
Cenpv	Cenpv-001	0.0439914	0.781857	-1.27901
Cep104	Cep104-001	0.023977	1.29001	1.29001
Cep112	Cep112-007	0.0296653	1.60992	1.60992
Cep162	Cep162-001	0.0191543	1.24001	1.24001
Cep162	Cep162-003	0.038837	2.14904	2.14904
Cep164	Cep164-001	0.0402815	0.893806	-1.11881
Cep170	Cep170-001	0.0445363	1.27068	1.27068
Cep170	Cep170-007	0.0378086	1.75986	1.75986
Cep170	Cep170-012	0.0058126	3.31608	3.31608
Cep19	Cep19-001	0.0180444	1.5223	1.5223
Cep19	Cep19-002	0.0441411	15.0263	15.0263
Cep192	Cep192-201	0.0211526	0.87907	-1.13757
Cep250	Cep250-005	0.0345457	1.32075	1.32075
Cep350	Cep350-001	0.0488799	0.895985	-1.11609
Cep350	Cep350-005	0.00650693	1.65433	1.65433
Cep57	Cep57-011	0.0437894	0.512056	-1.95291
Cep57	Cep57-012	0.00538061	2.7734	2.7734
Cep57l1	Cep57l1-002	0.0361916	1.81642	1.81642
Cep63	Cep63-002	0.0466888	1.68251	1.68251
Cep72	Cep72-201	0.00867376	1.34572	1.34572
Cep83os	Cep83os-201	0.0150752	1.59848	1.59848
Cep85l	Cep85l-201	0.00858821	1.3914	1.3914
Cep97	Cep97-001	4.6646e-05	0.703166	-1.42214
Cept1	Cept1-003	0.0462637	1.51072	1.51072
Cercam	Cercam-001	0.000661534	0.527208	-1.89678
Cerk	Cerk-003	0.0377428	0.536184	-1.86503
Cerk	Cerk-001	0.0477648	0.714755	-1.39908
Cers1	Cers1-201	0.00704349	1.19363	1.19363
Cers1	Cers1-202	0.00462933	1.22446	1.22446
Cers2	Cers2-001	0.013726	0.796673	-1.25522
Cers4	Cers4-001	0.000382471	0.533322	-1.87504
Cers4	Cers4-002	0.0384629	0.616644	-1.62168
Cers5	Cers5-002	0.0386174	0.450443	-2.22004
Cers5	Cers5-001	0.019129	0.833042	-1.20042
Cers6	Cers6-001	0.00119908	0.715006	-1.39859
Cetn4	Cetn4-002	0.0476346	0.16386	-6.10276
Cetn4	Cetn4-005	0.0245718	20.5149	20.5149
Cfap221	Cfap221-002	0.014723	7.69692	7.69692
Cfap43	Cfap43-201	0.00598152	0.742071	-1.34758
Cfap44	Cfap44-001	0.0480734	7.51247	7.51247
Cfap70	Cfap70-001	0.00599644	0.0407307	-24.5515
Cfap70	Cfap70-203	0.0247352	0.194827	-5.13275

Cfap70	Cfap70-201	0.00553875	6.13888	6.13888
Cfap74	Cfap74-201	0.00309514	0.0685131	-14.5958
Cfap74	Cfap74-007	0.00313822	0.0736799	-13.5722
Cfap74	Cfap74-004	0.0387218	2.14866	2.14866
Cfap97	Cfap97-201	0.00932795	1.37757	1.37757
Cfap97	Cfap97-004	0.0261411	5.47884	5.47884
Cfb	Cfb-001	0.0479896	913.042	913.042
Cflar	Cflar-003	0.000338326	0.852962	-1.17238
Cggbp1	Cggbp1-002	0.009803	1.49061	1.49061
Cgn	Cgn-001	0.00472648	2.16664	2.16664
Cgrrf1	Cgrrf1-002	0.0486317	3.42333	3.42333
Chac1	Chac1-001	3.20877e-05	3.24039	3.24039
Chac2	Chac2-001	0.0220532	1.33118	1.33118
Champ1	Champ1-002	0.0029369	2.15799	2.15799
Chchd3	Chchd3-005	0.0151715	0.742088	-1.34755
Chchd3	Chchd3-002	0.00302762	1.62216	1.62216
Chchd6	Chchd6-001	0.000267598	1.47203	1.47203
Chd3os	Chd3os-001	0.00238591	1.5132	1.5132
Chd4	Chd4-008	0.0168282	0.65829	-1.51909
Chd6	Chd6-003	0.0117974	0.909852	-1.09908
Chd6	Chd6-010	0.0171503	4.3666	4.3666
Chd7	Chd7-005	0.00297648	0.281995	-3.54616
Chd7	Chd7-001	0.00879946	0.741496	-1.34863
Chd7	Chd7-201	0.0452893	1.58194	1.58194
Chd8	Chd8-002	0.0148925	0.655211	-1.52623
Chek1	Chek1-006	0.00104463	2.77533	2.77533
Chek2	Chek2-002	0.000328922	1.51598	1.51598
Chfr	Chfr-011	0.0392178	0.471747	-2.11978
Chic2	Chic2-002	0.00038231	1.33047	1.33047
Chid1	Chid1-005	0.00529161	0.250017	-3.99973
Chkb	Chkb-001	0.0130783	0.538715	-1.85627
Chmp2b	Chmp2b-201	0.0250651	1.26927	1.26927
Chmp3	Chmp3-001	0.00217792	0.770126	-1.29849
Chmp3	Chmp3-002	0.0016938	1.91484	1.91484
Chmp3	Chmp3-008	0.0487139	2.40751	2.40751
Chn1	Chn1-201	0.0258625	0.728907	-1.37192
Chn2	Chn2-003	0.0402706	0.809842	-1.23481
Chn2	Chn2-002	0.00139056	1.59808	1.59808
Chn2	Chn2-006	0.00426387	2.28405	2.28405
Chp1	Chp1-005	0.0259909	5.70141e-06	-175395
Chrna1	Chrna1-001	0.00925351	1.27453	1.27453
Chst11	Chst11-001	8.04511e-06	0.380057	-2.63119
Chsy1	Chsy1-001	0.0194161	0.706506	-1.41542

Chtop	Chtop-009	0.0349333	3.20187	3.20187
Cib2	Cib2-001	0.0401309	2.04977	2.04977
Cic	Cic-004	0.0236892	0.797488	-1.25394
Cirbp	Cirbp-005	0.00335589	2.00943	2.00943
Cisd1	Cisd1-001	0.0127722	1.34599	1.34599
Cish	Cish-001	0.0230395	0.412115	-2.42651
Cish	Cish-003	0.0409712	0.696963	-1.4348
Cit	Cit-019	0.0177589	6.5683	6.5683
Cited2	Cited2-201	0.0494569	1.2688	1.2688
Ciz1	Ciz1-015	0.034643	2.1505	2.1505
Ckap2	Ckap2-201	6.08278e-06	1.22032	1.22032
Clasp1	Clasp1-022	0.000773795	0.737346	-1.35621
Clasp2	Clasp2-201	0.00646851	1.1067	1.1067
Clasp2	Clasp2-203	0.00469214	1.15991	1.15991
Clca2	Clca2-001	0.00385522	0.13522	-7.39533
Clcn2	Clcn2-005	0.0234379	0.22103	-4.52428
Clcn2	Clcn2-001	0.047462	0.352542	-2.83654
Clcn2	Clcn2-002	0.0404739	1.66785	1.66785
Clcn3	Clcn3-001	0.037691	0.844911	-1.18356
Clcn3	Clcn3-009	0.0150197	1.49482	1.49482
Clcn3	Clcn3-006	0.0251804	3.69491	3.69491
Clcn5	Clcn5-004	0.0438745	1.74172	1.74172
Clcn6	Clcn6-004	0.00954771	2.61026	2.61026
Cldn2	Cldn2-001	0.0454837	0.195075	-5.12622
Cldnd1	Cldnd1-003	0.0122042	23.7066	23.7066
Clec11a	Clec11a-001	0.0438314	1.89257	1.89257
Clec18a	Clec18a-002	0.0184052	0.143955	-6.94659
Clec2f	Clec2f-002	0.0483927	20.8977	20.8977
Clec2h	Clec2h-001	0.013044	0.471338	-2.12162
Clec2l	Clec2l-201	0.03851	1.425	1.425
Clic1	Clic1-001	1.73108e-05	0.670245	-1.49199
Clic4	Clic4-001	3.29563e-05	1.64659	1.64659
Clint1	Clint1-002	0.00763735	1.7077	1.7077
Clip1	Clip1-011	6.29098e-05	0.363612	-2.75018
Clip1	Clip1-009	0.0244582	0.446768	-2.2383
Clip1	Clip1-003	0.0322925	2.65768	2.65768
Clip2	Clip2-002	0.0498019	0.618122	-1.6178
Clip2	Clip2-001	0.0483064	0.746017	-1.34045
Clip4	Clip4-201	0.0100219	1.69317	1.69317
Clk1	Clk1-011	0.000950159	3.82625	3.82625
Clk2	Clk2-004	0.0485703	0.542532	-1.84321
Clk4	Clk4-004	0.0451311	0.511857	-1.95367
Cln3	Cln3-016	0.0325626	2.65156	2.65156

Cln5	Cln5-201	0.00356616	1.33979	1.33979
Cln8	Cln8-001	0.000688993	1.30296	1.30296
Cln8	Cln8-002	0.000903052	2.14646	2.14646
Clock	Clock-001	0.0353656	0.747631	-1.33756
Clpb	Clpb-003	0.00889584	1.26127	1.26127
Clpx	Clpx-003	0.0158804	3.03403	3.03403
Clstn1	Clstn1-002	0.0301817	1.29819	1.29819
Clstn3	Clstn3-001	0.00241697	1.51003	1.51003
Clu	Clu-001	0.049352	0.855439	-1.16899
Cluap1	Cluap1-003	0.043769	0.104755	-9.54612
Cluap1	Cluap1-001	0.00708906	1.54715	1.54715
Cluh	Cluh-003	0.0293265	8.88611	8.88611
Clvs1	Clvs1-006	0.037537	1.71521	1.71521
Clybl	Clybl-201	0.00419264	1.65717	1.65717
Cmas	Cmas-001	0.0235994	1.1718	1.1718
Cml1	Cml1-001	0.000219558	2.69377	2.69377
Cmss1	Cmss1-201	0.00116007	1.9656	1.9656
Cmtm4	Cmtm4-201	0.000157746	0.754672	-1.32508
Cmtm6	Cmtm6-201	0.00444134	0.803413	-1.24469
Cnbp	Cnbp-006	0.00379759	1.20016	1.20016
Cnep1r1	Cnep1r1-004	0.0154661	2.4903	2.4903
Cnga3	Cnga3-001	0.0409206	1.90671	1.90671
Cnga3	Cnga3-002	0.00908941	3.51469	3.51469
Cngb1	Cngb1-008	0.00489667	0.157943	-6.33139
Cnih1	Cnih1-001	0.0172754	0.825114	-1.21195
Cnih2	Cnih2-201	0.0166075	0.735059	-1.36044
Cnih4	Cnih4-004	0.000359072	0.361332	-2.76754
Cnih4	Cnih4-001	0.00051855	0.523718	-1.90943
Cnn2	Cnn2-002	0.0147678	0.495639	-2.0176
Cnn2	Cnn2-001	0.00034111	0.519169	-1.92615
Cnn3	Cnn3-004	0.0382346	0.114119	-8.76277
Cnn3	Cnn3-002	0.0299679	1.49766	1.49766
Cnnm2	Cnnm2-202	0.0218019	1.37744	1.37744
Cnot1	Cnot1-202	0.0425986	1.15359	1.15359
Cnot11	Cnot11-003	0.0455512	1.3046	1.3046
Cnot2	Cnot2-003	0.00851961	2.06149	2.06149
Cnot2	Cnot2-006	0.0407335	2.62226	2.62226
Cnot3	Cnot3-003	0.037084	1.41959	1.41959
Cnot4	Cnot4-003	0.025073	0.632573	-1.58085
Cnot4	Cnot4-004	0.00568619	2.12549	2.12549
Cnot6	Cnot6-001	0.000528926	0.53862	-1.8566
Cnot6l	Cnot6l-001	0.00662604	0.766112	-1.30529
Cnot8	Cnot8-001	0.0370441	1.15236	1.15236

Cnppd1	Cnppd1-002	0.0483063	1.28735	1.28735
Cnpy3	Cnpy3-002	0.00494091	2.2733	2.2733
Cnr2	Cnr2-002	0.0197668	1.36087	1.36087
Cnr2	Cnr2-001	0.019767	1.36088	1.36088
Cnst	Cnst-001	0.0119796	1.53907	1.53907
Cnst	Cnst-003	0.00102577	2.60281	2.60281
Cntln	Cntln-201	0.00110569	1.71115	1.71115
Cntnap2	Cntnap2-004	0.0111879	2.33598	2.33598
Cntrl	Cntrl-008	0.0111493	0.536938	-1.86241
Cntrl	Cntrl-001	0.00034722	1.70952	1.70952
Cntrob	Cntrob-001	0.0199688	1.21315	1.21315
Coa7	Coa7-005	0.0317166	11.4483	11.4483
Cog2	Cog2-001	0.000275784	1.36841	1.36841
Cog6	Cog6-001	1.84833e-06	1.46648	1.46648
Cog6	Cog6-002	0.013583	4.58156	4.58156
Cog8	Cog8-004	0.0116168	3.89231	3.89231
Col11a2	Col11a2-003	0.0426113	0.401412	-2.4912
Col14a1	Col14a1-202	0.0126148	0.307685	-3.25008
Col14a1	Col14a1-001	0.0126148	0.307685	-3.25008
Col14a1	Col14a1-201	0.0126148	0.307685	-3.25008
Col18a1	Col18a1-001	0.0420958	0.46183	-2.1653
Col22a1	Col22a1-001	0.000112884	0.477583	-2.09388
Col24a1	Col24a1-002	0.0284313	3481.53	3481.53
Col25a1	Col25a1-001	0.0434186	2.92991	2.92991
Col26a1	Col26a1-001	0.00264506	2.79271	2.79271
Col26a1	Col26a1-004	0.00264516	2.79271	2.79271
Col4a1	Col4a1-001	0.00051059	0.748926	-1.33525
Col4a2	Col4a2-001	0.00013971	0.597375	-1.67399
Col7a1	Col7a1-001	0.00207852	0.392896	-2.5452
Col7a1	Col7a1-201	0.0020786	0.392922	-2.54504
Col7a1	Col7a1-002	0.0457749	0.603678	-1.65651
Col9a3	Col9a3-005	0.0297233	0.141587	-7.06278
Commd1	Commd1-002	0.0369964	1.13494	1.13494
Commd10	Commd10-201	0.00266622	1.22827	1.22827
Commd2	Commd2-002	0.0290033	1.32742	1.32742
Commd3	Commd3-201	0.0268316	1.53713	1.53713
Commd7	Commd7-001	0.0326483	1.26341	1.26341
Commd7	Commd7-002	0.0150193	1.32886	1.32886
Commd8	Commd8-001	0.0408409	0.627825	-1.5928
Comt	Comt-001	0.0046745	1.27486	1.27486
Copa	Copa-001	0.00251227	1.27741	1.27741
Copa	Copa-006	0.000157541	1.32471	1.32471
Copb2	Copb2-001	0.000271065	1.2574	1.2574

Copg1	Copg1-001	0.0050667	1.33394	1.33394
Copg2	Copg2-001	0.0033623	2.6663	2.6663
Copg2	Copg2-007	0.0274851	51.4341	51.4341
Cops2	Cops2-001	0.0466588	0.80612	-1.24051
Cops2	Cops2-002	0.0069779	1.30233	1.30233
Cops3	Cops3-001	0.0102923	1.3995	1.3995
Cops3	Cops3-003	0.0144987	2.23483	2.23483
Cops4	Cops4-001	0.0453424	1.1369	1.1369
Cops7b	Cops7b-201	0.00870889	1.40343	1.40343
Copz1	Copz1-201	0.000725105	1.19031	1.19031
Coq2	Coq2-001	0.0458082	1.13617	1.13617
Coq3	Coq3-001	0.00590002	1.39789	1.39789
Coq4	Coq4-005	0.0195394	3.65199	3.65199
Coq5	Coq5-002	0.00136737	1.95063	1.95063
Coq9	Coq9-001	0.0405099	1.22706	1.22706
Corin	Corin-002	0.0378315	0.198553	-5.03644
Corin	Corin-001	0.0378315	0.198553	-5.03644
Coro1b	Coro1b-001	0.000177396	0.556308	-1.79756
Coro1c	Coro1c-002	0.0204227	0.655284	-1.52606
Coro1c	Coro1c-001	0.00159389	0.782077	-1.27865
Coro2a	Coro2a-001	0.00462593	1.31716	1.31716
Coro2a	Coro2a-201	0.0285481	1.44574	1.44574
Coro2a	Coro2a-002	0.0285675	1.44575	1.44575
Coro2b	Coro2b-001	0.0320367	0.848357	-1.17875
Coro7	Coro7-003	0.0301313	3.04327	3.04327
Coro7	Coro7-006	0.0228384	3.29454	3.29454
Cort	Cort-001	0.0192789	0.438203	-2.28205
Cox11	Cox11-002	0.017833	2.87375	2.87375
Cox14	Cox14-201	0.0169589	1.28097	1.28097
Cox16	Cox16-007	0.00473221	1.87212	1.87212
Cox17	Cox17-002	0.0130419	0.563604	-1.7743
Cox19	Cox19-007	0.0411773	1.55512	1.55512
Cox19	Cox19-006	0.0303518	6.11585	6.11585
Cox4i1	Cox4i1-001	0.0137485	0.922856	-1.08359
Cox6a1	Cox6a1-001	0.00352483	0.827845	-1.20796
Cox6a1	Cox6a1-002	0.0413679	2.49827	2.49827
Cox6a2	Cox6a2-001	0.00362524	2.35107	2.35107
Cox7b	Cox7b-003	0.0409854	1.13429	1.13429
Cox7b2	Cox7b2-002	0.0242054	0.78728	-1.2702
Cox7b2	Cox7b2-201	0.0242054	0.78728	-1.2702
Cpa4	Cpa4-001	0.0254745	1.38909	1.38909
Cpeb2	Cpeb2-006	0.0339235	2.00481	2.00481
Cpne1	Cpne1-008	0.0409088	17.1461	17.1461

Cpne1	Cpne1-019	0.0313144	4.35406e+06	4.35406e+06
Cpne5	Cpne5-004	5.4663e-05	0.587899	-1.70097
Cpne5	Cpne5-002	0.0014512	0.705112	-1.41821
Cpne7	Cpne7-003	0.0191622	0.415415	-2.40723
Cpne7	Cpne7-001	0.0240911	6.44438	6.44438
Cpne8	Cpne8-002	0.0103097	0.716957	-1.39478
Cpox	Cpox-201	1.53606e-05	1.83808	1.83808
Cpsf6	Cpsf6-001	0.00270183	0.887175	-1.12717
Cpsf6	Cpsf6-003	0.0338934	1.24709	1.24709
Cpt1c	Cpt1c-201	0.0229681	0.773354	-1.29307
Cpxm1	Cpxm1-002	0.00035699	26.1975	26.1975
Cr1l	Cr1l-004	0.00464671	1.51901	1.51901
Cr1l	Cr1l-005	0.00392808	1.67627	1.67627
Crabp2	Crabp2-001	0.0156902	1.75792	1.75792
Crat	Crat-006	0.026766	4.35278e-05	-22973.8
Crb1	Crb1-001	0.0126359	2.48566	2.48566
Crb2	Crb2-001	0.0237242	0.718762	-1.39128
Creb1	Creb1-001	0.00703642	0.713993	-1.40057
Creb1	Creb1-007	0.0161324	2.79813	2.79813
Creb3	Creb3-201	0.0184596	2.22644	2.22644
Crebzf	Crebzf-001	2.27763e-05	6.98762	6.98762
Creld1	Creld1-003	0.0339463	2.13676	2.13676
Crem	Crem-014	0.0033932	1.631	1.631
Crip1	Crip1-003	0.015526	0.389278	-2.56886
Crip1	Crip1-201	0.000130097	0.565974	-1.76687
Crip2	Crip2-003	0.0306339	0.564737	-1.77074
Crip2	Crip2-001	9.20154e-05	0.713381	-1.40178
Cript	Cript-001	0.00651029	1.27576	1.27576
Crk	Crk-001	0.0211862	0.751382	-1.33088
Crk	Crk-005	0.0156799	168.487	168.487
Crlf1	Crlf1-001	0.0357423	0.455853	-2.19369
Crlf3	Crlf3-001	0.00603733	1.50621	1.50621
Crlf3	Crlf3-006	0.0424827	3.35499	3.35499
Crls1	Crls1-003	0.00717295	1.63078	1.63078
Crls1	Crls1-001	0.0231277	1.65133	1.65133
Crmp1	Crmp1-003	0.0205292	0.855807	-1.16849
Crocc	Crocc-007	0.0498467	0.280635	-3.56334
Crtap	Crtap-001	0.0304445	0.7727	-1.29416
Crtc1	Crtc1-005	0.00615699	1.41148	1.41148
Crtc2	Crtc2-004	0.0481034	0.226603	-4.41299
Crtc2	Crtc2-006	0.00789311	1.85811	1.85811
Cry1	Cry1-201	0.000405085	1.5869	1.5869
Cry2	Cry2-001	0.000387844	1.72438	1.72438

Cryab	Cryab-201	0.0450305	0.748486	-1.33603
Cryz	Cryz-010	0.0130202	0.0329904	-30.3119
Cryzl1	Cryzl1-008	0.00173962	1.84859	1.84859
Cryzl1	Cryzl1-002	9.88248e-05	2.14662	2.14662
Cryzl1	Cryzl1-007	0.0396508	3.20697	3.20697
Csdc2	Csdc2-001	0.0481046	1.48147	1.48147
Csde1	Csde1-001	0.0176471	0.652234	-1.53319
Csde1	Csde1-002	0.023788	0.759786	-1.31616
Cse1l	Cse1l-005	0.0263026	0.602637	-1.65937
Cse1l	Cse1l-003	0.0156786	2.32379	2.32379
Csgalnact2	Csgalnact2-002	0.0328826	0.558357	-1.79097
Csgalnact2	Csgalnact2-001	0.00238607	1.26342	1.26342
Csnk1a1	Csnk1a1-001	0.0091931	0.794819	-1.25815
Csnk1a1	Csnk1a1-002	0.00955825	1.24451	1.24451
Csnk1d	Csnk1d-002	0.0202721	0.72521	-1.37891
Csnk1g1	Csnk1g1-001	0.0438756	0.838543	-1.19254
Csnk1g2	Csnk1g2-202	0.00580015	0.788169	-1.26876
Csnk2b	Csnk2b-008	0.0257845	2.07492	2.07492
Cspp1	Cspp1-015	0.0168218	0.273777	-3.6526
Csrnp3	Csrnp3-001	0.00768167	0.76354	-1.30969
Csrnp3	Csrnp3-004	0.0367017	4.16387	4.16387
Csrp1	Csrp1-001	0.00525689	0.710807	-1.40685
Csrp2bp	Csrp2bp-009	0.0262348	0.153827	-6.50082
Csrp2bp	Csrp2bp-002	0.025208	1.34904	1.34904
Cst3	Cst3-001	0.00850363	0.830576	-1.20398
Cstb	Cstb-201	0.0121465	0.812013	-1.23151
Cstf1	Cstf1-001	0.0147669	1.26985	1.26985
Cstf2	Cstf2-003	0.0353816	1.22974	1.22974
Cstf2	Cstf2-002	0.0133877	1.7601	1.7601
Cstf2	Cstf2-005	0.0435316	3.1679	3.1679
Cstf3	Cstf3-002	0.00340999	2.31498	2.31498
Ctage5	Ctage5-002	0.0314624	1.91221	1.91221
Ctage5	Ctage5-004	0.0417225	1.9958	1.9958
Ctage5	Ctage5-003	0.0138276	2.11375	2.11375
Ctbp1	Ctbp1-010	0.0311954	0.259943	-3.84699
Ctdsp2	Ctdsp2-202	0.0203119	1.22351	1.22351
Ctdspl2	Ctdspl2-006	0.0477892	0.779558	-1.28278
Ctgf	Ctgf-001	0.0133699	0.323929	-3.0871
Cth	Cth-001	3.18476e-05	4.3843	4.3843
Cth	Cth-003	0.0453703	6.78874	6.78874
Cthrc1	Cthrc1-001	0.00144943	0.827347	-1.20868
Ctnna1	Ctnna1-201	0.000433648	0.801266	-1.24802
Ctnnal1	Ctnnal1-001	0.0266373	0.775084	-1.29018

Ctnnal1	Ctnnal1-003	0.026327	1.90783	1.90783
Ctnnbip1	Ctnnbip1-002	0.00572075	3.55021	3.55021
Ctnnd1	Ctnnd1-020	0.0284046	0.340304	-2.93855
Ctnnd1	Ctnnd1-010	0.0315712	0.375804	-2.66096
Ctnnd1	Ctnnd1-001	0.0281769	0.82448	-1.21289
Ctns	Ctns-001	5.66381e-05	1.64666	1.64666
Ctps2	Ctps2-002	0.00557289	1.53041	1.53041
Ctr9	Ctr9-006	0.0438509	0.482166	-2.07398
Ctsa	Ctsa-004	0.0377851	0.599601	-1.66778
Ctsa	Ctsa-011	0.000455779	0.757815	-1.31958
Ctsb	Ctsb-201	0.00943738	0.874565	-1.14343
Ctsz	Ctsz-001	0.016608	0.895828	-1.11629
Cttn	Cttn-001	0.00323483	0.739302	-1.35263
Cttnbp2nl	Cttnbp2nl-002	0.00886371	0.705728	-1.41698
Ctu2	Ctu2-001	0.029602	0.573438	-1.74387
Cuedc1	Cuedc1-001	0.00136301	1.18286	1.18286
Cul1	Cul1-010	0.00815953	0.117159	-8.53541
Cul1	Cul1-003	0.000798111	12.5942	12.5942
Cul2	Cul2-003	0.000951369	1.33703	1.33703
Cul3	Cul3-002	0.00014611	1.29187	1.29187
Cul3	Cul3-004	0.0128816	2.06001	2.06001
Cul4a	Cul4a-001	0.0207419	0.787367	-1.27006
Cul4a	Cul4a-003	0.00644543	3.01301	3.01301
Cul4b	Cul4b-003	0.0113508	2.0312	2.0312
Cul5	Cul5-002	0.00034798	0.579983	-1.72419
Cul5	Cul5-001	0.00841346	1.44134	1.44134
Cul7	Cul7-002	0.0268747	0.72461	-1.38005
Cuta	Cuta-201	0.0136137	0.561924	-1.7796
Cutc	Cutc-002	0.0465238	2.08314	2.08314
Cux1	Cux1-013	0.0104222	0.191963	-5.20933
Cux1	Cux1-011	0.0307786	1.40734	1.40734
Cux1	Cux1-005	0.0309468	1.90535	1.90535
Cwc15	Cwc15-001	0.0127145	1.15134	1.15134
Cwc22	Cwc22-008	0.0439625	1.68664	1.68664
Cwc22	Cwc22-201	0.0264647	2.2134	2.2134
Cwc27	Cwc27-001	0.000789018	1.35172	1.35172
Cwf19l2	Cwf19l2-201	0.0295539	1.18395	1.18395
Cxadr	Cxadr-002	0.0449527	1.44972	1.44972
Cxcl1	Cxcl1-001	0.000637776	0.256383	-3.90041
Cxcl2	Cxcl2-002	0.0338588	0.382826	-2.61216
Cxcr2	Cxcr2-001	0.0381206	1.25664	1.25664
Cxxc4	Cxxc4-003	0.0318544	0.760782	-1.31444
Cxxc4	Cxxc4-201	0.0124101	1.66869	1.66869

Cyb5a	Cyb5a-001	0.00929691	3.94063	3.94063
Cyb5r1	Cyb5r1-003	0.00603859	1.8391	1.8391
Cyb5r1	Cyb5r1-001	0.00062857	2.31089	2.31089
Cyb5r1	Cyb5r1-008	0.00162051	4.2827	4.2827
Cyb5r2	Cyb5r2-201	0.00109681	2.14083	2.14083
Cyb5r3	Cyb5r3-003	0.0191539	0.625217	-1.59945
Cyb5r3	Cyb5r3-001	0.00172446	0.77425	-1.29157
Cyb5rl	Cyb5rl-201	0.0432208	3.21036	3.21036
Cyhr1	Cyhr1-001	0.0495288	0.788912	-1.26757
Cyld	Cyld-202	0.0459143	1.49369	1.49369
Cyld	Cyld-203	0.00554118	1.76734	1.76734
Cyld	Cyld-201	0.00245041	2.87316	2.87316
Cyp27a1	Cyp27a1-003	0.0125917	6.21026	6.21026
Cyp2c55	Cyp2c55-001	0.0138453	0.687753	-1.45401
Cyp2d22	Cyp2d22-201	0.000564381	0.156338	-6.39639
Cyp2j9	Cyp2j9-001	0.0175927	6.05374	6.05374
Cyp4b1	Cyp4b1-001	0.0149523	0.302976	-3.30059
Cyth4	Cyth4-201	0.00660976	0.435425	-2.29661
Cytip	Cytip-001	0.00488769	5.73794	5.73794
D030018L15Rik	D030018L15Rik-001	0.0316495	4.17004	4.17004
D030056L22Rik	D030056L22Rik-202	0.0235148	1.58798	1.58798
D15Ertd621e	D15Ertd621e-201	0.012043	0.761445	-1.31329
D16Ertd472e	D16Ertd472e-001	0.00165791	1.30775	1.30775
D16Ertd472e	D16Ertd472e-003	0.0225455	1.7766	1.7766
D17Wsu92e	D17Wsu92e-001	0.0034148	0.82115	-1.2178
D1Ertd622e	D1Ertd622e-002	0.0219495	0.750668	-1.33215
D2Wsu81e	D2Wsu81e-004	0.0474913	0.192682	-5.18989
D330041H03Rik	D330041H03Rik-001	0.0118925	1.32721	1.32721
D330041H03Rik	D330041H03Rik-004	0.0233337	1.83985	1.83985
D330041H03Rik	D330041H03Rik-002	0.0377082	1.96923	1.96923
D430042O09Rik	D430042O09Rik-004	0.0267451	1.79869	1.79869
D430042O09Rik	D430042O09Rik-006	0.00937926	2.66728	2.66728
D5Ertd579e	D5Ertd579e-005	0.0420768	1.77223	1.77223
D5Ertd579e	D5Ertd579e-007	0.0366683	1.88376	1.88376
D5Ertd615e	D5Ertd615e-002	0.0445854	0.504178	-1.98342
D630023F18Rik	D630023F18Rik-001	0.00806039	4.73543	4.73543
D6Wsu163e	D6Wsu163e-001	0.00764474	1.75718	1.75718
D6Wsu163e	D6Wsu163e-005	0.013434	3.12626	3.12626
D730003I15Rik	D730003I15Rik-002	0.00811937	2.11304	2.11304
D830030K20Rik	D830030K20Rik-001	0.0258312	0.643541	-1.5539
D830044I16Rik	D830044I16Rik-003	0.0244178	0.498058	-2.0078
D8Ertd82e	D8Ertd82e-001	0.0186058	1.23646	1.23646
D930028M14Rik	D930028M14Rik-001	0.00280358	2.58597	2.58597

Daam1	Daam1-201	9.53815e-05	0.604241	-1.65497
Daam2	Daam2-201	0.0217892	0.652875	-1.53169
Dab2ip	Dab2ip-001	0.0250645	0.741543	-1.34854
Dag1	Dag1-007	0.0141233	0.109499	-9.13248
Dag1	Dag1-002	0.0265987	0.36253	-2.75839
Dag1	Dag1-001	0.00138851	0.508028	-1.9684
Dagla	Dagla-001	0.0282711	1.64349	1.64349
Dalrd3	Dalrd3-005	0.0191724	0.0207648	-48.1584
Dalrd3	Dalrd3-001	0.00172853	0.524632	-1.9061
Dand5	Dand5-201	0.000237568	2.31924	2.31924
Dap3	Dap3-001	0.0246331	0.759228	-1.31713
Dap3	Dap3-011	0.0385744	1.86197	1.86197
Dap3	Dap3-007	0.0457608	5.20997	5.20997
Dars	Dars-001	0.0168106	1.15579	1.15579
Dazap1	Dazap1-005	0.0374655	1.21823	1.21823
Dazap1	Dazap1-002	0.00311417	1.4501	1.4501
Dazap2	Dazap2-201	1.90607e-05	0.481043	-2.07882
Dbf4	Dbf4-003	0.00178234	0.499208	-2.00317
Dbf4	Dbf4-006	0.0235229	0.540887	-1.84882
Dbhos	Dbhos-001	0.0298991	1.39049	1.39049
Dbn1	Dbn1-003	0.0328423	0.483005	-2.07037
Dbn1	Dbn1-010	0.038317	0.637715	-1.5681
Dbn1	Dbn1-002	0.0082623	0.640153	-1.56213
Dbn1	Dbn1-009	0.00306767	0.662096	-1.51036
Dbndd2	Dbndd2-003	0.0264352	0.711139	-1.40619
Dbt	Dbt-001	0.0367152	1.2665	1.2665
Dcaf11	Dcaf11-002	0.00470932	2.474	2.474
Dcaf12	Dcaf12-001	0.00159176	1.39597	1.39597
Dcaf17	Dcaf17-003	0.0409412	1.74118	1.74118
Dcakd	Dcakd-002	0.0154783	2.4134	2.4134
Dcbld2	Dcbld2-003	0.0356338	0.177229	-5.64241
Dcbld2	Dcbld2-001	0.000842623	0.547575	-1.82623
Dcdc2a	Dcdc2a-001	0.00340887	0.605437	-1.6517
Dcdc2b	Dcdc2b-201	0.00541575	2.83465	2.83465
Dcdc2c	Dcdc2c-001	0.0381398	1.21271	1.21271
Dchs1	Dchs1-001	0.00610947	0.715842	-1.39696
Dclk1	Dclk1-201	0.0483647	1.42257	1.42257
Dclk1	Dclk1-010	0.0422659	2.1658	2.1658
Dclk1	Dclk1-007	0.000131172	18.2932	18.2932
Dclk2	Dclk2-005	0.0306013	0.700791	-1.42696
Dclk2	Dclk2-003	0.0390395	0.908528	-1.10068
Dclre1b	Dclre1b-006	0.0231145	4.22603	4.22603
Dcp1a	Dcp1a-201	0.00434842	1.23168	1.23168

Dcp2	Dcp2-001	0.0309009	0.642369	-1.55674
Dcp2	Dcp2-002	0.0018992	5.39454	5.39454
Dcst1	Dcst1-004	0.00904689	2.42865	2.42865
Dcst1	Dcst1-006	0.00312782	10.5632	10.5632
Dctd	Dctd-006	0.014839	1.53781	1.53781
Dctd	Dctd-005	0.00616159	2.16135	2.16135
Dctn1	Dctn1-005	0.0204957	1.61308	1.61308
Dctn2	Dctn2-201	0.00435788	0.893783	-1.11884
Dctn3	Dctn3-001	0.000499929	0.807419	-1.23851
Dctn4	Dctn4-201	1.45161e-05	1.32428	1.32428
Dctn5	Dctn5-001	0.0134757	1.16732	1.16732
Dcun1d1	Dcun1d1-201	0.0157248	0.442107	-2.2619
Dcun1d3	Dcun1d3-003	0.000814975	1.84037	1.84037
Dcun1d4	Dcun1d4-002	0.00369944	0.516273	-1.93696
Dcun1d4	Dcun1d4-009	0.038623	11.989	11.989
Ddah1	Ddah1-002	0.00478677	2.41744	2.41744
Ddah2	Ddah2-001	0.000890575	0.60412	-1.6553
Ddb2	Ddb2-004	0.0139306	1.82311	1.82311
Ddhd1	Ddhd1-004	0.0146218	0.599653	-1.66763
Ddhd1	Ddhd1-009	0.0482988	0.727856	-1.3739
Ddi2	Ddi2-201	0.00850975	1.6657	1.6657
Ddr1	Ddr1-202	0.00345243	0.519928	-1.92334
Ddr1	Ddr1-002	0.00288855	0.531087	-1.88293
Ddr2	Ddr2-002	0.0168323	2.21038	2.21038
Ddr2	Ddr2-201	0.000168482	3.2512	3.2512
Ddt	Ddt-001	0.00017976	1.7213	1.7213
Ddx18	Ddx18-003	0.00772863	1.88068	1.88068
Ddx19a	Ddx19a-001	0.0433266	1.30285	1.30285
Ddx19b	Ddx19b-001	0.0004201	0.715758	-1.39712
Ddx19b	Ddx19b-002	0.00256154	4.61197	4.61197
Ddx20	Ddx20-001	0.0457322	1.42682	1.42682
Ddx25	Ddx25-001	0.0224737	1.57138	1.57138
Ddx26b	Ddx26b-006	0.0254226	0.2452	-4.0783
Ddx26b	Ddx26b-005	0.0122362	1.55704	1.55704
Ddx26b	Ddx26b-001	0.00170994	1.69111	1.69111
Ddx27	Ddx27-002	0.0108459	1.61252	1.61252
Ddx31	Ddx31-002	0.0409467	1.26842e-06	-788381
Ddx39b	Ddx39b-005	0.026127	1.18668	1.18668
Ddx3x	Ddx3x-003	0.0410893	2.9208	2.9208
Ddx47	Ddx47-005	0.0298827	0.504995	-1.98022
Ddx47	Ddx47-004	0.0358598	2.33687	2.33687
Ddx49	Ddx49-001	0.0313633	1.30416	1.30416
Ddx5	Ddx5-009	0.0435259	0.515629	-1.93938

Ddx5	Ddx5-001	0.0054639	0.675464	-1.48046
Ddx50	Ddx50-201	0.00247418	1.23901	1.23901
Ddx52	Ddx52-001	0.0322141	1.37066	1.37066
Ddx55	Ddx55-003	0.0374232	1.49473	1.49473
Ddx55	Ddx55-002	0.0240441	1.86349	1.86349
Deb1	Deb1-201	0.00545403	1.46387	1.46387
Decr2	Decr2-001	0.0387495	1.10912	1.10912
Def8	Def8-007	0.00132615	44.0618	44.0618
Degs1	Degs1-001	0.0223732	0.772167	-1.29506
Dennd2a	Dennd2a-001	0.00223901	0.680942	-1.46855
Dennd3	Dennd3-002	0.0407672	20.2141	20.2141
Dennd4a	Dennd4a-201	0.000615726	1.49918	1.49918
Dennd4b	Dennd4b-005	0.0357355	0.59812	-1.67191
Dennd4c	Dennd4c-003	0.00893153	2.07548	2.07548
Dennd5b	Dennd5b-002	0.0128693	3.02908	3.02908
Dennd6b	Dennd6b-201	0.000248914	1.43078	1.43078
Depdc1a	Depdc1a-001	0.0286492	1.2748	1.2748
Depdc5	Depdc5-001	0.0179181	1.57322	1.57322
Depdc5	Depdc5-015	0.000126691	2.34526	2.34526
Depdc5	Depdc5-006	0.0350966	2.43057	2.43057
Depdc7	Depdc7-001	0.00182043	1.38907	1.38907
Deptor	Deptor-003	0.0166674	4.7763	4.7763
Derl1	Derl1-201	9.83496e-05	1.24536	1.24536
Derl2	Derl2-001	0.00735139	0.776842	-1.28726
Derl2	Derl2-003	0.00556689	1.39717	1.39717
Derl2	Derl2-007	0.033484	1.6235	1.6235
Derl3	Derl3-201	0.0386342	0.290945	-3.43708
Des	Des-001	0.0118928	0.477989	-2.0921
Desi1	Desi1-006	0.0412305	5.7433	5.7433
Desi2	Desi2-001	9.32547e-05	1.42428	1.42428
Desi2	Desi2-002	0.000529294	1.78269	1.78269
Det1	Det1-003	0.0429412	1.3808	1.3808
Det1	Det1-001	0.0101646	1.68549	1.68549
Dffa	Dffa-001	0.00949388	1.30952	1.30952
Dfna5	Dfna5-201	0.0158999	2.61574	2.61574
Dfna5	Dfna5-003	0.0281695	73.3156	73.3156
Dgat1	Dgat1-001	0.0419919	0.752286	-1.32928
Dgat1	Dgat1-003	0.00483284	2.29203	2.29203
Dgat2	Dgat2-201	0.000100233	2.63373	2.63373
Dgcr2	Dgcr2-006	0.000164836	2.47411	2.47411
Dgcr6	Dgcr6-201	0.0127964	2.20894	2.20894
Dgcr6	Dgcr6-008	0.0473505	2.94488	2.94488
Dgcr6	Dgcr6-003	0.0269084	6.62164	6.62164

Dgcr8	Dgcr8-004	0.0146466	0.391565	-2.55386
Dgka	Dgka-201	0.0172144	0.595865	-1.67823
Dgkd	Dgkd-002	0.00467808	0.367729	-2.71939
Dgke	Dgke-001	0.0270934	0.581086	-1.72092
Dgki	Dgki-006	0.0125382	1.69593	1.69593
Dgkz	Dgkz-004	0.0228279	0.431531	-2.31733
Dgkz	Dgkz-001	0.0129792	0.666053	-1.50138
Dgkz	Dgkz-002	0.0214956	0.682225	-1.46579
Dhdds	Dhdds-001	0.0402544	1.76925	1.76925
Dhdds	Dhdds-002	0.0224585	2.15013	2.15013
Dhps	Dhps-001	0.0145513	0.88108	-1.13497
Dhps	Dhps-006	0.0238256	2.3974	2.3974
Dhrs7	Dhrs7-201	0.00074711	1.89242	1.89242
Dhx15	Dhx15-004	0.00473492	1.50396	1.50396
Dhx15	Dhx15-002	0.019069	2.42884	2.42884
Dhx34	Dhx34-003	0.0347892	0.0323325	-30.9287
Dhx38	Dhx38-201	0.00690397	1.23002	1.23002
Diap3	Diap3-201	0.00232778	0.642785	-1.55573
Dido1	Dido1-004	0.0100686	1.6429	1.6429
Dido1	Dido1-001	0.0229901	1.67987	1.67987
Dido1	Dido1-005	0.00903806	2.70279	2.70279
Diexf	Diexf-008	0.0329873	1.85003e+08	1.85003e+08
Dip2a	Dip2a-003	0.0320417	0.851177	-1.17484
Dip2b	Dip2b-201	0.0318311	0.5524	-1.81028
Dip2b	Dip2b-002	0.00628262	4.59216	4.59216
Dip2c	Dip2c-001	0.0262532	1.14544	1.14544
Dip2c	Dip2c-201	0.0192819	2.67142	2.67142
Diras2	Diras2-201	0.000689565	1.44993	1.44993
Disp1	Disp1-003	0.0460659	0.505256	-1.97919
Disp2	Disp2-002	0.00035992	0.613675	-1.62953
Disp2	Disp2-003	0.0183685	0.618	-1.61812
Disp2	Disp2-001	0.00676943	0.65295	-1.53151
Dixdc1	Dixdc1-008	0.0349086	16.8023	16.8023
Dkc1	Dkc1-004	0.0345124	0.0887967	-11.2617
Dkk3	Dkk3-201	0.0474009	1.11091	1.11091
Dlc1	Dlc1-007	0.00240813	0.0910497	-10.983
Dlg2	Dlg2-006	0.0487238	1.44896	1.44896
Dlg4	Dlg4-002	0.00837725	0.498383	-2.00649
Dlg4	Dlg4-012	0.0352943	0.529489	-1.88861
Dlg5	Dlg5-004	0.0444815	0.276302	-3.61923
Dlgap3	Dlgap3-001	0.0218565	0.806705	-1.23961
Dlx1	Dlx1-002	0.0142682	0.343706	-2.90946
Dlx1as	Dlx1as-002	0.0169019	3.4599	3.4599

Dmap1	Dmap1-002	0.0326575	0.402791	-2.48268
Dmap1	Dmap1-006	0.0407311	6.24257	6.24257
Dmbx1	Dmbx1-002	0.0465204	5.41004	5.41004
Dmbx1	Dmbx1-201	0.0465204	5.41004	5.41004
Dmpk	Dmpk-006	0.0371316	0.154391	-6.47706
Dmtf1	Dmtf1-009	0.0409179	2.11612	2.11612
Dmxl2	Dmxl2-001	0.0358882	1.62843	1.62843
Dmxl2	Dmxl2-008	0.0490146	3.73812	3.73812
Dnah3	Dnah3-201	0.0277354	4.66675	4.66675
Dnaic1	Dnaic1-003	0.00976032	74.866	74.866
Dnaja1	Dnaja1-201	0.0483216	0.383813	-2.60544
Dnaja1	Dnaja1-002	0.0110103	0.760012	-1.31577
Dnaja3	Dnaja3-002	0.000985851	1.41633	1.41633
Dnaja3	Dnaja3-001	0.00047122	2.37958	2.37958
Dnajb14	Dnajb14-001	0.0021316	0.745311	-1.34172
Dnajb2	Dnajb2-008	0.0399424	0.360422	-2.77452
Dnajb2	Dnajb2-003	0.0499911	1.89587	1.89587
Dnajb4	Dnajb4-006	0.00140123	1.40144	1.40144
Dnajb4	Dnajb4-001	0.0301195	5.07897	5.07897
Dnajc1	Dnajc1-006	0.00266831	9.00941	9.00941
Dnajc12	Dnajc12-001	0.00759554	2.09168	2.09168
Dnajc15	Dnajc15-201	0.0415273	1.25094	1.25094
Dnajc16	Dnajc16-001	0.0235257	1.24133	1.24133
Dnajc19	Dnajc19-001	0.0158861	1.59433	1.59433
Dnajc2	Dnajc2-006	0.0341595	1.30642	1.30642
Dnajc21	Dnajc21-001	0.00301836	1.29497	1.29497
Dnajc24	Dnajc24-003	0.0287535	4.93553	4.93553
Dnajc27	Dnajc27-202	0.00516535	2.78804	2.78804
Dnajc28	Dnajc28-201	0.0295201	1.66597	1.66597
Dnajc5	Dnajc5-004	0.0272088	2.88996	2.88996
Dnajc7	Dnajc7-004	0.0414417	1.19694	1.19694
Dnajc7	Dnajc7-010	0.0419579	1.44687	1.44687
Dnase2b	Dnase2b-002	0.0497806	0.170332	-5.87088
Dner	Dner-003	0.0487831	0.117661	-8.499
Dnm2	Dnm2-008	0.00858275	2.59093	2.59093
Dnmt1	Dnmt1-203	0.040861	0.649673	-1.53924
Dnmt3a	Dnmt3a-201	0.0106967	0.0909963	-10.9895
Dnmt3a	Dnmt3a-001	0.00109204	0.636907	-1.57009
Dnmt3a	Dnmt3a-008	0.0362447	2.28073	2.28073
Dnmt3a	Dnmt3a-005	0.0345577	6.99716	6.99716
Dnmt3a	Dnmt3a-009	0.0115776	12.2233	12.2233
Dnmt3b	Dnmt3b-008	0.00480953	3.56514	3.56514
Dnpep	Dnpep-006	0.012945	0.521213	-1.9186

Dnpep	Dnpep-004	0.010905	0.850288	-1.17607
Dock3	Dock3-002	0.00324519	2.93556	2.93556
Dock3	Dock3-011	0.0446387	21.1698	21.1698
Dock4	Dock4-201	0.00152067	0.595192	-1.68013
Dock7	Dock7-002	0.0149368	0.392881	-2.5453
Dock8	Dock8-201	0.00168949	3.9658	3.9658
Dopey1	Dopey1-004	0.00854934	1.67949	1.67949
Dopey1	Dopey1-002	0.0060438	7.46068	7.46068
Dot1l	Dot1l-006	0.0160937	6.20402	6.20402
Dpcd	Dpcd-201	0.0319019	1.14567	1.14567
Dpf1	Dpf1-008	0.012771	0.123966	-8.06674
Dpf2	Dpf2-003	0.0269003	5.8435	5.8435
Dpf3	Dpf3-203	0.0055256	0.224446	-4.45541
Dpf3	Dpf3-202	0.0483239	1.23453	1.23453
Dph3	Dph3-003	0.019112	0.0531883	-18.8011
Dph5	Dph5-002	0.0261811	1.81094	1.81094
Dph7	Dph7-003	0.0138553	1.92971	1.92971
Dph7	Dph7-004	0.0149625	8.24468	8.24468
Dpm1	Dpm1-006	0.0341459	2.73453	2.73453
Dpp7	Dpp7-001	0.00232932	0.463193	-2.15893
Dppa4	Dppa4-202	0.0125411	0.469427	-2.13026
Dpy19l1	Dpy19l1-001	0.00794329	0.36853	-2.71348
Dpyd	Dpyd-005	0.0346303	0.218936	-4.56754
Dpysl2	Dpysl2-001	0.000713007	0.793972	-1.25949
Dpysl3	Dpysl3-006	0.0215813	0.331613	-3.01556
Dpysl3	Dpysl3-001	2.89139e-06	0.456188	-2.19208
Dpysl3	Dpysl3-002	0.0117496	0.594278	-1.68271
Dpysl5	Dpysl5-001	0.0123186	0.697156	-1.4344
Dram1	Dram1-001	0.0109821	5.89238	5.89238
Dram1	Dram1-002	0.0109821	5.89238	5.89238
Dram2	Dram2-003	0.00016008	2.22795	2.22795
Drosha	Drosha-001	0.0113317	1.56114	1.56114
Dscam	Dscam-001	0.00946044	1.38978	1.38978
Dscc1	Dscc1-003	0.02989	0.522363	-1.91438
Dst	Dst-001	0.00135706	0.13399	-7.46322
Dst	Dst-201	0.00405653	0.144292	-6.93037
Dst	Dst-013	0.00831823	0.166891	-5.99195
Dst	Dst-009	0.00275636	0.556962	-1.79545
Dst	Dst-016	0.0283418	0.70196	-1.42458
Dst	Dst-019	0.0289085	2.0893	2.0893
Dst	Dst-003	0.0175577	2.10543	2.10543
Dst	Dst-007	0.0453406	3.62293	3.62293
Dst	Dst-018	0.027604	4.72863	4.72863

Dstn	Dstn-001	0.0186379	0.843554	-1.18546
Dstyk	Dstyk-001	0.0421641	0.668706	-1.49542
Dtd1	Dtd1-001	0.00186385	1.82197	1.82197
Dtnb	Dtnb-023	0.0146744	0.0222463	-44.9512
Dtnb	Dtnb-005	0.0010325	2.30789	2.30789
Dtx2	Dtx2-003	0.0433943	11.9315	11.9315
Dus2	Dus2-001	0.00238558	1.39861	1.39861
Dus2	Dus2-003	0.017448	1.56423	1.56423
Dusp19	Dusp19-001	0.0241437	1.68168	1.68168
Dusp7	Dusp7-001	0.0192972	1.2978	1.2978
Dusp9	Dusp9-001	0.0100094	0.361709	-2.76465
Dvl2	Dvl2-006	0.0332955	1.79622	1.79622
Dvl2	Dvl2-201	0.0332955	1.79622	1.79622
Dync1h1	Dync1h1-006	0.0235939	0.0534858	-18.6965
Dync1h1	Dync1h1-005	0.0236947	0.422197	-2.36856
Dync1h1	Dync1h1-001	0.0293198	0.857061	-1.16678
Dync1i2	Dync1i2-006	0.0121077	0.800279	-1.24956
Dync1li2	Dync1li2-201	0.00512111	1.22317	1.22317
Dync2li1	Dync2li1-201	0.00500665	1.31539	1.31539
Dynll1	Dynll1-001	0.00794755	0.720783	-1.38738
Dynll2	Dynll2-201	0.0433413	1.47656	1.47656
Dynlrb1	Dynlrb1-001	0.00558101	1.17701	1.17701
Dynlt1b	Dynlt1b-201	0.018083	1.23264	1.23264
Dynlt1f	Dynlt1f-201	0.0392959	1.14568	1.14568
Dysf	Dysf-018	0.0130157	0.50103	-1.99589
Dyx1c1	Dyx1c1-002	0.0316842	3.41199	3.41199
E230016M11Rik	E230016M11Rik-001	0.00380879	7.3823	7.3823
E2f3	E2f3-002	0.0126517	4.30152	4.30152
E2f6	E2f6-201	0.0094347	1.3722	1.3722
E2f7	E2f7-007	0.0432124	158.322	158.322
E330009J07Rik	E330009J07Rik-001	3.98373e-06	1.53077	1.53077
E430025E21Rik	E430025E21Rik-201	0.0439989	1.24956	1.24956
E4f1	E4f1-201	0.025839	1.21317	1.21317
Eaf1	Eaf1-201	0.00970537	0.787494	-1.26985
Eapp	Eapp-001	0.0102529	0.662887	-1.50855
Eapp	Eapp-201	0.000140374	0.729758	-1.37032
Eapp	Eapp-002	0.00208595	1.31935	1.31935
Eapp	Eapp-005	0.000190599	8.0427	8.0427
Ears2	Ears2-003	0.00388524	2.12122	2.12122
Ebf4	Ebf4-002	0.0240775	0.495469	-2.01829
Ebpl	Ebpl-201	0.0198457	1.29393	1.29393
Ecel1	Ecel1-004	0.0419494	0.436437	-2.29128
Ech1	Ech1-001	0.00125598	0.532053	-1.87951

Echdc1	Echdc1-002	0.0248277	1.27778	1.27778
Echdc1	Echdc1-001	0.0246336	1.30467	1.30467
Echdc1	Echdc1-003	0.0024113	4.79685	4.79685
Echdc2	Echdc2-004	0.00370249	2.08177	2.08177
Ecm1	Ecm1-002	0.0464595	0.274114	-3.64812
Ecm2	Ecm2-201	0.00111533	1.48342	1.48342
Ecscr	Ecscr-002	0.0306819	0.389516	-2.56729
Ect2	Ect2-004	0.0361399	1.85838	1.85838
Edc3	Edc3-001	0.0315049	1.15033	1.15033
Edem1	Edem1-004	0.0338101	1.31259	1.31259
Edem3	Edem3-004	0.0453788	4.6805	4.6805
Edf1	Edf1-002	0.00486999	1.86557	1.86557
Edrf1	Edrf1-003	0.00031156	1.26634	1.26634
Edrf1	Edrf1-001	0.0166145	1.44333	1.44333
Eea1	Eea1-201	0.0352725	1.12456	1.12456
Eed	Eed-001	0.00760392	1.10871	1.10871
Eef1b2	Eef1b2-003	0.0169716	0.570215	-1.75373
Eef1b2	Eef1b2-009	0.0395736	0.860792	-1.16172
Eefsec	Eefsec-002	0.00788061	1.27596	1.27596
Efcab14	Efcab14-005	0.0447955	0.509682	-1.96201
Efcab14	Efcab14-002	0.0084482	1.44701	1.44701
Efcab14	Efcab14-004	0.0219323	12.7731	12.7731
Efcab7	Efcab7-004	0.0250051	42374.2	42374.2
Efna4	Efna4-001	0.0287353	0.476403	-2.09906
Efr3a	Efr3a-001	0.000633661	1.61835	1.61835
Efr3a	Efr3a-002	0.0290441	1.69184	1.69184
Efr3a	Efr3a-007	0.0427062	5.18373	5.18373
Eftud1	Eftud1-001	0.00730028	0.660856	-1.51319
Eftud2	Eftud2-004	0.0361561	0.498104	-2.00761
Eftud2	Eftud2-002	0.00976862	1.35176	1.35176
Egfl6	Egfl6-001	0.0404853	3.18883	3.18883
Egfl7	Egfl7-016	0.0299443	0.0150555	-66.4211
Ehbp1l1	Ehbp1l1-202	0.00529695	0.60889	-1.64233
Ehd4	Ehd4-001	0.000788227	1.39945	1.39945
Ehmt1	Ehmt1-001	0.0489243	0.406604	-2.4594
Ehmt1	Ehmt1-015	0.0448556	1.17887	1.17887
Ehmt1	Ehmt1-012	0.0295261	3.26883	3.26883
Ehmt2	Ehmt2-001	0.0136473	0.621068	-1.61013
Ehmt2	Ehmt2-003	0.0152702	0.706512	-1.4154
Ei24	Ei24-008	0.0237441	0.500388	-1.99845
Eid2b	Eid2b-201	0.0470919	1.41294	1.41294
Eif1	Eif1-001	0.00767242	1.39963	1.39963
Eif1	Eif1-005	0.00839919	1.45417	1.45417

Eif1	Eif1-003	0.0021156	1.88629	1.88629
Eif1a	Eif1a-201	0.00738766	0.730325	-1.36925
Eif2ak2	Eif2ak2-002	0.0258641	0.129283	-7.73496
Eif2b3	Eif2b3-004	0.0403618	2.63363e+06	2.63363e+06
Eif2d	Eif2d-006	0.00832907	1.5618	1.5618
Eif2s2	Eif2s2-201	0.000107333	1.38018	1.38018
Eif2s2	Eif2s2-005	0.00930316	1.90321	1.90321
Eif2s2	Eif2s2-003	0.0275165	3.53232	3.53232
Eif2s3x	Eif2s3x-001	0.0378914	1.24028	1.24028
Eif3a	Eif3a-201	0.0295937	1.10131	1.10131
Eif3c	Eif3c-001	7.62787e-05	1.37552	1.37552
Eif3c	Eif3c-003	0.00160769	1.92524	1.92524
Eif3d	Eif3d-201	0.00263127	1.16165	1.16165
Eif3f	Eif3f-003	0.0336668	0.451441	-2.21513
Eif3g	Eif3g-201	0.0237152	1.26135	1.26135
Eif3i	Eif3i-002	0.0474219	1.84189	1.84189
Eif3i	Eif3i-004	0.0117487	3.18769e+08	3.18769e+08
Eif4a1	Eif4a1-013	0.0117369	5.84859	5.84859
Eif4e	Eif4e-001	0.000536075	1.13307	1.13307
Eif4e	Eif4e-005	0.000733141	1.22917	1.22917
Eif4e	Eif4e-003	0.0140645	1.63289	1.63289
Eif4e3	Eif4e3-001	0.00335535	0.861288	-1.16105
Eif4ebp1	Eif4ebp1-201	0.00023968	1.51234	1.51234
Eif4g1	Eif4g1-017	0.0075984	0.771384	-1.29637
Eif4g2	Eif4g2-012	0.00102133	0.23108	-4.32751
Eif4g2	Eif4g2-003	0.00876473	1.98938	1.98938
Eif4g3	Eif4g3-011	0.0324609	0.340229	-2.93919
Eif4h	Eif4h-003	0.0140479	0.634836	-1.57521
Eif4h	Eif4h-004	0.0360571	0.751626	-1.33045
Eif5	Eif5-201	0.00962104	1.43076	1.43076
Eif5a	Eif5a-012	0.0375934	1.72511	1.72511
Eif5b	Eif5b-003	0.014139	2.08223	2.08223
Eif6	Eif6-003	0.0173866	0.209159	-4.78106
Elac1	Elac1-001	0.0105034	1.45829	1.45829
Elavl2	Elavl2-001	0.0300793	0.637698	-1.56814
Elavl2	Elavl2-014	0.0140726	1.52996	1.52996
Elavl4	Elavl4-006	0.00246635	0.508898	-1.96503
Elf1	Elf1-201	0.0132182	0.45419	-2.20172
Elf2	Elf2-001	0.00293349	0.36899	-2.7101
Elf2	Elf2-012	0.0329178	0.466052	-2.14568
Elk3	Elk3-001	0.000149036	0.500036	-1.99986
Elk4	Elk4-002	0.012596	0.841219	-1.18875
Ell	Ell-201	0.00956333	1.33195	1.33195

Elmod2	Elmod2-001	0.00196373	0.683519	-1.46302
Elovl1	Elovl1-004	0.0368313	0.387508	-2.5806
Elovl4	Elovl4-001	0.010501	1.36817	1.36817
Elovl5	Elovl5-001	0.00517012	0.874634	-1.14334
Elp2	Elp2-201	0.00960758	1.30548	1.30548
Elp3	Elp3-201	0.00521239	1.25699	1.25699
Emb	Emb-001	0.00615824	1.18632	1.18632
Emc10	Emc10-002	0.0134687	0.794686	-1.25836
Emc10	Emc10-006	0.0112902	1.65863	1.65863
Emc9	Emc9-002	0.0132307	1.55545	1.55545
Emc9	Emc9-003	0.0272687	1.76531	1.76531
Emid1	Emid1-005	0.0272385	0.0213723	-46.7896
Emid1	Emid1-002	0.00527371	0.291112	-3.4351
Emid1	Emid1-001	0.00770428	0.570232	-1.75367
Emilin1	Emilin1-001	0.00754943	0.593787	-1.68411
Emilin3	Emilin3-001	0.0451322	3.47586	3.47586
Emilin3	Emilin3-201	0.0451322	3.47586	3.47586
Eml4	Eml4-202	0.0190382	1.31699	1.31699
Eml4	Eml4-203	0.0257062	1.40205	1.40205
Eml5	Eml5-201	0.00291538	0.858695	-1.16456
Eml6	Eml6-001	0.0407378	1.28237	1.28237
Emp1	Emp1-001	0.00866775	0.449907	-2.22268
Emp1	Emp1-201	4.32988e-05	0.506838	-1.97302
Emp3	Emp3-201	0.0246672	0.511272	-1.9559
Emx1	Emx1-001	0.0365887	5.00905e-06	-199639
Enah	Enah-004	0.00320515	1.79036	1.79036
Enah	Enah-005	0.000683467	1.85774	1.85774
Enah	Enah-002	3.68106e-05	2.34876	2.34876
Enc1	Enc1-201	0.00692118	0.623391	-1.60413
Endod1	Endod1-201	0.0381527	0.622986	-1.60517
Endov	Endov-011	0.025914	2.23132	2.23132
Endov	Endov-006	0.014824	6.34532	6.34532
Eng	Eng-005	0.0391304	0.637132	-1.56953
Engase	Engase-007	0.00276659	0.236148	-4.23463
Engase	Engase-005	0.00827166	1.83254	1.83254
Enho	Enho-001	0.0225795	1.31638	1.31638
Eno3	Eno3-201	0.0182503	0.437956	-2.28334
Enpp1	Enpp1-001	0.000180214	0.325244	-3.07461
Enpp3	Enpp3-201	0.000823786	0.432546	-2.3119
Enpp6	Enpp6-001	0.0395154	2.81167	2.81167
Enpp6	Enpp6-003	0.0283853	4.37377	4.37377
Enpp6	Enpp6-004	0.00949155	7.33191	7.33191
Ensa	Ensa-001	0.0140008	1.16492	1.16492

Enthd2	Enthd2-004	0.0191136	2.00632	2.00632
Entpd2	Entpd2-001	0.0168854	0.284464	-3.51538
Entpd6	Entpd6-003	0.030185	1.7013	1.7013
Entpd6	Entpd6-001	0.00931713	1.7233	1.7233
Entpd6	Entpd6-002	0.0260031	1.88075e+06	1.88075e+06
Entpd7	Entpd7-003	0.0145089	7.49175	7.49175
Epas1	Epas1-201	0.03466	1.21238	1.21238
Epb41	Epb41-012	0.0291566	2.64546	2.64546
Epb41l1	Epb41l1-008	0.00371304	0.074266	-13.4651
Epb41l1	Epb41l1-002	0.0273237	0.262742	-3.80602
Epb41l1	Epb41l1-004	0.0233315	1.80458	1.80458
Epb41l1	Epb41l1-003	0.0114869	2.12519	2.12519
Epb41l1	Epb41l1-012	0.012516	2.22078	2.22078
Epb41l1	Epb41l1-010	0.0229967	2.99699	2.99699
Epb41l5	Epb41l5-003	0.0110565	0.885916	-1.12878
Epc1	Epc1-001	0.0278183	0.569237	-1.75674
Epdr1	Epdr1-201	0.000964545	1.39353	1.39353
Epg5	Epg5-201	0.00815901	1.23251	1.23251
Epm2aip1	Epm2aip1-001	0.0466491	1.21511	1.21511
Epor	Epor-201	0.0197577	0.702568	-1.42335
Eprs	Eprs-001	3.03622e-07	1.69855	1.69855
Eprs	Eprs-003	0.00546938	3.56027	3.56027
Eps15	Eps15-006	0.0184994	0.581936	-1.7184
Eps15	Eps15-001	0.0256058	0.816194	-1.2252
Eps15	Eps15-008	0.0389168	8.66359	8.66359
Eps15	Eps15-010	0.0113995	62.3411	62.3411
Eps15l1	Eps15l1-201	0.00135027	1.19206	1.19206
Ept1	Ept1-001	0.0011123	0.665232	-1.50323
Ept1	Ept1-005	0.0375163	1.83891	1.83891
Erbb2ip	Erbb2ip-001	0.00349467	0.476217	-2.09988
Erbb2ip	Erbb2ip-002	0.00864254	0.644065	-1.55264
Erbb3	Erbb3-001	0.0136803	1.74714	1.74714
Erc2	Erc2-201	0.00100867	1.25974	1.25974
Ercc4	Ercc4-005	0.0285268	0.596061	-1.67768
Ercc4	Ercc4-004	0.00830476	0.694407	-1.44008
Ercc5	Ercc5-004	0.0207434	0.0294447	-33.962
Ercc6	Ercc6-201	0.00349922	1.22413	1.22413
Ercc6l2	Ercc6l2-003	0.0483102	1.60827	1.60827
Ergic3	Ergic3-008	0.0258979	8.9183e-05	-11212.9
Ergic3	Ergic3-001	0.000441829	0.70426	-1.41993
Eri3	Eri3-005	0.00836338	1.62282	1.62282
Erlin1	Erlin1-001	0.00701625	1.73046	1.73046
Erlin1	Erlin1-201	0.00602358	2.11145	2.11145

Ermap	Ermap-002	0.0235795	1.16444	1.16444
Ermap	Ermap-003	0.0235802	1.16444	1.16444
Ermp1	Ermp1-002	0.00382922	1.14669	1.14669
Ermp1	Ermp1-003	0.00598756	1.48808	1.48808
Ern1	Ern1-001	0.0448274	1.13495	1.13495
Ern1	Ern1-003	0.00756559	3.99072	3.99072
Ero1lb	Ero1lb-201	0.027666	1.3281	1.3281
Erp29	Erp29-001	0.01079	0.654281	-1.5284
Esco1	Esco1-001	0.00721702	1.3366	1.3366
Esco1	Esco1-003	0.0289961	3.55729	3.55729
Esd	Esd-006	0.0292131	0.663414	-1.50735
Esd	Esd-009	0.0135189	1.41708	1.41708
Esr1	Esr1-003	0.00728745	3.0798	3.0798
Esx1	Esx1-003	0.00941591	2.25446e-05	-44356.4
Esyt1	Esyt1-201	0.00276554	0.703523	-1.42142
Etfa	Etfa-001	0.04997	1.08607	1.08607
Etfdh	Etfdh-004	0.00967937	0.459898	-2.1744
Ethe1	Ethe1-001	0.00395165	1.36325	1.36325
Ets1	Ets1-001	0.00787575	0.689471	-1.45039
Etv3	Etv3-201	0.0263744	1.33128	1.33128
Etv4	Etv4-001	0.0131294	0.408261	-2.44941
Etv4	Etv4-007	0.039296	0.692885	-1.44324
Etv5	Etv5-001	0.000996748	1.25111	1.25111
Etv6	Etv6-005	0.0480895	84.9919	84.9919
Etv6	Etv6-004	0.0235759	865.596	865.596
Eva1b	Eva1b-003	0.0403254	0.225615	-4.43232
Eva1c	Eva1c-001	0.0290291	0.630564	-1.58588
Evi2a	Evi2a-004	0.00849924	0.185429	-5.39289
Evi5	Evi5-001	0.000251521	0.872796	-1.14574
Exd1	Exd1-001	0.0174322	1.54717	1.54717
Exoc2	Exoc2-201	0.00197114	1.12128	1.12128
Exoc3	Exoc3-201	0.0267701	1.18943	1.18943
Exoc4	Exoc4-004	0.00325759	1.45172	1.45172
Exoc4	Exoc4-002	0.0454195	2.763	2.763
Exoc5	Exoc5-001	0.00482787	0.596964	-1.67514
Exoc5	Exoc5-003	0.0411575	0.751832	-1.33009
Exoc6	Exoc6-201	0.00166116	1.24119	1.24119
Exoc6b	Exoc6b-001	0.0332985	1.17141	1.17141
Exoc7	Exoc7-003	0.0153742	0.388128	-2.57647
Exosc10	Exosc10-004	0.0204396	1.71726	1.71726
Exosc2	Exosc2-002	0.0106056	2.4587	2.4587
Exosc2	Exosc2-003	0.00628087	5.13827	5.13827
Exosc3	Exosc3-003	0.0270835	0.141762	-7.05406

Exosc7	Exosc7-003	0.0136052	1.9336	1.9336
Exosc8	Exosc8-003	0.0160024	1.48407	1.48407
Ext1	Ext1-002	0.0138422	0.574368	-1.74104
Ext1	Ext1-004	0.00127238	0.682592	-1.465
Ext1	Ext1-001	0.000448768	0.700096	-1.42837
Ext2	Ext2-001	0.0013337	1.2817	1.2817
Ext2	Ext2-005	0.00017249	13.586	13.586
Extl1	Extl1-001	0.000359738	2.10227	2.10227
Extl2	Extl2-006	9.89638e-05	0.841919	-1.18776
Extl3	Extl3-201	0.0037682	0.747992	-1.33691
Eya1	Eya1-002	0.0456971	0.00730462	-136.9
Eya3	Eya3-001	0.00176664	0.81047	-1.23385
Eya3	Eya3-002	0.0489796	1.95255	1.95255
Eya3	Eya3-004	0.0025029	2.60617	2.60617
Eya3	Eya3-003	0.00644356	7.63994	7.63994
F2r	F2r-201	7.80012e-05	0.554918	-1.80207
F2rl2	F2rl2-001	0.0357935	0.789888	-1.266
F730035M05Rik	F730035M05Rik-001	0.0473975	1.42544	1.42544
F830112A20Rik	F830112A20Rik-001	0.000209333	0.141563	-7.064
Fa2h	Fa2h-003	0.0344726	16.0984	16.0984
Faah	Faah-005	0.0385181	1.93353	1.93353
Faah	Faah-004	0.0442858	3.42158	3.42158
Fads3	Fads3-201	0.012966	0.841289	-1.18865
Faf2	Faf2-006	6.86537e-05	1.76297	1.76297
Fahd1	Fahd1-201	0.0314091	1.30607	1.30607
Faim2	Faim2-201	0.0294241	1.37085	1.37085
Fam114a2	Fam114a2-003	0.0191896	5.86879	5.86879
Fam117b	Fam117b-001	0.0485441	0.811771	-1.23187
Fam118a	Fam118a-201	0.0283286	1.17232	1.17232
Fam118b	Fam118b-003	0.0129618	1.95263	1.95263
Fam120a	Fam120a-201	0.0255002	0.813557	-1.22917
Fam122b	Fam122b-002	0.0247145	0.54409	-1.83793
Fam122b	Fam122b-001	0.00669719	0.569786	-1.75505
Fam124a	Fam124a-001	0.0110696	1.20318	1.20318
Fam126b	Fam126b-009	0.0051182	3.11903	3.11903
Fam129b	Fam129b-001	0.00105532	0.686623	-1.4564
Fam132a	Fam132a-003	0.00205655	5.48546	5.48546
Fam133b	Fam133b-002	0.0329677	1.21032	1.21032
Fam134b	Fam134b-201	0.0497256	1.29597	1.29597
Fam135a	Fam135a-002	0.0396061	1.75154	1.75154
Fam135a	Fam135a-005	0.0122127	4.18661	4.18661
Fam135b	Fam135b-201	0.0301894	1.69367	1.69367
Fam136a	Fam136a-003	0.0452954	4.32813	4.32813

Fam149a	Fam149a-003	0.000918389	1.53536	1.53536
Fam149b	Fam149b-202	0.0282125	3.21939	3.21939
Fam155a	Fam155a-201	0.0295241	0.533203	-1.87546
Fam160a2	Fam160a2-004	0.0215528	0.551993	-1.81162
Fam160b2	Fam160b2-201	0.0273521	0.782477	-1.27799
Fam161a	Fam161a-004	0.0262246	2.77786	2.77786
Fam161b	Fam161b-001	0.023169	1.70731	1.70731
Fam168b	Fam168b-003	0.00938209	2.58815	2.58815
Fam171a1	Fam171a1-004	0.00606902	0.403238	-2.47993
Fam171a1	Fam171a1-001	0.00151447	0.506336	-1.97497
Fam171a2	Fam171a2-001	0.0401233	0.725491	-1.37838
Fam171b	Fam171b-001	9.6271e-05	1.48145	1.48145
Fam173b	Fam173b-003	0.00712524	1.21954	1.21954
Fam173b	Fam173b-001	0.033399	1.39078	1.39078
Fam175a	Fam175a-007	0.0470905	1.57925	1.57925
Fam175a	Fam175a-201	0.0019464	2.01251	2.01251
Fam175a	Fam175a-006	0.0110706	2.31	2.31
Fam175a	Fam175a-004	0.00659993	3.22934	3.22934
Fam185a	Fam185a-001	0.00246735	0.663189	-1.50787
Fam186b	Fam186b-201	3.35896e-05	7.49003	7.49003
Fam188a	Fam188a-003	0.0139521	0.575171	-1.73861
Fam188a	Fam188a-015	0.00180276	1.90378	1.90378
Fam192a	Fam192a-201	0.0238271	1.21973	1.21973
Fam195b	Fam195b-001	0.000720862	1.36607	1.36607
Fam196b	Fam196b-202	0.00522113	4.59478	4.59478
Fam199x	Fam199x-001	0.00193495	1.14324	1.14324
Fam19a1	Fam19a1-001	0.0457628	0.293222	-3.41038
Fam19a4	Fam19a4-001	0.0197438	0.258569	-3.86745
Fam204a	Fam204a-201	0.000712283	1.55681	1.55681
Fam20b	Fam20b-004	0.0187018	0.510103	-1.96039
Fam212a	Fam212a-001	0.0382074	0.558024	-1.79204
Fam217b	Fam217b-001	0.0362749	0.836983	-1.19477
Fam220a	Fam220a-201	0.0171726	1.29057	1.29057
Fam220a	Fam220a-001	0.01716	1.29062	1.29062
Fam220a	Fam220a-004	0.01716	1.29062	1.29062
Fam227a	Fam227a-202	0.00887712	1.43911	1.43911
Fam228b	Fam228b-201	0.00487757	1.80816	1.80816
Fam229b	Fam229b-007	0.0310071	5.72521	5.72521
Fam229b	Fam229b-008	0.0310073	5.72522	5.72522
Fam234a	Fam234a-002	0.00698121	0.752	-1.32979
Fam234a	Fam234a-001	0.00384503	0.768917	-1.30053
Fam234b	Fam234b-002	0.0169939	1.11081e-05	-90024.3
Fam234b	Fam234b-001	0.000543907	1.37734	1.37734

Fam3c	Fam3c-001	0.000775038	0.421853	-2.3705
Fam3c	Fam3c-003	0.0220492	2.29091	2.29091
Fam3c	Fam3c-006	0.0280129	26.2756	26.2756
Fam46a	Fam46a-002	0.000282374	0.703169	-1.42213
Fam46b	Fam46b-001	0.0116774	3.67702	3.67702
Fam49b	Fam49b-201	0.0223456	0.591465	-1.69072
Fam50a	Fam50a-001	0.00305036	1.25154	1.25154
Fam53a	Fam53a-006	0.0124283	0.759186	-1.3172
Fam53b	Fam53b-003	0.00325629	7.99582	7.99582
Fam53b	Fam53b-007	0.00289506	10.8283	10.8283
Fam53c	Fam53c-202	0.0012628	1.24731	1.24731
Fam57b	Fam57b-004	0.00198301	0.672102	-1.48787
Fam58b	Fam58b-001	0.0449699	1.5118	1.5118
Fam60a	Fam60a-006	0.0196038	2.19619	2.19619
Fam60a	Fam60a-005	0.0110057	4.70421	4.70421
Fam63a	Fam63a-201	0.010327	0.57247	-1.74682
Fam69b	Fam69b-002	0.0268927	1.3624	1.3624
Fam71e1	Fam71e1-004	0.0250198	2.72353	2.72353
Fam73a	Fam73a-001	0.0271893	0.619768	-1.61351
Fam76b	Fam76b-001	0.0181693	0.671389	-1.48945
Fam78b	Fam78b-002	0.000722295	0.463996	-2.15519
Fam98b	Fam98b-001	0.0100684	1.62471	1.62471
Fam98b	Fam98b-002	0.00136095	2.52576	2.52576
Fam98c	Fam98c-002	0.0437174	2.86667	2.86667
Fanca	Fanca-009	0.0478395	0.00200906	-497.746
Fancb	Fancb-001	0.0304496	1.91843	1.91843
Fancd2	Fancd2-007	0.0253331	1.37709	1.37709
Fance	Fance-014	0.0442749	3.98677	3.98677
Fancl	Fancl-001	0.0124729	1.46032	1.46032
Fancl	Fancl-002	0.0329461	2.07274	2.07274
Far1	Far1-001	0.0120053	0.660606	-1.51376
Far1	Far1-005	0.00164541	8.62879	8.62879
Farp1	Farp1-004	0.0287165	0.669249	-1.49421
Farp1	Farp1-001	0.00389763	0.762309	-1.3118
Farp2	Farp2-001	0.0124857	2.31999	2.31999
Farsb	Farsb-001	0.00603085	1.46409	1.46409
Fas	Fas-201	0.0481833	0.555679	-1.7996
Fastk	Fastk-013	0.00661198	2.29802	2.29802
Fastk	Fastk-001	0.000140744	2.70961	2.70961
Fat2	Fat2-001	0.0480531	5.64582	5.64582
Fat2	Fat2-201	0.0480531	5.64582	5.64582
Fau	Fau-203	0.00698974	1.96698	1.96698
Faxc	Faxc-001	0.0229086	1.88003	1.88003

Fbf1	Fbf1-011	0.0141711	0.21101	-4.73912
Fbf1	Fbf1-201	0.00214787	0.641627	-1.55854
Fbf1	Fbf1-012	0.0103292	0.677525	-1.47596
Fblim1	Fblim1-001	0.0101236	0.365611	-2.73515
Fblim1	Fblim1-201	0.0401304	0.485446	-2.05996
Fbln1	Fbln1-002	0.000685971	0.574562	-1.74046
Fbln1	Fbln1-001	0.00574734	0.765845	-1.30575
Fbln2	Fbln2-003	0.0123456	0.578613	-1.72827
Fbln2	Fbln2-001	0.0323339	0.700633	-1.42728
Fbrs	Fbrs-008	0.019072	1.64515	1.64515
Fbrs	Fbrs-005	0.036481	3.16658	3.16658
Fbrsl1	Fbrsl1-201	0.0491749	0.767229	-1.30339
Fbxl12os	Fbxl12os-002	0.00550958	0.227037	-4.40456
Fbxl17	Fbxl17-005	0.0206555	0.279218	-3.58143
Fbxl18	Fbxl18-201	0.0340313	50.8854	50.8854
Fbxl19	Fbxl19-006	0.0137933	0.732564	-1.36507
Fbxl19	Fbxl19-003	0.0220558	0.748339	-1.33629
Fbxl5	Fbxl5-003	0.0185936	0.581595	-1.71941
Fbxo10	Fbxo10-001	0.042057	1.20332	1.20332
Fbxo16	Fbxo16-201	0.0205178	1.71083	1.71083
Fbxo17	Fbxo17-001	0.0140711	2.33332	2.33332
Fbxo18	Fbxo18-010	0.0435521	0.570894	-1.75164
Fbxo18	Fbxo18-001	0.0174959	1.10334	1.10334
Fbxo2	Fbxo2-001	0.0375372	0.660818	-1.51328
Fbxo21	Fbxo21-004	0.0397668	1.43904	1.43904
Fbxo22	Fbxo22-001	0.0321936	1.71353	1.71353
Fbxo24	Fbxo24-002	0.0406608	0.345214	-2.89675
Fbxo28	Fbxo28-001	0.00157796	0.573753	-1.74291
Fbxo36	Fbxo36-001	0.00181342	1.83599	1.83599
Fbxo47	Fbxo47-001	0.0158175	1.45938	1.45938
Fbxo6	Fbxo6-007	0.00534264	0.405096	-2.46855
Fbxo7	Fbxo7-006	0.0128859	8.16984	8.16984
Fbxo8	Fbxo8-001	0.00213324	1.21865	1.21865
Fbxo9	Fbxo9-007	0.0161825	2.5615	2.5615
Fbxw11	Fbxw11-004	0.0132052	0.136857	-7.30691
Fbxw11	Fbxw11-201	0.0448021	0.673154	-1.48555
Fbxw11	Fbxw11-001	0.0111026	0.787627	-1.26964
Fbxw5	Fbxw5-001	0.0204067	0.848394	-1.1787
Fbxw5	Fbxw5-006	0.0302592	1.68044	1.68044
Fcho1	Fcho1-009	0.0263807	1.84049	1.84049
Fcor	Fcor-001	0.0274877	2.45226	2.45226
Fem1b	Fem1b-201	0.0397469	0.858271	-1.16513
Fermt3	Fermt3-201	0.00701389	0.570201	-1.75377

Fes	Fes-006	0.0360358	0.6913	-1.44655
Ffar4	Ffar4-201	0.0394611	1.48763	1.48763
Fgd2	Fgd2-004	0.00558151	0.353767	-2.82672
Fgd2	Fgd2-001	0.0073858	0.462822	-2.16066
Fgd3	Fgd3-003	0.0427886	1.57383	1.57383
Fgd4	Fgd4-001	0.0355039	1.3411	1.3411
Fgd6	Fgd6-001	0.0160007	0.736206	-1.35831
Fgf13	Fgf13-001	0.0099305	0.359723	-2.77992
Fgf2os	Fgf2os-001	0.0462829	1.78489	1.78489
Fgf7	Fgf7-003	0.0103377	0.000394542	-2534.58
Fgfr1	Fgfr1-010	0.00894022	0.393266	-2.54281
Fgfr1	Fgfr1-003	0.0149522	0.627485	-1.59366
Fgfrl1	Fgfrl1-001	0.0141946	0.388527	-2.57382
Fgl1	Fgl1-001	0.00089249	0.234636	-4.26193
Fhl3	Fhl3-001	0.000128472	0.627355	-1.59399
Fhod1	Fhod1-001	0.0150451	0.620324	-1.61206
Ficd	Ficd-001	0.0131458	1.29236	1.29236
Fign	Fign-002	0.00350773	1.64817	1.64817
Fignl2	Fignl2-201	0.00668295	0.795877	-1.25648
Filip1	Filip1-002	0.00869544	2.14513	2.14513
Fip1l1	Fip1l1-003	0.000107615	0.481985	-2.07475
Fip1l1	Fip1l1-004	0.00251203	1.5306	1.5306
Fip1l1	Fip1l1-005	0.0331512	2.00638	2.00638
Fis1	Fis1-001	0.0402746	0.782304	-1.27828
Fis1	Fis1-003	0.0335068	2.4489	2.4489
Fkbp10	Fkbp10-001	0.044012	0.593227	-1.6857
Fkbp1a	Fkbp1a-010	0.0209268	0.510654	-1.95827
Fkbp2	Fkbp2-201	0.00703987	0.638203	-1.5669
Fkbp2	Fkbp2-202	0.0305757	0.709577	-1.40929
Fkbp7	Fkbp7-001	0.0125345	0.710287	-1.40788
Fkbp8	Fkbp8-010	0.00164194	0.630412	-1.58626
Fkbp9	Fkbp9-001	0.0232066	1.09207	1.09207
Fktn	Fktn-001	0.00826158	0.632201	-1.58177
Flcn	Flcn-005	0.0130072	1.40781	1.40781
Flcn	Flcn-004	0.00328413	1.73185	1.73185
Flii	Flii-001	0.0297894	0.886479	-1.12806
Flii	Flii-003	0.0317735	2.94043	2.94043
Flna	Flna-002	0.0285217	0.574028	-1.74208
Flna	Flna-006	0.0400265	0.58508	-1.70917
Flna	Flna-001	0.0136232	0.614271	-1.62794
Flna	Flna-003	0.0404701	0.630194	-1.58681
Flna	Flna-201	0.0159594	0.645211	-1.54988
Flna	Flna-011	0.0490784	0.735041	-1.36047

Flnb	Flnb-001	0.00507053	0.68226	-1.46572
Flrt1	Flrt1-201	0.00149602	1.46091	1.46091
Fmn1	Fmn1-201	0.000392404	0.361793	-2.76401
Fmnl1	Fmnl1-001	0.00076397	0.504498	-1.98217
Fmnl2	Fmnl2-004	0.0487463	0.564907	-1.7702
Fmnl2	Fmnl2-001	0.00172051	0.606307	-1.64933
Fmnl2	Fmnl2-002	0.0452711	0.810254	-1.23418
Fmr1	Fmr1-008	0.00853376	0.60766	-1.64566
Fn1	Fn1-018	0.000575258	0.107624	-9.29163
Fn1	Fn1-012	0.024156	0.192654	-5.19064
Fn1	Fn1-011	0.00519818	0.252449	-3.9612
Fn1	Fn1-017	0.00177871	0.32671	-3.06082
Fn1	Fn1-008	0.0014516	0.345928	-2.89078
Fn1	Fn1-007	3.68475e-05	0.352257	-2.83884
Fn1	Fn1-003	7.40566e-06	0.382942	-2.61136
Fn1	Fn1-001	0.00137602	0.454002	-2.20263
Fn1	Fn1-005	0.000910918	0.492065	-2.03225
Fn1	Fn1-002	0.0151491	0.493686	-2.02558
Fn1	Fn1-006	0.0114078	0.493862	-2.02486
Fn3krp	Fn3krp-001	0.0231434	1.28958	1.28958
Fnbp1	Fnbp1-201	0.0420344	0.0188901	-52.9379
Fnbp1	Fnbp1-015	0.000648647	0.0829622	-12.0537
Fnbp1	Fnbp1-001	0.000714327	0.127738	-7.82851
Fnbp1	Fnbp1-003	0.0236988	0.68744	-1.45467
Fnbp1l	Fnbp1l-005	0.0323187	0.844468	-1.18418
Fndc3b	Fndc3b-001	0.0263009	1.20306	1.20306
Fnip2	Fnip2-001	0.0318982	1.15238	1.15238
Fntb	Fntb-001	0.00988598	1.26944	1.26944
Fntb	Fntb-002	0.0274552	10.9068	10.9068
Folr1	Folr1-014	0.0336313	0.239613	-4.1734
Fos	Fos-002	0.0388652	32.0911	32.0911
Fosb	Fosb-201	0.0232391	2.11032	2.11032
Foxj1	Foxj1-001	0.0168626	0.645889	-1.54825
Foxj2	Foxj2-001	0.00780956	1.31519	1.31519
Foxk1	Foxk1-001	0.0366947	0.90413	-1.10604
Foxk2	Foxk2-001	0.0100579	0.858634	-1.16464
Foxn3	Foxn3-001	0.0201423	0.521463	-1.91768
Foxp1	Foxp1-032	0.00484521	1.84944	1.84944
Foxp4	Foxp4-006	0.0301394	1.80817	1.80817
Foxred2	Foxred2-001	0.00214887	1.38493	1.38493
Foxs1	Foxs1-001	0.0101382	0.179574	-5.56875
Fpgs	Fpgs-006	0.007495	2.70555	2.70555
Fra10ac1	Fra10ac1-201	0.0277419	1.35961	1.35961

Frmd4a	Frmd4a-007	0.00560968	0.58746	-1.70224
Frmd4a	Frmd4a-011	0.000570916	2.30783	2.30783
Frmd4a	Frmd4a-012	0.0327539	3.24972	3.24972
Frrs1	Frrs1-005	0.028978	1.85405	1.85405
Fry	Fry-004	0.000615193	30.2474	30.2474
Fryl	Fryl-009	0.0293405	0.275402	-3.63106
Fryl	Fryl-001	0.0132395	0.823884	-1.21376
Fsbp	Fsbp-001	0.0180888	0.61104	-1.63655
Fscn1	Fscn1-001	0.0370512	0.680793	-1.46887
Fscn1	Fscn1-002	0.0312782	0.727779	-1.37404
Fscn2	Fscn2-001	0.0172518	1.81186	1.81186
Fsd1l	Fsd1l-201	0.0480108	0.773475	-1.29287
Fsd2	Fsd2-001	0.00193936	2.77006	2.77006
Fst	Fst-201	0.0316608	0.585614	-1.70761
Fstl1	Fstl1-005	0.00109304	0.039299	-25.446
Fstl1	Fstl1-001	0.0471573	0.691342	-1.44646
Fstl4	Fstl4-001	0.0223845	1.94526	1.94526
Fth1	Fth1-201	0.00714294	0.853884	-1.17112
Ftl1	Ftl1-002	0.00705409	1.27118	1.27118
Ftl1	Ftl1-003	0.0297385	1.65118	1.65118
Fto	Fto-001	0.00772849	1.17491	1.17491
Ftx	Ftx-001	0.00929862	1.22831	1.22831
Fubp1	Fubp1-001	0.00354601	0.745017	-1.34225
Fubp1	Fubp1-005	0.00728679	1.69774	1.69774
Fubp1	Fubp1-002	0.0437055	1.70063	1.70063
Fubp1	Fubp1-016	0.0274261	4.40586	4.40586
Fubp3	Fubp3-001	0.00329333	1.29572	1.29572
Fuca1	Fuca1-002	0.0237162	0.343615	-2.91023
Fuca1	Fuca1-003	0.0244522	0.53463	-1.87045
Fuca1	Fuca1-001	1.92273e-06	0.608171	-1.64427
Fuca2	Fuca2-002	0.00202457	1.25212	1.25212
Fundc1	Fundc1-001	0.010844	1.28096	1.28096
Fundc2	Fundc2-003	0.0471186	1.42079	1.42079
Fuom	Fuom-002	0.0440359	1.62145	1.62145
Fuom	Fuom-013	0.0493215	2.49925	2.49925
Furin	Furin-201	0.000605206	0.515986	-1.93804
Fus	Fus-012	0.0223446	0.549009	-1.82146
Fus	Fus-015	0.0177186	0.624221	-1.602
Fuz	Fuz-201	0.00556141	0.692116	-1.44484
Fxn	Fxn-002	0.0234888	0.409045	-2.44472
Fxr1	Fxr1-001	0.001403	0.579467	-1.72572
Fxr1	Fxr1-005	0.00984318	1.40173	1.40173
Fyco1	Fyco1-201	0.0182344	1.39233	1.39233

Fyttd1	Fyttd1-009	0.0066123	1.57938	1.57938
Fzd4	Fzd4-201	0.00667213	1.36598	1.36598
Fzd7	Fzd7-001	0.00109714	0.59228	-1.68839
Fzr1	Fzr1-002	0.00466598	1.28877	1.28877
G0s2	G0s2-001	0.0195336	4.57458	4.57458
G2e3	G2e3-001	0.0144738	0.780777	-1.28077
G2e3	G2e3-002	0.0437211	1.66569	1.66569
G2e3	G2e3-003	0.0356283	3.1703	3.1703
G3bp1	G3bp1-002	0.00530862	1.51115	1.51115
G3bp2	G3bp2-201	0.00237916	0.475096	-2.10484
G3bp2	G3bp2-001	0.0218881	0.754085	-1.32611
G3bp2	G3bp2-003	0.0241439	1.7795	1.7795
G430095P16Rik	G430095P16Rik-001	0.000176531	2.46814	2.46814
G630016G05Rik	G630016G05Rik-001	0.0482797	2.41239	2.41239
G6pdx	G6pdx-004	0.0341928	1.67284	1.67284
Gabarapl1	Gabarapl1-004	0.00583228	32.962	32.962
Gabbr1	Gabbr1-002	0.0196472	0.631915	-1.58249
Gabpa	Gabpa-001	0.000966116	0.84326	-1.18587
Gabpa	Gabpa-002	0.0207057	1.48573	1.48573
Gabpa	Gabpa-004	0.0297641	18.4688	18.4688
Gabrb3	Gabrb3-006	0.0224691	0.336748	-2.96958
Gabrb3	Gabrb3-001	0.00859552	0.591783	-1.68981
Gabre	Gabre-003	0.00034924	3.29138	3.29138
Gadd45g	Gadd45g-002	0.00389205	0.287255	-3.48122
Gak	Gak-001	0.00095141	1.38524	1.38524
Gak	Gak-002	0.00228169	1.73267	1.73267
Gak	Gak-009	0.0414695	2.32321	2.32321
Gak	Gak-004	0.0134333	2.53228	2.53228
Galc	Galc-201	0.0204569	1.21921	1.21921
Galk2	Galk2-004	0.0298743	2591.05	2591.05
Galns	Galns-201	0.0493261	0.766451	-1.30472
Galnt1	Galnt1-201	0.000155148	0.634377	-1.57635
Galnt1	Galnt1-001	0.0280501	0.667224	-1.49875
Galnt11	Galnt11-004	0.0393239	1.84586	1.84586
Galnt15	Galnt15-001	0.0326697	0.254512	-3.92909
Galnt3	Galnt3-004	0.0430144	1.985	1.985
Galnt3	Galnt3-003	0.0280842	2.43426	2.43426
Gamt	Gamt-001	0.0121809	0.544994	-1.83488
Gapdh	Gapdh-013	0.0402132	0.0805854	-12.4092
Garem	Garem-201	0.0250735	0.840374	-1.18995
Garnl3	Garnl3-011	0.0433868	0.662512	-1.50941
Garnl3	Garnl3-008	0.0305934	0.727429	-1.37471
Gars	Gars-001	3.7732e-07	1.54298	1.54298

Gas5	Gas5-011	0.0252555	0.143494	-6.96893
Gata2	Gata2-004	0.0130181	3.52606	3.52606
Gata3	Gata3-001	0.0124175	1.66022	1.66022
Gata4	Gata4-005	0.0151086	0.230679	-4.33502
Gata4	Gata4-004	0.0151092	0.230681	-4.335
Gata4	Gata4-002	0.034053	1.36594	1.36594
Gatad2b	Gatad2b-201	0.00136777	3.80764	3.80764
Gatc	Gatc-001	0.0380947	1.30843	1.30843
Gba2	Gba2-001	0.0160276	0.699816	-1.42895
Gbe1	Gbe1-201	0.0072049	0.215234	-4.64611
Gbe1	Gbe1-203	0.03051	3.84653	3.84653
Gbf1	Gbf1-002	0.0144344	1.36277	1.36277
Gcat	Gcat-201	0.0181247	1.41882	1.41882
Gcfc2	Gcfc2-004	0.0270992	1.77351	1.77351
Gck	Gck-004	0.0264413	6.80776	6.80776
Gck	Gck-001	0.0264413	6.80776	6.80776
Gclc	Gclc-001	0.000189979	1.47143	1.47143
Gclm	Gclm-002	0.00727231	1.31663	1.31663
Gclm	Gclm-201	0.00333364	1.55865	1.55865
Gcnt7	Gcnt7-002	0.0346789	0.130821	-7.64403
Gdap1	Gdap1-003	0.0136781	0.0758092	-13.191
Gdap2	Gdap2-004	0.031608	2.23417	2.23417
Gdnf	Gdnf-201	0.036574	2.10019	2.10019
Gdpd3	Gdpd3-003	0.0250668	0.07856	-12.7291
Gdpd5	Gdpd5-201	0.0093225	0.560291	-1.78479
Gem	Gem-001	0.0147362	0.62421	-1.60203
Gemin2	Gemin2-003	0.0302258	0.491676	-2.03386
Gemin2	Gemin2-001	0.000386358	1.57341	1.57341
Gemin5	Gemin5-001	0.049978	1.5484	1.5484
Gemin7	Gemin7-001	0.00844224	0.201506	-4.96264
Get4	Get4-002	0.00223634	1.3967	1.3967
Gfm2	Gfm2-008	0.0297574	0.728786	-1.37214
Gfpt1	Gfpt1-001	0.0102053	1.13238	1.13238
Gfpt1	Gfpt1-002	0.00254934	4.9334	4.9334
Gfra2	Gfra2-201	0.00689474	0.600762	-1.66455
Gfra4	Gfra4-007	0.0496471	0.111342	-8.98135
Gfra4	Gfra4-008	0.0461751	2.87298	2.87298
Gga2	Gga2-004	0.000266472	1.39727	1.39727
Gga2	Gga2-003	0.0325986	1.41057	1.41057
Ggact	Ggact-001	0.00118169	1.74833	1.74833
Ggct	Ggct-003	0.0218397	2.36725	2.36725
Ggct	Ggct-005	0.0113692	4.09669	4.09669
Ggcx	Ggcx-001	0.00152765	1.53323	1.53323

Ggn	Ggn-202	0.0195382	1.61882	1.61882
Ggps1	Ggps1-201	0.0112484	1.15643	1.15643
Ggt7	Ggt7-004	0.0183808	0.339845	-2.94251
Ggt7	Ggt7-001	0.0112203	0.811998	-1.23153
Ghitm	Ghitm-201	0.0315069	1.60141	1.60141
Gigyf2	Gigyf2-006	0.0442914	1.79226	1.79226
Gin1	Gin1-004	0.048422	1.67785	1.67785
Ginm1	Ginm1-001	0.00668763	1.30876	1.30876
Gins1	Gins1-004	0.0157053	2.25143	2.25143
Git1	Git1-001	0.0226709	0.792853	-1.26127
Gja8	Gja8-001	0.0332549	6.66145	6.66145
Gjd2	Gjd2-001	0.000447444	3.83319	3.83319
Gk5	Gk5-001	0.00103963	1.68718	1.68718
Glb1	Glb1-201	0.000600394	0.764238	-1.30849
Glb1l	Glb1l-006	0.00919613	7.24353	7.24353
Glcci1	Glcci1-012	0.00398062	0.675934	-1.47943
Glce	Glce-001	0.0327801	1.67412	1.67412
Gldc	Gldc-201	0.00746744	0.784163	-1.27524
Glg1	Glg1-001	0.00724787	0.859242	-1.16382
Glis2	Glis2-001	0.00169179	0.401497	-2.49068
Glmp	Glmp-003	7.27806e-05	0.39633	-2.52315
Glmp	Glmp-009	0.0173679	0.435186	-2.29787
Glmp	Glmp-001	0.0105975	0.469567	-2.12962
Glmp	Glmp-010	0.0245059	0.483192	-2.06957
Glo1	Glo1-201	0.00877397	1.30443	1.30443
Glo1	Glo1-202	0.00411434	1.57138	1.57138
Glod4	Glod4-001	0.00369878	1.44469	1.44469
Gls	Gls-011	0.0233236	0.78323	-1.27676
Gls	Gls-002	0.00183859	1.37498	1.37498
Glt8d2	Glt8d2-007	0.0445773	0.0194214	-51.4897
Glt8d2	Glt8d2-001	0.0422753	2.31018	2.31018
Glud1	Glud1-001	0.0125179	0.732755	-1.36471
Glul	Glul-001	0.0052508	0.743262	-1.34542
Glyctk	Glyctk-001	0.0135139	2.50776	2.50776
Gm10109	Gm10109-001	0.00203598	9.98105	9.98105
Gm10222	Gm10222-001	0.018313	0.851279	-1.1747
Gm10244	Gm10244-001	0.00389163	3.3322	3.3322
Gm10277	Gm10277-001	0.000335506	0.319592	-3.12899
Gm10376	Gm10376-201	0.0340855	0.0277093	-36.089
Gm10409	Gm10409-001	0.00501253	1.29638	1.29638
Gm10439	Gm10439-001	1.4751e-05	0.75146	-1.33074
Gm10459	Gm10459-001	0.0377188	0.51602	-1.93791
Gm10545	Gm10545-001	0.0486336	2.34682	2.34682

Gm10548	Gm10548-001	0.0425926	2.78579	2.78579
Gm10557	Gm10557-201	0.00028124	1.53199	1.53199
Gm10662	Gm10662-001	0.0117343	2.34698	2.34698
Gm10699	Gm10699-201	0.0393649	1.93987	1.93987
Gm10709	Gm10709-201	0.000718329	1.84182	1.84182
Gm10785	Gm10785-001	0.0324475	1.37654	1.37654
Gm10800	Gm10800-201	0.0196143	2.69465	2.69465
Gm10925	Gm10925-001	0.0396392	0.884782	-1.13022
Gm11209	Gm11209-001	0.0351117	0.194513	-5.14104
Gm11400	Gm11400-001	0.0310664	1.21934	1.21934
Gm1141	Gm1141-002	0.0415278	3.20082e+07	3.20082e+07
Gm11410	Gm11410-001	0.0198389	0.598007	-1.67222
Gm11454	Gm11454-001	0.00184765	3.26286	3.26286
Gm11457	Gm11457-003	0.0389093	0.376268	-2.65768
Gm11491	Gm11491-001	0.0457391	0.344472	-2.903
Gm11510	Gm11510-001	0.0498375	0.327284	-3.05545
Gm11525	Gm11525-001	0.027548	1.36446	1.36446
Gm11638	Gm11638-001	0.00907547	2.64928	2.64928
Gm11736	Gm11736-001	0.0241782	2.9928	2.9928
Gm11739	Gm11739-001	0.00345424	0.372996	-2.681
Gm11744	Gm11744-202	0.0352872	2.82671e-05	-35376.8
Gm11748	Gm11748-001	0.00473786	3.0957	3.0957
Gm11766	Gm11766-001	0.0423613	1.58731	1.58731
Gm11772	Gm11772-001	0.0164066	2.44804	2.44804
Gm12060	Gm12060-001	0.0367376	2.99598	2.99598
Gm12196	Gm12196-001	0.00970798	21.3832	21.3832
Gm12258	Gm12258-001	0.0027656	2.19395	2.19395
Gm12404	Gm12404-001	0.01017	0.707944	-1.41254
Gm12454	Gm12454-001	0.0337127	2.57563	2.57563
Gm12743	Gm12743-001	0.0178606	2.00914	2.00914
Gm12762	Gm12762-001	0.00324383	1.56145	1.56145
Gm12784	Gm12784-001	0.0015486	2.41354	2.41354
Gm12847	Gm12847-001	0.00101378	20.6775	20.6775
Gm12932	Gm12932-001	0.00234892	3.48123	3.48123
Gm12940	Gm12940-004	0.0190445	0.664964	-1.50384
Gm12940	Gm12940-002	0.0167414	0.706213	-1.416
Gm13033	Gm13033-001	0.00788625	2.29708	2.29708
Gm13071	Gm13071-001	0.0329241	0.51942	-1.92522
Gm13111	Gm13111-001	3.53011e-07	0.360142	-2.77668
Gm13117	Gm13117-001	0.000503891	8.16158	8.16158
Gm13154	Gm13154-001	0.0141162	0.744178	-1.34376
Gm13214	Gm13214-001	0.00247867	6.02507	6.02507
Gm13230	Gm13230-001	0.00558392	0.574952	-1.73928

Gm13270	Gm13270-001	0.0109251	13.5961	13.5961
Gm13341	Gm13341-001	0.0446862	0.709807	-1.40883
Gm13370	Gm13370-001	0.0455933	0.046615	-21.4523
Gm13421	Gm13421-002	0.00384841	2.2359	2.2359
Gm13528	Gm13528-001	0.00519003	4.04774	4.04774
Gm13544	Gm13544-001	0.00773104	1.32412	1.32412
Gm13647	Gm13647-001	0.00154592	1.62598	1.62598
Gm13663	Gm13663-001	0.0112877	1.80308	1.80308
Gm13716	Gm13716-001	0.000161501	2.50532	2.50532
Gm13830	Gm13830-005	0.0322395	1.90136	1.90136
Gm13832	Gm13832-001	0.0436267	4.27112	4.27112
Gm13844	Gm13844-001	0.0235984	2.91242	2.91242
Gm13848	Gm13848-001	0.0111153	1.75684	1.75684
Gm14042	Gm14042-001	0.000267662	2.65807	2.65807
Gm14165	Gm14165-001	0.000266069	6.81659	6.81659
Gm14236	Gm14236-001	0.0204245	7.54337	7.54337
Gm14321	Gm14321-002	0.000438559	2.23662	2.23662
Gm14391	Gm14391-004	0.0489259	1.58951	1.58951
Gm14399	Gm14399-001	0.0321512	3.92239	3.92239
Gm14472	Gm14472-001	0.00748722	0.1607	-6.22279
Gm14480	Gm14480-001	0.0381692	12.3596	12.3596
Gm14540	Gm14540-001	0.0382316	0.169666	-5.89395
Gm14634	Gm14634-001	0.0154484	3.45331	3.45331
Gm14963	Gm14963-001	0.012441	1.82727	1.82727
Gm14966	Gm14966-002	0.0471062	2.0979	2.0979
Gm15091	Gm15091-001	0.0254646	0.736642	-1.35751
Gm15097	Gm15097-201	0.0292847	0.590707	-1.69289
Gm15107	Gm15107-001	0.0164587	0.838058	-1.19324
Gm15286	Gm15286-001	0.0124285	1.93232	1.93232
Gm15440	Gm15440-001	0.0104215	0.355089	-2.81619
Gm15443	Gm15443-001	0.0410888	1.79427	1.79427
Gm15446	Gm15446-002	0.00489616	1.42485	1.42485
Gm15446	Gm15446-201	0.0452423	1.99961	1.99961
Gm15487	Gm15487-001	0.0377804	0.269017	-3.71723
Gm15510	Gm15510-002	0.00993722	3.49849	3.49849
Gm15533	Gm15533-002	0.00728619	2.43658	2.43658
Gm15535	Gm15535-002	0.0151627	5.38264	5.38264
Gm15545	Gm15545-001	0.00944005	1.66448	1.66448
Gm15545	Gm15545-004	0.0376342	2.23391	2.23391
Gm15592	Gm15592-001	0.0328768	0.31522	-3.17239
Gm15651	Gm15651-001	0.0235523	3.62081	3.62081
Gm15788	Gm15788-001	0.0235073	3.30234	3.30234
Gm15800	Gm15800-001	0.0109403	0.896109	-1.11594

Gm15816	Gm15816-001	0.0366611	2.43158	2.43158
Gm15832	Gm15832-001	0.00402236	0.741687	-1.34828
Gm15834	Gm15834-002	0.00465783	1.86712	1.86712
Gm15847	Gm15847-001	0.00337071	3.40012	3.40012
Gm15850	Gm15850-001	0.00185396	16.8378	16.8378
Gm15880	Gm15880-001	0.0472664	2.83488	2.83488
Gm15898	Gm15898-001	0.0428303	0.428615	-2.3331
Gm15927	Gm15927-004	0.0189832	2.93638	2.93638
Gm15929	Gm15929-001	0.00532244	11.5921	11.5921
Gm15934	Gm15934-001	0.00102055	6.4772	6.4772
Gm16008	Gm16008-001	0.0190235	2.21144	2.21144
Gm16091	Gm16091-001	0.0294317	0.668961	-1.49485
Gm16133	Gm16133-002	0.0383977	5.03248	5.03248
Gm16153	Gm16153-001	0.0233447	1.26162	1.26162
Gm16181	Gm16181-001	0.0326733	0.510503	-1.95885
Gm16188	Gm16188-001	0.00643793	3.14422	3.14422
Gm16193	Gm16193-001	0.0451584	3.61851	3.61851
Gm16327	Gm16327-001	0.00729166	2.34445	2.34445
Gm16334	Gm16334-001	0.00385955	3.48874	3.48874
Gm16365	Gm16365-004	0.0499455	2.8756	2.8756
Gm16386	Gm16386-201	0.0165035	1.65847	1.65847
Gm16487	Gm16487-201	0.0277379	0.329693	-3.03313
Gm16536	Gm16536-001	0.0179974	0.858917	-1.16426
Gm16552	Gm16552-001	0.00190841	6.17926	6.17926
Gm16575	Gm16575-001	0.0388093	2.10344	2.10344
Gm16617	Gm16617-201	0.0197827	0.509561	-1.96247
Gm16701	Gm16701-001	0.00627084	1.43252	1.43252
Gm16712	Gm16712-201	0.00950247	1.3959	1.3959
Gm16907	Gm16907-201	0.0174619	0.606939	-1.64761
Gm17029	Gm17029-001	0.0105748	1.68926	1.68926
Gm17034	Gm17034-001	0.0147977	2.25917	2.25917
Gm17146	Gm17146-001	0.0245307	0.211907	-4.71904
Gm17195	Gm17195-001	0.0123971	0.601856	-1.66153
Gm17210	Gm17210-001	0.010929	0.350441	-2.85355
Gm17249	Gm17249-201	0.00805795	1.81238	1.81238
Gm17250	Gm17250-201	0.000521787	1.82324	1.82324
Gm17296	Gm17296-201	0.0265983	1.36663	1.36663
Gm17396	Gm17396-001	0.0252546	1.56576	1.56576
Gm17484	Gm17484-201	0.00715061	1.73345	1.73345
Gm17491	Gm17491-201	0.00501608	1.30771	1.30771
Gm17552	Gm17552-201	0.034507	1.91518	1.91518
Gm17586	Gm17586-201	0.0232608	2.0769	2.0769
Gm17733	Gm17733-001	0.00628243	1.97613	1.97613

Gm1947	Gm1947-001	0.00321351	0.492819	-2.02914
Gm19744	Gm19744-005	0.0324953	0.000141151	-7084.62
Gm19744	Gm19744-001	0.00480703	1.71189	1.71189
Gm19744	Gm19744-004	0.0115569	1.75175	1.75175
Gm19744	Gm19744-003	0.0101843	5.28366	5.28366
Gm20421	Gm20421-001	0.000399206	15.9601	15.9601
Gm20457	Gm20457-001	0.0171367	1.71939	1.71939
Gm20470	Gm20470-001	0.0489315	3.11464	3.11464
Gm20496	Gm20496-002	0.0462947	0.248622	-4.02216
Gm20498	Gm20498-003	0.0334837	1.36038	1.36038
Gm20507	Gm20507-001	0.0405313	1.98822	1.98822
Gm20517	Gm20517-001	0.0228046	3.21169	3.21169
Gm20541	Gm20541-001	0.0295412	1.45518	1.45518
Gm20559	Gm20559-001	0.036735	1.63109	1.63109
Gm20627	Gm20627-002	0.00287997	2.66803	2.66803
Gm20628	Gm20628-001	0.00214623	3.85005	3.85005
Gm20634	Gm20634-001	6.19798e-05	6.96883	6.96883
Gm20667	Gm20667-001	0.00148256	0.420107	-2.38035
Gm20684	Gm20684-001	0.0135872	2.03288	2.03288
Gm20695	Gm20695-002	0.042146	12.1947	12.1947
Gm20715	Gm20715-001	0.0275665	4.29908	4.29908
Gm20939	Gm20939-201	0.00532803	1.50752	1.50752
Gm21750	Gm21750-201	0.0317612	0.393145	-2.54359
Gm21769	Gm21769-201	0.028705	1.32738	1.32738
Gm21781	Gm21781-001	0.000558296	1.73931	1.73931
Gm21814	Gm21814-002	0.0113312	10.2795	10.2795
Gm21817	Gm21817-001	0.0166926	0.729602	-1.37061
Gm21887	Gm21887-202	0.0363352	0.537358	-1.86096
Gm22270	Gm22270-201	0.0181154	0.630014	-1.58727
Gm22455	Gm22455-201	0.00290842	2.78964	2.78964
Gm22613	Gm22613-201	0.0179613	0.214702	-4.65761
Gm22711	Gm22711-201	0.00206699	5.69789	5.69789
Gm23130	Gm23130-201	0.0307424	0.422329	-2.36782
Gm23442	Gm23442-201	0.00524697	8.60455	8.60455
Gm23455	Gm23455-201	0.0305498	0.584208	-1.71172
Gm24157	Gm24157-201	0.00392438	1.46848	1.46848
Gm26529	Gm26529-201	0.0428479	2.4405	2.4405
Gm26549	Gm26549-201	0.0408287	1.91106	1.91106
Gm26563	Gm26563-201	0.00123844	0.407362	-2.45482
Gm26564	Gm26564-201	0.03143	3.43778	3.43778
Gm26615	Gm26615-201	0.0233082	2.23755	2.23755
Gm26664	Gm26664-201	0.0459051	2.52118	2.52118
Gm26696	Gm26696-001	0.0056708	10.1434	10.1434

Gm26703	Gm26703-001	0.0116613	8.47628	8.47628
Gm26706	Gm26706-201	0.00512515	7.72948	7.72948
Gm26744	Gm26744-201	0.0327644	5.43632	5.43632
Gm26753	Gm26753-201	0.000503891	8.16158	8.16158
Gm26782	Gm26782-202	0.0281143	2.77312	2.77312
Gm26788	Gm26788-002	0.00112339	2.39042	2.39042
Gm26793	Gm26793-201	0.00876164	2.36347	2.36347
Gm26812	Gm26812-001	0.000219009	4.42341	4.42341
Gm26824	Gm26824-201	0.00741384	0.574437	-1.74083
Gm26839	Gm26839-201	0.0108992	2.29731	2.29731
Gm26848	Gm26848-201	0.0248878	1.50631	1.50631
Gm26868	Gm26868-201	0.00167462	1.6956	1.6956
Gm26885	Gm26885-201	0.0360138	1.34049	1.34049
Gm26886	Gm26886-001	0.0108813	1.18223	1.18223
Gm26888	Gm26888-201	0.000531611	2.07923	2.07923
Gm26947	Gm26947-001	0.0324319	1.60787	1.60787
Gm27029	Gm27029-001	0.0332457	0.296727	-3.3701
Gm27204	Gm27204-001	0.0145611	0.523614	-1.9098
Gm27223	Gm27223-001	0.0240303	1.3192e+06	1.3192e+06
Gm27517	Gm27517-201	0.0439002	6.03395	6.03395
Gm28036	Gm28036-001	9.05796e-05	1.61322	1.61322
Gm28037	Gm28037-001	0.003531	2.26527	2.26527
Gm28042	Gm28042-003	0.00689486	1.61499	1.61499
Gm28151	Gm28151-001	0.0317012	1.87725	1.87725
Gm28285	Gm28285-006	0.00630804	2.1564e-06	-463735
Gm28285	Gm28285-005	0.0019393	5.46315	5.46315
Gm28370	Gm28370-001	0.00412996	1.18509	1.18509
Gm28424	Gm28424-001	0.00277888	1.84731	1.84731
Gm28437	Gm28437-001	0.0037538	0.796822	-1.25499
Gm28439	Gm28439-001	0.00398853	0.824177	-1.21333
Gm28661	Gm28661-001	0.0318891	0.827957	-1.20779
Gm28875	Gm28875-001	0.0175378	0.388767	-2.57224
Gm28876	Gm28876-001	0.00824684	3.80311	3.80311
Gm28933	Gm28933-001	4.98387e-06	0.535251	-1.86828
Gm29170	Gm29170-001	0.00273246	1.56508	1.56508
Gm29245	Gm29245-001	0.0477925	0.315264	-3.17194
Gm29595	Gm29595-002	0.0106007	2.55347	2.55347
Gm29666	Gm29666-001	0.00176033	1.70679	1.70679
Gm3020	Gm3020-001	0.0130675	1.25937	1.25937
Gm32496	Gm32496-001	0.0384588	11.9559	11.9559
Gm33280	Gm33280-001	0.0442032	1.30957	1.30957
Gm34248	Gm34248-001	0.0327626	0.342072	-2.92336
Gm34882	Gm34882-001	0.0332959	9.43246	9.43246

Gm364	Gm364-001	0.00287853	0.774872	-1.29054
Gm3654	Gm3654-001	0.0486113	2.59877	2.59877
Gm36999	Gm36999-001	0.0233291	7.85105	7.85105
Gm37060	Gm37060-001	0.0148737	0.170575	-5.86251
Gm37074	Gm37074-001	0.000971092	2.34262	2.34262
Gm37090	Gm37090-001	0.000266017	1.53377	1.53377
Gm37110	Gm37110-001	0.02542	1.63929	1.63929
Gm37120	Gm37120-001	0.0464055	3.67669	3.67669
Gm3716	Gm3716-002	0.0158247	0.580712	-1.72202
Gm37214	Gm37214-001	0.0378593	1.74436	1.74436
Gm37264	Gm37264-001	0.0314274	1.57619	1.57619
Gm37271	Gm37271-001	0.00316658	4.66049	4.66049
Gm37296	Gm37296-001	0.0116654	0.282039	-3.5456
Gm37305	Gm37305-001	0.00503783	1.56787	1.56787
Gm37333	Gm37333-001	0.0301469	0.366654	-2.72736
Gm37334	Gm37334-001	0.00175207	0.225744	-4.42979
Gm37354	Gm37354-001	0.0181643	2.3214	2.3214
Gm37362	Gm37362-001	0.0401034	2.38993	2.38993
Gm37397	Gm37397-001	0.030736	1.81668	1.81668
Gm37438	Gm37438-001	0.0454837	0.195075	-5.12622
Gm37446	Gm37446-001	0.00674278	1.74054	1.74054
Gm37447	Gm37447-001	0.0122697	1.79166	1.79166
Gm37499	Gm37499-001	0.012074	0.395007	-2.5316
Gm37509	Gm37509-001	0.0168194	5.25225	5.25225
Gm37537	Gm37537-001	0.045492	0.189843	-5.26752
Gm37567	Gm37567-001	0.0360762	1.36154	1.36154
Gm37584	Gm37584-001	0.000517293	1.82359	1.82359
Gm37643	Gm37643-001	0.003358	0.193632	-5.16443
Gm37677	Gm37677-001	0.0180669	1.6386	1.6386
Gm37716	Gm37716-001	0.00414009	3.11965	3.11965
Gm37759	Gm37759-001	0.0151901	5.78604	5.78604
Gm37768	Gm37768-001	0.0191635	0.653517	-1.53018
Gm37800	Gm37800-002	0.0377101	3.25797	3.25797
Gm37829	Gm37829-001	0.045926	1.3715	1.3715
Gm37848	Gm37848-001	0.042994	0.378913	-2.63913
Gm37861	Gm37861-001	0.00699604	0.387467	-2.58087
Gm37868	Gm37868-001	0.0462484	0.25134	-3.97867
Gm37874	Gm37874-001	1.51841e-05	0.474507	-2.10745
Gm37894	Gm37894-001	0.0404049	1.8058	1.8058
Gm37924	Gm37924-001	0.00828509	1.80305	1.80305
Gm37935	Gm37935-001	0.00824039	11.9107	11.9107
Gm37949	Gm37949-001	0.0325566	2.06411	2.06411
Gm37953	Gm37953-001	0.0110836	1.52433	1.52433

Gm37954	Gm37954-001	0.0285285	2.2097	2.2097
Gm37964	Gm37964-001	0.0306807	0.299722	-3.33643
Gm37968	Gm37968-001	0.0460924	0.833867	-1.19923
Gm38080	Gm38080-001	0.00807795	0.110341	-9.0628
Gm38250	Gm38250-002	0.028632	1.90034	1.90034
Gm38253	Gm38253-001	0.000357555	1.63321	1.63321
Gm38293	Gm38293-001	0.0427471	2.99265	2.99265
Gm3830	Gm3830-001	0.0175247	0.703735	-1.42099
Gm38313	Gm38313-001	0.0417141	2.16839	2.16839
Gm38329	Gm38329-001	0.0153135	3.88846	3.88846
Gm38357	Gm38357-001	0.00249255	2.09726	2.09726
Gm38368	Gm38368-001	0.0441716	0.0975293	-10.2533
Gm38377	Gm38377-001	0.0461261	2.63684	2.63684
Gm38379	Gm38379-001	0.0452725	3.48401	3.48401
Gm38394	Gm38394-001	0.0142013	0.69869	-1.43125
Gm38394	Gm38394-201	0.0142013	0.69869	-1.43125
Gm3972	Gm3972-001	0.0187369	0.282118	-3.54462
Gm4076	Gm4076-201	0.0123621	0.845699	-1.18245
Gm42469	Gm42469-001	0.0352913	1.42696	1.42696
Gm42471	Gm42471-001	0.00348402	2.87845	2.87845
Gm42477	Gm42477-001	0.0244487	15.9779	15.9779
Gm42508	Gm42508-001	0.013318	0.177149	-5.64496
Gm42546	Gm42546-001	0.0258991	2.19421	2.19421
Gm42547	Gm42547-001	0.000471939	0.799398	-1.25094
Gm42553	Gm42553-001	0.00287964	3.41494	3.41494
Gm42633	Gm42633-001	0.0280559	0.35812	-2.79236
Gm42690	Gm42690-001	0.0172206	2.29307	2.29307
Gm42778	Gm42778-001	0.0225268	0.404319	-2.47329
Gm42791	Gm42791-001	0.00403158	4.86707	4.86707
Gm42840	Gm42840-001	0.0214333	0.278363	-3.59243
Gm42841	Gm42841-001	0.0309602	5.57568	5.57568
Gm4285	Gm4285-001	0.0107469	1.80473	1.80473
Gm42863	Gm42863-001	0.0347065	1.90943	1.90943
Gm42876	Gm42876-001	0.00622752	3.80935	3.80935
Gm42878	Gm42878-201	0.0334244	0.204473	-4.89062
Gm42905	Gm42905-001	0.0475555	0.449991	-2.22227
Gm42910	Gm42910-001	0.00668419	0.411235	-2.4317
Gm42923	Gm42923-001	0.0466529	2.1565	2.1565
Gm42940	Gm42940-001	0.0142373	0.237928	-4.20295
Gm42950	Gm42950-001	0.00341106	0.527809	-1.89463
Gm42979	Gm42979-001	0.0292677	1.40263	1.40263
Gm42984	Gm42984-001	0.0470117	3.00701	3.00701
Gm43042	Gm43042-001	0.00118113	2.91113	2.91113

Gm43080	Gm43080-001	0.000700715	1.54702	1.54702
Gm43081	Gm43081-001	0.0366697	2.41644	2.41644
Gm43127	Gm43127-001	0.0160821	6.85842	6.85842
Gm43128	Gm43128-001	0.00366976	0.0757438	-13.2024
Gm43172	Gm43172-001	0.00305255	2.12124	2.12124
Gm43267	Gm43267-001	0.00559909	2.67816	2.67816
Gm43270	Gm43270-001	0.0363567	1.80077	1.80077
Gm43283	Gm43283-001	0.0248084	0.188702	-5.29936
Gm43289	Gm43289-001	0.0465189	0.160512	-6.23006
Gm43314	Gm43314-001	1.62247e-05	4.0822	4.0822
Gm43332	Gm43332-001	0.0454696	1.99988	1.99988
Gm43335	Gm43335-001	0.0116013	0.328389	-3.04517
Gm43336	Gm43336-001	0.0380926	0.517147	-1.93369
Gm43338	Gm43338-001	0.00236231	2.09247	2.09247
Gm43379	Gm43379-001	0.0133071	1.70966	1.70966
Gm43417	Gm43417-001	0.0471088	0.636411	-1.57131
Gm43488	Gm43488-001	0.012617	2.5376	2.5376
Gm43513	Gm43513-001	0.0189265	1.76052	1.76052
Gm43546	Gm43546-001	0.0255308	1.67336	1.67336
Gm43571	Gm43571-001	0.0320827	0.548394	-1.82351
Gm43577	Gm43577-001	0.00582519	12.3141	12.3141
Gm43634	Gm43634-001	0.0493329	0.499417	-2.00233
Gm43667	Gm43667-001	0.0118489	2.157	2.157
Gm43689	Gm43689-001	0.0185208	4.2578	4.2578
Gm43742	Gm43742-001	0.0373297	0.477377	-2.09478
Gm43800	Gm43800-001	0.0376216	0.330914	-3.02193
Gm43807	Gm43807-001	0.0494456	0.464483	-2.15293
Gm43823	Gm43823-001	0.00463336	2.70078	2.70078
Gm43841	Gm43841-001	0.0370602	0.54514	-1.83439
Gm43857	Gm43857-001	0.0112788	1.58304	1.58304
Gm4419	Gm4419-201	0.0301032	3.37641	3.37641
Gm4673	Gm4673-002	0.0414768	1.80691	1.80691
Gm4723	Gm4723-001	0.0302582	14.7097	14.7097
Gm4889	Gm4889-201	0.00127296	0.506682	-1.97362
Gm4924	Gm4924-201	0.0403317	1.23948	1.23948
Gm4961	Gm4961-001	0.0130718	0.467116	-2.14079
Gm4980	Gm4980-001	0.00995876	0.729637	-1.37055
Gm5108	Gm5108-002	0.0356996	0.0238592	-41.9126
Gm5108	Gm5108-003	0.0356996	0.0238592	-41.9126
Gm5113	Gm5113-001	0.0342304	1.26319	1.26319
Gm5466	Gm5466-001	0.000504002	8.16395	8.16395
Gm5615	Gm5615-001	0.00151038	3.67791	3.67791
Gm5617	Gm5617-201	0.00261131	2.07916	2.07916

Gm5891	Gm5891-001	0.0117343	2.34698	2.34698
Gm5900	Gm5900-201	0.0384289	1.74398	1.74398
Gm5930	Gm5930-001	0.00968414	11.9421	11.9421
Gm6166	Gm6166-201	0.00748054	0.549576	-1.81958
Gm6297	Gm6297-201	0.0224516	2.41236	2.41236
Gm6377	Gm6377-001	0.0329589	5.09133	5.09133
Gm648	Gm648-001	0.00608932	2.83371	2.83371
Gm6483	Gm6483-003	0.00210764	1.34972	1.34972
Gm6483	Gm6483-005	0.0189296	1.76508	1.76508
Gm6611	Gm6611-001	0.0217591	2.52495	2.52495
Gm6712	Gm6712-001	0.0497613	2.90286	2.90286
Gm6890	Gm6890-001	0.036017	0.315385	-3.17073
Gm6934	Gm6934-001	0.0263033	15.8215	15.8215
Gm7008	Gm7008-001	0.0395878	2.21779	2.21779
Gm7102	Gm7102-001	0.00262598	1.28729	1.28729
Gm7160	Gm7160-002	0.00716916	1.47259	1.47259
Gm7393	Gm7393-001	0.0199292	0.173815	-5.75324
Gm7598	Gm7598-001	0.0114174	0.19358	-5.16582
Gm7609	Gm7609-003	0.00875248	1.44946	1.44946
Gm773	Gm773-001	0.0464669	1.31041	1.31041
Gm807	Gm807-201	0.00164733	0.78069	-1.28092
Gm8378	Gm8378-003	0.0472541	0.0589159	-16.9733
Gm8580	Gm8580-001	0.00207446	1.67422	1.67422
Gm8756	Gm8756-001	0.012796	2.58028	2.58028
Gm8765	Gm8765-001	0.00250896	0.548312	-1.82378
Gm8923	Gm8923-002	0.0414867	1.79643	1.79643
Gm8923	Gm8923-004	0.024807	11.0151	11.0151
Gm9095	Gm9095-001	0.0466043	0.291114	-3.43508
Gm9403	Gm9403-001	0.00627127	0.267805	-3.73406
Gm9522	Gm9522-001	0.0221461	2.18091	2.18091
Gm9710	Gm9710-001	0.00815491	2.97759	2.97759
Gm9732	Gm9732-001	0.0178994	1.31358	1.31358
Gm9747	Gm9747-201	0.0276005	0.247108	-4.04682
Gm9754	Gm9754-001	0.025365	0.403283	-2.47965
Gm9776	Gm9776-201	0.00779655	1.67473	1.67473
Gm9801	Gm9801-201	0.00110274	9.15247	9.15247
Gm9881	Gm9881-201	0.00701635	1.93739	1.93739
Gm9888	Gm9888-001	0.0436074	2.85209	2.85209
Gm9903	Gm9903-001	0.00797841	5.52312	5.52312
Gm9905	Gm9905-201	0.0128754	2.38413	2.38413
Gm9929	Gm9929-001	0.0244136	1.15411	1.15411
Gmcl1	Gmcl1-001	0.00674866	1.21858	1.21858
Gmeb2	Gmeb2-001	0.00594799	1.17084	1.17084

Gmeb2	Gmeb2-004	0.046986	29.8325	29.8325
Gmfb	Gmfb-201	0.00577121	0.584644	-1.71044
Gmfb	Gmfb-001	0.0125177	0.678735	-1.47333
Gmip	Gmip-001	0.0135392	0.673181	-1.48548
Gmppb	Gmppb-002	0.0305911	0.635712	-1.57304
Gna12	Gna12-001	0.000131784	0.739065	-1.35306
Gna13	Gna13-001	0.039324	0.868516	-1.15139
Gnai1	Gnai1-001	0.00328634	0.553381	-1.80707
Gnal	Gnal-202	0.0432572	0.678254	-1.47437
Gnas	Gnas-202	0.00776739	0.092867	-10.7681
Gnas	Gnas-010	0.0292615	0.142597	-7.01277
Gnas	Gnas-007	0.0211138	0.430856	-2.32096
Gnas	Gnas-006	0.0385719	0.67948	-1.47171
Gnas	Gnas-001	0.00209626	0.706612	-1.4152
Gnb1	Gnb1-001	0.00223798	0.733771	-1.36282
Gnb1	Gnb1-007	0.0176265	1.24508	1.24508
Gnb1	Gnb1-003	0.000647886	1.77043	1.77043
Gnb1l	Gnb1l-008	0.0136913	1.57546	1.57546
Gnb1l	Gnb1l-006	0.0350255	19.2031	19.2031
Gnb2	Gnb2-004	0.00178854	0.626637	-1.59582
Gnb2	Gnb2-014	0.0211182	0.650298	-1.53776
Gnb4	Gnb4-001	0.000197835	0.584919	-1.70964
Gnb5	Gnb5-201	0.008935	0.617973	-1.61819
Gne	Gne-014	0.0487619	0.0014437	-692.664
Gne	Gne-002	0.0207805	2.00786	2.00786
Gng11	Gng11-001	0.0178714	0.266588	-3.75111
Gng2	Gng2-001	0.00395485	0.731544	-1.36697
Gng2	Gng2-005	0.00820324	2.0344	2.0344
Gnl2	Gnl2-005	0.04336	0.474824	-2.10604
Gnl2	Gnl2-001	0.0239755	1.22865	1.22865
Gnpat	Gnpat-001	0.00763584	1.30551	1.30551
Gnpda1	Gnpda1-001	0.0031917	1.40431	1.40431
Gnpnat1	Gnpnat1-201	0.00193319	1.39547	1.39547
Gnptab	Gnptab-004	0.0476425	3.11043	3.11043
Golga1	Golga1-009	0.0339537	4.23607	4.23607
Golga3	Golga3-001	0.0355605	0.827928	-1.20783
Golga3	Golga3-002	0.00162713	1.38919	1.38919
Golga3	Golga3-005	0.0192585	2.86457	2.86457
Golga4	Golga4-201	0.000485099	0.844547	-1.18407
Golga7	Golga7-004	0.0030177	2.29341	2.29341
Golgb1	Golgb1-006	0.0307639	0.37891	-2.63915
Golgb1	Golgb1-004	0.0326144	3.10688	3.10688
Gorab	Gorab-001	0.0169549	1.33827	1.33827

Gorasp2	Gorasp2-001	0.00143465	1.38241	1.38241
Gorasp2	Gorasp2-003	0.000729395	2.15241	2.15241
Gosr1	Gosr1-001	0.0157336	1.23077	1.23077
Got1	Got1-001	0.0404701	1.49719	1.49719
Got1	Got1-002	0.0135733	2.57624	2.57624
Got1	Got1-003	0.00496999	3.85141	3.85141
Gpaa1	Gpaa1-007	0.043514	0.078188	-12.7897
Gpaa1	Gpaa1-002	0.0462241	0.446583	-2.23922
Gpaa1	Gpaa1-001	0.0116295	0.51276	-1.95023
Gpalpp1	Gpalpp1-201	0.0413304	1.32105	1.32105
Gpatch11	Gpatch11-201	0.000570889	1.45737	1.45737
Gpatch2	Gpatch2-005	0.0216379	2.35029	2.35029
Gpatch2l	Gpatch2l-201	0.0457817	1.11537	1.11537
Gpatch8	Gpatch8-001	0.0161467	0.813234	-1.22966
Gpatch8	Gpatch8-002	0.0156133	1.317	1.317
Gpc6	Gpc6-002	0.000878373	2.16192	2.16192
Gpd2	Gpd2-201	0.0325265	0.678289	-1.4743
Gpd2	Gpd2-202	0.0438196	0.717576	-1.39358
Gpi1	Gpi1-001	0.0432805	0.882998	-1.13251
Gpkow	Gpkow-001	0.036894	1.32093	1.32093
Gpm6b	Gpm6b-002	0.0110956	0.251607	-3.97445
Gpm6b	Gpm6b-003	0.00327183	0.310848	-3.21701
Gpm6b	Gpm6b-001	0.0193936	0.407449	-2.45429
Gpm6b	Gpm6b-005	0.0437685	0.846835	-1.18087
Gpn1	Gpn1-002	0.0297535	1.31955	1.31955
Gpn1	Gpn1-007	0.0127968	1.75727	1.75727
Gpr137	Gpr137-205	0.00268195	0.552372	-1.81038
Gpr137c	Gpr137c-001	0.00670545	0.534771	-1.86996
Gpr153	Gpr153-002	0.0217891	0.329529	-3.03464
Gpr19	Gpr19-010	0.00592133	0.28003	-3.57105
Gpr19	Gpr19-002	0.00634421	1.492	1.492
Gpr35	Gpr35-004	0.0139477	0.121827	-8.20838
Gpr35	Gpr35-003	0.0139508	0.12193	-8.2014
Gpr35	Gpr35-002	0.0139509	0.12193	-8.2014
Gpr4	Gpr4-001	0.0327898	2.18003	2.18003
Gpr45	Gpr45-003	0.000525037	2.25217	2.25217
Gpr68	Gpr68-002	0.000128718	1.48644	1.48644
Gpr68	Gpr68-201	0.004875	2.1693	2.1693
Gpr75	Gpr75-001	0.0395184	0.752118	-1.32958
Gprasp1	Gprasp1-002	0.0466874	1.46674	1.46674
Gprasp1	Gprasp1-004	0.00167552	1.88497	1.88497
Gprc5d	Gprc5d-001	0.0167087	0.222613	-4.4921
Gprc5d	Gprc5d-002	0.0167087	0.222613	-4.4921

Gps1	Gps1-007	0.042858	0.841513	-1.18834
Gpsm1	Gpsm1-004	0.0422524	0.492532	-2.03033
Gpsm2	Gpsm2-001	0.0019694	1.39962	1.39962
Gpt2	Gpt2-002	0.0294679	1.31635	1.31635
Gpt2	Gpt2-001	0.00180737	1.52837	1.52837
Gpx4	Gpx4-001	0.0134199	0.747033	-1.33863
Gpx4	Gpx4-007	0.0128587	0.805517	-1.24144
Gpx4	Gpx4-006	0.0284042	1.96995	1.96995
Gpx7	Gpx7-001	0.000894397	2.70429	2.70429
Gramd1a	Gramd1a-201	0.0256051	0.493127	-2.02788
Gramd1a	Gramd1a-004	0.0248343	0.660678	-1.5136
Gramd1a	Gramd1a-003	0.00364723	0.670416	-1.49161
Gramd1b	Gramd1b-010	0.0260397	1.77533	1.77533
Gramd2	Gramd2-001	0.0137391	1.47298	1.47298
Gramd2	Gramd2-003	0.0176344	1.80788	1.80788
Gramd3	Gramd3-201	0.00218744	1.52023	1.52023
Gramd4	Gramd4-002	0.00531676	0.587148	-1.70315
Grap2	Grap2-201	0.0144242	0.366287	-2.7301
Grb14	Grb14-001	0.00593864	1.51236	1.51236
Grb2	Grb2-005	0.00717534	3.62514	3.62514
Grcc10	Grcc10-004	0.0127833	0.000377288	-2650.49
Grcc10	Grcc10-001	7.416e-05	0.441857	-2.26317
Greb1	Greb1-011	0.0473811	1.57447	1.57447
Greb1	Greb1-201	0.00062507	1.76664	1.76664
Greb1	Greb1-001	0.000625295	1.76665	1.76665
Grhl1	Grhl1-202	0.0177646	1.78269	1.78269
Grhl1	Grhl1-201	0.0257755	8.34567	8.34567
Grhpr	Grhpr-001	0.0208416	1.40942	1.40942
Gria2	Gria2-002	0.000829336	1.53792	1.53792
Gria2	Gria2-006	0.0370144	2.40794	2.40794
Grik4	Grik4-201	0.0447421	0.687338	-1.45489
Grik5	Grik5-003	0.0170181	1.92782	1.92782
Grip1	Grip1-014	0.0237295	0.46221	-2.16352
Grip1os1	Grip1os1-002	0.0278167	0.00197186	-507.136
Gripap1	Gripap1-007	0.0292467	1.54153	1.54153
Grm1	Grm1-002	0.0355568	0.674326	-1.48296
Grn	Grn-001	0.000130792	0.668992	-1.49479
Grrp1	Grrp1-001	0.000365025	0.451493	-2.21488
Grsf1	Grsf1-002	0.0414084	1.2677	1.2677
Gsg1l	Gsg1l-001	0.00409232	1.39158	1.39158
Gskip	Gskip-201	0.00556108	1.32191	1.32191
Gsn	Gsn-005	0.00528891	0.635712	-1.57304
Gspt1	Gspt1-004	0.0162398	1.76357	1.76357

Gspt2	Gspt2-001	0.00488475	1.37453	1.37453
Gsr	Gsr-003	0.0465153	1.32071	1.32071
Gss	Gss-003	0.007861	1.53161	1.53161
Gss	Gss-001	0.0229198	2.46806	2.46806
Gstm5	Gstm5-006	0.00859478	3.7872	3.7872
Gstm6	Gstm6-006	0.0142359	2.9791	2.9791
Gstm6	Gstm6-002	0.0289418	7.91428	7.91428
Gstm7	Gstm7-001	0.00163693	4.2822	4.2822
Gsto1	Gsto1-001	0.00738268	1.61289	1.61289
Gstp1	Gstp1-001	0.000726408	1.29291	1.29291
Gstt2	Gstt2-201	0.00424137	2.31977	2.31977
Gstz1	Gstz1-201	0.021866	0.838147	-1.19311
Gtf2a1	Gtf2a1-001	0.00974244	1.26034	1.26034
Gtf2a1	Gtf2a1-002	0.0114643	5.40908	5.40908
Gtf2f2	Gtf2f2-201	0.0119495	1.32538	1.32538
Gtf2h2	Gtf2h2-001	0.00741501	1.25025	1.25025
Gtf2h2	Gtf2h2-004	0.0348854	1.30177	1.30177
Gtf2h2	Gtf2h2-005	0.0101657	1.7286	1.7286
Gtf2h2	Gtf2h2-002	0.00235339	2.46583	2.46583
Gtf2h3	Gtf2h3-003	0.00102886	2.03033	2.03033
Gtf2h5	Gtf2h5-002	0.0233606	1.2978	1.2978
Gtf2i	Gtf2i-017	0.0497135	0.836308	-1.19573
Gtf2ird1	Gtf2ird1-027	0.00891564	3.29783	3.29783
Gtf3a	Gtf3a-001	0.00325014	1.47505	1.47505
Gtf3c3	Gtf3c3-001	0.0130584	1.13511	1.13511
Gtpbp2	Gtpbp2-012	0.0145201	1.57421	1.57421
Gtpbp2	Gtpbp2-016	0.0331263	2.03931	2.03931
Gtpbp2	Gtpbp2-009	0.0477252	22.216	22.216
Gtpbp4	Gtpbp4-201	0.0458975	1.27835	1.27835
Gtpbp8	Gtpbp8-004	0.00729047	2.91975	2.91975
Gucy1a2	Gucy1a2-201	0.0433811	0.67605	-1.47918
Gucy1a3	Gucy1a3-001	0.000634291	1.47399	1.47399
Gucy1b3	Gucy1b3-004	0.000306313	1.30794	1.30794
Gucy2g	Gucy2g-001	0.0308202	1.61545	1.61545
Guf1	Guf1-007	0.0107767	0.763967	-1.30896
Gusb	Gusb-001	0.000517366	0.289369	-3.4558
Gusb	Gusb-006	0.00127045	0.386878	-2.58479
Gxylt1	Gxylt1-002	0.00993607	3.18752	3.18752
H13	H13-005	0.00861336	1.46545	1.46545
H2-DMa	H2-DMa-001	0.0131512	2.01959	2.01959
H2-K1	H2-K1-007	0.0104038	1.92833	1.92833
H2-K1	H2-K1-002	0.0472592	1.94362	1.94362
H2-K1	H2-K1-010	0.0437913	2.38894	2.38894

H2-M3	H2-M3-001	0.00726432	1.37793	1.37793
H2-T10	H2-T10-003	0.0336987	0.320362	-3.12147
H2-T22	H2-T22-001	0.0140105	0.338582	-2.95349
H2-T23	H2-T23-001	0.0454315	1.15186	1.15186
H2afj	H2afj-001	0.0291485	0.70291	-1.42266
H2afx	H2afx-001	0.0309301	1.53514	1.53514
H2afy	H2afy-001	0.00760695	0.76154	-1.31313
H2afy	H2afy-007	0.0489885	1.64263	1.64263
H3f3b	H3f3b-001	0.00114171	3.01312	3.01312
H60b	H60b-201	0.0115386	0.576194	-1.73553
H60b	H60b-001	0.0115385	0.576195	-1.73552
Hadh	Hadh-001	0.00233382	1.20658	1.20658
Hadha	Hadha-007	0.0354867	0.274611	-3.64151
Hagh	Hagh-007	0.0409774	1826.23	1826.23
Haghl	Haghl-009	0.024066	1.67386	1.67386
Harbi1	Harbi1-005	0.0418107	1394.55	1394.55
Hars2	Hars2-005	0.0234992	1.74232	1.74232
Has3	Has3-001	0.0128842	0.675272	-1.48088
Hat1	Hat1-001	0.00757329	2.18916	2.18916
Haus8	Haus8-004	0.000730352	2.04382	2.04382
Hax1	Hax1-001	0.000209545	1.57912	1.57912
Hbegf	Hbegf-201	0.0416116	0.685528	-1.45873
Hbs1l	Hbs1l-201	0.00465742	0.845443	-1.18281
Hcfc1	Hcfc1-007	0.0276506	2.26305	2.26305
Hcfc2	Hcfc2-001	0.00624211	1.24242	1.24242
Hcfc2	Hcfc2-004	0.00984103	1.26813	1.26813
Hcn2	Hcn2-201	0.01455	0.606839	-1.64788
Hcn2	Hcn2-001	0.00201645	0.64386	-1.55313
Hcn3	Hcn3-001	0.00272934	0.689983	-1.44931
Hcrtr1	Hcrtr1-001	0.016851	5.53522	5.53522
Hcrtr1	Hcrtr1-002	0.016851	5.53522	5.53522
Hcrtr1	Hcrtr1-003	0.016851	5.53523	5.53523
Hdac1	Hdac1-003	0.0245294	0.546437	-1.83004
Hdac10	Hdac10-004	0.00793461	0.335779	-2.97815
Hdac2	Hdac2-001	0.000496678	0.736604	-1.35758
Hdac3	Hdac3-001	0.000666946	0.724047	-1.38113
Hdac4	Hdac4-003	0.0416699	0.219541	-4.55497
Hdac4	Hdac4-201	0.00189414	4.5157	4.5157
Hdac6	Hdac6-201	0.00930838	0.644593	-1.55137
Hdac8	Hdac8-001	0.00760412	1.80364	1.80364
Hdac9	Hdac9-201	0.010317	1.68862	1.68862
Hddc3	Hddc3-008	0.0128137	2.05168	2.05168
Hddc3	Hddc3-003	0.0220281	2.08548	2.08548

Hdhd2	Hdhd2-001	0.0366983	1.28081	1.28081
Hdhd2	Hdhd2-002	0.012821	4.40262	4.40262
Hdlbp	Hdlbp-001	1.7381e-06	0.671199	-1.48987
Hdlbp	Hdlbp-004	0.00367947	1.40247	1.40247
Hdlbp	Hdlbp-003	0.000245089	1.88012	1.88012
Heatr5b	Heatr5b-201	0.00440714	1.15275	1.15275
Heatr6	Heatr6-002	0.0206746	1.69923	1.69923
Hectd3	Hectd3-001	0.0275956	0.874574	-1.14341
Hells	Hells-004	0.0165411	1.5646	1.5646
Helz	Helz-002	0.0111478	0.52397	-1.9085
Helz	Helz-201	0.001566	1.63122	1.63122
Hemk1	Hemk1-001	0.0102004	1.54643	1.54643
Herc1	Herc1-002	0.00195106	2.21355	2.21355
Herc3	Herc3-001	0.00207101	1.40117	1.40117
Herpud1	Herpud1-002	0.00203889	1.77061	1.77061
Herpud1	Herpud1-001	0.000418971	1.83375	1.83375
Herpud1	Herpud1-003	0.00455012	1.96768	1.96768
Hes6	Hes6-001	0.0397685	0.732687	-1.36484
Hexa	Hexa-201	0.00592273	0.742498	-1.34681
Hexdc	Hexdc-003	0.013388	1.49498	1.49498
Hexim2	Hexim2-004	0.0229614	4.60474	4.60474
Hey1	Hey1-001	0.0119238	1.55953	1.55953
Hfm1	Hfm1-002	0.0478365	0.197564	-5.06166
Hgs	Hgs-001	0.0323616	1.42475	1.42475
Hibch	Hibch-001	0.00531942	1.35375	1.35375
Higd2a	Higd2a-001	0.0137191	1.20552	1.20552
Hilpda	Hilpda-001	0.0283418	1.42275	1.42275
Hinfp	Hinfp-201	0.0127237	1.14836	1.14836
Hint1	Hint1-002	0.0373774	0.421449	-2.37277
Hint3	Hint3-001	0.0278398	2.61449	2.61449
Hint3	Hint3-003	0.0259289	2.63359	2.63359
Hip1	Hip1-009	0.00322514	3.19327	3.19327
Hip1	Hip1-003	0.0173738	4.38785	4.38785
Hip1r	Hip1r-001	0.00191628	0.770164	-1.29842
Hipk2	Hipk2-005	0.000837241	2.74581	2.74581
Hipk3	Hipk3-001	0.0481362	0.672246	-1.48755
Hira	Hira-005	0.0308008	1.70144	1.70144
Hira	Hira-004	0.0302662	1.84071	1.84071
Hist1h1b	Hist1h1b-001	0.0173845	2.91306	2.91306
Hist1h1c	Hist1h1c-001	0.0117938	2.33557	2.33557
Hist1h2ac	Hist1h2ac-001	0.0322865	1.11787e+08	1.11787e+08
Hist1h2ae	Hist1h2ae-001	0.0348806	4.25677	4.25677
Hist1h2ba	Hist1h2ba-001	0.0178364	8.55629	8.55629

Hist1h2bb	Hist1h2bb-001	0.00765669	5.13356	5.13356
Hist1h2bc	Hist1h2bc-001	0.00639844	3.66849	3.66849
Hist1h4i	Hist1h4i-001	0.0152185	2.78257	2.78257
Hist1h4j	Hist1h4j-001	0.00243705	5.28821	5.28821
Hist1h4k	Hist1h4k-001	0.029558	3.71705	3.71705
Hist2h2bb	Hist2h2bb-001	0.019462	4.56813	4.56813
Hist2h3c2	Hist2h3c2-001	0.000493455	11.8837	11.8837
Hist2h4	Hist2h4-001	0.00202255	4.45376	4.45376
Hist3h2a	Hist3h2a-001	0.0332671	2.09789	2.09789
Hist3h2ba	Hist3h2ba-001	0.000109377	2.34079	2.34079
Hk1	Hk1-003	0.0340092	0.00495796	-201.696
Hk1	Hk1-011	0.0340089	0.00495798	-201.695
Hk1os	Hk1os-002	0.0012283	2.04967	2.04967
Hk2	Hk2-002	0.00333493	1.97902	1.97902
Hk3	Hk3-002	0.00178459	0.179944	-5.55728
Hkdc1	Hkdc1-001	0.0403615	2.10597	2.10597
Hmbox1	Hmbox1-006	7.67221e-06	0.552592	-1.80965
Hmbox1	Hmbox1-003	0.0427711	0.63328	-1.57908
Hmbox1	Hmbox1-201	0.0192422	0.785076	-1.27376
Hmces	Hmces-001	0.00229224	1.47897	1.47897
Hmg20b	Hmg20b-001	0.012826	0.443071	-2.25697
Hmg20b	Hmg20b-004	0.00122878	0.707823	-1.41278
Hmg20b	Hmg20b-008	0.0320082	3.51334	3.51334
Hmga1-rs1	Hmga1-rs1-001	0.018849	0.560325	-1.78468
Hmgb3	Hmgb3-006	0.0192967	1.70503	1.70503
Hmgcl	Hmgcl-005	0.0353202	1.86682	1.86682
Hmgcll1	Hmgcll1-001	0.000109031	0.57905	-1.72697
Hmgn2	Hmgn2-002	0.019894	0.544101	-1.83789
Hmgn5	Hmgn5-001	0.0202884	1.20713	1.20713
Hmgxb3	Hmgxb3-201	0.00364265	1.32521	1.32521
Hmha1	Hmha1-004	0.000144392	0.463479	-2.1576
Hmox1	Hmox1-001	0.000443031	2.80665	2.80665
Hn1	Hn1-001	0.0033798	0.616104	-1.6231
Hn1	Hn1-003	0.0475974	1.9403	1.9403
Hnrnpa1	Hnrnpa1-201	0.0193735	0.646889	-1.54586
Hnrnpa2b1	Hnrnpa2b1-201	0.00221724	1.2915	1.2915
Hnrnpa3	Hnrnpa3-001	0.00911136	0.903112	-1.10728
Hnrnpa3	Hnrnpa3-002	0.0171259	1.08933	1.08933
Hnrnpa3	Hnrnpa3-202	0.0171758	1.18727	1.18727
Hnrnpc	Hnrnpc-201	0.00162802	1.20616	1.20616
Hnrnpd	Hnrnpd-001	0.0030968	0.38672	-2.58585
Hnrnpdl	Hnrnpdl-007	0.0219518	0.0678247	-14.7439
Hnrnpdl	Hnrnpdl-004	0.000767909	0.70428	-1.41989

Hnrnph1	Hnrnph1-003	0.00337065	0.309328	-3.23281
Hnrnph1	Hnrnph1-014	0.03732	0.641634	-1.55852
Hnrnph1	Hnrnph1-007	0.00191494	0.765423	-1.30647
Hnrnph1	Hnrnph1-009	0.0447895	0.804707	-1.24269
Hnrnph3	Hnrnph3-002	0.0266502	1.40046	1.40046
Hnrnpk	Hnrnpk-003	0.0289635	0.702482	-1.42352
Hnrnpk	Hnrnpk-021	0.00045055	1.26327	1.26327
Hnrnpk	Hnrnpk-014	0.00460747	1.26489	1.26489
Hnrnpk	Hnrnpk-020	0.0330212	1.64527	1.64527
Hnrnpl	Hnrnpl-009	0.0011184	2.02985	2.02985
Hnrnpl	Hnrnpl-010	6.03208e-06	2.67679	2.67679
Hnrnpm	Hnrnpm-004	0.0371394	0.846475	-1.18137
Hnrnpr	Hnrnpr-001	0.0277784	1.14413	1.14413
Hnrnpr	Hnrnpr-005	0.0350637	2.22095	2.22095
Hnrnpr	Hnrnpr-006	0.0162028	3.6065	3.6065
Hnrnpul1	Hnrnpul1-001	0.0411592	1.28688	1.28688
Homer1	Homer1-008	0.0298014	0.558308	-1.79113
Homez	Homez-004	0.0412812	0.50334	-1.98673
Hook1	Hook1-001	0.0387654	0.900148	-1.11093
Hoxc4	Hoxc4-001	0.031476	0.341925	-2.92462
Hoxc6	Hoxc6-003	0.0445215	2.09995	2.09995
Hoxc6	Hoxc6-002	0.00657277	2.5595	2.5595
Hp1bp3	Hp1bp3-202	0.0395684	1.48648	1.48648
Hp1bp3	Hp1bp3-007	0.0363498	61.2301	61.2301
Hpca	Hpca-001	0.0481737	0.700715	-1.42711
Hpn	Hpn-003	0.0296768	0.0256358	-39.008
Hpn	Hpn-005	0.0491223	0.248934	-4.01712
Hpn	Hpn-009	0.0252116	0.257778	-3.8793
Hpn	Hpn-008	0.0148659	0.404147	-2.47435
Hprt	Hprt-001	0.0225906	1.12649	1.12649
Hps5	Hps5-013	0.0191929	6.46183	6.46183
Hr	Hr-001	0.0479849	0.818557	-1.22166
Hrsp12	Hrsp12-001	0.00573889	1.66993	1.66993
Hs2st1	Hs2st1-002	0.0416726	3.2069	3.2069
Hs3st2	Hs3st2-001	0.0111449	0.404971	-2.46931
Hs3st3b1	Hs3st3b1-001	0.0389639	0.771737	-1.29578
Hs6st1	Hs6st1-002	8.66024e-06	0.623429	-1.60403
Hsbp1	Hsbp1-201	0.0036928	0.908941	-1.10018
Hsd11b2	Hsd11b2-201	0.00662151	0.452906	-2.20796
Hsd17b11	Hsd17b11-001	0.0184876	0.776513	-1.28781
Hsd17b4	Hsd17b4-001	0.00728643	1.18181	1.18181
Hsd17b7	Hsd17b7-002	0.0115528	1.98812	1.98812
Hsf2	Hsf2-201	2.04163e-05	1.25384	1.25384

Hsf4	Hsf4-001	0.0274778	0.0112432	-88.9429
Hsp90b1	Hsp90b1-005	2.74453e-08	7.7396e+07	7.7396e+07
Hspa12a	Hspa12a-201	0.0173846	0.834684	-1.19806
Hspa4	Hspa4-003	0.0458145	1.20952	1.20952
Hspa4l	Hspa4l-001	5.01362e-05	0.67788	-1.47519
Hspa5	Hspa5-001	0.00279439	1.39567	1.39567
Hspa9	Hspa9-001	0.00078479	1.2919	1.2919
Hspb6	Hspb6-002	0.021042	1.55436	1.55436
Hspb6	Hspb6-003	0.0117865	1.8413	1.8413
Hspb8	Hspb8-001	0.00260705	1.25269	1.25269
Hspg2	Hspg2-201	0.00273282	0.430963	-2.32038
Hspg2	Hspg2-001	0.000578463	0.453927	-2.203
Hsph1	Hsph1-009	0.00555864	0.273489	-3.65645
Hsph1	Hsph1-010	0.0148476	0.587816	-1.70121
Hsph1	Hsph1-005	0.010053	4.39939	4.39939
Htatsf1	Htatsf1-001	0.0153385	1.34479	1.34479
Htra2	Htra2-007	0.000453771	0.190071	-5.2612
Htra2	Htra2-001	4.83092e-05	0.578193	-1.72952
Hus1	Hus1-006	0.00874111	1.69187	1.69187
Hus1	Hus1-005	0.0449729	11.5592	11.5592
Hvcn1	Hvcn1-002	0.00643982	0.275418	-3.63084
Hyou1	Hyou1-001	0.0281637	1.32592	1.32592
Iars	Iars-201	0.0214548	1.21888	1.21888
Iars	Iars-001	0.00892801	1.2304	1.2304
Ica1	Ica1-009	0.0498346	0.0885026	-11.2991
Ica1	Ica1-006	0.00402075	1.466	1.466
Icam1	Icam1-201	0.0346484	0.353446	-2.82929
Icam2	Icam2-001	0.000461168	0.655049	-1.5266
Ice1	Ice1-201	0.0196514	1.20128	1.20128
Icmt	Icmt-001	0.00207385	0.762642	-1.31123
Id1	Id1-001	0.0160507	1.74868	1.74868
Id1	Id1-002	0.0245613	3.39492	3.39492
Ide	Ide-003	0.000539714	2.92548	2.92548
Ide	Ide-002	0.00711357	5.65798	5.65798
Idh1	Idh1-201	0.0330265	1.50434	1.50434
Idh2	Idh2-003	0.00426516	0.121877	-8.20499
Idh3b	Idh3b-001	0.00895656	1.10277	1.10277
Ids	Ids-001	0.000182159	1.49305	1.49305
Ier2	Ier2-201	0.0474933	1.14794	1.14794
Ier5l	Ier5l-001	0.0368868	0.60289	-1.65868
Iffo1	Iffo1-004	0.00521774	0.249231	-4.01234
Ifi27	Ifi27-001	0.0477498	0.676323	-1.47858
Ifih1	Ifih1-002	0.0147829	6.33774	6.33774

Ifit3b	Ifit3b-001	0.0389536	2.39344	2.39344
Ifitm3	Ifitm3-001	0.0121738	1.30902	1.30902
Ifngr1	Ifngr1-005	0.0230137	2.74457	2.74457
Ifngr2	Ifngr2-001	0.000471558	0.707619	-1.41319
Ifrd1	Ifrd1-007	0.0223903	1.17522	1.17522
Ift122	Ift122-003	0.0116069	1.52011	1.52011
Ift122	Ift122-001	0.00877127	1.60801	1.60801
Ift20	Ift20-002	0.0183367	1.89217	1.89217
Ift22	Ift22-004	0.0379462	1.3751	1.3751
Ift43	Ift43-201	0.00803743	0.756046	-1.32267
Ift46	Ift46-011	0.0494819	1.33014	1.33014
Ift52	Ift52-001	0.0318157	1.10197	1.10197
Ift52	Ift52-002	0.0284587	2.61193	2.61193
Ift57	Ift57-003	0.00483134	1.35685	1.35685
Ift80	Ift80-201	0.030845	0.603063	-1.6582
Ift80	Ift80-002	0.0118819	1.69577	1.69577
Ift81	Ift81-001	0.00112364	1.27485	1.27485
Ift81	Ift81-008	0.0333567	2.50326	2.50326
Igf2bp3	Igf2bp3-001	0.0167553	0.538913	-1.85559
Igf2r	Igf2r-001	0.00086362	0.377493	-2.64906
Igfbp4	Igfbp4-001	0.00475436	0.780473	-1.28127
Igfbp4	Igfbp4-004	0.00245884	0.807071	-1.23905
Igfbp5	Igfbp5-002	0.0276416	0.426342	-2.34554
Igfbp5	Igfbp5-001	0.0495186	0.721002	-1.38696
Igfbp7	Igfbp7-001	0.00824915	0.499687	-2.00125
Ighmbp2	Ighmbp2-001	0.0321073	1.30639	1.30639
Igkv4-78	Igkv4-78-001	0.00669575	0.140578	-7.1135
Igsf23	Igsf23-201	0.000976719	1.35579	1.35579
Igsf8	Igsf8-002	0.00317535	0.676743	-1.47767
Ik	Ik-201	0.000441918	1.23146	1.23146
Ikbkap	Ikbkap-006	0.00722959	0.560989	-1.78256
Ikbkb	Ikbkb-002	0.00187346	0.304857	-3.28022
Ikbkb	Ikbkb-001	0.0295862	0.468235	-2.13568
Ikbkb	Ikbkb-010	0.0109839	0.692322	-1.44442
Ikbkb	Ikbkb-011	0.0181706	0.70252	-1.42345
Ikbkg	Ikbkg-006	0.0374564	0.738195	-1.35466
Ikbkg	Ikbkg-005	0.0374802	1.78323	1.78323
Ikzf2	Ikzf2-001	0.00727622	0.477816	-2.09285
Il11ra1	Il11ra1-003	0.0128996	0.339519	-2.94534
Il17rc	Il17rc-003	0.0337251	0.702749	-1.42298
Il17rd	Il17rd-201	0.00432213	0.729767	-1.3703
Il18bp	Il18bp-201	0.00705914	1.33076	1.33076
Il1rap	Il1rap-001	0.0294271	10.9058	10.9058

Il27ra	Il27ra-201	0.00104286	0.701124	-1.42628
Il4i1	Il4i1-201	3.32881e-06	1.5142	1.5142
Il4ra	Il4ra-002	0.0083128	0.811875	-1.23172
Il6	Il6-001	0.049203	0.333271	-3.00057
Il6	Il6-004	0.000231881	0.421775	-2.37093
Il6st	Il6st-008	0.0157302	6.26411	6.26411
Ilf3	Ilf3-202	0.0197228	0.916898	-1.09063
Ilk	Ilk-001	0.0376815	0.745999	-1.34048
Ilkap	Ilkap-013	0.0212232	0.0349304	-28.6283
Ilkap	Ilkap-011	0.00104759	0.537421	-1.86074
Immp1l	Immp1l-001	0.043517	1.27469	1.27469
Immt	Immt-010	0.00818694	0.609836	-1.63979
Impa1	Impa1-004	0.0295693	1.52162	1.52162
Impa2	Impa2-201	0.0492195	0.859135	-1.16396
Impact	Impact-201	0.00243058	1.29414	1.29414
Impdh1	Impdh1-001	0.0087307	1.1415	1.1415
Impg2	Impg2-001	0.0281199	0.253643	-3.94255
Inafm2	Inafm2-001	0.0030494	0.676395	-1.47843
Inf2	Inf2-201	0.00432226	0.794391	-1.25883
Ing3	Ing3-003	0.0239654	1.95276	1.95276
Inhbc	Inhbc-201	0.00169825	4.68627	4.68627
Ino80	Ino80-001	0.00370968	1.18543	1.18543
Ino80c	Ino80c-001	0.016831	0.567077	-1.76343
Ino80d	Ino80d-001	0.0428531	4.51527	4.51527
Ino80d	Ino80d-002	0.025723	919.067	919.067
Inpp5b	Inpp5b-002	0.00289197	2.08614	2.08614
Inpp5e	Inpp5e-002	0.0363646	2.55833	2.55833
Inpp5j	Inpp5j-006	0.0446547	2.35715	2.35715
Inpp5j	Inpp5j-004	0.00522149	17.1624	17.1624
Inppl1	Inppl1-003	0.0235821	0.725015	-1.37928
Insr	Insr-001	0.00370084	0.662264	-1.50997
Ints10	Ints10-004	0.0203983	1.34037	1.34037
Ints12	Ints12-001	0.00163798	1.49028	1.49028
Ints2	Ints2-008	0.0105324	0.71177	-1.40495
Ints4	Ints4-001	0.0295307	1.35493	1.35493
Ints4	Ints4-002	0.0240287	2.3966	2.3966
Ints7	Ints7-002	0.0437925	1.37784	1.37784
Ints7	Ints7-003	0.000325927	1.63982	1.63982
Ints7	Ints7-006	1.09983e-05	2.37048	2.37048
Ip6k2	Ip6k2-004	0.00560298	2.40395	2.40395
Ipmk	Ipmk-001	0.00621586	0.754086	-1.32611
Ipmk	Ipmk-003	0.0300919	1.96522	1.96522
Ipo11	Ipo11-005	0.0435887	2.4545	2.4545

Ipo13	Ipo13-004	0.0484968	0.337681	-2.96137
Ipo4	Ipo4-002	0.0342676	0.504828	-1.98087
Ipo4	Ipo4-003	0.0162767	0.625566	-1.59855
Ipo8	Ipo8-002	0.0351556	1.35364	1.35364
Ipp	Ipp-001	0.0258722	1.87078	1.87078
Iqce	Iqce-002	0.0104907	1.58916	1.58916
Iqce	Iqce-010	0.0244156	1.72022	1.72022
Iqgap1	Iqgap1-001	0.000195421	0.56964	-1.7555
Irak1	Irak1-001	0.00927612	0.761227	-1.31367
Irak1	Irak1-018	0.00923829	1.79855	1.79855
Irak1	Irak1-014	0.0468692	3.52933	3.52933
Irak1bp1	Irak1bp1-001	0.00813164	1.1873	1.1873
Irak4	Irak4-001	0.0355625	1.45794	1.45794
Irf2bpl	Irf2bpl-201	0.042752	0.863937	-1.15749
Irf3	Irf3-201	0.0451891	0.593861	-1.68389
Irf3	Irf3-202	0.00314761	0.624715	-1.60073
Irgm2	Irgm2-001	0.00394533	2.16169	2.16169
Irgq	Irgq-001	0.0425006	0.861422	-1.16087
Isg15	Isg15-201	0.000545774	0.661293	-1.51219
Isl2	Isl2-002	0.0395562	2.09631	2.09631
Islr2	Islr2-203	0.0314449	0.721143	-1.38669
Isyna1	Isyna1-201	1.18938e-05	0.518314	-1.92933
Itch	Itch-002	0.00474905	0.436018	-2.29349
Itfg2	Itfg2-001	0.040592	1.35448	1.35448
Itga1	Itga1-201	0.00666268	0.679302	-1.4721
Itga11	Itga11-001	0.0182899	0.345908	-2.89094
Itga2	Itga2-201	0.00821909	0.383886	-2.60494
Itgae	Itgae-001	0.0420638	1.74875	1.74875
Itgal	Itgal-003	0.00168446	0.155016	-6.45096
Itgav	Itgav-001	0.000593972	0.827876	-1.20791
Itgb1	Itgb1-001	0.000355467	0.749725	-1.33382
Itgb1bp1	Itgb1bp1-006	0.0278556	1.30855	1.30855
Itgb1bp2	Itgb1bp2-001	0.0386059	0.50269	-1.9893
Itgb3	Itgb3-001	0.0239467	0.554889	-1.80216
Itgb5	Itgb5-005	0.0274861	5016.11	5016.11
Itgbl1	Itgbl1-003	0.0225087	1.29319	1.29319
Itgbl1	Itgbl1-001	0.0225056	1.2932	1.2932
Itm2b	Itm2b-201	0.00667109	0.786062	-1.27216
Itpk1	Itpk1-001	0.0301931	0.840217	-1.19017
Itpka	Itpka-001	0.0179466	0.744825	-1.3426
Itpkb	Itpkb-001	0.0039969	1.65673	1.65673
Itpr2	Itpr2-001	0.0308055	0.65026	-1.53785
Itpr2	Itpr2-004	0.0336224	348.351	348.351

Itsn1	Itsn1-005	0.00442934	2.07804	2.07804
Itsn2	Itsn2-201	0.000102269	1.29653	1.29653
Ivd	Ivd-004	0.0499749	0.437344	-2.28653
Ivd	Ivd-007	0.00974013	0.801282	-1.248
Jade1	Jade1-005	0.00383563	1.68349	1.68349
Jade1	Jade1-004	0.00503962	2.06487	2.06487
Jakmip2	Jakmip2-201	0.00452482	1.21093	1.21093
Jarid2	Jarid2-001	0.0154355	0.716029	-1.39659
Jkamp	Jkamp-005	0.0357968	2.05203	2.05203
Jmjd6	Jmjd6-007	0.0289835	0.50838	-1.96703
Jmjd7	Jmjd7-001	0.00376844	3.0104	3.0104
Josd2	Josd2-005	0.0357396	1.26377	1.26377
Jph3	Jph3-001	0.0154102	1.55926	1.55926
Jph4	Jph4-004	0.0349395	0.597617	-1.67331
Jph4	Jph4-002	0.0280754	0.672001	-1.48809
Jph4	Jph4-001	0.0234198	0.780581	-1.2811
Jup	Jup-001	0.000903371	0.440718	-2.26903
Kalrn	Kalrn-016	0.00213095	0.103094	-9.69988
Kalrn	Kalrn-002	0.00031883	0.400087	-2.49946
Kalrn	Kalrn-015	0.0140391	0.611393	-1.63561
Kank2	Kank2-201	0.000969605	0.794145	-1.25922
Kansl2	Kansl2-202	0.0344429	1.47482	1.47482
Kansl3	Kansl3-001	0.000172296	1.24981	1.24981
Kansl3	Kansl3-005	0.0280944	2.1527	2.1527
Kantr	Kantr-001	0.0261564	1.60196	1.60196
Kat2a	Kat2a-004	0.010414	0.680258	-1.47003
Kat5	Kat5-201	0.000440637	1.35074	1.35074
Kat7	Kat7-009	0.0492459	2.2428e-09	-4.45871e+08
Kat8	Kat8-002	0.00337878	1.59773	1.59773
Katna1	Katna1-001	0.00358776	1.30833	1.30833
Katna1	Katna1-201	0.00110058	1.33818	1.33818
Katnal1	Katnal1-006	0.00220619	4.29327	4.29327
Katnal2	Katnal2-005	0.0158321	6.58661	6.58661
Kbtbd11	Kbtbd11-002	0.0249541	1.74279	1.74279
Kbtbd3	Kbtbd3-201	0.00316931	1.67604	1.67604
Kcmf1	Kcmf1-001	0.000484518	0.660736	-1.51346
Kcmf1	Kcmf1-002	0.00166526	1.33958	1.33958
Kcmf1	Kcmf1-006	0.00470525	1.62074	1.62074
Kcnab1	Kcnab1-001	0.00705336	1.28372	1.28372
Kcnab2	Kcnab2-008	0.0327106	2.63654	2.63654
Kcnc1	Kcnc1-002	0.0252749	0.614797	-1.62655
Kcnc1	Kcnc1-001	0.020611	0.676923	-1.47727
Kcnh3	Kcnh3-201	0.00994839	0.550553	-1.81636

Kcnh7	Kcnh7-001	0.0120349	1.44044	1.44044
Kcnip2	Kcnip2-201	0.0321357	0.373695	-2.67598
Kcnip2	Kcnip2-004	0.0103425	3.3452	3.3452
Kcnj6	Kcnj6-201	0.00909376	0.322859	-3.09733
Kcnk1	Kcnk1-201	0.0263554	1.26984	1.26984
Kcnmb4	Kcnmb4-001	6.18666e-05	0.596716	-1.67584
Kcnmb4	Kcnmb4-002	0.0142753	1.72378	1.72378
Kcnmb4os2	Kcnmb4os2-001	0.0485706	3.1534	3.1534
Kcnn3	Kcnn3-002	0.00492353	0.359761	-2.77962
Kcnn3	Kcnn3-001	0.0202748	0.524202	-1.90766
Kcnq2	Kcnq2-013	0.0292186	0.602578	-1.65954
Kcnq2	Kcnq2-018	0.0304173	0.662006	-1.51056
Kcnq4	Kcnq4-001	0.046693	3.48298	3.48298
Kcnq5	Kcnq5-008	0.0276609	48.2382	48.2382
Kcnu1	Kcnu1-001	0.0218181	4.13516	4.13516
Kctd13	Kctd13-002	0.0207022	1.57454	1.57454
Kctd18	Kctd18-010	0.0331336	0.484706	-2.0631
Kctd20	Kctd20-004	0.045537	2.98606	2.98606
Kctd20	Kctd20-201	0.00577119	4.50889	4.50889
Kctd20	Kctd20-002	0.0317322	5.37231	5.37231
Kctd3	Kctd3-006	0.0058468	0.227706	-4.39162
Kctd3	Kctd3-001	0.0335614	1.26101	1.26101
Kctd7	Kctd7-001	0.0238877	1.49223	1.49223
Kdm1a	Kdm1a-009	0.0127866	2.3451	2.3451
Kdm1b	Kdm1b-005	0.0181595	0.177019	-5.64912
Kdm3a	Kdm3a-004	0.0117563	0.677464	-1.47609
Kdm3a	Kdm3a-201	0.0435352	0.750113	-1.33313
Kdm3b	Kdm3b-201	0.00183212	0.840625	-1.18959
Kdm4a	Kdm4a-002	0.0164877	1.36029	1.36029
Kdm4a	Kdm4a-005	0.0261981	1.40843	1.40843
Kdm5a	Kdm5a-001	0.00760359	0.840755	-1.18941
Kdm5c	Kdm5c-004	0.00614398	1.26545	1.26545
Kdm5c	Kdm5c-005	0.045054	2.77625	2.77625
Kdm6b	Kdm6b-002	0.0215663	1.53251	1.53251
Khk	Khk-002	0.00207787	0.0234417	-42.659
Khsrp	Khsrp-201	0.00186103	1.13433	1.13433
Kidins220	Kidins220-201	0.00345986	0.830534	-1.20404
Kif17	Kif17-002	0.000454183	2.1538	2.1538
Kif18a	Kif18a-001	0.00120041	1.32287	1.32287
Kif1b	Kif1b-001	0.0140611	0.801667	-1.2474
Kif1b	Kif1b-003	0.0467271	2.56962	2.56962
Kif1bp	Kif1bp-006	0.0279828	0.368982	-2.71016
Kif1bp	Kif1bp-001	0.0265442	1.19906	1.19906

Kif1c	Kif1c-001	0.010045	0.206903	-4.83319
Kif24	Kif24-001	0.015308	0.818339	-1.22199
Kif26a	Kif26a-001	0.000174051	0.572422	-1.74696
Kif2a	Kif2a-001	0.0263752	0.712473	-1.40356
Kif2a	Kif2a-005	0.0210472	1.27532	1.27532
Kif5c	Kif5c-001	0.0168727	0.834331	-1.19856
Kifc1	Kifc1-005	0.0448874	1.79012	1.79012
Kifc3	Kifc3-203	0.0326372	0.793353	-1.26047
Kifc5b	Kifc5b-201	0.00748434	1.16124	1.16124
Klf3	Klf3-004	0.035256	0.597966	-1.67233
Klf4	Klf4-001	0.0324177	1.42171	1.42171
Klf7	Klf7-001	0.00380036	0.770607	-1.29768
Klhdc1	Klhdc1-001	0.0221982	1.67912	1.67912
Klhdc10	Klhdc10-001	0.000797683	0.564767	-1.77064
Klhdc10	Klhdc10-003	0.00572456	3.36111	3.36111
Klhdc2	Klhdc2-002	0.0215778	0.678098	-1.47471
Klhl12	Klhl12-201	0.016381	1.45955	1.45955
Klhl15	Klhl15-002	0.0368869	7.94097	7.94097
Klhl17	Klhl17-201	0.002275	1.17764	1.17764
Klhl18	Klhl18-002	0.0333324	2.08283	2.08283
Klhl18	Klhl18-006	0.0405016	5.11407	5.11407
Klhl20	Klhl20-003	0.0465107	1.92051	1.92051
Klhl3	Klhl3-001	0.019536	2.03119	2.03119
Klhl33	Klhl33-201	0.000825576	3.87305	3.87305
Klhl36	Klhl36-201	0.0435526	1.31457	1.31457
Klhl7	Klhl7-003	0.0118558	0.577269	-1.73229
Klhl8	Klhl8-004	0.026067	4.55595	4.55595
Klhl8	Klhl8-003	0.0420798	165.116	165.116
Kmt2a	Kmt2a-201	0.0281441	0.776607	-1.28765
Kmt2c	Kmt2c-006	0.016388	0.724996	-1.37932
Knop1	Knop1-001	0.00786157	3.21425	3.21425
Knop1	Knop1-004	0.0313149	4.22733	4.22733
Kntc1	Kntc1-007	0.0362477	0.616663	-1.62163
Kpna1	Kpna1-003	0.0498581	1.93926	1.93926
Kpna2	Kpna2-003	0.0430828	0.187262	-5.3401
Kptn	Kptn-001	0.00100263	0.466589	-2.14322
Kras	Kras-001	0.0459503	0.845546	-1.18267
Krba1	Krba1-004	0.0499321	1.43662	1.43662
Kremen1	Kremen1-001	0.000414763	0.596265	-1.67711
Krit1	Krit1-012	0.0399907	0.277129	-3.60842
Krit1	Krit1-004	0.0382083	0.54456	-1.83635
Krit1	Krit1-006	0.0106097	3.7208	3.7208
Krtcap2	Krtcap2-004	0.00106503	1.27804	1.27804

Ksr2	Ksr2-001	0.00356947	0.495703	-2.01734
Ktn1	Ktn1-005	0.0145291	0.453191	-2.20658
Ktn1	Ktn1-019	0.0309175	1.5648	1.5648
Ktn1	Ktn1-013	0.0310016	1.75776	1.75776
L1cam	L1cam-001	0.0462569	0.681912	-1.46647
L1cam	L1cam-008	0.0111845	0.710279	-1.4079
L1cam	L1cam-005	0.041107	4.15817	4.15817
l7Rn6	l7Rn6-005	0.0366241	0.288174	-3.47013
Lace1	Lace1-001	0.0388561	0.737132	-1.35661
Lactb	Lactb-201	0.0206458	0.692084	-1.44491
Lactb2	Lactb2-001	0.000887996	1.36656	1.36656
Lactb2	Lactb2-002	0.00413989	2.35013	2.35013
Lama4	Lama4-007	0.0187889	0.214825	-4.65494
Lama4	Lama4-001	0.00237363	0.537912	-1.85904
Lama5	Lama5-002	0.00632145	0.086887	-11.5092
Lamb2	Lamb2-001	0.000145655	0.313521	-3.18958
Lamc1	Lamc1-001	0.000750432	0.795581	-1.25694
Lamc2	Lamc2-004	0.000436235	0.152901	-6.54017
Lamc2	Lamc2-001	0.0017202	0.538664	-1.85644
Lamp2	Lamp2-002	6.7935e-06	0.38504	-2.59713
Lamp2	Lamp2-003	0.00149531	0.516131	-1.93749
Lancl1	Lancl1-003	0.000304681	1.40239	1.40239
Laptm4a	Laptm4a-001	0.0105834	0.845039	-1.18338
Large	Large-201	0.00973843	0.682832	-1.46449
Larp1	Larp1-003	0.0334001	1.87951	1.87951
Larp1b	Larp1b-201	0.0227324	0.126821	-7.8851
Larp1b	Larp1b-012	0.0415503	0.225567	-4.43327
Larp4	Larp4-201	0.04953	0.693677	-1.44159
Larp4b	Larp4b-001	0.0264638	1.29972	1.29972
Lars	Lars-201	0.00219946	1.50443	1.50443
Las1l	Las1l-001	0.00261466	1.50674	1.50674
Lasp1	Lasp1-010	0.0298139	0.348661	-2.86811
Lasp1	Lasp1-001	0.000542885	0.676685	-1.47779
Lats1	Lats1-201	0.00231334	2.02632	2.02632
Layn	Layn-201	0.0253987	1.36621	1.36621
Lca5	Lca5-001	0.012507	1.31672	1.31672
Lcmt1	Lcmt1-201	0.00152348	1.27456	1.27456
Ldah	Ldah-201	0.00206573	0.516109	-1.93758
Ldb1	Ldb1-003	0.0379774	0.494954	-2.02039
Ldha	Ldha-008	0.00112927	2.3075	2.3075
Ldlrad4	Ldlrad4-201	0.00190408	0.692465	-1.44412
Leng9	Leng9-001	0.0172491	1.58539	1.58539
Leprot	Leprot-001	0.0190255	0.828268	-1.20734

Leprotl1	Leprotl1-001	0.011784	1.59698	1.59698
Letm1	Letm1-003	0.0213699	1.72205	1.72205
Letmd1	Letmd1-201	0.00331989	1.23886	1.23886
Lfng	Lfng-001	0.00212694	0.605774	-1.65078
Lgals1	Lgals1-201	0.000680627	0.514183	-1.94483
Lgals3bp	Lgals3bp-001	2.61261e-05	0.775414	-1.28963
Lgals4	Lgals4-002	0.000240612	0.0220268	-45.3993
Lgals4	Lgals4-001	0.00111795	0.216244	-4.6244
Lgals7	Lgals7-003	0.0478431	0.327592	-3.05258
Lgalsl	Lgalsl-001	0.0250134	1.2119	1.2119
Lgi3	Lgi3-201	0.024302	1.76284	1.76284
Lhfp	Lhfp-002	0.0342609	492.505	492.505
Lhfpl2	Lhfpl2-001	0.00497297	1.99784	1.99784
Lhfpl2	Lhfpl2-003	0.000938676	3.29897	3.29897
Lhfpl4	Lhfpl4-001	0.00338642	1.41675	1.41675
Lhx1	Lhx1-004	0.0421425	0.274951	-3.63701
Lhx1	Lhx1-001	0.0408952	1.52835	1.52835
Lhx1os	Lhx1os-002	0.00817127	4.60169	4.60169
Lig3	Lig3-007	0.0211307	2.26058	2.26058
Lig3	Lig3-011	0.0311107	2.93864	2.93864
Lima1	Lima1-003	0.0036548	0.532146	-1.87919
Limch1	Limch1-019	0.0185849	1.64361	1.64361
Limd2	Limd2-001	7.88522e-05	0.616553	-1.62192
Limd2	Limd2-004	0.00277267	0.774547	-1.29108
Lime1	Lime1-001	0.0169944	0.656702	-1.52276
Limk2	Limk2-012	0.0180701	6.49851	6.49851
Lin54	Lin54-004	0.0493849	0.660106	-1.51491
Lin54	Lin54-008	0.040275	2.5578	2.5578
Lin7c	Lin7c-001	0.00350001	1.419	1.419
Lincenc1	Lincenc1-001	0.0413547	2.50638	2.50638
Lingo1	Lingo1-003	0.0145997	0.58644	-1.7052
Lingo3	Lingo3-201	0.0452965	1.52298	1.52298
Lins1	Lins1-002	0.0278683	2.08479	2.08479
Lipa	Lipa-201	0.00906439	2.16099	2.16099
Llgl1	Llgl1-003	0.0187627	2.54135	2.54135
Lman1	Lman1-001	0.000265512	1.13817	1.13817
Lman1	Lman1-002	0.000592112	1.43893	1.43893
Lman2l	Lman2l-004	0.035832	0.572033	-1.74815
Lman2l	Lman2l-001	0.00629564	0.735825	-1.35902
Lmbrd1	Lmbrd1-002	3.84221e-05	0.595881	-1.67819
Lmbrd1	Lmbrd1-004	0.00225593	2.59454	2.59454
Lmf1	Lmf1-005	0.00521779	1.43181	1.43181
Lmna	Lmna-006	9.83808e-05	0.600468	-1.66537

Lmna	Lmna-001	0.00201194	0.65678	-1.52258
Lmntd1	Lmntd1-003	0.0458075	0.204673	-4.88584
Lmo4	Lmo4-005	0.0431651	3.6976	3.6976
Lmtk2	Lmtk2-001	0.026433	0.908966	-1.10015
Lmtk3	Lmtk3-007	0.00404351	5.53157	5.53157
Lmx1a	Lmx1a-001	0.0306188	3.14358	3.14358
Lmx1a	Lmx1a-002	0.0306188	3.14358	3.14358
Lnp	Lnp-002	0.0326006	1.30638	1.30638
Lnp	Lnp-004	0.0068559	1.44269	1.44269
Lonp2	Lonp2-004	0.028966	0.462695	-2.16125
Lonrf1	Lonrf1-201	0.00369472	1.1669	1.1669
Loxl2	Loxl2-001	0.00759052	0.636289	-1.57161
Loxl3	Loxl3-001	0.00284616	0.452426	-2.21031
Loxl3	Loxl3-002	0.0286842	36.8141	36.8141
Lpcat1	Lpcat1-003	0.0250455	0.729173	-1.37142
Lpcat1	Lpcat1-002	0.0154066	3.67727	3.67727
Lpgat1	Lpgat1-005	0.0451118	4.15103	4.15103
Lpin2	Lpin2-005	0.000354044	6.80405	6.80405
Lpin3	Lpin3-004	0.0363997	5.90305	5.90305
Lpp	Lpp-201	0.0236574	0.476524	-2.09853
Lppr4	Lppr4-003	0.0102098	4.05522	4.05522
Lrba	Lrba-005	0.0102225	0.275601	-3.62843
Lrch2	Lrch2-201	0.0494366	1.21904	1.21904
Lrch2	Lrch2-001	0.049424	1.21905	1.21905
Lrch2	Lrch2-002	0.0344992	1.50851	1.50851
Lrch4	Lrch4-002	0.0133865	3.66023	3.66023
Lrif1	Lrif1-012	0.00241912	1.46569	1.46569
Lrig2	Lrig2-001	0.00269253	0.706228	-1.41597
Lrig2	Lrig2-002	0.0381974	2.02241	2.02241
Lrp1	Lrp1-001	0.00925305	0.558834	-1.78944
Lrp10	Lrp10-201	0.00215856	0.810658	-1.23357
Lrp11	Lrp11-005	0.0411599	0.342899	-2.91631
Lrp6	Lrp6-001	0.0368815	1.1004	1.1004
Lrp8	Lrp8-008	0.00590245	5.38064	5.38064
Lrp8	Lrp8-009	0.00111654	8.87622	8.87622
Lrrc14	Lrrc14-007	0.025227	3.87665	3.87665
Lrrc23	Lrrc23-004	0.0335535	2.53517	2.53517
Lrrc28	Lrrc28-001	0.0181731	1.99901	1.99901
Lrrc36	Lrrc36-001	0.0109032	2.01228	2.01228
Lrrc4	Lrrc4-001	0.0408074	0.688166	-1.45314
Lrrc41	Lrrc41-003	0.0380842	9.66127e+08	9.66127e+08
Lrrc45	Lrrc45-003	0.0131019	3.01992	3.01992
Lrrc51	Lrrc51-003	0.0132322	3.65854	3.65854

Lrrc55	Lrrc55-202	0.00532898	1.73291	1.73291
Lrrc61	Lrrc61-003	0.0228779	1.75674	1.75674
Lrrc8b	Lrrc8b-001	0.00132739	0.701758	-1.42499
Lrrc8c	Lrrc8c-001	0.000570566	0.595356	-1.67967
Lrrc8c	Lrrc8c-004	0.036495	2.75003	2.75003
Lrrcc1	Lrrcc1-002	0.0258333	1.70009	1.70009
Lrrfip2	Lrrfip2-005	0.000114749	1.67722	1.67722
Lrriq3	Lrriq3-002	0.0171989	3.25169	3.25169
Lrwd1	Lrwd1-008	0.0492048	11.2375	11.2375
Lsg1	Lsg1-004	0.011836	1.5669	1.5669
Lsg1	Lsg1-006	0.0375248	1.63751	1.63751
Lsg1	Lsg1-007	0.000714365	3.55807	3.55807
Lsg1	Lsg1-002	0.000114839	3.60169	3.60169
Lsm11	Lsm11-001	0.000820288	0.672083	-1.48791
Lsm11	Lsm11-002	0.0316901	1.97145	1.97145
Lsm12	Lsm12-001	0.00425132	1.20703	1.20703
Lsm14a	Lsm14a-006	0.00105409	1.20138	1.20138
Lsm2	Lsm2-008	0.0135002	0.117807	-8.48847
Lsm6	Lsm6-004	0.045471	1.42995	1.42995
Lsp1	Lsp1-005	0.0222019	0.739759	-1.35179
Lsp1	Lsp1-012	0.0242721	4.74567	4.74567
Lss	Lss-005	0.0460115	274.26	274.26
Lta4h	Lta4h-201	0.000362732	1.21137	1.21137
Ltbp2	Ltbp2-001	0.0484046	0.30286	-3.30186
Ltbp3	Ltbp3-201	1.66848e-05	0.596902	-1.67532
Ltbp4	Ltbp4-002	0.000261989	0.491382	-2.03508
Ltbp4	Ltbp4-201	0.00136813	0.518694	-1.92792
Ltbp4	Ltbp4-001	0.0108519	0.530276	-1.88581
Luc7l	Luc7l-009	0.00181315	77.2425	77.2425
Luc7l3	Luc7l3-009	0.0197353	1.6789	1.6789
Lurap1	Lurap1-001	0.00954509	2.10877	2.10877
Luzp4	Luzp4-001	0.0374668	0.588516	-1.69919
Ly6g5b	Ly6g5b-001	0.0494098	0.297663	-3.3595
Ly6g6c	Ly6g6c-001	0.0355502	0.406091	-2.4625
Ly75	Ly75-001	0.00923705	0.713801	-1.40095
Lyar	Lyar-010	0.0128597	2.01064	2.01064
Lypla1	Lypla1-001	0.010006	1.26657	1.26657
Lypla2	Lypla2-002	0.00255614	0.586141	-1.70607
Lypla2	Lypla2-001	0.0320404	0.796393	-1.25566
Lypla2	Lypla2-005	0.00279788	1.97449	1.97449
Lyplal1	Lyplal1-001	0.0274962	1.31281	1.31281
Lyplal1	Lyplal1-002	0.021953	3.74649	3.74649
Lyrm4	Lyrm4-201	0.0111596	1.36785	1.36785

Lyrm5	Lyrm5-003	0.0258718	15.5682	15.5682
Lyrm7	Lyrm7-002	0.0354751	7.26886	7.26886
Lyrm7	Lyrm7-001	0.0415665	14.0034	14.0034
Lyrm9	Lyrm9-001	0.000149626	1.40931	1.40931
Lysmd3	Lysmd3-201	0.00111047	1.22588	1.22588
Lztfl1	Lztfl1-006	0.0153856	1.96885	1.96885
Lztr1	Lztr1-201	0.00339029	0.797738	-1.25354
Lztr1	Lztr1-002	0.0146059	1.63609	1.63609
Lzts3	Lzts3-001	0.0250067	0.335364	-2.98184
Macf1	Macf1-002	0.0145518	0.466444	-2.14388
Macf1	Macf1-008	0.0010563	0.692178	-1.44471
Mad1l1	Mad1l1-003	0.0106748	11.9904	11.9904
Madcam1	Madcam1-201	0.00111837	0.199651	-5.00873
Madd	Madd-021	0.0373831	0.725869	-1.37766
Mafb	Mafb-001	0.00777419	0.575939	-1.73629
Mafg	Mafg-004	0.0272502	1.34292	1.34292
Mafg	Mafg-005	0.00372311	1.54223	1.54223
Magea2	Magea2-201	0.0237188	10.5832	10.5832
Mageh1	Mageh1-001	0.00142205	1.23837	1.23837
Magel2	Magel2-201	0.0470162	1.65401	1.65401
Magi1	Magi1-201	0.0203583	0.374054	-2.67341
Magohb	Magohb-005	0.0473283	0.710682	-1.4071
Malsu1	Malsu1-003	0.00373964	1.37784	1.37784
Malsu1	Malsu1-004	0.010014	1.71808	1.71808
Maml2	Maml2-002	0.0381372	0.0635759	-15.7292
Maml2	Maml2-001	0.00557514	0.744446	-1.34328
Man1a2	Man1a2-006	0.0175493	1.78218	1.78218
Man1b1	Man1b1-001	0.0103139	1.18084	1.18084
Man1c1	Man1c1-001	0.000557859	0.546593	-1.82952
Man1c1	Man1c1-201	0.00656831	0.552617	-1.80957
Man2b1	Man2b1-201	0.00205562	0.698312	-1.43202
Man2c1	Man2c1-019	0.0415496	0.630455	-1.58616
Man2c1	Man2c1-011	0.0010096	2.04923	2.04923
Man2c1	Man2c1-017	0.00474036	2.88808	2.88808
Manba	Manba-002	0.0310074	0.236073	-4.23598
Manba	Manba-001	0.0218724	0.763578	-1.30962
Manbal	Manbal-001	0.000421878	1.32159	1.32159
Manf	Manf-003	0.0336636	2.88187	2.88187
Map1a	Map1a-002	0.000232557	0.898941	-1.11242
Map1lc3b	Map1lc3b-001	0.0232193	0.848212	-1.17895
Map1lc3b	Map1lc3b-003	0.0277485	1.34455	1.34455
Map2	Map2-010	0.00977316	2.13852	2.13852
Map2	Map2-018	0.00726081	28.7954	28.7954

Map3k10	Map3k10-004	0.0438787	5.43313	5.43313
Map3k13	Map3k13-201	0.00186161	1.69832	1.69832
Map3k2	Map3k2-201	0.00297042	1.27215	1.27215
Map3k3	Map3k3-001	1.35369e-05	0.523464	-1.91035
Map3k5	Map3k5-007	0.00882876	0.000430453	-2323.13
Map3k5	Map3k5-001	0.00647784	0.707678	-1.41307
Map3k6	Map3k6-001	0.0142646	0.611719	-1.63474
Map3k7	Map3k7-003	0.0134884	0.539942	-1.85205
Map4	Map4-006	0.0149474	0.152686	-6.54938
Map4k1	Map4k1-201	0.0013053	0.621215	-1.60975
Map4k3	Map4k3-201	0.0124176	1.27753	1.27753
Map4k4	Map4k4-019	0.000147169	0.56445	-1.77163
Map4k4	Map4k4-201	0.0170952	0.667835	-1.49738
Map4k4	Map4k4-002	0.0065761	1.62831	1.62831
Map4k4	Map4k4-004	0.00565242	3.35046	3.35046
Map4k5	Map4k5-202	0.0297934	2.48702	2.48702
Map6	Map6-004	0.000554131	0.625796	-1.59796
Map6	Map6-002	0.000186984	0.67123	-1.4898
Map6d1	Map6d1-201	3.23535e-06	2.55383	2.55383
Map7d2	Map7d2-003	0.0181	1.3467	1.3467
Map7d2	Map7d2-001	0.000348634	1.40675	1.40675
Map7d2	Map7d2-002	0.00152615	1.69157	1.69157
Map9	Map9-001	0.0222285	1.2327	1.2327
Mapk1	Mapk1-001	8.40348e-08	0.489313	-2.04368
Mapk1	Mapk1-006	0.00204388	12.7439	12.7439
Mapk10	Mapk10-003	0.0333236	0.279128	-3.58259
Mapk10	Mapk10-001	0.0448075	0.673464	-1.48486
Mapk10	Mapk10-006	0.0122734	1.79136	1.79136
Mapk11	Mapk11-201	0.017035	0.684516	-1.46089
Mapk12	Mapk12-201	4.47171e-05	0.749731	-1.33381
Mapk15	Mapk15-001	0.00260222	4.01816	4.01816
Mapk1ip1l	Mapk1ip1l-201	0.00173369	0.540389	-1.85052
Mapk6	Mapk6-001	0.0492188	0.81826	-1.2221
Mapk8	Mapk8-001	0.00437639	0.67851	-1.47382
Mapk8	Mapk8-009	0.0319916	1.76563	1.76563
Mapk8	Mapk8-003	0.00081022	4.92344	4.92344
Mapk8ip1	Mapk8ip1-001	0.000191948	1.1981	1.1981
Mapk8ip3	Mapk8ip3-012	0.0311293	0.000183473	-5450.39
Mapk8ip3	Mapk8ip3-001	0.0472788	0.703881	-1.4207
Mapk8ip3	Mapk8ip3-004	0.0450412	0.782365	-1.27818
Mapk9	Mapk9-202	0.0398877	0.760033	-1.31573
Mapk9	Mapk9-002	0.000463415	1.92113	1.92113
Mapkap1	Mapkap1-004	0.00119731	4.84396	4.84396

Mapkapk2	Mapkapk2-001	1.42712e-07	0.441153	-2.26679
Mapkapk3	Mapkapk3-004	0.0279726	4.44331e+07	4.44331e+07
Mapre2	Mapre2-201	0.0412709	0.043328	-23.0798
Mapre2	Mapre2-001	0.00914827	0.797588	-1.25378
Mapre2	Mapre2-006	0.0159764	1.36605	1.36605
Mapt	Mapt-003	0.0270963	0.538758	-1.85612
Marc2	Marc2-003	0.0339746	1.40979	1.40979
March3	March3-002	0.00346757	0.488394	-2.04753
March4	March4-001	0.0460567	0.638625	-1.56586
March6	March6-201	0.00224324	1.19872	1.19872
March7	March7-003	0.0346976	2.33423	2.33423
March7	March7-201	0.0391323	2.79639	2.79639
March11	March11-002	0.00288529	0.473344	-2.11263
Mark2	Mark2-015	0.00838779	2.66879	2.66879
Mark3	Mark3-202	0.017074	0.832893	-1.20063
Mark4	Mark4-201	0.0143405	0.790878	-1.26442
Mars	Mars-001	0.0481479	1.39066	1.39066
Masp1	Masp1-201	0.000577414	2.0517	2.0517
Mat2a	Mat2a-002	0.000565247	1.18484	1.18484
Mat2a	Mat2a-003	0.0075937	4.51313	4.51313
Mat2b	Mat2b-001	0.0499678	0.511688	-1.95432
Mat2b	Mat2b-002	0.0103313	0.639391	-1.56399
Matr3	Matr3-010	0.0469791	0.517719	-1.93155
Matr3	Matr3-003	0.0452934	4.12622	4.12622
Max	Max-201	0.00833169	1.37292	1.37292
Mbd4	Mbd4-001	0.0141558	1.31048	1.31048
Mbd4	Mbd4-004	0.0115456	1.47315	1.47315
Mbd4	Mbd4-003	0.0142662	1.53087	1.53087
Mbd5	Mbd5-010	0.0224392	2.41781	2.41781
Mbd6	Mbd6-001	0.0495209	1.25771	1.25771
Mblac2	Mblac2-201	0.0307252	0.726763	-1.37596
Mbnl1	Mbnl1-018	0.0412231	0.123207	-8.11642
Mbnl1	Mbnl1-002	0.0411405	0.318889	-3.13589
Mboat4	Mboat4-201	4.60001e-05	2.68888	2.68888
Mboat7	Mboat7-001	0.00187633	0.754475	-1.32543
Mbtd1	Mbtd1-012	0.0196297	0.292072	-3.42382
Mbtd1	Mbtd1-002	0.00462903	0.450962	-2.21748
Mbtps2	Mbtps2-001	6.48658e-05	0.533787	-1.87341
Mbtps2	Mbtps2-003	0.0479008	1.61792	1.61792
Mbtps2	Mbtps2-201	0.0478973	1.61793	1.61793
Mbtps2	Mbtps2-004	7.53858e-05	1.70829	1.70829
Mbtps2	Mbtps2-005	0.0373971	3.46783	3.46783
Mcam	Mcam-001	0.00125755	0.684001	-1.46199

Mcee	Mcee-004	0.0447482	0.0641984	-15.5767
Mcee	Mcee-001	0.00622859	1.42561	1.42561
Mcemp1	Mcemp1-201	0.0285255	2.18482	2.18482
Mcf2l	Mcf2l-013	0.0263829	3.39428	3.39428
Mcfd2	Mcfd2-001	0.0177856	0.80393	-1.24389
Mcm8	Mcm8-002	0.00211431	1.55415	1.55415
Mcmbp	Mcmbp-002	0.019148	1.30756	1.30756
Mcoln1	Mcoln1-001	0.0270023	0.847992	-1.17926
Mcoln1	Mcoln1-007	0.025689	1.39833	1.39833
Mcoln3	Mcoln3-001	0.037941	0.745289	-1.34176
Mcph1	Mcph1-004	0.00336089	2.14883	2.14883
Mcrs1	Mcrs1-201	0.0401329	1.17411	1.17411
Mcts1	Mcts1-003	0.00914251	0.305699	-3.2712
Mcts2	Mcts2-001	0.0242186	1.23706	1.23706
Mcu	Mcu-001	0.00894275	1.28228	1.28228
Mcu	Mcu-003	0.0473897	2.32778	2.32778
Mdfi	Mdfi-008	0.0354901	85.3423	85.3423
Mdh1b	Mdh1b-001	0.0173428	6.83268	6.83268
Mdk	Mdk-003	0.0351411	0.384955	-2.59771
Mdm2	Mdm2-003	0.000484773	1.41174	1.41174
Mdm2	Mdm2-002	0.00205274	1.80995	1.80995
Mdp1	Mdp1-201	0.00346316	1.36868	1.36868
Me1	Me1-001	0.0210381	1.3572	1.3572
Me1	Me1-003	0.00143838	1.51548	1.51548
Mea1	Mea1-201	0.0263917	1.21889	1.21889
Mecp2	Mecp2-201	0.0306421	0.63129	-1.58406
Mecp2	Mecp2-003	0.0159192	2.25596	2.25596
Mecp2	Mecp2-002	0.0240581	2.32219	2.32219
Mecr	Mecr-001	0.013432	1.6769	1.6769
Med1	Med1-004	0.00926629	2.03198	2.03198
Med1	Med1-002	0.0296356	2.83899	2.83899
Med13	Med13-002	7.57363e-05	3.04677e-05	-32821.6
Med13	Med13-003	0.039603	65.2491	65.2491
Med16	Med16-001	0.0233189	1.28597	1.28597
Med23	Med23-006	0.0286085	1.37622	1.37622
Med23	Med23-004	7.63948e-05	2.44288	2.44288
Med24	Med24-009	0.00144838	1.81831	1.81831
Med28	Med28-001	0.0241819	0.589092	-1.69753
Med30	Med30-201	0.00346242	1.30786	1.30786
Med31	Med31-002	0.00174972	0.493604	-2.02592
Med31	Med31-001	0.0185677	1.33285	1.33285
Med8	Med8-008	0.000167564	8.85812	8.85812
Med9os	Med9os-003	0.0310589	1.47009	1.47009

Medag	Medag-003	0.0341358	0.583743	-1.71308
Mef2a	Mef2a-002	0.000189258	0.478129	-2.09148
Mef2a	Mef2a-004	0.00808313	0.548218	-1.82409
Mef2b	Mef2b-005	0.0234003	2.07559	2.07559
Mef2d	Mef2d-001	0.0118134	0.711675	-1.40514
Mef2d	Mef2d-002	0.0308065	1.23983	1.23983
Megf10	Megf10-001	0.00345813	0.425177	-2.35196
Megf8	Megf8-002	0.000779824	0.575668	-1.73711
Megf8	Megf8-001	0.000725771	0.683025	-1.46408
Meis1	Meis1-006	0.000247401	1.63274	1.63274
Meis2	Meis2-011	0.017462	0.560885	-1.7829
Meis3	Meis3-010	0.0341671	3.69229	3.69229
Melk	Melk-001	0.00295696	0.679773	-1.47108
Mepce	Mepce-002	0.0445426	0.466882	-2.14187
Metap1d	Metap1d-004	0.0304311	2.0764	2.0764
Metap2	Metap2-002	0.00763259	0.355788	-2.81066
Metap2	Metap2-001	0.00150272	0.50636	-1.97488
Metap2	Metap2-014	0.0181032	67.7055	67.7055
Metap2	Metap2-012	8.83982e-05	3453.96	3453.96
Metrn	Metrn-201	0.0037689	0.493591	-2.02597
Metrn	Metrn-202	0.0168364	0.586634	-1.70464
Mettl14	Mettl14-001	0.00138513	1.23158	1.23158
Mettl14	Mettl14-002	0.00410196	4.65061	4.65061
Mettl15	Mettl15-001	0.000528673	1.49377	1.49377
Mettl21b	Mettl21b-001	0.0171572	1.46963	1.46963
Mettl21c	Mettl21c-002	0.0297622	5.52341	5.52341
Mettl24	Mettl24-001	0.0295324	1.64673	1.64673
Mettl3	Mettl3-001	0.0267516	1.25213	1.25213
Mettl4	Mettl4-201	0.00199269	1.53791	1.53791
Mex3a	Mex3a-001	0.00889401	0.50278	-1.98894
Mex3c	Mex3c-001	0.00022351	0.572423	-1.74696
Mex3d	Mex3d-002	0.0249193	0.33651	-2.97168
Mfap1b	Mfap1b-001	0.0398032	1.32209	1.32209
Mfap2	Mfap2-001	0.00103652	0.547352	-1.82698
Mff	Mff-010	0.0371269	10.3437	10.3437
Mfng	Mfng-003	0.0221639	3.15553	3.15553
Mfrp	Mfrp-005	0.00128638	0.0634086	-15.7707
Mfsd11	Mfsd11-003	0.0214874	0.0517758	-19.314
Mfsd11	Mfsd11-004	0.0315332	0.596092	-1.67759
Mfsd12	Mfsd12-001	0.000923807	0.59571	-1.67867
Mfsd6	Mfsd6-001	0.0462429	0.820412	-1.2189
Mfsd6	Mfsd6-004	0.00493552	2.28031	2.28031
Mfsd7b	Mfsd7b-005	0.0116454	2.04726	2.04726

Mfsd8	Mfsd8-001	0.0217927	0.802888	-1.2455
Mga	Mga-004	0.0296918	5.68082e-08	-1.76031e+07
Mgat1	Mgat1-003	0.02224	3.51508	3.51508
Mgat1	Mgat1-012	0.00654767	7.6418	7.6418
Mgat3	Mgat3-201	0.0267357	0.850761	-1.17542
Mgat4a	Mgat4a-003	0.0295278	5.4629e-10	-1.83053e+09
Mgat4b	Mgat4b-003	0.0289231	1.33432	1.33432
Mgat4b	Mgat4b-002	0.0113143	4.90287	4.90287
Mgat5	Mgat5-001	0.00167492	0.728346	-1.37297
Mia3	Mia3-004	0.0224619	0.687153	-1.45528
Mia3	Mia3-201	0.032144	0.696192	-1.43639
Mia3	Mia3-001	0.00168766	0.715151	-1.39831
Mia3	Mia3-003	0.00462106	0.720293	-1.38832
Mia3	Mia3-002	0.0406426	0.832796	-1.20077
Mib1	Mib1-001	0.000985699	1.35803	1.35803
Mib1	Mib1-201	0.0129319	1.98033	1.98033
Mib2	Mib2-001	0.0421127	1.3214	1.3214
Mib2	Mib2-002	0.0194589	1.74022	1.74022
Mib2	Mib2-003	0.0280742	2.95882	2.95882
Mical2	Mical2-005	0.0111357	0.307212	-3.25508
Mical2	Mical2-001	0.0183493	0.560837	-1.78305
Mical3	Mical3-013	0.0480513	0.301496	-3.31679
Micall2	Micall2-003	0.00390072	0.154917	-6.45508
Micall2	Micall2-006	0.00104184	0.162479	-6.15466
Micall2	Micall2-001	0.000637652	0.396049	-2.52494
Micu1	Micu1-001	0.0393213	1.48829	1.48829
Micu1	Micu1-003	0.0198585	1.74127	1.74127
Micu1	Micu1-011	0.0307047	374445	374445
Mid1ip1	Mid1ip1-002	0.00289448	1.51292	1.51292
Mid1ip1	Mid1ip1-001	0.0338139	1.92889	1.92889
Mid2	Mid2-201	0.0290866	0.668293	-1.49635
Midn	Midn-001	0.0408191	0.803784	-1.24412
Mien1	Mien1-001	0.00657282	1.17975	1.17975
Mier2	Mier2-201	0.021411	0.209743	-4.76775
Mier2	Mier2-002	0.0194342	1.33178	1.33178
Mier2	Mier2-003	0.000444915	5.13571	5.13571
Miip	Miip-005	0.0140162	0.714211	-1.40015
Milr1	Milr1-004	0.0171805	0.192175	-5.2036
Milr1	Milr1-006	0.0073711	0.234688	-4.26097
Milr1	Milr1-001	0.0345067	0.542791	-1.84233
Mipep	Mipep-201	9.6687e-05	1.48529	1.48529
Mipol1	Mipol1-006	0.0126869	2.04348	2.04348
Mir124a-1hg	Mir124a-1hg-001	0.00722173	1.45207	1.45207

Mir6955	Mir6955-201	0.00608952	7.81375	7.81375
Mirg	Mirg-002	0.0230607	0.28956	-3.45352
Mis18a	Mis18a-002	0.0217111	0.570077	-1.75415
Mitd1	Mitd1-001	0.019492	1.33682	1.33682
Mitf	Mitf-001	0.000959372	1.72086	1.72086
Mkks	Mkks-003	0.00833998	1.35028	1.35028
Mkln1	Mkln1-001	0.00222962	0.724694	-1.37989
Mkln1	Mkln1-005	0.0221657	1.23983	1.23983
Mkln1	Mkln1-010	0.0304406	1.24125	1.24125
Mknk1	Mknk1-001	0.0055684	1.49888	1.49888
Mknk1	Mknk1-013	0.00629487	1.79548	1.79548
Mknk1	Mknk1-002	0.023553	1.8842	1.8842
Mknk1	Mknk1-003	0.0314985	4.44015	4.44015
Mknk2	Mknk2-201	0.00283429	0.54045	-1.85031
Mkrn1	Mkrn1-001	0.0163032	1.15852	1.15852
Mkrn1	Mkrn1-002	0.0052794	3.23367	3.23367
Mkrn2	Mkrn2-001	0.00953429	1.19178	1.19178
Mkrn3	Mkrn3-201	0.000807457	1.42645	1.42645
Mkx	Mkx-001	0.0238791	1.48552	1.48552
Mkx	Mkx-005	0.045743	8.97341	8.97341
Mlh3	Mlh3-201	0.0152593	1.34689	1.34689
Mlh3	Mlh3-202	0.0110784	1.35521	1.35521
Mlkl	Mlkl-001	0.0298893	1.65579	1.65579
Mllt10	Mllt10-002	0.012278	1.54057	1.54057
Mllt11	Mllt11-003	0.0446774	22.364	22.364
Mllt6	Mllt6-004	0.000204008	0.809971	-1.23461
Mlx	Mlx-001	0.00389022	1.81767	1.81767
Mmaa	Mmaa-201	0.00141104	2.32635	2.32635
Mmadhc	Mmadhc-001	0.000129205	1.32897	1.32897
Mmd	Mmd-001	0.0184137	0.802068	-1.24678
Mmd	Mmd-004	0.0054543	1.97475	1.97475
Mmp23	Mmp23-001	0.00175962	0.385772	-2.5922
Mmp24	Mmp24-001	0.0272919	1.19981	1.19981
Mmp9	Mmp9-001	0.00172422	0.150799	-6.63136
mmu-mir-1291	mmu-mir-1291.1-201	0.0343506	0.255377	-3.91578
Mob1a	Mob1a-002	0.00708015	0.575589	-1.73735
Mob1b	Mob1b-004	0.0337371	2.51929	2.51929
Mob3a	Mob3a-201	0.0249034	0.781625	-1.27939
Mob3c	Mob3c-001	0.0378349	1.26517	1.26517
Mocs2	Mocs2-002	0.00740739	1.72674	1.72674
Morc3	Morc3-001	0.000377559	1.59585	1.59585
Morc3	Morc3-201	0.000800153	1.60332	1.60332
Morc3	Morc3-004	0.0360913	3.21316	3.21316

Morc3	Morc3-005	5.7547e-05	4.53978	4.53978
Morf4l2	Morf4l2-003	0.00133589	1.20804	1.20804
Morf4l2	Morf4l2-001	0.00739224	1.2587	1.2587
Morf4l2	Morf4l2-201	0.0145084	1.31415	1.31415
Morf4l2	Morf4l2-204	0.0104416	1.45248	1.45248
Morn2	Morn2-201	0.00725254	1.62395	1.62395
Mospd2	Mospd2-002	0.0231268	1.36088	1.36088
Mov10	Mov10-005	0.0432911	7850.74	7850.74
Mpc2	Mpc2-003	0.0233701	1.50425	1.50425
Mphosph6	Mphosph6-201	0.0012769	1.29213	1.29213
Mplkip	Mplkip-201	0.0279044	1.20801	1.20801
Mpnd	Mpnd-001	0.0293513	0.596968	-1.67513
Mpp3	Mpp3-008	0.0372793	1.6142	1.6142
Mpp4	Mpp4-001	0.0222627	0.00014441	-6924.74
Mpp5	Mpp5-201	7.47308e-07	1.3755	1.3755
Mpp6	Mpp6-003	0.0159121	1.49667	1.49667
Mpp6	Mpp6-002	0.00160119	1.51302	1.51302
Mprip	Mprip-001	0.00450342	0.634263	-1.57663
Mprip	Mprip-003	0.0274513	0.775423	-1.28962
Mpst	Mpst-203	0.0390088	0.511056	-1.95673
Mpv17l	Mpv17l-001	0.0250418	0.379621	-2.63421
Mpv17l	Mpv17l-002	0.00502139	1.46387	1.46387
Mpv17l2	Mpv17l2-201	0.0271082	0.750447	-1.33254
Mpzl1	Mpzl1-001	0.0110129	0.683712	-1.4626
Mpzl1	Mpzl1-002	0.00438961	1.44664	1.44664
Mpzl1	Mpzl1-006	0.0298018	163.029	163.029
Mrap2	Mrap2-003	0.0274751	1.76966	1.76966
Mrc2	Mrc2-005	0.0288654	0.0118663	-84.272
Mre11a	Mre11a-001	0.000299186	1.48109	1.48109
Mrgprx2	Mrgprx2-001	0.00104715	0.544761	-1.83567
Mroh1	Mroh1-008	0.0449598	1.78096	1.78096
Mrpl1	Mrpl1-002	5.56097e-05	1.63386	1.63386
Mrpl10	Mrpl10-002	0.0403639	2.22588	2.22588
Mrpl13	Mrpl13-003	0.0368477	1.32372	1.32372
Mrpl15	Mrpl15-006	0.0214076	1.24315	1.24315
Mrpl24	Mrpl24-005	0.00501438	1.51275	1.51275
Mrpl27	Mrpl27-001	0.00654761	1.32707	1.32707
Mrpl30	Mrpl30-007	0.00837964	1.20503	1.20503
Mrpl30	Mrpl30-008	0.000677242	1.47528	1.47528
Mrpl32	Mrpl32-201	0.000135741	1.27565	1.27565
Mrpl33	Mrpl33-006	0.0373977	1.55141	1.55141
Mrpl33	Mrpl33-003	0.0107912	1.86462	1.86462
Mrpl35	Mrpl35-001	0.0385378	1.226	1.226

Mrpl35	Mrpl35-002	0.0274931	1.42935	1.42935
Mrpl47	Mrpl47-002	0.0337609	1.462	1.462
Mrpl48	Mrpl48-008	0.0143507	1.27239	1.27239
Mrpl50	Mrpl50-001	0.0303259	1.14951	1.14951
Mrpl53	Mrpl53-001	0.00258079	1.3939	1.3939
Mrpl57	Mrpl57-201	0.00361355	1.32369	1.32369
Mrps14	Mrps14-003	0.00481271	1.26093	1.26093
Mrps18b	Mrps18b-008	0.039389	2.44376	2.44376
Mrps22	Mrps22-001	0.0179055	1.40259	1.40259
Mrps31	Mrps31-201	0.00125177	1.39645	1.39645
Mrps34	Mrps34-002	0.0415411	1.70684	1.70684
Mrps35	Mrps35-004	0.0226968	3.55825	3.55825
Mrps36	Mrps36-002	0.0128547	1.52119	1.52119
Mrps5	Mrps5-007	0.0399102	1.39473	1.39473
Mrps5	Mrps5-003	0.00675284	1.52238	1.52238
Mrps5	Mrps5-005	0.00222194	1.5745	1.5745
Mrps5	Mrps5-004	0.00854059	1.84359	1.84359
Mrps5	Mrps5-002	0.00697293	1.98291	1.98291
Msantd2	Msantd2-201	0.0352178	0.718842	-1.39113
Msantd3	Msantd3-003	0.0166262	1.49786	1.49786
Msi1	Msi1-001	0.00523566	0.810364	-1.23401
Msi1	Msi1-003	0.0110167	2.74999	2.74999
Msi2	Msi2-001	0.02784	0.780991	-1.28042
Msl3	Msl3-004	0.0367061	7.42841	7.42841
Msl3l2	Msl3l2-201	0.0344041	0.646114	-1.54771
Msrb3	Msrb3-002	0.0461933	1.0793e-06	-926527
Msto1	Msto1-005	0.0420741	1.78307	1.78307
Msto1	Msto1-002	0.0313977	2.04642	2.04642
Mt1	Mt1-201	0.0149904	1.67036	1.67036
Mt3	Mt3-201	0.0109397	1.71854	1.71854
Mta2	Mta2-006	0.00899985	0.194863	-5.13181
Mta3	Mta3-002	0.00973761	2.70762	2.70762
Mtbp	Mtbp-001	0.00529991	1.72624	1.72624
Mtbp	Mtbp-005	0.020812	1.89829	1.89829
Mtch1	Mtch1-006	0.0454984	0.622083	-1.6075
Mtch1	Mtch1-002	0.0224715	0.663776	-1.50653
Mtcl1	Mtcl1-004	0.00387462	1.7032	1.7032
Mterf2	Mterf2-201	0.0177454	1.33943	1.33943
Mterf3	Mterf3-001	0.03043	1.33828	1.33828
Mterf3	Mterf3-010	0.00224848	3.7545	3.7545
Mterf4	Mterf4-001	0.00101922	1.52593	1.52593
Mtf2	Mtf2-003	0.00702059	0.502641	-1.98949
Mtf2	Mtf2-001	0.000321768	0.717373	-1.39398

Mtf2	Mtf2-005	0.0172916	6.03539	6.03539
Mtfmt	Mtfmt-001	0.00889229	1.27935	1.27935
Mtfr1	Mtfr1-003	0.000337404	5.62074	5.62074
Mtfr1l	Mtfr1l-005	0.0250432	2.67701	2.67701
Mtg1	Mtg1-006	0.0129019	2.43758	2.43758
Mtg2	Mtg2-001	0.0170639	1.91428	1.91428
Mthfd2	Mthfd2-001	0.0100829	0.838074	-1.19321
Mthfd2l	Mthfd2l-001	0.0030886	0.640387	-1.56156
Mthfr	Mthfr-002	0.00495473	1.78496	1.78496
Mthfr	Mthfr-001	0.00395694	1.7884	1.7884
Mthfr	Mthfr-007	0.00582166	1.86465	1.86465
Mthfs	Mthfs-004	0.0198679	15.7593	15.7593
Mthfsd	Mthfsd-005	0.0342637	2.27403	2.27403
Mthfsl	Mthfsl-001	0.0353712	1.67346	1.67346
Mtif2	Mtif2-201	0.00659899	1.3161	1.3161
Mtif2	Mtif2-003	0.0461203	7.41659	7.41659
Mtm1	Mtm1-201	0.0204818	0.220241	-4.54049
Mtm1	Mtm1-003	0.0204818	0.220241	-4.54048
Mtmr11	Mtmr11-001	0.0399217	0.568297	-1.75964
Mtmr3	Mtmr3-004	0.00264984	1.32726	1.32726
Mtmr4	Mtmr4-003	0.00795747	2.84008	2.84008
Mtmr7	Mtmr7-001	0.000147576	1.43824	1.43824
Mtor	Mtor-002	0.0101965	3.14266	3.14266
Mtpap	Mtpap-201	0.00176433	1.16364	1.16364
Mtpn	Mtpn-002	0.00981465	4.1269	4.1269
Mtr	Mtr-201	0.0169206	1.33632	1.33632
Mtrr	Mtrr-201	0.0229635	1.43226	1.43226
Mttp	Mttp-001	0.0205521	1.76763	1.76763
Mtus1	Mtus1-002	0.0353961	5.27737	5.27737
Mtx1	Mtx1-015	0.0145125	0.563796	-1.77369
Mtx2	Mtx2-002	0.0124817	1.37463	1.37463
Mutyh	Mutyh-005	0.0314066	0.0421926	-23.7009
Mvb12a	Mvb12a-201	0.0138464	0.780288	-1.28158
Mvb12b	Mvb12b-001	0.013998	0.760403	-1.31509
Mvb12b	Mvb12b-003	0.000476158	1.84939	1.84939
Mvp	Mvp-001	0.0100947	0.340595	-2.93604
Mvp	Mvp-003	0.0203586	0.595527	-1.67918
Mxra7	Mxra7-002	0.00315218	0.433477	-2.30693
Mxra7	Mxra7-001	1.00057e-05	0.746809	-1.33903
Mxra8	Mxra8-003	0.0482636	0.589606	-1.69605
Myadm	Myadm-002	0.0052846	0.46601	-2.14588
Myadm	Myadm-003	0.00907275	0.588776	-1.69844
Mybbp1a	Mybbp1a-007	0.00328929	2.22761	2.22761

Mybl1	Mybl1-001	0.0255179	0.852042	-1.17365
Mybpc2	Mybpc2-001	0.0032647	5.50303	5.50303
Myc	Myc-006	0.0288222	1.35737	1.35737
Mycbp	Mycbp-001	0.0410462	0.738451	-1.35419
Mycbp2	Mycbp2-003	0.0335332	0.712714	-1.40309
Mycn	Mycn-002	0.000720226	0.544611	-1.83617
Myct1	Myct1-201	0.0298669	0.412424	-2.42469
Myef2	Myef2-003	0.00207813	0.530229	-1.88598
Myg1	Myg1-007	0.0303983	1.29854	1.29854
Myh9	Myh9-004	0.0423094	0.333531	-2.99823
Myh9	Myh9-001	0.000737377	0.763912	-1.30905
Myh9	Myh9-002	7.58963e-05	2.60356	2.60356
Myl12a	Myl12a-002	0.0393155	0.434046	-2.3039
Myl12a	Myl12a-004	0.0187706	0.588056	-1.70052
Myl6	Myl6-201	0.00563558	0.752369	-1.32913
Mylpf	Mylpf-006	0.0275515	0.544446	-1.83673
Mynn	Mynn-004	0.0248655	1.22743	1.22743
Myo10	Myo10-001	0.0288864	0.743463	-1.34506
Myo18a	Myo18a-002	0.00256547	0.333997	-2.99404
Myo18a	Myo18a-201	0.0125794	0.423817	-2.35951
Myo18a	Myo18a-006	0.0140849	0.459609	-2.17576
Myo18a	Myo18a-202	0.0222074	0.480577	-2.08083
Myo18a	Myo18a-001	0.0499603	0.620782	-1.61087
Myo1a	Myo1a-201	0.0120461	0.72153	-1.38594
Myo1b	Myo1b-012	0.00368758	0.505972	-1.9764
Myo1c	Myo1c-009	0.0408767	0.0115342	-86.6987
Myo1h	Myo1h-004	0.0416875	5053.93	5053.93
Myo5a	Myo5a-001	0.00497981	0.599251	-1.66875
Myo5a	Myo5a-009	0.00450118	1.24862	1.24862
Myo5a	Myo5a-003	0.00247121	2.14714	2.14714
Myo5a	Myo5a-010	0.028428	9.90496	9.90496
Myo5c	Myo5c-201	0.0037057	1.58905	1.58905
Myo7a	Myo7a-005	0.018175	0.557513	-1.79368
Myo9a	Myo9a-001	0.0439581	0.646478	-1.54684
Myo9b	Myo9b-201	0.0406551	0.775426	-1.28961
Mypopos	Mypopos-002	0.0467217	1.18129	1.18129
Mysm1	Mysm1-003	0.0228571	0.24204	-4.13155
Mysm1	Mysm1-001	0.00438169	1.18491	1.18491
Mysm1	Mysm1-005	0.00097804	2.67764	2.67764
Mysm1	Mysm1-004	0.0454621	11.6535	11.6535
Myt1	Myt1-003	0.0386511	1.62932	1.62932
Myt1	Myt1-002	0.0042052	1.96395	1.96395
Myt1l	Myt1l-201	0.0318311	0.841339	-1.18858

Mzt1	Mzt1-201	0.00467942	1.09807	1.09807
Mzt2	Mzt2-002	0.020867	1.50764	1.50764
N4bp2l2	N4bp2l2-001	0.000423325	0.799563	-1.25068
N6amt1	N6amt1-001	0.00218704	1.63384	1.63384
Naa15	Naa15-201	0.0271217	1.32408	1.32408
Naa15	Naa15-004	4.21712e-05	2.81685	2.81685
Naa15	Naa15-008	0.031011	2.83606	2.83606
Naa15	Naa15-007	0.0041391	5.40607	5.40607
Naa16	Naa16-001	0.00415292	1.53268	1.53268
Naa20	Naa20-006	0.0036932	2.22838	2.22838
Naa25	Naa25-003	0.00633947	1.64072	1.64072
Naa25	Naa25-006	0.000246397	2.16203	2.16203
Naa25	Naa25-004	0.00405203	8.37172	8.37172
Naa35	Naa35-015	0.0391561	10.9189	10.9189
Naa50	Naa50-001	0.0140874	1.21481	1.21481
Naa50	Naa50-004	0.0249285	1.79572	1.79572
Naa60	Naa60-010	0.00988891	1.46811	1.46811
Naalad2	Naalad2-001	0.00911004	2.82912	2.82912
Nab1	Nab1-002	0.00117756	2.2919	2.2919
Nabp1	Nabp1-003	0.0448585	0.384197	-2.60283
Nabp1	Nabp1-004	0.0269884	0.411124	-2.43236
Nabp1	Nabp1-001	0.0199694	0.783523	-1.27629
Nabp1	Nabp1-008	0.00400212	7.22706	7.22706
Nabp2	Nabp2-009	0.0277981	1.93757	1.93757
Nacad	Nacad-001	0.0165316	0.795581	-1.25694
Nadsyn1	Nadsyn1-006	0.0389975	2.13386	2.13386
Nagk	Nagk-007	0.042723	0.619648	-1.61382
Naip5	Naip5-001	0.0175449	0.707926	-1.41258
Naip6	Naip6-201	0.018365	0.539145	-1.85479
Nanog	Nanog-002	0.015915	1.47907	1.47907
Nanog	Nanog-003	0.0159162	1.47908	1.47908
Nanos1	Nanos1-201	0.03071	0.811509	-1.23227
Nanp	Nanp-001	0.0331472	1.54874	1.54874
Nap1l1	Nap1l1-202	0.00015473	0.559272	-1.78804
Napa	Napa-007	0.00553432	1.67584	1.67584
Napepld	Napepld-002	0.0190099	1.46891	1.46891
Napg	Napg-001	0.00209089	0.837949	-1.19339
Napg	Napg-004	0.0245201	2.19716	2.19716
Nars	Nars-201	1.54893e-05	1.50179	1.50179
Nat10	Nat10-013	0.0190105	3.77468	3.77468
Nat9	Nat9-001	0.031769	1.32299	1.32299
Nat9	Nat9-002	0.00803034	2.33079	2.33079
Nat9	Nat9-006	0.0487558	2.7905	2.7905

Nat9	Nat9-005	0.0324481	3.00582	3.00582
Natd1	Natd1-001	0.0238103	1.35074	1.35074
Nav1	Nav1-201	0.0143162	0.300215	-3.33095
Nav1	Nav1-001	0.010808	0.642974	-1.55527
Nav1	Nav1-002	0.0263792	0.823409	-1.21446
Nbas	Nbas-201	0.000165043	1.25409	1.25409
Nbea	Nbea-006	0.0394535	5.98803	5.98803
Ncam1	Ncam1-006	0.0355884	0.78043	-1.28134
Ncam1	Ncam1-002	0.0150626	1.82475	1.82475
Ncam1	Ncam1-003	0.0115551	1.96692	1.96692
Ncapd2	Ncapd2-007	0.0257886	4.76206	4.76206
Ncapd3	Ncapd3-201	0.000218581	1.13705	1.13705
Ncapg	Ncapg-002	1.80836e-05	1.28724	1.28724
Ncbp1	Ncbp1-001	0.010793	1.38408	1.38408
Nck1	Nck1-006	0.00954914	1.28871	1.28871
Nck2	Nck2-001	0.00381029	0.778284	-1.28488
Nckap1	Nckap1-003	0.0427472	0.639605	-1.56346
Ncoa1	Ncoa1-201	0.00454569	1.1669	1.1669
Ncoa3	Ncoa3-201	0.00536866	0.724998	-1.37931
Ncoa3	Ncoa3-001	4.6621e-05	1.78499	1.78499
Ncoa4	Ncoa4-001	0.0293897	0.511446	-1.95524
Ncoa5	Ncoa5-004	0.0474219	2.59031	2.59031
Ncoa6	Ncoa6-201	0.0012121	1.12113	1.12113
Ncoa6	Ncoa6-002	0.0372528	2.93599	2.93599
Ncoa7	Ncoa7-201	0.0208292	0.845432	-1.18283
Ncor1	Ncor1-004	0.0329126	1.35252	1.35252
Ncor1	Ncor1-008	0.0286489	3.59712	3.59712
Ncor2	Ncor2-001	0.0427306	0.797124	-1.25451
Ncstn	Ncstn-001	0.0225656	0.857595	-1.16605
Ncstn	Ncstn-005	0.00335628	2.10911	2.10911
Ndc1	Ndc1-003	0.00102054	1.93812	1.93812
Ndc80	Ndc80-201	0.0141432	1.28741	1.28741
Ndfip2	Ndfip2-003	0.0330571	1.16249	1.16249
Ndrg1	Ndrg1-002	0.0494825	0.636044	-1.57222
Ndrg3	Ndrg3-002	0.0205375	1.40977	1.40977
Ndrg3	Ndrg3-001	0.0240714	1.54143	1.54143
Ndst2	Ndst2-201	0.0394975	1.38757	1.38757
Ndst4	Ndst4-005	0.0216644	0.464318	-2.1537
Ndufa7	Ndufa7-006	0.0240069	1.40763	1.40763
Ndufa8	Ndufa8-001	0.0117904	1.2937	1.2937
Ndufa9	Ndufa9-003	0.0227235	1.21567	1.21567
Ndufab1	Ndufab1-006	0.0312756	1.40791	1.40791
Ndufaf3	Ndufaf3-002	0.0377453	1.34939	1.34939

Ndufaf3	Ndufaf3-001	0.0203916	1.39544	1.39544
Ndufaf5	Ndufaf5-001	0.00395934	1.42269	1.42269
Ndufaf7	Ndufaf7-201	8.02324e-05	1.79108	1.79108
Ndufb11	Ndufb11-001	0.0116746	0.810789	-1.23337
Ndufb3	Ndufb3-001	0.00738901	1.21998	1.21998
Ndufb5	Ndufb5-001	0.00521786	0.585598	-1.70766
Ndufb5	Ndufb5-007	0.0159055	0.657296	-1.52138
Ndufb8	Ndufb8-001	0.0175582	1.17824	1.17824
Ndufc1	Ndufc1-004	0.0323133	1.07321	1.07321
Ndufs1	Ndufs1-008	0.0263897	0.0568977	-17.5754
Ndufs5	Ndufs5-001	0.023957	1.12743	1.12743
Ndufs6	Ndufs6-002	0.00175367	2.47784	2.47784
Ndufv1	Ndufv1-005	0.0223791	1.60743	1.60743
Ndufv2	Ndufv2-002	0.00666164	1.33294	1.33294
Ndufv2	Ndufv2-001	0.0284555	1.58162	1.58162
Neat1	Neat1-001	0.0173232	1.3445	1.3445
Nebl	Nebl-001	0.00152782	0.678728	-1.47335
Necap2	Necap2-009	0.0167875	1.90302	1.90302
Nedd1	Nedd1-201	0.00625164	1.3025	1.3025
Nedd4	Nedd4-006	0.00324579	21.0126	21.0126
Neil3	Neil3-001	0.0494009	0.737497	-1.35594
Nek2	Nek2-001	0.0271235	1.28866	1.28866
Nek2	Nek2-002	0.0498266	1.47607	1.47607
Nek4	Nek4-201	0.00476385	1.35202	1.35202
Nek6	Nek6-002	0.029463	1.79774	1.79774
Nek6	Nek6-003	0.00763365	1.99661	1.99661
Nelfa	Nelfa-001	0.0199113	1.2963	1.2963
Nelfb	Nelfb-004	0.0110703	0.323215	-3.09391
Nes	Nes-003	0.0284311	0.499532	-2.00188
Nes	Nes-001	0.0205098	0.581009	-1.72114
Neu1	Neu1-001	0.0414708	1.30235	1.30235
Neu3	Neu3-201	0.00869943	1.15233	1.15233
Neurl1a	Neurl1a-201	0.00906927	0.577165	-1.73261
Neurl2	Neurl2-001	0.000215988	0.623004	-1.60513
Neurl3	Neurl3-001	0.00049715	0.429744	-2.32696
Neurl4	Neurl4-003	0.046607	0.312631	-3.19866
Nf1	Nf1-007	0.049752	1.80247	1.80247
Nf1	Nf1-003	0.0345326	2.05649	2.05649
Nfat5	Nfat5-010	0.00108177	3.34908	3.34908
Nfat5	Nfat5-005	0.00889782	4.55765	4.55765
Nfat5	Nfat5-004	0.0170482	5.83131	5.83131
Nfe2l1	Nfe2l1-204	0.0190967	1.53328	1.53328
Nfib	Nfib-202	0.00693022	0.674849	-1.48181

Nfib	Nfib-201	0.0288047	0.684001	-1.46199
Nfib	Nfib-002	0.0404338	6.40935	6.40935
Nfic	Nfic-001	0.00361156	0.831677	-1.20239
Nfil3	Nfil3-001	0.0244756	1.55611	1.55611
Nfix	Nfix-001	0.0306987	1.31432	1.31432
Nfkb2	Nfkb2-201	0.013095	0.388777	-2.57217
Nfkbia	Nfkbia-201	0.00568179	0.436791	-2.28942
Nfkbie	Nfkbie-201	0.0154487	0.672753	-1.48643
Nfkbiz	Nfkbiz-004	0.0184237	0.651766	-1.53429
Nfu1	Nfu1-001	0.00045526	1.4843	1.4843
Nfu1	Nfu1-005	0.0456111	1.69949	1.69949
Nfx1	Nfx1-002	0.0344718	0.725921	-1.37756
Nfx1	Nfx1-007	0.000892261	1.39772	1.39772
Nfxl1	Nfxl1-001	0.0191409	1.3506	1.3506
Nfya	Nfya-001	0.0251167	0.799659	-1.25053
Ngef	Ngef-001	0.0410991	17.2232	17.2232
Ngfr	Ngfr-001	0.04854	0.732397	-1.36538
Ngfrap1	Ngfrap1-002	0.0029976	1.31946	1.31946
Ngfrap1	Ngfrap1-003	9.30037e-05	1.45222	1.45222
Ngly1	Ngly1-001	0.000217072	1.41501	1.41501
Ngrn	Ngrn-001	0.00223051	1.83463	1.83463
Nhlh2	Nhlh2-002	0.0464455	3.11942	3.11942
Nhlh2	Nhlh2-001	0.0464456	3.11942	3.11942
Nhlrc2	Nhlrc2-201	0.0493591	1.22569	1.22569
Nin	Nin-001	0.010468	0.679794	-1.47103
Nipa2	Nipa2-011	0.0428487	0.519527	-1.92483
Nipsnap1	Nipsnap1-004	0.0267684	188.328	188.328
Nit1	Nit1-002	0.00361478	1.28273	1.28273
Nit2	Nit2-201	0.00525004	1.27347	1.27347
Nkain1	Nkain1-001	0.0469726	0.692808	-1.4434
Nkap	Nkap-001	0.0188793	0.875115	-1.14271
Nkiras1	Nkiras1-002	0.0394659	1.30157	1.30157
Nkiras2	Nkiras2-001	0.0397551	1.17921	1.17921
Nle1	Nle1-001	0.0165148	1.57154	1.57154
Nlgn1	Nlgn1-202	0.0164364	0.42466	-2.35483
Nlgn2	Nlgn2-001	0.0142496	0.671286	-1.48968
Nlk	Nlk-002	0.00677885	1.91116	1.91116
Nlrp4f	Nlrp4f-201	0.0209886	0.725124	-1.37908
Nlrp9b	Nlrp9b-001	0.00273909	0.569399	-1.75624
Nme6	Nme6-011	0.0313	0.000708901	-1410.63
Nmnat1	Nmnat1-001	0.0280543	1.74442	1.74442
Nmnat2	Nmnat2-001	0.0123238	0.681804	-1.4667
Nmnat2	Nmnat2-006	0.0298893	1.41549	1.41549

Nmnat2	Nmnat2-004	0.00642432	4.32804	4.32804
Nmral1	Nmral1-006	0.0156207	2.00649	2.00649
Nmrk1	Nmrk1-004	0.0478341	0.472495	-2.11642
Nmrk1	Nmrk1-001	0.00448615	1.24872	1.24872
Nmt1	Nmt1-003	0.0023795	1.43867	1.43867
Nmt2	Nmt2-001	0.00158587	0.846852	-1.18084
Nmt2	Nmt2-003	0.014978	1.66974	1.66974
Nmt2	Nmt2-002	0.000125192	2.01168	2.01168
Nob1	Nob1-001	0.00431675	1.40529	1.40529
Noc2l	Noc2l-201	0.048097	1.39647	1.39647
Noc2l	Noc2l-202	0.00162733	1.94089	1.94089
Nod1	Nod1-002	0.00436127	0.516089	-1.93765
Nol11	Nol11-201	0.0475239	1.55652	1.55652
Nol11	Nol11-002	0.0147016	1.80208	1.80208
Nol4l	Nol4l-002	0.0309816	1.32726	1.32726
Nol7	Nol7-201	0.00108545	1.17562	1.17562
Nono	Nono-007	0.000412296	1.54631	1.54631
Nop14	Nop14-001	0.00695887	1.41845	1.41845
Nop56	Nop56-014	0.0297054	0.407274	-2.45535
Nop58	Nop58-005	0.0182307	2.44857	2.44857
Nos1	Nos1-001	0.00288443	0.24649	-4.05697
Nosip	Nosip-202	0.000530095	5.25116	5.25116
Notch2	Notch2-001	0.00196729	0.709646	-1.40915
Notum	Notum-004	0.015881	4.59829	4.59829
Nova1	Nova1-201	0.0109586	0.730364	-1.36918
Nova2	Nova2-001	0.0152814	0.898177	-1.11337
Nox1	Nox1-001	0.0195127	4.97715	4.97715
Npas3	Npas3-201	0.00511322	1.89518	1.89518
Npb	Npb-001	0.00510442	154428	154428
Npc1	Npc1-001	0.00284871	1.23225	1.23225
Npc1l1	Npc1l1-001	0.00203096	2.07332	2.07332
Npepps	Npepps-007	0.00416303	0.200789	-4.98036
Npff	Npff-001	0.0403418	0.0148852	-67.1809
Npm1	Npm1-005	0.0210084	0.621232	-1.60971
Npm1	Npm1-002	0.00547174	1.24829	1.24829
Nppb	Nppb-001	0.00483572	0.166692	-5.9991
Npr1	Npr1-005	0.000252636	1.56778	1.56778
Nptn	Nptn-006	0.0058963	0.181706	-5.5034
Npy	Npy-001	0.00451348	0.558531	-1.79041
Nqo1	Nqo1-001	0.044244	1.27423	1.27423
Nr1d2	Nr1d2-001	0.00270238	0.864486	-1.15676
Nr1h2	Nr1h2-008	0.025268	2.13977	2.13977
Nr2c1	Nr2c1-002	0.00643295	1.69065	1.69065

Nr2c2	Nr2c2-001	0.0133218	0.74932	-1.33454
Nr2c2	Nr2c2-006	0.0244128	1.98504	1.98504
Nr4a2	Nr4a2-006	0.0132447	1.83556	1.83556
Nr6a1	Nr6a1-202	0.0226709	0.255749	-3.91008
Nr6a1	Nr6a1-001	0.0472226	1.89304	1.89304
Nradd	Nradd-001	0.0483035	0.773256	-1.29323
Nras	Nras-001	1.33259e-05	0.544272	-1.83732
Nrbp2	Nrbp2-201	0.0188589	1.46573	1.46573
Nrde2	Nrde2-201	0.00185516	1.19643	1.19643
Nrep	Nrep-202	0.0338124	0.302644	-3.30421
Nrep	Nrep-203	0.0124516	0.42595	-2.3477
Nrip2	Nrip2-003	0.000950956	3.33571	3.33571
Nrm	Nrm-001	0.00182134	0.669215	-1.49429
Nrn1	Nrn1-001	0.040824	0.854816	-1.16984
Nrn1l	Nrn1l-002	0.0267709	0.226862	-4.40796
Nrsn1	Nrsn1-001	0.0440814	1.20207	1.20207
Nrxn1	Nrxn1-017	0.0481958	0.0192845	-51.8551
Nrxn2	Nrxn2-004	0.0329117	0.873804	-1.14442
Nrxn2	Nrxn2-006	0.00499952	1.22605	1.22605
Nrxn2	Nrxn2-012	0.0418195	2.76896	2.76896
Nsd1	Nsd1-201	0.0425715	1.45382	1.45382
Nsf	Nsf-001	0.0126967	1.52957	1.52957
Nsfl1c	Nsfl1c-002	0.00486942	1.33832	1.33832
Nsg2	Nsg2-001	0.001751	1.51712	1.51712
Nsl1	Nsl1-001	0.0119164	1.21149	1.21149
Nsmaf	Nsmaf-007	0.0439491	965.543	965.543
Nt5c3	Nt5c3-001	0.0216313	1.309	1.309
Nt5dc1	Nt5dc1-001	0.0439528	1.60122	1.60122
Ntan1	Ntan1-008	0.00549014	1.84506	1.84506
Ntn1	Ntn1-201	0.0469925	0.482453	-2.07274
Ntn1	Ntn1-001	0.0469929	0.482453	-2.07274
Ntpcr	Ntpcr-001	0.00101703	1.3574	1.3574
Ntrk3	Ntrk3-002	0.0144368	0.869472	-1.15012
Nuak1	Nuak1-001	0.0323902	0.662996	-1.5083
Nucb1	Nucb1-005	0.0016843	0.620041	-1.6128
Nucb1	Nucb1-001	0.0201208	0.831338	-1.20288
Nucb2	Nucb2-001	0.000437065	1.35502	1.35502
Nucks1	Nucks1-001	0.00672947	1.26386	1.26386
Nudt17	Nudt17-002	0.0263737	2.61758	2.61758
Nudt21	Nudt21-201	0.0474579	0.821896	-1.2167
Nudt3	Nudt3-003	0.0314602	2.19806	2.19806
Nudt5	Nudt5-003	0.032559	1.6491	1.6491
Nudt5	Nudt5-201	0.00443908	1.67341	1.67341

Nudt9	Nudt9-001	0.00219747	1.40406	1.40406
Nudt9	Nudt9-003	0.0409619	2.33491	2.33491
Nufip1	Nufip1-201	0.00345686	1.17176	1.17176
Nufip2	Nufip2-001	0.0170839	1.12874	1.12874
Numa1	Numa1-001	0.00122914	0.861896	-1.16023
Nup160	Nup160-003	0.0465897	2.61738	2.61738
Nup205	Nup205-006	0.0482284	0.745666	-1.34108
Nup210	Nup210-002	0.0202366	0.803562	-1.24446
Nup54	Nup54-001	0.00440585	1.19208	1.19208
Nup88	Nup88-004	0.0235378	2.14242	2.14242
Nupl2	Nupl2-002	0.0400211	2.31007	2.31007
Nupr1	Nupr1-001	0.00149218	2.42684	2.42684
Nupr1	Nupr1-005	0.01212	2.8272	2.8272
Nus1	Nus1-001	0.0229143	1.1611	1.1611
Nus1	Nus1-002	0.00271096	3.24923	3.24923
Nusap1	Nusap1-001	0.00690681	1.1681	1.1681
Nwd2	Nwd2-002	0.0177741	18.1051	18.1051
Nxf1	Nxf1-001	0.021515	1.19069	1.19069
Nynrin	Nynrin-202	0.0370189	0.663887	-1.50628
Oasl2	Oasl2-007	0.0102368	0.653705	-1.52974
Obfc1	Obfc1-001	0.0411127	1.3837	1.3837
Obscn	Obscn-005	0.0407536	160.689	160.689
Obsl1	Obsl1-002	0.02653	2.43711	2.43711
Ocrl	Ocrl-004	0.0153218	2.05708	2.05708
Odc1	Odc1-201	0.0463379	0.708102	-1.41223
Odf2	Odf2-017	0.0436498	0.265182	-3.771
Odf2	Odf2-002	0.0326939	0.70585	-1.41673
Odf2l	Odf2l-014	0.0414148	1.98967	1.98967
Odf3	Odf3-002	0.00025821	6.99511e-09	-1.42957e+08
Odf3b	Odf3b-201	0.0359741	5.16616	5.16616
Ogdhl	Ogdhl-201	8.41212e-05	0.768504	-1.30123
Ogfod1	Ogfod1-001	0.0442901	1.43353	1.43353
Ogfod1	Ogfod1-003	0.0261435	2.64705	2.64705
Ogfod3	Ogfod3-001	0.00325805	0.750283	-1.33283
Ogg1	Ogg1-008	0.00616193	0.187133	-5.34378
Ola1	Ola1-004	0.0178919	1.17766	1.17766
Olfm1	Olfm1-001	0.0258369	0.569389	-1.75627
Olfml2a	Olfml2a-001	0.0074544	0.661414	-1.51191
Olfml2b	Olfml2b-001	0.00628675	0.674418	-1.48276
Olfr1372-ps1	Olfr1372-ps1-002	0.0222093	2.29374	2.29374
Olfr222	Olfr222-001	0.0193535	1.75327	1.75327
Olfr368	Olfr368-001	0.00261213	1.54014	1.54014
Olfr69	Olfr69-001	0.000767801	2.05454	2.05454

Opa1	Opa1-003	0.00170645	1.96321	1.96321
Opa1	Opa1-002	0.0490079	3.60008	3.60008
Opa3	Opa3-002	0.00265476	0.365807	-2.73368
Optn	Optn-001	0.0235422	0.679156	-1.47242
Orai3	Orai3-001	0.00222679	0.623546	-1.60373
Orc3	Orc3-010	0.0210854	2.41471	2.41471
Orc4	Orc4-004	0.00545657	1.36058	1.36058
Ormdl3	Ormdl3-001	0.0121114	1.35409	1.35409
Osbp	Osbp-201	0.000182979	1.23111	1.23111
Osbpl5	Osbpl5-001	0.00670547	0.725209	-1.37891
Osbpl6	Osbpl6-006	0.0152168	3.21904	3.21904
Osbpl8	Osbpl8-202	0.000312563	0.79691	-1.25485
Oser1	Oser1-001	0.00900461	1.17048	1.17048
Osgep	Osgep-007	0.0126859	0.465983	-2.146
Osgep	Osgep-008	0.0300537	0.766986	-1.3038
Ost4	Ost4-001	0.0489087	1.21748	1.21748
Ostc	Ostc-001	0.000350606	1.25979	1.25979
Otop2	Otop2-001	0.0366753	2.2042	2.2042
Otub2	Otub2-001	0.00382054	1.82286	1.82286
Otub2	Otub2-003	0.00936506	2.06979	2.06979
Otub2	Otub2-201	0.0367092	2.44049	2.44049
Otud1	Otud1-001	0.00367776	4.74739	4.74739
Otud3	Otud3-001	0.00918621	1.24275	1.24275
Otud7b	Otud7b-001	0.00727639	0.494454	-2.02243
Otud7b	Otud7b-201	0.0120505	0.577312	-1.73217
Otud7b	Otud7b-006	0.0330062	24.0701	24.0701
Otx1	Otx1-001	0.019923	1.35149	1.35149
Oxct1	Oxct1-001	0.00303302	0.605383	-1.65185
Oxct1	Oxct1-002	0.00634886	2.16844	2.16844
Oxld1	Oxld1-001	0.0101599	1.6129	1.6129
Oxnad1	Oxnad1-005	0.00600104	3.80884	3.80884
Oxsr1	Oxsr1-001	0.0111724	0.808494	-1.23687
Oxsr1	Oxsr1-002	1.28295e-05	10.5656	10.5656
P2rx1	P2rx1-001	0.00481235	0.0747174	-13.3838
P2rx2	P2rx2-001	0.0481253	4.79947	4.79947
P2rx3	P2rx3-002	0.0352138	2.04796	2.04796
P2rx3	P2rx3-005	0.00107198	3.13885	3.13885
P2rx4	P2rx4-002	0.0410048	1.2664	1.2664
P2rx7	P2rx7-001	0.00115165	0.635911	-1.57255
P2ry2	P2ry2-201	0.028811	0.548307	-1.8238
P2ry6	P2ry6-201	0.000826814	0.528796	-1.89109
P3h1	P3h1-003	0.0209171	0.28248	-3.54007
P3h1	P3h1-008	0.0302853	3.40094	3.40094

P3h4	P3h4-002	0.017909	0.363607	-2.75022
P3h4	P3h4-001	0.0143611	0.772756	-1.29407
P4ha2	P4ha2-016	0.0383926	1.34428	1.34428
P4ha2	P4ha2-001	0.0417562	1.52848	1.52848
P4ha2	P4ha2-002	0.00527029	1.62966	1.62966
P4ha2	P4ha2-005	0.0146008	2.03766	2.03766
P4ha3	P4ha3-001	0.0149423	2.96699e-05	-33704.2
P4hb	P4hb-001	0.0474816	1.06033	1.06033
P4htm	P4htm-001	0.0205181	1.80258	1.80258
Pa2g4	Pa2g4-002	0.0230213	1.4738	1.4738
Pabpc1	Pabpc1-005	0.0145012	0.481272	-2.07783
Pabpc1	Pabpc1-006	0.0375757	0.763093	-1.31046
Pabpc1	Pabpc1-001	0.0190633	0.803082	-1.2452
Pabpc1l	Pabpc1l-005	0.002993	0.000888438	-1125.57
Pabpc4	Pabpc4-006	0.0490924	0.578934	-1.72731
Pacrgl	Pacrgl-009	0.0297802	11.4506	11.4506
Pacsin1	Pacsin1-004	0.0128547	2.82112	2.82112
Pacsin2	Pacsin2-203	0.0259574	0.727799	-1.37401
Pacsin2	Pacsin2-202	0.0420887	1.40121	1.40121
Pacsin3	Pacsin3-010	0.0465454	0.224116	-4.46198
Padi2	Padi2-001	0.0163508	0.695933	-1.43692
Padi3	Padi3-001	0.0125061	0.623844	-1.60296
Paf1	Paf1-008	0.0288187	0.424984	-2.35303
Pafah1b1	Pafah1b1-004	0.0396603	0.617111	-1.62045
Pafah1b1	Pafah1b1-001	0.00219664	1.65471	1.65471
Paics	Paics-002	0.00754507	0.818231	-1.22215
Paip2b	Paip2b-003	0.00943012	3.12555	3.12555
Pak4	Pak4-003	0.0152886	2.33696	2.33696
Palb2	Palb2-008	0.0210624	0.189063	-5.28924
Palb2	Palb2-005	0.0110836	0.369368	-2.70732
Palb2	Palb2-001	0.0294029	1.52463	1.52463
Palld	Palld-003	0.00689909	2.72418	2.72418
Palld	Palld-004	0.0422929	3.08155	3.08155
Pank1	Pank1-202	0.0309681	1.52886	1.52886
Pank3	Pank3-001	0.00195236	0.762282	-1.31185
Pank4	Pank4-002	0.0215832	0.431125	-2.31951
Papd5	Papd5-201	0.0458545	0.723023	-1.38308
Papln	Papln-001	0.00872442	0.284437	-3.51571
Papln	Papln-002	0.00741079	5.91052	5.91052
Papola	Papola-004	0.0402449	0.775123	-1.29012
Papola	Papola-001	0.0165303	0.915782	-1.09196
Papola	Papola-010	0.0315153	1.43624	1.43624
Papola	Papola-009	0.000921819	3.5859	3.5859

Papolg	Papolg-001	0.0145285	1.23669	1.23669
Papolg	Papolg-002	0.0212124	4.6129	4.6129
Papss2	Papss2-201	0.0454234	1.13334	1.13334
Paqr3	Paqr3-004	0.0177232	1.95154	1.95154
Pard3	Pard3-002	0.00876724	0.694398	-1.4401
Pard3	Pard3-006	0.0448248	2.11743	2.11743
Pard3	Pard3-019	0.00126886	2.23878	2.23878
Pard6a	Pard6a-202	0.00737244	1.34573	1.34573
Park7	Park7-007	0.0224108	1.75458	1.75458
Park7	Park7-009	0.0385933	2.02451	2.02451
Parp11	Parp11-001	0.0416189	1.50977	1.50977
Parp16	Parp16-201	0.00705702	1.28334	1.28334
Parp3	Parp3-004	0.000278944	0.373528	-2.67718
Parp4	Parp4-001	0.0332616	1.1487	1.1487
Parp8	Parp8-201	0.0246018	0.87413	-1.144
Parp9	Parp9-001	0.00090959	1.88101	1.88101
Parva	Parva-001	0.0436538	0.700127	-1.42831
Patl1	Patl1-201	0.00197269	1.47325	1.47325
Pax6	Pax6-024	0.0298507	5.38071	5.38071
Paxip1	Paxip1-005	0.00102985	0.635913	-1.57254
Paxip1	Paxip1-006	0.00607776	2.95556	2.95556
Pbld2	Pbld2-001	0.000446169	2.40706	2.40706
Pbld2	Pbld2-002	0.0172194	7.07762	7.07762
Pbx1	Pbx1-002	0.0115067	0.818781	-1.22133
Pbx2	Pbx2-001	0.0260197	0.761117	-1.31386
Pbx3	Pbx3-003	0.0358834	9.42196	9.42196
Pbxip1	Pbxip1-001	0.00130316	0.72234	-1.38439
Pcbp2	Pcbp2-203	0.00219268	1.20398	1.20398
Pcbp3	Pcbp3-008	0.0349113	0.475221	-2.10429
Pcbp3	Pcbp3-005	0.0170376	2.00179	2.00179
Pcbp4	Pcbp4-001	0.00226333	0.77798	-1.28538
Pcdh1	Pcdh1-004	4.4003e-06	0.582714	-1.71611
Pcdh1	Pcdh1-002	0.000664631	0.640423	-1.56147
Pcdh1	Pcdh1-005	0.0464844	1.25493	1.25493
Pcdh12	Pcdh12-004	0.00715493	0.404271	-2.47359
Pcdh17	Pcdh17-201	0.0421855	1.26185	1.26185
Pcdha3	Pcdha3-001	0.000320238	2.1645	2.1645
Pcdha8	Pcdha8-001	0.0403806	0.648085	-1.54301
Pcdha9	Pcdha9-001	0.00064012	0.488112	-2.04871
Pcdhb1	Pcdhb1-001	0.000976554	2.54967	2.54967
Pcdhb10	Pcdhb10-001	0.00405937	1.67875	1.67875
Pcdhb11	Pcdhb11-001	0.0250712	1.31834	1.31834
Pcdhb17	Pcdhb17-001	0.0179121	1.29042	1.29042

Pcdhb2	Pcdhb2-001	0.0026687	1.79764	1.79764
Pcdhb20	Pcdhb20-001	0.00516441	1.21627	1.21627
Pcdhb4	Pcdhb4-001	0.000146543	0.662394	-1.50968
Pcdhb6	Pcdhb6-001	0.0101575	1.92064	1.92064
Pcdhb7	Pcdhb7-001	0.0239614	1.56029	1.56029
Pcdhb8	Pcdhb8-002	0.000148903	9.54693	9.54693
Pcdhb9	Pcdhb9-001	0.00862561	2.51375	2.51375
Pcdhgb1	Pcdhgb1-001	0.000762636	0.416949	-2.39837
Pcdhgb7	Pcdhgb7-001	0.0118584	0.242883	-4.11721
Pcdhgb8	Pcdhgb8-001	0.0228839	0.697828	-1.43302
Pcdhgc3	Pcdhgc3-003	0.0032665	0.39189	-2.55174
Pcdhgc3	Pcdhgc3-001	0.00137531	0.580067	-1.72394
Pcgf1	Pcgf1-007	0.0142983	2.67518	2.67518
Pcgf2	Pcgf2-002	0.00891178	1.26349	1.26349
Pcgf3	Pcgf3-001	0.00845781	0.695487	-1.43784
Pcid2	Pcid2-001	0.00298092	1.29797	1.29797
Pcif1	Pcif1-004	0.0371634	2.08657	2.08657
Pcmtd1	Pcmtd1-001	0.0436776	1.25074	1.25074
Pcnp	Pcnp-004	0.0333516	13.4245	13.4245
Pcnxl2	Pcnxl2-002	0.00153462	1.34373	1.34373
Pcnxl3	Pcnxl3-004	0.0274103	0.693002	-1.443
Pcp4	Pcp4-001	0.00107686	1.37762	1.37762
Pcx	Pcx-201	0.0039838	1.19392	1.19392
Pdcd10	Pdcd10-004	0.0310537	0.41447	-2.41272
Pdcd10	Pdcd10-005	0.0367851	28.368	28.368
Pdcd2	Pdcd2-001	0.0116489	1.24522	1.24522
Pdcd6ip	Pdcd6ip-001	0.0420691	0.815056	-1.22691
Pdcd6ip	Pdcd6ip-002	0.0498744	1.16565	1.16565
Pdcd7	Pdcd7-201	0.000529557	0.624424	-1.60148
Pde10a	Pde10a-001	0.0463478	0.660314	-1.51443
Pde10a	Pde10a-009	0.00500658	2.36332	2.36332
Pde4a	Pde4a-001	0.03679	0.746066	-1.34036
Pde4b	Pde4b-012	0.0391499	0.31143	-3.21099
Pde4b	Pde4b-003	0.00908964	0.522845	-1.91261
Pde4b	Pde4b-008	0.0399348	2.8886	2.8886
Pde4c	Pde4c-004	0.0223343	0.135887	-7.35904
Pde4dip	Pde4dip-005	0.000328895	1.76225	1.76225
Pde4dip	Pde4dip-004	0.000516831	2.68097	2.68097
Pde7a	Pde7a-009	0.0456013	0.51765	-1.93181
Pde7a	Pde7a-001	0.000233703	0.648354	-1.54237
Pdha1	Pdha1-002	0.0233745	2.90538	2.90538
Pdia5	Pdia5-201	0.0027034	1.43336	1.43336
Pdk1	Pdk1-001	0.000443397	0.732107	-1.36592

Pdlim7	Pdlim7-002	0.00460368	0.436104	-2.29303
Pdlim7	Pdlim7-005	0.000953372	2.26286	2.26286
Pdpk1	Pdpk1-007	0.0335248	2.10542	2.10542
Pdpk1	Pdpk1-002	0.000712129	3.07634	3.07634
Pdrg1	Pdrg1-001	0.0165149	1.50294	1.50294
Pdss2	Pdss2-002	0.011637	2.0095	2.0095
Pdxk	Pdxk-001	0.00397051	1.62177	1.62177
Pdzd11	Pdzd11-001	0.0368351	1.60812	1.60812
Pdzd2	Pdzd2-004	0.00455664	0.66853	-1.49582
Pea15a	Pea15a-001	0.000625734	0.727197	-1.37514
Peak1	Peak1-002	2.73272e-05	0.685378	-1.45905
Pebp1	Pebp1-001	0.00111761	1.17334	1.17334
Pecr	Pecr-001	0.0286682	1.21639	1.21639
Pef1	Pef1-201	0.0460451	1.1596	1.1596
Peg13	Peg13-001	0.0368113	0.856104	-1.16808
Peg3	Peg3-001	0.0040434	0.787758	-1.26943
Peli3	Peli3-001	0.0326413	88443.3	88443.3
Per2	Per2-001	0.000290483	1.47772	1.47772
Pes1	Pes1-002	0.0308406	1.48963	1.48963
Pex11a	Pex11a-001	0.00289812	0.159517	-6.26892
Pex11g	Pex11g-003	0.00860095	0.154307	-6.48059
Pex16	Pex16-001	0.043768	0.455291	-2.1964
Pex19	Pex19-004	0.0225484	0.409155	-2.44406
Pex19	Pex19-005	0.0349884	2.73189	2.73189
Pex2	Pex2-001	0.00316201	1.80149	1.80149
Pex5	Pex5-001	0.0151013	1.60274	1.60274
Pfdn1	Pfdn1-201	0.00020412	1.30898	1.30898
Pfdn2	Pfdn2-003	0.00156212	1.27076	1.27076
Pfdn2	Pfdn2-002	0.00450345	1.37861	1.37861
Pfdn4	Pfdn4-004	0.00393247	1.17952	1.17952
Pfkfb4	Pfkfb4-001	0.0438186	9.28245	9.28245
Pfkm	Pfkm-202	0.0106508	1.62449	1.62449
Pfn1	Pfn1-003	0.0172565	0.78992	-1.26595
Pfn2	Pfn2-002	0.028146	0.723578	-1.38202
Pgap1	Pgap1-004	0.00156797	13.9349	13.9349
Pgap2	Pgap2-002	0.0421863	1.425	1.425
Pgap2	Pgap2-020	0.00902197	7.63491	7.63491
Pgap2	Pgap2-011	0.0446193	9.64037	9.64037
Pgap3	Pgap3-001	0.0131427	1.85573	1.85573
Pgbd5	Pgbd5-005	0.0143071	2.72553	2.72553
Pgd	Pgd-003	0.0473549	0.813947	-1.22858
Pggt1b	Pggt1b-201	0.0465399	1.09476	1.09476
Pglyrp1	Pglyrp1-002	0.0018603	3.5802	3.5802

Pgrmc2	Pgrmc2-001	0.00217967	1.19716	1.19716
Phactr2	Phactr2-201	3.4709e-05	0.592983	-1.68639
Phactr4	Phactr4-001	0.00321378	0.62025	-1.61225
Phc1	Phc1-002	0.0297516	1.63832	1.63832
Phc2	Phc2-005	0.0134396	0.483697	-2.06741
Phc2	Phc2-004	0.0284028	0.778335	-1.28479
Phc3	Phc3-001	0.00110267	0.819155	-1.22077
Phc3	Phc3-201	0.00985163	8.92674	8.92674
Phf10	Phf10-001	0.000112515	1.46701	1.46701
Phf10	Phf10-002	0.00883908	1.60452	1.60452
Phf10	Phf10-004	0.0456941	2.81336	2.81336
Phf13	Phf13-001	0.000142673	0.825993	-1.21066
Phf14	Phf14-009	0.0127885	2.26924	2.26924
Phf19	Phf19-005	0.0497873	4.01196	4.01196
Phf21a	Phf21a-201	0.00592145	0.518321	-1.92931
Phf21a	Phf21a-202	0.0248064	0.556818	-1.79592
Phf21b	Phf21b-003	0.00127673	2.49047	2.49047
Phf23	Phf23-009	0.0132426	1.49614	1.49614
Phf8	Phf8-001	0.0474733	1.22904	1.22904
Phgdh	Phgdh-001	0.000610899	1.35128	1.35128
Phip	Phip-006	0.00961591	0.173067	-5.77811
Phkg1	Phkg1-006	0.0378772	1.73586	1.73586
Phkg1	Phkg1-004	0.0444635	1.78982	1.78982
Phkg2	Phkg2-003	0.00127668	1.72648	1.72648
Phldb1	Phldb1-015	0.0419016	0.624003	-1.60256
Phospho1	Phospho1-001	0.0346727	1.79046	1.79046
Phpt1	Phpt1-002	0.0455555	0.724756	-1.37977
Phyh	Phyh-001	0.00200711	0.652235	-1.53319
Phyhd1	Phyhd1-011	0.022362	0.6256	-1.59847
Pi16	Pi16-002	0.00810992	0.676948	-1.47722
Pi4k2b	Pi4k2b-002	0.044859	0.489545	-2.04271
Pi4k2b	Pi4k2b-001	0.0414991	0.719363	-1.39012
Pianp	Pianp-001	0.00678699	0.380894	-2.6254
Pias4	Pias4-004	0.046931	0.553683	-1.80609
Pick1	Pick1-004	0.00519369	4.72623	4.72623
Pidd1	Pidd1-201	0.0164454	0.112673	-8.87523
Piezo1	Piezo1-003	0.00253761	0.420732	-2.37681
Piezo1	Piezo1-001	0.00124029	0.632038	-1.58218
Piezo1	Piezo1-201	0.0326742	0.692889	-1.44323
Pigb	Pigb-201	0.0299068	0.609987	-1.63938
Pigb	Pigb-005	0.00441464	3.99808	3.99808
Pigf	Pigf-001	0.0294764	1.2088	1.2088
Pigg	Pigg-002	0.0274334	1.80775	1.80775

Pigh	Pigh-201	0.000417169	2.10963	2.10963
Pigk	Pigk-002	0.00985459	1.67108	1.67108
Pigl	Pigl-001	0.0189396	1.27949	1.27949
Pign	Pign-002	0.000378498	4.60505	4.60505
Pigp	Pigp-002	0.0253505	0.307496	-3.25208
Pigp	Pigp-006	0.000170379	5.42017	5.42017
Pigq	Pigq-001	0.00179522	0.625332	-1.59915
Pigq	Pigq-006	0.0132554	2.03253	2.03253
Pigq	Pigq-005	0.0121243	4.44926	4.44926
Pigs	Pigs-001	2.23152e-06	0.541479	-1.84679
Pigt	Pigt-001	0.0295313	0.788419	-1.26836
Pigu	Pigu-003	0.0191726	0.680208	-1.47014
Pigu	Pigu-001	0.00582652	0.838773	-1.19222
Pigx	Pigx-001	0.0171013	0.562617	-1.77741
Pigx	Pigx-008	0.0303965	2.24369	2.24369
Pik3c2a	Pik3c2a-001	0.000218503	1.28671	1.28671
Pik3c2a	Pik3c2a-006	0.00974307	1.80971	1.80971
Pik3cb	Pik3cb-002	0.0127167	1.27152	1.27152
Pik3cd	Pik3cd-008	0.0349497	0.479326	-2.08626
Pik3ip1	Pik3ip1-001	0.00872629	0.423515	-2.36119
Pik3r1	Pik3r1-001	0.00192832	0.657286	-1.52141
Pik3r2	Pik3r2-001	0.0220177	1.43394	1.43394
Pik3r3	Pik3r3-001	0.0163207	0.778346	-1.28477
Pikfyve	Pikfyve-001	0.013169	0.789731	-1.26625
Pim1	Pim1-001	0.00932178	0.756956	-1.32108
Pim2	Pim2-001	0.0345158	1.16285	1.16285
Pim3	Pim3-201	0.00722347	1.37794	1.37794
Pip4k2b	Pip4k2b-001	0.0111627	0.788636	-1.26801
Pip4k2c	Pip4k2c-201	0.0198482	0.760304	-1.31526
Pip5k1a	Pip5k1a-004	0.0145797	0.810294	-1.23412
Pir	Pir-001	0.0431051	1.31764	1.31764
Pirt	Pirt-001	8.50788e-05	0.524771	-1.90559
Pisd	Pisd-005	0.00184139	0.148234	-6.7461
Pisd	Pisd-003	0.0384464	0.349084	-2.86464
Pisd	Pisd-013	0.0312047	0.851812	-1.17397
Pisd-ps1	Pisd-ps1-003	0.00197567	0.404815	-2.47027
Pitpna	Pitpna-202	0.0442583	0.430811	-2.3212
Pitpnb	Pitpnb-001	0.0324589	1.30204	1.30204
Pitpnc1	Pitpnc1-004	0.0105591	18.1035	18.1035
Pitpnm1	Pitpnm1-001	0.000656572	0.387238	-2.58239
Pitpnm2	Pitpnm2-002	0.0262763	164.064	164.064
Piwil4	Piwil4-002	0.00693518	1.55809	1.55809
Pkd1	Pkd1-201	0.00866261	0.865682	-1.15516

Pkd2	Pkd2-001	0.0226638	0.685406	-1.45899
Pkhd1l1	Pkhd1l1-201	0.0294153	2.11255	2.11255
Pkhd1l1	Pkhd1l1-202	0.0232116	2.91458	2.91458
Pkia	Pkia-001	0.0355987	1.48916	1.48916
Pkig	Pkig-001	0.0194393	0.726581	-1.37631
Pkn3	Pkn3-005	0.0124472	0.182632	-5.47551
Pkn3	Pkn3-001	0.0168569	0.455333	-2.1962
Pknox1	Pknox1-001	0.0067602	0.829281	-1.20586
Pkp2	Pkp2-001	0.0265834	1.40232	1.40232
Pla2g12a	Pla2g12a-001	0.00518777	1.26245	1.26245
Pla2g4b	Pla2g4b-002	0.0316346	0.303609	-3.29371
Pla2g5	Pla2g5-001	0.0264451	191.885	191.885
Plaa	Plaa-003	0.0198302	0.714917	-1.39876
Platr16	Platr16-001	0.00146483	0.272165	-3.67424
Plbd2	Plbd2-001	0.00139838	0.794922	-1.25798
Plcb3	Plcb3-201	0.000496395	0.54433	-1.83712
Plcg1	Plcg1-005	0.0370387	0.398022	-2.51242
Plcg2	Plcg2-201	0.017967	5.39466	5.39466
Plcl2	Plcl2-201	0.00345572	1.178	1.178
Plcxd3	Plcxd3-001	0.0123542	0.728886	-1.37196
Pld6	Pld6-002	0.0426774	2.87023	2.87023
Plec	Plec-201	0.00634399	0.485985	-2.05768
Plec	Plec-205	0.0286806	0.501699	-1.99323
Plec	Plec-204	0.00519383	0.555643	-1.79972
Plec	Plec-005	0.00328548	0.573485	-1.74372
Plec	Plec-013	0.00324707	0.596859	-1.67544
Plec	Plec-003	0.0203898	0.60046	-1.66539
Plec	Plec-202	0.000673128	0.60795	-1.64487
Plec	Plec-006	0.0307817	0.716889	-1.39492
Plek2	Plek2-001	0.0336854	2.01631	2.01631
Plekha1	Plekha1-002	0.00996054	1.37362	1.37362
Plekha2	Plekha2-001	0.000534695	0.533792	-1.87339
Plekha2	Plekha2-003	0.0465102	7.51379	7.51379
Plekha3	Plekha3-001	0.00291184	0.757278	-1.32052
Plekha5	Plekha5-001	0.0163885	0.628502	-1.59109
Plekha6	Plekha6-001	0.00400165	0.650325	-1.53769
Plekha6	Plekha6-201	0.0382605	0.700013	-1.42854
Plekha6	Plekha6-004	0.00808752	1.44078	1.44078
Plekha6	Plekha6-003	0.0273498	1.6429	1.6429
Plekha6	Plekha6-002	0.00106884	9.65499	9.65499
Plekha7	Plekha7-006	0.0217519	0.69143	-1.44628
Plekhb2	Plekhb2-001	0.0442592	1.19309	1.19309
Plekhg1	Plekhg1-003	0.014738	0.396862	-2.51976

Plekhg2	Plekhg2-006	0.00185024	0.483656	-2.06759
Plekhg2	Plekhg2-003	0.0472972	0.59307	-1.68614
Plekhm3	Plekhm3-006	0.032972	5.88943	5.88943
Plgrkt	Plgrkt-002	0.0147481	2.44609	2.44609
Plin4	Plin4-201	0.0101178	1.46669	1.46669
Plin4	Plin4-001	0.0101175	1.4667	1.4667
Plk3	Plk3-001	0.046325	1.53955	1.53955
Plod1	Plod1-001	0.00169088	0.810284	-1.23413
Plod2	Plod2-002	0.00426247	0.725477	-1.3784
Plod3	Plod3-006	0.0364985	0.799984	-1.25002
Plxnb1	Plxnb1-001	0.0240449	0.660378	-1.51428
Plxnb2	Plxnb2-201	8.48707e-05	0.703391	-1.42168
Plxnb2	Plxnb2-001	0.00112407	0.728425	-1.37283
Pmch	Pmch-001	0.0409679	0.531602	-1.8811
Pmpca	Pmpca-004	0.0295628	1.75809	1.75809
Pmpcb	Pmpcb-001	0.0218855	1.1124	1.1124
Pms1	Pms1-001	7.29994e-05	1.57022	1.57022
Pms1	Pms1-003	0.0174673	2.38946	2.38946
Pms2	Pms2-014	0.0373512	0.288583	-3.46521
Pnisr	Pnisr-006	0.00559246	2.25875	2.25875
Pnkd	Pnkd-004	0.0315816	0.604511	-1.65423
Pnkd	Pnkd-003	0.0341003	0.793249	-1.26064
Pnmal1	Pnmal1-001	0.000256813	1.2481	1.2481
Pnpla2	Pnpla2-006	0.0304867	0.533535	-1.87429
Pnpla2	Pnpla2-005	0.0114388	0.546449	-1.83
Pnpla2	Pnpla2-002	0.00485665	0.594015	-1.68346
Pnpla7	Pnpla7-001	0.0272937	1.43574	1.43574
Poc1b	Poc1b-003	0.00840233	0.619377	-1.61453
Poc5	Poc5-201	0.0422027	1.18067	1.18067
Pogk	Pogk-003	0.0246928	1.50318	1.50318
Pogk	Pogk-001	0.035111	1.8703	1.8703
Poglut1	Poglut1-005	0.0135629	0.664898	-1.50399
Pogz	Pogz-010	0.00283257	0.256458	-3.89927
Pogz	Pogz-004	0.0429583	1.87044	1.87044
Polb	Polb-201	2.05382e-05	1.28346	1.28346
Pold3	Pold3-002	0.0175666	2.89466	2.89466
Poldip2	Poldip2-002	0.0303104	1.64836	1.64836
Polg	Polg-004	0.0368296	0.515469	-1.93998
Polg	Polg-001	0.0099976	0.631425	-1.58372
Polh	Polh-201	0.00770376	1.19796	1.19796
Poli	Poli-003	0.00838478	1.78514	1.78514
Polk	Polk-202	0.00247116	2.26326	2.26326
Polr1c	Polr1c-001	0.00557569	1.37238	1.37238

Polr1c	Polr1c-007	0.0421662	1.90768	1.90768
Polr2c	Polr2c-201	0.0382882	1.24037	1.24037
Polr2e	Polr2e-003	0.0382656	0.390957	-2.55783
Polr2e	Polr2e-001	0.000288314	0.68605	-1.45762
Polr2h	Polr2h-001	0.0373219	0.868569	-1.15132
Polr2k	Polr2k-002	0.0319672	0.783472	-1.27637
Polrmt	Polrmt-003	0.0444584	1.80014	1.80014
Pomc	Pomc-201	0.0025404	1.86415	1.86415
Pomgnt1	Pomgnt1-012	0.0315118	1.95044	1.95044
Pomt1	Pomt1-012	0.000493601	0.116628	-8.57428
Pomt1	Pomt1-001	0.00850847	0.559127	-1.7885
Pop5	Pop5-005	0.041371	1.78403	1.78403
Popdc2	Popdc2-003	0.0498642	0.0540611	-18.4976
Popdc3	Popdc3-002	0.00167297	3.27784	3.27784
Pot1a	Pot1a-001	0.000514379	1.43091	1.43091
Pot1a	Pot1a-201	0.0161407	1.956	1.956
Pot1b	Pot1b-201	0.00292472	1.29874	1.29874
Pou2f1	Pou2f1-201	0.034869	0.8816	-1.1343
Ppap2a	Ppap2a-201	0.00128973	0.62632	-1.59663
Ppap2a	Ppap2a-202	0.0119948	0.686706	-1.45623
Ppap2c	Ppap2c-001	0.0249194	0.699712	-1.42916
Ppapdc1b	Ppapdc1b-005	0.042428	3.9687	3.9687
Ppapdc2	Ppapdc2-001	0.0295856	1.3944	1.3944
Ppara	Ppara-001	0.0178378	2.45625	2.45625
Ppfia1	Ppfia1-004	0.0444694	0.736338	-1.35807
Ppfia4	Ppfia4-007	0.0136698	2.90238	2.90238
Ppfibp1	Ppfibp1-005	0.00706687	0.570194	-1.75379
Ppfibp1	Ppfibp1-001	0.00879466	0.693115	-1.44276
Ppfibp1	Ppfibp1-002	0.013407	0.698384	-1.43188
Ppfibp2	Ppfibp2-201	0.0375422	1.19746	1.19746
Pphln1	Pphln1-201	0.00882753	1.74489	1.74489
Ppig	Ppig-002	0.00573552	1.18605	1.18605
Ppig	Ppig-011	0.00695324	1.44332	1.44332
Ppig	Ppig-005	0.0308728	3.03128	3.03128
Ppil3	Ppil3-006	0.0367681	0.356219	-2.80727
Ppil4	Ppil4-001	0.00513841	1.49048	1.49048
Ppil4	Ppil4-003	0.000281677	1.92739	1.92739
Ppip5k1	Ppip5k1-005	0.0274772	1.7169	1.7169
Ppip5k1	Ppip5k1-014	0.0244367	2.60642	2.60642
Ppm1b	Ppm1b-202	0.00136456	0.711193	-1.40609
Ppm1e	Ppm1e-001	0.000673669	1.16053	1.16053
Ppm1h	Ppm1h-002	0.035298	1.69104	1.69104
Ppm1l	Ppm1l-001	0.00159763	0.56943	-1.75614

Ppm1m	Ppm1m-004	0.00924153	0.40506	-2.46877
Ppox	Ppox-001	0.0354198	0.69854	-1.43156
Ppp1r12b	Ppp1r12b-004	0.00624243	3.7998	3.7998
Ppp1r13b	Ppp1r13b-201	0.0116816	0.793713	-1.2599
Ppp1r14b	Ppp1r14b-201	0.000873986	0.398838	-2.50729
Ppp1r14c	Ppp1r14c-201	0.000286673	0.759462	-1.31672
Ppp1r15a	Ppp1r15a-001	0.000118597	2.57319	2.57319
Ppp1r16b	Ppp1r16b-003	0.00422136	2.29483	2.29483
Ppp1r18	Ppp1r18-001	0.0425805	0.700026	-1.42852
Ppp1r2	Ppp1r2-002	0.045735	1.26188	1.26188
Ppp1r32	Ppp1r32-201	0.0366854	2.22046	2.22046
Ppp1r35	Ppp1r35-001	0.0407803	0.72287	-1.38338
Ppp1r3b	Ppp1r3b-201	0.0493387	1.33476	1.33476
Ppp1r9a	Ppp1r9a-001	0.0168723	0.831137	-1.20317
Ppp2r1a	Ppp2r1a-001	0.0227159	0.876119	-1.1414
Ppp2r2a	Ppp2r2a-201	0.000223189	1.27592	1.27592
Ppp2r3c	Ppp2r3c-201	0.0305524	1.18779	1.18779
Ppp2r3d	Ppp2r3d-003	0.00196742	0.651263	-1.53548
Ppp2r4	Ppp2r4-003	0.0108655	0.436862	-2.28905
Ppp2r4	Ppp2r4-001	0.00944473	0.637216	-1.56933
Ppp2r5a	Ppp2r5a-001	0.00385526	1.23788	1.23788
Ppp2r5a	Ppp2r5a-005	0.0421492	2.83666	2.83666
Ppp3ca	Ppp3ca-001	0.0184451	0.841934	-1.18774
Ppp5c	Ppp5c-004	0.0269859	3.24653e-07	-3.08021e+06
Ppp6c	Ppp6c-001	0.00297795	0.727747	-1.3741
Ppp6c	Ppp6c-003	0.00046811	1.53608	1.53608
Ppp6c	Ppp6c-005	0.00323567	1.96996	1.96996
Ppp6r3	Ppp6r3-203	0.00862468	1.4619	1.4619
Ppt1	Ppt1-001	0.0015396	0.659407	-1.51651
Pqbp1	Pqbp1-003	0.0260586	1.60033	1.60033
Pqlc2	Pqlc2-003	0.000277554	2.94704	2.94704
Praf2	Praf2-001	0.0158262	1.31111	1.31111
Prc1	Prc1-013	0.0223485	0.463502	-2.15749
Prdm11	Prdm11-001	0.040142	0.397887	-2.51328
Prdm16	Prdm16-013	0.0325189	0.00608354	-164.378
Prdm2	Prdm2-001	0.00723628	1.1306	1.1306
Prdm9	Prdm9-201	0.0156151	1.51793	1.51793
Prdx2	Prdx2-004	0.00357214	0.222082	-4.50285
Prdx2	Prdx2-003	0.021456	0.609614	-1.64038
Prdx4	Prdx4-002	0.0189841	0.761878	-1.31255
Prdx4	Prdx4-001	0.0174629	0.888405	-1.12561
Preb	Preb-003	0.0326582	0.177096	-5.64665
Preb	Preb-008	0.000196965	1.19556	1.19556

Preb	Preb-007	0.0362064	2.80536	2.80536
Prex1	Prex1-001	0.000979655	0.6847	-1.46049
Prickle2	Prickle2-003	0.0172594	0.641453	-1.55896
Prim1	Prim1-002	0.000564982	1.92502	1.92502
Prkag2	Prkag2-010	0.0124353	1.63845	1.63845
Prkag2	Prkag2-001	0.0307758	1.88227	1.88227
Prkar2b	Prkar2b-001	0.000728208	0.595116	-1.68034
Prkca	Prkca-002	0.0236721	3.12317	3.12317
Prkcd	Prkcd-202	0.0412313	0.496426	-2.0144
Prkcd	Prkcd-001	0.0287422	0.735164	-1.36024
Prkce	Prkce-001	0.00728615	1.19344	1.19344
Prkce	Prkce-003	0.0381406	17.8456	17.8456
Prkcq	Prkcq-002	0.0351565	0.0374414	-26.7084
Prkcz	Prkcz-001	0.000361575	2.0631	2.0631
Prkrip1	Prkrip1-004	0.0037895	1.64921	1.64921
Prkx	Prkx-001	0.000177895	0.470512	-2.12535
Prmt10	Prmt10-201	0.0162105	1.3429	1.3429
Prmt3	Prmt3-001	0.000224663	1.21739	1.21739
Prmt7	Prmt7-002	0.020203	0.476309	-2.09948
Prmt7	Prmt7-001	0.0457439	1.14269	1.14269
Procr	Procr-001	0.0144659	0.485187	-2.06106
Prokr2	Prokr2-001	0.0335295	0.131225	-7.62051
Prorsd1	Prorsd1-001	0.000241463	1.78737	1.78737
Prosc	Prosc-202	0.0157911	1.27303	1.27303
Proser3	Proser3-002	0.00998343	1.44503	1.44503
Prpf18	Prpf18-002	0.000681666	2.20856	2.20856
Prpf38b	Prpf38b-007	0.0118904	0.762622	-1.31127
Prpf40a	Prpf40a-005	0.000986922	1.26447	1.26447
Prpf40a	Prpf40a-002	0.0202557	1.2882	1.2882
Prpf6	Prpf6-201	0.00181183	1.39471	1.39471
Prpf8	Prpf8-001	0.0450205	1.33603	1.33603
Prpf8	Prpf8-002	0.00476283	1.519	1.519
Prps1	Prps1-001	0.00531503	0.71643	-1.39581
Prpsap1	Prpsap1-006	0.00994923	1.62492	1.62492
Prpsap2	Prpsap2-201	0.011844	2.66359	2.66359
Prr13	Prr13-001	0.0219713	0.404354	-2.47308
Prr14	Prr14-002	0.0383922	0.680511	-1.46948
Prr22	Prr22-001	0.0356311	2.55721	2.55721
Prr7	Prr7-201	0.0225239	1.77563	1.77563
Prrc2a	Prrc2a-005	0.000722505	0.595589	-1.67901
Prrc2a	Prrc2a-006	0.0339054	1.82735	1.82735
Prrc2b	Prrc2b-001	0.00275829	0.532488	-1.87798
Prrc2b	Prrc2b-002	0.0155123	2.89609	2.89609

Prrc2c	Prrc2c-003	0.0302501	1.22007	1.22007
Prrc2c	Prrc2c-005	0.0140814	1.42757	1.42757
Prrc2c	Prrc2c-006	0.0261456	1.43419	1.43419
Prrg2	Prrg2-201	0.0164742	1.49284	1.49284
Prrt2	Prrt2-004	0.027045	75.7807	75.7807
Prrt3	Prrt3-003	0.0321974	3.27647	3.27647
Prrxl1	Prrxl1-002	0.0164162	3.09219	3.09219
Prss16	Prss16-004	0.0395807	0.36135	-2.7674
Prss33	Prss33-003	0.0268621	0.304352	-3.28567
Prss35	Prss35-201	0.0478574	6.05127	6.05127
Prss36	Prss36-002	0.0330097	4.05072	4.05072
Prss41	Prss41-003	0.0362909	1.33156	1.33156
Prtg	Prtg-001	0.00119125	0.776674	-1.28754
Prx	Prx-002	0.0207306	0.168603	-5.93109
Psap	Psap-201	0.0095693	0.559679	-1.78674
Psat1	Psat1-001	0.00341281	1.1406	1.1406
Psat1	Psat1-002	0.0102324	1.72985	1.72985
Psat1	Psat1-007	0.0292817	2.79721	2.79721
Psd2	Psd2-006	0.0204224	7.8208	7.8208
Psd4	Psd4-201	0.0256121	0.0569595	-17.5563
Psd4	Psd4-002	0.0116943	865.979	865.979
Psenen	Psenen-201	0.00893916	1.18497	1.18497
Psg16	Psg16-002	0.00950884	5.43245	5.43245
Psg29	Psg29-001	0.00942673	2.08298	2.08298
Psip1	Psip1-002	0.0185377	1.27483	1.27483
Psip1	Psip1-008	0.0337292	2.59203	2.59203
Psma1	Psma1-002	0.0196628	1.50677	1.50677
Psma4	Psma4-003	0.00521908	2.40105	2.40105
Psmb1	Psmb1-201	0.0466136	1.07665	1.07665
Psmb6	Psmb6-001	0.00380752	1.23893	1.23893
Psmb6	Psmb6-003	0.0143136	1.36746	1.36746
Psmb7	Psmb7-001	0.00623748	0.831774	-1.20225
Psmb8	Psmb8-001	0.00533905	0.794649	-1.25842
Psmc3	Psmc3-010	0.0364235	1.43017	1.43017
Psmc3	Psmc3-002	0.0383154	1.56422	1.56422
Psmc6	Psmc6-201	0.00141194	1.22165	1.22165
Psmd11	Psmd11-004	0.009063	1.50452	1.50452
Psmd11	Psmd11-009	0.00197399	2.19046	2.19046
Psmd4	Psmd4-007	0.0054822	2.89363	2.89363
Psmd5	Psmd5-002	0.0294964	1.41337	1.41337
Psmd6	Psmd6-003	0.0481811	1.62978	1.62978
Psmd9	Psmd9-005	0.00711523	1.43725	1.43725
Psme1	Psme1-010	0.0260149	0.683595	-1.46285

Psme2	Psme2-201	0.0197416	0.564058	-1.77287
Psme2	Psme2-001	0.0358325	0.781098	-1.28025
Psmf1	Psmf1-006	0.00324736	1.54626	1.54626
Psmg4	Psmg4-002	0.0447575	3.3722	3.3722
Psph	Psph-001	0.00185148	1.76022	1.76022
Psph	Psph-004	0.00729966	2.32323	2.32323
Psph	Psph-007	0.00476651	2.72016	2.72016
Pstk	Pstk-001	0.0131554	1.6029	1.6029
Ptar1	Ptar1-201	0.0233669	1.2734	1.2734
Ptbp1	Ptbp1-008	0.0190229	1.56258	1.56258
Ptbp1	Ptbp1-010	0.0191219	2.49566	2.49566
Ptbp1	Ptbp1-013	0.00607067	2.81957	2.81957
Ptbp2	Ptbp2-201	1.14131e-05	0.711864	-1.40476
Ptcd2	Ptcd2-201	0.0166538	1.4035	1.4035
Ptcd3	Ptcd3-010	0.0265188	1.36854	1.36854
Ptch1	Ptch1-005	0.0108161	1.11043	1.11043
Ptdss2	Ptdss2-001	0.0400051	0.825499	-1.21139
Pten	Pten-001	3.98366e-05	0.501303	-1.9948
Ptges3	Ptges3-002	0.00789106	1.24421	1.24421
Ptges3	Ptges3-003	0.034022	1.60852	1.60852
Ptgis	Ptgis-001	0.0482345	0.20467	-4.88592
Ptgs1	Ptgs1-004	0.0195455	0.669748	-1.4931
Ptov1	Ptov1-201	0.000825935	0.693573	-1.44181
Ptp4a1	Ptp4a1-004	0.0117698	0.214392	-4.66435
Ptp4a1	Ptp4a1-006	0.0173848	1.30972	1.30972
Ptp4a1	Ptp4a1-005	0.0152358	1.34433	1.34433
Ptp4a2	Ptp4a2-201	0.0283946	0.646783	-1.54611
Ptp4a2	Ptp4a2-006	0.00131631	1.74662	1.74662
Ptpn1	Ptpn1-006	0.0147387	4.22421	4.22421
Ptpn11	Ptpn11-002	0.010912	0.584275	-1.71152
Ptpn14	Ptpn14-002	0.0258181	10448	10448
Ptpn2	Ptpn2-001	0.0090032	1.31457	1.31457
Ptpn22	Ptpn22-001	0.0245007	0.0865288	-11.5568
Ptpn3	Ptpn3-001	0.000160322	1.42021	1.42021
Ptpn4	Ptpn4-001	2.4082e-05	0.795716	-1.25673
Ptpn4	Ptpn4-003	0.0168885	3.6185	3.6185
Ptpn4	Ptpn4-002	0.0133238	7.12405	7.12405
Ptpre	Ptpre-201	0.00280179	0.710011	-1.40843
Ptprf	Ptprf-001	0.00573438	0.859664	-1.16325
Ptprg	Ptprg-001	0.000256577	0.692819	-1.44338
Ptprg	Ptprg-002	0.0114979	1.56448	1.56448
Ptprm	Ptprm-201	0.026925	2.39396	2.39396
Ptprn2	Ptprn2-002	0.00437419	0.688019	-1.45345

Ptprv	Ptprv-010	0.0472716	0.357638	-2.79612
Ptrh1	Ptrh1-001	0.00532472	1.66456	1.66456
Pum2	Pum2-005	0.00387796	2.25794	2.25794
Pum2	Pum2-003	0.0215924	2.54105	2.54105
Pura	Pura-001	0.00777714	0.856906	-1.16699
Purb	Purb-001	0.0260856	0.685659	-1.45845
Pus1	Pus1-004	0.0481652	0.516061	-1.93775
Pus1	Pus1-003	0.0289742	1.70205	1.70205
Pus10	Pus10-007	0.0230461	4.43418	4.43418
Pus3	Pus3-001	0.00848104	1.67606	1.67606
Pvr	Pvr-001	0.045132	1.19799	1.19799
Pvrl1	Pvrl1-201	0.00309072	0.809519	-1.2353
Pvrl2	Pvrl2-201	0.0482338	0.624927	-1.60019
Pvrl3	Pvrl3-003	0.0066026	0.701807	-1.42489
Pvrl4	Pvrl4-001	0.0014066	2.9431	2.9431
Pvrl4	Pvrl4-002	0.00140665	2.9431	2.9431
Pwwp2a	Pwwp2a-004	0.00134414	0.545284	-1.83391
Pwwp2a	Pwwp2a-002	0.000734731	0.698614	-1.43141
Pwwp2a	Pwwp2a-001	0.027783	0.810993	-1.23306
Pxdn	Pxdn-001	6.80436e-05	0.632961	-1.57988
Pxylp1	Pxylp1-001	0.00867656	1.8099	1.8099
Pycr1	Pycr1-005	0.0364071	16271.6	16271.6
Pygb	Pygb-001	0.027221	0.881676	-1.1342
Qars	Qars-009	0.00989361	0.238888	-4.18606
Qars	Qars-003	0.0490724	0.653747	-1.52964
Qars	Qars-001	0.0470331	0.768248	-1.30166
Qars	Qars-007	0.0456381	3.93366	3.93366
Qdpr	Qdpr-007	0.0411725	4.63481	4.63481
Qk	Qk-201	0.0242405	1.19431	1.19431
Qrfp	Qrfp-001	0.0343714	0.402344	-2.48543
Qrich1	Qrich1-011	0.00640317	0.373562	-2.67693
Qrsl1	Qrsl1-001	0.0283203	1.40334	1.40334
R3hcc1	R3hcc1-001	0.0172048	1.42573	1.42573
R3hcc1l	R3hcc1l-001	0.0371827	1.16635	1.16635
R3hcc1l	R3hcc1l-003	0.00612638	2.05416	2.05416
R3hdm1	R3hdm1-010	0.00201816	0.360181	-2.77639
R3hdm1	R3hdm1-001	0.00278847	1.22694	1.22694
R3hdm2	R3hdm2-018	0.0213214	0.191851	-5.21238
R3hdm4	R3hdm4-201	4.13364e-05	0.617108	-1.62046
R74862	R74862-003	0.0354555	0.175809	-5.68798
Rab10os	Rab10os-001	0.00195464	1.2637	1.2637
Rab10os	Rab10os-009	0.0117642	1.80843	1.80843
Rab10os	Rab10os-010	0.0315391	2.11662	2.11662

Rab11a	Rab11a-001	0.00169224	0.652013	-1.53371
Rab13	Rab13-001	0.0204097	0.720158	-1.38858
Rab1a	Rab1a-002	6.97805e-05	1.53281	1.53281
Rab22a	Rab22a-003	0.00276615	1.50726	1.50726
Rab23	Rab23-006	0.0386981	2.06418	2.06418
Rab23	Rab23-009	0.0354257	2.9069	2.9069
Rab24	Rab24-201	0.0132626	0.708997	-1.41044
Rab26	Rab26-005	0.0440791	2.25336	2.25336
Rab2b	Rab2b-003	0.0216484	1.95761	1.95761
Rab2b	Rab2b-008	0.0243493	318.166	318.166
Rab32	Rab32-001	0.000597583	1.62424	1.62424
Rab33a	Rab33a-001	0.00692844	2.05252	2.05252
Rab34	Rab34-004	0.000726291	4.05201e+06	4.05201e+06
Rab36	Rab36-004	0.00453162	1.51923	1.51923
Rab39	Rab39-001	0.0374031	1.10904	1.10904
Rab39b	Rab39b-001	0.00312792	1.43674	1.43674
Rab3gap1	Rab3gap1-001	5.622e-05	1.29378	1.29378
Rab3gap2	Rab3gap2-001	0.0400497	0.655069	-1.52656
Rab3gap2	Rab3gap2-003	0.0182783	1.16004	1.16004
Rab3ip	Rab3ip-201	0.00959831	1.17711	1.17711
Rab4b	Rab4b-001	0.00110185	0.792722	-1.26148
Rab5b	Rab5b-201	0.000466869	1.24795	1.24795
Rab8b	Rab8b-201	0.0110462	0.843182	-1.18598
Rab9b	Rab9b-001	0.0246093	1.8615	1.8615
Rabac1	Rabac1-006	0.0186525	0.39372	-2.53988
Rabep1	Rabep1-004	0.00133215	2.45689	2.45689
Rabgap1	Rabgap1-003	0.0221186	17.1726	17.1726
Rabgap1l	Rabgap1l-003	0.00185975	0.739995	-1.35136
Rabgap1l	Rabgap1l-011	3.66382e-05	18.4724	18.4724
Rabggtb	Rabggtb-011	0.0323889	0.00215959	-463.05
Rabggtb	Rabggtb-002	0.0372313	1.16897	1.16897
Rabggtb	Rabggtb-003	0.0491778	3.01667	3.01667
Rabif	Rabif-001	0.000190519	1.30919	1.30919
Rabl2	Rabl2-004	0.0390485	2.41848	2.41848
Rabl3	Rabl3-001	0.00577602	1.27593	1.27593
Rabl6	Rabl6-001	0.0213576	0.925	-1.08108
Rabl6	Rabl6-004	0.00236773	4.53197	4.53197
Rac1	Rac1-003	0.0139187	1.2737	1.2737
Racgap1	Racgap1-002	0.0456364	0.848755	-1.1782
Rad17	Rad17-201	0.0243219	1.57353	1.57353
Rad18	Rad18-003	0.000311622	1.33066	1.33066
Rad23a	Rad23a-002	0.00571729	0.515008	-1.94172
Rad23b	Rad23b-001	0.0227704	1.2445	1.2445

Rad23b	Rad23b-003	0.0279043	1.45774	1.45774
Rad51ap1	Rad51ap1-001	0.0129283	1.41548	1.41548
Rad51c	Rad51c-002	0.0217375	0.560402	-1.78443
Rad52	Rad52-003	0.0220564	0.598926	-1.66965
Rad52	Rad52-014	0.00485644	0.673939	-1.48381
Rad54l2	Rad54l2-001	0.000258589	0.506635	-1.97381
Rad9a	Rad9a-201	0.000849944	0.64329	-1.55451
Ralbp1	Ralbp1-202	0.0424431	1.44179	1.44179
Ralgapa1	Ralgapa1-202	0.00306064	0.763183	-1.3103
Ralgapa2	Ralgapa2-005	0.00994833	0.00167716	-596.246
Ralgapa2	Ralgapa2-010	0.0291451	0.0227758	-43.9063
Ralgapb	Ralgapb-001	0.0389192	1.32981	1.32981
Ralgapb	Ralgapb-201	0.0141463	1.39921	1.39921
Ralgapb	Ralgapb-004	0.0365224	5.75332	5.75332
Ralgds	Ralgds-004	0.0203346	0.441705	-2.26395
Ralgds	Ralgds-001	0.00858913	0.646573	-1.54662
Ralgps2	Ralgps2-001	3.40983e-05	0.539523	-1.85349
Ralgps2	Ralgps2-008	0.0380488	1.53684	1.53684
Raly	Raly-003	0.0326971	0.748116	-1.33669
Ramp2	Ramp2-001	0.017759	0.439768	-2.27393
Ranbp17	Ranbp17-002	0.00265095	1.78587	1.78587
Ranbp6	Ranbp6-001	0.017254	1.35128	1.35128
Rap1a	Rap1a-001	0.0450783	0.758716	-1.31802
Rap1a	Rap1a-002	0.02596	1.21574	1.21574
Rap1a	Rap1a-004	0.00102357	1.42753	1.42753
Rap1a-ps2	Rap1a-ps2-001	0.0179777	3.89919	3.89919
Rap2a	Rap2a-001	0.00746252	0.569096	-1.75717
Rapgef1	Rapgef1-007	0.0258579	1.38412	1.38412
Rapgef1	Rapgef1-006	0.00421351	2.54331	2.54331
Rapgef1	Rapgef1-005	0.00305095	2.83835	2.83835
Rapgef2	Rapgef2-001	0.0470946	1.39245	1.39245
Rapgef3	Rapgef3-002	0.037638	0.217257	-4.60284
Rapgef5	Rapgef5-201	0.009623	1.18116	1.18116
Rapgef6	Rapgef6-008	0.0182373	0.376366	-2.65699
Rapgef6	Rapgef6-003	0.00782516	4.26541	4.26541
Raph1	Raph1-201	0.0408063	16.4598	16.4598
Raph1	Raph1-005	0.0185703	17.9447	17.9447
Rapsn	Rapsn-004	0.0312549	2.24195e+06	2.24195e+06
Rara	Rara-004	0.0030389	0.723657	-1.38187
Rarg	Rarg-006	0.0295184	7.31992	7.31992
Rasa3	Rasa3-001	0.00169761	0.682774	-1.46461
Rasal2	Rasal2-002	0.00321943	2.13892	2.13892
Rasgrp2	Rasgrp2-015	0.00246623	1.66101e-05	-60204.4

Rasgrp2	Rasgrp2-004	0.0309436	0.12809	-7.80698
Rasgrp2	Rasgrp2-018	0.0379413	0.253369	-3.94681
Rasl11b	Rasl11b-003	0.00466543	0.0300663	-33.2599
Rassf1	Rassf1-004	0.00779455	1.57521	1.57521
Rassf3	Rassf3-201	2.81624e-05	0.723263	-1.38262
Rb1cc1	Rb1cc1-001	0.00609351	0.397573	-2.51526
Rb1cc1	Rb1cc1-011	0.0173378	1.35603	1.35603
Rbbp4	Rbbp4-001	0.0212945	0.807998	-1.23763
Rbbp5	Rbbp5-001	0.0188482	2.30144	2.30144
Rbbp5	Rbbp5-003	0.0228164	2.43332	2.43332
Rbbp9	Rbbp9-001	7.91442e-05	1.38367	1.38367
Rbck1	Rbck1-007	0.00878557	1.27397	1.27397
Rbck1	Rbck1-003	0.0230367	3.67705	3.67705
Rbl2	Rbl2-001	0.031005	0.888898	-1.12499
Rbm12	Rbm12-001	0.0151878	0.755418	-1.32377
Rbm15	Rbm15-003	0.00729957	1.34678	1.34678
Rbm15	Rbm15-001	0.0331386	1.66603	1.66603
Rbm18	Rbm18-001	0.00178736	1.25261	1.25261
Rbm20	Rbm20-201	0.00871893	2.90179	2.90179
Rbm22	Rbm22-002	0.0442905	0.728451	-1.37278
Rbm25	Rbm25-002	0.00276859	0.843814	-1.1851
Rbm26	Rbm26-004	0.0013411	0.815213	-1.22667
Rbm28	Rbm28-003	0.0280315	1.24275	1.24275
Rbm28	Rbm28-002	0.0216818	1.37439	1.37439
Rbm33	Rbm33-003	0.00445553	1.63375	1.63375
Rbm38	Rbm38-001	0.0445791	29.5243	29.5243
Rbm39	Rbm39-025	0.0282581	2.09576	2.09576
Rbm3os	Rbm3os-001	0.0499775	1.32593	1.32593
Rbm43	Rbm43-005	0.0398374	0.271339	-3.68542
Rbm46	Rbm46-001	0.015871	6.08531	6.08531
Rbm46	Rbm46-004	0.015871	6.08531	6.08531
Rbm4b	Rbm4b-004	0.0435635	9.02492	9.02492
Rbm5	Rbm5-011	0.0340429	0.011494	-87.0022
Rbm7	Rbm7-201	0.0191468	1.12948	1.12948
Rbm8a	Rbm8a-001	0.042692	0.547162	-1.82761
Rbms1	Rbms1-001	0.00157804	0.727095	-1.37534
Rbms3	Rbms3-001	0.00672402	0.601969	-1.66121
Rbpms	Rbpms-201	0.0315213	0.654126	-1.52876
Rbpms2	Rbpms2-201	0.00737298	0.016545	-60.4413
Rc3h1	Rc3h1-002	0.00902148	1.70151	1.70151
Rc3h2	Rc3h2-006	0.018622	1.83587	1.83587
Rcan3	Rcan3-001	0.0471807	0.924028	-1.08222
Rcan3	Rcan3-003	0.0321006	168.461	168.461

Rcbtb1	Rcbtb1-001	0.0161919	1.33018	1.33018
Rcbtb1	Rcbtb1-007	0.0387462	3.50872	3.50872
Rcbtb2	Rcbtb2-202	0.0169105	0.143685	-6.95966
Rcbtb2	Rcbtb2-008	0.0194724	0.22877	-4.37121
Rcn1	Rcn1-002	0.0262001	0.754703	-1.32502
Rcn2	Rcn2-001	0.0442004	0.929866	-1.07542
Rcor2	Rcor2-003	0.0071567	0.428106	-2.33587
Rcor3	Rcor3-007	0.0152046	1.57217	1.57217
Recql4	Recql4-201	0.0489569	1.54045	1.54045
Recql5	Recql5-007	0.00968784	0.118165	-8.46273
Recql5	Recql5-003	0.0141961	0.338024	-2.95837
Reep6	Reep6-201	0.0100601	1.56773	1.56773
Relb	Relb-001	0.00365665	0.575002	-1.73912
Relb	Relb-201	0.00365658	0.575008	-1.73911
Rell2	Rell2-008	0.0262347	0.410747	-2.43459
Reln	Reln-013	0.0102828	9.33417	9.33417
Renbp	Renbp-001	0.0225104	0.709153	-1.41013
Renbp	Renbp-010	0.0286813	31.3993	31.3993
Rep15	Rep15-001	0.0142815	0.360945	-2.7705
Reps1	Reps1-005	0.0129289	1.11364	1.11364
Reps1	Reps1-001	0.00187689	1.24837	1.24837
Reps1	Reps1-006	0.0131404	10861.3	10861.3
Rere	Rere-004	0.0252193	0.290608	-3.44107
Rere	Rere-008	0.0111614	2.42622	2.42622
Rest	Rest-002	0.00269198	0.611885	-1.6343
Ret	Ret-002	0.000737876	0.665773	-1.50201
Ret	Ret-001	0.0439749	0.758847	-1.31779
Retsat	Retsat-004	0.0262968	0.573015	-1.74515
Rev1	Rev1-010	0.0195658	0.357597	-2.79644
Rexo1	Rexo1-007	0.00577793	0.239219	-4.18027
Rexo4	Rexo4-008	0.00479809	2.02054e+11	2.02054e+11
Rfc1	Rfc1-002	0.0403211	0.517693	-1.93165
Rfc1	Rfc1-004	0.0369415	0.747195	-1.33834
Rfesd	Rfesd-203	0.000225828	1.502	1.502
Rfk	Rfk-001	0.00534501	1.49062	1.49062
Rftn1	Rftn1-001	0.0164427	0.485718	-2.05881
Rfwd2	Rfwd2-008	0.0216186	1.35372	1.35372
Rfx2	Rfx2-202	0.0445928	1.53523	1.53523
Rfxap	Rfxap-002	0.000223642	2.72487	2.72487
Rgl2	Rgl2-001	0.0220505	0.761538	-1.31313
Rgl2	Rgl2-010	0.00299575	1.38936	1.38936
Rgs10	Rgs10-001	0.0302838	0.745113	-1.34208
Rgs11	Rgs11-004	0.0184073	2.77275	2.77275

Rgs19	Rgs19-006	0.0132821	2.87978	2.87978
Rgs19	Rgs19-011	0.0233994	4.27845	4.27845
Rgs3	Rgs3-017	0.0370764	3.6113e-06	-276909
Rgs3	Rgs3-008	0.0199278	0.735563	-1.3595
Rgs3	Rgs3-001	0.0192094	2.84773	2.84773
Rgs7	Rgs7-001	0.00326613	1.54445	1.54445
Rgs7	Rgs7-201	0.0223372	2.06465	2.06465
Rhbdd1	Rhbdd1-003	0.0103923	2.43944	2.43944
Rhbdd2	Rhbdd2-003	0.0297311	1.71518	1.71518
Rhbdd2	Rhbdd2-005	0.0461315	3.50501	3.50501
Rhbdd2	Rhbdd2-006	0.0354833	294.894	294.894
Rhbdd3	Rhbdd3-002	0.0486067	0.603876	-1.65597
Rhbdf1	Rhbdf1-008	0.019758	0.611302	-1.63585
Rhbdf1	Rhbdf1-006	0.0379283	0.695943	-1.4369
Rhbdl2	Rhbdl2-001	0.0191134	1.88902	1.88902
Rhbdl2	Rhbdl2-201	0.0191134	1.88902	1.88902
Rhd	Rhd-001	0.00114931	1.55674	1.55674
Rheb	Rheb-001	0.00466772	1.21199	1.21199
Rheb	Rheb-003	0.0194512	1.39309	1.39309
Rhebl1	Rhebl1-002	0.00795132	1.47923	1.47923
Rhoa	Rhoa-005	0.0182236	0.330059	-3.02976
Rhobtb1	Rhobtb1-003	0.0485942	11.988	11.988
Rhobtb3	Rhobtb3-002	0.0322746	1.9586	1.9586
Rhoc	Rhoc-001	0.000161502	0.586314	-1.70557
Rhoc	Rhoc-002	0.0201359	0.619461	-1.61431
Rhoc	Rhoc-005	0.0145101	0.718403	-1.39198
Rhot1	Rhot1-002	0.0495591	1.25786	1.25786
Rhot1	Rhot1-001	0.0426174	1.65495	1.65495
Rhot2	Rhot2-009	0.0353846	215.447	215.447
Rhot2	Rhot2-006	0.0441361	569.184	569.184
Rhox1	Rhox1-001	0.0291463	1.47477	1.47477
Rhox2-ps	Rhox2-ps-001	0.0442543	1.41274	1.41274
Rhox2a	Rhox2a-001	0.000638985	1.31854	1.31854
Rhox2c	Rhox2c-001	0.0314472	1.40088	1.40088
Rhox2f	Rhox2f-001	0.00115632	1.39042	1.39042
Rhox4a	Rhox4a-001	0.0103618	2.04503	2.04503
Rhox4a2	Rhox4a2-001	0.042552	1.43704	1.43704
Rhox4b	Rhox4b-001	0.00157419	2.57409	2.57409
Rhox7-ps2	Rhox7-ps2-001	0.0149953	1.46055	1.46055
Rhox7a	Rhox7a-001	0.0478017	1.30265	1.30265
Ribc1	Ribc1-001	0.0309741	2.04305	2.04305
Ric3	Ric3-002	0.0164284	2.0647	2.0647
Ric8	Ric8-003	0.0316789	1.72312	1.72312

Ric8	Ric8-006	0.0437082	4.50971	4.50971
Ric8b	Ric8b-201	0.0301767	1.25792	1.25792
Riiad1	Riiad1-001	0.0495787	3.30371	3.30371
Rimbp3	Rimbp3-001	0.0226293	2.97536	2.97536
Rimkla	Rimkla-001	0.00263184	1.39595	1.39595
Rims2	Rims2-201	0.0453881	1.76978	1.76978
Rims3	Rims3-002	0.00120348	0.631072	-1.58461
Rint1	Rint1-007	0.0364805	2.00141	2.00141
Rita1	Rita1-001	0.0146728	1.83951	1.83951
Rmnd1	Rmnd1-005	0.00437338	2.32519	2.32519
Rnaseh2a	Rnaseh2a-005	0.0274987	0.22275	-4.48933
Rnaseh2a	Rnaseh2a-201	0.00424404	0.506007	-1.97626
Rnaseh2b	Rnaseh2b-002	0.0252254	2.55296	2.55296
Rnasek	Rnasek-001	0.0398231	0.865471	-1.15544
Rnaset2b	Rnaset2b-201	0.0248939	1.25349	1.25349
Rnd1	Rnd1-001	0.0015022	1.6384	1.6384
Rnd2	Rnd2-001	0.0356724	0.68271	-1.46475
Rnf10	Rnf10-009	0.0451241	1.30246	1.30246
Rnf11	Rnf11-002	0.0024172	1.30825	1.30825
Rnf112	Rnf112-003	0.00237885	3.33869	3.33869
Rnf113a1	Rnf113a1-001	0.0158789	1.36831	1.36831
Rnf114	Rnf114-002	0.0165607	2.38087	2.38087
Rnf121	Rnf121-002	0.00280701	0.402268	-2.48591
Rnf121	Rnf121-001	0.0263032	1.22033	1.22033
Rnf123	Rnf123-014	0.0466984	0.56909	-1.75719
Rnf13	Rnf13-001	0.0458051	1.25273	1.25273
Rnf14	Rnf14-201	0.0390011	0.843441	-1.18562
Rnf141	Rnf141-001	0.00822082	0.744053	-1.34399
Rnf141	Rnf141-003	0.040872	2.14986	2.14986
Rnf145	Rnf145-006	0.0088423	0.785201	-1.27356
Rnf157	Rnf157-001	0.0292148	1.1236	1.1236
Rnf165	Rnf165-007	0.009223	2.2888	2.2888
Rnf165	Rnf165-005	0.00767127	3.22205	3.22205
Rnf167	Rnf167-001	0.0380645	1.33554	1.33554
Rnf167	Rnf167-005	0.0428278	2.52697	2.52697
Rnf169	Rnf169-001	0.00465105	0.749596	-1.33405
Rnf180	Rnf180-201	0.0476152	0.29239	-3.42009
Rnf181	Rnf181-001	0.0122335	1.2465	1.2465
Rnf181	Rnf181-004	0.024226	5.77417	5.77417
Rnf183	Rnf183-001	0.0267928	0.228448	-4.37737
Rnf183	Rnf183-002	0.0267928	0.228448	-4.37737
Rnf185	Rnf185-001	0.000264063	1.30776	1.30776
Rnf186	Rnf186-001	0.000169048	1.94688	1.94688

Rnf19b	Rnf19b-202	0.0209459	0.616452	-1.62219
Rnf19b	Rnf19b-001	0.0275886	0.849712	-1.17687
Rnf2	Rnf2-001	0.0191833	1.14218	1.14218
Rnf20	Rnf20-002	0.0390925	3.47983	3.47983
Rnf214	Rnf214-002	0.033638	1.45662	1.45662
Rnf216	Rnf216-001	0.0262203	1.11066	1.11066
Rnf217	Rnf217-001	0.0241579	0.917358	-1.09009
Rnf25	Rnf25-009	0.043553	401.815	401.815
Rnf38	Rnf38-013	0.0449482	15.4062	15.4062
Rnf40	Rnf40-001	0.0271749	0.806357	-1.24015
Rnf40	Rnf40-003	0.0380447	1.53958	1.53958
Rnf44	Rnf44-015	0.0201316	0.529934	-1.88703
Rnf44	Rnf44-201	0.0361796	0.715148	-1.39831
Rnf5	Rnf5-001	0.00214135	0.824925	-1.21223
Rnf6	Rnf6-201	0.0127138	0.551781	-1.81231
Rnf6	Rnf6-001	0.0270117	1.34693	1.34693
Rnf7	Rnf7-001	0.00139562	1.37797	1.37797
Rnft2	Rnft2-001	0.00772269	2.62424	2.62424
Rnft2	Rnft2-006	0.00290681	3.16501	3.16501
Rnmt	Rnmt-005	0.0268612	0.216635	-4.61606
Rnmt	Rnmt-001	0.0197411	0.829118	-1.2061
Rnmt	Rnmt-002	0.0228634	1.19195	1.19195
Rnmt	Rnmt-003	0.00819575	1.79706	1.79706
Rnpc3	Rnpc3-007	0.00541589	3.02258e-05	-33084.3
Rnpc3	Rnpc3-003	0.000324409	2.80829	2.80829
Rnpepl1	Rnpepl1-005	0.0490658	1.45217	1.45217
Romo1	Romo1-003	0.0170214	1.9782	1.9782
Rorb	Rorb-005	0.0214572	0.783313	-1.27663
RP23-114E15.14	RP23-114E15.14-001	0.0263708	0.00552097	-181.127
RP23-137A24.6	RP23-137A24.6-001	0.0375217	0.344362	-2.90392
RP23-166E8.2	RP23-166E8.2-001	0.0325446	0.293234	-3.41024
RP23-178C20.2	RP23-178C20.2-002	0.0103125	0.102629	-9.74387
RP23-20L3.2	RP23-20L3.2-001	0.0368672	0.680873	-1.4687
RP23-226O20.10	RP23-226O20.10-001	0.0344025	2.72604	2.72604
RP23-287N15.3	RP23-287N15.3-001	0.0230239	1.45487	1.45487
RP23-312I2.7	RP23-312I2.7-001	0.00662692	0.0915325	-10.9251
RP23-324J22.6	RP23-324J22.6-001	0.0378347	0.399011	-2.5062
RP23-331I23.5	RP23-331I23.5-001	0.0274882	1.75652	1.75652
RP23-334M3.7	RP23-334M3.7-001	0.0149566	0.504653	-1.98156
RP23-335G1.1	RP23-335G1.1-003	0.0449112	4.80397	4.80397
RP23-335G1.5	RP23-335G1.5-001	0.0376095	0.385639	-2.5931
RP23-337M7.12	RP23-337M7.12-001	0.0180362	2.33295	2.33295
RP23-338G5.3	RP23-338G5.3-001	0.000611851	1.96574	1.96574

RP23-355N5.6	RP23-355N5.6-001	0.025553	0.502677	-1.98935
RP23-359M3.4	RP23-359M3.4-001	0.0227696	0.303217	-3.29797
RP23-367H8.4	RP23-367H8.4-002	0.0101464	0.481161	-2.07831
RP23-370A2.5	RP23-370A2.5-001	0.0172982	0.697242	-1.43422
RP23-406J21.2	RP23-406J21.2-001	0.013318	0.177149	-5.64496
RP23-42H18.6	RP23-42H18.6-001	0.0185205	1.56588	1.56588
RP23-434H14.7	RP23-434H14.7-001	0.0127845	0.535431	-1.86765
RP23-441J16.5	RP23-441J16.5-001	0.0211553	2.09764	2.09764
RP23-49L8.4	RP23-49L8.4-001	0.000477699	4.22413	4.22413
RP23-4F16.12	RP23-4F16.12-001	0.0415714	1.41801	1.41801
RP23-60P18.13	RP23-60P18.13-001	0.0112665	2.32166	2.32166
RP23-74G1.5	RP23-74G1.5-001	0.0137942	1.79023	1.79023
RP23-74N13.2	RP23-74N13.2-001	0.00921816	3.22542	3.22542
RP23-84M17.5	RP23-84M17.5-001	0.00348339	2.70421	2.70421
RP23-8I8.2	RP23-8I8.2-001	0.00746645	0.199472	-5.01325
RP24-108G5.5	RP24-108G5.5-001	0.00394875	0.135993	-7.35334
RP24-144C5.1	RP24-144C5.1-001	0.00419343	2.18065	2.18065
RP24-171J16.3	RP24-171J16.3-001	0.0108061	2.85498	2.85498
RP24-201D17.2	RP24-201D17.2-001	0.0366159	0.426339	-2.34555
RP24-204J8.6	RP24-204J8.6-001	0.045356	3.3333	3.3333
RP24-211G11.3	RP24-211G11.3-001	0.00450173	7.06977	7.06977
RP24-259P13.2	RP24-259P13.2-001	0.0457847	0.465333	-2.149
RP24-441E15.4	RP24-441E15.4-001	0.00236841	4.74108	4.74108
RP24-63G4.13	RP24-63G4.13-001	0.0279301	1.68598	1.68598
Rpa1	Rpa1-005	0.0356902	2.41654	2.41654
Rpa3	Rpa3-001	0.00698339	1.30344	1.30344
Rpap1	Rpap1-009	0.0462765	0.0203157	-49.223
Rpap1	Rpap1-001	0.0202662	1.8544	1.8544
Rpap1	Rpap1-007	0.00771648	6.73375	6.73375
Rpap2	Rpap2-001	0.00934223	1.49046	1.49046
Rpap3	Rpap3-201	0.0196519	1.45327	1.45327
Rpf1	Rpf1-002	2.94342e-06	2.318	2.318
Rpf2	Rpf2-006	0.00332921	1.9292	1.9292
Rph3al	Rph3al-002	0.0489708	0.46286	-2.16048
Rpia	Rpia-001	0.00809479	1.46524	1.46524
Rpl10	Rpl10-002	2.61005e-06	1.73203	1.73203
Rpl10a	Rpl10a-003	0.0212626	1.60712	1.60712
Rpl12	Rpl12-003	0.000432138	1.59797	1.59797
Rpl13	Rpl13-003	0.00601836	1.42824	1.42824
Rpl18	Rpl18-002	0.0441036	1.29296	1.29296
Rpl21	Rpl21-001	0.027032	1.39903	1.39903
Rpl22	Rpl22-004	0.0297742	0.544298	-1.83723
Rpl22	Rpl22-003	0.0165566	3.61598	3.61598

Rpl29	Rpl29-001	0.0230882	1.35451	1.35451
Rpl29	Rpl29-002	5.97919e-05	1.674	1.674
Rpl3-ps1	Rpl3-ps1-001	0.017359	1.61695	1.61695
Rpl35	Rpl35-003	0.00850831	1.15055	1.15055
Rpl35a	Rpl35a-003	0.043287	0.727827	-1.37395
Rpl35a	Rpl35a-004	0.0318463	1.49161	1.49161
Rpl35a	Rpl35a-002	0.0457712	2.31552	2.31552
Rpl36a	Rpl36a-003	0.00396714	1.46147	1.46147
Rpl41	Rpl41-002	0.0186053	1.36605	1.36605
Rpl41	Rpl41-007	0.0187436	1.47028	1.47028
Rpl6	Rpl6-004	0.028146	1.82129	1.82129
Rpl9	Rpl9-003	0.0198394	0.278523	-3.59037
Rpl9	Rpl9-007	0.0466811	1.30809	1.30809
Rpl9	Rpl9-010	0.0133249	1.71763	1.71763
Rpp14	Rpp14-001	0.0287717	1.47055	1.47055
Rpp21	Rpp21-005	0.0471206	1.83808	1.83808
Rpp30	Rpp30-201	0.0454226	1.33362	1.33362
Rpp38	Rpp38-001	0.0443626	1.50404	1.50404
Rprd1a	Rprd1a-201	0.0280876	1.22119	1.22119
Rprd1b	Rprd1b-006	0.0339702	15.7994	15.7994
Rprml	Rprml-001	0.00935741	0.704336	-1.41978
Rps12-ps26	Rps12-ps26-001	0.0486558	2.39425	2.39425
Rps14	Rps14-005	0.0287149	1.26707	1.26707
Rps16	Rps16-003	0.046733	1.65995	1.65995
Rps2	Rps2-001	0.000212126	1.65637	1.65637
Rps27	Rps27-009	0.0246849	4.9379	4.9379
Rps28	Rps28-003	0.00466818	2.98649	2.98649
Rps4x	Rps4x-003	0.0192103	5.14778	5.14778
Rps5	Rps5-002	0.000742329	2.2239	2.2239
Rps6ka2	Rps6ka2-201	0.00685947	0.81224	-1.23116
Rps6ka3	Rps6ka3-002	0.0138844	2.86868	2.86868
Rps6ka4	Rps6ka4-202	0.000919594	0.730558	-1.36882
Rps6kb1	Rps6kb1-001	0.0017191	0.645362	-1.54952
Rps6kb1	Rps6kb1-004	0.0460835	1.4305	1.4305
Rps6kb1	Rps6kb1-003	0.00358561	33.76	33.76
Rps9	Rps9-008	0.0104684	2.07491	2.07491
Rragb	Rragb-001	0.0105644	1.2526	1.2526
Rragd	Rragd-001	0.00385984	1.18969	1.18969
Rras2	Rras2-201	3.76555e-05	0.67565	-1.48006
Rrm2	Rrm2-002	0.0465107	0.443065	-2.257
Rrm2b	Rrm2b-001	0.00116931	0.617678	-1.61897
Rsad1	Rsad1-002	0.0429565	1.42226	1.42226
Rsbn1	Rsbn1-001	0.0479731	0.792407	-1.26198

Rsbn1	Rsbn1-003	0.0392876	2.08164	2.08164
Rsf1	Rsf1-007	0.0227413	0.451689	-2.21391
Rsf1	Rsf1-001	0.0323457	0.5415	-1.84672
Rsl24d1	Rsl24d1-001	0.0453822	0.831009	-1.20336
Rsph1	Rsph1-201	1.45918e-05	0.641588	-1.55863
Rsrc1	Rsrc1-007	0.0107875	0.308578	-3.24067
Rsrc1	Rsrc1-002	0.048482	0.681782	-1.46674
Rsrc1	Rsrc1-003	0.000990945	2.80306	2.80306
Rsrc2	Rsrc2-018	0.00787974	2.58188	2.58188
Rsu1	Rsu1-004	0.00149553	3.33889	3.33889
Rtca	Rtca-005	0.00960668	0.274117	-3.64808
Rtca	Rtca-001	0.0116106	0.78536	-1.2733
Rtel1	Rtel1-015	0.0187783	1.35161	1.35161
Rtel1	Rtel1-014	0.00709836	1.97646	1.97646
Rtkn2	Rtkn2-002	0.0277216	1.2485	1.2485
Rtn3	Rtn3-201	0.00673706	0.786756	-1.27104
Rtn3	Rtn3-203	0.0211406	1.27146	1.27146
Rtn4ip1	Rtn4ip1-001	0.0026239	1.35765	1.35765
Rufy2	Rufy2-001	0.00802156	1.15727	1.15727
Rufy2	Rufy2-006	0.0454601	32.2795	32.2795
Rufy3	Rufy3-004	0.0104564	1.77441	1.77441
Rufy3	Rufy3-005	0.0101891	2.02981	2.02981
Rundc3b	Rundc3b-002	0.00984703	1.76882	1.76882
Runx3	Runx3-001	0.0064593	0.667482	-1.49817
Rusc1	Rusc1-002	0.0159093	0.773165	-1.29338
Ruvbl2	Ruvbl2-002	0.00132752	1.28357	1.28357
Rwdd1	Rwdd1-001	0.0418724	1.08797	1.08797
Rwdd2a	Rwdd2a-003	0.0266263	1.83935	1.83935
Rwdd3	Rwdd3-002	0.0154266	2.22529	2.22529
Rwdd4a	Rwdd4a-002	0.00550051	2.40147	2.40147
Rxrb	Rxrb-009	0.0188983	2.60217	2.60217
Rybp	Rybp-001	0.000121887	0.560669	-1.78358
Rybp	Rybp-003	0.00885702	2.91623	2.91623
Ryk	Ryk-002	0.0318694	0.281646	-3.55055
S100a10	S100a10-002	0.0362255	0.546696	-1.82917
S100a10	S100a10-001	0.018801	0.587845	-1.70113
S100a13	S100a13-001	0.0341786	0.663792	-1.5065
S100a14	S100a14-003	0.0332266	18.5009	18.5009
S100a4	S100a4-001	0.00061524	0.39241	-2.54836
S100a6	S100a6-003	0.035336	0.505158	-1.97958
S100a6	S100a6-002	0.0125281	0.523503	-1.91021
S100pbp	S100pbp-010	0.00601144	0.60534	-1.65196
Saal1	Saal1-003	0.00461879	1.53165	1.53165

Safb	Safb-006	0.0413695	1.39471	1.39471
Safb	Safb-002	0.0200281	1.78744	1.78744
Safb2	Safb2-009	0.0313275	3.69052	3.69052
Samd10	Samd10-001	0.0213351	1.84189	1.84189
Samd15	Samd15-002	0.0492224	0.226507	-4.41488
Samhd1	Samhd1-201	0.00302979	5.04192	5.04192
Sapcd2	Sapcd2-001	0.0435201	0.188951	-5.29237
Sar1a	Sar1a-201	0.00548879	0.743425	-1.34513
Sarm1	Sarm1-005	0.033254	0.34941	-2.86197
Sars	Sars-002	0.00615145	1.43215	1.43215
Sars	Sars-001	9.28027e-05	1.43652	1.43652
Sars	Sars-003	0.0200875	1.61302	1.61302
Sart1	Sart1-201	0.0448269	1.14477	1.14477
Satb1	Satb1-201	0.0466807	0.383771	-2.60572
Sbf1	Sbf1-003	0.0436332	0.811932	-1.23163
Sbf1	Sbf1-007	0.0319033	1.37188	1.37188
Sbsn	Sbsn-001	0.0213082	2.43254	2.43254
Sbsn	Sbsn-006	0.0435554	14.5979	14.5979
Sc5d	Sc5d-202	0.0148184	1.37874	1.37874
Scaf1	Scaf1-003	0.000641587	0.755955	-1.32283
Scaf11	Scaf11-201	0.00850266	1.10671	1.10671
Scaf4	Scaf4-201	0.00273251	1.45646	1.45646
Scaf8	Scaf8-201	0.0349448	1.16585	1.16585
Scai	Scai-001	0.00300292	1.30416	1.30416
Scai	Scai-010	0.0215042	2.01781	2.01781
Scamp1	Scamp1-001	0.000193764	0.615679	-1.62422
Scamp4	Scamp4-201	0.00166383	0.65745	-1.52103
Scap	Scap-001	0.0196738	0.881531	-1.13439
Scap	Scap-002	0.0364021	3.23632	3.23632
Scarb1	Scarb1-001	0.012103	0.753937	-1.32637
Scarb2	Scarb2-001	0.00309614	0.850074	-1.17637
Scel	Scel-201	0.0108714	4.7254	4.7254
Scfd2	Scfd2-003	0.00667562	25.1166	25.1166
Scg2	Scg2-002	0.00102199	0.579428	-1.72584
Scg3	Scg3-201	0.000187289	0.831084	-1.20325
Schip1	Schip1-004	0.0379689	0.116207	-8.60532
Schip1	Schip1-002	0.0497991	0.125422	-7.97308
Schip1	Schip1-009	0.031139	0.55424	-1.80427
Scml2	Scml2-003	0.0234345	1.84225	1.84225
Scml4	Scml4-003	0.00348374	0.41926	-2.38515
Scml4	Scml4-005	0.0448817	2.23477	2.23477
Scn1a	Scn1a-004	0.00496297	2.70925	2.70925
Scn1a	Scn1a-002	0.00363525	12.0643	12.0643

Scn1b	Scn1b-201	0.012071	0.66453	-1.50482
Scn2a1	Scn2a1-201	0.000339043	1.88975	1.88975
Scn2a1	Scn2a1-001	0.0106331	1.9549	1.9549
Scn3b	Scn3b-002	0.0326396	1.52015	1.52015
Scn8a	Scn8a-202	0.0244358	0.709138	-1.41016
Scn8a	Scn8a-001	0.0325561	0.768543	-1.30116
Scn8a	Scn8a-012	0.00591316	6.15567	6.15567
Scnm1	Scnm1-001	0.0122988	1.58728	1.58728
Scoc	Scoc-202	0.0162346	1.40438	1.40438
Scp2	Scp2-001	0.00571889	1.44581	1.44581
Scpep1	Scpep1-001	0.021005	1.3415	1.3415
Scrib	Scrib-001	0.00211648	0.748787	-1.33549
Scrn1	Scrn1-005	0.00362645	1.59601	1.59601
Scrn3	Scrn3-001	0.00207014	1.87383	1.87383
Sct	Sct-201	0.00431136	0.337435	-2.96354
Sctr	Sctr-002	0.0164085	2.61324	2.61324
Scube3	Scube3-001	0.0013586	1.51359	1.51359
Scube3	Scube3-004	0.0248724	1.61897	1.61897
Scyl2	Scyl2-002	0.0317849	1.22148	1.22148
Sdad1	Sdad1-002	0.0491201	1.79119	1.79119
Sdc3	Sdc3-001	0.000455362	0.684675	-1.46055
Sdccag3	Sdccag3-011	0.0305176	2.25469	2.25469
Sdccag8	Sdccag8-006	0.000179772	0.283189	-3.53121
Sde2	Sde2-001	0.0450121	1.21279	1.21279
Sdf4	Sdf4-001	0.000322476	0.6703	-1.49187
Sdf4	Sdf4-002	0.000615214	1.33277	1.33277
Sdhaf3	Sdhaf3-001	0.0153568	1.24959	1.24959
Sdhc	Sdhc-001	0.0130036	1.15456	1.15456
Sdhc	Sdhc-002	0.0199642	1.24526	1.24526
Sdpr	Sdpr-001	0.00668044	0.812803	-1.23031
Sdr42e1	Sdr42e1-001	0.0140506	0.191128	-5.2321
Sdr42e1	Sdr42e1-003	0.0140506	0.191128	-5.2321
Sdsl	Sdsl-002	0.00695018	0.352686	-2.83538
Sdsl	Sdsl-001	0.00118925	0.605512	-1.65149
Sec11a	Sec11a-002	0.00916691	1.56866	1.56866
Sec11c	Sec11c-003	0.00135796	0.452926	-2.20787
Sec11c	Sec11c-001	7.4004e-05	0.587335	-1.70261
Sec16b	Sec16b-002	0.0200582	1.6776	1.6776
Sec22b	Sec22b-002	0.000921241	1.69155	1.69155
Sec22c	Sec22c-001	0.0349539	2.6116	2.6116
Sec23a	Sec23a-004	0.0124252	1.56894	1.56894
Sec23b	Sec23b-001	0.0183655	1.38688	1.38688
Sec23ip	Sec23ip-001	0.0492583	1.28767	1.28767

Sec24a	Sec24a-006	0.0167127	1.52686	1.52686
Sec24a	Sec24a-003	0.000199373	3.12129	3.12129
Sec24b	Sec24b-001	0.0268468	1.13733	1.13733
Sec24c	Sec24c-201	3.59763e-05	1.22051	1.22051
Sec24d	Sec24d-001	0.0165745	1.291	1.291
Sec31a	Sec31a-001	0.0381003	1.27125	1.27125
Sec31a	Sec31a-012	0.0179614	2.38642	2.38642
Sec61a2	Sec61a2-005	0.0473396	0.420015	-2.38087
Sec61g	Sec61g-005	0.024235	1.61525	1.61525
Sec63	Sec63-001	0.00326137	0.753763	-1.32668
Sec63	Sec63-004	0.0156335	1.20955	1.20955
Sec63	Sec63-002	0.00652008	4.82649	4.82649
Sectm1a	Sectm1a-001	0.0214785	1.56611	1.56611
Sel1l	Sel1l-006	0.0466711	1.32461	1.32461
Sel1l	Sel1l-001	0.000667687	1.72448	1.72448
Sel1l	Sel1l-004	0.0141667	2.45742	2.45742
Selo	Selo-001	0.0191657	1.3968	1.3968
Sema3f	Sema3f-001	0.0238669	0.5013	-1.99481
Sema3g	Sema3g-001	0.000103648	2.74589	2.74589
Sema4b	Sema4b-004	0.0271286	0.820579	-1.21865
Sema4c	Sema4c-005	0.0289582	0.0388706	-25.7264
Sema4c	Sema4c-002	0.00857461	0.677284	-1.47648
Sema4d	Sema4d-006	0.006946	0.00727898	-137.382
Sema5b	Sema5b-001	0.0499782	0.34462	-2.90175
Sema6b	Sema6b-201	0.0113978	0.305113	-3.27748
Sema6c	Sema6c-201	0.0146904	0.00789755	-126.622
Sema6d	Sema6d-008	0.000718166	0.311887	-3.20629
Sema6d	Sema6d-201	0.00300455	0.343112	-2.9145
Senp1	Senp1-007	0.0200635	0.00749534	-133.416
Senp1	Senp1-001	0.0371172	0.691815	-1.44547
Senp5	Senp5-004	0.0107634	14.1788	14.1788
Senp6	Senp6-013	0.0241869	0.0648657	-15.4165
Senp6	Senp6-006	0.00975194	1.36066	1.36066
Senp7	Senp7-010	0.0203397	0.406744	-2.45855
Senp7	Senp7-004	0.0181262	0.489783	-2.04172
Sephs1	Sephs1-001	0.0452225	0.572633	-1.74632
Sepn1	Sepn1-001	0.0265347	0.761232	-1.31366
Sepsecs	Sepsecs-001	0.00161769	0.595715	-1.67866
06-Sep	Sept6-003	0.0316827	0.553148	-1.80783
09-Sep	Sept9-002	0.0213299	0.673428	-1.48494
10-Sep	Sept10-201	0.00176807	0.646084	-1.54779
Serac1	Serac1-001	0.0396487	1.2552	1.2552
Serbp1	Serbp1-004	0.0264568	1.22603	1.22603

Serbp1	Serbp1-011	0.0113745	1.23566	1.23566
Serinc2	Serinc2-005	0.0132758	2.88558	2.88558
Serinc5	Serinc5-201	0.0255679	0.783329	-1.2766
Serpinb6b	Serpinb6b-002	0.0359269	0.000753321	-1327.46
Serpinb6b	Serpinb6b-001	0.0359269	0.000753321	-1327.45
Serpinc1	Serpinc1-001	0.0336029	0.315151	-3.17308
Serpinf1	Serpinf1-004	0.0379067	2.50466e-08	-3.99257e+07
Serpinf1	Serpinf1-002	0.0374244	0.115894	-8.6286
Serpinf1	Serpinf1-007	0.000859417	0.428707	-2.33259
Serpinf1	Serpinf1-008	0.0117968	0.448431	-2.23
Serpinh1	Serpinh1-201	0.00162005	2.44397	2.44397
Sesn2	Sesn2-001	0.00935682	1.66222	1.66222
Set	Set-005	1.51831e-05	1.75439	1.75439
Setd3	Setd3-004	0.0108146	0.57569	-1.73704
Setd3	Setd3-001	9.84673e-05	0.842223	-1.18733
Setd4	Setd4-003	0.0283808	1.52008	1.52008
Setd4	Setd4-201	0.00580823	2.11916	2.11916
Sez6	Sez6-001	0.00225003	0.479861	-2.08394
Sez6	Sez6-004	0.0442879	4.84194	4.84194
Sez6l2	Sez6l2-003	0.00207199	0.584398	-1.71116
Sez6l2	Sez6l2-002	0.00639063	0.610858	-1.63704
Sez6l2	Sez6l2-001	0.00555387	0.734645	-1.3612
Sf1	Sf1-001	0.00416933	1.36867	1.36867
Sfi1	Sfi1-011	0.0256776	1.25558	1.25558
Sft2d1	Sft2d1-009	0.0290247	3.76451	3.76451
Sft2d2	Sft2d2-001	0.0046639	0.756067	-1.32263
Sft2d2	Sft2d2-004	0.00296254	6.03594	6.03594
Sfxn3	Sfxn3-202	0.0277947	0.354866	-2.81797
Sfxn5	Sfxn5-009	0.00684312	4.06776	4.06776
Sgcb	Sgcb-001	0.0184669	1.34449	1.34449
Sgce	Sgce-007	0.0161154	1.7709	1.7709
Sgip1	Sgip1-007	0.0303372	0.490444	-2.03897
Sgk3	Sgk3-010	0.0365916	4.14618e-07	-2.41186e+06
Sgms1	Sgms1-004	0.027207	0.560003	-1.7857
Sgms1	Sgms1-003	0.0289972	6.14101	6.14101
Sgol1	Sgol1-003	0.0331204	2.5121e-05	-39807.3
Sgpl1	Sgpl1-002	0.00228642	0.625474	-1.59879
Sgpl1	Sgpl1-001	0.023289	0.696595	-1.43555
Sgsm1	Sgsm1-002	0.038114	9.73124	9.73124
Sgsm3	Sgsm3-001	0.0205564	1.34759	1.34759
Sgta	Sgta-201	0.0479374	1.20519	1.20519
Sh2b1	Sh2b1-007	0.043077	1.95174	1.95174
Sh2b1	Sh2b1-006	0.0450403	2.66035	2.66035

Sh3bgrl	Sh3bgrl-001	0.00633512	0.862105	-1.15995
Sh3bgrl3	Sh3bgrl3-001	0.0164902	0.859427	-1.16357
Sh3bp1	Sh3bp1-201	0.000719516	1.27079	1.27079
Sh3bp2	Sh3bp2-006	0.00315922	0.462103	-2.16402
Sh3bp5	Sh3bp5-002	0.0190029	7.61462	7.61462
Sh3gl3	Sh3gl3-001	0.0265771	0.558545	-1.79037
Sh3glb1	Sh3glb1-003	2.88515e-05	0.433798	-2.30522
Sh3kbp1	Sh3kbp1-007	0.0185863	0.414782	-2.4109
Sh3kbp1	Sh3kbp1-004	0.0387378	0.663892	-1.50627
Sh3kbp1	Sh3kbp1-002	0.00972372	0.743633	-1.34475
Sh3kbp1	Sh3kbp1-001	0.030827	0.754581	-1.32524
Sh3pxd2a	Sh3pxd2a-202	0.00816011	0.530331	-1.88562
Sh3pxd2a	Sh3pxd2a-201	0.00837741	0.691131	-1.4469
Sh3tc1	Sh3tc1-001	0.0330294	0.0014338	-697.449
Sh3tc1	Sh3tc1-004	0.0141971	0.153697	-6.5063
Shc1	Shc1-004	0.0145725	0.697497	-1.4337
Shc3	Shc3-201	0.0446113	0.632252	-1.58165
Shcbp1l	Shcbp1l-001	0.0298009	1.58591	1.58591
She	She-001	0.0375017	0.532087	-1.87939
Shf	Shf-007	0.0293529	0.271926	-3.67747
Shfm1	Shfm1-001	0.0038438	1.08136	1.08136
Shisa2	Shisa2-201	0.00801586	1.69882	1.69882
Shisa4	Shisa4-001	0.0430584	0.615783	-1.62395
Shisa7	Shisa7-001	0.0279441	0.583378	-1.71415
Shisa8	Shisa8-201	0.0112901	0.255186	-3.91872
Shkbp1	Shkbp1-003	0.0455463	0.541282	-1.84746
Shkbp1	Shkbp1-001	0.00156433	0.546157	-1.83098
Shmt2	Shmt2-201	0.0152057	1.16607	1.16607
Shoc2	Shoc2-201	0.0165024	0.847609	-1.17979
Shpk	Shpk-002	0.0178325	2.14699	2.14699
Shprh	Shprh-002	0.0456627	1.95235	1.95235
Shprh	Shprh-007	0.0165838	5.29479	5.29479
Shtn1	Shtn1-201	0.00117608	0.774807	-1.29064
Siah1a	Siah1a-201	0.00309371	1.231	1.231
Sidt2	Sidt2-002	0.0302599	0.705797	-1.41684
Sik1	Sik1-201	0.0259448	0.799302	-1.25109
Sik2	Sik2-003	0.0260282	0.540129	-1.85141
Sik3	Sik3-002	0.00117096	1.91067	1.91067
Sin3b	Sin3b-202	0.00818318	1.29608	1.29608
Sipa1	Sipa1-203	0.0272011	0.660088	-1.51495
Sipa1l2	Sipa1l2-201	0.0359557	0.850853	-1.17529
Sipa1l3	Sipa1l3-002	0.0451835	0.873293	-1.14509
Sipa1l3	Sipa1l3-201	0.0451835	0.873293	-1.14509

Sirt1	Sirt1-002	0.0299674	0.573913	-1.74243
Sirt1	Sirt1-001	0.0124337	2.41148	2.41148
Sirt1	Sirt1-201	0.0124345	2.41156	2.41156
Sirt2	Sirt2-006	0.0189891	8.89667	8.89667
Sirt3	Sirt3-002	0.00392127	1.60014	1.60014
Sirt4	Sirt4-003	0.0346172	2.17656	2.17656
Sirt6	Sirt6-002	0.0396001	1.55005	1.55005
Sirt7	Sirt7-008	0.033861	0.301199	-3.32007
Sirt7	Sirt7-006	0.00147165	0.529118	-1.88994
Six3os1	Six3os1-002	0.0202115	1.73279	1.73279
Six4	Six4-001	0.00673152	0.89721	-1.11457
Six5	Six5-001	0.0156847	0.769597	-1.29938
Ska2	Ska2-002	0.0103553	1.78618	1.78618
Ska3	Ska3-201	0.0311212	1.14184	1.14184
Skil	Skil-002	0.00406015	2.7499	2.7499
Skiv2l	Skiv2l-002	0.0035973	0.460374	-2.17215
Skiv2l	Skiv2l-001	0.0106478	0.633557	-1.57839
Skiv2l	Skiv2l-004	0.0127827	1.56621	1.56621
Skp1a	Skp1a-003	0.0109805	0.135441	-7.38327
Slamf8	Slamf8-001	0.0230493	18.5196	18.5196
Slbp	Slbp-003	0.024502	7.82466	7.82466
Slc10a4	Slc10a4-002	0.0378124	1.64387	1.64387
Slc10a4	Slc10a4-001	0.0064725	1.67095	1.67095
Slc11a1	Slc11a1-010	0.0238847	0.0384643	-25.9981
Slc12a5	Slc12a5-004	0.00147622	0.415803	-2.40499
Slc12a5	Slc12a5-001	0.0164446	0.621661	-1.60859
Slc12a6	Slc12a6-006	0.0364035	0.665714	-1.50215
Slc12a6	Slc12a6-001	0.00598367	0.757686	-1.31981
Slc12a7	Slc12a7-201	0.000104111	0.650327	-1.53769
Slc12a8	Slc12a8-004	0.0433362	27.4704	27.4704
Slc12a9	Slc12a9-001	0.00314437	0.800731	-1.24886
Slc15a4	Slc15a4-005	0.040109	0.0147966	-67.583
Slc15a5	Slc15a5-004	0.00240363	2.9071	2.9071
Slc16a14	Slc16a14-001	0.00219413	2.09844	2.09844
Slc16a14	Slc16a14-002	0.0204205	186.467	186.467
Slc16a3	Slc16a3-201	0.0317316	1.30361	1.30361
Slc16a4	Slc16a4-001	0.0219353	0.647454	-1.54451
Slc16a5	Slc16a5-005	0.0318848	2.47498	2.47498
Slc16a6	Slc16a6-001	0.00200519	0.608352	-1.64379
Slc16a6	Slc16a6-004	0.0271735	1.7693	1.7693
Slc17a9	Slc17a9-002	0.0226797	1.96448	1.96448
Slc17a9	Slc17a9-001	0.0339603	2.31764	2.31764
Slc1a4	Slc1a4-201	0.00646698	1.61723	1.61723

Slc1a7	Slc1a7-001	0.00984854	3.38257	3.38257
Slc22a18	Slc22a18-001	0.00581294	0.187675	-5.32835
Slc22a7	Slc22a7-201	0.0127146	1.73968	1.73968
Slc24a2	Slc24a2-006	9.23095e-05	8.90553	8.90553
Slc24a3	Slc24a3-001	0.0432861	0.021367	-46.8011
Slc25a1	Slc25a1-006	0.0269233	0.67981	-1.471
Slc25a11	Slc25a11-001	0.0425642	0.77097	-1.29707
Slc25a20	Slc25a20-001	0.0195869	0.752259	-1.32933
Slc25a23	Slc25a23-002	0.0120193	1.542	1.542
Slc25a24	Slc25a24-001	1.5047e-07	0.698913	-1.43079
Slc25a25	Slc25a25-008	0.0359505	0.0007185	-1391.79
Slc25a25	Slc25a25-002	0.03393	2.59427	2.59427
Slc25a33	Slc25a33-001	0.0284432	1.30577	1.30577
Slc25a37	Slc25a37-001	0.0102229	0.131186	-7.62278
Slc25a37	Slc25a37-003	0.0191002	1.91267	1.91267
Slc25a39	Slc25a39-008	0.0272944	0.000564104	-1772.72
Slc25a39	Slc25a39-002	0.0134447	1.39986	1.39986
Slc25a39	Slc25a39-010	0.0420646	15.0178	15.0178
Slc25a40	Slc25a40-003	0.025846	0.349783	-2.85891
Slc25a40	Slc25a40-002	0.0118021	1.7262	1.7262
Slc25a51	Slc25a51-201	0.0177051	0.841406	-1.18849
Slc25a51	Slc25a51-001	0.0177226	0.84143	-1.18845
Slc25a51	Slc25a51-003	6.62705e-05	1.47986	1.47986
Slc25a53	Slc25a53-004	0.0273269	3.60146	3.60146
Slc26a11	Slc26a11-001	0.00597946	1.41796	1.41796
Slc26a11	Slc26a11-004	0.00142247	4.01523	4.01523
Slc29a1	Slc29a1-015	0.0320127	5.02662e-06	-198941
Slc29a1	Slc29a1-024	0.0336218	0.483134	-2.06982
Slc29a1	Slc29a1-003	0.0204344	0.682503	-1.4652
Slc2a6	Slc2a6-001	0.0459455	0.583059	-1.71509
Slc2a6	Slc2a6-003	0.00139213	2.71951	2.71951
Slc2a8	Slc2a8-001	0.0149193	0.727202	-1.37513
Slc30a1	Slc30a1-002	0.0110469	237.798	237.798
Slc30a5	Slc30a5-201	0.0180217	1.32215	1.32215
Slc30a9	Slc30a9-006	0.0219662	0.912414	-1.09599
Slc30a9	Slc30a9-005	0.00731947	1.55594	1.55594
Slc35a3	Slc35a3-002	0.0383979	2.00756	2.00756
Slc35a5	Slc35a5-006	0.00847915	0.392178	-2.54986
Slc35a5	Slc35a5-002	0.034245	10.0065	10.0065
Slc35b4	Slc35b4-002	0.0326528	1.47323	1.47323
Slc35c2	Slc35c2-014	0.012023	1.63367	1.63367
Slc35c2	Slc35c2-001	0.00034954	9.43847	9.43847
Slc35e2	Slc35e2-003	0.0373079	1.33523	1.33523

Slc35e3	Slc35e3-201	0.0421818	1.23081	1.23081
Slc35e4	Slc35e4-201	0.0333636	0.766899	-1.30395
Slc37a1	Slc37a1-202	0.0205517	0.489809	-2.04161
Slc37a2	Slc37a2-004	0.0319185	7.23444	7.23444
Slc37a3	Slc37a3-003	0.0237139	10.0859	10.0859
Slc38a1	Slc38a1-203	0.0229894	1.31272	1.31272
Slc38a10	Slc38a10-012	0.00805726	0.448676	-2.22878
Slc38a10	Slc38a10-001	0.00209542	0.623273	-1.60443
Slc38a10	Slc38a10-201	0.000115527	0.675356	-1.4807
Slc38a10	Slc38a10-004	0.0292983	0.782182	-1.27847
Slc38a6	Slc38a6-001	0.0215907	1.15119	1.15119
Slc38a7	Slc38a7-001	0.000261074	1.36959	1.36959
Slc38a7	Slc38a7-002	3.43842e-05	1.75022	1.75022
Slc39a1	Slc39a1-001	0.00205786	0.846709	-1.18104
Slc39a12	Slc39a12-002	0.0416013	0.417712	-2.39399
Slc39a13	Slc39a13-002	0.0376496	1.86434	1.86434
Slc39a13	Slc39a13-012	0.0236631	6.76812	6.76812
Slc39a14	Slc39a14-001	0.0118605	1.43508	1.43508
Slc3a2	Slc3a2-009	0.0280861	0.0461903	-21.6496
Slc3a2	Slc3a2-002	0.000591014	0.853195	-1.17207
Slc41a1	Slc41a1-001	0.0112574	1.35405	1.35405
Slc41a2	Slc41a2-001	0.00489493	0.819801	-1.21981
Slc43a2	Slc43a2-001	0.0252506	0.337177	-2.9658
Slc44a1	Slc44a1-006	0.0171215	0.791884	-1.26281
Slc44a2	Slc44a2-201	0.00995228	0.660051	-1.51503
Slc45a4	Slc45a4-001	0.00162275	0.290242	-3.4454
Slc45a4	Slc45a4-201	0.0478441	0.349987	-2.85725
Slc48a1	Slc48a1-001	0.00274972	1.45528	1.45528
Slc4a1ap	Slc4a1ap-003	0.042604	0.144362	-6.92705
Slc4a1ap	Slc4a1ap-201	0.00529055	1.90769	1.90769
Slc4a2	Slc4a2-002	0.00882881	0.341713	-2.92644
Slc4a3	Slc4a3-001	0.0456365	0.317868	-3.14596
Slc4a8	Slc4a8-001	0.0341677	0.690233	-1.44879
Slc6a15	Slc6a15-202	0.00767021	1.21502	1.21502
Slc6a17	Slc6a17-003	0.0383667	0.215325	-4.64414
Slc6a17	Slc6a17-010	0.0310229	3.57187e+08	3.57187e+08
Slc6a6	Slc6a6-001	0.0066876	0.736197	-1.35833
Slc6a8	Slc6a8-015	0.0332014	0.00200838	-497.913
Slc6a8	Slc6a8-014	0.0261982	2.739e+08	2.739e+08
Slc7a3	Slc7a3-201	0.0104236	1.68426	1.68426
Slc7a3	Slc7a3-003	0.00665556	2.41414	2.41414
Slc7a5	Slc7a5-001	0.0151691	0.765761	-1.30589
Slc8a1	Slc8a1-201	0.039901	0.688589	-1.45224

Slc8a2	Slc8a2-201	0.00105156	0.659002	-1.51745
Slc8b1	Slc8b1-001	0.0100635	0.506313	-1.97506
Slc9a7	Slc9a7-002	0.0345408	0.000330554	-3025.22
Slco1a6	Slco1a6-001	0.0346449	2.26338	2.26338
Slco3a1	Slco3a1-006	0.035664	0.220881	-4.52733
Slco3a1	Slco3a1-002	0.000746462	0.672693	-1.48656
Slfn5	Slfn5-001	0.0241311	0.592341	-1.68822
Slit2	Slit2-001	0.0188646	0.455493	-2.19542
Slit2	Slit2-007	0.00751037	0.509905	-1.96115
Slit2	Slit2-012	0.0226431	2.25003	2.25003
Slit2	Slit2-021	0.000180358	2.69539	2.69539
Slit2	Slit2-003	0.0082263	3.01014	3.01014
Slitrk3	Slitrk3-001	0.0411895	0.190912	-5.23802
Slk	Slk-001	5.23506e-07	0.622917	-1.60535
Slk	Slk-002	0.0167191	1.48697	1.48697
Slu7	Slu7-002	0.00760179	1.36397	1.36397
Slx1b	Slx1b-003	0.0387002	0.0394494	-25.3489
Slx4	Slx4-004	0.0455385	3.80351	3.80351
Smad4	Smad4-001	0.00107281	0.871678	-1.14721
Smad5	Smad5-005	0.0305782	0.0511966	-19.5325
Smad5	Smad5-001	1.83659e-07	0.581016	-1.72112
Smad5	Smad5-004	0.0226589	7.66765	7.66765
Smad7	Smad7-003	0.0415941	8.76567	8.76567
Smad9	Smad9-001	0.0458861	1.66987	1.66987
Smap1	Smap1-001	0.0463501	1.1821	1.1821
Smap2	Smap2-001	0.0149819	1.18085	1.18085
Smarca2	Smarca2-006	0.0285884	0.805338	-1.24172
Smarcad1	Smarcad1-005	0.0447868	1.89229	1.89229
Smarcad1	Smarcad1-004	0.018037	3.82312	3.82312
Smarcal1	Smarcal1-010	0.042957	3.77868	3.77868
Smarcb1	Smarcb1-004	0.010757	1.45716	1.45716
Smarcc1	Smarcc1-002	0.0175915	1.80535	1.80535
Smarcd2	Smarcd2-001	0.0155352	0.755905	-1.32292
Smc3	Smc3-001	0.0165472	1.30644	1.30644
Smc3	Smc3-002	0.00537202	1.73875	1.73875
Smc4	Smc4-001	0.00308245	0.790507	-1.26501
Smco4	Smco4-202	0.0135698	1.55186	1.55186
Smcr8	Smcr8-201	0.0166539	1.30602	1.30602
Smcr8	Smcr8-001	0.0467976	1.53411	1.53411
Smek2	Smek2-003	0.00263247	1.253	1.253
Smek2	Smek2-001	0.0120287	7.16802	7.16802
Smg1	Smg1-001	0.000551708	0.704047	-1.42036
Smg1	Smg1-005	0.00210987	3.89022	3.89022

Smg5	Smg5-002	0.0246823	1.4687	1.4687
Smg6	Smg6-001	0.0149266	1.15971	1.15971
Smg6	Smg6-005	0.0204424	1.9104	1.9104
Smim10l1	Smim10l1-002	0.021709	1.32129	1.32129
Smim17	Smim17-001	0.0467026	2.59535	2.59535
Smim17	Smim17-003	0.0401796	228137	228137
Smim24	Smim24-002	0.011091	0.0742042	-13.4763
Smim24	Smim24-001	0.0308991	3.91641	3.91641
Smim4	Smim4-201	0.0395298	2.16113	2.16113
Smndc1	Smndc1-201	0.0011265	1.42008	1.42008
Smpd4	Smpd4-022	0.0496227	1.67512	1.67512
Smpd4	Smpd4-012	0.0423583	2.78889	2.78889
Sms	Sms-001	0.000289744	0.580334	-1.72315
Sms	Sms-005	0.00186583	0.815483	-1.22627
Smtn	Smtn-009	0.0074167	1.80645	1.80645
Smu1	Smu1-001	0.0465875	1.21499	1.21499
Smyd2	Smyd2-001	0.0448522	0.850758	-1.17542
Smyd2	Smyd2-002	0.000197603	1.91102	1.91102
Smyd3	Smyd3-002	0.00378276	1.98602	1.98602
Smyd3	Smyd3-003	0.0202515	2.13232	2.13232
Smyd3	Smyd3-004	0.000286685	2.31573	2.31573
Smyd3	Smyd3-007	0.0102558	3.21042	3.21042
Snap23	Snap23-002	0.0417661	1.55872	1.55872
Snap47	Snap47-001	0.00729164	1.27864	1.27864
Snap91	Snap91-008	0.0178469	2.10043	2.10043
Snapc1	Snapc1-201	0.0131715	1.2179	1.2179
Snapc4	Snapc4-011	0.0145977	0.473717	-2.11097
Sncg	Sncg-001	0.0413924	0.513756	-1.94645
Snd1	Snd1-002	0.0389968	1.45106	1.45106
Snd1	Snd1-012	0.00256539	2.18985	2.18985
Snhg1	Snhg1-007	0.0149617	3.92142	3.92142
Snhg14	Snhg14-002	0.0354128	0.697691	-1.4333
Snhg17	Snhg17-003	0.00893039	0.876292	-1.14117
Snhg20	Snhg20-001	0.0180516	1.72846	1.72846
Snhg3	Snhg3-002	0.00719638	5.63661	5.63661
Snhg5	Snhg5-001	0.0428757	1.15045	1.15045
Snhg6	Snhg6-004	0.0155051	0.801046	-1.24837
Snhg7os	Snhg7os-001	0.0406955	3.15255	3.15255
Snora17	Snora17-201	0.0383839	2.3487	2.3487
Snora2b	Snora2b-201	0.0178118	2.82497	2.82497
Snora43	Snora43-201	0.0368566	9.62124	9.62124
SNORA50	SNORA50.1-201	0.002067	5.69789	5.69789
Snord100	Snord100-201	0.00315243	0.329785	-3.03228

Snord16a	Snord16a-201	0.0221093	1.82811	1.82811
Snrk	Snrk-001	0.0185362	0.833886	-1.1992
Snrnp200	Snrnp200-001	0.00127359	1.43757	1.43757
Snrnp25	Snrnp25-001	0.0202369	1.4241	1.4241
Snrnp27	Snrnp27-001	0.00418255	1.21655	1.21655
Snrnp48	Snrnp48-201	0.0369737	1.71293	1.71293
Snrnp70	Snrnp70-004	0.000285949	0.441719	-2.26388
Snrnp70	Snrnp70-001	0.00146986	1.49028	1.49028
Snrnp70	Snrnp70-013	0.017675	1.61124	1.61124
Snrnp70	Snrnp70-010	0.0195321	1.98218	1.98218
Snrpa	Snrpa-006	0.00533663	0.367499	-2.72109
Snrpa	Snrpa-001	0.0345011	1.34924	1.34924
Snrpa	Snrpa-201	0.00396874	1.39506	1.39506
Snrpa1	Snrpa1-003	0.0288027	0.616279	-1.62264
Snrpa1	Snrpa1-002	0.0124302	2.03296	2.03296
Snrpa1	Snrpa1-001	0.00130948	2.10801	2.10801
Snrpb	Snrpb-001	0.0331364	0.635002	-1.5748
Snrpe	Snrpe-004	0.0286344	2.49099	2.49099
Snrpn	Snrpn-001	0.00086168	1.31862	1.31862
Snx1	Snx1-002	0.0261204	2.00416	2.00416
Snx12	Snx12-003	0.0170152	2.11084	2.11084
Snx16	Snx16-001	0.00138855	1.48827	1.48827
Snx18	Snx18-201	0.00867675	0.850038	-1.17642
Snx21	Snx21-005	0.0474095	1.34668	1.34668
Snx29	Snx29-005	0.0424117	1.74726	1.74726
Snx29	Snx29-003	0.00746239	13.5274	13.5274
Snx3	Snx3-003	0.0462954	1.19195	1.19195
Snx30	Snx30-001	0.0201159	0.806771	-1.23951
Snx33	Snx33-201	0.00550894	1.42897	1.42897
Snx5	Snx5-004	0.0394343	0.62606	-1.59729
Snx6	Snx6-201	0.00070379	1.23168	1.23168
Snx8	Snx8-002	0.0152151	1.82567	1.82567
Soat1	Soat1-001	0.0140821	0.711382	-1.40571
Sobp	Sobp-001	0.000690359	0.793112	-1.26086
Socs2	Socs2-001	0.000666317	0.637927	-1.56758
Socs4	Socs4-001	0.0318246	0.842165	-1.18742
Soga1	Soga1-001	0.0159698	0.685561	-1.45866
Soga3	Soga3-201	0.00664097	0.845101	-1.18329
Sohlh2	Sohlh2-001	0.000668874	1.19497	1.19497
Sorbs3	Sorbs3-201	0.00576164	0.544757	-1.83568
Sord	Sord-001	0.000358455	1.22775	1.22775
Sort1	Sort1-004	0.0278227	0.641816	-1.55808
Sos1	Sos1-201	0.00173021	0.798793	-1.25189

Sos2	Sos2-001	0.0207127	0.905618	-1.10422
Sox18	Sox18-001	0.0429367	2.18307	2.18307
Sox4	Sox4-001	0.0272564	0.752752	-1.32846
Sox5	Sox5-001	0.0413559	1.71098	1.71098
Sox5	Sox5-201	0.0413559	1.71098	1.71098
Sox7	Sox7-201	0.0107952	0.37007	-2.70219
Sox8	Sox8-001	3.16745e-05	0.66504	-1.50367
Sp1	Sp1-005	0.00227088	2.98202	2.98202
Sp6	Sp6-001	0.0189086	0.541751	-1.84587
Spag4	Spag4-002	0.0137944	5.07796	5.07796
Spag5	Spag5-001	0.000844301	0.884025	-1.13119
Spag5	Spag5-003	0.014926	2.56945	2.56945
Spag5	Spag5-005	0.00986286	2.57573	2.57573
Spag6l	Spag6l-005	0.0145858	8.44273	8.44273
Spag9	Spag9-201	0.046924	0.651433	-1.53508
Spag9	Spag9-008	0.00802466	0.686599	-1.45645
Sparc	Sparc-002	0.00882547	0.530465	-1.88514
Sparc	Sparc-001	0.00539554	0.574196	-1.74157
Spata2	Spata2-001	0.00181944	1.74384	1.74384
Spata33	Spata33-201	0.0235984	2.91242	2.91242
Spata5	Spata5-002	0.0326039	1.28843	1.28843
Spata5l1	Spata5l1-002	0.00755095	3.5537	3.5537
Spata7	Spata7-001	0.0225644	1.48211	1.48211
Spats2	Spats2-201	0.00304194	1.26323	1.26323
Spc25	Spc25-003	0.0255076	0.543061	-1.84142
Spcs2	Spcs2-201	0.00608858	1.28627	1.28627
Spcs3	Spcs3-001	0.0381308	1.11984	1.11984
Specc1	Specc1-008	0.00658017	2.00773e-05	-49807.5
Specc1l	Specc1l-201	0.00575835	0.842709	-1.18665
Speg	Speg-011	0.0322598	0.252481	-3.9607
Speg	Speg-012	0.022366	0.308543	-3.24104
Speg	Speg-003	0.0228884	0.830228	-1.20449
Speg	Speg-015	0.02835	1.90061	1.90061
Spg11	Spg11-001	0.00525202	1.20241	1.20241
Spg11	Spg11-002	0.00586066	4.8488	4.8488
Spg20	Spg20-008	0.0399771	1.50895	1.50895
Spg7	Spg7-008	0.000487418	0.0559057	-17.8873
Spg7	Spg7-002	0.00164267	0.434677	-2.30056
Spg7	Spg7-003	0.0292114	0.652192	-1.53329
Spice1	Spice1-003	0.0257461	1.31691	1.31691
Spint2	Spint2-201	0.0344603	0.407498	-2.454
Spn	Spn-001	0.00894736	0.249045	-4.01534
Spns1	Spns1-002	0.0460739	1.19051	1.19051

Spns3	Spns3-001	0.0300163	5.91893	5.91893
Spns3	Spns3-002	0.0300163	5.91893	5.91893
Spopl	Spopl-001	0.00445841	0.764221	-1.30852
Spopl	Spopl-005	0.0205064	1.77543	1.77543
Sppl2a	Sppl2a-001	0.0407936	1.09885	1.09885
Sppl2a	Sppl2a-003	0.00533313	1.85516	1.85516
Sppl3	Sppl3-001	0.00407176	0.865301	-1.15567
Spred1	Spred1-001	0.00060027	0.635567	-1.5734
Spryd3	Spryd3-001	0.00395841	1.31977	1.31977
Spryd7	Spryd7-002	0.0370323	1.14476	1.14476
Spsb2	Spsb2-002	0.0188083	0.542553	-1.84314
Sptan1	Sptan1-012	0.00139051	0.229674	-4.35399
Sptan1	Sptan1-003	0.00460481	0.770812	-1.29733
Sptan1	Sptan1-001	0.020811	0.806246	-1.24032
Sptan1	Sptan1-201	0.0294684	0.82322	-1.21474
Sptb	Sptb-002	0.0129453	11.518	11.518
Sptbn1	Sptbn1-004	0.0122367	0.251396	-3.97779
Sptbn1	Sptbn1-006	0.0055781	0.321278	-3.11256
Sptbn1	Sptbn1-201	0.000456012	0.631058	-1.58464
Sptbn1	Sptbn1-001	0.0193928	0.788469	-1.26828
Sptbn5	Sptbn5-001	0.012843	6.5526	6.5526
Sptlc2	Sptlc2-001	0.0420238	0.635205	-1.57429
Sptlc2	Sptlc2-005	0.0376223	1.21888	1.21888
Sptssa	Sptssa-201	0.00126899	0.750968	-1.33161
Sqstm1	Sqstm1-001	0.0471677	1.53267	1.53267
Sra1	Sra1-005	0.00178018	1.48589	1.48589
Src	Src-201	0.0155865	0.527734	-1.89489
Srcap	Srcap-201	0.0305157	3.40572	3.40572
Srfbp1	Srfbp1-201	0.0457338	1.18431	1.18431
Srgap2	Srgap2-001	0.00818112	0.75171	-1.3303
Srgap3	Srgap3-004	0.00298998	0.348982	-2.86548
Srgap3	Srgap3-001	0.0240343	0.86357	-1.15798
Srgap3	Srgap3-003	0.0264512	2.93464	2.93464
Sri	Sri-006	1.35227e-05	1.75596	1.75596
Srl	Srl-002	0.019182	1.74105	1.74105
Srl	Srl-004	0.0370192	5.31791	5.31791
Srp54a	Srp54a-201	0.0416907	1.25951	1.25951
Srp54b	Srp54b-201	0.00878333	1.19683	1.19683
Srp54b	Srp54b-202	0.0210092	1.30244	1.30244
Srp68	Srp68-001	0.000930626	1.24696	1.24696
Srp72	Srp72-001	0.0226573	0.885412	-1.12942
Srp72	Srp72-008	0.00161207	2.08702	2.08702
Srp72	Srp72-003	0.0119367	2.39697	2.39697

Srpk1	Srpk1-004	0.0324442	0.806476	-1.23996
Srrm1	Srrm1-003	0.00623078	0.886178	-1.12844
Srrm1	Srrm1-002	0.0172711	2.29309	2.29309
Srrm2	Srrm2-004	0.0434358	0.799041	-1.2515
Srsf1	Srsf1-004	0.00449782	1.3008	1.3008
Srsf10	Srsf10-002	0.00129468	0.584267	-1.71154
Srsf10	Srsf10-001	0.00725717	1.28477	1.28477
Srsf10	Srsf10-007	0.0161997	1.72406	1.72406
Srsf10	Srsf10-008	0.00758511	2.06368	2.06368
Srsf10	Srsf10-006	0.0156672	3.17709	3.17709
Srsf11	Srsf11-001	0.0240016	0.612167	-1.63354
Srsf12	Srsf12-002	0.00270282	31.2463	31.2463
Srsf2	Srsf2-004	0.0450095	0.8995	-1.11173
Srsf3	Srsf3-003	0.0226055	0.728553	-1.37258
Srsf4	Srsf4-001	0.0162462	1.12651	1.12651
Srsf5	Srsf5-006	0.000525186	1.37991	1.37991
Srxn1	Srxn1-001	0.0457847	1.466	1.466
Ssbp1	Ssbp1-004	0.0461478	1.60478	1.60478
Ssbp1	Ssbp1-002	0.017902	1.6589	1.6589
Ssbp2	Ssbp2-001	0.0190435	0.703523	-1.42142
Ssbp3	Ssbp3-016	0.0293788	0.308163	-3.24503
Ssbp3	Ssbp3-002	0.00838499	1.78791	1.78791
Ssbp4	Ssbp4-201	0.0106003	0.725387	-1.37857
Ssc4d	Ssc4d-003	0.0408674	1.94157	1.94157
Ssfa2	Ssfa2-003	0.0467585	0.59378	-1.68412
Ssh1	Ssh1-001	0.0321869	0.56943	-1.75614
Ssh2	Ssh2-001	0.0311076	0.734143	-1.36213
Ssh3	Ssh3-201	0.0168045	0.444597	-2.24923
Ssr2	Ssr2-001	0.0400165	1.33052	1.33052
Ssr3	Ssr3-001	0.00741114	0.835168	-1.19736
Ssr3	Ssr3-002	0.000362717	1.52939	1.52939
Ssr4	Ssr4-201	0.0188377	1.22275	1.22275
Ssrp1	Ssrp1-001	0.0267252	1.37256	1.37256
Sssca1	Sssca1-002	0.0459266	15.752	15.752
Sstr1	Sstr1-001	0.0461063	4.02192	4.02192
Ssu72	Ssu72-002	0.00105866	0.599534	-1.66796
Ssu72	Ssu72-001	0.00925902	0.669746	-1.4931
Ssu72	Ssu72-003	0.000696024	1.91965	1.91965
St13	St13-001	0.0464821	0.886669	-1.12782
St13	St13-005	0.0279733	1.56835	1.56835
St3gal6	St3gal6-001	0.00793731	0.00268608	-372.289
St6gal1	St6gal1-001	0.037162	0.568547	-1.75887
St6galnac4	St6galnac4-004	0.0148863	0.802309	-1.2464

St6galnac5	St6galnac5-001	0.0400731	0.757259	-1.32055
St6galnac6	St6galnac6-001	0.0337524	0.40896	-2.44523
St7l	St7l-010	0.0315228	1.61759	1.61759
Stam	Stam-001	0.0257276	0.672762	-1.48641
Stam2	Stam2-001	0.00222238	1.19805	1.19805
Stam2	Stam2-003	0.0113058	8.14284	8.14284
Stamos	Stamos-001	0.0040348	5.35945	5.35945
Star	Star-001	0.00776886	0.621355	-1.60939
Stard13	Stard13-001	0.0232946	1.76116	1.76116
Stard5	Stard5-001	0.0040341	2.39894	2.39894
Stard5	Stard5-005	0.00691383	2.44874	2.44874
Stard7	Stard7-005	0.0299875	1.39992	1.39992
Stard7	Stard7-003	0.0125953	1.51676	1.51676
Stau2	Stau2-009	0.0335253	2.7171	2.7171
Stau2	Stau2-012	0.00720904	2.98161	2.98161
Stc2	Stc2-001	0.0013256	1.66055	1.66055
Stc2	Stc2-004	0.000857011	2.00079	2.00079
Steap3	Steap3-001	0.00072932	0.445533	-2.2445
Stk11	Stk11-003	0.0182046	1.88161	1.88161
Stk11ip	Stk11ip-008	0.0206	0.320502	-3.12011
Stk11ip	Stk11ip-005	0.0386316	64.5052	64.5052
Stk16	Stk16-003	0.0270456	0.353102	-2.83205
Stk25	Stk25-005	0.00346594	0.675582	-1.48021
Stk3	Stk3-002	0.045199	1.42406	1.42406
Stk32a	Stk32a-201	6.4038e-06	1.57405	1.57405
Stk38	Stk38-015	0.00686454	0.273798	-3.65233
Stk38l	Stk38l-002	0.0040316	1.3887	1.3887
Stk39	Stk39-002	0.00830317	2.26937	2.26937
Stmn1	Stmn1-002	0.00513464	0.521787	-1.91649
Stmn1	Stmn1-001	0.0176719	0.797794	-1.25346
Stmn2	Stmn2-001	0.0102723	0.65225	-1.53315
Stradb	Stradb-002	0.039573	1.60496	1.60496
Strbp	Strbp-001	2.96017e-05	1.35973	1.35973
Strn4	Strn4-008	0.0395293	1.5946	1.5946
Strn4	Strn4-006	0.000977305	2.12956	2.12956
Stt3a	Stt3a-001	0.00561394	1.20937	1.20937
Stt3a	Stt3a-006	0.020048	1.32492	1.32492
Stt3a	Stt3a-005	0.00963261	1.36409	1.36409
Stx12	Stx12-001	0.00127113	0.864141	-1.15722
Stx17	Stx17-003	0.0471394	1.84117	1.84117
Stx18	Stx18-005	0.0130347	2.52564	2.52564
Stx1a	Stx1a-002	0.0159311	0.25127	-3.97979
Stx2	Stx2-001	0.0232694	0.783435	-1.27643

Stx8	Stx8-003	0.0108768	0.638834	-1.56535
Stx8	Stx8-001	0.0296679	0.765391	-1.30652
Stxbp2	Stxbp2-006	0.0238253	0.48739	-2.05175
Stxbp2	Stxbp2-001	0.036514	0.793947	-1.25953
Stxbp4	Stxbp4-001	0.0314922	1.20287	1.20287
Stxbp4	Stxbp4-005	0.0353076	1.91848	1.91848
Stxbp5	Stxbp5-004	0.041205	0.371801	-2.68961
Stxbp5	Stxbp5-001	0.000675386	0.576459	-1.73473
Stxbp5	Stxbp5-003	0.00170364	2.33379	2.33379
Stxbp6	Stxbp6-002	0.0152571	0.572911	-1.74547
Sub1	Sub1-001	0.00499076	0.531147	-1.88272
Sucla2	Sucla2-003	0.0473814	0.786181	-1.27197
Suclg1	Suclg1-001	0.0241777	1.32105	1.32105
Suclg2	Suclg2-002	0.00584955	1.16486	1.16486
Suds3	Suds3-002	0.011318	1.24954	1.24954
Sugp2	Sugp2-002	0.00639086	0.616659	-1.62164
Sult4a1	Sult4a1-201	0.043588	1.16544	1.16544
Sumf1	Sumf1-003	0.00132462	2.75573	2.75573
Sumf1	Sumf1-004	0.0141257	5.98691	5.98691
Sun2	Sun2-003	0.000689294	0.447022	-2.23703
Supt20	Supt20-007	0.0487843	1.70761	1.70761
Supt3	Supt3-001	0.0328176	1.46219	1.46219
Supt4a	Supt4a-001	0.0334857	1.24119	1.24119
Supt5	Supt5-201	0.00799105	1.1732	1.1732
Supv3l1	Supv3l1-004	0.0189224	0.373654	-2.67627
Surf2	Surf2-003	0.0360766	2.98437	2.98437
Surf6	Surf6-001	0.0153993	1.40782	1.40782
Suv420h1	Suv420h1-001	0.0087562	0.550558	-1.81634
Suv420h1	Suv420h1-005	0.00314324	2.08559	2.08559
Sv2a	Sv2a-001	0.0013777	0.768495	-1.30124
Sv2c	Sv2c-002	0.0209689	3.07582	3.07582
Svip	Svip-201	0.00867345	1.49497	1.49497
Svopl	Svopl-001	0.011236	2.00674	2.00674
Swap70	Swap70-201	0.00109477	1.62474	1.62474
Syde1	Syde1-201	0.0489397	1.43145	1.43145
Syn2	Syn2-003	0.000318534	0.509404	-1.96308
Syncrip	Syncrip-007	0.03603	0.379852	-2.63261
Syncrip	Syncrip-004	0.00831079	0.691195	-1.44677
Syncrip	Syncrip-001	0.026237	0.743239	-1.34546
Syncrip	Syncrip-008	0.00438683	4.34228	4.34228
Syngap1	Syngap1-007	0.0473729	0.0323376	-30.9238
Syngr1	Syngr1-002	0.0019403	1.70365	1.70365
Syngr2	Syngr2-006	0.0378954	0.265428	-3.7675

Syngr2	Syngr2-003	0.0385547	0.381518	-2.62111
Syngr3	Syngr3-001	0.0323565	0.874595	-1.14339
Synj2	Synj2-002	0.000488735	0.214244	-4.66757
Synj2	Synj2-014	0.0244447	0.526725	-1.89852
Synj2	Synj2-006	0.0226631	0.567092	-1.76338
Synj2	Synj2-011	0.0332676	8.15778	8.15778
Synpo2	Synpo2-004	0.0272398	0.443387	-2.25537
Synpo2	Synpo2-001	0.0314051	0.672122	-1.48783
Syp	Syp-001	0.039453	1.21556	1.21556
Syp	Syp-006	0.00796143	6.21513	6.21513
Sypl	Sypl-001	0.00110464	0.724068	-1.38108
Sypl	Sypl-002	0.0126966	1.32963	1.32963
Syt1	Syt1-001	0.00858207	0.655023	-1.52666
Syt1	Syt1-006	0.000204519	0.809308	-1.23562
Syt11	Syt11-201	0.00127933	0.794638	-1.25844
Syt15	Syt15-001	0.0205697	1.35088	1.35088
Syt4	Syt4-201	0.000299053	1.43498	1.43498
Syt7	Syt7-203	0.00193859	0.542926	-1.84187
Syt9	Syt9-001	0.00252465	1.70683	1.70683
Sytl4	Sytl4-003	0.0421536	1.58877	1.58877
Szrd1	Szrd1-002	0.0184234	1.28812	1.28812
Tab2	Tab2-001	0.0452586	1.08729	1.08729
Tac2	Tac2-202	0.00481312	6.82523	6.82523
Tacc3	Tacc3-004	0.00162202	5.21611	5.21611
Tada1	Tada1-001	0.0163084	1.27856	1.27856
Tada3	Tada3-006	0.00127121	0.124754	-8.0158
Taf1	Taf1-005	0.0284109	1.36291	1.36291
Taf10	Taf10-003	0.0142713	1.23729	1.23729
Taf11	Taf11-001	0.036596	1.30789	1.30789
Taf12	Taf12-001	0.0342412	1.52023	1.52023
Taf12	Taf12-003	0.0121356	1.57406	1.57406
Taf15	Taf15-001	0.0300462	1.12774	1.12774
Taf15	Taf15-002	0.0189195	1.99556	1.99556
Taf1a	Taf1a-002	0.0108003	1.24463	1.24463
Taf1b	Taf1b-201	0.00449764	1.3093	1.3093
Taf6l	Taf6l-006	0.0336964	2.48787	2.48787
Taf7	Taf7-001	0.00619932	1.53141	1.53141
Tagln2	Tagln2-003	0.00210495	0.623918	-1.60277
Tamm41	Tamm41-005	0.00627988	2.07218	2.07218
Tamm41	Tamm41-004	0.00494463	2.41232	2.41232
Tanc1	Tanc1-006	0.0136003	0.346741	-2.88399
Tanc2	Tanc2-005	0.046062	1.47431	1.47431
Tango2	Tango2-004	0.0349937	5.69952	5.69952

Tango6	Tango6-201	0.000149552	1.41142	1.41142
Tank	Tank-001	0.0482902	0.351068	-2.84845
Taok2	Taok2-001	0.00114146	0.863221	-1.15845
Taok3	Taok3-002	0.0479342	0.65759	-1.52071
Taok3	Taok3-007	0.0380888	1.45828	1.45828
Tap2	Tap2-002	0.0399075	0.850031	-1.17643
Tapbp	Tapbp-003	0.0127911	0.481193	-2.07817
Tapbp	Tapbp-001	5.65968e-05	0.561383	-1.78132
Tapt1	Tapt1-001	0.0326695	1.3712	1.3712
Tapt1	Tapt1-006	0.0252158	1.60146	1.60146
Tardbp	Tardbp-012	0.00965945	2.82442	2.82442
Tardbp	Tardbp-017	0.0118137	43.952	43.952
Tars	Tars-201	0.0349979	1.33668	1.33668
Tarsl2	Tarsl2-001	0.000201219	1.43255	1.43255
Tas1r1	Tas1r1-001	0.0369227	2.18948	2.18948
Tasp1	Tasp1-003	0.0189749	1.6852	1.6852
Tasp1	Tasp1-201	0.0190699	2.63454	2.63454
Tatdn1	Tatdn1-201	0.0129689	1.28569	1.28569
Tatdn3	Tatdn3-001	0.00379064	5.53216	5.53216
Tax1bp1	Tax1bp1-001	0.00200679	1.13464	1.13464
Tax1bp3	Tax1bp3-004	0.00167703	0.550835	-1.81542
Taz	Taz-001	0.0200723	0.391822	-2.55218
Taz	Taz-018	0.0481148	1.62953	1.62953
Tbc1d10c	Tbc1d10c-201	0.00182515	0.65055	-1.53716
Tbc1d14	Tbc1d14-006	0.00619023	0.431445	-2.31779
Tbc1d15	Tbc1d15-201	0.00355001	1.32388	1.32388
Tbc1d17	Tbc1d17-001	0.0388703	0.482804	-2.07124
Tbc1d19	Tbc1d19-007	0.0476538	2.11973	2.11973
Tbc1d20	Tbc1d20-001	0.0100735	1.20155	1.20155
Tbc1d23	Tbc1d23-201	0.0330858	1.14233	1.14233
Tbc1d31	Tbc1d31-003	0.0020892	3.0676	3.0676
Tbc1d32	Tbc1d32-201	2.85638e-05	1.57007	1.57007
Tbc1d8b	Tbc1d8b-001	0.00364561	1.37502	1.37502
Tbc1d9b	Tbc1d9b-003	0.0270376	3.52775	3.52775
Tbc1d9b	Tbc1d9b-004	0.00239379	4.46477	4.46477
Tbccd1	Tbccd1-201	0.0308164	1.38663	1.38663
Tbce	Tbce-006	0.0374601	2.00029	2.00029
Tbce	Tbce-008	0.000749835	2.18412	2.18412
Tbcel	Tbcel-002	0.0213577	23.2639	23.2639
Tbk1	Tbk1-201	8.79744e-05	1.1692	1.1692
Tbl1xr1	Tbl1xr1-001	0.00382577	0.655443	-1.52569
Tbl1xr1	Tbl1xr1-004	0.0293855	0.855674	-1.16867
Tbl1xr1	Tbl1xr1-003	0.0242222	2.7373	2.7373

Tbl1xr1	Tbl1xr1-006	0.00268499	2.98431	2.98431
Tbl2	Tbl2-002	0.00216114	1.69817	1.69817
Tbpl1	Tbpl1-002	1.72355e-06	1.85625	1.85625
Tbpl1	Tbpl1-004	0.000267066	3.07875	3.07875
Tbrg4	Tbrg4-004	0.0204875	1.72609	1.72609
Tbx4	Tbx4-002	0.0178841	1.52536	1.52536
Tc2n	Tc2n-001	0.0199294	1.46282	1.46282
Tcaim	Tcaim-001	0.0111297	1.41697	1.41697
Tcam1	Tcam1-001	0.0155446	0.229622	-4.35498
Tcea1	Tcea1-001	0.00193772	1.14879	1.14879
Tceanc	Tceanc-001	0.00469011	1.34444	1.34444
Tcerg1	Tcerg1-002	0.0105472	1.40546	1.40546
Tcerg1l	Tcerg1l-003	0.0295373	1.33306	1.33306
Tcf23	Tcf23-001	0.0454837	0.195075	-5.12622
Tcf3	Tcf3-003	0.00465007	0.430703	-2.32179
Tcf3	Tcf3-010	0.0107737	0.483283	-2.06918
Tcf4	Tcf4-033	0.0200451	0.387745	-2.57901
Tcf4	Tcf4-002	0.0266549	3.53434	3.53434
Tcf4	Tcf4-017	0.0355629	3.85277	3.85277
Tcf4	Tcf4-019	0.038112	4.78693	4.78693
Tcf4	Tcf4-054	0.0012055	4.97268	4.97268
Tchp	Tchp-001	0.0208014	2.01709	2.01709
Tcirg1	Tcirg1-010	0.0107135	1.89283e-05	-52830.9
Tcp1	Tcp1-007	0.00424323	1.78591	1.78591
Tcp11l1	Tcp11l1-001	0.00186348	1.30681	1.30681
Tcta	Tcta-001	0.00451559	1.64332	1.64332
Tcte2	Tcte2-010	0.0270709	0.337649	-2.96165
Tcte2	Tcte2-006	0.0422029	22.4274	22.4274
Tctn1	Tctn1-001	0.0226032	0.795397	-1.25723
Tdp1	Tdp1-001	0.000606531	1.38689	1.38689
Tdrd9	Tdrd9-001	0.0298583	0.141526	-7.06583
Tdrkh	Tdrkh-002	0.0478383	1.22939	1.22939
Tdrkh	Tdrkh-005	0.0432394	3.21354	3.21354
Tdrp	Tdrp-001	0.0216179	1.32291	1.32291
Tead1	Tead1-201	0.0484636	0.854734	-1.16995
Tead1	Tead1-006	0.0303693	35.141	35.141
Tec	Tec-005	0.0234666	0.334793	-2.98692
Tec	Tec-001	0.0469629	3.76017	3.76017
Tecpr2	Tecpr2-201	0.00540653	1.25961	1.25961
Tefm	Tefm-001	0.0206375	1.7512	1.7512
Ten1	Ten1-004	0.0187955	0.683496	-1.46307
Tenm4	Tenm4-009	0.00304804	0.559828	-1.78626
Tesc	Tesc-001	0.00214294	0.37754	-2.64873

Tesc	Tesc-006	0.00444727	0.61209	-1.63375
Tesk1	Tesk1-003	0.0117392	0.163868	-6.10248
Tex19.1	Tex19.1-001	0.0181172	3.12219	3.12219
Tex2	Tex2-001	0.0145468	0.822717	-1.21548
Tex264	Tex264-009	0.0401651	0.00145973	-685.056
Tex264	Tex264-003	0.000341612	0.405405	-2.46667
Tex9	Tex9-003	0.0431926	0.113855	-8.7831
Tfb1m	Tfb1m-201	0.000357092	1.8042	1.8042
Tfdp2	Tfdp2-014	0.0441418	0.046994	-21.2793
Tfdp2	Tfdp2-002	0.000186854	0.585682	-1.70741
Tfe3	Tfe3-014	0.0477456	2.50467	2.50467
Tfeb	Tfeb-009	0.0241069	0.520079	-1.92278
Tfpt	Tfpt-002	0.0146849	2.73557	2.73557
Tgfb1	Tgfb1-001	0.0219895	0.355175	-2.81551
Tgfb1i1	Tgfb1i1-008	0.00150046	526.516	526.516
Tgfb2	Tgfb2-001	0.00117525	0.545626	-1.83276
Tgfbi	Tgfbi-201	0.0138311	0.736613	-1.35757
Tgfbr1	Tgfbr1-001	0.00522948	0.724948	-1.37941
Tgfbrap1	Tgfbrap1-006	0.00717843	1.61796	1.61796
Tgfbrap1	Tgfbrap1-002	0.0139065	1.88094	1.88094
Tgs1	Tgs1-001	0.0385784	1.39389	1.39389
Tgs1	Tgs1-005	0.00344716	2.37588	2.37588
Th	Th-006	0.039341	422.901	422.901
Thap1	Thap1-001	0.00397084	1.35022	1.35022
Thap3	Thap3-001	0.0168509	1.44063	1.44063
Thap4	Thap4-005	0.0406958	1.38601	1.38601
Thap8	Thap8-201	0.00633861	8.90179	8.90179
Thbs3	Thbs3-001	0.00185949	0.369561	-2.70591
Them4	Them4-001	0.000792678	1.97141	1.97141
Thnsl1	Thnsl1-001	0.00215892	1.25071	1.25071
Thra	Thra-005	0.0275823	7.15085e-07	-1.39844e+06
Thrb	Thrb-201	0.0423656	1.78525e+06	1.78525e+06
Thrb	Thrb-001	0.0423656	2.7453e+06	2.7453e+06
Thsd7a	Thsd7a-003	0.0381074	0.324643	-3.08031
Thsd7a	Thsd7a-201	0.00351079	0.406	-2.46305
Thumpd2	Thumpd2-201	0.0183928	1.25793	1.25793
Thumpd3	Thumpd3-001	0.0087958	1.34414	1.34414
Tia1	Tia1-002	0.0447205	0.00573027	-174.512
Tia1	Tia1-018	0.0466087	1.92447	1.92447
Tia1	Tia1-013	0.0296475	2.84231	2.84231
Tial1	Tial1-001	0.0362123	0.424582	-2.35526
Tial1	Tial1-007	0.00362504	0.638181	-1.56695
Tial1	Tial1-003	0.0376006	2.03576	2.03576

Tiam1	Tiam1-009	0.0336925	0.195756	-5.1084
Tiam2	Tiam2-201	0.0110832	0.541413	-1.84702
Tigd2	Tigd2-001	0.00025197	1.51623	1.51623
Timm10b	Timm10b-004	0.00688566	0.0841164	-11.8883
Timm17a	Timm17a-002	0.0469578	5.08833	5.08833
Timm17b	Timm17b-003	0.0347104	1.26837	1.26837
Timm8b	Timm8b-201	9.92865e-06	1.2742	1.2742
Timp2	Timp2-001	0.0288145	0.810291	-1.23413
Timp3	Timp3-001	0.0360413	0.71629	-1.39608
Tinagl1	Tinagl1-001	0.000608963	0.139186	-7.18461
Tinf2	Tinf2-201	0.0368045	0.896512	-1.11543
Tiprl	Tiprl-004	0.0122984	1.25468	1.25468
Tiprl	Tiprl-001	0.00546229	1.25811	1.25811
Tjap1	Tjap1-202	0.0321839	0.661643	-1.51139
Tjp1	Tjp1-005	0.0204465	1.66623	1.66623
Tjp2	Tjp2-201	0.0279189	1.58479	1.58479
Tk1	Tk1-001	0.0076927	0.53413	-1.8722
Tk1	Tk1-005	0.0352509	0.691872	-1.44535
Tk2	Tk2-201	0.0121087	1.24232	1.24232
Tkfc	Tkfc-201	0.0108407	0.654233	-1.52851
Tldc1	Tldc1-201	0.0150166	1.50817	1.50817
Tle1	Tle1-008	0.00147765	2.11572	2.11572
Tle3	Tle3-005	0.0112445	0.618721	-1.61624
Tle3	Tle3-012	0.0145859	0.722989	-1.38315
Tlk2	Tlk2-002	0.0271193	1.80588	1.80588
Tln2	Tln2-202	0.00372745	0.744735	-1.34276
Tlr12	Tlr12-001	0.0160248	0.630157	-1.58691
Tlr4	Tlr4-002	0.0294059	5.72025	5.72025
Tm2d3	Tm2d3-002	0.0370557	1.3646	1.3646
Tm6sf2	Tm6sf2-003	0.0235822	0.23027	-4.34272
Tm6sf2	Tm6sf2-005	0.0401024	91.0784	91.0784
Tm7sf2	Tm7sf2-003	0.0153797	0.346397	-2.88686
Tm9sf1	Tm9sf1-006	0.0400812	1.64102	1.64102
Tma16	Tma16-003	0.0400463	1.57004	1.57004
Tma16	Tma16-001	0.00359428	1.79824	1.79824
Tmbim1	Tmbim1-003	0.0470673	0.453596	-2.20461
Tmbim1	Tmbim1-001	0.0226489	0.643345	-1.55438
Tmbim4	Tmbim4-003	0.0231496	1.69402	1.69402
Tmbim6	Tmbim6-002	0.032263	13.495	13.495
Tmc6	Tmc6-007	0.0419795	0.392535	-2.54754
Tmc6	Tmc6-001	0.016332	0.733293	-1.36371
Tmc8	Tmc8-004	0.013881	0.353646	-2.82768
Tmcc1	Tmcc1-003	0.0252441	0.553575	-1.80644

Tmcc1	Tmcc1-007	0.0460073	2.66859	2.66859
Tmcc1	Tmcc1-008	0.0398593	2.77275	2.77275
Tmcc3	Tmcc3-004	0.0183186	16.885	16.885
Tmcc3os	Tmcc3os-001	0.00834	2.00411	2.00411
Tmco6	Tmco6-201	0.0248627	1.34034	1.34034
Tmed3	Tmed3-001	0.0458118	1.12637	1.12637
Tmed5	Tmed5-002	0.0148819	1.70643	1.70643
Tmed7	Tmed7-002	0.00272118	0.704134	-1.42018
Tmed9	Tmed9-201	0.0123772	1.18543	1.18543
Tmeff1	Tmeff1-001	0.0337804	0.828436	-1.20709
Tmem101	Tmem101-002	0.00326859	9.78187	9.78187
Tmem106a	Tmem106a-001	0.000545938	0.596009	-1.67783
Tmem106b	Tmem106b-005	0.0366054	2.23437	2.23437
Tmem107	Tmem107-001	0.0447803	0.778522	-1.28449
Tmem107	Tmem107-002	0.00955772	2.14839	2.14839
Tmem108	Tmem108-003	1.77487e-05	0.366067	-2.73174
Tmem108	Tmem108-005	0.0160967	0.566675	-1.76468
Tmem108	Tmem108-001	0.0324696	0.575116	-1.73878
Tmem109	Tmem109-001	0.0138243	1.41733	1.41733
Tmem109	Tmem109-002	0.0197062	2.50142	2.50142
Tmem11	Tmem11-001	1.15865e-05	1.42955	1.42955
Tmem110	Tmem110-001	0.0248915	0.756608	-1.32169
Tmem110	Tmem110-002	0.00282905	2.213	2.213
Tmem120a	Tmem120a-001	0.00906895	0.753249	-1.32758
Tmem120b	Tmem120b-001	0.0389678	1.51198	1.51198
Tmem123	Tmem123-001	0.00105428	0.445417	-2.24509
Tmem129	Tmem129-002	0.00157986	0.714636	-1.39931
Tmem129	Tmem129-001	0.0390488	1.28909	1.28909
Tmem131	Tmem131-201	0.00395285	1.29113	1.29113
Tmem134	Tmem134-001	0.0187447	0.213636	-4.68086
Tmem134	Tmem134-013	0.0292436	1.49844	1.49844
Tmem135	Tmem135-001	0.00661347	1.21846	1.21846
Tmem135	Tmem135-007	0.0339209	2.13999	2.13999
Tmem136	Tmem136-201	0.0143173	1.27211	1.27211
Tmem144	Tmem144-002	0.0207187	2.45711	2.45711
Tmem147os	Tmem147os-001	0.0174928	1.88987	1.88987
Tmem150a	Tmem150a-001	0.0144909	0.590346	-1.69392
Tmem151a	Tmem151a-001	0.0204322	1.16764	1.16764
Tmem151b	Tmem151b-201	0.00160006	0.39892	-2.50677
Tmem161a	Tmem161a-006	0.0148902	3.20027	3.20027
Tmem161a	Tmem161a-014	0.0292394	24.369	24.369
Tmem164	Tmem164-005	0.00461476	0.294276	-3.39817
Tmem164	Tmem164-008	0.00599147	0.3028	-3.30251

Tmem164	Tmem164-002	0.00469947	0.430381	-2.32352
Tmem164	Tmem164-001	0.0231264	0.685638	-1.45849
Tmem168	Tmem168-001	0.0017099	0.776107	-1.28848
Tmem168	Tmem168-003	0.0246925	1.81617	1.81617
Tmem170	Tmem170-201	0.00932011	1.21348	1.21348
Tmem173	Tmem173-201	0.000459405	0.41387	-2.41622
Tmem176b	Tmem176b-004	0.0124714	0.514224	-1.94468
Tmem176b	Tmem176b-007	0.0325694	0.735431	-1.35975
Tmem177	Tmem177-001	4.00155e-05	1.60324	1.60324
Tmem183a	Tmem183a-001	0.00548458	0.712037	-1.40442
Tmem183a	Tmem183a-002	0.00714009	1.57034	1.57034
Tmem184a	Tmem184a-001	0.0319465	24.4868	24.4868
Tmem19	Tmem19-201	0.0270604	0.789855	-1.26606
Tmem194b	Tmem194b-001	0.00120544	3.53531	3.53531
Tmem198b	Tmem198b-001	0.0165935	0.68891	-1.45157
Tmem199	Tmem199-001	0.0112082	1.52082	1.52082
Tmem199	Tmem199-004	0.00531783	1.57615	1.57615
Tmem2	Tmem2-201	0.00807362	0.627174	-1.59445
Tmem2	Tmem2-202	0.00322893	0.636839	-1.57026
Tmem203	Tmem203-001	0.028177	1.4165	1.4165
Tmem206	Tmem206-001	0.0152075	1.19974	1.19974
Tmem208	Tmem208-004	0.0113758	1.7066	1.7066
Tmem209	Tmem209-001	0.0141664	0.858643	-1.16463
Tmem209	Tmem209-005	0.0224308	1.38375	1.38375
Tmem209	Tmem209-003	0.0226912	1.59931	1.59931
Tmem214	Tmem214-004	0.00172313	2.01621	2.01621
Tmem216	Tmem216-002	0.0123684	2.7718	2.7718
Tmem218	Tmem218-201	0.000145796	1.79509	1.79509
Tmem222	Tmem222-004	0.0352503	1.66262	1.66262
Tmem222	Tmem222-005	0.0157742	1.85616	1.85616
Tmem234	Tmem234-010	0.0464252	1.85975	1.85975
Tmem240	Tmem240-003	0.0445155	1.29561	1.29561
Tmem243	Tmem243-004	0.0254342	0.674731	-1.48207
Tmem245	Tmem245-001	0.0234837	0.905847	-1.10394
Tmem248	Tmem248-001	0.0425492	1.2749	1.2749
Tmem253	Tmem253-201	0.00338013	1.90679	1.90679
Tmem256	Tmem256-002	0.0341281	0.310472	-3.2209
Tmem256	Tmem256-004	0.017899	0.410465	-2.43626
Tmem259	Tmem259-005	0.00135295	1.97071	1.97071
Tmem261	Tmem261-001	0.00560611	1.59002	1.59002
Tmem28	Tmem28-001	0.00830335	0.344126	-2.90592
Tmem30a	Tmem30a-001	0.000194822	0.738177	-1.35469
Tmem30a	Tmem30a-003	0.00570087	1.6101	1.6101

Tmem33	Tmem33-002	0.0360202	2.02223	2.02223
Tmem37	Tmem37-001	0.00523462	1.41121	1.41121
Tmem39a	Tmem39a-003	0.00198837	1.48029	1.48029
Tmem39a	Tmem39a-004	0.00161205	1.69254	1.69254
Tmem39a	Tmem39a-011	0.0458816	3.36294	3.36294
Tmem39b	Tmem39b-001	0.0172595	1.43461	1.43461
Tmem39b	Tmem39b-003	0.0285412	13.3161	13.3161
Tmem41b	Tmem41b-003	0.00170402	1.2336	1.2336
Tmem41b	Tmem41b-005	0.0193642	1.37922	1.37922
Tmem41b	Tmem41b-004	0.0222307	2.02091	2.02091
Tmem43	Tmem43-002	0.00233403	0.0812753	-12.3039
Tmem43	Tmem43-001	0.00468836	0.768887	-1.30058
Tmem50b	Tmem50b-001	0.0013145	0.776907	-1.28716
Tmem55b	Tmem55b-007	0.015903	2.28835	2.28835
Tmem56	Tmem56-001	0.0298711	0.699204	-1.4302
Tmem56	Tmem56-002	0.0101019	2.03241	2.03241
Tmem59	Tmem59-002	0.0236012	0.747559	-1.33769
Tmem60	Tmem60-001	0.0187694	1.24125	1.24125
Tmem62	Tmem62-001	0.0161765	1.68242	1.68242
Tmem63a	Tmem63a-003	0.0376183	0.217938	-4.58845
Tmem63b	Tmem63b-001	0.000249261	0.755189	-1.32417
Tmem63b	Tmem63b-007	0.01897	1.33577	1.33577
Tmem63b	Tmem63b-008	0.0427345	1.44563	1.44563
Tmem65	Tmem65-001	0.0136151	1.19224	1.19224
Tmem68	Tmem68-006	0.0452749	10.6146	10.6146
Tmem71	Tmem71-201	0.00227874	2.13156	2.13156
Tmem74b	Tmem74b-002	0.00794287	0.145462	-6.87467
Tmem74b	Tmem74b-001	0.00350265	3.47581	3.47581
Tmem79	Tmem79-003	0.0334999	0.447576	-2.23426
Tmem79	Tmem79-004	0.0470415	2.41689	2.41689
Tmem8	Tmem8-001	0.0349915	1.53632	1.53632
Tmem8	Tmem8-002	0.00841059	5.94469	5.94469
Tmem80	Tmem80-004	0.0120773	2.10636	2.10636
Tmem9	Tmem9-005	0.0177009	0.0428831	-23.3192
Tmem9	Tmem9-001	0.000147124	0.574313	-1.74121
Tmem91	Tmem91-002	0.0344813	4.43479	4.43479
Tmem9b	Tmem9b-002	0.0227607	2.35746	2.35746
Tmigd3	Tmigd3-006	0.0290194	356.935	356.935
Tmigd3	Tmigd3-004	0.0120261	656.608	656.608
Tmod1	Tmod1-001	0.0169296	0.664964	-1.50384
Tmod2	Tmod2-201	0.0332069	0.585034	-1.7093
Tmpo	Tmpo-201	0.0339463	1.27375	1.27375
Tmsb10	Tmsb10-001	0.00194026	0.828668	-1.20676

Tmsb4x	Tmsb4x-001	0.00170471	0.531524	-1.88138
Tmtc1	Tmtc1-002	0.012798	9.7339	9.7339
Tmtc2	Tmtc2-001	0.000683496	0.665305	-1.50307
Tmtc3	Tmtc3-003	0.00981368	8.20838	8.20838
Tmub1	Tmub1-005	0.0231397	0.188771	-5.29743
Tmub1	Tmub1-001	0.00275682	2.11145	2.11145
Tmx3	Tmx3-201	0.0148849	0.830372	-1.20428
Tmx4	Tmx4-001	0.00615894	1.27794	1.27794
Tnfaip2	Tnfaip2-011	0.0294755	406.214	406.214
Tnfaip3	Tnfaip3-002	0.0124354	0.216725	-4.61414
Tnfrsf12a	Tnfrsf12a-201	0.0133864	1.18579	1.18579
Tnfrsf25	Tnfrsf25-004	0.040923	0.555854	-1.79903
Tnfrsf25	Tnfrsf25-002	0.0146227	0.576879	-1.73347
Tnfrsf9	Tnfrsf9-202	0.000866033	0.0647954	-15.4332
Tnfsf12	Tnfsf12-001	0.000351737	1.58035	1.58035
Tnip2	Tnip2-007	0.0262828	0.572744	-1.74598
Tnk2	Tnk2-005	0.0106579	1.96526	1.96526
Tnks	Tnks-201	0.0126412	0.825091	-1.21199
Tnni1	Tnni1-001	0.0435874	0.323549	-3.09072
Tnnt2	Tnnt2-004	0.0284883	0.000378816	-2639.8
Tnnt2	Tnnt2-006	0.0226049	0.217852	-4.59027
Tnpo1	Tnpo1-003	0.00556123	2.27979	2.27979
Tnpo2	Tnpo2-202	0.0108168	0.840763	-1.1894
Tnpo2	Tnpo2-201	0.00288515	0.871318	-1.14769
Tnpo3	Tnpo3-010	0.040583	0.558826	-1.78947
Tnrc6a	Tnrc6a-001	0.00401424	0.884721	-1.1303
Tnrc6a	Tnrc6a-005	0.0394921	1.23994	1.23994
Tnrc6c	Tnrc6c-003	0.00301543	0.640685	-1.56083
Tnrc6c	Tnrc6c-004	0.0181035	0.712014	-1.40447
Tns1	Tns1-201	0.00121772	0.55876	-1.78968
Tns1	Tns1-008	0.0314131	0.572769	-1.7459
Tns1	Tns1-001	0.0392967	0.603627	-1.65665
Tns3	Tns3-004	0.0109269	19.7449	19.7449
Toe1	Toe1-003	0.007289	2.76945e-08	-3.61082e+07
Tom1	Tom1-202	0.0499044	1.16961	1.16961
Tom1	Tom1-201	0.0350508	4.07539	4.07539
Tom1l1	Tom1l1-002	0.000473262	1.47529	1.47529
Tom1l1	Tom1l1-008	0.000280923	1.79645	1.79645
Tomm34	Tomm34-001	0.00473305	0.870393	-1.14891
Tomm40l	Tomm40l-008	0.0406432	0.046636	-21.4427
Tomm6	Tomm6-002	0.0102087	1.31497	1.31497
Top2a	Top2a-007	7.97413e-06	0.466403	-2.14407
Top2a	Top2a-001	0.0398851	0.866186	-1.15449

Top2b	Top2b-003	0.0332997	0.0961018	-10.4056
Topbp1	Topbp1-010	0.0369609	0.577421	-1.73184
Topbp1	Topbp1-001	0.0310836	0.726245	-1.37695
Tor3a	Tor3a-001	0.0396806	0.801932	-1.24699
Tor4a	Tor4a-001	0.0180043	0.481189	-2.07819
Tpcn1	Tpcn1-004	0.0106971	0.47162	-2.12035
Tpd52l2	Tpd52l2-002	0.0479206	0.638359	-1.56652
Tph1	Tph1-001	0.0100815	0.652488	-1.53259
Tpm1	Tpm1-020	0.0426864	4.88363	4.88363
Tpm2	Tpm2-201	0.00730428	0.795309	-1.25737
Tpm3	Tpm3-002	0.03499	0.388017	-2.5772
Tpm4	Tpm4-001	0.000263707	0.584864	-1.7098
Tpp1	Tpp1-201	0.000968549	0.869196	-1.15049
Tpp2	Tpp2-009	0.0114839	2.90329	2.90329
Tpr	Tpr-003	0.0331388	0.728573	-1.37255
Tpra1	Tpra1-017	0.00362523	0.632947	-1.57991
Tprgl	Tprgl-001	0.00968496	0.754659	-1.3251
Tprkb	Tprkb-003	0.00186186	1.498	1.498
Tprkb	Tprkb-002	0.0122646	1.85577	1.85577
Tpst2	Tpst2-002	0.0270282	1.85531	1.85531
Tpt1	Tpt1-003	0.0164052	0.382355	-2.61537
Tpx2	Tpx2-002	0.0152784	1.24263	1.24263
Tpx2	Tpx2-001	0.0469207	1.3898	1.3898
Trabd	Trabd-001	0.0374261	0.817946	-1.22257
Trabd2b	Trabd2b-001	0.0254975	0.233307	-4.2862
Tradd	Tradd-001	0.0218767	0.749169	-1.33481
Traf3	Traf3-004	0.0197466	10.1762	10.1762
Traf3ip2	Traf3ip2-002	0.00562865	0.337561	-2.96242
Traf3ip2	Traf3ip2-001	0.0348043	0.791032	-1.26417
Traf4	Traf4-002	0.0118674	0.417688	-2.39413
Traf4	Traf4-001	0.00055105	0.597431	-1.67383
Trak1	Trak1-201	0.00401972	0.715891	-1.39686
Tram1	Tram1-001	0.032384	1.14053	1.14053
Trappc12	Trappc12-201	0.0412468	1.71213	1.71213
Trappc13	Trappc13-002	0.0299924	0.566626	-1.76483
Trappc2	Trappc2-002	0.0467392	1.94061	1.94061
Trappc2l	Trappc2l-201	0.0457593	0.808873	-1.23629
Trappc8	Trappc8-201	0.0115583	1.23326	1.23326
Trdmt1	Trdmt1-001	0.00275572	1.60446	1.60446
Trdmt1	Trdmt1-003	0.000384833	2.01327	2.01327
Trerf1	Trerf1-001	0.00237673	0.48825	-2.04813
Trerf1	Trerf1-005	0.0320012	2.5726	2.5726
Trex1	Trex1-001	0.0490109	0.314873	-3.17588

Trib2	Trib2-001	0.00124707	0.826541	-1.20986
Trib3	Trib3-001	0.000771336	2.85465	2.85465
Trib3	Trib3-003	0.00729184	3.15751	3.15751
Trim16	Trim16-005	0.0422444	2.89093	2.89093
Trim2	Trim2-004	0.00132832	0.728539	-1.37261
Trim2	Trim2-010	0.00142824	2.73147	2.73147
Trim23	Trim23-001	0.0161737	1.22018	1.22018
Trim25	Trim25-002	0.0228076	0.66072	-1.5135
Trim28	Trim28-004	0.0426571	1.90167	1.90167
Trim33	Trim33-001	0.00800344	0.630912	-1.58501
Trim33	Trim33-004	0.0285058	1.58658	1.58658
Trim35	Trim35-001	2.17831e-05	0.658124	-1.51947
Trim37	Trim37-001	0.00184224	0.605539	-1.65142
Trim39	Trim39-003	0.0137984	1.89612	1.89612
Trim39	Trim39-004	0.039742	2.76451	2.76451
Trim41	Trim41-001	0.00123097	1.15658	1.15658
Trim46	Trim46-003	0.00358663	1.73237	1.73237
Trim47	Trim47-006	0.00132831	0.00916725	-109.084
Trim47	Trim47-002	0.0101692	0.529866	-1.88727
Trim47	Trim47-001	0.0377337	0.705913	-1.41661
Trim59	Trim59-005	0.00527218	0.30406	-3.28882
Trim62	Trim62-002	0.0101007	0.357252	-2.79915
Trim62	Trim62-001	5.06961e-05	0.565973	-1.76687
Trim66	Trim66-004	4.62558e-05	6.91517	6.91517
Trim66	Trim66-003	0.00592616	9.29273	9.29273
Trim66	Trim66-002	0.00592544	9.29274	9.29274
Trim67	Trim67-201	0.000270633	0.41123	-2.43173
Trim7	Trim7-001	0.0345445	2.02205	2.02205
Trim71	Trim71-001	0.0384166	0.785968	-1.27232
Trim80	Trim80-001	0.0423103	2.6794	2.6794
Trio	Trio-201	0.0178578	1.12631	1.12631
Triobp	Triobp-005	0.0175374	0.769843	-1.29897
Triobp	Triobp-201	0.0212588	0.801462	-1.24772
Trip10	Trip10-201	0.000677108	0.621047	-1.61018
Trip12	Trip12-201	0.00541587	0.534432	-1.87115
Trip12	Trip12-001	0.00956864	0.548129	-1.82439
Trip4	Trip4-002	0.0327246	1.35654	1.35654
Trip6	Trip6-001	0.0212457	0.683135	-1.46384
Trit1	Trit1-001	0.0463067	1.55537	1.55537
Trmt1	Trmt1-030	0.0159545	2.3226	2.3226
Trmt10b	Trmt10b-006	0.0301279	0.137877	-7.25282
Trmt10b	Trmt10b-002	0.0110433	1.7749	1.7749
Trmt11	Trmt11-201	0.0218174	1.48922	1.48922

Trmt61b	Trmt61b-003	0.0126902	1.84558	1.84558
Trmt61b	Trmt61b-001	0.0292091	2.67168	2.67168
Trnau1ap	Trnau1ap-012	0.0178583	14.1909	14.1909
Trnp1	Trnp1-001	0.0417288	0.749148	-1.33485
Trnp1	Trnp1-002	0.00462808	1.9456	1.9456
Tro	Tro-001	0.00320362	1.19349	1.19349
Trove2	Trove2-001	0.0194519	0.707005	-1.41442
Trp53	Trp53-003	0.036371	0.678236	-1.47441
Trp53bp1	Trp53bp1-002	0.000419816	0.507963	-1.96865
Trp53bp1	Trp53bp1-001	0.0170867	0.671567	-1.48905
Trp53cor1	Trp53cor1-001	0.0057585	1.55531	1.55531
Trp53i11	Trp53i11-001	0.00850099	1.41414	1.41414
Trp53i13	Trp53i13-005	0.0491995	2.49745	2.49745
Trp53inp2	Trp53inp2-001	0.0010812	0.790218	-1.26547
Trpc3	Trpc3-002	0.024947	1.27185e+10	1.27185e+10
Trpc4	Trpc4-002	0.0152745	4.09429	4.09429
Trpm3	Trpm3-204	0.0119348	0.236461	-4.22902
Trpm7	Trpm7-010	0.0435267	0.00227027	-440.476
Trrap	Trrap-004	0.00895547	0.669746	-1.4931
Trub1	Trub1-202	0.00675211	0.429359	-2.32905
Trub1	Trub1-201	0.00321505	0.643393	-1.55426
Trub2	Trub2-004	0.0140042	3.34932	3.34932
Tsc1	Tsc1-004	0.0222701	2.12556	2.12556
Tsc22d2	Tsc22d2-002	0.0175337	1.31893	1.31893
Tsc22d4	Tsc22d4-002	0.0428468	0.78537	-1.27329
Tsc22d4	Tsc22d4-001	0.0456769	1.28288	1.28288
Tsen15	Tsen15-001	0.0022067	1.45248	1.45248
Tsen2	Tsen2-001	0.00591165	1.3012	1.3012
Tsen2	Tsen2-005	0.0165071	2.20001	2.20001
Tsks	Tsks-202	0.0316187	0.335214	-2.98317
Tsks	Tsks-201	0.0316188	0.335214	-2.98317
Tsku	Tsku-003	0.0264207	0.262434	-3.81048
Tspan13	Tspan13-001	0.00119584	1.14708	1.14708
Tspan14	Tspan14-201	0.00963523	0.687288	-1.45499
Tspan15	Tspan15-201	0.000799412	1.52223	1.52223
Tspan18	Tspan18-001	0.00128112	0.580756	-1.72189
Tspan18	Tspan18-004	0.0440885	0.647708	-1.54391
Tspan2	Tspan2-001	0.0128613	0.805182	-1.24196
Tspan33	Tspan33-003	0.0247851	16.6055	16.6055
Tspan4	Tspan4-001	0.0238393	0.622503	-1.60642
Tspan9	Tspan9-011	0.0336102	0.000568201	-1759.94
Tspo	Tspo-201	0.000181874	0.564963	-1.77003
Tspo2	Tspo2-002	0.00195026	20.8405	20.8405

Tspyl2	Tspyl2-001	0.012755	1.37304	1.37304
Tspyl2	Tspyl2-002	0.0351434	1.87237	1.87237
Tspyl2	Tspyl2-003	0.0499743	2.19915	2.19915
Tspyl2	Tspyl2-004	0.00475172	7.4677	7.4677
Tspyl3	Tspyl3-001	4.00342e-05	0.515192	-1.94103
Tspyl4	Tspyl4-001	0.0315713	1.20039	1.20039
Tsr1	Tsr1-006	0.041291	3.87941	3.87941
Tsr2	Tsr2-002	0.00297434	1.35925	1.35925
Tsr3	Tsr3-201	0.0125364	1.19493	1.19493
Tssc1	Tssc1-201	0.00718965	1.2406	1.2406
Tstd1	Tstd1-001	0.0061958	0.336382	-2.97281
Tstd3	Tstd3-001	0.000464171	1.25706	1.25706
Ttbk2	Ttbk2-005	0.035273	0.172727	-5.78949
Ttbk2	Ttbk2-002	0.0376199	0.739513	-1.35224
Ttc1	Ttc1-201	0.025903	1.30422	1.30422
Ttc13	Ttc13-007	0.0156249	0.655864	-1.52471
Ttc13	Ttc13-004	0.012009	1.41943	1.41943
Ttc16	Ttc16-005	0.0386769	36.5219	36.5219
Ttc17	Ttc17-002	0.0113206	1.65875	1.65875
Ttc19	Ttc19-005	0.000382969	2.76003	2.76003
Ttc19	Ttc19-004	0.00444978	5.46414	5.46414
Ttc21b	Ttc21b-003	0.0356999	1.85547	1.85547
Ttc23	Ttc23-006	0.0058625	0.186511	-5.36161
Ttc23	Ttc23-004	0.0430109	3.21999	3.21999
Ttc25	Ttc25-002	0.0431057	2.56811	2.56811
Ttc26	Ttc26-001	0.00437511	1.54989	1.54989
Ttc3	Ttc3-001	0.012411	1.29771	1.29771
Ttc3	Ttc3-005	0.0271829	1.43368	1.43368
Ttc33	Ttc33-004	0.0279448	100891	100891
Ttc37	Ttc37-201	0.0241375	1.23089	1.23089
Ttc39a	Ttc39a-002	0.00840059	0.477475	-2.09435
Ttc39aos1	Ttc39aos1-002	0.0290435	0.357999	-2.7933
Ttc39b	Ttc39b-002	0.000480256	3.84249	3.84249
Ttc4	Ttc4-001	0.0180154	1.26505	1.26505
Ttc41	Ttc41-201	0.0467755	0.0170468	-58.6619
Ttc7b	Ttc7b-201	0.049258	0.905182	-1.10475
Ttc8	Ttc8-006	0.038913	1.71742	1.71742
Tti2	Tti2-201	0.0148552	1.29922	1.29922
Ttll1	Ttll1-001	4.13231e-05	1.53576	1.53576
Ttll13	Ttll13-004	0.0451916	2.83505	2.83505
Ttll5	Ttll5-009	0.0308216	2.67597	2.67597
Ttyh2	Ttyh2-001	0.00136436	0.604486	-1.6543
Ttyh3	Ttyh3-005	0.0157192	0.257914	-3.87726

Ttyh3	Ttyh3-001	0.000478018	0.396805	-2.52013
Ttyh3	Ttyh3-002	0.000110873	0.41741	-2.39573
Ttyh3	Ttyh3-004	0.0419672	0.661102	-1.51263
Tuba1a	Tuba1a-001	0.00133701	0.748388	-1.33621
Tuba1a	Tuba1a-002	0.0063181	1.51417	1.51417
Tuba1b	Tuba1b-002	0.026959	1.58592	1.58592
Tubb2a	Tubb2a-201	0.0186728	0.819921	-1.21963
Tubb2b	Tubb2b-201	0.0496579	0.566048	-1.76663
Tubb3	Tubb3-001	0.00011428	0.725926	-1.37755
Tubb6	Tubb6-201	0.00188441	0.722151	-1.38475
Tubgcp4	Tubgcp4-002	0.00423922	2.29554	2.29554
Tulp4	Tulp4-005	0.0273233	0.715571	-1.39749
Tulp4	Tulp4-002	0.00769421	0.779495	-1.28288
Tvp23b	Tvp23b-001	0.00760681	1.15741	1.15741
Tvp23b	Tvp23b-002	0.00437409	3.92526	3.92526
Txlng	Txlng-003	0.000665052	1.66311	1.66311
Txn2	Txn2-001	0.00919884	1.38147	1.38147
Txndc11	Txndc11-003	0.018833	1.41299	1.41299
Txndc11	Txndc11-007	0.0242367	27.332	27.332
Txndc16	Txndc16-001	0.0224928	1.36192	1.36192
Txndc9	Txndc9-007	0.0139378	1.34346	1.34346
Txnl4a	Txnl4a-002	0.0292045	2.00546	2.00546
Txnrd3	Txnrd3-001	0.00329829	0.789275	-1.26699
Tyk2	Tyk2-201	0.0068823	0.627793	-1.59288
Tyms	Tyms-003	0.00125706	0.552901	-1.80864
Tyro3	Tyro3-002	0.00319903	0.652857	-1.53173
Tyw1	Tyw1-002	0.0010633	1.60956	1.60956
Tyw1	Tyw1-201	0.0010633	1.60956	1.60956
Tyw1	Tyw1-003	0.0269919	2.41677	2.41677
U2af1l4	U2af1l4-022	0.0401466	1.88065	1.88065
U2af1l4	U2af1l4-009	0.0371103	8.6349	8.6349
U2surp	U2surp-004	0.0367565	0.410048	-2.43874
U2surp	U2surp-002	0.000495707	0.67066	-1.49107
U2surp	U2surp-009	0.00127074	1.69719	1.69719
Uap1l1	Uap1l1-006	0.0266033	2.92359	2.92359
Uba3	Uba3-004	0.000380639	1.89573	1.89573
Uba5	Uba5-004	0.00273111	1.37569	1.37569
Uba52	Uba52-004	0.0189671	1.54748	1.54748
Uba52	Uba52-003	0.00411093	1.60263	1.60263
Uba6	Uba6-001	0.0114797	0.841545	-1.18829
Uba6	Uba6-003	0.00681521	1.86998	1.86998
Uba7	Uba7-007	0.0165201	0.115194	-8.681
Ubap1	Ubap1-002	0.00421395	0.61027	-1.63862

Ubap2l	Ubap2l-023	0.00998185	0.289342	-3.45612
Ubap2l	Ubap2l-006	0.00602522	0.440612	-2.26957
Ubap2l	Ubap2l-003	0.00726214	1.38306	1.38306
Ubash3b	Ubash3b-001	0.00977988	0.695753	-1.43729
Ubb	Ubb-002	0.0321416	1.28407	1.28407
Ubb	Ubb-001	0.0103802	1.29545	1.29545
Ubc	Ubc-002	0.0117757	2.20056	2.20056
Ube2b	Ube2b-001	0.00385789	0.820047	-1.21944
Ube2c	Ube2c-002	0.00359668	2.55297	2.55297
Ube2d3	Ube2d3-001	0.00230296	0.462908	-2.16025
Ube2d3	Ube2d3-012	0.00167573	5.37441	5.37441
Ube2e1	Ube2e1-001	0.0146407	1.34277	1.34277
Ube2e2	Ube2e2-012	0.0146007	0.162701	-6.14626
Ube2g2	Ube2g2-004	0.0341892	3.84447	3.84447
Ube2h	Ube2h-004	0.0395623	251.441	251.441
Ube2i	Ube2i-010	0.0432468	1.48791	1.48791
Ube2k	Ube2k-003	0.0385268	0.206461	-4.84353
Ube2k	Ube2k-007	0.0436554	0.254443	-3.93016
Ube2k	Ube2k-010	0.0154817	0.473965	-2.10986
Ube2k	Ube2k-001	0.000233198	0.513529	-1.94731
Ube2k	Ube2k-004	0.0413396	1.28572	1.28572
Ube2k	Ube2k-002	0.0341735	1.41119	1.41119
Ube2l3	Ube2l3-003	0.0108461	1.35753	1.35753
Ube2l6	Ube2l6-001	0.00126801	0.749191	-1.33477
Ube2m	Ube2m-003	0.0437846	1.38244	1.38244
Ube2o	Ube2o-001	0.000674016	1.3189	1.3189
Ube2q1	Ube2q1-002	0.000197931	1.25866	1.25866
Ube2r2	Ube2r2-003	0.000166784	0.645249	-1.54979
Ube2r2	Ube2r2-001	0.000474457	0.670543	-1.49133
Ube2v1	Ube2v1-001	0.0129317	1.55426	1.55426
Ube2v2	Ube2v2-001	0.0117411	0.744584	-1.34303
Ube2w	Ube2w-001	6.97744e-05	1.49938	1.49938
Ube2w	Ube2w-006	0.00216206	2.3584	2.3584
Ube3b	Ube3b-001	0.0348326	1.187	1.187
Ube3b	Ube3b-004	0.0480259	2816.12	2816.12
Ube4b	Ube4b-009	0.0147403	5.2963	5.2963
Ubfd1	Ubfd1-002	0.00319645	1.56288	1.56288
Ubfd1	Ubfd1-005	0.0196372	2.03876	2.03876
Ubfd1	Ubfd1-004	0.0202864	8.77086	8.77086
Ubl3	Ubl3-201	0.0385036	2.01465	2.01465
Ubl5	Ubl5-007	0.0188151	3.12139	3.12139
Ubl7	Ubl7-201	0.00254883	1.26963	1.26963
Ubn2	Ubn2-001	0.00947472	0.826661	-1.20969

Ubox5	Ubox5-001	0.0334351	1.52288	1.52288
Ubqln1	Ubqln1-201	0.00214162	1.36168	1.36168
Ubr2	Ubr2-202	0.00256287	1.43329	1.43329
Ubr3	Ubr3-201	0.0148229	1.4852	1.4852
Ubtf	Ubtf-201	0.0410806	0.525547	-1.90278
Ubtfl1	Ubtfl1-001	0.00115679	0.296714	-3.37025
Ubtfl1	Ubtfl1-002	0.0011568	0.296714	-3.37025
Ubxn4	Ubxn4-001	0.0290974	1.10053	1.10053
Ubxn4	Ubxn4-004	0.0311094	2.2468	2.2468
Ubxn4	Ubxn4-003	0.02188	2.25263	2.25263
Uchl1os	Uchl1os-001	0.0243193	2.90161	2.90161
Uck2	Uck2-008	0.00177247	1.82009	1.82009
Uck2	Uck2-002	0.00157431	1.93447	1.93447
Uck2	Uck2-009	0.00025645	1.9914	1.9914
Uckl1	Uckl1-017	0.0389982	0.158428	-6.31201
Uckl1	Uckl1-009	0.0338018	0.435517	-2.29612
Uckl1	Uckl1-012	0.00179423	0.497492	-2.01008
Uckl1	Uckl1-001	0.0467944	0.573961	-1.74228
Ucp2	Ucp2-002	0.0441005	0.410468	-2.43625
Ufc1	Ufc1-005	0.0298872	1.59448	1.59448
Ufc1	Ufc1-004	0.0083991	2.07163	2.07163
Ufl1	Ufl1-003	0.0476569	1.33022	1.33022
Ufsp2	Ufsp2-001	0.00851785	1.41666	1.41666
Ugdh	Ugdh-004	0.028297	0.797728	-1.25356
Uggt1	Uggt1-002	0.00109668	1.89022	1.89022
Uggt1	Uggt1-007	0.00042299	7.00865	7.00865
Uhrf1	Uhrf1-001	0.0165628	0.582852	-1.7157
Uhrf1	Uhrf1-007	0.00968302	0.622404	-1.60667
Uhrf2	Uhrf2-003	0.0395491	1.45351	1.45351
Ulbp1	Ulbp1-202	0.0104582	0.26921	-3.71457
Umad1	Umad1-005	0.0466945	0.56727	-1.76283
Unc119	Unc119-005	0.0186482	0.0915918	-10.918
Unc119b	Unc119b-001	0.0148292	1.2097	1.2097
Unc119b	Unc119b-002	0.0244531	1.93055	1.93055
Unc13a	Unc13a-001	2.08996e-05	0.616978	-1.6208
Unc13a	Unc13a-006	0.00206156	0.692344	-1.44437
Unc13b	Unc13b-013	0.00817966	4.95456	4.95456
Unc45a	Unc45a-007	0.0493384	2.7247	2.7247
Unc50	Unc50-004	0.0155739	2.00759	2.00759
Unc5c	Unc5c-001	0.0125929	0.717563	-1.39361
Unc80	Unc80-001	0.0273298	1.13021	1.13021
Unc80	Unc80-004	0.00143103	2.91189	2.91189
Unc93b1	Unc93b1-003	0.00967924	0.507162	-1.97176

Upf3a	Upf3a-201	0.00859198	1.22028	1.22028
Uqcc1	Uqcc1-001	0.0276636	0.474811	-2.1061
Uqcc1	Uqcc1-011	0.000520164	5.55838	5.55838
Uqcc2	Uqcc2-001	0.0031701	1.23567	1.23567
Uqcrb	Uqcrb-001	0.00244896	1.34091	1.34091
Uqcrc2	Uqcrc2-001	0.0309833	1.08934	1.08934
Uqcrc2	Uqcrc2-003	0.0474656	8.24575	8.24575
Uri1	Uri1-003	0.0172922	1.47466	1.47466
Urm1	Urm1-001	0.0267174	1.34805	1.34805
Urm1	Urm1-002	0.00523015	2.50951	2.50951
Urod	Urod-001	0.0184336	0.637399	-1.56888
Uros	Uros-004	0.0218402	1.58086	1.58086
Usb1	Usb1-004	0.0300601	0.3226	-3.09981
Usb1	Usb1-001	0.00768631	1.30808	1.30808
Ushbp1	Ushbp1-201	0.0353727	0.60259	-1.6595
Uso1	Uso1-001	0.00132888	1.34978	1.34978
Uso1	Uso1-004	0.00527942	1.88101	1.88101
Uso1	Uso1-002	0.00347796	2.89873	2.89873
Usp10	Usp10-004	0.0215726	1.46572	1.46572
Usp15	Usp15-201	0.0499207	1.21637	1.21637
Usp21	Usp21-008	0.0461865	0.205921	-4.85623
Usp21	Usp21-010	0.0121922	1.46427	1.46427
Usp3	Usp3-010	0.0282859	0.215465	-4.64113
Usp3	Usp3-003	0.0325773	0.834572	-1.19822
Usp34	Usp34-004	0.048777	1.33897e-08	-7.46843e+07
Usp34	Usp34-003	0.0258451	0.330101	-3.02938
Usp36	Usp36-005	0.0144703	1.6343	1.6343
Usp36	Usp36-003	0.0104278	1.83225	1.83225
Usp37	Usp37-001	0.00234688	3.13298	3.13298
Usp4	Usp4-001	0.026401	1.26698	1.26698
Usp40	Usp40-003	0.00955196	1.3976	1.3976
Usp40	Usp40-008	0.0484858	2.61308	2.61308
Usp43	Usp43-004	0.00636171	0.394912	-2.53221
Usp45	Usp45-007	0.0135688	66.4619	66.4619
Usp46	Usp46-004	0.0313767	0.0702283	-14.2393
Usp48	Usp48-017	0.00218033	0.260541	-3.83817
Usp48	Usp48-010	0.0396063	1.6434	1.6434
Usp48	Usp48-003	0.00789475	2.66818	2.66818
Usp49	Usp49-001	0.0321021	0.761642	-1.31295
Usp49	Usp49-004	0.00808928	56.581	56.581
Usp53	Usp53-001	0.00774041	1.15876	1.15876
Usp7	Usp7-001	0.0292123	1.62295	1.62295
Usp8	Usp8-002	0.049944	1.27376	1.27376

Uspl1	Uspl1-007	0.0134484	0.333744	-2.9963
Uspl1	Uspl1-005	0.0021623	1.6094	1.6094
Utp11l	Utp11l-002	0.0349495	1.29999	1.29999
Utp6	Utp6-002	0.00117602	11.5433	11.5433
Uvrag	Uvrag-201	0.000260599	1.34801	1.34801
Uxs1	Uxs1-001	0.00372249	0.605053	-1.65275
Vamp2	Vamp2-004	0.0469937	0.492963	-2.02855
Vamp2	Vamp2-001	0.0184483	1.15325	1.15325
Vamp3	Vamp3-001	0.00583896	0.92875	-1.07672
Vamp4	Vamp4-002	0.00430959	1.26292	1.26292
Vamp5	Vamp5-002	0.000616481	0.314215	-3.18253
Vamp7-ps	Vamp7-ps-001	0.0470094	1.32953	1.32953
Vamp8	Vamp8-001	0.00383961	0.71365	-1.40125
Vangl2	Vangl2-001	0.00195467	0.751588	-1.33052
Vangl2	Vangl2-002	0.00358222	6.66668	6.66668
Vapa	Vapa-201	0.00307953	1.16644	1.16644
Vapb	Vapb-002	0.0114355	1.31082	1.31082
Vars	Vars-010	0.0170114	2.66809	2.66809
Vars2	Vars2-012	0.0295941	613606	613606
Vat1	Vat1-001	0.00530728	0.761602	-1.31302
Vat1l	Vat1l-004	0.0264357	0.66433	-1.50528
Vat1l	Vat1l-001	0.0065619	0.767228	-1.30339
Vcan	Vcan-001	0.00958152	0.355044	-2.81655
Vcan	Vcan-004	0.00887541	0.457583	-2.1854
Vcl	Vcl-201	0.0406816	0.807243	-1.23878
Vcpkmt	Vcpkmt-201	0.00581084	1.22033	1.22033
Vdac2	Vdac2-006	0.0229237	0.350745	-2.85108
Vdac2	Vdac2-007	0.00507957	0.692783	-1.44345
Vdac3	Vdac3-202	0.00161437	1.20462	1.20462
Vdac3	Vdac3-201	0.000175116	1.41975	1.41975
Vdr	Vdr-001	1.14582e-06	0.540244	-1.85102
Vezf1	Vezf1-002	0.0167402	0.51358	-1.94712
Vgf	Vgf-003	0.0451497	0.533298	-1.87512
Vgll4	Vgll4-004	0.033189	7.78638e-08	-1.28429e+07
Vgll4	Vgll4-007	0.00782997	0.180413	-5.54283
Vgll4	Vgll4-001	0.0222843	0.658132	-1.51945
Vgll4	Vgll4-002	0.00727717	0.790512	-1.265
Vim	Vim-001	0.00299197	0.478952	-2.08789
Vim	Vim-004	0.00200812	0.484569	-2.06369
Vim	Vim-006	0.0013928	0.490024	-2.04072
Vim	Vim-005	0.0332695	0.541787	-1.84574
Vmn1r-ps60	Vmn1r-ps60-001	0.0458406	0.695816	-1.43716
Vmn2r46	Vmn2r46-001	0.0187369	0.282117	-3.54462

Vmp1	Vmp1-001	0.0444507	1.1611	1.1611
Vps11	Vps11-201	0.00685227	1.18042	1.18042
Vps13a	Vps13a-201	0.0102664	0.777321	-1.28647
Vps13c	Vps13c-201	0.0382654	1.15944	1.15944
Vps16	Vps16-001	0.000276524	0.699909	-1.42876
Vps16	Vps16-003	0.00793567	1.6825	1.6825
Vps26a	Vps26a-202	0.0104819	1.13963	1.13963
Vps26b	Vps26b-201	0.00691608	1.215	1.215
Vps29	Vps29-001	0.00300381	0.508704	-1.96578
Vps29	Vps29-009	0.0352	0.783312	-1.27663
Vps33b	Vps33b-001	0.0408782	0.750995	-1.33157
Vps35	Vps35-201	7.48641e-05	1.15501	1.15501
Vps36	Vps36-001	0.00569586	1.30975	1.30975
Vps39	Vps39-009	0.0345509	3.04814	3.04814
Vps4a	Vps4a-001	0.000945795	1.23113	1.23113
Vps4b	Vps4b-002	0.00114801	0.457522	-2.18568
Vps4b	Vps4b-001	0.00515045	1.11787	1.11787
Vps51	Vps51-008	0.0469343	0.229332	-4.3605
Vps51	Vps51-004	0.00102488	2.35436	2.35436
Vps52	Vps52-003	0.0183015	0.410647	-2.43518
Vps8	Vps8-003	3.80953e-05	1.63884e-11	-6.10186e+10
Vrk3	Vrk3-001	0.0128034	0.654707	-1.5274
Vrk3	Vrk3-004	0.0370007	4.85233	4.85233
Vsig1	Vsig1-002	0.0340401	1.94617	1.94617
Vta1	Vta1-006	0.00839817	1.3032	1.3032
Vwa3a	Vwa3a-201	0.0312784	0.046989	-21.2816
Vwa3a	Vwa3a-001	0.0312785	0.046989	-21.2816
Vwa3a	Vwa3a-002	0.0312785	0.0469889	-21.2816
Vwa5b2	Vwa5b2-201	0.0415557	0.654686	-1.52745
Vwa5b2	Vwa5b2-005	0.0455542	1.88692	1.88692
Vwa5b2	Vwa5b2-008	0.0298422	3.75396	3.75396
Vwa7	Vwa7-001	0.00268913	0.610273	-1.63861
Vwa9	Vwa9-201	0.0318711	2.34641	2.34641
Vwf	Vwf-008	0.0239585	2.27725	2.27725
Wac	Wac-005	0.00512101	1.23309	1.23309
Wac	Wac-008	0.00226535	2.27958	2.27958
Wac	Wac-012	0.00249251	2.54749	2.54749
Wac	Wac-015	0.0147447	13.5535	13.5535
Wapl	Wapl-001	0.0199833	0.764871	-1.30741
Wapl	Wapl-201	0.00872796	2.05648	2.05648
Wapl	Wapl-003	0.0227805	2.12989	2.12989
Wars	Wars-001	2.51164e-05	1.60241	1.60241
Wars	Wars-007	0.0371607	1.77641	1.77641

Wars	Wars-003	0.00127067	1.79847	1.79847
Wasf1	Wasf1-002	0.0184409	1.24101	1.24101
Wasf3	Wasf3-001	3.14482e-05	1.35597	1.35597
Wasl	Wasl-001	0.00716251	1.19185	1.19185
Wbp1	Wbp1-007	0.0432904	0.580035	-1.72404
Wbp1	Wbp1-005	0.0019584	10.7923	10.7923
Wbp11	Wbp11-001	0.0294173	1.29628	1.29628
Wbp1l	Wbp1l-002	0.0488751	0.667098	-1.49903
Wbp2	Wbp2-001	0.0314817	1.33314	1.33314
Wbp5	Wbp5-001	0.0032831	1.32678	1.32678
Wdfy1	Wdfy1-008	0.0136484	0.306245	-3.26536
Wdfy1	Wdfy1-009	0.0163317	0.417514	-2.39513
Wdfy1	Wdfy1-002	0.0170089	1.82191	1.82191
Wdfy1	Wdfy1-013	0.015842	12.2095	12.2095
Wdr1	Wdr1-001	0.00166004	0.647929	-1.54338
Wdr11	Wdr11-001	0.0278732	1.14443	1.14443
Wdr17	Wdr17-002	0.0127701	2.14336	2.14336
Wdr17	Wdr17-004	0.0233577	2.23278	2.23278
Wdr19	Wdr19-001	0.0139753	0.727114	-1.3753
Wdr20	Wdr20-002	0.0269723	1.18414	1.18414
Wdr26	Wdr26-002	0.00287137	0.647047	-1.54548
Wdr26	Wdr26-003	0.0363387	1.34582	1.34582
Wdr38	Wdr38-003	0.0299049	2.28268	2.28268
Wdr41	Wdr41-201	0.0117287	1.34174	1.34174
Wdr45	Wdr45-008	0.0337349	29121.5	29121.5
Wdr45b	Wdr45b-001	0.00542247	1.18078	1.18078
Wdr5	Wdr5-001	0.0264724	1.14212	1.14212
Wdr53	Wdr53-001	0.0315627	1.7215	1.7215
Wdr53	Wdr53-002	0.0166014	4.33143	4.33143
Wdr5b	Wdr5b-201	0.0479639	1.66254	1.66254
Wdr60	Wdr60-201	0.0225116	1.60506	1.60506
Wdr61	Wdr61-001	0.0463098	1.33079	1.33079
Wdr70	Wdr70-201	0.00253644	1.2801	1.2801
Wdr76	Wdr76-005	0.0330594	15.6303	15.6303
Wdr77	Wdr77-002	0.0284772	0.758197	-1.31892
Wdr77	Wdr77-003	0.0364913	2.16677	2.16677
Wdr83	Wdr83-001	0.00223233	0.644816	-1.55083
Wdr83	Wdr83-003	0.0340285	2.29176	2.29176
Wdr90	Wdr90-004	0.0490367	1.50865	1.50865
Wdr90	Wdr90-011	0.0376387	2.0721	2.0721
Wdr90	Wdr90-005	0.00890461	3.77053	3.77053
Wdr92	Wdr92-002	0.0375248	2.28963	2.28963
Wfikkn2	Wfikkn2-001	0.0124459	1.67105	1.67105

Wfs1	Wfs1-001	0.007977	1.51321	1.51321
Whamm	Whamm-201	0.0429413	1.24497	1.24497
Whsc1	Whsc1-006	0.0102398	0.309056	-3.23566
Whsc1	Whsc1-007	0.0119343	0.322738	-3.09848
Whsc1	Whsc1-201	0.00602922	0.391686	-2.55306
Whsc1	Whsc1-005	0.0186292	0.65245	-1.53268
Whsc1	Whsc1-003	0.00921604	1.49017	1.49017
Whsc1	Whsc1-002	0.00151763	1.52556	1.52556
Whsc1l1	Whsc1l1-201	0.00463695	0.727881	-1.37385
Whsc1l1	Whsc1l1-005	0.00174181	0.743128	-1.34566
Whsc1l1	Whsc1l1-006	0.0198575	2.13529	2.13529
Wipf3	Wipf3-202	0.00552978	0.610693	-1.63748
Wipi2	Wipi2-003	0.000112304	1.57638	1.57638
Wipi2	Wipi2-005	0.0189914	2.10665	2.10665
Wisp2	Wisp2-001	0.0131096	7.47655	7.47655
Wiz	Wiz-007	0.0137125	1.47282	1.47282
Wnk1	Wnk1-016	0.0166409	0.398968	-2.50647
Wnk1	Wnk1-002	0.0247838	0.483042	-2.07021
Wnk1	Wnk1-015	0.0271943	0.509797	-1.96157
Wnk1	Wnk1-023	0.0423818	0.722593	-1.38391
Wnk1	Wnk1-022	0.0354232	0.802893	-1.2455
Wnk1	Wnk1-021	0.0227983	0.853688	-1.17139
Wnt3	Wnt3-001	0.0299219	1.51304	1.51304
Wnt9a	Wnt9a-001	0.030409	2.51593	2.51593
Wrb	Wrb-002	0.0350288	0.368436	-2.71417
Wrb	Wrb-001	0.005982	0.437076	-2.28793
Wrn	Wrn-202	0.00206728	0.686611	-1.45643
Wrnip1	Wrnip1-001	0.0126102	1.06837	1.06837
Wsb1	Wsb1-003	0.0489834	0.497126	-2.01156
Wtap	Wtap-005	0.0100647	0.0864794	-11.5634
Wwox	Wwox-003	0.048575	6.73357	6.73357
Wwp1	Wwp1-201	0.0323841	1.42933	1.42933
Xbp1	Xbp1-001	0.0284623	1.3312	1.3312
Xiap	Xiap-005	0.00900318	0.752779	-1.32841
Xkr6	Xkr6-002	0.0379857	5.42969	5.42969
Xkr8	Xkr8-001	0.025825	1.18313	1.18313
Xkr8	Xkr8-002	0.00607688	6.89092	6.89092
Xlr3a	Xlr3a-002	0.000810653	1.47658	1.47658
Xlr3c	Xlr3c-001	3.54087e-05	1.40985	1.40985
Xlr4a	Xlr4a-002	0.0460399	2.15252	2.15252
Xlr4b	Xlr4b-002	0.0150975	1.35894	1.35894
Xndc1	Xndc1-006	0.00476899	4.93134	4.93134
Xpnpep1	Xpnpep1-002	0.0390778	1.16914	1.16914

Xpo1	Xpo1-202	0.048412	0.661609	-1.51147
Xpo4	Xpo4-002	0.0290305	4.98192	4.98192
Xpo6	Xpo6-005	0.0191573	0.322744	-3.09843
Xpo6	Xpo6-012	0.0492628	1.80669	1.80669
Xpo6	Xpo6-011	0.013843	5.1786	5.1786
Xpo7	Xpo7-201	0.0351261	1.25579	1.25579
Xpot	Xpot-201	0.018256	1.34095	1.34095
Xpr1	Xpr1-001	0.000872811	0.586723	-1.70438
Xrcc6	Xrcc6-001	0.00462554	1.31832	1.31832
Xrcc6bp1	Xrcc6bp1-201	0.0406473	0.49686	-2.01264
Xrcc6bp1	Xrcc6bp1-202	0.0339819	1.58142	1.58142
Xrn2	Xrn2-001	0.0161856	1.11689	1.11689
Xrra1	Xrra1-201	0.000763645	2.26631	2.26631
Xylb	Xylb-201	0.0497953	1.67047	1.67047
Xylt2	Xylt2-001	0.0478795	1.28201	1.28201
Yars	Yars-001	0.00043946	1.24742	1.24742
Yars	Yars-003	0.0182333	1.65753	1.65753
Ybx3	Ybx3-004	0.0292242	0.732895	-1.36445
Yipf3	Yipf3-001	0.0236696	1.40459	1.40459
Yipf3	Yipf3-005	0.00189482	1.81183	1.81183
Yipf5	Yipf5-201	0.0486144	1.24323	1.24323
Yjefn3	Yjefn3-001	0.000215617	0.62187	-1.60805
Ylpm1	Ylpm1-001	0.0284067	0.787813	-1.26934
Yme1l1	Yme1l1-001	0.00339651	1.27966	1.27966
Yod1	Yod1-001	0.000345196	1.50476	1.50476
Ythdc1	Ythdc1-002	0.031839	1.83732	1.83732
Ythdf1	Ythdf1-002	0.0169691	6.27298	6.27298
Ywhae	Ywhae-001	0.0278654	1.08701	1.08701
Ywhah	Ywhah-001	0.00734342	0.892175	-1.12086
Ywhaq	Ywhaq-002	0.0329298	0.637451	-1.56875
Ywhaz	Ywhaz-002	0.0135712	1.08034	1.08034
Zadh2	Zadh2-201	0.0154366	0.730613	-1.36871
Zbtb11	Zbtb11-001	0.0261203	1.23175	1.23175
Zbtb20	Zbtb20-014	0.00379706	0.245675	-4.07042
Zbtb24	Zbtb24-201	0.0321985	0.861818	-1.16034
Zbtb34	Zbtb34-002	0.0314973	4.22166	4.22166
Zbtb37	Zbtb37-004	0.0260319	39.2666	39.2666
Zbtb38	Zbtb38-201	0.00841852	0.568533	-1.75891
Zbtb4	Zbtb4-005	0.0381312	0.109394	-9.14128
Zbtb44	Zbtb44-202	0.0191789	1.41104	1.41104
Zbtb46	Zbtb46-001	0.00491132	0.646523	-1.54673
Zbtb46	Zbtb46-002	0.00892011	1.5126	1.5126
Zbtb48	Zbtb48-007	0.0470058	3.75616	3.75616

Zbtb49	Zbtb49-007	0.0155792	1.64761	1.64761
Zbtb7a	Zbtb7a-001	0.0210576	0.653859	-1.52938
Zbtb7c	Zbtb7c-201	0.000514415	1.80274	1.80274
Zc3h11a	Zc3h11a-006	0.0262988	0.668368	-1.49618
Zc3h11a	Zc3h11a-002	0.0133561	0.75493	-1.32463
Zc3h14	Zc3h14-204	0.0348575	1.19221	1.19221
Zc3h15	Zc3h15-001	0.00969672	1.22912	1.22912
Zc3h3	Zc3h3-201	0.00160614	1.25902	1.25902
Zc3h7a	Zc3h7a-011	0.00650067	1.4688	1.4688
Zc3h7a	Zc3h7a-016	0.0472991	1.48417	1.48417
Zc3h8	Zc3h8-001	0.00302605	1.80082	1.80082
Zc3hav1	Zc3hav1-003	0.0011016	2.44834	2.44834
Zc3hc1	Zc3hc1-002	0.0210072	1.45737	1.45737
Zc3hc1	Zc3hc1-007	1.52968e-05	3.95815	3.95815
Zcchc11	Zcchc11-008	0.0342716	355159	355159
Zcchc12	Zcchc12-001	0.0161134	1.18018	1.18018
Zcchc12	Zcchc12-004	0.0148986	1.66075	1.66075
Zcchc14	Zcchc14-001	0.0304601	0.743691	-1.34465
Zcchc17	Zcchc17-003	0.0204136	1.90958	1.90958
Zcchc2	Zcchc2-001	0.0377421	1.40331	1.40331
Zcchc4	Zcchc4-003	0.00489024	0.705433	-1.41757
Zcchc9	Zcchc9-001	0.0127539	1.22529	1.22529
Zcrb1	Zcrb1-008	0.0277121	1.80032	1.80032
Zcwpw1	Zcwpw1-002	0.0408743	0.186353	-5.36616
Zdhhc12	Zdhhc12-001	0.00439031	0.55272	-1.80923
Zdhhc13	Zdhhc13-001	0.00117152	1.35362	1.35362
Zdhhc17	Zdhhc17-201	0.00964182	0.852765	-1.17266
Zdhhc18	Zdhhc18-004	0.0456231	2.00648	2.00648
Zdhhc2	Zdhhc2-001	0.0256323	0.700111	-1.42834
Zdhhc2	Zdhhc2-201	0.0199449	1.74549	1.74549
Zdhhc20	Zdhhc20-201	3.17096e-05	0.543068	-1.84139
Zdhhc21	Zdhhc21-001	5.83623e-06	0.620007	-1.61289
Zdhhc23	Zdhhc23-201	0.00616652	3.1817	3.1817
Zdhhc3	Zdhhc3-001	0.00363903	1.49352	1.49352
Zdhhc3	Zdhhc3-005	0.0451455	8.87977	8.87977
Zdhhc7	Zdhhc7-002	0.0442157	3.01937	3.01937
Zeb1	Zeb1-003	0.0120735	1.6088	1.6088
Zeb2	Zeb2-006	0.00817849	0.214789	-4.65574
Zeb2	Zeb2-001	0.0434188	0.484074	-2.0658
Zeb2	Zeb2-005	0.0307881	0.613091	-1.63108
Zfand4	Zfand4-201	0.0480691	1.67014	1.67014
Zfand5	Zfand5-003	0.00177671	0.744827	-1.34259
Zfc3h1	Zfc3h1-001	0.000870016	1.42273	1.42273

Zfhx2	Zfhx2-004	0.00580204	1.62206	1.62206
Zfp106	Zfp106-002	0.0431155	1.84983	1.84983
Zfp106	Zfp106-005	0.00279427	4.90209	4.90209
Zfp106	Zfp106-011	0.0063668	5.8526	5.8526
Zfp119b	Zfp119b-001	0.0242304	1.54126	1.54126
Zfp131	Zfp131-202	0.00526654	1.49855	1.49855
Zfp133-ps	Zfp133-ps-003	0.00305399	1.66663	1.66663
Zfp146	Zfp146-001	0.0269897	1.30494	1.30494
Zfp148	Zfp148-202	0.00579235	0.723881	-1.38144
Zfp180	Zfp180-003	0.0180394	1.70095	1.70095
Zfp180	Zfp180-005	0.00195568	1.94119	1.94119
Zfp182	Zfp182-004	0.0295697	0.396608	-2.52138
Zfp184	Zfp184-004	0.0482827	1.6641	1.6641
Zfp207	Zfp207-003	0.0397386	0.900561	-1.11042
Zfp207	Zfp207-001	0.0158116	1.61102	1.61102
Zfp207	Zfp207-006	0.00640716	6.86559	6.86559
Zfp212	Zfp212-001	0.00518506	1.20233	1.20233
Zfp217	Zfp217-201	0.00297811	0.680389	-1.46975
Zfp219	Zfp219-201	0.00551511	0.568199	-1.75995
Zfp219	Zfp219-202	0.0275898	1.44414	1.44414
Zfp236	Zfp236-002	0.00433039	0.565246	-1.76914
Zfp236	Zfp236-001	0.0190088	1.39477	1.39477
Zfp260	Zfp260-001	0.00718065	0.655513	-1.52552
Zfp260	Zfp260-002	0.000250143	1.30838	1.30838
Zfp275	Zfp275-001	0.00251599	0.6724	-1.48721
Zfp277	Zfp277-201	0.00376234	0.724359	-1.38053
Zfp280c	Zfp280c-002	0.0368127	1.30735	1.30735
Zfp287	Zfp287-001	0.000410943	1.88403	1.88403
Zfp287	Zfp287-005	0.0127754	4.03301	4.03301
Zfp317	Zfp317-201	0.00270973	1.2214	1.2214
Zfp322a	Zfp322a-004	0.0466647	1.92906	1.92906
Zfp329	Zfp329-001	0.0276923	1.31373	1.31373
Zfp330	Zfp330-201	0.00437544	1.39215	1.39215
Zfp335	Zfp335-001	0.0290203	1.16957	1.16957
Zfp335os	Zfp335os-001	0.0133562	2.78441	2.78441
Zfp35	Zfp35-001	0.0478018	1.54151	1.54151
Zfp354c	Zfp354c-001	0.00227271	0.753304	-1.32749
Zfp362	Zfp362-201	0.00932778	0.601609	-1.66221
Zfp365	Zfp365-001	0.00025925	1.4528	1.4528
Zfp367	Zfp367-001	0.0189304	0.843981	-1.18486
Zfp369	Zfp369-001	0.00382517	1.32199	1.32199
Zfp369	Zfp369-003	0.0232286	2.9921	2.9921
Zfp36l2	Zfp36l2-001	0.0274071	0.740441	-1.35055

Zfp385c	Zfp385c-001	0.0345851	2.10686	2.10686
Zfp386	Zfp386-003	0.00178754	2.46037	2.46037
Zfp397	Zfp397-201	0.0231365	1.38042	1.38042
Zfp420	Zfp420-003	0.000229554	2.92012	2.92012
Zfp420	Zfp420-002	0.00131241	2.99693	2.99693
Zfp422	Zfp422-004	0.0235203	12.3628	12.3628
Zfp433	Zfp433-001	0.0142291	1.31434	1.31434
Zfp438	Zfp438-201	0.00084325	1.56699	1.56699
Zfp444	Zfp444-002	0.000694771	1.50454	1.50454
Zfp446	Zfp446-005	0.040071	1.39619	1.39619
Zfp451	Zfp451-002	0.0138841	0.750185	-1.333
Zfp451	Zfp451-001	0.00452617	1.33295	1.33295
Zfp462	Zfp462-001	0.00611559	0.811584	-1.23216
Zfp467	Zfp467-001	0.00807676	0.630595	-1.58581
Zfp467	Zfp467-009	0.0160351	6.88995	6.88995
Zfp473	Zfp473-006	0.0157844	8.45476	8.45476
Zfp51	Zfp51-201	0.0418482	1.3106	1.3106
Zfp511	Zfp511-201	0.023568	1.19277	1.19277
Zfp512	Zfp512-008	0.019135	2.78264	2.78264
Zfp518b	Zfp518b-001	0.0307181	0.527547	-1.89557
Zfp526	Zfp526-001	0.00510662	1.19216	1.19216
Zfp532	Zfp532-002	0.0156079	0.434161	-2.30329
Zfp532	Zfp532-003	0.00620888	0.478564	-2.08959
Zfp560	Zfp560-002	0.00797771	1.78854	1.78854
Zfp579	Zfp579-002	0.0123643	1.3001	1.3001
Zfp579	Zfp579-003	0.0316618	3.15861	3.15861
Zfp580	Zfp580-201	0.0119682	1.56513	1.56513
Zfp597	Zfp597-003	0.00832457	4.17004	4.17004
Zfp598	Zfp598-201	9.42774e-07	1.41798	1.41798
Zfp606	Zfp606-004	0.0176369	1.80067	1.80067
Zfp607	Zfp607-001	0.00677956	1.25418	1.25418
Zfp62	Zfp62-201	0.00477928	0.656874	-1.52236
Zfp62	Zfp62-010	0.0422719	1.77576	1.77576
Zfp626	Zfp626-201	0.0142475	9.48277	9.48277
Zfp637	Zfp637-001	0.023702	0.549584	-1.81956
Zfp637	Zfp637-002	0.0480388	1.85423	1.85423
Zfp638	Zfp638-007	0.0400566	6.86618	6.86618
Zfp639	Zfp639-007	0.0128155	1.38143	1.38143
Zfp639	Zfp639-201	0.0358356	1.39792	1.39792
Zfp64	Zfp64-001	0.012943	0.676147	-1.47897
Zfp64	Zfp64-002	0.027214	1.31817	1.31817
Zfp652	Zfp652-003	0.0161398	0.197429	-5.0651
Zfp652	Zfp652-201	0.000356369	2.35319	2.35319

Zfp667	Zfp667-001	0.0398079	2.24501	2.24501
Zfp667	Zfp667-201	0.0352848	4.81705	4.81705
Zfp672	Zfp672-007	0.01311	1.86631	1.86631
Zfp689	Zfp689-002	0.0164326	2.00116	2.00116
Zfp704	Zfp704-001	0.0206177	0.821851	-1.21677
Zfp715	Zfp715-001	0.00771641	1.66872	1.66872
Zfp740	Zfp740-003	0.033578	0.666061	-1.50137
Zfp740	Zfp740-008	0.0321622	186.012	186.012
Zfp746	Zfp746-002	0.0466137	2.10832	2.10832
Zfp748	Zfp748-002	0.0127157	1.56915	1.56915
Zfp787	Zfp787-201	0.00327032	1.18484	1.18484
Zfp819	Zfp819-001	0.0478514	2.71911	2.71911
Zfp819	Zfp819-201	0.0478514	2.71911	2.71911
Zfp825	Zfp825-001	0.038612	1.23817	1.23817
Zfp862-ps	Zfp862-ps-002	0.0147235	2.60531	2.60531
Zfp865	Zfp865-202	0.0163037	1.44472	1.44472
Zfp874a	Zfp874a-001	0.00243669	1.69529	1.69529
Zfp874b	Zfp874b-001	0.0287512	1.2448	1.2448
Zfp91	Zfp91-003	0.0114112	3.53233	3.53233
Zfp930	Zfp930-201	0.00184662	1.34315	1.34315
Zfp932	Zfp932-001	0.0336924	1.3884	1.3884
Zfp932	Zfp932-005	0.0409856	2.77065	2.77065
Zfp935	Zfp935-201	0.0358644	1.26655	1.26655
Zfp938	Zfp938-002	0.021586	2.07544	2.07544
Zfp94	Zfp94-003	0.00388146	2.70278	2.70278
Zfp941	Zfp941-001	0.0286566	2.27906	2.27906
Zfp942	Zfp942-001	0.0171808	1.37283	1.37283
Zfp942	Zfp942-002	0.0252329	1.58646	1.58646
Zfp943	Zfp943-001	0.0114779	1.50661	1.50661
Zfp944	Zfp944-201	0.0121393	1.36005	1.36005
Zfp945	Zfp945-001	0.0365273	0.477264	-2.09528
Zfp945	Zfp945-201	0.0250618	0.717648	-1.39344
Zfp945	Zfp945-003	0.0161808	0.730443	-1.36903
Zfp946	Zfp946-002	0.0493377	0.598596	-1.67058
Zfp946	Zfp946-001	0.0498276	1.14675	1.14675
Zfp948	Zfp948-201	0.0173598	0.86387	-1.15758
Zfp950	Zfp950-002	0.0392116	1.45423	1.45423
Zfp955a	Zfp955a-201	0.0494534	1.27324	1.27324
Zfp956	Zfp956-001	0.0247071	1.51546	1.51546
Zfp961	Zfp961-201	0.00509536	9.84821	9.84821
Zfp964	Zfp964-002	0.0376999	1.78477	1.78477
Zfpl1	Zfpl1-010	0.034292	0.450191	-2.22128
Zfpl1	Zfpl1-007	0.00191993	13.0827	13.0827

Zfr	Zfr-001	0.000256981	1.30588	1.30588
Zfr2	Zfr2-006	0.0389866	1.44344	1.44344
Zfr2	Zfr2-002	0.0470807	1.61212	1.61212
Zfx	Zfx-201	0.0069356	1.76339	1.76339
Zfyve1	Zfyve1-201	0.00232817	0.791212	-1.26388
Zfyve16	Zfyve16-001	0.0485069	1.09499	1.09499
Zfyve19	Zfyve19-001	0.0470122	1.39855	1.39855
Zfyve26	Zfyve26-201	0.0209263	1.09122	1.09122
Zfyve27	Zfyve27-002	0.000368442	0.366469	-2.72874
Zfyve28	Zfyve28-005	0.00302494	12.3324	12.3324
Zhx3	Zhx3-201	0.000227307	1.8744	1.8744
Zkscan1	Zkscan1-201	0.00753815	0.405894	-2.4637
Zkscan2	Zkscan2-002	0.00238813	1.91854	1.91854
Zkscan8	Zkscan8-001	0.00414152	1.40423	1.40423
Zmynd11	Zmynd11-014	0.0363588	2.06614e-08	-4.83993e+07
Zmynd8	Zmynd8-013	0.0231305	1.30286	1.30286
Zmynd8	Zmynd8-010	0.00239993	1.34652	1.34652
Znhit1	Znhit1-002	0.0396575	1.31468	1.31468
Znrd1	Znrd1-002	0.00517748	1.95131	1.95131
Znrd1as	Znrd1as-001	0.00955109	1.49563	1.49563
Znrf1	Znrf1-203	0.0134578	0.28556	-3.50189
Znrf1	Znrf1-003	0.00241372	0.686733	-1.45617
Znrf1	Znrf1-002	0.00040824	0.703291	-1.42189
Znrf2	Znrf2-002	0.00130067	15.6736	15.6736
Zranb1	Zranb1-001	0.00108654	0.678725	-1.47335
Zranb2	Zranb2-004	0.0154469	1.31754	1.31754
Zranb2	Zranb2-001	0.0371552	1.52491	1.52491
Zranb2	Zranb2-006	0.0280898	2.71372	2.71372
Zranb3	Zranb3-001	0.03999	0.899396	-1.11186
Zranb3	Zranb3-002	0.0132354	3.41471	3.41471
Zrsr2	Zrsr2-004	0.0146624	1.27464	1.27464
Zscan10	Zscan10-008	0.0353337	647844	647844
Zscan20	Zscan20-006	0.0327055	0.438417	-2.28093
Zscan20	Zscan20-001	0.0042806	1.33393	1.33393
Zscan26	Zscan26-001	0.00385384	1.53108	1.53108
Zswim1	Zswim1-001	0.010891	1.34116	1.34116
Zwilch	Zwilch-005	0.00905122	0.323038	-3.09561
Zwilch	Zwilch-004	0.0208299	0.65683	-1.52246
Zwint	Zwint-003	0.0428366	0.748329	-1.33631
Zyg11a	Zyg11a-001	0.00751581	3.21361	3.21361
Zyg11a	Zyg11a-201	0.00751587	3.21361	3.21361
Zyx	Zyx-001	0.0461108	0.725084	-1.37915
Zzz3	Zzz3-001	0.00796284	0.686733	-1.45617

Supplementary Table S3

miR-132/212 network in 3xTg-AD mice	miR-132/212 network in Neuro2a cells	Common targets
adamts5	abr	aebp2
adgrl3	acsl4	anp32a
aebp2	aebp2	atp10d
anp32a	anp32a	bmpr1a
atp10d	ap1g1	brwd1
atp6v0a2	arhgap21	calu
bmpr1a	arhgap32	celsr3
bnip2	arhgef40	fam76b
brwd1	arid2	fkbp2
calu	arid4b	foxn3
caprin1	atp10d	gna12
cbx1	azin1	hbegf
cdkn1a	b4galt6	hnrnph1
celsr3	bcl2l11	kdm5a
ctdspl	bmpr1a	klhdc10
desi2	bri3	mapk1
eif4a2	brwd1	mapt
epb41	btbd7	meis2
esrrg	btg2	nfya
fam76b	c6orf106	pcgf3
fkbp2	calu	pik3r1
foxn3	camsap2	sephs1
foxo3	cc2d1b	sgk3
frk	ccdc71l	sirt1
gapvd1	cd164	smad5
gna12	cdk19	spred1
hbegf	celsr3	srgap2
hey1	cers2	srsf10
hira	cnih1	tmeff1
hnrnph1	cpsf6	trib2
isl1	crk	zeb2
kcnma1	csde1	zfyve1
kdm5a	csrnp3	
kdm5b	ctdspl2	
kdm7a	ctgf	
klhdc10	dag1	
mapk1	dazap2	
mapt	dnajb14	
mef2c	dnmt3a	
meis2	dock4	
mfap1	dpysl3	
mtf1	dusp9	
nab1	extl2	
ndrg4	fam76b	
nfya	fbxo28	

pcgf3
pik3r1
pou4f1
pprc1
ptch1
rab15
sept8
sephs1
setd5
sgk3
sirt1
slc2a1
slc6a1
smad5
sox11
spred1
srgap2
srsf1
srsf10
taf4
tceb1
tcf7l2
tex30
tmeff1
tmem64
trib2
zeb2
zfyve1
znf516

fbxw11
fgf7
fkbp2
fmr1
foxn3
fubp1
fxr1
gmfb
gna12
gnb1
gpd2
hbegf
hn1
hnrnph1
hnrnpm
homer1
kcmf1
kdm5a
klhdc10
kmt5b
kras
ksr2
large
lsm11
lzts3
map3k3
mapk1
mapt
mecp2
meis2
mepce
mex3a
mex3c
mia3
mycbp2
nfib
nfya
nmnat2
nmt2
nova1
nrep
nrn1
olfm1
osbpl8
papola
pcgf3
pea15
pfn2

pik3ip1
pik3r1
ppfibp1
ppp3ca
prickle2
ptbp2
ptpn4
purb
rapgef6
rbm12
sema6d
sephs1
sgk3
shoc2
sirt1
six4
slc12a6
slc24a3
slc44a1
smad5
snx18
sos1
sox4
sppl3
spred1
sptssa
srgap2
srgap3
srsf10
ssh2
sucla2
tcf4
tead1
tgfbi
tjap1
tln2
tmeff1
tmem2
tmem43
tmem50b
trib2
trim2
ube2e2
vdac2
whsc1l1
zeb2
zfyve1
znf148

znf236
znf362

Supplementary Table S4

miR-132/212 Network in 3xTg-AD mice

Category	ID	Name	p-value	Hit Count in Query List	Hit Count in Genome	Hit in Query List
GO: Molecular Function	GO:0003700	transcription factor activity, sequence-specific DNA binding	5.418E-9	21	1230	FOXO3,SOX11,KDM5B,BMPR1A,TCF7L2,HEY1,KDM5A,MTF1,ESRRG,ZNF516,NFYA,AEBP2,POU4F1,ISL1,FOXN3,TAF4,ZEB2,MEF2C,MEIS2,HIRA,SMAD5
GO: Molecular Function	GO:0001071	nucleic acid binding transcription factor activity	5.418E-9	21	1230	FOXO3,SOX11,KDM5B,BMPR1A,TCF7L2,HEY1,KDM5A,MTF1,ESRRG,ZNF516,NFYA,AEBP2,POU4F1,ISL1,FOXN3,TAF4,ZEB2,MEF2C,MEIS2,HIRA,SMAD5
GO: Molecular Function	GO:0008134	transcription factor binding	4.369E-8	14	577	FOXO3,TCF7L2,TRIB2,HEY1,PPRC1,PIK3R1,NAB1,ZNF516,NFYA,ISL1,MAPK1,MEF2C,SIRT1,MEIS2
GO: Molecular Function	GO:0001228	transcriptional activator activity, RNA polymerase II transcription regulatory region sequence-specific binding	1.345E-7	11	357	FOXO3,SOX11,TCF7L2,MTF1,ESRRG,NFYA,POU4F1,ISL1,ZEB2,MEF2C,MEIS2
GO: Molecular Function	GO:0000981	RNA polymerase II transcription factor activity, sequence-specific DNA binding	2.075E-7	14	655	FOXO3,SOX11,BMPR1A,TCF7L2,HEY1,MTF1,ESRRG,NFYA,AEBP2,POU4F1,ISL1,ZEB2,MEF2C,MEIS2
GO: Molecular Function	GO:0001047	core promoter binding	2.174E-7	8	161	FOXO3,SOX11,TCF7L2,MTF1,NFYA,MEF2C,SIRT1,SMAD5
GO: Molecular Function	GO:0001046	core promoter sequence-specific DNA binding	2.588E-7	7	111	SOX11,TCF7L2,MTF1,NFYA,MEF2C,SIRT1,SMAD5
GO: Molecular Function	GO:1990837	sequence-specific double-stranded DNA binding	6.600E-7	14	721	SOX11,TCF7L2,HEY1,MTF1,ESRRG,ZNF516,NFYA,AEBP2,POU4F1,ISL1,MEF2C,SIRT1,MEIS2,SMAD5
GO: Molecular Function	GO:0000982	transcription factor activity, RNA polymerase II core promoter proximal region sequence-specific binding	1.450E-6	10	363	SOX11,TCF7L2,HEY1,MTF1,NFYA,AEBP2,POU4F1,ISL1,MEF2C,MEIS2
GO: Molecular Function	GO:0000976	transcription regulatory region sequence-specific DNA binding	2.440E-6	13	690	SOX11,TCF7L2,MTF1,ESRRG,ZNF516,NFYA,AEBP2,POU4F1,ISL1,MEF2C,SIRT1,MEIS2,SMAD5
GO: Molecular Function	GO:0003690	double-stranded DNA binding	2.495E-6	14	807	SOX11,TCF7L2,HEY1,MTF1,ESRRG,ZNF516,NFYA,AEBP2,POU4F1,ISL1,MEF2C,SIRT1,MEIS2,SMAD5
GO: Molecular Function	GO:0043565	sequence-specific DNA binding	2.922E-6	16	1069	FOXO3,SOX11,TCF7L2,HEY1,MTF1,ESRRG,ZNF516,NFYA,AEBP2,POU4F1,ISL1,FOXN3,MEF2C,SIRT1,MEIS2,SMAD5
GO: Molecular Function	GO:0000988	transcription factor activity, protein binding	3.955E-6	12	609	SOX11,KDM5B,HEY1,KDM5A,MTF1,PPRC1,AEBP2,ISL1,TAF4,SIRT1,MEIS2,HIRA
GO: Molecular Function	GO:0044212	transcription regulatory region DNA binding	3.972E-6	14	840	FOXO3,SOX11,TCF7L2,MTF1,ESRRG,ZNF516,NFYA,AEBP2,POU4F1,ISL1,MEF2C,SIRT1,MEIS2,SMAD5
GO: Molecular Function	GO:0000975	regulatory region DNA binding	4.139E-6	14	843	FOXO3,SOX11,TCF7L2,MTF1,ESRRG,ZNF516,NFYA,AEBP2,POU4F1,ISL1,MEF2C,SIRT1,MEIS2,SMAD5
GO: Molecular Function	GO:0001067	regulatory region nucleic acid binding	4.312E-6	14	846	FOXO3,SOX11,TCF7L2,MTF1,ESRRG,ZNF516,NFYA,AEBP2,POU4F1,ISL1,MEF2C,SIRT1,MEIS2,SMAD5
GO: Molecular Function	GO:0001077	transcriptional activator activity, RNA polymerase II core promoter proximal region sequence-specific binding	7.692E-6	8	259	SOX11,TCF7L2,MTF1,NFYA,POU4F1,ISL1,MEF2C,MEIS2
GO: Molecular Function	GO:0003712	transcription cofactor activity	1.075E-5	11	560	SOX11,KDM5B,KDM5A,MTF1,PPRC1,AEBP2,ISL1,TAF4,SIRT1,MEIS2,HIRA
GO: Molecular Function	GO:0000989	transcription factor activity, transcription factor binding	2.205E-5	11	605	SOX11,KDM5B,KDM5A,MTF1,PPRC1,AEBP2,ISL1,TAF4,SIRT1,MEIS2,HIRA
GO: Molecular Function	GO:0000977	RNA polymerase II regulatory region sequence-specific DNA binding	2.721E-5	11	619	SOX11,TCF7L2,MTF1,ESRRG,NFYA,AEBP2,POU4F1,ISL1,MEF2C,MEIS2,SMAD5
GO: Molecular Function	GO:0001012	RNA polymerase II regulatory region DNA binding	3.016E-5	11	626	SOX11,TCF7L2,MTF1,ESRRG,NFYA,AEBP2,POU4F1,ISL1,MEF2C,MEIS2,SMAD5
GO: Molecular Function	GO:0000979	RNA polymerase II core promoter sequence-specific DNA binding	1.017E-4	4	62	SOX11,TCF7L2,MTF1,SMAD5
GO: Molecular Function	GO:0000987	core promoter proximal region sequence-specific DNA binding	1.585E-4	8	397	TCF7L2,MTF1,ZNF516,NFYA,AEBP2,ISL1,MEF2C,MEIS2
GO: Molecular Function	GO:0001159	core promoter proximal region DNA binding	1.641E-4	8	399	TCF7L2,MTF1,ZNF516,NFYA,AEBP2,ISL1,MEF2C,MEIS2
GO: Molecular Function	GO:0001205	transcriptional activator activity, RNA polymerase II distal enhancer sequence-specific binding	2.395E-4	3	31	POU4F1,ZEB2,MEF2C
GO: Molecular Function	GO:0000907	sulfonate dioxygenase activity	2.395E-4	3	31	KDM5B,KDM5A,KDM7A
GO: Molecular Function	GO:0052635	C-20 gibberellin 2-beta-dioxygenase activity	2.395E-4	3	31	KDM5B,KDM5A,KDM7A
GO: Molecular Function	GO:0034792	hypophosphite dioxygenase activity	2.395E-4	3	31	KDM5B,KDM5A,KDM7A
GO: Molecular Function	GO:0045543	gibberellin 2-beta-dioxygenase activity	2.395E-4	3	31	KDM5B,KDM5A,KDM7A
GO: Molecular Function	GO:0018602	2,4-dichlorophenoxyacetate alpha-ketoglutarate dioxygenase activity	2.395E-4	3	31	KDM5B,KDM5A,KDM7A
GO: Molecular Function	GO:0052634	C-19 gibberellin 2-beta-dioxygenase activity	2.395E-4	3	31	KDM5B,KDM5A,KDM7A
GO: Molecular Function	GO:0043734	DNA-N1-methyladenine dioxygenase activity	2.635E-4	3	32	KDM5B,KDM5A,KDM7A
GO: Molecular Function	GO:0050733	RS domain binding	3.137E-4	2	7	SRSF10,SRSF1
GO: Molecular Function	GO:0019798	procollagen-proline dioxygenase activity	4.407E-4	3	38	KDM5B,KDM5A,KDM7A
GO: Molecular Function	GO:0001158	enhancer sequence-specific DNA binding	4.475E-4	4	91	SOX11,POU4F1,ISL1,MEF2C
GO: Molecular Function	GO:0031543	peptidyl-proline dioxygenase activity	5.930E-4	3	42	KDM5B,KDM5A,KDM7A
GO: Molecular Function	GO:0019899	enzyme binding	6.548E-4	17	1851	CDKN1A,FOXO3,TCF7L2,TRIB2,MTF1,MAPT,CALU,PIK3R1,CBX1,SPRED1,SRGAP2,MAPK1,MEF2C,SLC2A1,SIRT1,ATP6V0A2,SMAD5
GO: Molecular Function	GO:0000978	RNA polymerase II core promoter proximal region sequence-specific DNA binding	6.891E-4	7	378	TCF7L2,MTF1,NFYA,AEBP2,ISL1,MEF2C,MEIS2
GO: Molecular Function	GO:0035326	enhancer binding	7.410E-4	4	104	SOX11,POU4F1,ISL1,MEF2C
GO: Molecular Function	GO:0003705	transcription factor activity, RNA polymerase II distal enhancer sequence-specific binding	7.681E-4	4	105	SOX11,POU4F1,ZEB2,MEF2C
GO: Molecular Function	GO:0044877	macromolecular complex binding	1.021E-3	15	1578	CDKN1A,FOXO3,TCEB1,TCF7L2,KDM5A,MAPT,PIK3R1,KCNMA1,CBX1,GNA12,ISL1,ADAMTS5,PTCH1,MEF2C,SIRT1
GO: Molecular Function	GO:0000983	transcription factor activity, RNA polymerase II core promoter sequence-specific	1.148E-3	2	13	HEY1,MEF2C
GO: Molecular Function	GO:0019900	kinase binding	1.271E-3	9	677	FOXO3,TCF7L2,TRIB2,MAPT,PIK3R1,SPRED1,MAPK1,SLC2A1,SIRT1
GO: Molecular Function	GO:0003713	transcription coactivator activity	1.371E-3	6	311	SOX11,KDM5A,MTF1,PPRC1,ISL1,TAF4
GO: Molecular Function	GO:0016706	oxidoreductase activity, acting on paired donors, with incorporation or reduction of molecular oxygen, 2-oxoglutarate as one donor, and incorporation of one atom each of oxygen into both donors	1.681E-3	3	60	KDM5B,KDM5A,KDM7A
GO: Molecular Function	GO:0033613	activating transcription factor binding	1.681E-3	3	60	ZNF516,ISL1,MEF2C
GO: Molecular Function	GO:0003714	transcription corepressor activity	2.082E-3	5	230	KDM5B,AEBP2,SIRT1,MEIS2,HIRA
GO: Molecular Function	GO:0019901	protein kinase binding	2.551E-3	8	608	FOXO3,TCF7L2,TRIB2,MAPT,PIK3R1,SPRED1,MAPK1,SIRT1
GO: Molecular Function	GO:0005545	1-phosphatidylinositol binding	3.322E-3	2	22	ZFYVE1,EPB41
GO: Molecular Function	GO:0035257	nuclear hormone receptor binding	3.516E-3	4	159	TCF7L2,PIK3R1,ISL1,SIRT1
GO: Molecular Function	GO:0030332	cyclin binding	3.630E-3	2	23	CDKN1A,PTCH1
GO: Molecular Function	GO:0034647	histone demethylase activity (H3-trimethyl-K4 specific)	3.932E-3	1	1	KDM5B
GO: Molecular Function	GO:0051213	dioxygenase activity	5.144E-3	3	89	KDM5B,KDM5A,KDM7A
GO: Molecular Function	GO:0004860	protein kinase inhibitor activity	5.144E-3	3	89	CDKN1A,TRIB2,SPRED1
GO: Molecular Function	GO:0001221	transcription cofactor binding	5.355E-3	2	28	FOXO3,NFYA
GO: Molecular Function	GO:0019210	kinase inhibitor activity	5.473E-3	3	91	CDKN1A,TRIB2,SPRED1
GO: Molecular Function	GO:0051427	hormone receptor binding	5.562E-3	4	181	TCF7L2,PIK3R1,ISL1,SIRT1
GO: Molecular Function	GO:0030291	protein serine/threonine kinase inhibitor activity	5.737E-3	2	29	CDKN1A,SPRED1
GO: Molecular Function	GO:0019902	phosphatase binding	6.004E-3	4	185	MAPT,PIK3R1,SPRED1,MAPK1
GO: Molecular Function	GO:0043425	bHLH transcription factor binding	6.131E-3	2	30	ISL1,SIRT1
GO: Molecular Function	GO:0001104	RNA polymerase II transcription cofactor activity	6.166E-3	3	95	SOX11,PPRC1,ISL1
GO: Biological Process	GO:1903508	positive regulation of nucleic acid-templated transcription	1.728E-9	24	1505	FOXO3,SOX11,TCEB1,BMPR1A,TCF7L2,HEY1,KDM5A,MTF1,PPRC1,PIK3R1,ESRRG,ZNF516,NFYA,POU4F1,KDM7A,ISL1,MAPK1,TAF4,PTCH1,ZEB2,MEF2C,SIRT1,MEIS2,SMAD5
GO: Biological Process	GO:0045893	positive regulation of transcription, DNA-templated	1.728E-9	24	1505	FOXO3,SOX11,TCEB1,BMPR1A,TCF7L2,HEY1,KDM5A,MTF1,PPRC1,PIK3R1,ESRRG,ZNF516,NFYA,POU4F1,KDM7A,ISL1,MAPK1,TAF4,PTCH1,ZEB2,MEF2C,SIRT1,MEIS2,SMAD5
GO: Biological Process	GO:1902680	positive regulation of RNA biosynthetic process	2.464E-9	24	1532	FOXO3,SOX11,TCEB1,BMPR1A,TCF7L2,HEY1,KDM5A,MTF1,PPRC1,PIK3R1,ESRRG,ZNF516,NFYA,POU4F1,KDM7A,ISL1,MAPK1,TAF4,PTCH1,ZEB2,MEF2C,SIRT1,MEIS2,SMAD5
GO: Biological Process	GO:0051254	positive regulation of RNA metabolic process	4.110E-9	24	1572	FOXO3,SOX11,TCEB1,BMPR1A,TCF7L2,HEY1,KDM5A,MTF1,PPRC1,PIK3R1,ESRRG,ZNF516,NFYA,POU4F1,KDM7A,ISL1,MAPK1,TAF4,PTCH1,ZEB2,MEF2C,SIRT1,MEIS2,SMAD5
GO: Biological Process	GO:0010628	positive regulation of gene expression	1.337E-8	25	1810	FOXO3,SOX11,TCEB1,KDM5B,BMPR1A,TCF7L2,HEY1,KDM5A,MTF1,PPRC1,PIK3R1,ESRRG,ZNF516,NFYA,POU4F1,KDM7A,ISL1,MAPK1,TAF4,PTCH1,ZEB2,MEF2C,SIRT1,MEIS2,SMAD5
GO: Biological Process	GO:0010557	positive regulation of macromolecule biosynthetic process	2.827E-8	24	1735	FOXO3,SOX11,TCEB1,BMPR1A,TCF7L2,HEY1,KDM5A,MTF1,PPRC1,PIK3R1,ESRRG,ZNF516,NFYA,POU4F1,KDM7A,ISL1,MAPK1,TAF4,PTCH1,ZEB2,MEF2C,SIRT1,MEIS2,SMAD5
GO: Biological Process	GO:0051253	negative regulation of RNA metabolic process	3.873E-8	21	1353	FOXO3,SOX11,KDM5B,SRSF10,TCF7L2,TRIB2,HEY1,KDM5A,EIF4A2,NAB1,AEBP2,CBX1,POU4F1,ISL1,FOXN3,PTCH1,ZEB2,MEF2C,SIRT1,MEIS2,HIRA
GO: Biological Process	GO:0045935	positive regulation of nucleobase-containing compound metabolic process	5.038E-8	24	1788	FOXO3,SOX11,TCEB1,BMPR1A,TCF7L2,HEY1,KDM5A,MTF1,PPRC1,PIK3R1,ESRRG,ZNF516,NFYA,POU4F1,KDM7A,ISL1,MAPK1,TAF4,PTCH1,ZEB2,MEF2C,SIRT1,MEIS2,SMAD5
GO: Biological Process	GO:0045595	regulation of cell differentiation	5.652E-8	23	1656	FOXO3,SOX11,BMPR1A,TCF7L2,BNIP2,TRIB2,HEY1,MAPT,PIK3R1,NFYA,POU4F1,SRGAP2,ISL1,MAPK1,PTCH1,CAPRIN1,ZEB2,MEF2C,NDRG4,TMEM64,SIRT1,MEIS2,SMAD5
GO: Biological Process	GO:0010558	negative regulation of macromolecule biosynthetic process	5.828E-8	22	1520	FOXO3,SOX11,KDM5B,TCF7L2,TRIB2,HEY1,KDM5A,HBEGF,EIF4A2,NAB1,AEBP2,CBX1,POU4F1,ISL1,FOXN3,PTCH1,CAPRIN1,ZEB2,MEF2C,SIRT1,MEIS2,HIRA
GO: Biological Process	GO:1903507	negative regulation of nucleic acid-templated transcription	9.316E-8	20	1292	FOXO3,SOX11,KDM5B,TCF7L2,TRIB2,HEY1,KDM5A,EIF4A2,NAB1,AEBP2,CBX1,POU4F1,ISL1,FOXN3,PTCH1,ZEB2,MEF2C,SIRT1,MEIS2,HIRA
GO: Biological Process	GO:0022008	neurogenesis	9.439E-8	24	1848	FOXO3,SOX11,BMPR1A,TCF7L2,BNIP2,CELSR3,HEY1,MAPT,CALU,HBEGF,NAB1,KCNMA1,SPRED1,POU4F1,SRGAP2,ISL1,MAPK1,PTCH1,CAPRIN1,ZEB2,MEF2C,NDRG4,SIRT1,ADGRL3
GO: Biological Process	GO:1902679	negative regulation of RNA biosynthetic process	1.125E-7	20	1307	FOXO3,SOX11,KDM5B,TCF7L2,TRIB2,HEY1,KDM5A,EIF4A2,NAB1,AEBP2,CBX1,POU4F1,ISL1,FOXN3,PTCH1,ZEB2,MEF2C,SIRT1,MEIS2,HIRA
GO: Biological Process	GO:0031327	negative regulation of cellular biosynthetic process	1.169E-7	22	1581	FOXO3,SOX11,KDM5B,TCF7L2,TRIB2,HEY1,KDM5A,HBEGF,EIF4A2,NAB1,AEBP2,CBX1,POU4F1,ISL1,FOXN3,PTCH1,CAPRIN1,ZEB2,MEF2C,SIRT1,MEIS2,HIRA
GO: Biological Process	GO:0031328	positive regulation of cellular biosynthetic process	1.204E-7	24	1872	FOXO3,SOX11,TCEB1,BMPR1A,TCF7L2,HEY1,KDM5A,MTF1,PPRC1,PIK3R1,ESRRG,ZNF516,NFYA,POU4F1,KDM7A,ISL1,MAPK1,TAF4,PTCH1,ZEB2,MEF2C,SIRT1,MEIS2,SMAD5
GO: Biological Process	GO:0006357	regulation of transcription from RNA polymerase II promoter	1.386E-7	24	1886	FOXO3,SOX11,TCEB1,BMPR1A,TCF7L2,HEY1,KDM5A,MTF1,PPRC1,PIK3R1,ESRRG,NFYA,AEBP2,POU4F1,ISL1,TAF4,PTCH1,ZEB2,MEF2C,SIRT1,MEIS2,HIRA,SMAD5,BRWD1
GO: Biological Process	GO:0051173	positive regulation of nitrogen compound metabolic process	1.400E-7	24	1887	FOXO3,SOX11,TCEB1,BMPR1A,TCF7L2,HEY1,KDM5A,MTF1,PPRC1,PIK3R1,ESRRG,ZNF516,NFYA,POU4F1,KDM7A,ISL1,MAPK1,TAF4,PTCH1,ZEB2,MEF2C,SIRT1,MEIS2,SMAD5
GO: Biological Process	GO:0045944	positive regulation of transcription from RNA polymerase II promoter	1.600E-7	18	1080	FOXO3,SOX11,TCEB1,BMPR1A,TCF7L2,HEY1,MTF1,PPRC1,PIK3R1,ESRRG,NFYA,POU4F1,ISL1,ZEB2,MEF2C,SIRT1,MEIS2,SMAD5
GO: Biological Process	GO:0009890	negative regulation of biosynthetic process	1.608E-7	22	1610	FOXO3,SOX11,KDM5B,TCF7L2,TRIB2,HEY1,KDM5A,HBEGF,EIF4A2,NAB1,AEBP2,CBX1,POU4F1,ISL1,FOXN3,PTCH1,CAPRIN1,ZEB2,MEF2C,SIRT1,MEIS2,HIRA
GO: Biological Process	GO:0051172	negative regulation of nitrogen compound metabolic process	1.661E-7	22	1613	FOXO3,SOX11,KDM5B,SRSF10,TCF7L2,TRIB2,HEY1,KDM5A,EIF4A2,NAB1,AEBP2,CBX1,POU4F1,ISL1,FOXN3,PTCH1,CAPRIN1,ZEB2,MEF2C,SIRT1,MEIS2,HIRA
GO: Biological Process	GO:0048699	generation of neurons	1.703E-7	23	1759	FOXO3,SOX11,BMPR1A,TCF7L2,BNIP2,CELSR3,HEY1,MAPT,CALU,HBEGF,KCNMA1,SPRED1,POU4F1,SRGAP2,ISL1,MAPK1,PTCH1,CAPRIN1,ZEB2,MEF2C,NDRG4,SIRT1,ADGRL3
GO: Biological Process	GO:0009891	positive regulation of biosynthetic process	1.757E-7	24	1910	FOXO3,SOX11,TCEB1,BMPR1A,TCF7L2,HEY1,KDM5A,MTF1,PPRC1,PIK3R1,ESRRG,ZNF516,NFYA,POU4F1,KDM7A,ISL1,MAPK1,TAF4,PTCH1,ZEB2,MEF2C,SIRT1,MEIS2,SMAD5
GO: Biological Process	GO:0051960	regulation of nervous system development	1.898E-7	16	855	FOXO3,SOX11,BMPR1A,TCF7L2,BNIP2,HEY1,MAPT,POU4F1,SRGAP2,ISL1,CAPRIN1,ZEB2,MEF2C,NDRG4,SIRT1,ADGRL3
GO: Biological Process	GO:0045934	negative regulation of nucleobase-containing compound metabolic process	1.955E-7	21	1488	FOXO3,SOX11,KDM5B,SRSF10,TCF7L2,TRIB2,HEY1,KDM5A,EIF4A2,NAB1,AEBP2,CBX1,POU4F1,ISL1,FOXN3,PTCH1,ZEB2,MEF2C,SIRT1,MEIS2,HIRA
GO: Biological Process	GO:0050767	regulation of neurogenesis	2.437E-7	15	759	FOXO3,SOX11,BMPR1A,TCF7L2,BNIP2,HEY1,MAPT,POU4F1,SRGAP2,ISL1,CAPRIN1,ZEB2,MEF2C,NDRG4,SIRT1
GO: Biological Process	GO:0051094	positive regulation of developmental process	3.901E-7	19	1277	FOXO3,SOX11,KDM5B,BMPR1A,TCF7L2,BNIP2,HEY1,MAPT,PIK3R1,ISL1,PTCH1,CAPRIN1,ZEB2,MEF2C,NDRG4,TMEM64,SIRT1,ADGRL3,SMAD5
GO: Biological Process	GO:2000113	negative regulation of cellular macromolecule biosynthetic process	4.900E-7	20	1432	FOXO3,SOX11,KDM5B,TCF7L2,TRIB2,HEY1,KDM5A,HBEGF,NAB1,AEBP2,CBX1,POU4F1,ISL1,FOXN3,PTCH1,CAPRIN1,ZEB2,MEF2C,SIRT1,MEIS2
GO: Biological Process	GO:0051240	positive regulation of multicellular organismal process	5.147E-7	21	1577	FOXO3,SOX11,KDM5B,BMPR1A,TCF7L2,BNIP2,HEY1,MAPT,HBEGF,PIK3R1,KCNMA1,ISL1,PTCH1,CAPRIN1,ZEB2,MEF2C,NDRG4,TMEM64,SIRT1,ADGRL3,SMAD5
GO: Biological Process	GO:0045597	positive regulation of cell differentiation	6.857E-7	16	941	FOXO3,SOX11,BMPR1A,TCF7L2,BNIP2,HEY1,MAPT,PIK3R1,PTCH1,CAPRIN1,ZEB2,MEF2C,NDRG4,TMEM64,SIRT1,SMAD5
GO: Biological Process	GO:0060284	regulation of cell development	8.222E-7	16	954	FOXO3,SOX11,BMPR1A,TCF7L2,BNIP2,HEY1,MAPT,NFYA,POU4F1,SRGAP2,ISL1,CAPRIN1,ZEB2,MEF2C,NDRG4,SIRT1
GO: Biological Process	GO:2000026	regulation of multicellular organismal development	8.810E-7	22	1777	FOXO3,SOX11,KDM5B,BMPR1A,TCF7L2,BNIP2,HEY1,MAPT,PIK3R1,NAB1,POU4F1,SRGAP2,ISL1,PTCH1,CAPRIN1,ZEB2,MEF2C,NDRG4,TMEM64,SIRT1,ADGRL3,MEIS2
GO: Biological Process	GO:0010629	negative regulation of gene expression	8.861E-7	21	1630	CDKN1A,FOXO3,SOX11,KDM5B,SRSF10,TCF7L2,TRIB2,HEY1,KDM5A,NAB1,AEBP2,CBX1,POU4F1,ISL1,FOXN3,PTCH1,CAPRIN1,ZEB2,MEF2C,SIRT1,MEIS2
GO: Biological Process	GO:0014033	neural crest cell differentiation	9.554E-7	6	83	SOX11,BMPR1A,ISL1,MAPK1,ZEB2,MEF2C
GO: Biological Process	GO:0048762	mesenchymal cell differentiation	1.138E-6	8	197	SOX11,BMPR1A,TCF7L2,HEY1,ISL1,MAPK1,ZEB2,MEF2C
GO: Biological Process	GO:0045892	negative regulation of transcription, DNA-templated	1.361E-6	18	1250	FOXO3,SOX11,KDM5B,TCF7L2,TRIB2,HEY1,KDM5A,NAB1,AEBP2,CBX1,POU4F1,ISL1,FOXN3,PTCH1,ZEB2,MEF2C,SIRT1,MEIS2
GO: Biological Process	GO:0045596	negative regulation of cell differentiation	2.296E-6	13	675	FOXO3,SOX11,BMPR1A,TCF7L2,TRIB2,HEY1,PIK3R1,SRGAP2,ISL1,MAPK1,PTCH1,SIRT1,MEIS2
GO: Biological Process	GO:0048863	stem cell differentiation	2.661E-6	9	297	SOX11,BMPR1A,TCF7L2,HEY1,NFYA,ISL1,MAPK1,ZEB2,MEF2C
GO: Biological Process	GO:0043009	chordate embryonic development	2.970E-6	13	691	SOX11,BMPR1A,TCF7L2,BNIP2,SRSF1,HEY1,HBEGF,GNA12,ISL1,MAPK1,PTCH1,ZEB2,MEF2C
GO: Biological Process	GO:0009792	embryo development ending in birth or egg hatching	3.422E-6	13	700	SOX11,BMPR1A,TCF7L2,BNIP2,SRSF1,HEY1,HBEGF,GNA12,ISL1,MAPK1,PTCH1,ZEB2,MEF2C
GO: Biological Process	GO:0030182	neuron differentiation	3.832E-6	20	1634	FOXO3,SOX11,BNIP2,CELSR3,HEY1,MAPT,CALU,HBEGF,KCNMA1,SPRED1,POU4F1,SRGAP2,ISL1,MAPK1,PTCH1,CAPRIN1,ZEB2,MEF2C,NDRG4,SIRT1
GO: Biological Process	GO:0045669	positive regulation of osteoblast differentiation	6.428E-6	5	66	SOX11,BMPR1A,HEY1,MEF2C,SMAD5
GO: Biological Process	GO:0003007	heart morphogenesis	6.482E-6	8	249	SOX11,BMPR1A,HEY1,POU4F1,ISL1,PTCH1,MEF2C,NDRG4
GO: Biological Process	GO:0009790	embryo development	7.025E-6	16	1126	SOX11,BMPR1A,TCF7L2,BNIP2,SRSF1,HEY1,HBEGF,GNA12,ISL1,MAPK1,PTCH1,ZEB2,MEF2C,NDRG4,HIRA,SMAD5
GO: Biological Process	GO:0014070	response to organic cyclic compound	8.076E-6	14	879	SLC6A1,CDKN1A,HEY1,CALU,PIK3R1,ESRRG,KCNMA1,ISL1,MAPK1,PTCH1,MEF2C,SIRT1,ADGRL3,SMAD5
GO: Biological Process	GO:0014031	mesenchymal cell development	8.210E-6	7	183	SOX11,BMPR1A,TCF7L2,HEY1,ISL1,MAPK1,ZEB2
GO: Biological Process	GO:0045667	regulation of osteoblast differentiation	8.234E-6	6	120	SOX11,BMPR1A,HEY1,PTCH1,MEF2C,SMAD5
GO: Biological Process	GO:0048545	response to steroid hormone	8.497E-6	10	436	SLC6A1,CDKN1A,HEY1,PIK3R1,ESRRG,KCNMA1,ISL1,MAPK1,PTCH1,SIRT1
GO: Biological Process	GO:0060485	mesenchyme development	8.644E-6	8	259	SOX11,BMPR1A,TCF7L2,HEY1,ISL1,MAPK1,ZEB2,MEF2C
GO: Biological Process	GO:0003208	cardiac ventricle morphogenesis	9.221E-6	5	71	SOX11,HEY1,POU4F1,ISL1,MEF2C
GO: Biological Process	GO:0048864	stem cell development	1.013E-5	7	189	SOX11,BMPR1A,TCF7L2,HEY1,ISL1,MAPK1,ZEB2
GO: Biological Process	GO:0009719	response to endogenous stimulus	1.023E-5	20	1744	SLC6A1,CDKN1A,FOXO3,SOX11,BMPR1A,TCF7L2,HEY1,HBEGF,PIK3R1,ESRRG,KCNMA1,SPRED1,ISL1,MAPK1,PTCH1,MEF2C,SIRT1,ADGRL3,ATP6V0A2,SMAD5
GO: Biological Process	GO:0014032	neural crest cell development	1.207E-5	5	75	SOX11,BMPR1A,ISL1,MAPK1,ZEB2
GO: Biological Process	GO:0000122	negative regulation of transcription from RNA polymerase II promoter	1.278E-5	13	791	FOXO3,SOX11,TCF7L2,HEY1,KDM5A,AEBP2,POU4F1,ISL1,PTCH1,ZEB2,MEF2C,SIRT1,MEIS2
GO: Biological Process	GO:0021536	diencephalon development	1.656E-5	5	80	BMPR1A,TCF7L2,POU4F1,ISL1,ZEB2
GO: Biological Process	GO:0050673	epithelial cell proliferation	1.932E-5	9	380	SOX11,KDM5B,BMPR1A,TCF7L2,HEY1,MAPK1,PTCH1,MEF2C,SIRT1
GO: Biological Process	GO:0001649	osteoblast differentiation	2.404E-5	7	216	SOX11,BMPR1A,HEY1,PTCH1,MEF2C,HIRA,SMAD5
GO: Biological Process	GO:0001503	ossification	2.418E-5	9	391	SOX11,BMPR1A,TCF7L2,HEY1,NAB1,PTCH1,MEF2C,HIRA,SMAD5
GO: Biological Process	GO:0060563	neuroepithelial cell differentiation	3.096E-5	5	91	SOX11,HEY1,KCNMA1,ZEB2,MEF2C
GO: Biological Process	GO:0002065	columnar/cuboidal epithelial cell differentiation	3.161E-5	6	152	CDKN1A,SOX11,HEY1,KCNMA1,ZEB2,MEF2C
GO: Biological Process	GO:0050678	regulation of epithelial cell proliferation	3.456E-5	8	314	SOX11,KDM5B,BMPR1A,TCF7L2,HEY1,PTCH1,MEF2C,SIRT1
GO: Biological Process	GO:0045778	positive regulation of ossification	3.810E-5	5	95	SOX11,BMPR1A,HEY1,MEF2C,SMAD5
GO: Biological Process	GO:0045664	regulation of neuron differentiation	3.817E-5	11	632	FOXO3,SOX11,BNIP2,HEY1,MAPT,ISL1,CAPRIN1,ZEB2,MEF2C,NDRG4,SIRT1
GO: Biological Process	GO:0051093	negative regulation of developmental process	4.389E-5	13	890	FOXO3,SOX11,BMPR1A,TCF7L2,TRIB2,HEY1,PIK3R1,SRGAP2,ISL1,MAPK1,PTCH1,SIRT1,MEIS2
GO: Biological Process	GO:0071495	cellular response to endogenous stimulus	4.393E-5	16	1306	CDKN1A,FOXO3,SOX11,BMPR1A,TCF7L2,HEY1,HBEGF,PIK3R1,ESRRG,SPRED1,ISL1,MAPK1,MEF2C,SIRT1,ATP6V0A2,SMAD5
GO: Biological Process	GO:0061061	muscle structure development	4.527E-5	11	644	SOX11,TCF7L2,BNIP2,HEY1,HBEGF,PIK3R1,POU4F1,ISL1,MEF2C,SIRT1,HIRA
GO: Biological Process	GO:2000111	positive regulation of macrophage apoptotic process	4.672E-5	2	3	MEF2C,SIRT1
GO: Biological Process	GO:0042127	regulation of cell proliferation	5.972E-5	18	1646	CDKN1A,FOXO3,SOX11,FRK,KDM5B,BMPR1A,TCF7L2,HEY1,HBEGF,PIK3R1,NFYA,ISL1,MAPK1,PTCH1,MEF2C,NDRG4,SIRT1,SGK3
GO: Biological Process	GO:0035295	tube development	6.911E-5	11	675	CDKN1A,SOX11,KDM5B,BMPR1A,TCF7L2,MAPK1,PTCH1,ZEB2,MEF2C,NDRG4,SMAD5
GO: Biological Process	GO:0060429	epithelium development	7.160E-5	15	1213	CDKN1A,SOX11,KDM5B,BMPR1A,TCF7L2,HEY1,HBEGF,NAB1,KCNMA1,MAPK1,PTCH1,ZEB2,MEF2C,NDRG4,SMAD5
GO: Biological Process	GO:0006913	nucleocytoplasmic transport	7.294E-5	9	450	CDKN1A,BMPR1A,SRSF10,TCF7L2,SRSF1,PIK3R1,ANP32A,MAPK1,SIRT1
GO: Biological Process	GO:0009725	response to hormone	7.407E-5	14	1074	SLC6A1,CDKN1A,HEY1,HBEGF,PIK3R1,ESRRG,KCNMA1,SPRED1,ISL1,MAPK1,PTCH1,MEF2C,SIRT1,ATP6V0A2
GO: Biological Process	GO:0048729	tissue morphogenesis	7.480E-5	11	681	SOX11,KDM5B,BMPR1A,HEY1,HBEGF,POU4F1,ISL1,PTCH1,ZEB2,MEF2C,NDRG4
GO: Biological Process	GO:0003203	endocardial cushion morphogenesis	7.635E-5	3	21	BMPR1A,HEY1,ISL1
GO: Biological Process	GO:0051169	nuclear transport	7.828E-5	9	455	CDKN1A,BMPR1A,SRSF10,TCF7L2,SRSF1,PIK3R1,ANP32A,MAPK1,SIRT1
GO: Biological Process	GO:0045666	positive regulation of neuron differentiation	8.349E-5	8	356	SOX11,BNIP2,MAPT,CAPRIN1,ZEB2,MEF2C,NDRG4,SIRT1
GO: Biological Process	GO:0033002	muscle cell proliferation	9.158E-5	6	184	TCF7L2,HBEGF,PIK3R1,MEF2C,NDRG4,SIRT1
GO: Biological Process	GO:0048880	sensory system development	9.320E-5	2	4	POU4F1,ISL1
GO: Biological Process	GO:0045165	cell fate commitment	1.033E-4	7	272	BMPR1A,TCF7L2,POU4F1,ISL1,PTCH1,MEF2C,SMAD5
GO: Biological Process	GO:0051049	regulation of transport	1.067E-4	19	1885	SLC6A1,CDKN1A,SOX11,KDM5B,BMPR1A,GAPVD1,TCF7L2,MAPT,PIK3R1,KCNMA1,RAB15,ISL1,MAPK1,PTCH1,MEF2C,NDRG4,SLC2A1,SIRT1,SGK3
GO: Biological Process	GO:0051241	negative regulation of multicellular organismal process	1.073E-4	14	1112	FOXO3,SOX11,BMPR1A,TCF7L2,TRIB2,HEY1,PIK3R1,KCNMA1,SRGAP2,ISL1,PTCH1,MEF2C,SIRT1,MEIS2
GO: Biological Process	GO:0003206	cardiac chamber morphogenesis	1.160E-4	5	120	SOX11,HEY1,POU4F1,ISL1,MEF2C
GO: Biological Process	GO:0030278	regulation of ossification	1.369E-4	6	198	SOX11,BMPR1A,HEY1,PTCH1,MEF2C,SMAD5
GO: Biological Process	GO:0007167	enzyme linked receptor protein signaling pathway	1.377E-4	15	1286	CDKN1A,FOXO3,SOX11,FRK,BMPR1A,TCF7L2,HBEGF,PIK3R1,SPRED1,MAPK1,MEF2C,NDRG4,SIRT1,ATP6V0A2,SMAD5
GO: Biological Process	GO:0003231	cardiac ventricle development	1.406E-4	5	125	SOX11,HEY1,POU4F1,ISL1,MEF2C
GO: Biological Process	GO:0060914	heart formation	1.471E-4	3	26	BMPR1A,ISL1,MEF2C
GO: Biological Process	GO:0001880	Mullerian duct regression	1.549E-4	2	5	BMPR1A,SMAD5
GO: Biological Process	GO:0003151	outflow tract morphogenesis	1.550E-4	4	68	SOX11,HEY1,ISL1,MEF2C
GO: Biological Process	GO:0048645	organ formation	1.641E-4	4	69	BMPR1A,ISL1,MAPK1,MEF2C
GO: Biological Process	GO:0007517	muscle organ development	1.709E-4	8	395	SOX11,TCF7L2,HEY1,HBEGF,POU4F1,ISL1,MEF2C,SIRT1
GO: Biological Process	GO:0051962	positive regulation of nervous system development	1.826E-4	9	509	SOX11,BNIP2,MAPT,CAPRIN1,ZEB2,MEF2C,NDRG4,SIRT1,ADGRL3
GO: Biological Process	GO:0060322	head development	1.899E-4	11	757	BMPR1A,TCF7L2,MAPT,POU4F1,KDM7A,SRGAP2,ISL1,MAPK1,PTCH1,ZEB2,NDRG4
GO: Biological Process	GO:0072358	cardiovascular system development	1.967E-4	13	1033	SOX11,BMPR1A,TCF7L2,HEY1,HBEGF,POU4F1,ISL1,MAPK1,PTCH1,MEF2C,NDRG4,SIRT1,SMAD5
GO: Biological Process	GO:0072359	circulatory system development	1.967E-4	13	1033	SOX11,BMPR1A,TCF7L2,HEY1,HBEGF,POU4F1,ISL1,MAPK1,PTCH1,MEF2C,NDRG4,SIRT1,SMAD5
GO: Biological Process	GO:0051098	regulation of binding	2.134E-4	7	306	SOX11,TCF7L2,HEY1,ISL1,MEF2C,EPB41,SIRT1
GO: Biological Process	GO:0048598	embryonic morphogenesis	2.334E-4	10	647	SOX11,BMPR1A,TCF7L2,GNA12,MAPK1,PTCH1,ZEB2,MEF2C,NDRG4,HIRA
GO: Biological Process	GO:0001701	in utero embryonic development	2.590E-4	8	420	BMPR1A,BNIP2,SRSF1,HEY1,HBEGF,GNA12,MAPK1,PTCH1
GO: Biological Process	GO:0008543	fibroblast growth factor receptor signaling pathway	2.596E-4	7	316	CDKN1A,FOXO3,TCF7L2,HBEGF,PIK3R1,SPRED1,MAPK1
GO: Biological Process	GO:0048666	neuron development	2.603E-4	15	1363	CELSR3,HEY1,MAPT,CALU,HBEGF,SPRED1,POU4F1,SRGAP2,ISL1,MAPK1,CAPRIN1,ZEB2,MEF2C,NDRG4,SIRT1
GO: Biological Process	GO:0031175	neuron projection development	2.614E-4	14	1211	CELSR3,MAPT,CALU,HBEGF,SPRED1,POU4F1,SRGAP2,ISL1,MAPK1,CAPRIN1,ZEB2,MEF2C,NDRG4,SIRT1
GO: Biological Process	GO:0071363	cellular response to growth factor stimulus	2.626E-4	12	922	CDKN1A,FOXO3,SOX11,BMPR1A,TCF7L2,HBEGF,PIK3R1,SPRED1,MAPK1,MEF2C,SIRT1,SMAD5
GO: Biological Process	GO:0048589	developmental growth	2.872E-4	10	664	FOXO3,KDM5B,BMPR1A,TCF7L2,MAPT,HBEGF,PTCH1,ZEB2,MEF2C,NDRG4
GO: Biological Process	GO:0040007	growth	2.928E-4	13	1076	CDKN1A,FOXO3,KDM5B,BMPR1A,TCF7L2,MAPT,HBEGF,PTCH1,ZEB2,MEF2C,NDRG4,SIRT1,SGK3
GO: Biological Process	GO:0010720	positive regulation of cell development	3.109E-4	9	547	SOX11,TCF7L2,BNIP2,MAPT,CAPRIN1,ZEB2,MEF2C,NDRG4,SIRT1
GO: Biological Process	GO:0043523	regulation of neuron apoptotic process	3.149E-4	6	231	FOXO3,KCNMA1,POU4F1,ISL1,MEF2C,SIRT1
GO: Biological Process	GO:0033034	positive regulation of myeloid cell apoptotic process	3.237E-4	2	7	MEF2C,SIRT1
GO: Biological Process	GO:0034720	histone H3-K4 demethylation	3.237E-4	2	7	KDM5B,KDM5A
GO: Biological Process	GO:0070848	response to growth factor	3.257E-4	12	944	CDKN1A,FOXO3,SOX11,BMPR1A,TCF7L2,HBEGF,PIK3R1,SPRED1,MAPK1,MEF2C,SIRT1,SMAD5
GO: Biological Process	GO:0003197	endocardial cushion development	3.310E-4	3	34	BMPR1A,HEY1,ISL1
GO: Biological Process	GO:0007417	central nervous system development	3.652E-4	12	956	SOX11,BMPR1A,TCF7L2,MAPT,POU4F1,KDM7A,SRGAP2,ISL1,MAPK1,PTCH1,ZEB2,NDRG4
GO: Biological Process	GO:0050769	positive regulation of neurogenesis	3.702E-4	8	443	SOX11,BNIP2,MAPT,CAPRIN1,ZEB2,MEF2C,NDRG4,SIRT1
GO: Biological Process	GO:0007169	transmembrane receptor protein tyrosine kinase signaling pathway	3.757E-4	12	959	CDKN1A,FOXO3,FRK,TCF7L2,HBEGF,PIK3R1,SPRED1,MAPK1,MEF2C,NDRG4,SIRT1,ATP6V0A2
GO: Biological Process	GO:0044344	cellular response to fibroblast growth factor stimulus	3.760E-4	7	336	CDKN1A,FOXO3,TCF7L2,HBEGF,PIK3R1,SPRED1,MAPK1
GO: Biological Process	GO:0045445	myoblast differentiation	3.830E-4	4	86	TCF7L2,PIK3R1,ISL1,MEF2C
GO: Biological Process	GO:0000902	cell morphogenesis	3.849E-4	16	1574	TCF7L2,CELSR3,HEY1,MAPT,HBEGF,SPRED1,GNA12,POU4F1,SRGAP2,ISL1,MAPK1,CAPRIN1,ZEB2,MEF2C,EPB41,BRWD1
GO: Biological Process	GO:0006468	protein phosphorylation	4.036E-4	18	1915	CDKN1A,FRK,BMPR1A,BNIP2,TRIB2,CELSR3,HBEGF,PIK3R1,SPRED1,ISL1,MAPK1,CTDSPL,ZEB2,MEF2C,NDRG4,SIRT1,SMAD5,SGK3
GO: Biological Process	GO:0071774	response to fibroblast growth factor	4.109E-4	7	341	CDKN1A,FOXO3,TCF7L2,HBEGF,PIK3R1,SPRED1,MAPK1
GO: Biological Process	GO:0001764	neuron migration	4.170E-4	5	158	CELSR3,MAPT,SRGAP2,MEF2C,ADGRL3
GO: Biological Process	GO:0001708	cell fate specification	4.180E-4	4	88	BMPR1A,POU4F1,ISL1,PTCH1
GO: Biological Process	GO:0003139	secondary heart field specification	4.305E-4	2	8	ISL1,MEF2C
GO: Biological Process	GO:0090400	stress-induced premature senescence	4.305E-4	2	8	CDKN1A,SIRT1
GO: Biological Process	GO:0016477	cell migration	4.484E-4	14	1277	CELSR3,MAPT,HBEGF,PIK3R1,GNA12,POU4F1,SRGAP2,ISL1,MAPK1,ZEB2,MEF2C,NDRG4,ADGRL3,SGK3
GO: Biological Process	GO:0009888	tissue development	4.522E-4	18	1933	CDKN1A,SOX11,KDM5B,BMPR1A,TCF7L2,HEY1,HBEGF,NAB1,ZNF516,KCNMA1,POU4F1,ISL1,MAPK1,PTCH1,ZEB2,MEF2C,NDRG4,SMAD5
GO: Biological Process	GO:0090311	regulation of protein deacetylation	4.613E-4	3	38	KDM5A,SPRED1,SIRT1
GO: Biological Process	GO:0070076	histone lysine demethylation	4.613E-4	3	38	KDM5B,KDM5A,KDM7A
GO: Biological Process	GO:0007507	heart development	4.645E-4	9	578	SOX11,BMPR1A,HEY1,POU4F1,ISL1,MAPK1,PTCH1,MEF2C,NDRG4
GO: Biological Process	GO:0007420	brain development	4.818E-4	10	709	BMPR1A,TCF7L2,MAPT,POU4F1,KDM7A,SRGAP2,ISL1,PTCH1,ZEB2,NDRG4
GO: Biological Process	GO:0016577	histone demethylation	4.984E-4	3	39	KDM5B,KDM5A,KDM7A
GO: Biological Process	GO:0061053	somite development	5.156E-4	4	93	SOX11,BMPR1A,PTCH1,ZEB2
GO: Biological Process	GO:0051402	neuron apoptotic process	5.211E-4	6	254	FOXO3,KCNMA1,POU4F1,ISL1,MEF2C,SIRT1
GO: Biological Process	GO:0031960	response to corticosteroid	5.369E-4	5	167	CDKN1A,HEY1,PIK3R1,KCNMA1,ISL1
GO: Biological Process	GO:0021559	trigeminal nerve development	5.520E-4	2	9	POU4F1,ISL1
GO: Biological Process	GO:0048568	embryonic organ development	5.632E-4	8	472	SOX11,BMPR1A,TCF7L2,HEY1,MAPK1,PTCH1,MEF2C,NDRG4
GO: Biological Process	GO:0050679	positive regulation of epithelial cell proliferation	5.667E-4	5	169	SOX11,KDM5B,BMPR1A,TCF7L2,SIRT1
GO: Biological Process	GO:0003205	cardiac chamber development	5.667E-4	5	169	SOX11,HEY1,POU4F1,ISL1,MEF2C
GO: Biological Process	GO:0044092	negative regulation of molecular function	5.761E-4	13	1155	CDKN1A,SOX11,TCF7L2,TRIB2,HEY1,KDM5A,CALU,EIF4A2,SPRED1,ISL1,PTCH1,ZFYVE1,SIRT1
GO: Biological Process	GO:0048511	rhythmic process	5.770E-4	7	361	FOXO3,KDM5B,KDM5A,NFYA,KCNMA1,TAF4,SIRT1
GO: Biological Process	GO:0008214	protein dealkylation	5.780E-4	3	41	KDM5B,KDM5A,KDM7A
GO: Biological Process	GO:0006482	protein demethylation	5.780E-4	3	41	KDM5B,KDM5A,KDM7A
GO: Biological Process	GO:0033993	response to lipid	6.192E-4	11	870	SLC6A1,CDKN1A,HEY1,PIK3R1,ESRRG,KCNMA1,ISL1,MAPK1,PTCH1,MEF2C,SIRT1
GO: Biological Process	GO:0021983	pituitary gland development	6.207E-4	3	42	BMPR1A,TCF7L2,ISL1
GO: Biological Process	GO:0032870	cellular response to hormone stimulus	6.307E-4	10	734	HEY1,HBEGF,PIK3R1,ESRRG,SPRED1,ISL1,MAPK1,MEF2C,SIRT1,ATP6V0A2
GO: Biological Process	GO:0072132	mesenchyme morphogenesis	6.653E-4	3	43	BMPR1A,HEY1,ISL1
GO: Biological Process	GO:0060896	neural plate pattern specification	6.883E-4	2	10	BMPR1A,PTCH1
GO: Biological Process	GO:0003211	cardiac ventricle formation	6.883E-4	2	10	SOX11,MEF2C
GO: Biological Process	GO:2000109	regulation of macrophage apoptotic process	6.883E-4	2	10	MEF2C,SIRT1
GO: Biological Process	GO:0032989	cellular component morphogenesis	6.920E-4	16	1660	TCF7L2,CELSR3,HEY1,MAPT,HBEGF,SPRED1,GNA12,POU4F1,SRGAP2,ISL1,MAPK1,CAPRIN1,ZEB2,MEF2C,EPB41,BRWD1
GO: Biological Process	GO:0060341	regulation of cellular localization	7.295E-4	14	1341	CDKN1A,SOX11,KDM5B,BMPR1A,TCF7L2,MAPT,PIK3R1,RAB15,ISL1,MAPK1,MEF2C,NDRG4,SLC2A1,SIRT1
GO: Biological Process	GO:0009887	organ morphogenesis	7.352E-4	12	1034	SOX11,KDM5B,BMPR1A,TCF7L2,HEY1,NAB1,POU4F1,ISL1,MAPK1,PTCH1,MEF2C,NDRG4
GO: Biological Process	GO:0007389	pattern specification process	7.386E-4	8	492	BMPR1A,HEY1,ISL1,PTCH1,ZEB2,MEF2C,NDRG4,SMAD5
GO: Biological Process	GO:0051101	regulation of DNA binding	7.578E-4	4	103	SOX11,HEY1,ISL1,SIRT1
GO: Biological Process	GO:0055010	ventricular cardiac muscle tissue morphogenesis	7.607E-4	3	45	HEY1,POU4F1,ISL1
GO: Biological Process	GO:0050768	negative regulation of neurogenesis	7.896E-4	6	275	FOXO3,SOX11,BMPR1A,HEY1,SRGAP2,ISL1
GO: Biological Process	GO:0023061	signal release	8.201E-4	8	500	SLC6A1,SOX11,KDM5B,TCF7L2,ISL1,MEF2C,SLC2A1,SIRT1
GO: Biological Process	GO:0014706	striated muscle tissue development	8.299E-4	7	384	SOX11,TCF7L2,HEY1,POU4F1,ISL1,MEF2C,NDRG4
GO: Biological Process	GO:0051090	regulation of sequence-specific DNA binding transcription factor activity	8.299E-4	7	384	TCF7L2,TRIB2,PPRC1,MAPK1,PTCH1,SIRT1,SGK3
GO: Biological Process	GO:0045599	negative regulation of fat cell differentiation	8.643E-4	3	47	TCF7L2,TRIB2,SIRT1
GO: Biological Process	GO:0008284	positive regulation of cell proliferation	9.049E-4	11	911	CDKN1A,SOX11,KDM5B,BMPR1A,TCF7L2,HBEGF,NFYA,ISL1,MAPK1,MEF2C,SIRT1
GO: Biological Process	GO:0031399	regulation of protein modification process	9.756E-4	17	1886	CDKN1A,BMPR1A,BNIP2,TRIB2,CELSR3,KDM5A,HBEGF,PIK3R1,SPRED1,ISL1,MAPK1,ZFYVE1,CTDSPL,ZEB2,MEF2C,NDRG4,SIRT1
GO: Biological Process	GO:0003207	cardiac chamber formation	1.004E-3	2	12	SOX11,MEF2C
GO: Biological Process	GO:0003128	heart field specification	1.004E-3	2	12	ISL1,MEF2C
GO: Biological Process	GO:0071888	macrophage apoptotic process	1.004E-3	2	12	MEF2C,SIRT1
GO: Biological Process	GO:0044744	protein targeting to nucleus	1.021E-3	6	289	CDKN1A,BMPR1A,TCF7L2,PIK3R1,MAPK1,SIRT1
GO: Biological Process	GO:0006606	protein import into nucleus	1.021E-3	6	289	CDKN1A,BMPR1A,TCF7L2,PIK3R1,MAPK1,SIRT1
GO: Biological Process	GO:1902593	single-organism nuclear import	1.021E-3	6	289	CDKN1A,BMPR1A,TCF7L2,PIK3R1,MAPK1,SIRT1
GO: Biological Process	GO:0043392	negative regulation of DNA binding	1.036E-3	3	50	SOX11,HEY1,SIRT1
GO: Biological Process	GO:0051674	localization of cell	1.043E-3	14	1391	CELSR3,MAPT,HBEGF,PIK3R1,GNA12,POU4F1,SRGAP2,ISL1,MAPK1,ZEB2,MEF2C,NDRG4,ADGRL3,SGK3
GO: Biological Process	GO:0048870	cell motility	1.043E-3	14	1391	CELSR3,MAPT,HBEGF,PIK3R1,GNA12,POU4F1,SRGAP2,ISL1,MAPK1,ZEB2,MEF2C,NDRG4,ADGRL3,SGK3
GO: Biological Process	GO:0097285	cell-type specific apoptotic process	1.056E-3	8	520	FOXO3,TCF7L2,PIK3R1,KCNMA1,POU4F1,ISL1,MEF2C,SIRT1
GO: Biological Process	GO:0060537	muscle tissue development	1.083E-3	7	402	SOX11,TCF7L2,HEY1,POU4F1,ISL1,MEF2C,NDRG4
GO: Biological Process	GO:0051170	nuclear import	1.096E-3	6	293	CDKN1A,BMPR1A,TCF7L2,PIK3R1,MAPK1,SIRT1
GO: Biological Process	GO:0001709	cell fate determination	1.097E-3	3	51	ISL1,PTCH1,MEF2C
GO: Biological Process	GO:0030330	DNA damage response, signal transduction by p53 class mediator	1.107E-3	4	114	CDKN1A,FOXO3,SPRED1,SIRT1
GO: Biological Process	GO:0048011	neurotrophin TRK receptor signaling pathway	1.115E-3	7	404	CDKN1A,FOXO3,HBEGF,PIK3R1,SPRED1,MAPK1,MEF2C
GO: Biological Process	GO:0042692	muscle cell differentiation	1.131E-3	7	405	TCF7L2,BNIP2,HEY1,PIK3R1,ISL1,MEF2C,HIRA
GO: Biological Process	GO:0090398	cellular senescence	1.161E-3	3	52	CDKN1A,BMPR1A,SIRT1
GO: Biological Process	GO:0003229	ventricular cardiac muscle tissue development	1.161E-3	3	52	HEY1,POU4F1,ISL1
GO: Biological Process	GO:0038179	neurotrophin signaling pathway	1.164E-3	7	407	CDKN1A,FOXO3,HBEGF,PIK3R1,SPRED1,MAPK1,MEF2C
GO: Biological Process	GO:0051961	negative regulation of nervous system development	1.175E-3	6	297	FOXO3,SOX11,BMPR1A,HEY1,SRGAP2,ISL1
GO: Biological Process	GO:0001542	ovulation from ovarian follicle	1.184E-3	2	13	FOXO3,SIRT1
GO: Biological Process	GO:0048935	peripheral nervous system neuron development	1.184E-3	2	13	POU4F1,ISL1
GO: Biological Process	GO:0048934	peripheral nervous system neuron differentiation	1.184E-3	2	13	POU4F1,ISL1
GO: Biological Process	GO:0007548	sex differentiation	1.195E-3	6	298	FOXO3,BMPR1A,TCF7L2,TAF4,SIRT1,SMAD5
GO: Biological Process	GO:1901214	regulation of neuron death	1.195E-3	6	298	FOXO3,KCNMA1,POU4F1,ISL1,MEF2C,SIRT1
GO: Biological Process	GO:0045637	regulation of myeloid cell differentiation	1.234E-3	5	201	FOXO3,PIK3R1,MEF2C,TMEM64,MEIS2
GO: Biological Process	GO:0035239	tube morphogenesis	1.248E-3	7	412	SOX11,KDM5B,TCF7L2,PTCH1,ZEB2,MEF2C,NDRG4
GO: Biological Process	GO:0071407	cellular response to organic cyclic compound	1.248E-3	7	412	HEY1,ESRRG,ISL1,PTCH1,MEF2C,SIRT1,SMAD5
GO: Biological Process	GO:0010875	positive regulation of cholesterol efflux	1.378E-3	2	14	PTCH1,SIRT1
GO: Biological Process	GO:0060033	anatomical structure regression	1.378E-3	2	14	BMPR1A,SMAD5
GO: Biological Process	GO:0003272	endocardial cushion formation	1.378E-3	2	14	BMPR1A,HEY1
GO: Biological Process	GO:0048738	cardiac muscle tissue development	1.406E-3	5	207	HEY1,POU4F1,ISL1,MEF2C,NDRG4
GO: Biological Process	GO:0009267	cellular response to starvation	1.422E-3	4	122	CDKN1A,TCF7L2,SLC2A1,SIRT1
GO: Biological Process	GO:0031401	positive regulation of protein modification process	1.424E-3	13	1274	CDKN1A,BMPR1A,BNIP2,TRIB2,HBEGF,PIK3R1,SPRED1,ISL1,MAPK1,ZEB2,MEF2C,NDRG4,SIRT1
GO: Biological Process	GO:0071396	cellular response to lipid	1.433E-3	7	422	HEY1,ESRRG,ISL1,MAPK1,PTCH1,MEF2C,SIRT1
GO: Biological Process	GO:0060411	cardiac septum morphogenesis	1.440E-3	3	56	SOX11,HEY1,ISL1
GO: Biological Process	GO:0000904	cell morphogenesis involved in differentiation	1.479E-3	12	1121	TCF7L2,HEY1,MAPT,HBEGF,SPRED1,POU4F1,SRGAP2,ISL1,MAPK1,CAPRIN1,ZEB2,MEF2C
GO: Biological Process	GO:0046883	regulation of hormone secretion	1.486E-3	6	311	SOX11,KDM5B,TCF7L2,ISL1,SLC2A1,SIRT1
GO: Biological Process	GO:0061564	axon development	1.559E-3	10	827	CELSR3,MAPT,CALU,HBEGF,SPRED1,POU4F1,SRGAP2,ISL1,MAPK1,ZEB2
GO: Biological Process	GO:0038095	Fc-epsilon receptor signaling pathway	1.586E-3	6	315	CDKN1A,FOXO3,HBEGF,PIK3R1,SPRED1,MAPK1
GO: Biological Process	GO:0048660	regulation of smooth muscle cell proliferation	1.602E-3	4	126	TCF7L2,HBEGF,PIK3R1,NDRG4
GO: Biological Process	GO:0060047	heart contraction	1.661E-3	5	215	SRSF1,HBEGF,PIK3R1,SIRT1,SMAD5
GO: Biological Process	GO:0033554	cellular response to stress	1.696E-3	17	1983	SLC6A1,CDKN1A,FOXO3,TCEB1,BMPR1A,TCF7L2,MAPT,CALU,PIK3R1,SPRED1,ISL1,FOXN3,MAPK1,ZEB2,MEF2C,SLC2A1,SIRT1
GO: Biological Process	GO:0003015	heart process	1.730E-3	5	217	SRSF1,HBEGF,PIK3R1,SIRT1,SMAD5
GO: Biological Process	GO:0042770	signal transduction in response to DNA damage	1.746E-3	4	129	CDKN1A,FOXO3,SPRED1,SIRT1
GO: Biological Process	GO:0014855	striated muscle cell proliferation	1.758E-3	3	60	MEF2C,NDRG4,SIRT1
GO: Biological Process	GO:0030030	cell projection organization	1.795E-3	15	1642	CELSR3,HEY1,MAPT,CALU,HBEGF,SPRED1,POU4F1,SRGAP2,ISL1,MAPK1,CAPRIN1,ZEB2,MEF2C,NDRG4,SIRT1
GO: Biological Process	GO:0048659	smooth muscle cell proliferation	1.847E-3	4	131	TCF7L2,HBEGF,PIK3R1,NDRG4
GO: Biological Process	GO:0055008	cardiac muscle tissue morphogenesis	1.932E-3	3	62	HEY1,POU4F1,ISL1
GO: Biological Process	GO:0003006	developmental process involved in reproduction	1.932E-3	9	708	FOXO3,KDM5B,BMPR1A,TCF7L2,HEY1,MAPK1,TAF4,SIRT1,SMAD5
GO: Biological Process	GO:0022603	regulation of anatomical structure morphogenesis	1.943E-3	11	1002	BMPR1A,TCF7L2,HEY1,MAPT,GNA12,ISL1,CAPRIN1,ZEB2,MEF2C,EPB41,BRWD1
GO: Biological Process	GO:0001932	regulation of protein phosphorylation	1.969E-3	14	1487	CDKN1A,BMPR1A,BNIP2,TRIB2,CELSR3,HBEGF,PIK3R1,SPRED1,ISL1,MAPK1,CTDSPL,ZEB2,NDRG4,SIRT1
GO: Biological Process	GO:0019220	regulation of phosphate metabolic process	1.990E-3	16	1834	CDKN1A,BMPR1A,BNIP2,TRIB2,CELSR3,HBEGF,PIK3R1,SPRED1,ISL1,MAPK1,ZFYVE1,CTDSPL,ZEB2,MEF2C,NDRG4,SIRT1
GO: Biological Process	GO:0045722	positive regulation of gluconeogenesis	2.043E-3	2	17	TCF7L2,SIRT1
GO: Biological Process	GO:0051174	regulation of phosphorus metabolic process	2.140E-3	16	1847	CDKN1A,BMPR1A,BNIP2,TRIB2,CELSR3,HBEGF,PIK3R1,SPRED1,ISL1,MAPK1,ZFYVE1,CTDSPL,ZEB2,MEF2C,NDRG4,SIRT1
GO: Biological Process	GO:0046822	regulation of nucleocytoplasmic transport	2.147E-3	5	228	CDKN1A,BMPR1A,TCF7L2,PIK3R1,SIRT1
GO: Biological Process	GO:0070997	neuron death	2.162E-3	6	335	FOXO3,KCNMA1,POU4F1,ISL1,MEF2C,SIRT1
GO: Biological Process	GO:0051100	negative regulation of binding	2.175E-3	4	137	SOX11,HEY1,ISL1,SIRT1
GO: Biological Process	GO:0017038	protein import	2.260E-3	6	338	CDKN1A,BMPR1A,TCF7L2,PIK3R1,MAPK1,SIRT1
GO: Biological Process	GO:0060548	negative regulation of cell death	2.286E-3	11	1023	CDKN1A,SOX11,TCF7L2,BNIP2,PIK3R1,POU4F1,SRGAP2,ISL1,MEF2C,SIRT1,SGK3
GO: Biological Process	GO:0044093	positive regulation of molecular function	2.287E-3	16	1859	CDKN1A,GAPVD1,TCF7L2,BNIP2,TRIB2,PPRC1,HBEGF,SPRED1,SRGAP2,ISL1,MAPK1,ZEB2,MEF2C,EPB41,SIRT1,SGK3
GO: Biological Process	GO:0032373	positive regulation of sterol transport	2.292E-3	2	18	PTCH1,SIRT1
GO: Biological Process	GO:0032376	positive regulation of cholesterol transport	2.292E-3	2	18	PTCH1,SIRT1
GO: Biological Process	GO:2000678	negative regulation of transcription regulatory region DNA binding	2.292E-3	2	18	SOX11,HEY1
GO: Biological Process	GO:0010721	negative regulation of cell development	2.328E-3	6	340	FOXO3,SOX11,BMPR1A,HEY1,SRGAP2,ISL1
GO: Biological Process	GO:0030856	regulation of epithelial cell differentiation	2.353E-3	4	140	BMPR1A,HEY1,PTCH1,ZEB2
GO: Biological Process	GO:0045682	regulation of epidermis development	2.412E-3	3	67	HEY1,NAB1,PTCH1
GO: Biological Process	GO:0051099	positive regulation of binding	2.477E-3	4	142	TCF7L2,ISL1,MEF2C,EPB41
GO: Biological Process	GO:0008286	insulin receptor signaling pathway	2.503E-3	6	345	HBEGF,PIK3R1,SPRED1,MAPK1,SIRT1,ATP6V0A2
GO: Biological Process	GO:0034644	cellular response to UV	2.516E-3	3	68	CDKN1A,PIK3R1,SIRT1
GO: Biological Process	GO:0060037	pharyngeal system development	2.555E-3	2	19	ISL1,PTCH1
GO: Biological Process	GO:0048557	embryonic digestive tract morphogenesis	2.555E-3	2	19	SOX11,TCF7L2
GO: Biological Process	GO:0070988	demethylation	2.623E-3	3	69	KDM5B,KDM5A,KDM7A
GO: Biological Process	GO:0048608	reproductive structure development	2.632E-3	7	470	FOXO3,KDM5B,TCF7L2,HEY1,MAPK1,TAF4,SIRT1
GO: Biological Process	GO:0043433	negative regulation of sequence-specific DNA binding transcription factor activity	2.671E-3	4	145	TCF7L2,TRIB2,PTCH1,SIRT1
GO: Biological Process	GO:0046887	positive regulation of hormone secretion	2.738E-3	4	146	SOX11,TCF7L2,ISL1,SIRT1
GO: Biological Process	GO:0048667	cell morphogenesis involved in neuron differentiation	2.757E-3	10	894	MAPT,HBEGF,SPRED1,POU4F1,SRGAP2,ISL1,MAPK1,CAPRIN1,ZEB2,MEF2C
GO: Biological Process	GO:0061458	reproductive system development	2.760E-3	7	474	FOXO3,KDM5B,TCF7L2,HEY1,MAPK1,TAF4,SIRT1
GO: Biological Process	GO:0036473	cell death in response to oxidative stress	2.845E-3	3	71	FOXO3,MAPT,SIRT1
GO: Biological Process	GO:0060415	muscle tissue morphogenesis	2.961E-3	3	72	HEY1,POU4F1,ISL1
GO: Biological Process	GO:0032386	regulation of intracellular transport	2.989E-3	8	614	CDKN1A,BMPR1A,TCF7L2,MAPT,PIK3R1,MAPK1,NDRG4,SIRT1
GO: Biological Process	GO:0034504	protein localization to nucleus	3.007E-3	6	358	CDKN1A,BMPR1A,TCF7L2,PIK3R1,MAPK1,SIRT1
GO: Biological Process	GO:0001934	positive regulation of protein phosphorylation	3.031E-3	11	1061	CDKN1A,BMPR1A,BNIP2,HBEGF,PIK3R1,SPRED1,ISL1,MAPK1,ZEB2,NDRG4,SIRT1
GO: Biological Process	GO:0045937	positive regulation of phosphate metabolic process	3.077E-3	12	1224	CDKN1A,BMPR1A,BNIP2,HBEGF,PIK3R1,SPRED1,ISL1,MAPK1,ZEB2,MEF2C,NDRG4,SIRT1
GO: Biological Process	GO:0010562	positive regulation of phosphorus metabolic process	3.077E-3	12	1224	CDKN1A,BMPR1A,BNIP2,HBEGF,PIK3R1,SPRED1,ISL1,MAPK1,ZEB2,MEF2C,NDRG4,SIRT1
GO: Biological Process	GO:0030509	BMP signaling pathway	3.091E-3	4	151	SOX11,BMPR1A,TCF7L2,SMAD5
GO: Biological Process	GO:1900078	positive regulation of cellular response to insulin stimulus	3.122E-3	2	21	PIK3R1,SIRT1
GO: Biological Process	GO:0010874	regulation of cholesterol efflux	3.122E-3	2	21	PTCH1,SIRT1
GO: Biological Process	GO:0042593	glucose homeostasis	3.139E-3	5	249	FOXO3,TCF7L2,PIK3R1,PTCH1,SIRT1
GO: Biological Process	GO:0033500	carbohydrate homeostasis	3.139E-3	5	249	FOXO3,TCF7L2,PIK3R1,PTCH1,SIRT1
GO: Biological Process	GO:0035270	endocrine system development	3.165E-3	4	152	BMPR1A,TCF7L2,ISL1,MAPK1
GO: Biological Process	GO:0071560	cellular response to transforming growth factor beta stimulus	3.304E-3	5	252	SOX11,BMPR1A,MEF2C,SIRT1,SMAD5
GO: Biological Process	GO:0033158	regulation of protein import into nucleus, translocation	3.426E-3	2	22	CDKN1A,SIRT1
GO: Biological Process	GO:0007173	epidermal growth factor receptor signaling pathway	3.442E-3	6	368	CDKN1A,FOXO3,HBEGF,PIK3R1,SPRED1,MAPK1
GO: Biological Process	GO:2000648	positive regulation of stem cell proliferation	3.451E-3	3	76	SOX11,BMPR1A,NFYA
GO: Biological Process	GO:0060996	dendritic spine development	3.451E-3	3	76	SRGAP2,CAPRIN1,MEF2C
GO: Biological Process	GO:0043524	negative regulation of neuron apoptotic process	3.474E-3	4	156	POU4F1,ISL1,MEF2C,SIRT1
GO: Biological Process	GO:0071559	response to transforming growth factor beta	3.475E-3	5	255	SOX11,BMPR1A,MEF2C,SIRT1,SMAD5
GO: Biological Process	GO:0051046	regulation of secretion	3.510E-3	9	774	SLC6A1,SOX11,KDM5B,TCF7L2,RAB15,ISL1,MEF2C,SLC2A1,SIRT1
GO: Biological Process	GO:0046879	hormone secretion	3.535E-3	6	370	SOX11,KDM5B,TCF7L2,ISL1,SLC2A1,SIRT1
GO: Biological Process	GO:0051384	response to glucocorticoid	3.554E-3	4	157	CDKN1A,HEY1,PIK3R1,ISL1
GO: Biological Process	GO:0003002	regionalization	3.582E-3	6	371	BMPR1A,HEY1,ISL1,PTCH1,ZEB2,MEF2C
GO: Biological Process	GO:0060562	epithelial tube morphogenesis	3.582E-3	6	371	SOX11,KDM5B,PTCH1,ZEB2,MEF2C,NDRG4
GO: Biological Process	GO:0042325	regulation of phosphorylation	3.623E-3	14	1589	CDKN1A,BMPR1A,BNIP2,TRIB2,CELSR3,HBEGF,PIK3R1,SPRED1,ISL1,MAPK1,CTDSPL,ZEB2,NDRG4,SIRT1
GO: Biological Process	GO:0008015	blood circulation	3.705E-3	7	500	SRSF1,HBEGF,PIK3R1,KCNMA1,EPB41,SIRT1,SMAD5
GO: Biological Process	GO:0071383	cellular response to steroid hormone stimulus	3.718E-3	4	159	HEY1,ESRRG,ISL1,SIRT1
GO: Biological Process	GO:0043496	regulation of protein homodimerization activity	3.742E-3	2	23	ISL1,MEF2C
GO: Biological Process	GO:0060716	labyrinthine layer blood vessel development	3.742E-3	2	23	HEY1,MAPK1
GO: Biological Process	GO:0045663	positive regulation of myoblast differentiation	3.742E-3	2	23	PIK3R1,MEF2C
GO: Biological Process	GO:0006925	inflammatory cell apoptotic process	3.742E-3	2	23	MEF2C,SIRT1
GO: Biological Process	GO:0038127	ERBB signaling pathway	3.774E-3	6	375	CDKN1A,FOXO3,HBEGF,PIK3R1,SPRED1,MAPK1
GO: Biological Process	GO:0003013	circulatory system process	3.786E-3	7	502	SRSF1,HBEGF,PIK3R1,KCNMA1,EPB41,SIRT1,SMAD5
GO: Biological Process	GO:0010035	response to inorganic substance	3.786E-3	7	502	SLC6A1,FOXO3,MTF1,KCNMA1,PTCH1,MEF2C,SIRT1
GO: Biological Process	GO:0001568	blood vessel development	3.844E-3	8	640	TCF7L2,HEY1,HBEGF,ISL1,MAPK1,MEF2C,SIRT1,SMAD5
GO: Biological Process	GO:0032024	positive regulation of insulin secretion	3.849E-3	3	79	TCF7L2,ISL1,SIRT1
GO: Biological Process	GO:0038093	Fc receptor signaling pathway	3.873E-3	6	377	CDKN1A,FOXO3,HBEGF,PIK3R1,SPRED1,MAPK1
GO: Biological Process	GO:0042594	response to starvation	3.888E-3	4	161	CDKN1A,TCF7L2,SLC2A1,SIRT1
GO: Biological Process	GO:0071773	cellular response to BMP stimulus	3.974E-3	4	162	SOX11,BMPR1A,TCF7L2,SMAD5
GO: Biological Process	GO:0071772	response to BMP	3.974E-3	4	162	SOX11,BMPR1A,TCF7L2,SMAD5
GO: Biological Process	GO:0007422	peripheral nervous system development	3.987E-3	3	80	NAB1,POU4F1,ISL1
GO: Biological Process	GO:0048644	muscle organ morphogenesis	3.987E-3	3	80	HEY1,POU4F1,ISL1
GO: Biological Process	GO:0061180	mammary gland epithelium development	3.987E-3	3	80	KDM5B,MAPK1,PTCH1
GO: Biological Process	GO:0051545	negative regulation of elastin biosynthetic process	3.994E-3	1	1	HBEGF
GO: Biological Process	GO:0035332	positive regulation of hippo signaling	3.994E-3	1	1	SOX11
GO: Biological Process	GO:0003185	sinoatrial valve morphogenesis	3.994E-3	1	1	MEF2C
GO: Biological Process	GO:0051355	proprioception involved in equilibrioception	3.994E-3	1	1	POU4F1
GO: Biological Process	GO:0060025	regulation of synaptic activity	3.994E-3	1	1	MEF2C
GO: Biological Process	GO:0034721	histone H3-K4 demethylation, trimethyl-H3-K4-specific	3.994E-3	1	1	KDM5B
GO: Biological Process	GO:1900260	negative regulation of RNA-directed RNA polymerase activity	3.994E-3	1	1	EIF4A2
GO: Biological Process	GO:0003172	sinoatrial valve development	3.994E-3	1	1	MEF2C
GO: Biological Process	GO:1900259	regulation of RNA-directed RNA polymerase activity	3.994E-3	1	1	EIF4A2
GO: Biological Process	GO:2000861	regulation of estrogen secretion	3.994E-3	1	1	KDM5B
GO: Biological Process	GO:0060082	eye blink reflex	3.994E-3	1	1	KCNMA1
GO: Biological Process	GO:2000864	regulation of estradiol secretion	3.994E-3	1	1	KDM5B
GO: Biological Process	GO:0048338	mesoderm structural organization	3.994E-3	1	1	BMPR1A
GO: Biological Process	GO:2001207	regulation of transcription elongation from RNA polymerase I promoter	3.994E-3	1	1	POU4F1
GO: Biological Process	GO:0045081	negative regulation of interleukin-10 biosynthetic process	3.994E-3	1	1	TRIB2
GO: Biological Process	GO:1901726	negative regulation of histone deacetylase activity	3.994E-3	1	1	KDM5A
GO: Biological Process	GO:2000654	regulation of cellular response to testosterone stimulus	3.994E-3	1	1	SIRT1
GO: Biological Process	GO:0051543	regulation of elastin biosynthetic process	3.994E-3	1	1	HBEGF
GO: Biological Process	GO:2001208	negative regulation of transcription elongation from RNA polymerase I promoter	3.994E-3	1	1	POU4F1
GO: Biological Process	GO:0006343	establishment of chromatin silencing	3.994E-3	1	1	SIRT1
GO: Biological Process	GO:2000614	positive regulation of thyroid-stimulating hormone secretion	3.994E-3	1	1	SIRT1
GO: Biological Process	GO:0035937	estrogen secretion	3.994E-3	1	1	KDM5B
GO: Biological Process	GO:1902748	positive regulation of lens fiber cell differentiation	3.994E-3	1	1	ZEB2
GO: Biological Process	GO:0003363	lamellipodium assembly involved in ameboidal cell migration	3.994E-3	1	1	SRGAP2
GO: Biological Process	GO:0001544	initiation of primordial ovarian follicle growth	3.994E-3	1	1	FOXO3
GO: Biological Process	GO:0048352	paraxial mesoderm structural organization	3.994E-3	1	1	BMPR1A
GO: Biological Process	GO:0003138	primary heart field specification	3.994E-3	1	1	MEF2C
GO: Biological Process	GO:0071441	negative regulation of histone H3-K14 acetylation	3.994E-3	1	1	SIRT1
GO: Biological Process	GO:2000655	negative regulation of cellular response to testosterone stimulus	3.994E-3	1	1	SIRT1
GO: Biological Process	GO:0051939	gamma-aminobutyric acid import	3.994E-3	1	1	SLC6A1
GO: Biological Process	GO:0021998	neural plate mediolateral regionalization	3.994E-3	1	1	BMPR1A
GO: Biological Process	GO:0035938	estradiol secretion	3.994E-3	1	1	KDM5B
GO: Biological Process	GO:0048646	anatomical structure formation involved in morphogenesis	4.018E-3	12	1265	SOX11,BMPR1A,CELSR3,HEY1,HBEGF,ISL1,MAPK1,PTCH1,ZEB2,MEF2C,SIRT1,SMAD5
GO: Biological Process	GO:0030099	myeloid cell differentiation	4.026E-3	6	380	FOXO3,PIK3R1,MEF2C,TMEM64,MEIS2,SMAD5
GO: Biological Process	GO:0042327	positive regulation of phosphorylation	4.046E-3	11	1102	CDKN1A,BMPR1A,BNIP2,HBEGF,PIK3R1,SPRED1,ISL1,MAPK1,ZEB2,NDRG4,SIRT1
GO: Biological Process	GO:0060384	innervation	4.072E-3	2	24	POU4F1,ISL1
GO: Biological Process	GO:0032092	positive regulation of protein binding	4.129E-3	3	81	TCF7L2,MEF2C,EPB41
GO: Biological Process	GO:0009914	hormone transport	4.130E-3	6	382	SOX11,KDM5B,TCF7L2,ISL1,SLC2A1,SIRT1
GO: Biological Process	GO:0010976	positive regulation of neuron projection development	4.157E-3	5	266	MAPT,CAPRIN1,ZEB2,NDRG4,SIRT1
GO: Biological Process	GO:0006476	protein deacetylation	4.274E-3	3	82	KDM5A,SPRED1,SIRT1
GO: Biological Process	GO:0031669	cellular response to nutrient levels	4.334E-3	4	166	CDKN1A,TCF7L2,SLC2A1,SIRT1
GO: Biological Process	GO:0030855	epithelial cell differentiation	4.377E-3	8	654	CDKN1A,SOX11,BMPR1A,HEY1,KCNMA1,PTCH1,ZEB2,MEF2C
GO: Biological Process	GO:0030004	cellular monovalent inorganic cation homeostasis	4.421E-3	3	83	KCNMA1,ATP6V0A2,SGK3
GO: Biological Process	GO:0035107	appendage morphogenesis	4.427E-3	4	167	SOX11,BMPR1A,GNA12,PTCH1
GO: Biological Process	GO:0035108	limb morphogenesis	4.427E-3	4	167	SOX11,BMPR1A,GNA12,PTCH1
GO: Biological Process	GO:0042063	gliogenesis	4.428E-3	5	270	SOX11,TCF7L2,NAB1,SRGAP2,MAPK1
GO: Biological Process	GO:0030900	forebrain development	4.566E-3	6	390	BMPR1A,TCF7L2,POU4F1,SRGAP2,ISL1,ZEB2
GO: Biological Process	GO:2000772	regulation of cellular senescence	4.770E-3	2	26	BMPR1A,SIRT1
GO: Biological Process	GO:0001944	vasculature development	4.834E-3	8	665	TCF7L2,HEY1,HBEGF,ISL1,MAPK1,MEF2C,SIRT1,SMAD5
GO: Biological Process	GO:0051247	positive regulation of protein metabolic process	4.937E-3	14	1645	CDKN1A,BMPR1A,TCF7L2,BNIP2,TRIB2,HBEGF,PIK3R1,SPRED1,ISL1,MAPK1,ZEB2,MEF2C,NDRG4,SIRT1
GO: Biological Process	GO:0002009	morphogenesis of an epithelium	5.031E-3	7	529	SOX11,KDM5B,HBEGF,PTCH1,ZEB2,MEF2C,NDRG4
GO: Biological Process	GO:0035601	protein deacylation	5.043E-3	3	87	KDM5A,SPRED1,SIRT1
GO: Biological Process	GO:0030728	ovulation	5.139E-3	2	27	FOXO3,SIRT1
GO: Biological Process	GO:0098732	macromolecule deacylation	5.206E-3	3	88	KDM5A,SPRED1,SIRT1
GO: Biological Process	GO:0019827	stem cell population maintenance	5.221E-3	4	175	FOXO3,BMPR1A,TCF7L2,NFYA
GO: Biological Process	GO:0002768	immune response-regulating cell surface receptor signaling pathway	5.292E-3	7	534	CDKN1A,FOXO3,HBEGF,PIK3R1,SPRED1,MAPK1,MEF2C
GO: Biological Process	GO:0010770	positive regulation of cell morphogenesis involved in differentiation	5.327E-3	4	176	TCF7L2,MAPT,CAPRIN1,ZEB2
GO: Biological Process	GO:0098727	maintenance of cell number	5.434E-3	4	177	FOXO3,BMPR1A,TCF7L2,NFYA
GO: Biological Process	GO:0030318	melanocyte differentiation	5.520E-3	2	28	ZEB2,MEF2C
GO: Biological Process	GO:0060021	palate development	5.542E-3	3	90	SOX11,BMPR1A,MEF2C
GO: Biological Process	GO:0010243	response to organonitrogen compound	5.585E-3	10	988	SLC6A1,CDKN1A,HBEGF,PIK3R1,SPRED1,MAPK1,MEF2C,SIRT1,ADGRL3,ATP6V0A2
GO: Biological Process	GO:0072331	signal transduction by p53 class mediator	5.652E-3	4	179	CDKN1A,FOXO3,SPRED1,SIRT1
GO: Biological Process	GO:0032869	cellular response to insulin stimulus	5.871E-3	6	411	HBEGF,PIK3R1,SPRED1,MAPK1,SIRT1,ATP6V0A2
GO: Biological Process	GO:0043393	regulation of protein binding	5.876E-3	4	181	TCF7L2,ISL1,MEF2C,EPB41
GO: Biological Process	GO:0007569	cell aging	5.891E-3	3	92	CDKN1A,BMPR1A,SIRT1
GO: Biological Process	GO:0043516	regulation of DNA damage response, signal transduction by p53 class mediator	5.913E-3	2	29	SPRED1,SIRT1
GO: Biological Process	GO:0006376	mRNA splice site selection	5.913E-3	2	29	SRSF10,SRSF1
GO: Biological Process	GO:1901700	response to oxygen-containing compound	6.063E-3	14	1684	SLC6A1,CDKN1A,FOXO3,TCF7L2,HBEGF,PIK3R1,KCNMA1,SPRED1,MAPK1,PTCH1,MEF2C,SIRT1,ADGRL3,ATP6V0A2
GO: Biological Process	GO:0021915	neural tube development	6.106E-3	4	183	SOX11,TCF7L2,PTCH1,ZEB2
GO: Biological Process	GO:0016049	cell growth	6.135E-3	7	549	CDKN1A,MAPT,HBEGF,ZEB2,NDRG4,SIRT1,SGK3
GO: Biological Process	GO:0033032	regulation of myeloid cell apoptotic process	6.320E-3	2	30	MEF2C,SIRT1
GO: Biological Process	GO:0036075	replacement ossification	6.320E-3	2	30	NAB1,MEF2C
GO: Biological Process	GO:0001958	endochondral ossification	6.320E-3	2	30	NAB1,MEF2C
GO: Biological Process	GO:0042306	regulation of protein import into nucleus	6.463E-3	4	186	CDKN1A,BMPR1A,PIK3R1,SIRT1
Mouse Phenotype	MP:0004251	failure of heart looping	6.353E-6	5	42	SOX11,ISL1,MEF2C,HIRA,SMAD5
Mouse Phenotype	MP:0003935	abnormal craniofacial development	7.689E-6	11	340	SOX11,BMPR1A,TCF7L2,MAPT,ISL1,MAPK1,PTCH1,ZEB2,MEF2C,HIRA,SMAD5
Mouse Phenotype	MP:0002884	abnormal pharyngeal arch morphology	3.615E-5	7	146	SOX11,ISL1,MAPK1,ZEB2,MEF2C,HIRA,SMAD5
Pathway	83109	Endometrial cancer	7.631E-5	4	52	FOXO3,TCF7L2,PIK3R1,MAPK1
Pathway	695200	HIF-1 signaling pathway	9.649E-5	5	106	CDKN1A,TCEB1,PIK3R1,MAPK1,SLC2A1
Pathway	83107	Renal cell carcinoma	1.942E-4	4	66	TCEB1,PIK3R1,MAPK1,SLC2A1
Pathway	83105	Pathways in cancer	5.195E-4	7	327	CDKN1A,TCEB1,TCF7L2,PIK3R1,MAPK1,PTCH1,SLC2A1
Pathway	83049	ErbB signaling pathway	5.854E-4	4	88	CDKN1A,HBEGF,PIK3R1,MAPK1
Pathway	83111	Prostate cancer	6.110E-4	4	89	CDKN1A,TCF7L2,PIK3R1,MAPK1
Pathway	83119	Non-small cell lung cancer	1.878E-3	3	56	FOXO3,PIK3R1,MAPK1
Pathway	83117	Acute myeloid leukemia	1.977E-3	3	57	TCF7L2,PIK3R1,MAPK1

Pathway	83106	Colorectal cancer	2.516E-3	3	62	TCF7L2,PIK3R1,MAPK1
Pathway	83110	Glioma	2.879E-3	3	65	CDKN1A,PIK3R1,MAPK1
Pathway	782000	Proteoglycans in cancer	2.962E-3	5	225	CDKN1A,HBEGF,PIK3R1,MAPK1,PTCH1
Pathway	83114	Melanoma	3.699E-3	3	71	CDKN1A,PIK3R1,MAPK1
Pathway	814182	Prolactin signaling pathway	3.848E-3	3	72	FOXO3,PIK3R1,MAPK1
Pathway	83116	Chronic myeloid leukemia	4.000E-3	3	73	CDKN1A,PIK3R1,MAPK1
Pathway	83064	TGF-beta signaling pathway	5.172E-3	3	80	BMPR1A,MAPK1,SMAD5
Pathway	137928	LPA receptor mediated events	1.830E-4	4	65	MAPT,HBEGF,PIK3R1,GNA12
Pathway	137991	ErbB4 signaling events	3.619E-4	3	32	HBEGF,PIK3R1,MAPK1
Pathway	137956	HIF-2-alpha transcription factor network	4.339E-4	3	34	TCEB1,SLC2A1,SIRT1
Pathway	137948	BMP receptor signaling	5.146E-4	3	36	MAPK1,CTDSPL,SMAD5
Pathway	137972	Signaling events mediated by HDAC Class III	6.525E-4	3	39	CDKN1A,FOXO3,SIRT1
Pathway	137917	Angiopoietin receptor Tie2-mediated signaling	1.201E-3	3	48	CDKN1A,PIK3R1,MAPK1
Pathway	138068	Signaling events mediated by Stem cell factor receptor (c-Kit)	1.516E-3	3	52	FOXO3,PIK3R1,SPRED1
Pathway	138069	S1P4 pathway	1.666E-3	2	14	GNA12,MAPK1
Pathway	137916	Regulation of retinoblastoma protein	1.878E-3	3	56	CDKN1A,MEF2C,SIRT1
Pathway	169353	Validated targets of C-MYC transcriptional repression	2.516E-3	3	62	CDKN1A,FOXO3,NFYA
Pathway	138048	Nongenotropic Androgen signaling	2.770E-3	2	18	PIK3R1,MAPK1
Pathway	138015	Regulation of cytoplasmic and nuclear SMAD2/3 signaling	3.087E-3	2	19	MAPK1,CTDSPL
Pathway	137950	Signaling events mediated by the Hedgehog family	4.135E-3	2	22	PIK3R1,PTCH1
Pathway	137969	Signaling events mediated by PRL	4.516E-3	2	23	CDKN1A,MAPK1
Pathway	137996	VEGFR3 signaling in lymphatic endothelium	4.516E-3	2	23	PIK3R1,MAPK1
Pathway	137963	Regulation of nuclear SMAD2/3 signaling	4.818E-3	3	78	CDKN1A,FOXO3,MEF2C
Pathway	137968	ALK1 signaling events	4.913E-3	2	24	MAPK1,SMAD5
Pathway	138059	S1P2 pathway	4.913E-3	2	24	GNA12,MAPK1
Pathway	137970	EGF receptor (ErbB1) signaling pathway	5.325E-3	2	25	PIK3R1,MAPK1
Pathway	137920	TRAIL signaling pathway	5.325E-3	2	25	PIK3R1,MAPK1
Pathway	138009	Plasma membrane estrogen receptor signaling	5.753E-3	2	26	HBEGF,PIK3R1
Pathway	138063	S1P3 pathway	6.654E-3	2	28	GNA12,MAPK1
Pathway	106459	NGF signalling via TRKA from the plasma membrane	2.331E-4	6	201	CDKN1A,FOXO3,HBEGF,PIK3R1,MAPK1,MEF2C
Pathway	477120	Signaling by SCF-KIT	3.323E-4	5	138	CDKN1A,FOXO3,HBEGF,PIK3R1,MAPK1
Pathway	160957	Downstream signaling of activated FGFR	4.043E-4	5	144	CDKN1A,FOXO3,HBEGF,PIK3R1,MAPK1
Pathway	530741	Signaling by ERBB4	4.728E-4	5	149	CDKN1A,FOXO3,HBEGF,PIK3R1,MAPK1
Pathway	685535	Constitutive PI3K/AKT Signaling in Cancer	5.606E-4	4	87	CDKN1A,FOXO3,HBEGF,PIK3R1
Pathway	106385	Downstream signal transduction	5.831E-4	5	156	CDKN1A,FOXO3,HBEGF,PIK3R1,MAPK1
Pathway	106343	Signaling by FGFR	6.179E-4	5	158	CDKN1A,FOXO3,HBEGF,PIK3R1,MAPK1
Pathway	685550	DAP12 signaling	6.359E-4	5	159	CDKN1A,FOXO3,HBEGF,PIK3R1,MAPK1
Pathway	530734	Signaling by ERBB2	6.359E-4	5	159	CDKN1A,FOXO3,HBEGF,PIK3R1,MAPK1
Pathway	685534	PI3K/AKT Signaling in Cancer	9.122E-4	4	99	CDKN1A,FOXO3,HBEGF,PIK3R1
Pathway	530743	PI3K events in ERBB4 signaling	9.122E-4	4	99	CDKN1A,FOXO3,HBEGF,PIK3R1
Pathway	530737	PI3K events in ERBB2 signaling	9.122E-4	4	99	CDKN1A,FOXO3,HBEGF,PIK3R1
Pathway	160961	PI-3K cascade	9.122E-4	4	99	CDKN1A,FOXO3,HBEGF,PIK3R1
Pathway	366239	PIP3 activates AKT signaling	9.122E-4	4	99	CDKN1A,FOXO3,HBEGF,PIK3R1
Pathway	645274	Signaling by FGFR in disease	9.310E-4	5	173	CDKN1A,FOXO3,HBEGF,PIK3R1,MAPK1
Pathway	106337	Signaling by EGFR	1.006E-3	5	176	CDKN1A,FOXO3,HBEGF,PIK3R1,MAPK1
Pathway	106474	PI3K/AKT activation	1.020E-3	4	102	CDKN1A,FOXO3,HBEGF,PIK3R1
Pathway	106341	GAB1 signalosome	1.058E-3	4	103	CDKN1A,FOXO3,HBEGF,PIK3R1
Pathway	530768	Signaling by EGFR in Cancer	1.058E-3	5	178	CDKN1A,FOXO3,HBEGF,PIK3R1,MAPK1
Pathway	106384	Signaling by PDGF	1.085E-3	5	179	CDKN1A,FOXO3,HBEGF,PIK3R1,MAPK1
Pathway	685549	DAP12 interactions	1.085E-3	5	179	CDKN1A,FOXO3,HBEGF,PIK3R1,MAPK1
Pathway	106439	Signalling by NGF	1.441E-3	6	284	CDKN1A,FOXO3,HBEGF,PIK3R1,MAPK1,MEF2C
Pathway	833825	Fc epsilon receptor (FCERI) signaling	1.551E-3	5	194	CDKN1A,FOXO3,HBEGF,PIK3R1,MAPK1
Pathway	833829	Role of LAT2/NTAL/LAB on calcium mobilization	2.098E-3	4	124	CDKN1A,FOXO3,HBEGF,PIK3R1
Pathway	530742	SHC1 events in ERBB4 signaling	3.770E-3	2	21	HBEGF,MAPK1
Pathway	106482	ERK/MAPK targets	3.770E-3	2	21	MAPK1,MEF2C
Pathway	530736	GRB2 events in ERBB2 signaling	4.516E-3	2	23	HBEGF,MAPK1
Pathway	106336	Signaling by BMP	4.516E-3	2	23	BMPR1A,SMAD5
Pathway	106481	Nuclear Events (kinase and transcription factor activation)	4.913E-3	2	24	MAPK1,MEF2C
Pathway	187170	Incretin Synthesis, Secretion, and Inactivation	4.913E-3	2	24	TCF7L2,ISL1
Pathway	530735	SHC1 events in ERBB2 signaling	5.325E-3	2	25	HBEGF,MAPK1
Pathway	576250	Downstream Signaling Events Of B Cell Receptor (BCR)	6.617E-3	4	171	CDKN1A,FOXO3,HBEGF,PIK3R1
Pathway	198802	Heart Development	1.773E-6	5	47	BMPR1A,HEY1,ISL1,MAPK1,MEF2C
Pathway	198885	IL-2 Signaling pathway	3.258E-5	4	42	FOXO3,MAPT,PIK3R1,MAPK1
Pathway	198774	TGF-beta Receptor Signaling Pathway	5.381E-5	6	154	CDKN1A,FOXO3,PIK3R1,NFYA,ZEB2,MEF2C
Pathway	198907	Energy Metabolism	5.556E-5	4	48	FOXO3,PPRC1,MEF2C,SIRT1
Pathway	198812	Endochondral Ossification	1.942E-4	4	66	BMPR1A,ADAMTS5,PTCH1,MEF2C
Pathway	712093	BDNF signaling pathway	4.043E-4	5	144	FOXO3,MAPT,PIK3R1,MAPK1,MEF2C
Pathway	672458	Signaling Pathways in Glioblastoma	4.074E-4	4	80	CDKN1A,FOXO3,PIK3R1,MAPK1
Pathway	198879	Delta-Notch Signaling Pathway	5.132E-4	4	85	HEY1,PIK3R1,MAPK1,MEF2C
Pathway	198845	Insulin Signaling	7.118E-4	5	163	FOXO3,PIK3R1,MAPK1,SLC2A1,SGK3
Pathway	198864	IL-6 Signaling Pathway	9.122E-4	4	99	FOXO3,MAPT,PIK3R1,MAPK1
Pathway	198857	IL-7 Signaling Pathway	9.312E-4	3	44	FOXO3,PIK3R1,MAPK1
Pathway	198844	ErbB signaling pathway	1.783E-3	3	55	CDKN1A,HBEGF,MAPK1
Pathway	672446	TSH signaling pathway	2.755E-3	3	64	PIK3R1,GNA12,MAPK1
Pathway	198826	Kit Receptor Signaling Pathway	3.138E-3	3	67	PIK3R1,SPRED1,MAPK1
Pathway	198770	IL-5 Signaling Pathway	3.411E-3	3	69	FOXO3,PIK3R1,MAPK1
Pathway	219801	Integrated Breast Cancer Pathway	4.578E-3	4	154	BMPR1A,NAB1,MAPK1,SIRT1
Pathway	198883	IL-9 Signaling Pathway	4.913E-3	2	24	PIK3R1,MAPK1
Pathway	198779	MAPK signaling pathway	5.965E-3	4	166	MAPT,GNA12,MAPK1,MEF2C
Pathway	198806	Androgen receptor signaling pathway	6.741E-3	3	88	CDKN1A,PIK3R1,SIRT1
Pathway	M10145	PTEN dependent cell cycle arrest and apoptosis	6.217E-5	3	18	FOXO3,PIK3R1,MAPK1
Pathway	M16811	Role of Erk5 in Neuronal Survival	6.217E-5	3	18	PIK3R1,MAPK1,MEF2C
Pathway	M2288	NFAT and Hypertrophy of the heart (Transcription in the broken heart)	8.857E-5	4	54	HBEGF,PIK3R1,MAPK1,MEF2C
Pathway	M4791	Regulation of eIF4e and p70 S6 Kinase	1.514E-4	3	24	PIK3R1,EIF4A2,MAPK1
Pathway	M14512	Influence of Ras and Rho proteins on G1 to S Transition	1.932E-4	3	26	CDKN1A,PIK3R1,MAPK1
Pathway	M17400	ALK in cardiac myocytes	5.582E-4	3	37	BMPR1A,MEF2C,SMAD5
Pathway	M13158	The IGF-1 Receptor and Longevity	1.917E-3	2	15	FOXO3,PIK3R1
Pathway	M16884	Role of nicotinic acetylcholine receptors in the regulation of apoptosis	2.185E-3	2	16	FOXO3,PIK3R1
Pathway	M8353	Human Cytomegalovirus and Map Kinase Pathways	2.469E-3	2	17	PIK3R1,MAPK1
Pathway	M15926	Trefoil Factors Initiate Mucosal Healing	3.770E-3	2	21	PIK3R1,MAPK1
Pathway	M15258	AKT Signaling Pathway	4.135E-3	2	22	FOXO3,PIK3R1
Pathway	M18719	Role of ERBB2 in Signal Transduction and Oncology	4.135E-3	2	22	PIK3R1,MAPK1
Pathway	M19613	Multiple antiapoptotic pathways from IGF-1R signaling lead to BAD phosphorylation	4.516E-3	2	23	PIK3R1,MAPK1
Pathway	M16563	mTOR Signaling Pathway	4.516E-3	2	23	PIK3R1,EIF4A2
Pathway	M11420	CTCF: First Multivalent Nuclear Factor	4.516E-3	2	23	PIK3R1,SMAD5
Pathway	M882	CXCR4 Signaling Pathway	4.913E-3	2	24	PIK3R1,MAPK1
Pathway	M6355	Erk and PI-3 Kinase Are Necessary for Collagen Binding in Corneal Epithelia	4.913E-3	2	24	PIK3R1,MAPK1
Pathway	M85	Regulation of BAD phosphorylation	5.753E-3	2	26	PIK3R1,MAPK1
Pathway	M10579	Phospholipids as signalling intermediaries	6.196E-3	2	27	PIK3R1,MAPK1
Pathway	M9070	Transcription factor CREB and its extracellular signals	6.196E-3	2	27	PIK3R1,MAPK1
Pathway	M9043	Growth Hormone Signaling Pathway	6.654E-3	2	28	PIK3R1,MAPK1
Pathway	M1718	Genes related to IL4 rceptor signaling in B lymphocytes	6.196E-3	2	27	PIK3R1,MAPK1
Pathway	P00052	TGF-beta signaling pathway	6.645E-4	4	91	BMPR1A,MAPK1,FKBP2,SMAD5
Pathway	P00059	p53 pathway	4.993E-3	3	79	CDKN1A,PIK3R1,SIRT1
Pathway	PW:0000343	altered Notch signaling involving promoters	4.416E-3	1	1	HEY1

miR-132/212 Network in Neuro2a cells

Category	ID	Name	p-value	Hit Count in Query List	Hit Count in Genome	Hit in Query List
GO: Molecular Function	GO:0000981	RNA polymerase II transcription factor activity, sequence-specific DNA binding	3.684E-5	16	655	SOX4,TCF4,ZNF148,CC2D1B,TEAD1,SIX4,ZEB2,MEIS2,BMPR1A,BTG2,CSRNP3,NFIB,FUBP1,PURB,NFYA,AEBP2
GO: Molecular Function	GO:0000982	transcription factor activity, RNA polymerase II core promoter proximal region sequence-specific binding	1.012E-4	11	363	SOX4,TCF4,ZNF148,CC2D1B,TEAD1,SIX4,MEIS2,BTG2,NFIB,NFYA,AEBP2
GO: Biological Process	GO:0031175	neuron projection development	6.493E-9	30	1211	KRAS,DPYSL3,FMR1,MYCBP2,MAPT,CALU,HBEGF,DUSP9,DAG1,SRGAP2,NREP,NRN1,MECP2,ZEB2,SIRT1,ACSL4,BTG2,PPP3CA,SEMA6D,CELSR3,NFIB,PRICKLE2,OLFM1,FGF7,SPRED1,SSH2,MAPK1,CAMSAP2,PEA15,SOS1
GO: Biological Process	GO:0048699	generation of neurons	7.511E-8	35	1759	SOX4,KRAS,TCF4,DPYSL3,FMR1,MYCBP2,MAPT,CALU,HBEGF,DUSP9,DAG1,SRGAP2,SIX4,NREP,NRN1,MECP2,ZEB2,SIRT1,ACSL4,BTG2,PPP3CA,SEMA6D,CELSR3,NFIB,PRICKLE2,OLFM1,FGF7,SPRED1,SSH2,MAPK1,CAMSAP2,PEA15,DNMT3A,SOS1
GO: Biological Process	GO:0048666	neuron development	9.234E-8	30	1363	KRAS,DPYSL3,FMR1,MYCBP2,MAPT,CALU,HBEGF,DUSP9,DAG1,SRGAP2,NREP,NRN1,MECP2,ZEB2,SIRT1,ACSL4,BTG2,PPP3CA,SEMA6D,CELSR3,NFIB,PRICKLE2,OLFM1,FGF7,SPRED1,SSH2,MAPK1,CAMSAP2,PEA15,SOS1
GO: Biological Process	GO:0030182	neuron differentiation	1.387E-7	33	1634	SOX4,KRAS,TCF4,DPYSL3,FMR1,MYCBP2,MAPT,CALU,HBEGF,DUSP9,DAG1,SRGAP2,NREP,NRN1,MECP2,ZEB2,SIRT1,ACSL4,BTG2,PPP3CA,SEMA6D,CELSR3,NFIB,PRICKLE2,OLFM1,FGF7,SPRED1,SSH2,MAPK1,CAMSAP2,PEA15,SOS1
GO: Biological Process	GO:0022008	neurogenesis	2.506E-7	35	1848	SOX4,KRAS,TCF4,DPYSL3,FMR1,MYCBP2,MAPT,CALU,HBEGF,DUSP9,DAG1,SRGAP2,SIX4,NREP,NRN1,MECP2,ZEB2,SIRT1,ACSL4,BMPR1A,BTG2,PPP3CA,SEMA6D,CELSR3,NFIB,PRICKLE2,OLFM1,FGF7,SPRED1,SSH2,MAPK1,CAMSAP2,PEA15,DNMT3A,SOS1
GO: Biological Process	GO:0061564	axon development	2.741E-7	22	827	KRAS,DPYSL3,MYCBP2,MAPT,CALU,HBEGF,DUSP9,DAG1,SRGAP2,NREP,NRN1,ZEB2,SEMA6D,CELSR3,NFIB,OLFM1,FGF7,SPRED1,SSH2,MAPK1,PEA15,SOS1
GO: Biological Process	GO:0030030	cell projection organization	5.010E-7	32	1642	KRAS,DPYSL3,RAPGEF6,FMR1,MYCBP2,MAPT,CALU,HBEGF,DUSP9,DAG1,SRGAP2,PFN2,NREP,NRN1,MECP2,ZEB2,SIRT1,ACSL4,BTG2,PPP3CA,SEMA6D,CELSR3,NFIB,PRICKLE2,OLFM1,FGF7,SPRED1,SSH2,MAPK1,CAMSAP2,PEA15,SOS1
GO: Biological Process	GO:0007265	Ras protein signal transduction	8.611E-7	16	485	KRAS,ARHGAP32,RAPGEF6,ABR,HBEGF,DUSP9,SHOC2,CRK,ARHGEF40,FGF7,SPRED1,GNA12,MAPK1,GNB1,PEA15,SOS1
GO: Biological Process	GO:0043085	positive regulation of catalytic activity	5.584E-6	29	1570	DOCK4,KRAS,ARHGAP32,RAPGEF6,TRIB2,ABR,BCL2L11,HBEGF,DUSP9,SRGAP2,TEAD1,PFN2,ZEB2,SIRT1,CRK,MAP3K3,AZIN1,ARHGEF40,SRGAP3,SNX18,OSBPL8,FGF7,GMFB,SPRED1,CTGF,MAPK1,PEA15,ARHGAP21,SOS1
GO: Biological Process	GO:0030336	negative regulation of cell migration	5.712E-6	10	215	DPYSL3,MIA3,ABR,DAG1,SRGAP2,PFN2,SEMA6D,SRGAP3,OSBPL8,OLFM1
GO: Biological Process	GO:0044093	positive regulation of molecular function	7.214E-6	32	1859	DOCK4,KRAS,ARHGAP32,RAPGEF6,TRIB2,ABR,BCL2L11,HBEGF,DUSP9,SPPL3,SRGAP2,TEAD1,PFN2,ZEB2,SIRT1,CRK,MAP3K3,AZIN1,SGK3,ARHGEF40,PPP3CA,SRGAP3,SNX18,OSBPL8,FGF7,GMFB,SPRED1,CTGF,MAPK1,PEA15,ARHGAP21,SOS1
GO: Biological Process	GO:2000146	negative regulation of cell motility	8.522E-6	10	225	DPYSL3,MIA3,ABR,DAG1,SRGAP2,PFN2,SEMA6D,SRGAP3,OSBPL8,OLFM1
GO: Biological Process	GO:0007409	axonogenesis	8.936E-6	19	794	KRAS,DPYSL3,MYCBP2,MAPT,HBEGF,DUSP9,DAG1,SRGAP2,NRN1,ZEB2,SEMA6D,NFIB,OLFM1,FGF7,SPRED1,SSH2,MAPK1,PEA15,SOS1
GO: Biological Process	GO:0000186	activation of MAPKK activity	1.341E-5	10	237	KRAS,HBEGF,DUSP9,CRK,MAP3K3,FGF7,SPRED1,MAPK1,PEA15,SOS1
GO: Biological Process	GO:0048667	cell morphogenesis involved in neuron differentiation	1.362E-5	20	894	KRAS,DPYSL3,MYCBP2,MAPT,HBEGF,DUSP9,DAG1,SRGAP2,NRN1,ZEB2,PPP3CA,SEMA6D,NFIB,OLFM1,FGF7,SPRED1,SSH2,MAPK1,PEA15,SOS1
GO: Biological Process	GO:0071363	cellular response to growth factor stimulus	2.124E-5	20	922	KRAS,TCF4,ABR,BCL2L11,CTDSPL2,HBEGF,DUSP9,NREP,SHOC2,SIRT1,CRK,BMPR1A,PIK3R1,FGF7,SPRED1,CTGF,MAPK1,PEA15,SMAD5,SOS1
GO: Biological Process	GO:0048812	neuron projection morphogenesis	2.366E-5	20	929	KRAS,DPYSL3,MYCBP2,MAPT,HBEGF,DUSP9,DAG1,SRGAP2,NRN1,ZEB2,PPP3CA,SEMA6D,NFIB,OLFM1,FGF7,SPRED1,SSH2,MAPK1,PEA15,SOS1
GO: Biological Process	GO:0051271	negative regulation of cellular component movement	2.692E-5	10	257	DPYSL3,MIA3,ABR,DAG1,SRGAP2,PFN2,SEMA6D,SRGAP3,OSBPL8,OLFM1
GO: Biological Process	GO:1902531	regulation of intracellular signal transduction	2.763E-5	29	1708	KRAS,ARHGAP32,TRIB2,ABR,BCL2L11,HBEGF,KSR2,DUSP9,SPPL3,DAG1,SRGAP2,SHOC2,ZEB2,SIRT1,CRK,MAP3K3,PIK3IP1,ARHGEF40,SRGAP3,OSBPL8,FGF7,SPRED1,GNA12,CTGF,MAPK1,PEA15,VDAC2,FBXW11,SOS1
GO: Biological Process	GO:0030334	regulation of cell migration	2.785E-5	17	711	DOCK4,DPYSL3,MIA3,ABR,HBEGF,DAG1,SRGAP2,PFN2,SGK3,SEMA6D,SRGAP3,PIK3R1,OSBPL8,OLFM1,FGF7,GNA12,MAPK1
GO: Biological Process	GO:0038095	Fc-epsilon receptor signaling pathway	2.875E-5	11	315	KRAS,HBEGF,DUSP9,PPP3CA,PIK3R1,FGF7,SPRED1,MAPK1,PEA15,FBXW11,SOS1
GO: Biological Process	GO:0008543	fibroblast growth factor receptor signaling pathway	2.960E-5	11	316	KRAS,HBEGF,DUSP9,SHOC2,PIK3R1,FGF7,SPRED1,CTGF,MAPK1,PEA15,SOS1
GO: Biological Process	GO:0070848	response to growth factor	2.971E-5	20	944	KRAS,TCF4,ABR,BCL2L11,CTDSPL2,HBEGF,DUSP9,NREP,SHOC2,SIRT1,CRK,BMPR1A,PIK3R1,FGF7,SPRED1,CTGF,MAPK1,PEA15,SMAD5,SOS1
GO: Biological Process	GO:0038093	Fc receptor signaling pathway	3.074E-5	12	377	KRAS,HBEGF,DUSP9,CRK,PPP3CA,PIK3R1,FGF7,SPRED1,MAPK1,PEA15,FBXW11,SOS1
GO: Biological Process	GO:0016477	cell migration	3.124E-5	24	1277	DOCK4,KRAS,DPYSL3,MIA3,ABR,MAPT,HBEGF,DAG1,SRGAP2,SIX4,PFN2,ZEB2,SGK3,SEMA6D,CELSR3,SRGAP3,PIK3R1,OSBPL8,OLFM1,FGF7,GNA12,CTGF,MAPK1,SOS1
GO: Biological Process	GO:0044344	cellular response to fibroblast growth factor stimulus	5.167E-5	11	336	KRAS,HBEGF,DUSP9,SHOC2,PIK3R1,FGF7,SPRED1,CTGF,MAPK1,PEA15,SOS1
GO: Biological Process	GO:2000145	regulation of cell motility	5.516E-5	17	751	DOCK4,DPYSL3,MIA3,ABR,HBEGF,DAG1,SRGAP2,PFN2,SGK3,SEMA6D,SRGAP3,PIK3R1,OSBPL8,OLFM1,FGF7,GNA12,MAPK1
GO: Biological Process	GO:0051270	regulation of cellular component movement	5.893E-5	18	832	DOCK4,DPYSL3,MIA3,ABR,MAPT,HBEGF,DAG1,SRGAP2,PFN2,SGK3,SEMA6D,SRGAP3,PIK3R1,OSBPL8,OLFM1,FGF7,GNA12,MAPK1
GO: Biological Process	GO:0071774	response to fibroblast growth factor	5.900E-5	11	341	KRAS,HBEGF,DUSP9,SHOC2,PIK3R1,FGF7,SPRED1,CTGF,MAPK1,PEA15,SOS1
GO: Biological Process	GO:0048011	neurotrophin TRK receptor signaling pathway	5.997E-5	12	404	KRAS,ABR,BCL2L11,HBEGF,DUSP9,CRK,PIK3R1,FGF7,SPRED1,MAPK1,PEA15,SOS1
GO: Biological Process	GO:0038179	neurotrophin signaling pathway	6.436E-5	12	407	KRAS,ABR,BCL2L11,HBEGF,DUSP9,CRK,PIK3R1,FGF7,SPRED1,MAPK1,PEA15,SOS1
GO: Biological Process	GO:0040013	negative regulation of locomotion	6.441E-5	10	285	DPYSL3,MIA3,ABR,DAG1,SRGAP2,PFN2,SEMA6D,SRGAP3,OSBPL8,OLFM1
GO: Biological Process	GO:0008286	insulin receptor signaling pathway	6.550E-5	11	345	KRAS,HBEGF,DUSP9,SIRT1,PIK3R1,OSBPL8,FGF7,SPRED1,MAPK1,PEA15,SOS1
GO: Biological Process	GO:0043434	response to peptide hormone	6.679E-5	15	614	KRAS,HBEGF,DUSP9,DAG1,SIRT1,BTG2,PIK3R1,OSBPL8,FGF7,SPRED1,CTGF,MAPK1,GNB1,PEA15,SOS1
GO: Biological Process	GO:0007264	small GTPase mediated signal transduction	7.996E-5	19	932	DOCK4,KRAS,ARHGAP32,RAPGEF6,ABR,HBEGF,DUSP9,SRGAP2,SHOC2,CRK,ARHGEF40,SRGAP3,FGF7,SPRED1,GNA12,MAPK1,GNB1,PEA15,SOS1
GO: Biological Process	GO:0034504	protein localization to nucleus	9.105E-5	11	358	HNRNPM,SPPL3,DAG1,SIX4,SIRT1,BMPR1A,PPP3CA,PIK3R1,OSBPL8,MAPK1,FBXW11
GO: Biological Process	GO:0048010	vascular endothelial growth factor receptor signaling pathway	9.324E-5	10	298	KRAS,HBEGF,DUSP9,CRK,PIK3R1,FGF7,SPRED1,MAPK1,PEA15,SOS1
GO: Biological Process	GO:0045595	regulation of cell differentiation	1.097E-4	27	1656	KRAS,TCF4,DPYSL3,TRIB2,MAPT,DAG1,SRGAP2,TEAD1,SIX4,NREP,MECP2,ZEB2,SIRT1,MEIS2,MEX3C,BMPR1A,PPP3CA,SEMA6D,PIK3R1,PURB,NFYA,OLFM1,CTGF,SSH2,MAPK1,SMAD5,SOS1
GO: Biological Process	GO:0060425	lung morphogenesis	1.151E-4	5	63	KRAS,DAG1,NFIB,FGF7,MAPK1
GO: Biological Process	GO:0051674	localization of cell	1.197E-4	24	1391	DOCK4,KRAS,DPYSL3,MIA3,ABR,MAPT,HBEGF,DAG1,SRGAP2,SIX4,PFN2,ZEB2,SGK3,SEMA6D,CELSR3,SRGAP3,PIK3R1,OSBPL8,OLFM1,FGF7,GNA12,CTGF,MAPK1,SOS1
GO: Biological Process	GO:0048870	cell motility	1.197E-4	24	1391	DOCK4,KRAS,DPYSL3,MIA3,ABR,MAPT,HBEGF,DAG1,SRGAP2,SIX4,PFN2,ZEB2,SGK3,SEMA6D,CELSR3,SRGAP3,PIK3R1,OSBPL8,OLFM1,FGF7,GNA12,CTGF,MAPK1,SOS1
GO: Biological Process	GO:1901652	response to peptide	1.236E-4	15	649	KRAS,HBEGF,DUSP9,DAG1,SIRT1,BTG2,PIK3R1,OSBPL8,FGF7,SPRED1,CTGF,MAPK1,GNB1,PEA15,SOS1
GO: Biological Process	GO:0002062	chondrocyte differentiation	1.264E-4	6	102	MIA3,MEX3C,TGFBI,BMPR1A,NFIB,CTGF
GO: Biological Process	GO:0031399	regulation of protein modification process	1.630E-4	29	1886	SOX4,KRAS,TRIB2,KDM5A,HBEGF,KSR2,DUSP9,SPPL3,DAG1,TEAD1,PFN2,ZFYVE1,MECP2,ZEB2,SIRT1,CRK,MAP3K3,BMPR1A,CELSR3,CSRNP3,PIK3R1,OSBPL8,FGF7,GMFB,SPRED1,CTGF,MAPK1,PEA15,SOS1
GO: Biological Process	GO:0006357	regulation of transcription from RNA polymerase II promoter	1.630E-4	29	1886	SOX4,TCF4,ZNF148,ARID4B,KDM5A,CTDSPL2,CC2D1B,TEAD1,SIX4,MECP2,ZEB2,SIRT1,MEIS2,CRK,BMPR1A,BTG2,PPP3CA,CSRNP3,NFIB,PIK3R1,FUBP1,ARID2,PURB,NFYA,AEBP2,MEPCE,SMAD5,DNMT3A,BRWD1
GO: Biological Process	GO:0006913	nucleocytoplasmic transport	1.655E-4	12	450	SOX4,SRSF10,CTDSPL2,SPPL3,DAG1,SIRT1,BMPR1A,PPP3CA,PIK3R1,ANP32A,MAPK1,FBXW11
GO: Biological Process	GO:0040012	regulation of locomotion	1.671E-4	17	823	DOCK4,DPYSL3,MIA3,ABR,HBEGF,DAG1,SRGAP2,PFN2,SGK3,SEMA6D,SRGAP3,PIK3R1,OSBPL8,OLFM1,FGF7,GNA12,MAPK1
GO: Biological Process	GO:0007411	axon guidance	1.686E-4	14	593	KRAS,DPYSL3,MYCBP2,HBEGF,DUSP9,DAG1,SRGAP2,SEMA6D,NFIB,FGF7,SPRED1,MAPK1,PEA15,SOS1
GO: Biological Process	GO:0097485	neuron projection guidance	1.686E-4	14	593	KRAS,DPYSL3,MYCBP2,HBEGF,DUSP9,DAG1,SRGAP2,SEMA6D,NFIB,FGF7,SPRED1,MAPK1,PEA15,SOS1
GO: Biological Process	GO:0051169	nuclear transport	1.833E-4	12	455	SOX4,SRSF10,CTDSPL2,SPPL3,DAG1,SIRT1,BMPR1A,PPP3CA,PIK3R1,ANP32A,MAPK1,FBXW11
GO: Biological Process	GO:0010628	positive regulation of gene expression	1.955E-4	28	1810	SOX4,KRAS,TCF4,ZNF148,FMR1,ARID4B,KDM5A,TEAD1,SIX4,MECP2,ZEB2,SIRT1,MEIS2,BMPR1A,BTG2,PPP3CA,CSRNP3,NFIB,PIK3R1,FUBP1,ARID2,NFYA,OLFM1,FGF7,CTGF,MAPK1,SMAD5,FBXW11
GO: Biological Process	GO:0007435	salivary gland morphogenesis	2.118E-4	4	39	DAG1,NFIB,BTBD7,FGF7
GO: Biological Process	GO:0010629	negative regulation of gene expression	2.132E-4	26	1630	TCF4,SRSF10,TRIB2,ZNF148,FMR1,ARID4B,KDM5A,PTBP2,CC2D1B,FOXN3,SIX4,MECP2,ZEB2,SIRT1,MEIS2,WHSC1L1,FXR1,BTG2,NFIB,PURB,AEBP2,MEPCE,OLFM1,CTGF,FBXW11,DNMT3A
GO: Biological Process	GO:0002768	immune response-regulating cell surface receptor signaling pathway	2.158E-4	13	534	KRAS,HBEGF,DUSP9,SPPL3,CRK,PPP3CA,PIK3R1,FGF7,SPRED1,MAPK1,PEA15,FBXW11,SOS1
GO: Biological Process	GO:0031401	positive regulation of protein modification process	2.355E-4	22	1274	SOX4,KRAS,TRIB2,HBEGF,KSR2,DUSP9,SPPL3,TEAD1,PFN2,ZEB2,SIRT1,CRK,MAP3K3,BMPR1A,PIK3R1,OSBPL8,FGF7,SPRED1,CTGF,MAPK1,PEA15,SOS1
GO: Biological Process	GO:0032270	positive regulation of cellular protein metabolic process	2.357E-4	25	1547	SOX4,KRAS,TRIB2,FMR1,BCL2L11,HBEGF,KSR2,DUSP9,SPPL3,TEAD1,PFN2,ZEB2,SIRT1,CRK,MAP3K3,BMPR1A,PIK3R1,OSBPL8,FGF7,SPRED1,CTGF,MAPK1,PEA15,FBXW11,SOS1
GO: Biological Process	GO:0048858	cell projection morphogenesis	2.420E-4	21	1188	KRAS,DPYSL3,MYCBP2,MAPT,HBEGF,DUSP9,DAG1,SRGAP2,NRN1,ZEB2,PPP3CA,SEMA6D,CELSR3,NFIB,OLFM1,FGF7,SPRED1,SSH2,MAPK1,PEA15,SOS1
GO: Biological Process	GO:0040011	locomotion	2.462E-4	29	1932	DOCK4,KRAS,DPYSL3,MIA3,ABR,MYCBP2,MAPT,HBEGF,DUSP9,DAG1,SRGAP2,SIX4,PFN2,ZEB2,SGK3,SEMA6D,CELSR3,NFIB,SRGAP3,PIK3R1,OSBPL8,OLFM1,FGF7,SPRED1,GNA12,CTGF,MAPK1,PEA15,SOS1
GO: Biological Process	GO:0006470	protein dephosphorylation	2.845E-4	10	342	CTDSPL2,DUSP9,SPPL3,ZFYVE1,PTPN4,PPP3CA,CSRNP3,SPRED1,SSH2,FBXW11
GO: Biological Process	GO:0051254	positive regulation of RNA metabolic process	3.009E-4	25	1572	SOX4,KRAS,TCF4,ZNF148,ARID4B,KDM5A,TEAD1,SIX4,MECP2,ZEB2,SIRT1,MEIS2,BMPR1A,BTG2,PPP3CA,CSRNP3,NFIB,PIK3R1,FUBP1,ARID2,NFYA,FGF7,MAPK1,SMAD5,FBXW11
GO: Biological Process	GO:0032869	cellular response to insulin stimulus	3.019E-4	11	411	KRAS,HBEGF,DUSP9,SIRT1,PIK3R1,OSBPL8,FGF7,SPRED1,MAPK1,PEA15,SOS1
GO: Biological Process	GO:0032990	cell part morphogenesis	3.031E-4	21	1208	KRAS,DPYSL3,MYCBP2,MAPT,HBEGF,DUSP9,DAG1,SRGAP2,NRN1,ZEB2,PPP3CA,SEMA6D,CELSR3,NFIB,OLFM1,FGF7,SPRED1,SSH2,MAPK1,PEA15,SOS1
GO: Biological Process	GO:0000904	cell morphogenesis involved in differentiation	3.061E-4	20	1121	KRAS,DPYSL3,MYCBP2,MAPT,HBEGF,DUSP9,DAG1,SRGAP2,NRN1,ZEB2,PPP3CA,SEMA6D,NFIB,OLFM1,FGF7,SPRED1,SSH2,MAPK1,PEA15,SOS1
GO: Biological Process	GO:0007431	salivary gland development	3.104E-4	4	43	DAG1,NFIB,BTBD7,FGF7
GO: Biological Process	GO:0006349	regulation of gene expression by genetic imprinting	3.201E-4	3	18	ARID4B,MECP2,DNMT3A
GO: Biological Process	GO:0051531	NFAT protein import into nucleus	3.201E-4	3	18	SPPL3,PPP3CA,PIK3R1
GO: Biological Process	GO:0045860	positive regulation of protein kinase activity	3.496E-4	14	637	KRAS,HBEGF,DUSP9,TEAD1,ZEB2,SIRT1,CRK,MAP3K3,OSBPL8,FGF7,SPRED1,MAPK1,PEA15,SOS1
GO: Biological Process	GO:0046822	regulation of nucleocytoplasmic transport	3.528E-4	8	228	SOX4,CTDSPL2,SPPL3,SIRT1,BMPR1A,PPP3CA,PIK3R1,FBXW11
GO: Biological Process	GO:0051056	regulation of small GTPase mediated signal transduction	3.644E-4	9	289	KRAS,ARHGAP32,ABR,SRGAP2,SHOC2,CRK,ARHGEF40,SRGAP3,SOS1
GO: Biological Process	GO:1903508	positive regulation of nucleic acid-templated transcription	3.884E-4	24	1505	SOX4,KRAS,TCF4,ZNF148,ARID4B,KDM5A,TEAD1,SIX4,MECP2,ZEB2,SIRT1,MEIS2,BMPR1A,PPP3CA,CSRNP3,NFIB,PIK3R1,FUBP1,ARID2,NFYA,FGF7,MAPK1,SMAD5,FBXW11
GO: Biological Process	GO:0045893	positive regulation of transcription, DNA-templated	3.884E-4	24	1505	SOX4,KRAS,TCF4,ZNF148,ARID4B,KDM5A,TEAD1,SIX4,MECP2,ZEB2,SIRT1,MEIS2,BMPR1A,PPP3CA,CSRNP3,NFIB,PIK3R1,FUBP1,ARID2,NFYA,FGF7,MAPK1,SMAD5,FBXW11
GO: Biological Process	GO:0007010	cytoskeleton organization	3.888E-4	20	1142	KRAS,DPYSL3,ABR,MYCBP2,MAPT,DAG1,SRGAP2,SIX4,PFN2,CRK,AP1G1,PIK3R1,FGF7,GMFB,TLN2,CTGF,SSH2,MAPK1,CAMSAP2,BRWD1
GO: Biological Process	GO:0043549	regulation of kinase activity	3.892E-4	18	969	KRAS,TRIB2,HBEGF,DUSP9,TEAD1,ZEB2,SIRT1,CRK,MAP3K3,PIK3IP1,PIK3R1,OSBPL8,FGF7,GMFB,SPRED1,MAPK1,PEA15,SOS1
GO: Biological Process	GO:0051093	negative regulation of developmental process	4.157E-4	17	890	KRAS,TCF4,DPYSL3,TRIB2,ABR,SRGAP2,SIX4,MECP2,SIRT1,MEIS2,BMPR1A,PPP3CA,SEMA6D,NFIB,PIK3R1,OLFM1,MAPK1
GO: Biological Process	GO:0001934	positive regulation of protein phosphorylation	4.186E-4	19	1061	KRAS,HBEGF,KSR2,DUSP9,TEAD1,PFN2,ZEB2,SIRT1,CRK,MAP3K3,BMPR1A,PIK3R1,OSBPL8,FGF7,SPRED1,CTGF,MAPK1,PEA15,SOS1
GO: Biological Process	GO:0048863	stem cell differentiation	4.443E-4	9	297	DAG1,TEAD1,SIX4,ZEB2,BMPR1A,SEMA6D,NFYA,OLFM1,MAPK1
GO: Biological Process	GO:0071375	cellular response to peptide hormone stimulus	4.795E-4	12	506	KRAS,HBEGF,DUSP9,SIRT1,PIK3R1,OSBPL8,FGF7,SPRED1,MAPK1,GNB1,PEA15,SOS1
GO: Biological Process	GO:0051248	negative regulation of protein metabolic process	4.852E-4	20	1162	SOX4,TRIB2,FMR1,MYCBP2,KDM5A,HBEGF,DUSP9,DAG1,ZFYVE1,MECP2,SIRT1,AZIN1,FXR1,BTG2,CSRNP3,PIK3R1,PURB,OLFM1,GMFB,SPRED1
GO: Biological Process	GO:0010243	response to organonitrogen compound	4.900E-4	18	988	KRAS,HBEGF,DUSP9,DAG1,SIRT1,BTG2,PPP3CA,PIK3R1,OSBPL8,FGF7,SPRED1,CTGF,MAPK1,GNB1,PEA15,HOMER1,DNMT3A,SOS1
GO: Biological Process	GO:0040007	growth	4.974E-4	19	1076	GPD2,BCL2L11,MAPT,HBEGF,SIX4,NRN1,ZEB2,SIRT1,MEX3C,SGK3,ACSL4,BMPR1A,SEMA6D,FUBP1,OLFM1,FGF7,CTGF,SOS1,LARGE
GO: Biological Process	GO:0051347	positive regulation of transferase activity	5.019E-4	15	740	KRAS,TRIB2,HBEGF,DUSP9,TEAD1,ZEB2,SIRT1,CRK,MAP3K3,OSBPL8,FGF7,SPRED1,MAPK1,PEA15,SOS1
GO: Biological Process	GO:1902680	positive regulation of RNA biosynthetic process	5.022E-4	24	1532	SOX4,KRAS,TCF4,ZNF148,ARID4B,KDM5A,TEAD1,SIX4,MECP2,ZEB2,SIRT1,MEIS2,BMPR1A,PPP3CA,CSRNP3,NFIB,PIK3R1,FUBP1,ARID2,NFYA,FGF7,MAPK1,SMAD5,FBXW11
GO: Biological Process	GO:1900078	positive regulation of cellular response to insulin stimulus	5.131E-4	3	21	SIRT1,PIK3R1,OSBPL8
GO: Biological Process	GO:0033674	positive regulation of kinase activity	5.283E-4	14	664	KRAS,HBEGF,DUSP9,TEAD1,ZEB2,SIRT1,CRK,MAP3K3,OSBPL8,FGF7,SPRED1,MAPK1,PEA15,SOS1
GO: Biological Process	GO:0048589	developmental growth	5.283E-4	14	664	GPD2,BCL2L11,MAPT,HBEGF,SIX4,NRN1,ZEB2,MEX3C,BMPR1A,SEMA6D,OLFM1,FGF7,SOS1,LARGE
GO: Biological Process	GO:0010557	positive regulation of macromolecule biosynthetic process	5.597E-4	26	1735	SOX4,KRAS,TCF4,ZNF148,FMR1,ARID4B,KDM5A,TEAD1,SIX4,MECP2,ZEB2,SIRT1,MEIS2,BMPR1A,PPP3CA,CSRNP3,NFIB,PIK3R1,FUBP1,ARID2,NFYA,FGF7,CTGF,MAPK1,SMAD5,FBXW11
GO: Biological Process	GO:0034773	histone H4-K20 trimethylation	5.621E-4	2	5	ARID4B,KMT5B
GO: Biological Process	GO:0001880	Mullerian duct regression	5.621E-4	2	5	BMPR1A,SMAD5
GO: Biological Process	GO:0019220	regulation of phosphate metabolic process	5.679E-4	27	1834	KRAS,TRIB2,HBEGF,KSR2,DUSP9,SPPL3,DAG1,TEAD1,PFN2,ZFYVE1,ZEB2,SIRT1,CRK,MAP3K3,PIK3IP1,BMPR1A,CELSR3,CSRNP3,PIK3R1,OSBPL8,FGF7,GMFB,SPRED1,CTGF,MAPK1,PEA15,SOS1
GO: Biological Process	GO:0060541	respiratory system development	5.836E-4	8	246	KRAS,DAG1,SIX4,BMPR1A,NFIB,FGF7,CTGF,MAPK1
GO: Biological Process	GO:0051247	positive regulation of protein metabolic process	5.918E-4	25	1645	SOX4,KRAS,TRIB2,FMR1,BCL2L11,HBEGF,KSR2,DUSP9,SPPL3,TEAD1,PFN2,ZEB2,SIRT1,CRK,MAP3K3,BMPR1A,PIK3R1,OSBPL8,FGF7,SPRED1,CTGF,MAPK1,PEA15,FBXW11,SOS1
GO: Biological Process	GO:1904589	regulation of protein import	6.208E-4	7	190	HNRNPM,SPPL3,SIRT1,BMPR1A,PPP3CA,PIK3R1,FBXW11
GO: Biological Process	GO:0045596	negative regulation of cell differentiation	6.209E-4	14	675	KRAS,DPYSL3,TRIB2,SRGAP2,SIX4,MECP2,SIRT1,MEIS2,BMPR1A,PPP3CA,SEMA6D,PIK3R1,OLFM1,MAPK1
GO: Biological Process	GO:1901653	cellular response to peptide	6.312E-4	12	522	KRAS,HBEGF,DUSP9,SIRT1,PIK3R1,OSBPL8,FGF7,SPRED1,MAPK1,GNB1,PEA15,SOS1
GO: Biological Process	GO:0051174	regulation of phosphorus metabolic process	6.334E-4	27	1847	KRAS,TRIB2,HBEGF,KSR2,DUSP9,SPPL3,DAG1,TEAD1,PFN2,ZFYVE1,ZEB2,SIRT1,CRK,MAP3K3,PIK3IP1,BMPR1A,CELSR3,CSRNP3,PIK3R1,OSBPL8,FGF7,GMFB,SPRED1,CTGF,MAPK1,PEA15,SOS1
GO: Biological Process	GO:0042327	positive regulation of phosphorylation	6.646E-4	19	1102	KRAS,HBEGF,KSR2,DUSP9,TEAD1,PFN2,ZEB2,SIRT1,CRK,MAP3K3,BMPR1A,PIK3R1,OSBPL8,FGF7,SPRED1,CTGF,MAPK1,PEA15,SOS1
GO: Biological Process	GO:0007167	enzyme linked receptor protein signaling pathway	6.902E-4	21	1286	KRAS,TCF4,ABR,BCL2L11,CTDSPL2,HBEGF,DUSP9,NREP,SHOC2,SIRT1,CRK,BMPR1A,PIK3R1,OSBPL8,FGF7,SPRED1,CTGF,MAPK1,PEA15,SMAD5,SOS1
GO: Biological Process	GO:0051216	cartilage development	7.237E-4	7	195	MIA3,MEX3C,TGFBI,BMPR1A,NFIB,CTGF,SMAD5
GO: Biological Process	GO:0032868	response to insulin	7.724E-4	11	460	KRAS,HBEGF,DUSP9,SIRT1,PIK3R1,OSBPL8,FGF7,SPRED1,MAPK1,PEA15,SOS1
GO: Biological Process	GO:0001932	regulation of protein phosphorylation	7.939E-4	23	1487	KRAS,TRIB2,HBEGF,KSR2,DUSP9,DAG1,TEAD1,PFN2,ZEB2,SIRT1,CRK,MAP3K3,BMPR1A,CELSR3,PIK3R1,OSBPL8,FGF7,GMFB,SPRED1,CTGF,MAPK1,PEA15,SOS1
GO: Biological Process	GO:0060485	mesenchyme development	8.165E-4	8	259	DAG1,TEAD1,SIX4,ZEB2,BMPR1A,SEMA6D,OLFM1,MAPK1
GO: Biological Process	GO:0042325	regulation of phosphorylation	8.427E-4	24	1589	KRAS,TRIB2,HBEGF,KSR2,DUSP9,DAG1,TEAD1,PFN2,ZEB2,SIRT1,CRK,MAP3K3,PIK3IP1,BMPR1A,CELSR3,PIK3R1,OSBPL8,FGF7,GMFB,SPRED1,CTGF,MAPK1,PEA15,SOS1
GO: Biological Process	GO:0023014	signal transduction by protein phosphorylation	8.443E-4	17	948	KRAS,TRIB2,HBEGF,KSR2,DUSP9,DAG1,ZEB2,CRK,MAP3K3,BMPR1A,FGF7,SPRED1,CTGF,MAPK1,PEA15,FBXW11,SOS1
GO: Biological Process	GO:1900087	positive regulation of G1/S transition of mitotic cell cycle	8.678E-4	3	25	UBE2E2,MEPCE,LSM11
GO: Biological Process	GO:1902533	positive regulation of intracellular signal transduction	8.695E-4	18	1038	KRAS,BCL2L11,HBEGF,KSR2,DUSP9,SPPL3,SHOC2,ZEB2,SIRT1,CRK,MAP3K3,OSBPL8,FGF7,SPRED1,CTGF,MAPK1,PEA15,SOS1
GO: Biological Process	GO:0007517	muscle organ development	8.720E-4	10	395	HBEGF,CD164,SIX4,SIRT1,FXR1,BTG2,PPP3CA,KMT5B,HOMER1,LARGE
GO: Biological Process	GO:0007417	central nervous system development	9.260E-4	17	956	SOX4,KRAS,ZNF148,FMR1,ABR,BCL2L11,MYCBP2,MAPT,SRGAP2,MECP2,ZEB2,BMPR1A,BTG2,SEMA6D,NFIB,MAPK1,SOS1
GO: Biological Process	GO:0045937	positive regulation of phosphate metabolic process	9.281E-4	20	1224	KRAS,HBEGF,KSR2,DUSP9,SPPL3,TEAD1,PFN2,ZEB2,SIRT1,CRK,MAP3K3,BMPR1A,PIK3R1,OSBPL8,FGF7,SPRED1,CTGF,MAPK1,PEA15,SOS1
GO: Biological Process	GO:0010562	positive regulation of phosphorus metabolic process	9.281E-4	20	1224	KRAS,HBEGF,KSR2,DUSP9,SPPL3,TEAD1,PFN2,ZEB2,SIRT1,CRK,MAP3K3,BMPR1A,PIK3R1,OSBPL8,FGF7,SPRED1,CTGF,MAPK1,PEA15,SOS1
GO: Biological Process	GO:0007169	transmembrane receptor protein tyrosine kinase signaling pathway	9.584E-4	17	959	KRAS,TCF4,ABR,BCL2L11,HBEGF,DUSP9,SHOC2,SIRT1,CRK,PIK3R1,OSBPL8,FGF7,SPRED1,CTGF,MAPK1,PEA15,SOS1
GO: Biological Process	GO:0060445	branching involved in salivary gland morphogenesis	9.756E-4	3	26	DAG1,BTBD7,FGF7
GO: Biological Process	GO:0021952	central nervous system projection neuron axonogenesis	9.756E-4	3	26	MYCBP2,ZEB2,NFIB
GO: Biological Process	GO:0016049	cell growth	9.781E-4	12	549	MAPT,HBEGF,NRN1,ZEB2,SIRT1,MEX3C,SGK3,ACSL4,SEMA6D,FUBP1,OLFM1,CTGF
GO: Biological Process	GO:0051172	negative regulation of nitrogen compound metabolic process	1.038E-3	24	1613	TCF4,SRSF10,TRIB2,ZNF148,FMR1,KDM5A,PTBP2,CC2D1B,FOXN3,SIX4,MECP2,ZEB2,SIRT1,MEIS2,WHSC1L1,FXR1,BTG2,NFIB,PURB,AEBP2,MEPCE,OLFM1,FBXW11,DNMT3A
GO: Biological Process	GO:0035272	exocrine system development	1.042E-3	4	59	DAG1,NFIB,BTBD7,FGF7
GO: Biological Process	GO:0006468	protein phosphorylation	1.096E-3	27	1915	KRAS,TRIB2,HBEGF,CDK19,KSR2,DUSP9,DAG1,TEAD1,PFN2,ZEB2,SIRT1,CRK,MAP3K3,SGK3,BMPR1A,CELSR3,PIK3R1,OSBPL8,FGF7,GMFB,SPRED1,CTGF,MAPK1,PEA15,SMAD5,FBXW11,SOS1
GO: Biological Process	GO:0043410	positive regulation of MAPK cascade	1.096E-3	13	635	KRAS,HBEGF,KSR2,DUSP9,ZEB2,CRK,MAP3K3,FGF7,SPRED1,CTGF,MAPK1,PEA15,SOS1
GO: Biological Process	GO:0017038	protein import	1.113E-3	9	338	HNRNPM,SPPL3,DAG1,SIRT1,BMPR1A,PPP3CA,PIK3R1,MAPK1,FBXW11
GO: Biological Process	GO:0032147	activation of protein kinase activity	1.128E-3	11	482	KRAS,HBEGF,DUSP9,CRK,MAP3K3,OSBPL8,FGF7,SPRED1,MAPK1,PEA15,SOS1
GO: Biological Process	GO:2000113	negative regulation of cellular macromolecule biosynthetic process	1.140E-3	22	1432	TCF4,TRIB2,ZNF148,FMR1,KDM5A,HBEGF,CC2D1B,FOXN3,SIX4,MECP2,ZEB2,SIRT1,MEIS2,WHSC1L1,FXR1,BTG2,NFIB,PURB,AEBP2,MEPCE,FBXW11,DNMT3A
GO: Biological Process	GO:2000794	regulation of epithelial cell proliferation involved in lung morphogenesis	1.169E-3	2	7	NFIB,FGF7
GO: Biological Process	GO:0030324	lung development	1.211E-3	7	213	KRAS,DAG1,BMPR1A,NFIB,FGF7,CTGF,MAPK1
GO: Biological Process	GO:1900180	regulation of protein localization to nucleus	1.244E-3	7	214	HNRNPM,SPPL3,SIRT1,BMPR1A,PPP3CA,PIK3R1,FBXW11
GO: Biological Process	GO:0051253	negative regulation of RNA metabolic process	1.313E-3	21	1353	TCF4,SRSF10,TRIB2,ZNF148,KDM5A,PTBP2,CC2D1B,FOXN3,SIX4,MECP2,ZEB2,SIRT1,MEIS2,WHSC1L1,BTG2,NFIB,PURB,AEBP2,MEPCE,FBXW11,DNMT3A
GO: Biological Process	GO:1901698	response to nitrogen compound	1.320E-3	18	1077	KRAS,HBEGF,DUSP9,DAG1,SIRT1,BTG2,PPP3CA,PIK3R1,OSBPL8,FGF7,SPRED1,CTGF,MAPK1,GNB1,PEA15,HOMER1,DNMT3A,SOS1
GO: Biological Process	GO:1904591	positive regulation of protein import	1.334E-3	5	107	HNRNPM,SPPL3,BMPR1A,PPP3CA,PIK3R1
GO: Biological Process	GO:0030323	respiratory tube development	1.348E-3	7	217	KRAS,DAG1,BMPR1A,NFIB,FGF7,CTGF,MAPK1
GO: Biological Process	GO:0071514	genetic imprinting	1.348E-3	3	29	ARID4B,MECP2,DNMT3A
GO: Biological Process	GO:1902808	positive regulation of cell cycle G1/S phase transition	1.348E-3	3	29	UBE2E2,MEPCE,LSM11
GO: Biological Process	GO:0045944	positive regulation of transcription from RNA polymerase II promoter	1.362E-3	18	1080	SOX4,TCF4,ZNF148,ARID4B,TEAD1,SIX4,ZEB2,SIRT1,MEIS2,BMPR1A,PPP3CA,CSRNP3,NFIB,PIK3R1,FUBP1,ARID2,NFYA,SMAD5
GO: Biological Process	GO:0046661	male sex differentiation	1.436E-3	6	161	ARID4B,BCL2L11,SIX4,CSDE1,BMPR1A,SMAD5
GO: Biological Process	GO:0045859	regulation of protein kinase activity	1.495E-3	16	911	KRAS,TRIB2,HBEGF,DUSP9,TEAD1,ZEB2,SIRT1,CRK,MAP3K3,OSBPL8,FGF7,GMFB,SPRED1,MAPK1,PEA15,SOS1
GO: Biological Process	GO:0032989	cellular component morphogenesis	1.536E-3	24	1660	KRAS,DPYSL3,MYCBP2,MAPT,HBEGF,DUSP9,DAG1,SRGAP2,SIX4,NRN1,ZEB2,PPP3CA,SEMA6D,CELSR3,NFIB,OLFM1,FGF7,SPRED1,GNA12,SSH2,MAPK1,PEA15,BRWD1,SOS1
GO: Biological Process	GO:0051547	regulation of keratinocyte migration	1.550E-3	2	8	HBEGF,FGF7
GO: Biological Process	GO:0051549	positive regulation of keratinocyte migration	1.550E-3	2	8	HBEGF,FGF7
GO: Biological Process	GO:0071679	commissural neuron axon guidance	1.550E-3	2	8	DAG1,NFIB
GO: Biological Process	GO:0051338	regulation of transferase activity	1.588E-3	18	1095	KRAS,TRIB2,HBEGF,DUSP9,TEAD1,ZEB2,SIRT1,CRK,MAP3K3,PIK3IP1,PIK3R1,OSBPL8,FGF7,GMFB,SPRED1,MAPK1,PEA15,SOS1
GO: Biological Process	GO:0043414	macromolecule methylation	1.608E-3	8	288	ARID4B,MECP2,SIRT1,WHSC1L1,BTG2,KMT5B,MEPCE,DNMT3A
GO: Biological Process	GO:0000165	MAPK cascade	1.617E-3	16	918	KRAS,TRIB2,HBEGF,KSR2,DUSP9,DAG1,ZEB2,CRK,MAP3K3,FGF7,SPRED1,CTGF,MAPK1,PEA15,FBXW11,SOS1
GO: Biological Process	GO:0044744	protein targeting to nucleus	1.643E-3	8	289	SPPL3,DAG1,SIRT1,BMPR1A,PPP3CA,PIK3R1,MAPK1,FBXW11
GO: Biological Process	GO:0006606	protein import into nucleus	1.643E-3	8	289	SPPL3,DAG1,SIRT1,BMPR1A,PPP3CA,PIK3R1,MAPK1,FBXW11
GO: Biological Process	GO:1902593	single-organism nuclear import	1.643E-3	8	289	SPPL3,DAG1,SIRT1,BMPR1A,PPP3CA,PIK3R1,MAPK1,FBXW11
GO: Biological Process	GO:0000902	cell morphogenesis	1.694E-3	23	1574	KRAS,DPYSL3,MYCBP2,MAPT,HBEGF,DUSP9,DAG1,SRGAP2,NRN1,ZEB2,PPP3CA,SEMA6D,CELSR3,NFIB,OLFM1,FGF7,SPRED1,GNA12,SSH2,MAPK1,PEA15,BRWD1,SOS1
GO: Biological Process	GO:0006479	protein methylation	1.730E-3	6	167	ARID4B,MECP2,SIRT1,WHSC1L1,BTG2,KMT5B
GO: Biological Process	GO:0008213	protein alkylation	1.730E-3	6	167	ARID4B,MECP2,SIRT1,WHSC1L1,BTG2,KMT5B
GO: Biological Process	GO:0071417	cellular response to organonitrogen compound	1.770E-3	13	670	KRAS,HBEGF,DUSP9,SIRT1,PIK3R1,OSBPL8,FGF7,SPRED1,MAPK1,GNB1,PEA15,DNMT3A,SOS1
GO: Biological Process	GO:1901701	cellular response to oxygen-containing compound	1.791E-3	18	1107	SOX4,KRAS,HBEGF,CDK19,DUSP9,TEAD1,ZNF236,SIRT1,PPP3CA,PIK3R1,OSBPL8,FGF7,SPRED1,MAPK1,GNB1,PEA15,DNMT3A,SOS1
GO: Biological Process	GO:0051170	nuclear import	1.791E-3	8	293	SPPL3,DAG1,SIRT1,BMPR1A,PPP3CA,PIK3R1,MAPK1,FBXW11
GO: Biological Process	GO:0016311	dephosphorylation	1.792E-3	10	435	CTDSPL2,DUSP9,SPPL3,ZFYVE1,PTPN4,PPP3CA,CSRNP3,SPRED1,SSH2,FBXW11
GO: Biological Process	GO:0060322	head development	1.849E-3	14	757	KRAS,ZNF148,ABR,BCL2L11,MAPT,SRGAP2,MECP2,ZEB2,BMPR1A,BTG2,SEMA6D,NFIB,MAPK1,SOS1
GO: Biological Process	GO:0043408	regulation of MAPK cascade	1.856E-3	15	843	KRAS,TRIB2,HBEGF,KSR2,DUSP9,DAG1,ZEB2,CRK,MAP3K3,FGF7,SPRED1,CTGF,MAPK1,PEA15,SOS1
GO: Biological Process	GO:0035295	tube development	1.889E-3	13	675	SOX4,KRAS,BCL2L11,DAG1,TEAD1,SIX4,ZEB2,BMPR1A,NFIB,FGF7,CTGF,MAPK1,SMAD5
GO: Biological Process	GO:0051173	positive regulation of nitrogen compound metabolic process	1.905E-3	26	1887	SOX4,KRAS,TCF4,ZNF148,FMR1,ARID4B,KDM5A,TEAD1,SIX4,MECP2,ZEB2,SIRT1,MEIS2,BMPR1A,BTG2,PPP3CA,CSRNP3,NFIB,PIK3R1,FUBP1,ARID2,NFYA,FGF7,MAPK1,SMAD5,FBXW11
GO: Biological Process	GO:0043523	regulation of neuron apoptotic process	1.926E-3	7	231	KRAS,TRIM2,BCL2L11,SIX4,MECP2,SIRT1,BTG2
GO: Biological Process	GO:0045935	positive regulation of nucleobase-containing compound metabolic process	1.928E-3	25	1788	SOX4,KRAS,TCF4,ZNF148,ARID4B,KDM5A,TEAD1,SIX4,MECP2,ZEB2,SIRT1,MEIS2,BMPR1A,BTG2,PPP3CA,CSRNP3,NFIB,PIK3R1,FUBP1,ARID2,NFYA,FGF7,MAPK1,SMAD5,FBXW11
GO: Biological Process	GO:0002764	immune response-regulating signaling pathway	1.939E-3	13	677	KRAS,HBEGF,DUSP9,SPPL3,CRK,PPP3CA,PIK3R1,FGF7,SPRED1,MAPK1,PEA15,FBXW11,SOS1
GO: Biological Process	GO:0014883	transition between fast and slow fiber	1.984E-3	2	9	TEAD1,PPP3CA
GO: Biological Process	GO:0034770	histone H4-K20 methylation	1.984E-3	2	9	ARID4B,KMT5B
GO: Biological Process	GO:0035562	negative regulation of chromatin binding	1.984E-3	2	9	PPP3CA,MEPCE
GO: Biological Process	GO:0007548	sex differentiation	1.990E-3	8	298	ARID4B,BCL2L11,SIX4,SIRT1,CSDE1,BMPR1A,FGF7,SMAD5
GO: Biological Process	GO:0007173	epidermal growth factor receptor signaling pathway	1.996E-3	9	368	KRAS,HBEGF,DUSP9,PIK3R1,FGF7,SPRED1,MAPK1,PEA15,SOS1
GO: Biological Process	GO:0009967	positive regulation of signal transduction	2.005E-3	22	1497	SOX4,KRAS,BCL2L11,HBEGF,KSR2,DUSP9,SPPL3,SHOC2,ZEB2,SIRT1,CRK,MAP3K3,BMPR1A,PIK3R1,OSBPL8,FGF7,SPRED1,CTGF,MAPK1,PEA15,HOMER1,SOS1
GO: Biological Process	GO:0021955	central nervous system neuron axonogenesis	2.148E-3	3	34	MYCBP2,ZEB2,NFIB
GO: Biological Process	GO:0009891	positive regulation of biosynthetic process	2.257E-3	26	1910	SOX4,KRAS,TCF4,ZNF148,FMR1,ARID4B,KDM5A,TEAD1,SIX4,MECP2,ZEB2,SIRT1,MEIS2,BMPR1A,PPP3CA,CSRNP3,NFIB,PIK3R1,FUBP1,ARID2,NFYA,FGF7,CTGF,MAPK1,SMAD5,FBXW11
GO: Biological Process	GO:0038127	ERBB signaling pathway	2.266E-3	9	375	KRAS,HBEGF,DUSP9,PIK3R1,FGF7,SPRED1,MAPK1,PEA15,SOS1
GO: Biological Process	GO:0046824	positive regulation of nucleocytoplasmic transport	2.377E-3	5	122	CTDSPL2,SPPL3,BMPR1A,PPP3CA,PIK3R1
GO: Biological Process	GO:1900182	positive regulation of protein localization to nucleus	2.377E-3	5	122	HNRNPM,SPPL3,BMPR1A,PPP3CA,PIK3R1
GO: Biological Process	GO:0060284	regulation of cell development	2.384E-3	16	954	TCF4,DPYSL3,MAPT,DAG1,SRGAP2,TEAD1,NREP,MECP2,ZEB2,SIRT1,BMPR1A,PPP3CA,SEMA6D,NFYA,OLFM1,SSH2
GO: Biological Process	GO:0010558	negative regulation of macromolecule biosynthetic process	2.424E-3	22	1520	TCF4,TRIB2,ZNF148,FMR1,KDM5A,HBEGF,CC2D1B,FOXN3,SIX4,MECP2,ZEB2,SIRT1,MEIS2,WHSC1L1,FXR1,BTG2,NFIB,PURB,AEBP2,MEPCE,FBXW11,DNMT3A
GO: Biological Process	GO:0060502	epithelial cell proliferation involved in lung morphogenesis	2.467E-3	2	10	NFIB,FGF7
GO: Biological Process	GO:0014032	neural crest cell development	2.535E-3	4	75	ZEB2,BMPR1A,SEMA6D,MAPK1
GO: Biological Process	GO:2000736	regulation of stem cell differentiation	2.641E-3	5	125	DAG1,TEAD1,SIX4,NFYA,OLFM1
GO: Biological Process	GO:0009888	tissue development	2.663E-3	26	1933	SOX4,KRAS,MIA3,RAPGEF6,ARID4B,HBEGF,DAG1,TEAD1,SIX4,ZEB2,MEX3C,TGFBI,BMPR1A,BTG2,PPP3CA,SEMA6D,NFIB,BTBD7,OLFM1,FGF7,CTGF,MAPK1,HOMER1,SMAD5,SOS1,LARGE
GO: Biological Process	GO:0014031	mesenchymal cell development	2.737E-3	6	183	DAG1,ZEB2,BMPR1A,SEMA6D,OLFM1,MAPK1
GO: Biological Process	GO:0000122	negative regulation of transcription from RNA polymerase II promoter	2.762E-3	14	791	TCF4,ZNF148,KDM5A,CC2D1B,MECP2,ZEB2,SIRT1,MEIS2,BTG2,NFIB,PURB,AEBP2,MEPCE,DNMT3A
GO: Biological Process	GO:0045892	negative regulation of transcription, DNA-templated	2.855E-3	19	1250	TCF4,TRIB2,ZNF148,KDM5A,CC2D1B,FOXN3,SIX4,MECP2,ZEB2,SIRT1,MEIS2,WHSC1L1,BTG2,NFIB,PURB,AEBP2,MEPCE,FBXW11,DNMT3A
GO: Biological Process	GO:0007420	brain development	2.892E-3	13	709	KRAS,ZNF148,ABR,BCL2L11,MAPT,SRGAP2,MECP2,ZEB2,BMPR1A,BTG2,SEMA6D,NFIB,SOS1
GO: Biological Process	GO:0090311	regulation of protein deacetylation	2.962E-3	3	38	KDM5A,SIRT1,SPRED1
GO: Biological Process	GO:0042306	regulation of protein import into nucleus	2.966E-3	6	186	SPPL3,SIRT1,BMPR1A,PPP3CA,PIK3R1,FBXW11
GO: Biological Process	GO:2001223	negative regulation of neuron migration	3.000E-3	2	11	SRGAP2,OLFM1
GO: Biological Process	GO:2001275	positive regulation of glucose import in response to insulin stimulus	3.000E-3	2	11	PIK3R1,OSBPL8
GO: Biological Process	GO:0007016	cytoskeletal anchoring at plasma membrane	3.000E-3	2	11	DAG1,TLN2
GO: Biological Process	GO:0051533	positive regulation of NFAT protein import into nucleus	3.000E-3	2	11	SPPL3,PPP3CA
GO: Biological Process	GO:1902237	positive regulation of endoplasmic reticulum stress-induced intrinsic apoptotic signaling pathway	3.000E-3	2	11	BCL2L11,SIRT1
GO: Biological Process	GO:0060693	regulation of branching involved in salivary gland morphogenesis	3.000E-3	2	11	BTBD7,FGF7

Supplementary Table S5

insoluble Aβ42 - miR-132

	R square	P value
**Ctrl**	0.141	0.229
**MCI**	0.02766	0.625
**AD**	0.1729	0.1789
**All**	0.1328	0.0314

insoluble Aβ42 - miR-212

	R square	P value
**Ctrl**	0.1701	0.1827
**MCI**	0.09083	0.3411
**AD**	0.2846	0.091
**All**	0.1907	0.0087

amyloid plaques - miR-132

	R square	P value
**Ctrl**	0.03043	0.5876
**MCI**	0.01056	0.7637
**AD**	0.2269	0.1175
**All**	0.1282	0.0347

amyloid plaques-miR-212

	R square	P value
**Ctrl**	0.04521	0.507
**MCI**	0.04117	0.5271
**AD**	0.1988	0.1463
**All**	0.1458	0.0216

Sirt1-miR-132

	R square	P value
**Ctrl**	0.000715	0.9456
**MCI**	0.2251	0.1969
**AD**	0.2118	0.1808
**All**	0.1526	0.0398

Sirt1-miR-212

	R square	P value
**Ctrl**	0.03451	0.5632
**MCI**	0.03584	0.5557
**AD**	0.01994	0.6788
**All**	0.000483	0.9003

insoluble Aβ42 - Sirt1

	R square	P value
**Ctrl**	0.002502	0.8983
**MCI**	0.1655	0.1893
**AD**	0.2331	0.1576
**All**	0.1099	0.0685
